# Genome- and Transcriptome-Wide Characterization and Expression Analyses of bHLH Transcription Factor Family Reveal Their Relevance to Salt Stress Response in Tomato

**DOI:** 10.3390/plants14020200

**Published:** 2025-01-12

**Authors:** Jianling Zhang, Xiaoying Liu, Zuozhen Yin, Tiantian Zhao, Dan Du, Jing Li, Mingku Zhu, Yueying Sun, Yu Pan

**Affiliations:** 1 Laboratory of Plant Germplasm Resources Innovation and Utilization, College of Agriculture and Biology, Liaocheng University, Liaocheng 252000, China; liuxiaoying0921@163.com (X.L.); yinzuozhen331@163.com (Z.Y.); zhaotiantian2526@163.com (T.Z.); syysun@aliyun.com (Y.S.); 2 College of Horticulture and Landscape Architecture, Southwest University, Chongqing 400715, China; dudan199009@163.com; 3 Chongqing Academy of Agricultural Sciences, Chongqing 850030, China; micy180605@163.com; 4 School of Life Sciences, Jiangsu Normal University, 101 Shanghai 16 Road, Xuzhou 221008, China; mingkuzhu007@126.com

**Keywords:** genome-wide analysis, transcriptome characterization, stress-responsive gene expression, plant tolerance, salt stress, bHLH transcription factors, tomato

## Abstract

The bHLH (basic helix–loop–helix) transcription factors function as crucial regulators in numerous biological processes including abiotic stress responses and plant development. According to our RNA-seq analysis of tomato seedlings under salt stress, we found that, although the bHLH gene family in tomato has been studied, there are still so many tomato bHLH genes that have not been identified and named, which will hinder the later study of *SlbHLHs*. In total, 195 *SlbHLHs* that were unevenly distributed onto 12 chromosomes were identified from the tomato genome and were classified into 27 subfamilies based on their molecular features. The collinearity between SlbHLHs and interrelated orthologs from 10 plants further revealed evolutionary insights into *SlbHLHs*. Cis-element investigations of *SlbHLHs* promotors further suggested the potential roles of *SlbHLHs* in tomato development and stress responses. A total of 30 *SlbHLHs* were defined as the differentially expressed genes in response to salt stress by RNA-seq. The expression profiles of selected *SlbHLHs* were varyingly and markedly induced by multiple abiotic stresses and hormone treatments. These results provide valuable information to further understand the significance and intricacy of the bHLH transcription factor family, and lay a foundation for further exploring functions and possible regulatory mechanisms of SlbHLH members in abiotic stress tolerance, which will be significant for the study of tomato stress resistance and agricultural productivity.

## 1. Introduction

Transcription factors (TFs) are crucial regulators identified in eukaryotes that take part in the adjustments of different signal transduction pathways and modify the transcript levels of diverse and numerous development- and stress-related genes via the interaction with cis-elements, such as MADS-box, bHLH, NAC, bZIP, WRKY, ERF, and GRAS. These TFs can help plants to mediate adaptations to different developmental stages and environmental stresses. Among them, the basic helix–loop–helix (bHLH) TFs have been widely studied in almost all eukaryotes with pleiotropic regulatory functions [1]. All the bHLH transcription factors share a conserved domain that comprises approximately 50 to 60 amino acids and forms two linked subregions (the helix–loop–helix (HLH) region and the basic region) [2]. The basic region which is located in the N-terminus of the bHLH domain contains 10 to 15 predominantly basic amino acids. It is a DNA-binding region that can bind to the E-box cis-element (5′-CANNTG-3′) of specific genes [3]. The binding of bHLH transcription factors to the E-box has been reported to control gene expression levels in multiple biological processes. The HLH region is located at the C-terminus of the bHLH domain and comprises around 40 to 50 amino acids. It is composed of two amphiphilic α-helices with hydrophobic residues linked by a loop of variable length and acts as a dimerization domain [3]. Structural studies of bHLH proteins in mammals and yeast indicated that the basic region is the main interface where the DNA contaction occurs, whereas these two helices promote bHLH proteins to form homo- or heterodimers, which is a precondition for DNA binding [4,5]. In addition, some bHLH proteins were found to have an additional MYC domain located at the N-terminus of proteins. In plants, Myc-bHLH protein harbors an MYB interaction region, which interacts with the R2R3-MYB protein to control gene transcript levels and downstream processes [6].

The bHLH transcription factors (TFs) are one of the largest TFs families in plants, and they have been widely revealed to play significant roles in the processes of multiple biological and physiological processes, including diverse plant growth and development and various environmental stresses [7]. Previous studies have reported that bHLH TFs regulate different developmental processes of various vegetative and reproductive tissues. For instance, increased transcripts of an atypical bHLH gene *SlPRE2* in tomato results in rolling leaves with reduced chlorophyll content and increased stem internode length, while the upregulation of *SlPRE2* leads to decreased fruits and placenta size and thin pericarp [8,9]. The overexpression of a bHLH transcription factor gene, *SlbHLH22*, promotes early flowering, accelerates fruit ripening, enhances fruit sensitivity to exogenous phytohormones, and shortens fruit shelf-life in tomato [10,11]. The bHLH transcription factor SlbHLH95 affects fruit ripening and multiple metabolisms, and the overexpression of *SlbHLH95* impacts trichome formation via regulating gibberellin biosynthesis in tomato [12,13]. The knockout of the *SlMS10* gene encoding the bHLH transcription factor using the CRISPR/Cas9 system confers the male sterility phenotype in tomato [14]. Two bHLH genes *AtRHD6* and *AtRSL1* control the development of root hair in Arabidopsis [15]. The loss of function of a bHLH transcription factor MUTE in Arabidopsis generates small, pale, and sterile plants with the complete absence of stomata [16]. The bHLH factor GL3 regulates the initiation of leaf trichome in Arabidopsis [17]. Two functionally diverging and partially redundant bHLH genes, *SPATULA* and *ALCATRAZ*, are required for gynoecium and fruit development in Arabidopsis [18,19]. The other three bHLH transcription factors, ABORTED MICROSPORES (AMS), BIGPETALp, and SPATULA, regulate the development of pollen [20], petal [21], and carpel [22], respectively. And, GhPRE1A can promote the elongation of cotton fiber by activating the bHLH factor GhPAS1 [23].

Meanwhile, some bHLH TFs also take part in the regulation of multifarious anabolic pathways and signal transductions, including flavonoid, anthocyanin, and tryptophan synthesis and light and hormone signal transduction. For instance, the bHLH protein MdbHLH3 in apples promotes fruit coloration and anthocyanin accumulation in response to low temperature [24]. The overexpression of tomato *SlbHLH22* transcription factor gene enhances fruit sensitivity to exogenous phytohormones and shortens fruit shelf-life, and also promotes early flowering and accelerates fruit ripening in tomato [10,11]. AtbHLH42 (TT8) regulates the biosynthesis of anthocyanin and procyanidin in vegetative organs in Arabidopsis [25]. Arabidopsis bHLH protein ATR2 activates the tryptophan pathway [26]. The bHLH protein AtPIF4 interacts with phytochrome B to regulate the transcripts of genes that are involved in regulating light response [27]. The HFR1, an atypical bHLH transcription factor, plays crucial roles in the signal transduction of phytochrome A via heterodimerization with PIF3 in Arabidopsis [28]. Three closely related bHLH proteins, BEE1, BEE2, and BEE3, through functional redundancy, positively regulate brassinosteroid signaling in Arabidopsis [29]. The bHLH protein INDEHISCENT (IND) in Arabidopsis controls gibberellin signal transduction by directly activating GA3ox1, which is a key gene in gibberellin biosynthesis [30]. The bHLH transcription factors also participate in various abiotic stress responses. For example, two bHLH proteins MYC2 in Arabidopsis and CabHLH035 in pepper are involved in the salt response [31,32]. The transcription factor AtbHLH68 has crucial functions in regulating the resistance to drought stress in Arabidopsis [33]. Two bHLH genes *MdbHLH3* and *MdCIbHLH1* play significant roles in regulating the cold stress response in apple [24,34]. A bHLH transcription factor, SlbHLH96, promotes drought tolerance in tomato [35]. Rice OsbHLH148 takes part in trauma response and drought stress and is involved in jasmonic acid (JA) signaling transduction [36]. The loss of function of the bHLH transcription factor Nrd1 in tomato enhances resistance to *Pseudomonas syringae* [37]. The transcripts of AtbHLH92 are dramatically induced in response to salt, drought, and cold stresses [38].

The diversity of bHLH proteins have been classified as numerous distinct groups according to phylogenetic analyses. Based on the functional properties, DNA binding specificity, and phylogenetic relationships, plant bHLHs are classified into 12–22 subgroups at the beginning [39,40,41]. Then, 27 subfamilies are defined to represent deep evolutionary relationships among plant bHLH proteins following the method described by Pires and Dolan [2]. Although the members of tomato bHLH transcription factor family have been identified earlier [42], some new tomato bHLH genes, which have not been identified in previous studies, have been reported to play significant roles in tomato development, such as *SlPRE2* (Solyc02g067380) [8,9,43], *SlPRE3* (Solyc06g070910) [44], and *SlPRE5* (Solyc04g005660) [45], and so on. Therefore, the genome-wide identification and characterization of bHLH TFs in tomato is lagging. The systematical and in-depth genome-wide identification of tomato bHLHs needs to be studied to identify more bHLH genes in tomato.

Abiotic stresses like salinity, cold, high temperature, and drought are major hurdles in crop productivity, significantly influencing development, plant growth, and food crop yields [46]. To cope up with abiotic stresses, it is essential to comprehensively investigate the roles of important gene family members in response to abiotic stress in plants. Tomato (*Solanum lycopersicum*) is one of the most economically valuable and widely cultivated vegetable crops in the world and has great potential for application. Tomato has rich genetic diversity. Due to its small genome size, short life cycle, high-density genetic maps, efficient stable transformation, and the completion of the tomato genome sequence, as well as many well-characterized tomato mutants, tomato is also considered as an excellent model plant for investigation. The long-term exposure of tomato plants to adverse environmental conditions such as high salt, high temperature, cold, and drought has a significant impact on their quality and productivity. The investigations of stress resistance in tomato are significant for scientific investigations and agricultural production, and they provide an important theoretical basis and genetic resources for the molecular breeding of tomato. Therefore, the identification and characterization of excellent candidate gene family members in response to stress tolerance not only lays a foundation for further exploring functions and possible regulatory mechanisms of these candidates in regulating tomato tolerance to abiotic stresses, but will also be significant for the study of plant stress resistance and agricultural productivity.

In this study, 195 bHLH genes were identified from the tomato genome using newer software and websites, and these *SlbHLHs* were named and classified into 27 subfamilies following the method described by Pires and Dolan [2]. In addition, the RNA-seq was performed to screen genes that respond to salt stress in tomato roots. In total, 30 tomato *SlbHLHs,* whose expression was significantly up- or downregulated, were defined as the differentially expressed genes (DEGs). The molecular characterization, chromosomal location, phylogenetic relationships, conserved domain, structural feature, motif, and collinearity investigation of *SlbHLHs* were studied. Moreover, the expression patterns of multiple *SlbHLHs* under various abiotic stresses and phytohormones were surveyed. The comprehensive characterization of tomato *SlbHLHs* provides empirical foundation for the further functional study of *SlbHLHs* in response to stress tolerance, and the identification of important bHLH TF families will provide an understanding of plant adaptive mechanisms of environmental stresses.

## 2. Results

### 2.1. Identification and Characterization of Tomato SlbHLH Genes

**Figure 1:**
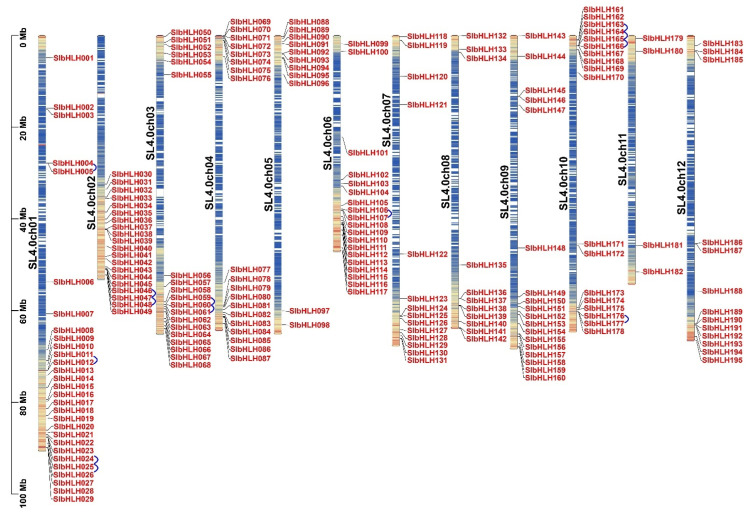
Localizations of 195 *SlbHLHs* in tomato chromosomes 1–12. Chromosomal map showing the uneven distribution of 195 *SlbHLHs* on 12 tomato chromosomes. The chromosome numbers are indicated to the left of each chromosome as Chr01–Chr12. The scales indicate the genome size of the sweet potato genome (Mb). The blue arcs behind some *SlbHLHs* represent the gene duplication of part of the bHLH genes in tomato.

In this study, the known bHLH proteins in rice and Arabidopsis were used as inquire sequences to screen all the possible bHLH TFs via the BLASTP program. Finally, a total of 195 putative non-redundant bHLH family members were identified from the known tomato genome database (SL4.0, Sol Genomics Network, https://solgenomics.net/organism/Solanum_lycopersicum/genome, 15 June 2024). Subsequently, these genes were named bHLH001-bHLH195 following their position on tomato 12 chromosomes (Figure 1). After that, the molecular weight, protein size (aa), phosphorylation site, and theoretical isoelectric point (pI) of 195 SlbHLH proteins were studied. The detailed results are listed in Table S1. The molecular weight and length of SlbHLHs varied widely, with molecular weight ranging from 9.80008 KDa (SlbHLH091) to 102.05852 KDa (SlbHLH182), and the length varied from 86 aa (SlbHLH091) to 930 aa (SlbHLH182). The theoretical pI ranged from 4.65 (SlbHLH079) to 11.45 (SlbHLH007). The subcellular location prediction displayed that most *SlbHLHs* were located in the nucleus, and very few *SlbHLHs* were located in the chloroplast and/or golgi apparatus. The prediction of the phosphorylation site of SlbHLHs showed significant variations from 13 (SlbHLH127) to 190 (SlbHLH182), and most SlbHLHs contained more Ser sites (8–142) than Thr (0–22) and Tyr (0–53) sites (Table S1). In addition, over 90.8% of the SlbHLHs contained at least 30 phosphorylation sites.

### 2.2. Chromosome Distribution of Tomato SlbHLH Genes

The chromosome localization investigation exhibited that the 195 *SlbHLHs* were mapped on all 12 chromosomes of tomato. Among these chromosomes, Chr 1 and Chr 2 contained the most abundant SlbHLH genes, with 29 and 20 members, respectively. However, Chr 11 only contained 4 *SlbHLHs*, and the rest of the chromosomes (Chr 5, Chr 7, Chr8, Chr 12) contained 11–14 *SlbHLHs* (Figure 1). The interesting thing was that the chromosome Chr 6 contained 19 *SlbHLHs*, while part of the larger chromosomes (Chr 5, Chr8, Chr 11) contained fewer *SlbHLHs* (11, 11, 4, respectively). In addition, most of the *SlbHLHs* were located at the ends of tomato chromosomes (Figure 1). These data indicated that the distribution of *SlbHLHs* was relatively clustered and uneven among tomato chromosomes and was also disproportionate to chromosome size.

### 2.3. Phylogenetic Relationship of SlbHLH Proteins in Tomato

**Figure 2:**
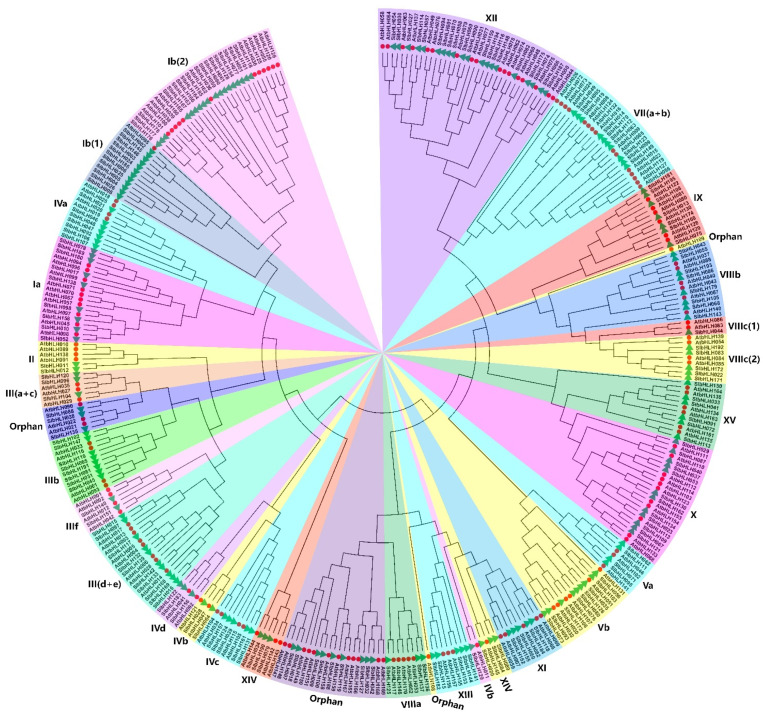
Phylogenetic tree of 195 tomato SlbHLHs and 169 Arabidopsis AtbHLHs. The phylogenetic relationships were analyzed using the MEGA X software (version 10) using the Maximum Likelihood method. The phylogenetic tree was calculated with the selected best WGA + G + F evolutionary model via MEGA-X with 1000 bootstrap value and partial deletions. These 364 bHLH proteins were classified into 27 subfamilies according to the classification method described by Pires et al. [2]. Different subfamilies are named using the Greek numerals following the studies in Arabidopsis, and different colors were used to distinguish each subfamily; “orphan” indicates that these SlbHLHs proteins are not included in any bHLH subfamilies and have a high degree of sequence divergence from other bHLHs, likely representing highly diverged lineage-specific genes. Red circles and green triangles represent tomato SlbHLHs and Arabidopsis AtbHLHs, respectively.

According to the topology of the tree, clade support values, and the classification of Arabidopsis AtbHLHs in a previous study [2], the phylogenetic analysis showed that these 195 tomato and 169 Arabidopsis bHLHs proteins were divided into 27 subfamilies (Figure 2). The number of SlbHLH members included in the 27 subfamilies ranged from 1 to 20, with subfamily XII containing the most SlbHLHs (20 members) and with only one SlbHLH in subfamily VIIIc (1). As for certain subfamilies, the bHLH TF family displayed obvious species bias in the time of evolution, suggesting that a specific gene duplication happened after species differentiation. Simultaneously, some genes exhibited one-to-one homology, for instance, subfamily IVb (AtbHLH011 and SlbHLH129, AtbHLH047 and SlbHLH064, AtbHLH121 and SlbHLH028), subfamily III (a+c) (AtbHLH029 and SlbHLH104), subfamily IIIf (AtbHLH042 and SlbHLH151), and so on (Figure 2). The member differences between AtbHLHs and SlbHLHs clustered in the same subfamily suggested that the bHLH gene family had apparent interspecific divergences between Arabidopsis and tomato.

### 2.4. Gene Duplication Survey of Tomato SlbHLH Genes

**Figure 3:**
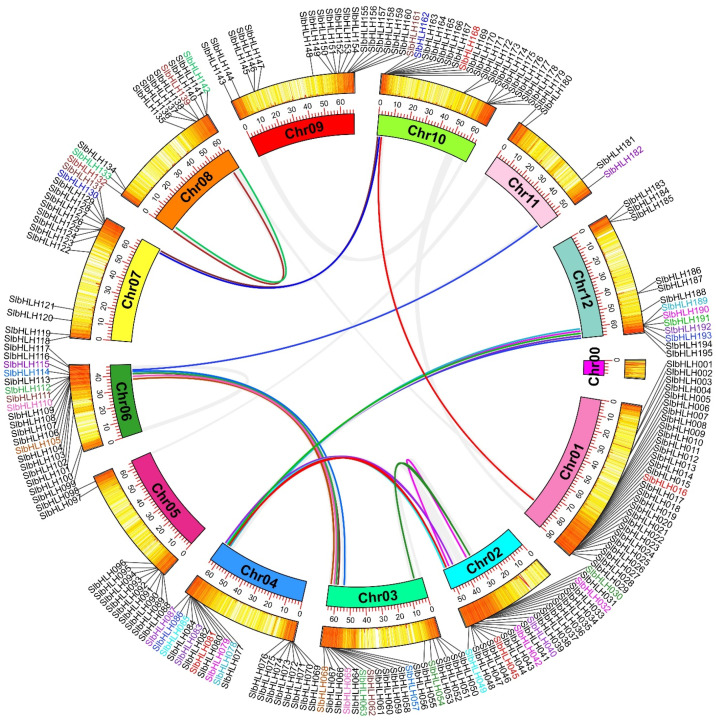
Segmental duplications and localizations of *SlbHLHs* in tomato chromosomes. A total of 195 *SlbHLHs* are located on all 12 chromosomes of tomato. Chr01–Chr12 are represented by different colored rectangles. The heatmap and polyline along each rectangle depict the gene density of each chromosome. Duplicated SlbHLH gene pairs in tomato chromosomes are indicated by colored lines, and these corresponding genes are also marked with colors. The black color was used to mark other SlbHLH genes that show no collinear relationships.

Genome duplication plays significant roles in the process of evolutions and the expansions of plant gene families [47]. The results of the collinear analysis showed that 13 tandem duplications among 195 *SlbHLHs* were found (Figure 1, Table 1 and Table S3-1). And, these SlbHLH genes displaying tandem repeats came from the same subfamily. Moreover, the MCScanX programs and BlastP were performed to find the segmental duplications and 21 gene pairs were found on 10 of 12 chromosomes (Figure 3, Table 1 and Table S3-2). These results showed that some chromosomes (Chr02, Chr04, Chr06, and Chr12) had more linkage groups than others. Analogously, these linked *SlbHLHs* were linked inside their subfamilies. However, no fragment duplication of SlbHLH gene pairs was detected on Chr05 and Chr09 (Figure 3). These results indicated that gene duplication events contributed to the expansion of *SlbHLHs*.

### 2.5. Collinearity Analysis of SlbHLHs Between Tomato and Other Plants

**Figure 4:**
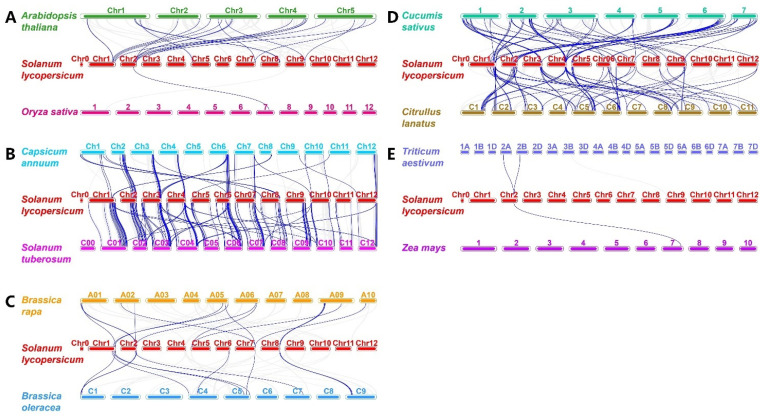
Synteny analyses of tomato SlbHLHs with 10 representative plants. These plants include *Arabidopsis thaliana* and *Oryza sativa* (**A**), *Capsicum annuum* and *Solanum tuberosum* (**B**), *Brassica rapa* and *Brassica oleracea* (**C**), *Cucumis sativus* and *Citrullus lanatus* (**D**), and *Triticum aestivum* and *Zea mays* (**E**). The chromosomes of each plant are distinguished using different colors. The blue lines linking two different chromosomes represent the syntenic bHLH gene pairs in the genomes of tomato and other plants.

The results of collinearity relationships between *SlbHLHs* and the orthologous genes of 10 representative plants showed that a total of 141 (72.3%) *SlbHLHs* shared collinearity relations with those in *Solanum tuberosum*, followed by *Capsicum annuum* (68), *Cucumis sativus* (63), *Citrullus lanatus* (61), *Arabidopsis thaliana* (20), *Brassica rapa* (12), *Brassica oleracea* (11), *Zea mays* (1), *Triticum aestivum* (1), and *Oryza sativa* (1) (Figure 4). Therefore, the most collinearity relationships existed between tomato *SlbHLHs* and *Solanum tuberosum* genes, indicating a closer relationship between tomato and potato. Moreover, our results also showed that many SlbHLH genes had syntenic relationships with between two and three genes of other plants, especially with *Solanum tuberosum* (Table S4). Analogously, the gene pairs where between two and three *SlbHLHs* had the same collinearity as the orthologous gene of the twelve tested plants were also observed (Table S4). These results suggested that some orthologous genes may be derived from a common ancestor of these plants.

### 2.6. Motif Composition Analysis of SlbHLH Proteins in Tomato

The motif composition analysis of SlbHLH proteins showed that a total of 30 conserved motifs were identified from 195 SlbHLHs using the MEME tool on the basis of settings in Arabidopsis (motifs 1–30; Figure S1). In general, the SlbHLHs in the same subfamily exhibited similar motif compositions, which further consolidated the subfamily classification. The results exhibited that the motif number of each SlbHLH ranged from 1 to 9, which also showed differences in motif composition. Motifs 1 and 2 were widely distributed in most SlbHLHs, and other motifs only existed in certain members. Universally, members in the same subfamily harbored similar motif structures. For instance, all members in subfamilies VIIIc, XI, and IVc contained motifs 1, 2, 3, motifs 1, 2, 3, 9, and motifs 1, 2, 15, 19, respectively. Moreover, some members in the same subfamily also had specific motifs in addition to their common motifs, and family members in different subfamilies also shared different specific motifs in their number and compositions (Figure S1). These data indicated that the number and compositions vary significantly in different bHLH subfamilies, and the specific motifs may indicate diverse and distinct functions of *SlbHLHs* in tomato.

### 2.7. Conserved Domain and Gene Structure Analyses of SlbHLHs

**Figure 5:**
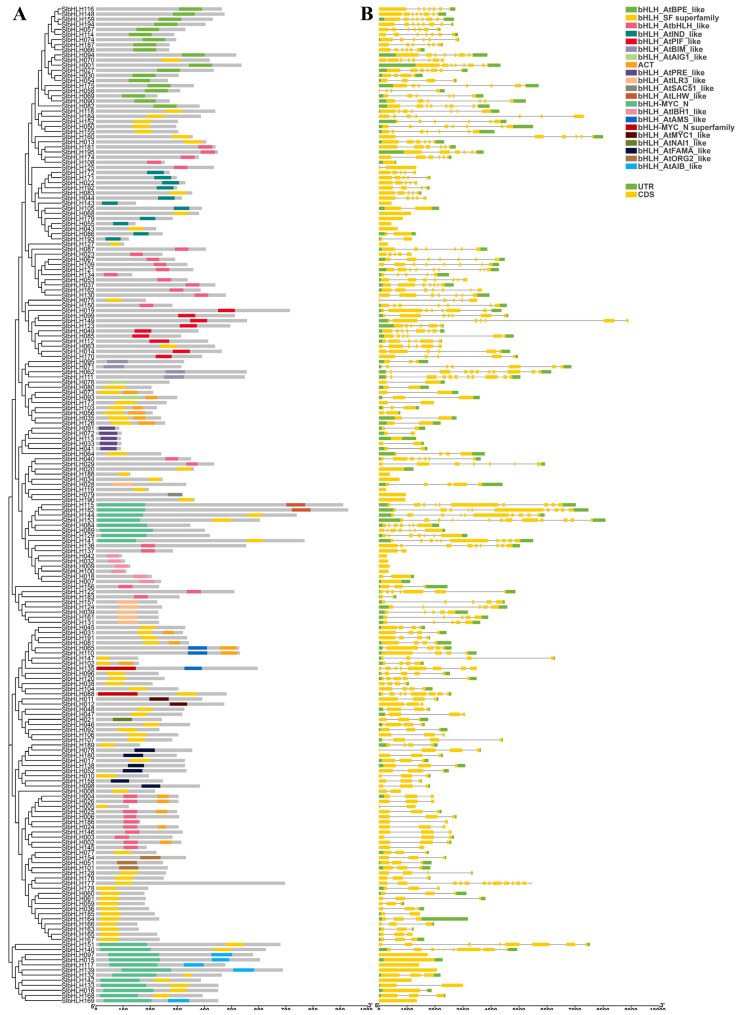
Conserved domain distributions and gene structures of 195 tomato *SlbHLHs*. (**A**) The phylogenetic tree and conserved domain distributions of tomato *SlbHLHs*. The phylogenetic tree of *SlbHLHs* with the same parameters as in Figure 2 was constructed by MEGA X. The CD-search in the NCBI database was used to detect the distributions of the conserved domains of *SlbHLHs*. The different colorful boxes present different conserved domains. (**B**) Gene structures of 195 tomato *SlbHLHs*. UTR and exons are represented by green and yellow bars, respectively. The introns are indicated by black lines. The scale at the bottom is used to estimate the length of SlbHLH proteins and genes.

Conserved domain detection by Batch CD-Search showed that 20 different forms of bHLH domains with higher diversity were detected, and all SlbHLHs share one or two conserved bHLH domains. In the same subfamilies, the conserved bHLH domain containing in the most SlbHLHs had similar location distribution and gene members. And, these SlbHLHs harbored similar bHLH domain types with few exceptions. These results suggested that only the bHLH domain can distinctly establish the evolutionary relations among bHLH subfamilies. In addition, part of members of Vb, IIIb, and Ib (1) contained an additional ATG domain (Figure 5A).

Gene structure analysis by Tbtools (V2.120) software showed that the exon numbers of *SlbHLHs* markedly varied from 1 to 13, with 25 *SlbHLHs* having no introns and 25 *SlbHLHs* only containing 1 intron. *SlbHLH177* contained the biggest exon number (13), followed by *SlbHLH153* with 12 exons. Furthermore, our results showed that the majority of *SlbHLHs* in the same subfamily generally had similar gene structures and displayed similar exon lengths and intron numbers. For example, most *SlbHLHs* had three or four exons, five or six exons, and four exons in subgroups VIIIc (2), XI, and IVc, respectively. However, some *SlbHLHs* exhibited exceptions with differential gene structures in the same subfamilies, such as *SlbHLH177* in subfamily Ib (2) and *SlbHLH136* in subfamily VIIIa (Figure 5B). These results suggested that phylogenetic relationships among family members were mainly associated with conserved bHLH domains and gene structure.

### 2.8. Cis-Element Analysis in the SlbHLH Promoter Regions

**Figure 6:**
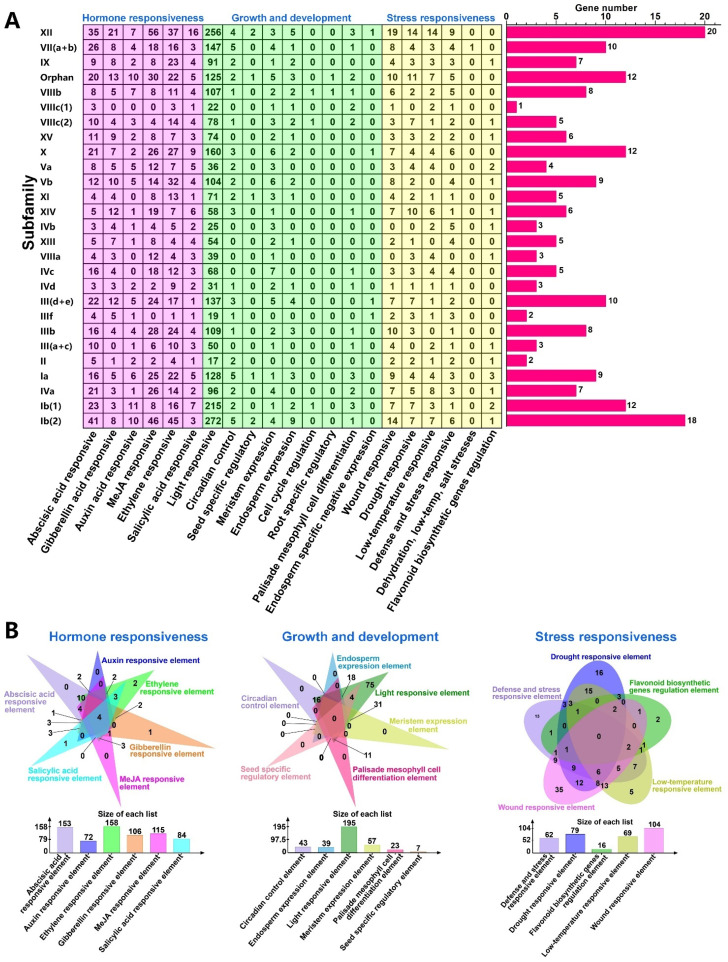
Predicted cis-elements in the promoters of 195 tomato *SlbHLHs*. (**A**). The number of cis-elements in the promoter of *SlbHLHs*. Red rectangles represent the gene number in each subfamily. The left table represents the number of each kind of cis-element detected in each subfamily. The red part indicates the hormone-responsiveness-related cis-elements. The green part indicates the growth- and development-related cis-elements. The yellow part indicates the stress-responsiveness-related cis-elements. (**B**). Venn diagram of various categories of cis-elements.

The cis-elements, which are non-coding DNA sequences in the gene promoter region, are crucial for gene transcription and are universally involved in regulating multiple biological processes [48]. To study the potential regulatory mechanism of *SlbHLHs* responding to development, abiotic stresses, and hormones, the cis-elements within the 2000bp upstream promoter regions of each SlbHLH gene were detected. The results showed that a total of 4799 cis-elements in the promoter sequence of SlbHLHs (Table S5-1) were predicted and 21 types of cis-elements were found (Figure S2, Table S5-1). Among them, *SlbHLH096* harbored a total of 44 cis-elements, which was the gene with the largest number of elements. The number of cis-elements varied in diverse subfamilies (Figure 6A) and all cis-elements can be categorized in three broad categories (Figure 6B).

The first category of cis-elements was relevant to hormone response (1554), including abscisic acid-responsive element (361, 7.52%), auxin-responsive element (93, 1.95%), gibberellin-responsive element (168, 3.52%), MeJA-responsive element (420, 8.79%), ethylene-responsive element (406, 8.50%), and salicylic acid-responsive element (106, 2.22%). The promoter sequences of all subfamilies of *SlbHLHs* abundantly harbored the elements that were in connection with ethylene-responsive (ERE), salicylic acid-responsive (TCA-element), and abscisic acid-responsive (ABRE) elements. Cis-elements (P-box, TATC-box, and GARE-motif) associated with the gibberellin response were nearly enriched in all subfamilies but absent from subfamilies VIIIc (1) and III (a+c). Cis-elements associated with auxin response (TGA-element, AuxRR-core, AuxRE, and TGA-box) were nearly enriched in all subfamilies but absent from subfamilies VIIIc (1) and VIIIa. Cis-elements associated with MeJA response (CGTCA-motif and TGACG-motif) were nearly enriched in all subfamilies except for the subfamilies VIIIc (1) and IIIf. In addition, in the promoter regions of some *SlbHLHs*, such as *SlbHLH015/017/023/029/070/096/110/147/148/175/195,* shared multiple identical hormone-responsive elements, indicating the possible that they had more intense and rapid responses to specific hormones. In the meantime, in the promoter regions of some *SlbHLHs*, such as *SlbHLH010/027/028/030/073/110/186/,* shared diverse hormone response elements, indicating their potential functions in numerous hormone regulatory networks (Figure S2 and Figure 6, Table S5-2).

The second category of cis-elements was associated with growth and development, including light-responsive element (2589, 53.95%), circadian control element (98, 2.04%), seed-specific regulatory element (7, 0.15%), meristem-expression-related element (73, 1.52%), endosperm-expression-related element (49, 1.02%), cell cycle regulation element (3, 0.06%), root-specific regulatory element (2, 0.04%), palisade-mesophyll-cell-differentiation-related element (25, 0.52%), and endosperm-expression-related element (49, 1.02%). Cis-elements related to light responsiveness (such as GA-motif, Box 4, G-box, chs-CMA1a, AE-box, TCT-motif, AT1-motif, GATA-motif, etc.) were presented in all *SlbHLH* promoters. Analogously, the cis-element related to the meristem expression element (CAT-box) also existed in nearly all *SlbHLHs* except for four genes in subfamilies IIIf and II. While other cis-elements involved in controlling growth and development were relatively insufficient, especially those elements associated with seed-specific regulation, palisade mesophyll cell differentiation, cell cycle regulation, root-specific regulation, and endosperm-specific negative expression. They were absent from numerous subfamilies such as RY-element for seed, MSA-like for cell cycle, motif I for root-specific regulation, HD-Zip 1 for palisade mesophyll cell differentiation, and AACA_motif for endosperm-specific negative expression (Figure S2 and Figure 6, Table S5-2).

The third category of cis-elements were related to stress responsiveness, including the wound-responsive element (151, 3.15%), drought-responsive element (112, 2.33%), low-temperature-responsive element (87, 1.81%), defense- and stress-responsive element (79, 1.65%), dehydration-, low-temp-, and salt-stress-related element (1, 0.02%), and flavonoid biosynthetic gene regulation element (18, 0.38%). Among them, cis-elements associated with stress and defense response (TC-rich repeats), low temperature (LTR), wound response (WUN-motif), and drought response (MBS) presented in almost all subfamilies, while the other two elements (dehydration and flavonoid biosynthetic gene regulation) only existed in a few subfamilies (Figure S2 and Figure 6, Table S5-2).

### 2.9. Protein Interaction Network Analysis of SlbHLHs in Tomato

Investigating the functional relationships of SlbHLH proteins is conducive to revealing their regulatory network. So, the protein interacting network of tomato SlbHLH proteins (Figure S3) based on the orthologous analysis of Arabidopsis AtbHLHs was investigated using the STRING software. In general, multiple members such as PRE1 (SlbHLH041), BIM2 (SlbHLH071/095), PYE (SlbHLH064), bHLH071 (SlbHLH138), bHLH093 (SlbHLH031/045/081/191), and AT5G50915 (SlbHLH057/114) had more interaction relationships than other members did. Members of bHLH proteins and their roles in Arabidopsis and their homologous in tomato are listed as follows (Table 2).

Therefore, the examination of the interactions of SlbHLH proteins indicated that many SlbHLH proteins tend to form complexes via protein interactions to play significant roles in controlling the development and/or stress resistance of plants. This serviceable information will be helpful in investigating the regulatory mechanism of SlbHLHs in regulating stress tolerances and in plant growth and development.

### 2.10. RNA-Seq of Tomato Roots in Response to Salt Stress

**Figure 7:**
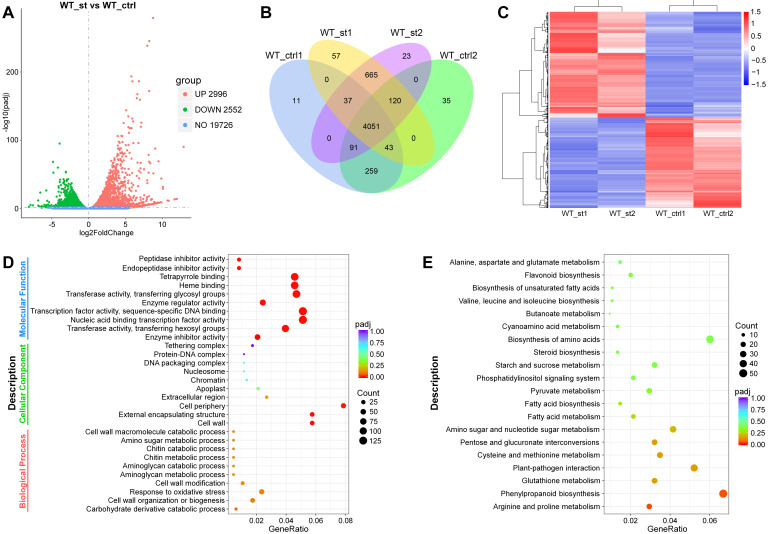
RNA-seq analysis of genes that respond to salt stress in tomato roots. (**A**) Volcano plot visualizing the differentially expressed genes (DEGs). The DEGs are shown in red and green. The *x*-axis represents the fold change in WT_st vs. WT_ctrl (on a log2 scale, WT_st indicates the experimental group where the WT tomato seedlings were treated with salt, the WT_ctrl indicates the control group with no treatment), and the *y*-axis represents the negative -log10-transformed *p*-values (*p* < 0.05) of the *t*-test for finding differences between the samples. (**B**) Venn diagram represents the DEGs detected in WT_st and WT_ctrl lines. Overlapping regions between WT_st and WT_ctrl lines indicate the DEGs that exist in these samples. (**C**) Hierarchical clustering analysis of salt-responsive DEGs in roots of WT_st and WT_ctrl lines. The red colors and blue colors indicate the induced and suppressed DEGs in the heat maps, respectively. (**D**) Gene ontology (GO) classification of DEGs that were enriched in cellular component (CC), biological process (BP), and molecular function (MF). (**E**) KEGG pathway enrichment analysis of up- and downregulated DEGs that were primarily enriched in the regulatory pathway. The copyright permission of KEGG image was obtained in this study.

Numerous members of various transcription factor families have been reported to be involved in regulating various abiotic stresses. The RNA-seq data exhibited that four cDNA libraries of each line were generated to be sequenced, and more than 31 million sequence reads of each cDNA library were yielded, representing > 9 Gb sequence data of each sample. Finally, a total of 25,274 genes were identified from the salt-treated and untreated tomato roots using FPKM > 1 as the standard for gene expression (Figure 7A). The RNA-seq data of two biological replicates exhibited good correlations and can be used for further investigation (Figure S4A). A summary of RNA-seq, assembly, annotation, and mapping is provided in Table S6-1. Finally, a total of 5548 genes with 2552 genes downregulated and 2996 genes upregulated were identified as the differentially expressed genes (DEGs) between salt-treated and untreated tomato roots using the |log2 (fold change)| > 1 and padj < 0.05 (a adjusted *p*-value) as the significance cut-off in DESeq2 (Figure 7A–C, Table S6-2). Using the H-cluster method, these 5548 DEGs were also divided into four clusters (sub-clusters 1–4, Figure S4B) with the similar expression trends of these DEGs of each cluster, which was consistent with the hierarchical clustering analysis. Among these DEGs, 2089 DEGs were identified as the members of different transcription factors families, such as the AP2/ERF, B3, bZIP, DEAD, GRAS, bHLH, Homeobox, MYB, NAC, WRKY, MADS-box, Dof family, and so on (Table S6-3).

The GO (gene ontology) functional enrichment analysis displayed that these DEGs were enriched in 888 GO terms (114 CC, 459 BP, 315 MF; padj < 0.05). For MF (molecular function), the DEGs were principally enriched in enzyme activity, transcription factor activity, and DNA binding. For CC (cellular component), the majority of the DEGs were associated with cell wall components, protein complexes, chromosomes, cytoplasmic parts, extracellular components, and the cell nucleus. For BP (biological process), most of the DEGs were connected to cell wall modification and metabolic processes, response to stress, transmembrane transport, and the carbohydrate metabolic process (Figure 7D, Table S6-4). KEGG analysis exhibited that 106 pathways were screened and most of the DEGs were mainly enriched in multiple pathways associated with the biosynthesis, metabolism, and plant hormone signal transduction of numerous amino acids (Figure 7E, Table S6-5). These data showed that these DEGs participated in response to salt stress perhaps through regulating the expression of genes related to cell component modification, amino acid metabolism, enzyme activity, transcription, and transmembrane transport.

### 2.11. Transcriptome Screening of Salt-Responsive SlbHLHs and Their Expression Patterns Under Abiotic Stresses

**Figure 8:**
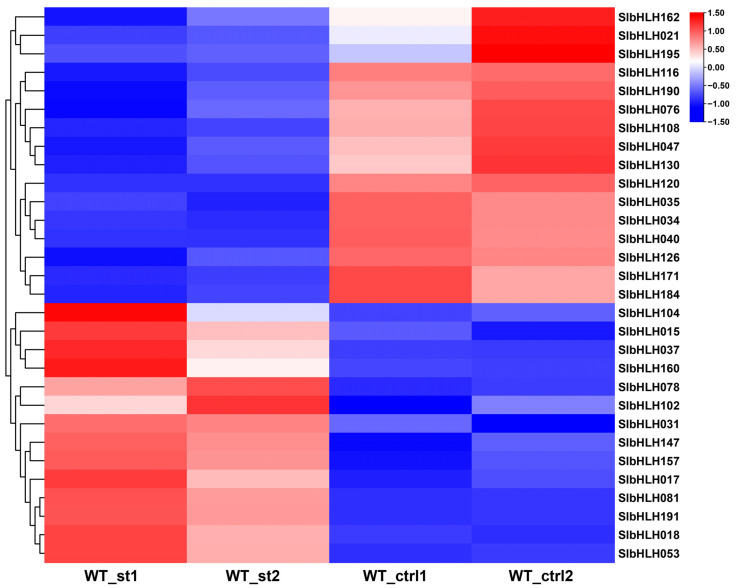
Heatmap of differentially expressed tomato bHLH genes in response to salt stress by RNA-seq. WT_st1, salt-treated tomato root sample 1; WT_st2, salt-treated tomato root sample 2; WT_ctrl1, control tomato root sample 1; WT_ctrl2, control tomato root sample 2.

**Figure 9:**
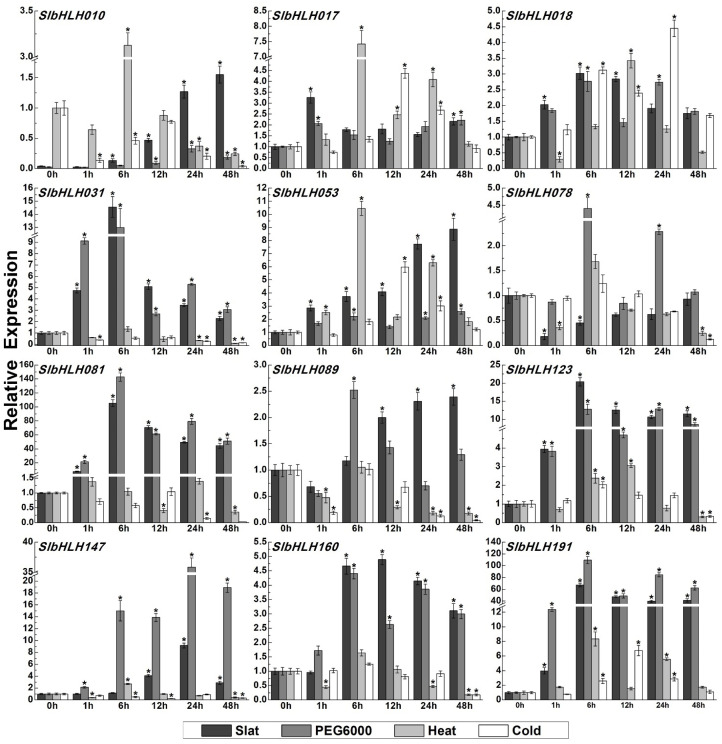
Expression profile analysis of 12 *SlbHLHs* that respond to multiple abiotic stresses using qRT-PCR. The abiotic stress treatments include salt (NaCl, 300 mM), drought (PEG6000, 20%, *m*/*v*), heat (42 °C), and cold (4 °C); 0h represents the WT seedlings that were not treated with every treatment. Bars indicate the mean of three biological replicates ± SE. The two-fold expression changes in SlbHLH genes in each post-treated sample compared to the 0h sample are considered as the significant expression changes. The significant differences were marked with the asterisks between the 0h sample and each post-treated sample using the Student’s *t*-test: * *p* < 0.05.

The expression level changes in *SlbHLHs* in WT and salt-treated tomato seedlings were analyzed based on our transcriptome data in this study. The results showed that almost one-sixth of the *SlbHLHs* (30 *SlbHLHs*) were identified as the DEGs in response to salt stress (Figure 8, Table S6-6). To verify the RNA-seq results, the expression of 12 *SlbHLHs* that displayed obviously different expression change in RNA-seq data were further detected under four kinds of abiotic stresses: drought, salt, cold, and heat by qRT-PCR (Figure 9). These results displayed that 12 *SlbHLHs* exhibited varied and remarkable expression levels under four abiotic stresses, particularly in response to NaCl and PEG6000 stress (Figure 9). Notably, *SlbHLH081* and *SlbHLH191* showed the highest induced expression levels under NaCl stress with about 67.4–105.3-fold changes. Higher and similar expression fold changes (8.8–20.3) were examined in the transcript levels of *SlbHLH031*/*053*/*123*/*147*, while low induction levels (2.3–4.8-fold) were detected in *SlbHLH017*/*018*/*089*/*160* expression under salt stress. The qRT-PCR results displayed good agreement with our RNA-seq data, while no remarkable induced expression changes in *SlbHLH078* were observed compared with the 0 h data (Figure 9).

For drought (PEG6000) stress, the expression levels of *SlbHLH081*/*191* were significantly upregulated with about 109.1–142.8-fold changes, following by the increased transcripts of *SlbHLH031*/*123*/*147* with 12.8–35.8-fold changes. The expression levels of *SlbHLH017*/*018*/*053*/*078*/*089*/*160* were increased by about 2.2–4.4-fold, while the transcripts of *SlbHLH010* showed no obvious expression changes. For heat stress, the expression levels of *SlbHLH017*/*053*/*191* were dramatically upregulated with about 7.4–10.4-fold changes, followed by the increased transcripts of *SlbHLH010*/*018*/*123*/*147* with 2.6–3.4-fold changes, while the transcripts of *SlbHLH031*/*078*/*083*/*089*/*160* showed no obvious changes (Figure 9). For cold stress, the transcripts of *SlbHLH017*/*018*/*053*/*191* were markedly increased with about 4.3–6.7-fold changes, followed by a small increased expression of *SlbHLH078*/*123*/*160* with 1.2–2.0-fold changes. Two bHLH genes (*SlbHLH081*/*134*) showed no obvious expression changes, while the expression levels of *SlbHLH010*/*031/089*/*147* were downregulated by cold stress (Figure 9). In conclusion, the diverse and significant transcript levels of *SlbHLHs* may function as differential and important regulatory genes in response to multiple abiotic stresses.

### 2.12. Expression Profiles of SlbHLHs Under Different Hormone Treatments

**Figure 10:**
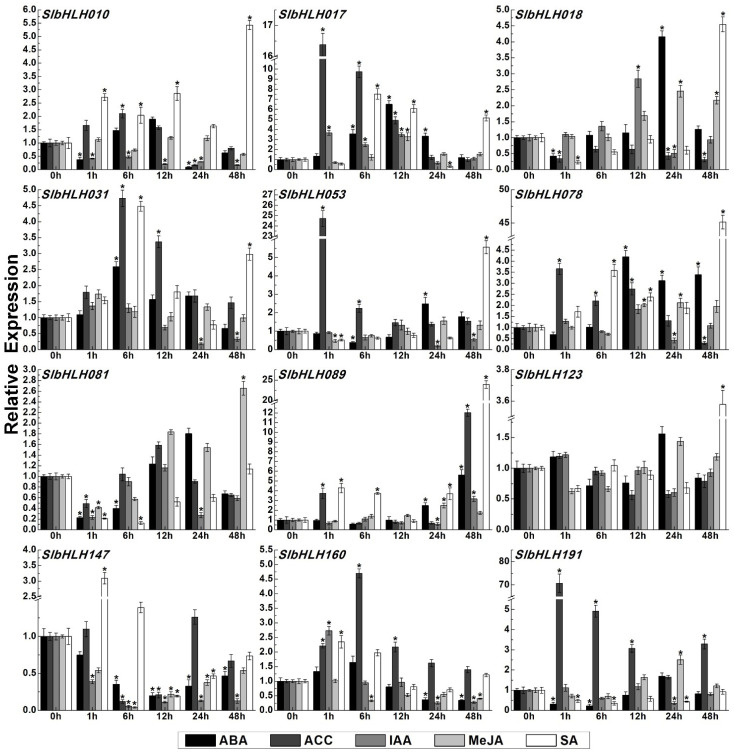
Relative expression levels of 12 *SlbHLHs* examined using qRT-PCR under diverse hormone treatments. The hormone treatments include SA (salicylic acid, 2 mM), ACC (1-Aminocyclopropane-1-carboxylic acid, 0.1 mM), ABA (abscisic acid, 0.1 mM), IAA (0.1 mM), and MeJA (methyl jasmonate, 0.1 mM); 0h represents the WT seedlings that were not treated with every treatment. Bars indicate the mean of three biological replicates ± SE. The two-fold expression changes in SlbHLH genes in each post-treated sample compared to the 0h sample are considered as the significant expression changes. The significant differences were marked with the asterisks between the 0h sample and each post-treated sample using the Student’s *t*-test: * *p* < 0.05.

The expression pattern detections of 12 *SlbHLHs* under various hormone treatments including ABA (abscisic acid), IAA (Indole-3-acetic acid), ACC (1-Aminocyclopropane-1-carboxylic acid), MeJA (methyl jasmonate), and SA (salicylic acid) were performed by qRT-PCR. These hormones were shown to play critical roles in response to various environmental conditions during plant development [49,50]. These results displayed that the transcriptional accumulations of these *SlbHLHs* were increased or reduced to varying degrees after hormone treatments (Figure 10), wherein the expression levels of *SlbHLH017*/*031*/*053*/*078*/*089* could be induced to various degrees by all the hormone treatments (ABA, ACC, IAA, MeJA, and SA). Contrarily, the transcripts of *SlbHLH010*/*018*/*123*/*147*/*160*/*191* were decreased under several hormone treatments. As a kind of stress hormone, ABA can induce the transcripts of almost all detected *SlbHLHs* to varying degrees, except *SlbHLH147*. The transcripts of *SlbHLH017*/*018*/*078*/*089* exhibited higher fold changes of 4.1–6.5-fold compared with the 0h data under ABA treatment. Notably, the transcripts of *SlbHLH017*/*053*/*089*/*191* were significantly upregulated with about 12.01–70.7-fold changes, following by the induced expression of *SlbHLH010*/*031*/*078*/*160* with 2.1–4.7-fold changes under ACC treatment. The transcripts of *SlbHLH017*/*089* were slightly induced by IAA (3.1–3.6-fold) and the accumulations of *SlbHLH017*/*018*/*078*/*083*/*089*/*191* were also increased to a similar degree (2.1–3.2-fold) after being treated with MeJA. Furthermore, SA treatment could markedly induce the transcripts of *SlbHLH078*/*089*/*123* to 12.8–45.1-fold, followed by the upregulated expression of *SlbHLH010*/*017*/*018/031/147/160* with 2.35–7.5-fold changes. In addition, the transcript accumulations of multiple *SlbHLHs* were decreased after being treated with different hormones at all or some time points (Figure 10). These results indicated that many SlbHLH genes may play crucial functions in response to multiple hormone (especially ACC and SA) and/or signal transduction.

## 3. Discussion

Nowadays, tomato is used as an excellent model plant for gene function study during plant growth and development. The genome-wide systematic characterization of gene families in tomato will provide insights for understanding the regulatory mechanism of crucial regulators during plant development and adaptation to adverse environmental conditions. The bHLH TFs are one of the largest transcription factor families that play crucial roles in controlling multifarious biological processes during plant development and in regulating tolerance against various adverse environmental conditions [7]. Although tomato bHLH transcription factors have been identified in previous studies [42], some new tomato bHLH genes which have not been identified in previous studies have been reported to play significant roles in tomato development, such as *SlPRE2* (Solyc02g067380) [8,9,43], *SlPRE3* (Solyc06g070910) [44], and *SlPRE5* (Solyc04g005660) [45], and so on. The genome-wide identification and characterization of bHLH TFs in tomato is lagging. The further genome-wide identification of tomato bHLHs is required to identify more bHLH genes in tomato. In this study, the DEGs that respond to salt stress in the root were analyzed and detected in the RNA-seq. Then, the features of *SlbHLHs* in tomato were systematically and deeply characterized at the genome level, and the transcripts of *SlbHLHs* in response to various hormones and abiotic stresses were also analyzed.

### 3.1. Characterization of SlbHLHs in Tomato

In this study, a total of 195 *SlbHLHs* were identified from tomato genomes; the protein lengths of these tomato bHLH TFs vary from 86 to 930 with the theoretical pI ranges from 4.65 to 11.45. The important variabilities and differences indicate the high levels of complicacy, which may be related to genome sizes or gene duplication events. The number of 195 *SlbHLH* genes in tomato was more than that in Arabidopsis (162) [41], rice (167) [41], tobacco (100) [51], pepper (122) [52], and wheat (159) [53], but less than maize (208) [54], *Medicago sativa* (469) [55], and mango (212) [56]. These results indicated that the significant divergence of bHLH genes occurred among monocot and dicot plants. Notably, no obvious correlation between the bHLH gene number and the genome size was observed. For instance, 162 bHLH genes were identified from Arabidopsis, while rice (373 Mb), tomato (950 Mb), Chinese cabbage (278 Mb), and wheat (13.7 Gb) had a genome size nearly 3.2, 8.3, 2.4, and 112.3 times larger than that of Arabidopsis (115 Mb), respectively, harboring more or fewer bHLH genes than Arabidopsis. Moreover, 195 *SlbHLHs* were located on all the chromosomes, but the number of *SlbHLHs* did not correspond to the size of the tomato chromosome. Analogously, this disproportionate distribution was also observed in rice [41], pepper [52], potato [57], and maize [54]. So, *SlbHLHs* distributions that were relatively clustered on tomato chromosomes may be due to the uneven duplication events of chromosome fragments.

Gene structures, conserved domains, and motifs of bHLH TFs can provided vital clues for investigating their evolutionary relationships. Though the protein properties of SlbHLHs showed significant differences, the domains, sequence motifs, and gene structures of *SlbHLHs* in the same clade were relatively conserved, which can provide the basic reference for the functional relevance and phylogenetic analysis of tomato bHLH genes. Gene structure, domain, and motif analysis displayed that closely related tomato *SlbHLHs* tend to exhibit similar domain, exon/intron structure and motif compositions, as found in other plants such as rice and Arabidopsis [41]. Gene structure study displayed that 85% of *SlbHLH* genes harbored 1–10 exons in their coding region, which were similar to the bHLH genes in pepper [52], wheat [53], and maize [54]. Nonetheless, several *SlbHLHs* exhibited the obvious exceptions of exon number (more than 10 exons), indicating high divergences among the *SlbHLH* genes. These increases or decreases may be caused by chromosomal fusion and rearrangement, and might lead to functional diversifications of gene family members [58].

In all SlbHLH proteins, 20 different types of bHLH domains with high diversity were observed in tomato, and these gene members, which belong to the same subfamily, had similar types of bHLH domains with few exceptions. The motif 1 and motif 2, which served as the components of the conserved bHLH domain, were highly conserved within almost all tomato bHLH proteins and were crucial for the functional specificities of bHLH TFs [59]. Although most bHLH proteins had similar conserved domains and motifs, significant differences in molecular characteristics among bHLH proteins were observed, which may result from the differences in their non-conserved regions. The additional compositions and numbers of motifs vary distinctly among different SlbHLH subfamilies. A number of specific motifs were found only in certain subfamilies, indicating that they may have diverse functions during tomato growth and development because of the crucial roles of bHLH genes, which were widely reported in numerous biological processes [7,60].

### 3.2. Phylogenetic Relationship and Collinearity Analysis of SlbHLHs in Tomato

The phylogenetic analysis showed that the 195 tomato bHLH TFs were divided into 27 subfamilies on the basis of classifications and sequence homologies of Arabidopsis AtbHLH proteins [1,2,41]. At least one SlbHLH protein was observed in each subfamily of Arabidopsis [2,39], indicating that the divergences of bHLH TFs may be earlier than dicots and monocots, while some new members and subfamilies were generated as evolution proceeded. The classifications of SlbHLHs were supported by both their gene structures and conserved motifs. In addition, the results of subfamily classification in tomato exhibited differences and similarities with other plants, manifesting more diversities in the function and structure of bHLH proteins among different plants [61]. Previously, phylogenetic studies reported that the bHLHs proteins in rice [41], Arabidopsis [62], tobacco [51], barley [63], and mango [56] were divided into 22, 21, 15, 24, and 27 subfamilies, respectively. These results indicate that the diversities of bHLH proteins in different plants result in the differences and complexities of bHLH classifications. Moreover, the classifications were also corroborated by the conserved domain and motif detections; the close SlbHLHs from the same subgroups generally include common domain and motif compositions. The results were consistent with the bHLHs in other plants. The data also support the previous conclusion that genomic structures could provide insights into comprehending the emergences and evolutions of a given gene family.

Gene duplications are one of the important driving forces for the evolution and expansion of gene families [47]. In our study, collinear analysis showed that many SlbHLHs were identified as the segmental and tandem duplications, and the contributions of duplications to the increase in SlbHLH genes are similar. The data indicate that segmental and tandem duplications play dominant roles in the diversity of bHLH genes, and some SlbHLH genes may emerge via gene duplications in tomato, further explaining the mechanism of bHLH gene expansion. Consistent results were also found in the bHLHs of rice [1,41], tobacco [51], pepper [52], Chinese cabbage [64], and apple [65]. It is worth mentioning that several duplicated SlbHLHs are involved in the response to various abiotic stresses. Moreover, the SlbHLH genes displaying tandem and segmental duplications are members of the same subfamily; the expansions of SlbHLH proteins from the specific subfamily might be beneficial to the adaptability of tomato to environments during domestication. These results further support the common mechanisms that segmental duplications or genome duplications may have a contribution to the expansion of bHLH genes that is observed in many other plants, such as Chinese cabbage [64], cauliflower [66], and apple [65].

In addition, the synteny analysis showed that the largest numbers of orthologous genes were identified between tomato and potato, followed by cucumber, pepper, watermelon, and Arabidopsis. These specific genes might be derived from a common ancestor, and might have arisen before their divergence. Furthermore, a more complex relationship such as single-potato-to-multiple-tomato genes was found, suggesting that these homologous genes may play significant roles in the evolutions of tomato *SlbHLHs*. But, only one or two homologous genes were found between tomato and three gramineae plants (maize, wheat, and rice); this phenomenon is probably due to the fact that their genomes have undergone massive chromosomal rearrangements and fusions, and selective gene loss has severely obscured the identifications of synteny relationships [67]. Interestingly, we also found that some bHLH genes were retained or lost in specific plants, leading to uncertainty as to whether these bHLH genes share a common ancestor. However, one SlbHLHs was found to be collinear with at least one syntenic gene among all the detected species with orthologous genes, suggesting that duplications may predate the divergences of some plants and play a vital role in the expansion of the bHLH gene family.

### 3.3. Transcriptome Analysis of DEGs Responding to Salt Stress

In plants species, numerous members of transcription factor families, such as bHLH [68], NAC [69], GRAS [70], MADS [71], bZIP [72], MYB [73], WRKY [74], and Dof [75], etc., have been reported to take part in the response to multiple biotic and/or abiotic stresses. Previous studies reported that numerous members of different gene families were identified in response to abiotic stress via the RNA-seq [76]. Also, our RNA-seq data showed that 5548 DEGs, with 2996 DEGs of tomato upregulated and 2552 DEGs downregulated, were identified as the significance cut-off in DESeq2. Among these 5548 DEGs, 2089 DEGs belong to members of 398 transcription factor families, such as the AP2/ERF, B3, C3H, bZIP, DEAD, GRAS, bHLH, Homeobox, MYB, NAC, WRKY, MADS-box, and Dof family, and so on. And, another 3459 DEGs also belong to different gene families, such as cytochrome P450, E3 ubiquitin-protein ligase, zinc finger protein, pectinesterase (PE), polygalacturonase (PG), and expansion, etc. All these DEGs are related to enzyme activity, DNA binding, cell wall components, cell wall modification and metabolic processes, the signal transduction of plant hormones, stress response, transmembrane transport, and carbohydrate metabolic process, and so on. These data indicate that plenty of genes play significant roles in response to salt stress via regulating or impacting the transcripts of genes related to cell component modification, amino acid metabolism, enzyme activity, signal transduction, transcription, and transmembrane transport, etc. In brief, our data provide a research basis for screening available gene resources in tomato that respond strongly to salt stress.

### 3.4. Expression Profiling and Functional Prediction of SlbHLHs in Tomato

To date, bHLH transcription factors have received wide attention because of their extensive involvements in regulating diverse biological processes during plant development, especially in the regulation of the adaptation to numerous abiotic stresses, including resistance to salt, drought, low/high-temperature stresses, oxidative stress, iron deficiency, heavy metal stress, and osmotic stress [68]. In addition, they also participate in the crosstalk of different hormone signaling, such as brassinosteroid (BR), salicylic acid (SA), abscisic acid (ABA), ethylene (ET), and jasmonic acid (JA) [60], and they are crucial for the growth and survival of plant species in the environment. The increasing number of investigations related to the significant roles of bHLH proteins in regulating plant responses to diverse adverse environmental stimulus show that bHLHs are promising candidates for increasing the tolerance of crops to numerous environmental stresses through molecular breeding.

Drought, high salinity, and cold stress are the major environmental factors that can cause biochemical and physiological effects on plants. These effects are mainly demonstrated by reduced enzyme activities, disordered hormone metabolism, decreased photosynthesis efficiency, and plant vigor and germination rates, leading to irreversible damage to plant yield, crop quality, and growth [77]. Many studies have reported that numerous bHLH TFs play significant roles in regulating numerous abiotic stress tolerances in various plant species. For instance, AtbHLH068 plays a significant role in response to drought stress via the ABA-dependent pathway by regulating the components of ABA metabolism and/or signaling [33]. Arabidopsis AtbHLH112 functions as a positive regulator in response to NaCl, osmotic, and drought stress through regulating genes relating to abiotic stress tolerance (salt, drought, and ABA) by binding to their GCG-box and E-box motifs [78]. The overexpression the bHLH gene *AtICE1*(*AtbHLH116*) increased the cold tolerance through the ABA-independent pathway in transgenic Arabidopsis [79]. A bHLH transcription factor, SlbHLH96, promotes drought tolerance in tomato [35]. The overexpression of a brassinosteroid-regulated bHLH transcription factor, SlCESTA, promotes chilling tolerance and fruit growth in tomato [80]. The ectopic expression of tomato bHLH gene *SlICE1a* in tobacco results in the enhanced cold and salt stress tolerance of transgenic plants [81]. The overexpression of a bHLH gene *OsWIH2* in rice led to significantly higher tolerance to drought [82]. The overexpression of rice bHLH gene *OsbHLH38,* which participates in ABA-dependent salt tolerance, enhanced the tolerance to salt stress [83]. Silencing *CabHLH035* in pepper decreased the resistance of transgenic pepper to cold stress, while the overexpression of *CabHLH035* resulted in an increased resistance to cold stress in Arabidopsis [84]. The bHLH transcription factor ZmPTF1 regulates the tolerance to drought stress through promoting ABA biosynthesis and root development in maize [85].

In addition, bHLH transcription factors play significant roles in many other biological processes, including flowering, high-temperature-mediated adaptations of plant architecture, phytochrome B signaling, and anthocyanin accumulation. For instance, the bHLH transcription factor DcTT8 regulates the biosynthesis of anthocyanin in *Dendrobium candidum* [86]. Two bHLH transcription factors, bHLH34 and bHLH104, regulate iron homeostasis in Arabidopsis [87]. Three bHLH TFs, MYC2 (AtbHLH002), MYC3 (AtbHLH005), and MYC4 (AtbHLH004), are needed for the jasmonate-mediated inhibition of Arabidopsis flowering [88]. The phytochrome-interacting bHLH TF PIL5 (AtbHLH015) controls gibberellin responsiveness through the direct regulation of GAI and RGA expression in Arabidopsis seeds [89]. The bHLH transcription factor HFR1/AtbHLH026 is involved in phytochrome A and cryptochrome signaling [90]. Three bHLH TFs, PIF3 (AtbHLH008), PIF4 (AtbHLH009), and PIF5 (AtbHLH065), play significant roles in dark-induced and age-triggered leaf senescence [91].

In our study, the data of RNA-seq analyses and qRT-PCR showed that most of the examined *SlbHLHs* exhibited obvious different expression patterns under different kinds of abiotic stresses and hormone treatments, suggesting the diverse and crucial functions of tomato bHLH TFs in resistance to diversified adverse environmental conditions. For instance, the transcripts of multiple *SlbHLHs*, particularly *SlbHLH031*, *SlbHLH081*, *SlbHLH147*, and *SlbHLH191*, were dramatically induced by various abiotic stresses. The increased transcript accumulation of these bHLH genes may help plants to decrease the damage generated by adverse abiotic stresses. Moreover, the potential roles of *SlbHLHs* in stress resistance were further certified by cis-element and phylogenetic analysis. Recent investigations displayed that bHLH members from multiple subgroups, including AtbHLH-068/-148 of subgroup orphan, AtbHLH122 of subgroup IX, AtbHLH112 of subgroup X, AtbHLH116 of subgroup IIIB, and AtbHLH-009/-015/-026 of subgroup VII, were all in connection with resistance to a variety of abiotic stresses, as described above [60,68,92]. Therefore, the transcripts of *SlbHLH018* in subgroup orphan, *SlbHLH017* in subgroup Ia, *SlbHLH160* in subgroup IX, *SlbHLH053* in subgroup X, *SlbHLH-031/-081/-147/-191* in subgroup IIIB, and *SlbHLH123* in subgroup VII were significantly increased by various stresses, indicating that they may participate in stress response pathways.

Phytohormones, especially SA, JA, ACC, and ABA, play vital functions in assisting plant species to resist various adverse environmental stresses [49]. For instance, AtbHLH068 regulates the response to drought stress through an ABA-dependent pathway in Arabidopsis [33]. OsbHLH062 can endow plant species with tolerance to salt stress by modulating the JA signaling pathway [93]. Furthermore, the cis-elements contained in the promoter regions of functional genes play crucial roles in regulating gene transcription. In this study, our data showed that numerous cis-elements related to different stresses and hormones including the DRE, P-box, CGTCA-motif, TCA-element, ABRE, MBS, LTR, GARE-motif, and ERE elements were observed in most *SlbHLHs* promoters. These results were similar to the previous studies of pepper [52] and *Nicotiana tabacum* [51]. The transcripts of most detected *SlbHLHs* (*SlbHLH-010/-017/-018/-031/-053/-078/-089/-160/-191*) were dramatically induced or decreased by one or a few phytohormones, such as ABA, ACC, IAA, MeJA, and SA. However, the detailed biological functions of most tomato *SlbHLHs* remain to be defined. Therefore, investigating the participation of *SlbHLHs* in the regulation of environmental stresses could provide valuable insights to study their potential functions in stress tolerance.

## 4. Materials and Methods

### 4.1. Plant Material and Growth Conditions

In this study, the tomato AC^++^ (*Solanum lycopersicum* Mill. cv. Ailsa Craig) was employed as the wild type (WT). All the WT tomato seedlings were cultivated under the standard cultivation environment (16/8 h day/night cycle at 25/18 °C, 250 μmol m^–2^·s^–1^, 80% humidity) until they were used. Three independent biological replicates were used for each treatment. Untreated seedlings were used as controls, and leaves or roots were collected at indicated time points.

### 4.2. Identification of Tomato bHLH Genes

The GFF annotations and genome sequences of *Solanum lycopersicum* and other plant species were obtained from Ensembl Plants database (http://plants.ensembl.org/index.html, 20 June 2024). All the amino acid sequences of Arabidopsis AtbHLHs were obtained from TAIR database (https://www.arabidopsis.org/, 20 June 2024) based on previous studies [41,62]. After that, these Arabidopsis bHLH proteins were used as inquiries to blast the tomato protein sequence database using the BLASTp program with E-value (1 × 10^−5^) to obtain the candidate bHLH protein sequences using the TBtools software (Guangzhou, China, V2.120) [94]. Subsequently, the Batch CD-Search database (https://www.ncbi.nlm.nih.gov/Structure/bwrpsb/bwrpsb.cgi, 20 June 2024), PROSITE database (https://prosite.expasy.org/, 20 June 2024), and the Pfam database (http://pfam.xfam.org/, 20 June 2024) were employed to confirm whether these collected candidate bHLH members contained a bHLH domain. Ultimately, the screened non-redundant sequences, which were regarded as the putative bHLH proteins, were used for further study. The nucleotide and protein sequences of each SlbHLH are listed in Supplementary file S1.

### 4.3. Phylogenetic Relationships of bHLH Proteins in Tomato

To study the phylogenetic relationships of tomato SlbHLHs, the complete protein sequences of 169 Arabidopsis AtbHLHs were tested together with identified 195 tomato SlbHLHs in this investigation (Supplementary Files S1 and S2, Table S2). A total of 169 Arabidopsis AtbHLHs, which were identified earlier [1,62,95], were used in this study. But, in Bailey’s and Toledo-Ortiz’s reports [62,95], we found that AtbHLH133 (At2g20095) and AtbHLH152 (At1g22380) were not bHLH proteins, so these two proteins were not used in this study. Then, a new bHLH protein (AT2G20100.3) was renamed AtbHLH133, but the serial number AtbHLH152 was not used in this study. In addition, another 8 AtbHLHs were named AtbHLH163–170 following Carretero-Paulet’s study [1]. After that, these 169 AtbHLH protein sequences from Arabidopsis, together with tomato SlbHLH sequences, were used to construct the unrooted evolutionary tree by MEGA-X software version 10.2. Then, the MEGA-X software [96], using the Maximum Likelihood method, was used to construct the unrooted phylogenetic tree. First, a total of 365 bHLH protein sequences were used for the sequence alignment employing the ClustalW program [97] of MEGA-X with default parameters [96]. Then, the MEGA-X software was employed to perform the construction of the phylogenetic tree using the obtained data via the Maximum Likelihood method [96]. The following parameters were employed: the best WGA + G + F evolutionary model calculated via MEGA-X with 1000 bootstrap replicas and partial deletion option. The construction of a phylogenetic tree of 195 SlbHLH proteins was also performed using the same method.

### 4.4. Protein Property, Conserved Domain, Motif, and Protein Interaction Analysis of SlbHLHs

Protein property, conserved domain, motif, and protein interaction analysis of SlbHLHs were performed using our previous methods [97]; each method is described as follows.

The ProtParam (https://web.expasy.org/protparam/, 21 June 2024) of the online ExPASy database (http://expasy.org/, Swiss Institute of Bioinformatics) was employed for the evaluation of the Mw (molecular weight) and the theoretical pI (isoelectric point) of 195 SlbHLH proteins. The online NetPhos 3.1 database (https://services.healthtech.dtu.dk/service.php?NetPhos-3.1, 21 June 2024) and the Plant-mPLoc database (http://www.csbio.sjtu.edu.cn/bioinf/plant-multi/, 21 June 2024) was employed to predict the phosphorylation sites and the subcellular locations of each SlbHLH protein, respectively.

To analyze the sequence diversity of tomato *SlbHLHs*, the conserved domains and exon–intron structures of each *SlbHLH* were detected. The Batch CD-Search database (https://www.ncbi.nlm.nih.gov/Structure/bwrpsb/bwrpsb.cgi, 22 June 2024) was applied to investigate the conserved domains of each SlbHLH protein.

To further evaluate the sequence diversities of SlbHLHs, we investigated the motif compositions. The MEME 5.5.0 (https://meme-suite.org/meme/tools/meme, 22 June 2024) was applied to study the motif of each SlbHLH protein.

Subsequently, the STRING 12.0 website (https://cn.string-db.org/, 22 June 2024) was employed to construct the potential protein interacting network.

### 4.5. Gene Structure of SlbHLH Genes and Cis-Element Analyses of Their Promotors

The intron–exon structure of each SlbHLH gene was illustrated using the genomic sequence and the GFF annotation data of 195 *SlbHLHs*, and the TBtools software was used to present the results [94]. The 2.0 kb sequence of each SlbHLH gene promoter was extracted from tomato genome by Tbtools software. Then, the promoter sequence of each SlbHLH gene was used to investigate the potential cis-elements that were associated with hormones, growth, development, and stress in the promoter region of 195 SlbHLHs using the plantCARE database (http://bioinformatics.psb.ugent.be/webtools/plantcare/html/, 23 June 2024). Finally, statistical analysis was performed to investigate the cis-elements of each *SlbHLH* promoter. The TBtools software and NovoMagic tool (https://magic.novogene.com/customer/main#/homeNew, 23 June 2024) were used to visualize all obtained results.

### 4.6. Chromosomal Distribution and Collinearity Analysis of Tomato SlbHLHs

The chromosomal distribution of 195 SlbHLH genes was analyzed on account of the GFF annotation information of tomato genomes provided by Sol Genomics Network (https://solgenomics.net/). To investigate the potential gene duplications among the 195 *SlbHLHs* in tomato, a collinear analysis of *SlbHLHs* was carried out. To further deduce the evolutionary relationships and origin of tomato *SlbHLHs*, the collinearity relationships between *SlbHLHs* and the orthologous genes of 10 representative plants were investigated, including two model plants (*Oryza sativa* and *Arabidopsis thaliana*), two Solanaceae plants (*Capsicum annuum* and *Solanum tuberosum*), two Brassica plants (*Brassica oleracea* and *B. rapa*), two Cucurbitaceous plants (*Cucumis sativus* and *Citrullus lanatus*), and two cereal plants (*Zea mays* and *Triticum aestivum*). For the collinearity analysis between *SlbHLHs* and associated genes in these 10 plants, the genome and the GFF annotation data of tomato and these 10 representative plants were downloaded from TAIR (https://www.arabidopsis.org/, 17 February 2024), Sol Genomics Network (https://solgenomics.net/), phytozome (https://phytozome-next.jgi.doe.gov/, 17 February 2024), and EnsemblPlants (http://plants.ensembl.org/index.html, 17 February 2024). The Multiple Collinearity Scan toolkit (MCScanX) with the default parameter was used to evaluate the collinearity relationships and gene duplications [98]. Finally, the TBtools (V2.120) software and advanced circos were employed to finish the visualization of results; the value 30 was set to the minimum block size [94,99].

### 4.7. Transcriptome-Wide Analysis of Salt-Responsive SlbHLH Genes

To explore the members of different transcription factor families in response to salt stress, the 8-week-old uniform WT tomato seedlings were selected for the salt stress treatment using 300 mM NaCl for 24 h. The RNA-seq of salt-treated and untreated tomato roots was carried out. Salt stress was carried out by submerging the roots of WT tomato seedlings in 300 mM NaCl for 24 h, and the untreated WT tomato seedlings were used as the control. Then, the roots of untreated and salt-treated tomato plants were harvested, respectively, from more than six different seedlings. Two biological replicates of untreated and salt-treated samples were used to perform the RNA-seq analyses. Then, 3 μg total RNA from each sample was obtained and employed as the input material for the construction of sequencing libraries. These 4 samples were sent to the Novogene Bioinformatics Institute (Beijing, China) for transcriptome sequencing and assembly.

All samples were sequenced and transcriptome analysis was performed by the Illumina HiSeq 2500 platform (San Diego, CA, USA). Clean reads were filtered by removing reads including poly-N, adapters, low-quality reads, and duplication sequences from the raw data. The transcriptome assembly was carried out by StringTie (v. 1.3.3b) [100]. The tomato reference genome database (https://solgenomics.net/organism/Solanum_lycopersicum/genome, 18 January 2024) was employed as the annotation of gene function. Read counts were used to estimate gene expression, and genes with |log2 (fold change)| > 1 and padj/*q*-value < 0.05 (adjusted *p*-value) determined by DESeq2 were considered as differentially expressed genes (DEGs) [101]. And, clustering analysis was performed using the hierarchical clustering analysis and the H-cluster method to analyze the identified DEGs. For the hierarchical clustering analysis, the log10 (FPKM + 1) value was normalized (scale number) and clustered. For the H-cluster method, after taking the value of log2 (FPKM + 1) for the expression of differentially expressed genes, the centralization correction was performed and then the clustering was performed.

To detect the potential functions of these DEGs in response to salt stress, GO (gene ontology) functional enrichment analysis was carried out to categorize them on the strength of their functions in cellular component (CC), biological process (BP), and molecular function (MF). The clusterProfiler R package [102] was used to implement the GO enrichment analysis of DEGs and to detect the statistical enrichment of DEGs by KEGG pathway enrichment analysis (https://www.genome.jp/kegg, 26 March 2024) [103,104,105]. The pathway of DEGs with padj (adjust *p*-value) < 0.05 was identified as the dramatically enriched GO terms and KEGG pathway enrichment. Ultimately, the SlbHLH genes’ and other gene families’ response to salt stress was screened from the obtained RNA-seq data. Read counts were used to detect the gene expression and DEGs were screened by Log2 (fold change) and false discovery rate (FDR), as previously described [106]. The RNA-seq data are available in the National Center for Biotechnology Information (NCBI) database under the accession numbers (SRR8184599; SRR8184598) of our previous study [106] and the accession numbers (SAMN38113668, SAMN38113669) of submission.

### 4.8. Abiotic Stress and Hormone Treatments

Tomato seedlings of AC^++^ were planted in a greenhouse; then, potted 8-week-old uniform WT tomato seedlings were selected for the performance of all treatments. The drought and salt stresses were performed, respectively, by submerging tomato roots in 20% (m/v) PEG6000 (Sinopharm Chemical Reagent Co., Ltd., Shanghai, China) and 300 mM NaCl (Sinopharm Chemical Reagent Co., Ltd., Shanghai, China) solutions, and then roots and leaves at the same position were harvested. Heat and cold stresses were performed, respectively, by culturing WT seedlings at 4 °C and 42 °C, and then leaves located at the same position were harvested. For the hormone treatments, all needed WT seedlings were sprayed, respectively, with SA (2 mM), ACC (0.1 mM), ABA (0.1 mM), IAA (0.1 mM), and MeJA (0.1 mM) solutions (Geneview, Beijing, China), and leaves located at the same position were harvested. After that, all samples were harvested at 0, 1, 6, 12, 24, and 48 h after different treatments. Three independent biological replicates were used for each treatment. All samples were harvested and liquid nitrogen was used to immediately freeze all samples. Finally, all harvested samples were stored at −80 °C until they were used to perform total RNA extraction.

### 4.9. RNA Extraction and Quantitative Real-Time PCR (qRT-PCR) Analysis of Salt-Responsive SlbHLH Genes

Total RNA was extracted from each sample using the plant RNA Extraction Kits (omega, Norcross, GA, USA, www.omegabiotek.com) with gDNA Eraser following the manufacturers’ instruction. Reverse transcription and the first-strand cDNA synthesis were performed using the same methods described before [107]. Then, the synthesized cDNAs were diluted to 15 ng/μL using RNase/DNase-free water. Then, the CFX96™ Real-Time System (Bio-Rad, Hercules, CA, USA) was used to perform the qRT-PCR following the same method as described before [107]. The tomato *EF1α* gene was used as the internal standard [108]. All required primers are listed in Table S7. The expression levels of the part of salt-responsive SlbHLH genes that were identified as the differentially expressed genes (DEGs) in our RNA-seq were tested using three independent biological replicates. Meanwhile, three technical replicates were also needed in each biological replicate.

### 4.10. Statistical Analyses

The experimental data were presented as the mean ± standard error from at least three independent biological replicates. It was adopted that a cut-off value of two-fold for the expression of each differential gene was considered as the biological significance [109]. The graphs were generated using the origin Pro software (OriginLab Corporation, Northampton, MA, USA, v8.0, SAS Institute).

## 5. Conclusions

In this study, numerous genes and transcription factors from different families were identified to be the potential and significant candidates that responded to salt stress through RNA-seq analysis. A total of 195 bHLH TFs were identified from tomato genome, and the relationships of *SlbHLHs* to abiotic stress were discussed in this study. The phylogenetic relationships, syntenic analysis, molecular characterization, conserved domains and motifs, chromosome localization, and gene structure of these 195 bHLH TFs were investigated systematically in this study. Both segmental and tandem duplications contributed to the expansions of *SlbHLHs* in tomato, and the collinearity analysis of orthologous genes in 10 representative plant species further provided significant clues to the evolutionary relationships of *SlbHLHs*. Expression and bioinformatics data supported the prospect that multiple *SlbHLHs* such as *SlbHLH031*, *SlbHLH081*, *SlbHLH123*, *SlbHLH147,* and *SlbHLH191* possess promising candidate regulators for the genetic engineering of tomato with enhanced stress tolerance. But, the regulatory mechanisms and specific roles of the excellent candidate *SlbHLHs* in response to stress tolerance at molecular and physiological levels need to be further investigated. In summary, all results obtained in this study will provide more valuable information to further understand the significance and intricacy of the bHLH transcription factor family, and promising prospects of multiple identified SlbHLH members in regulating tomato tolerance to abiotic stresses are expected.

## Figures and Tables

**Table 1:** Gene pairs that occurred in tandem or segmental duplication events in tomato.

Duplication Events	Gene Pairs that Occurred in Duplication Events
Tandem duplication	*SlbHLH004*-*SlbHLH005*, *SlbHLH011*-*SlbHLH012*, *SlbHLH024*-*SlbHLH025*, *SlbHLH025*-*SlbHLH026*, *SlbHLH046*-*SlbHLH047*, *SlbHLH047*-*SlbHLH048*, *SlbHLH059*-*SlbHLH060*, *SlbHLH060*-*SlbHLH061*, *SlbHLH106*-*SlbHLH107*, *SlbHLH163*-*SlbHLH164*, *SlbHLH164*-*SlbHLH165*, *SlbHLH165*-*SlbHLH166*, *SlbHLH176*-*SlbHLH177*
Segmental duplication	*SlbHLH016*-*SlbHLH168*, *SlbHLH030*-*SlbHLH054*, *SlbHLH032*-*SlbHLH042*, *SlbHLH040*-*SlbHLH087*, *SlbHLH045*-*SlbHLH081*, *SlbHLH049*-*SlbHLH085*, *SlbHLH057*-*SlbHLH114*, *SlbHLH062*-*SlbHLH111*, *SlbHLH063*-*SlbHLH112*, *SlbHLH065*-*SlbHLH110*, *SlbHLH068*-*SlbHLH105*, *SlbHLH078*-*SlbHLH189*, *SlbHLH079*-*SlbHLH190*, *SlbHLH081*-*SlbHLH191*, *SlbHLH083*-*SlbHLH192*, *SlbHLH086*-*SlbHLH193*, *SlbHLH115*-*SlbHLH182*, *SlbHLH130*-*SlbHLH162*, *SlbHLH131*-*SlbHLH161*, *SlbHLH133*-*SlbHLH142*, *SlbHLH132*-*SlbHLH139*

**Table 2:** Members of bHLH proteins and their roles in Arabidopsis and their homologous proteins in tomato.

bHLH Proteins in Arabidopsis	Homologous bHLH Proteins in Tomato	Roles of bHLH Genes in Arabidopsis
GL3	SlbHLH140	Participate in the regulation of root development via the pathway of brassinolide or jasmonic acid or auxin.
RHD6	SlbHLH044	
AT1G61660/AtbHLH112	SlbHLH037, SlbHLH053	
RSL2	SlbHLH083, SlbHLH192	
LHW	SlbHLH115, SlbHLH141, SlbHLH182	
LRL1	SlbHLH118, SlbHLH184	
UPB1	SlbHLH009, SlbHLH100	
BIM1	SlbHLH062, SlbHLH111	Play significant roles in controlling embryonic development and/or photomorphogenesis.
BIM2	SlbHLH071, SlbHLH095	
PAR1	SlbHLH127	
MYC2	SlbHLH132, SlbHLH139	
RGE1	SlbHLH002, SlbHLH003, SlbHLH006, SlbHLH024, SlbHLH146	
AT2G41130/AtbHLH106	SlbHLH080, SlbHLH173	Participate in controlling low-temperature and/or salt stress responses.
AT3G20640/AtbHLH123	SlbHLH130, SlbHLH162	
ICE1	SlbHLH065, SlbHLH110	
PIF4	SlbHLH123	
PIF7	SlbHLH063	
TT8	SlbHLH151	
AT4G29100/AtbHLH68	SlbHLH067, SlbHLH109, SlbHLH121	Enhance the drought stress tolerance through regulating ABA-induced genes under drought stress.
MYC2	SlbHLH132, SlbHLH139	

## Data Availability

Most data generated or analyzed during this study are included in this published article and its Supplementary Information Files. Sequence data that support the findings of this study have been deposited in the NCBI database (https://www.ncbi.nlm.nih.gov/sra, 9 November 2023) with the accession numbers (SRR26696993, SRR26696994, SRR26696995, SRR26696996).

## Supplementary Materials

The following supporting information can be downloaded at: https://www.mdpi.com/article/10.3390/plants14020200/s1, Figure S1: Distributions of 196 SlbHLH protein motifs identified by MEME data; Figure S2: Predicted cis-elements in the promoters of 195 tomato *SlbHLHs*; Figure S3: Interaction networks of SlbHLHs in tomato according to the orthologues in *Arabidopsis thaliana*; Figure S4: Correlation analysis and cluster analysis of DEGs; Supplementary file S1: The nucleotide sequences and amino acid sequences of 195 SlbHLHs identified in tomato genomes; Supplementary file S2: The nucleotide sequences and amino acid sequences of 169 AtbHLHs identified in the genomes of *Arabidopsis thaliana*; Table S1: Bioinformatics analysis of 195 SlbHLH proteins; Table S2: Accession numbers of bHLH genes in tomato and *Arabidopsis thaliana*; Table S3: Chromosomal locations and tandem and segmental duplication of tomato bHLH genes; Table S4: Synteny statistics of tomato bHLH genes with 10 other species; Table S5: Analysis of cis-elements in 195 bHLH gene promoter regions of tomato; Table S6: RNA-seq-related data; Table S7: Specific primer sequences used for qRT-PCR analysis.
