# Supplementary material for: Genome- and Transcriptome-Wide Characterization and Expression Analyses of bHLH Transcription Factor Family Reveal Their Relevance to Salt Stress Response in Tomato
Source: Plants (Basel). 2025 Jan 12;14(2):200. doi: 10.3390/plants14020200 (PMC11768425; doi:10.3390/plants14020200)
Supplement: Supplementary file 1 [file plants-14-00200-s001.zip › Supplementary figures.pdf]

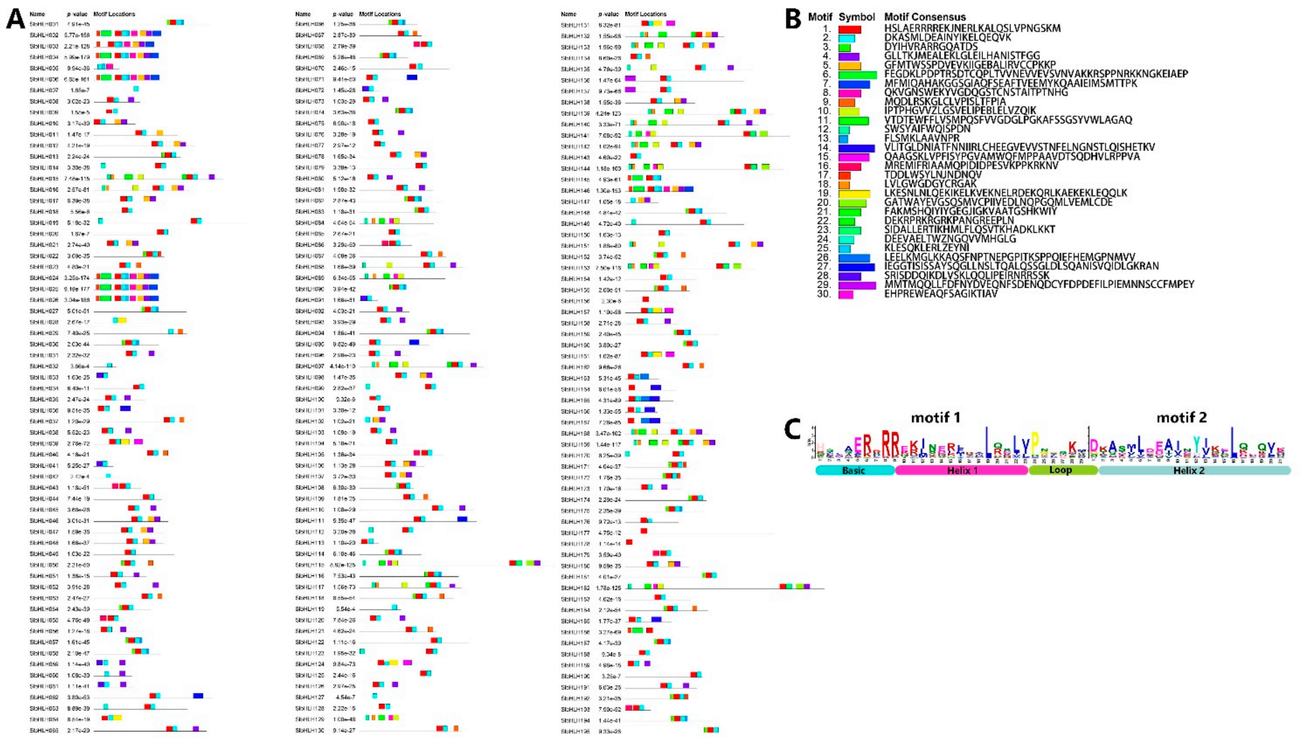

Figure S1 Distributions of 196 SlbHLH protein motifs identified by MEME data.

The number (30) and length (12-50) of conserved motifs are based on the settings in Arabidopsis. The distribution (A) and sequence consensus (B) of 30 most significantly conserved motifs in SlbHLHs. The colorful boxes denote different motifs. (C). Schematic representations of multiple sequence alignment of the helix–loop–helix (HLH) region and the basic region in SlbHLHs. The height of the letter at each position is positively correlated with the conservation of the corresponding amino acid residues at that position.

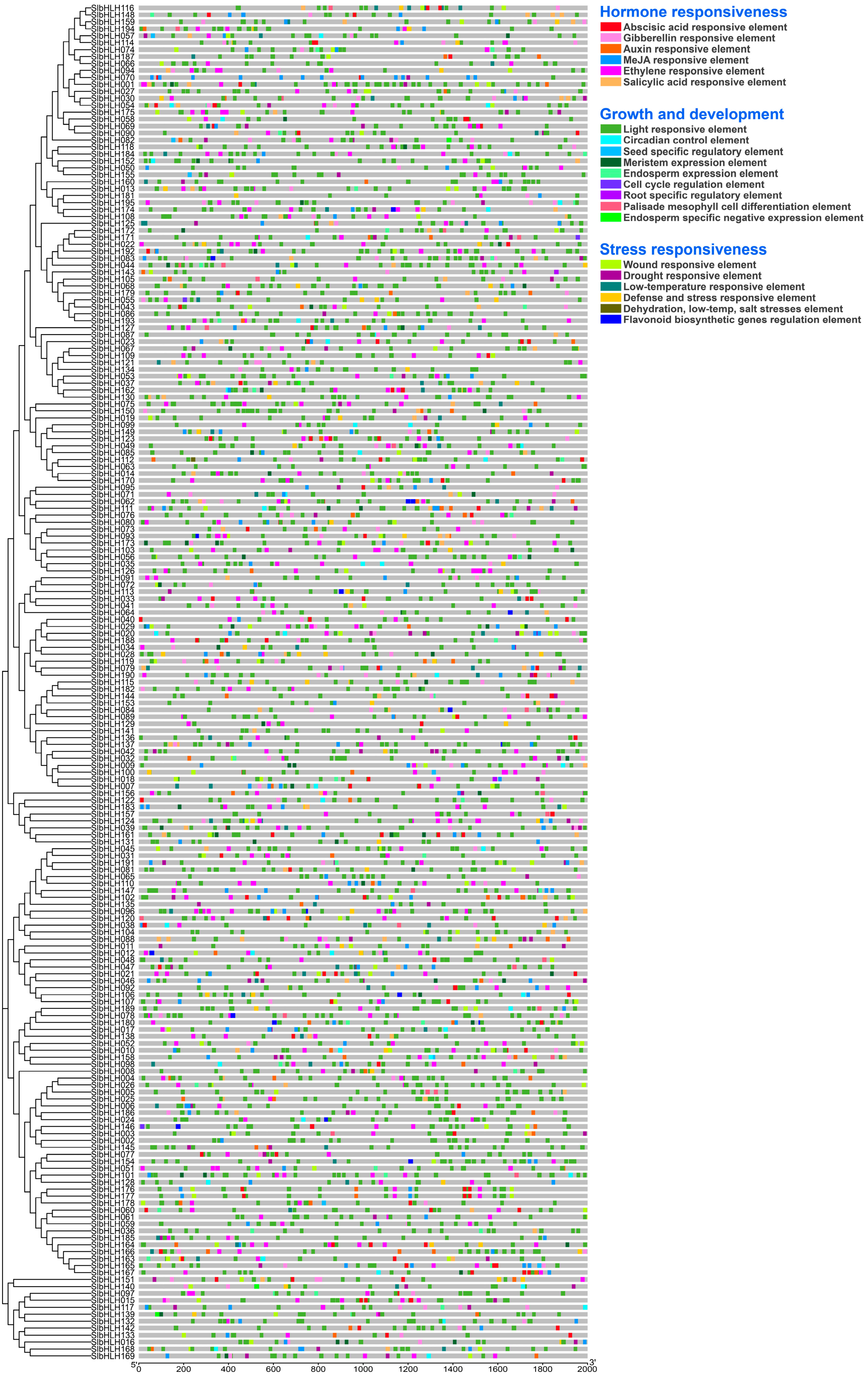
Hormone responsiveness
Abscisic acid responsive element
Gibberellin responsive element
Auxin responsive element
MeJA responsive element
Ethylene responsive element
Salicylic acid responsive element
Growth and development
Light responsive element
Circadian control element
Seed specific regulatory element
Meristem expression element
Endosperm expression element
Cell cycle regulation element
Root specific regulatory element
Palisade mesophyll cell differentiation element
Endosperm specific negative expression element
Stress responsiveness
Wound responsive element
Drought responsive element
Low-temperature responsive element
Defense and stress responsive element
Dehydration, low-temp, salt stresses element
Flavonoid biosynthetic genes regulation element
5'
0
200
400
600
800
1000
1200
1400
1600
1800
2000
3'

Figure S2 Predicted cis-elements in the promoters of 195 tomato *SlbHLHs*.

The phylogenetic tree and predicted cis-elements detected from 2000 bp promoter regions of each *SlbHLH* gene by PlantCARE database. The same phylogenetic tree as figure 4 was used. All cis-elements are classified into three categories: hormone responsiveness, growth and development, and stress responsiveness.

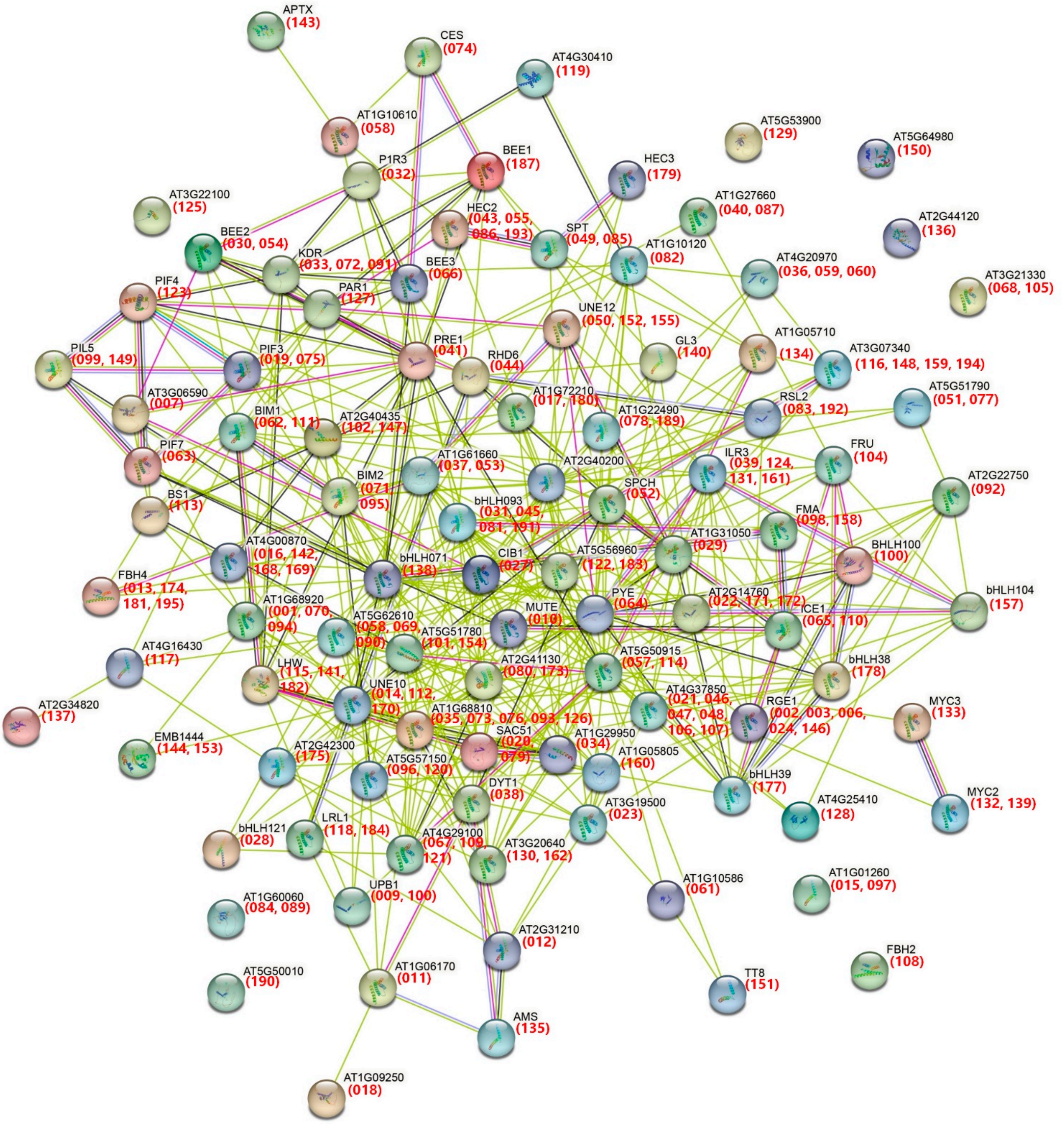

Figure S3 Interaction networks of SlbHLHs in tomato according to the orthologues in Arabidopsis.

The amino acid sequences of SlbHLHs in tomato were employed to search the STRING database. Network node represents proteins, and edge represents protein-protein associations. The different

colored lines between the nodes indicate the different kinds of interactions. The numbers (SlbHLH gene name) in brackets represent the corresponding orthologues in tomato. The filled and empty nodes delineate the proteins with known or predicted 3D structures and unknown 3D structures, respectively.

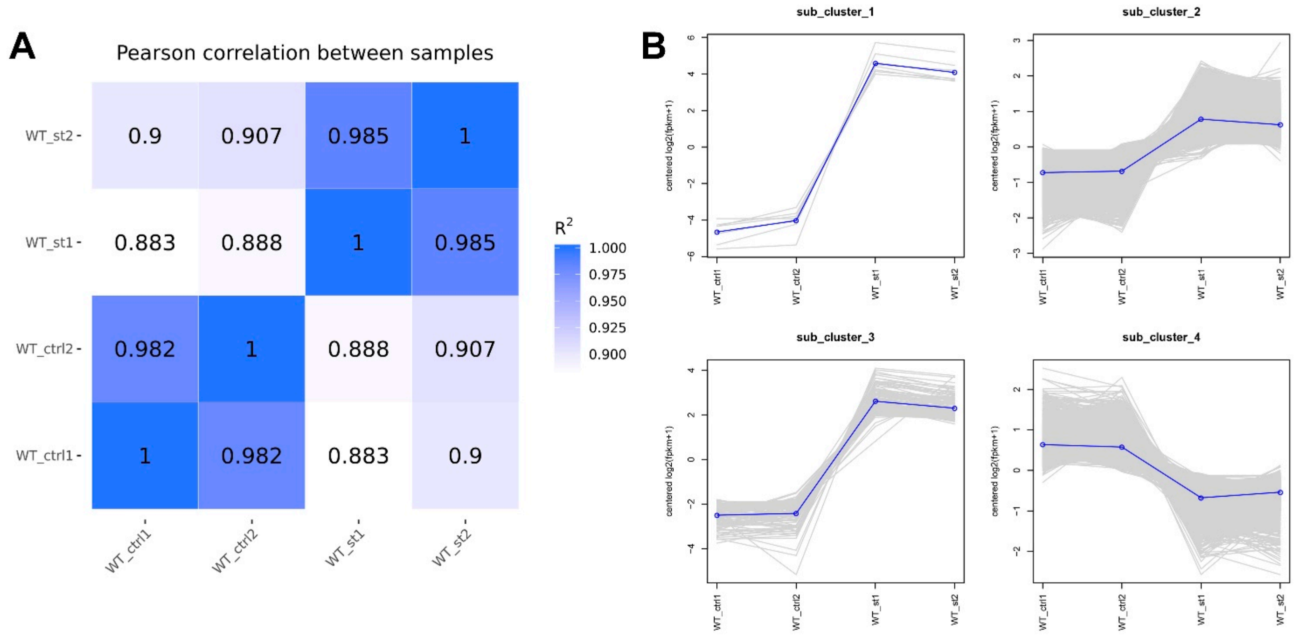

Figure S4 Correlation analysis and cluster analysis of DEGs.

(A). Correlation analysis (through Pearson's correlation coefficient) of RNA-seq data between two replicates. (B). Cluster analysis of DEGs using H-cluster method.
