# Supplementary material for: Genome- and Transcriptome-Wide Characterization and Expression Analyses of bHLH Transcription Factor Family Reveal Their Relevance to Salt Stress Response in Tomato
Source: Plants (Basel). 2025 Jan 12;14(2):200. doi: 10.3390/plants14020200 (PMC11768425; doi:10.3390/plants14020200)
Supplement: Supplementary file 1 [file plants-14-00200-s001.zip › Supplementary file S1 Gene and protein sequences of tomato 195 SlbHLHs.pdf]

Supplementary file S1. The nucleotide sequences and amino acid sequences of 195 SlbHLHs identified in tomato genomes.

195 nucleotide sequences

>SlbHLH001
ATGGATATGGGTAGCAAGAATGATACTCAAGGACAAAAGAGGAACAAGGATGCTACTAATT
TTCAGTCACCAAACATATCGTTGGACTGGCAATTAAGTGGAAGCAACTTGACAAATGCATC
AATGGGGATGATCCCTAATAGCAATCCTTTAGTAGATTCAGTTTTTCCCACTATTTGGGATCG
GCCTCCCAATTCGTCACACTTAGGTTTCTATGGTAATAATAATGCTAATGCTCAAATCAGTCC
TTGCATTATGAATCAGCACGAGACTGCAGCAATTGGCTCGGTCCCTACGAGAGGTAGTATGA
GTTGGAATCCACTGAATTCGATGCTGAAAGGGGCTATGTTTGTACCACCTATTCCTGGAATG
ATTCCTCAAAGCTTAGCTCAGCTTCCAGCTGATTTGGGTTTCATTGAGAGAGCTGCAAGGTT
TTCGTGCTTCAGTGGAGGAAACTTCAATGATATGATGAACCGGCCTTTAAGCGTCCCTGAGT
CTACTAAGCCTTGTTATAGGGGACCGGCACCAACGTGGAGAACCGAAGAGGTTCTTGCAAG
CAGCGGGTTAAACTCACCCTCTGCTGTGGACCCTTGGAAGCAGAACATACGAAGTGGTGTT
GATGGTTCCAAAGATGTTTCTTTACCTCATGAGAACAAAACTCATGAGCAAAGCCCTCTCA
AGATTGAGAAGAAGAATGAAATATTTGCGAGGTCTCGGGATGAAGGAAAAGAATCAGTTG
GATTGTCTGGAAATGAGTCTGATGAAGCTGAATGTAGCGGCCGTCAAGAGGAAATGGGAA
GTGCCGGACTGGAATCTTCCCCGAAGAGCCTTGGCTCAAGGAAAAGGAAAAAATATAGTC
AGGGTACCGAGCATGATCGAATGAAGCGAGTACAACAGCTGCCAGCTGAACCTGATAAAG
AACTAATTGAAACTCAGAAAGGAGATGGACGCTTACATTCACCTTCTAGCAAGCATGGCGG
AAAAAACAGTAAACAGAGGTCTCAGTCTTCAGATCCACCTAAAGAAGATTACATACATGTT
CGAGCTCGAAGAGGCCAAGCGACGAACAGCCATAGTCTTGCAGAAAGAGTAAGGCGAGA
GAAAATAAGTGAAAGGATGAAATTTCTGCAGGATCTTGTACCTGGTTGTGACAAGGTCACT
GGTAAAGCTGTAATGCTGGACGAAATCATTAATTATGTTCAATCTTTGCAAAGACAGGTTGA
GTTCCTCTCGATGAAGCTTTCAACAGTAAACCCAAGGCTAGATTTTAATCTTGATGGGGTCC
TTACAAAAGATTCACAAGCAGGTCCTTCGTCCGCACTTGCGTTTTCACCTGATAATATGACC
ATGACTTATGCTTCTTTGCATGGATGGCAATCTGGTCTGCTTCAATCAGGTCTTCCTGGTGAT
GGAAATTACATTGATGCGTTCCGTAGATCCAACACCCAACTCTCCTCTATGAGTGGTGGCTAT
AGAGATCCTTCATCTCAGGTACCTAGTGTATGGGATGATCAGCTGCATAATGTTGTGGATATG
GGATTCACTTCAACTGCACCCCTCGATTGCCAGGATTTAAGTTCTTTGCCACCCGACCAAAT
GAAAACAGAACCTTGA

>SlbHLH002
ATGAATGGAGGCGGCGAGAGTAATCATGTTTTTCCATGGGAAATAGATGATGTCTGGTCATA
CCTGAATATGAATGACAATCAAATTGGGAGTGGAGCGATGTTTGAGGGTGACATGATGCCA
GATCCACTTAGATTTGATACTTATCACCCGTTAACTGTTGTTAATGAGGTGGTTGGATCGAGT
ACTAATGTTGCTAAGAGGAGAAGTCCCCCCAACTCAAAAAAGAACGGTAAGAAAATTGTT
GAACCAAAATCCAGTGCTGATGGAGTCGGATATAGAGAAGGTTTAGAGCATGAGATACACA
TATGGACAGAGAGAGAAAGGACAAAGAAAATTGGAATCTTGTTCGAGACGCTCCGTGCTT

TGATTCCTAATATCTCTGCTAAGGCTGATGAGTGCACCATTATTGATGAAGCAGTGAACCACA
TCCTAAAGCTGAAGAATACTTTTGAGAAGCTTAAACAGAAAAAGCTAGATGATCTTCAAGA
ATATAACATAAGATTGATGAGTTCACAGAAATTTCCAGATGTTGGTAATAGTTGGGAGAATTA
TTTGGGTGATCAAGGAACGACGAGTAATTCATCTTTTATTAAACCAAAAATTCATGGTGCTA
CTCCCCTTATGGTGAATAATAATATTCCAACAAGTTTTATGACGTGGAGTTCACCAGATATGA
TACTAAACGTTTGTGGTAAAGATGCACATATTAGTGTGTGTTGTCCTAAGAAACTAGGGCTA
TTTACCTTCATTTGTTACGTTTTGGGGAAACACAATATTGAGATTGTGTCTGCTCAAGTTTCA
TCTGATCAATCTAGAACCATGTTCATGATCCAAGCTCATGCTAAATGTGGAAATGACATTGCT
CAATTCTCCGAAGCACCCACAGTGGAAGAAATATACAAGCAAGTTGCAATTAGGATAATGT
CCTTTGAAACCCCTAAATGA

> SlbHLH003
ATGAATGGAGGCGGTGAGAATAATCATGTTTTTCCATGGGAAACTAATGATGGATGGCCGTA
CCAAAACTTGAATGGCAATCAAATTGGGAGTGGAGTGACATTTGAGGGTGACAAGTTACAA
GATCCAAATAGATTTGATACTTATGAACCATTGACAGCTGTTAATGAGGTGATTGAAGCAAG
TACTAATGCTGGTAAGTCTACAATTGTTGAGAAAGCAGTGAACCACATCCAAAAATTGCAG
AATACTTTTGAGAAGCTTGAACATGAAAAACTAAAAAGGCTTCAAGAGCATAATGTAAGGT
GTATGAGTTCACAAAAATTTACTAATGCTGGTAACAATTGGGAGAAATATCAGGGTGATCAA
GGATCAACACATAATTCTTCCTCTATTGCATCAACAACTCATGGTACCAATCCCCTTATGGTG
AATAATAACATTCCAACAGGTTTTGTGACATGGAGTTCACCAAACGTGATAGTTAATGTTTG
TGGTGAAAATGCACATATTAGTGTGTGTTGTCCAAAGAAGTCAGGGCTATTCACCTTCATTG
GTTATGTTTTGGGGAAACATCAGATTGAGATTCTGTCTGCTCAAGTTTCATGTGATCAATTCA
GAAGCATGTTCATGATCCAAGCTCATGCTACAGGTGGAACTGGCATAGCTCAAGTCTCTGA
AGCATCCAGAGTTGAAGAAATGTACAAGCAAGTTGCGATTGAGATAATGTCGTTTGCAACC
CCCAATTGA

>SlbHLH004
ATGTTGCCAAAAAGAGAAGTTCAGCCAACCGGAAAAAAAAATGGTAAGGAAATTGCTGAA
CCAAATTCTGGTGTTTATGGAGCCGAAGTTAGAAAAACATCAAAACATGAGGTACGCAAAT
GGACAGAGAGAGAAAGGAGAAAGAAGATGCGAACCCAGTTCGAGAATCTTCATGCTTTGG
TTCCCAACCTCCCTGCTAAGGCTGATATGTCTAAAATAGTTTATGAAGCTGTGAACCGTATAC
GAAAGCTGAAGAATACTTTTAAAAAACTTGAAAGTCAAAAGCTAAAAAGTCTAGAAGAATA
TAACATAAGGTTGACGGGTTCACAAAAAGTTGATAATAGCTGGGAGAAGTATGTGGGTGAT
CAAGGATCAACCTGCAATTCTATAGCTATTATACCGACTAATCATGGTGCTAGTCCCCTTATTC
CAACAAGTTTTATGACATGGAGTTCACCAAATGTGATACTAAATGTATCTGGTGAAGATGCA
CATATTAGTGTGTGTTGTCCTAAGAAGCCAAGGCTATTTACCACCATTTGTTATGTTTTGGAG
AAACATAAGATTGACATTGTGTCTTCTCAAATTTCATCTGATCAATTCAGAAGCATGTTCATG
ATACAAGCTCACGCTAAAGATGGAAGTGGGGTTGCTCAATTCTCTGAAGCATTCACCGTGG
AAGACATGTACAAGCAAGCTGCAAATGAGATAATGTTGATGACAACCCCCAAATGA

>SlbHLH005
ATGTGGATTCTATTCAAGACTCTTCATGCTTTGGTTCCCAACCTCCCTTCTAAGGCTGATAAG
TCTAAAATAGTTTATGGAACTGTGAACCGTATCCGAAAGCTGGAGAATACTTTCAAAAAACT
TGAAAGTCAAAAGCTAAAAAGGCTAGAAGAATATAACATAAGGTTGACGGGTTCACAAAA

AGTTGGTAATAGCTGGGAGAATTATGTGGGTGATCAAGGATCAACCTGCAATTCTACAGCTA
TTACACCGACTAATCATGGTGCCAGTCCCCTTATTCCAACTGGTTTTATGACATGGAGTTCAC
CAAATGTGATACTAAATGTATATGGTGAAGATGCACATATTAATGTGTTGTCCTAA

>SlbHLH006
ATGAAAAAGAAAAAAATGAATGAAGGTGGTGGGAATGACCATGATGCTGATGTCTGGTCAT
ACCTGGACTTGAATGACTATCAAGTTGGGAGTGGAGAGAAGTTTGAGGGTGACAAGGTTCT
AGATCTGACTAGGTCGGATGCTTGTCAGCCGTTAACAATAGTTAATGAGATGTTTGAAGTGA
ATATTTATGCTGCCAAAAATAGAAGTCCACCAAATCGAGAAAAAAATGGTAAGGGCATTGCT
GAACCAAAGTCTGGTGTTGATAGAGGCGGAGGTAAACGAGAATCTAAGCACAAGATACAC
AAATGGACGGAGAGAGAAAGGAGAAAGAAGATGCTAACCTTGTTTGACACTCTTCATGATT
TGGTTCCTAATCTCCCTACTAAGGCTGATCAGTCTACAATAGTTGGTGAAGCAGTGAACCAC
ATCCTTGAACTGCAGAATATTTTCACAAAACTTAAAAGTCAAAAGCTAGAAAGGCTAGAAG
AATATAACATAAGGTTGATGAGTTCACAAAAAGTTGATAATAGTTGGGAGAAATATGTGGGC
GATCAAGTATCAACCAACAATTCAACTGTTATTACACCAACAACTCATGGTCCTAGTCCTCTT
ATTCCAACAGGTTTTATGACATGGAGTTCACCAAATGTGATACTGAATGTTTGTGGTGAGGA
TGCACATATTAGTGTGTGTTTTCCTAAGAATCCAAAATTATTTGCCATTATTTGTTATGTTTTA
GAAAAACATAAAATTGACATTCTGTCTGCTCAAGTTTCATCTGATCAATTCAGAAGCATGTT
CATGATCCAAGCTCATGCAAAAGGTGGAAGTGAGTTAGCTCTATTCTCCGAAGTATTCACAG
TGGAAGACATGTTCAAGCAAGCTGCAATTGAGATAAGGGCATTGACAACCTCCAAATGA

>SlbHLH007
ATGGAACCAATGGTCGTAGAGATGTCTTCAACGGTGATTTCGAATCCTGTAACGTCTTCTGA
CAGAGTAATATCAAGGAGAAAAAAGAGTAAGAAAAGTTTGAGAAATCAAACTCAGAACAG
TAGTAACAATAACAATAACAATAGTGAAACTCCGACTAATACTACTGAATGGAAAACTCAAG
CTCAACAACAAGTTTACTCTTCAAAGCTACTCAAAGCACTCCGTGAAGTGCGGATCAGTTC
ACCAGCGGCGGCGGCGACGACGACGACTTCATCGGTGCCGGCGCCAAAAGGCGGTCGAGC
CGTACGTGAAGTTGCTGACAGAGTCCTTGCCGTTACTGCCAAAGGACGATCCAGATGGAGC
CGAGCTATACTTACGAATCGGCTAAAGCTCAAGTTCATGAAGAAACACGCCAAGCGGCAGA
AAATGGCGGTTTCGTCTACCAGCCGGTTACCCAGGAAGCCGAGATTGGGGATTTTGAAGCT
GAAAACAAAGAATTTGCCGGCTTTTCAGAAAAAGGCTAGAGTTTTAGGACGGCTTGTTCCT
GGTTGCCGGAAACAACCGCTGCCGGTGATTCTAGATGAAGCTACTGATTACATAGCAGCTCT
TGAGATGCAAATTCGAGCGATGAGTGCTCTAGCTGATCTCCTTTCTGGTGCTTCCTCAAGTA
CTACAGCTCCTCTTGATCAACTCAGCTCGAGTCGACCTCCTCCTATTTGA

>SlbHLH008
ATGTGGCAAGAGATGATGATGAAGGAAGAAGCAGAAGAGGAAGAGATTGAGAGATTATTTA
TGGGTGTTATGGGAGGATATAAAGATGCTTTCATGCCGCCATTGACGGATTTTTCCACCATTA
ATGAGATGGGTTCAAGCTCATCCTATGGGAATAAGAGGCACAAAAAAGAAGCTCCTCTTGC
TGATGATAATATGCATAAGGAACGTAACAGAAGAGGGAAAATGGCTGAGTTGTACTCTCTG
CTTCAATCCTTAGTCCCAACAATCTCCCACATCCATAAGGCGACGAGGGAGAAGATTGTGG
CGGAATCTATAGATTACATCAAACGTCTTGAGGAAGAAGTATTGAGGTTGAAGAATCTGAA
GAAGTCAGTAGTGGTGTATAAACCTGCTTTATCTCAATGGAGAAACAGGGTTTCTTCTGTTA
ATGCTACTGTATCGAAAGGATTAGCATTTTTCGGGATCCAATTTCAGCTCACACAAGGACTG

ATGACTAACATCTTCTCGGTTCTTGACAAGCATCAGGCTGAGGTTTTGGCTGCTAATATCTCT
GTAAGTGACCATCAGCTAACGACATTGACAATCACAGTAACCATAGGAAATAATGAAAGTA
ATACAGTAGAGAGTATTCGGAGAGAATTGCTTCTTTTTTAG

>SlbHLH009
ATGGGTATTATTTCTCAACAACCATTGCTTTCTTCTCCTGAGTTTCTTAACAAATGTGACTTA
AGTGATCCTTTGTGGACAAAAGTTTTAGAAAAAGGTGTATACATTAGAAGGAGATCATGTTC
ATGGCTATGCAAGAAACATAATGGAGCGCGTATCATGAAGAGAAGGGTACGTGTAGAACGT
TCTAGAAGATCGAGTAATGAGGCTGAAAAGAATGTTAAGATGTTGGAGAAGTTGATTCCAA
ATTGTGAACCATTATCATCATCAATGGGGTTGGAGAGGCTATTTAGAGAGACAGCTGATTATA
TTTGGGCTTTAGAAATGAAAGTTAAGGTTATGAAGATGATTGTTAATGTGTTATCAACTCCTG
ACATTAATTAA

>SlbHLH010
AGTATCATGTCTCACATAGCAGTGGAGAGAAACAGGAGAAGACAAATGAATGAAAATCTCA
AGGTTTTGCGTTCCTTAACTCCTTGTTTCTACATCAAAAGGGGTGATCAAGCATCAATAATA
GCAGGAGTGATTGAATTCATCAAGGAATTACACCTTGTTCTTCAATCTCTTGAGGCGAAAAA
GCGACGAAAGAGTCTAAGCCCTAGCCCTGGTCCTACTACTCCAAGGCCATTGCAACTAAGT
CCTACACCTGAAAGTAGTCCATTTATTACTCATAATAATAACTTCAAGGAACTTGGAGCATGT
TGTAATTCTCCAGTGGCTGATGTTGAAGCAAGAATATGTGGGTCAAATGTTATGTTGAGAAC
AATTTCAAAGAGGATTCCAGGTCAAATTGTGAAGATAATCAATGTGCTAGAGAAACTTTCTT
TTGAGATTCTTCATCTCAACATCAGTACCATGCAGGATACTGTTCTATACTCCTTTGTCATCA
AGATAGGACTGGAGTGCCAACTTAGTGTGGAAGAGCTAGCACTGGAAGTTCAAAAAAGCT
TCACCTCATCTGATGTTTTATGTATCAACGAGATATAG

>SlbHLH011
ATGTATGCATCACAAAGTTTGTCTTTTCATCCAACTTCTGATCAGGAAGATGAGAAAAGTTT
TGTTCAAAATCATATTGCAGCATCTGGATTCGTGGAGCTGCAGCAGCAGCAGCAACAGTTT
CATTCTAGTGAAAACAACTTGAATATGCAATTAGAATTTGGGCATGACTCTAACAACACAAG
GTTTCATCCAAATTGGGAAGAAATTAGTTTTAATCCTTACAACAATCAACAACTTAGCTACC
CCATTTCTAATCCATCATTAGGCCTTTTGGGGGGTTTTCATCAAAGAACAGAATTAGCTTCAA
CTTCAACTAATCTTTTTTATGAACCCCCACAAATGAATATGCCTCTAAATCTTTGTACCCCAC
AATCTAGTTTATTCAAAGAGTTGTTTCACCTTTCTCCACATGGGTCCTCCTATGGGTTAGGAA
GCTCCGGGACGGGTTCTTTGTTTAGCCTTGGTCATGATCAGGAAGAGGTAACAGGTAATTTG
TACCATGATGGGAGTTTTCATGAGTTAACTGGAGATATGATGATTAATTCTGCAGCTATTAAG
AAGAGGATATTAGGTAAAGATATCAAACATCATGCTTCTGAGAAACAAAGGAGAGTTCATTT
TAGTGACAAGTTTCAAGCATTGAGGACTTTAATCCCAAATCCCTCAAAGAACAATAGAGCA
ACAATTATCGCGGATGCTATTGGCTACATCGATGAGCTAAAAATGAGAGTAAATGAGTTAAA
AGTTCAAGTAGATATAAAGAAAGAGAGAATCAAAAGGCGAAGATCAATGGTAGAAGAATAT
GGTGCAGTGATCATGGAAGATAATCAGGATGATCAACAAGTGATGATGAACAAATCTACTAA
TTGGCATCATCAAATAAAATCTTCCAAGAATAGTAATACTGAAGTTGATGTGAGAATTATGG
AAGATGAAGTGATTGTTAAATTTGTCCAACATAAGCAAATGCTCAAGGGAGTGAATTGCCTT
CTATTGGTATCAAAAGCACTTGATGAGCTTCAGTTGGATCTCCAACATGTTGCTGGTGGATT
GATTGGAGATCACTATAGCTACCTCTTAAACTCCAAGATTTGTGAAGGTTGTACAGTGTATG

CAAGTGTAATAGCTAACAAGGTTATTGACGTTTTGGACAAGGAACATGCAGACATTAATTAA

>SlbHLH012
ATGTATGTTGAAGAAAGTGTTTGTTATGATCCTGCTACTCATCATGTGCAGCATGAAGGACTA
ACAGAAGATGTTTTTGTCATACAAGAGCATACGTATCATAACAACAATGATTCCTCCCAACA
AGATGTAGCTGTAGCTGCTGCAGCGGCCGCGTTGGAGATTGAATTTCAACATCAATTGAATC
TTGAAATGGAACAATGTTATAATAATAATAATAATAATAATACTCATAACAACAACAATAATAT
TGTTAACGAAGGGTTATCTTGTGATCAAGCGAATTGGGGAGAAATGAATTTTCCTCCATACC
AAAATCAACAACACAATGATAATGGTAACAGCAACAACAACTTTCATCAACAAGATTTCTC
GAATCCGATATCTGAAACTCCATATTTAACAACCCCTGATCTTCTCAATATGTTTCCATTACCA
AGATGCACGCAATCATCTTTGCTTCCTCAAAAATCCCCAAATTTGTTAACTTCATTAGGCCTT
ATTGGTGACATTGATGGCGGCGGTGCGTCAACTTCAAGTGCAATATGTGATCCTTCTTCTTTA
CTACTCCCATTGAATCTACCCCCACAACCCCCTTTACTAAGAGAATTGTTCCACTCTTTTCCA
CATGGCTATGGTTTGAGAAACTTGAGAAGCAACAACAATACCTCTTTCTTTAATGGATTGGA
GGAAACAGATCAAGGTTTATACCAAGAAAATGGAGAAACAAGACCTTTTCAGAATGGGATT
TTTGAGTTTTCTGGTGGTATGAATGATATAGCCAAAAACAGAGATGGTATAAAAGAGACTAA
ACATTTTGCTACTGAGAGGCAGAGGAGGGTGCATTTAAATGACAAGTATAAAGCTTTGAGG
AGTATGGTTCCAAATCCAAGCAAGAATGATAGAGCATCAATAGTGAAGGATGCAATTGATTA
CATCAATGAGTTGAAAAGGGGAGTGAATGAGTTAAAACTTATGGCTGAGAAGAAGAGATG
CAATAAAGATAGGATTAAGAGGCAAAAGACAGAAGGTGGTACTACTATTTCCATGGATGGT
AGTGATGCAAAGCAAATAATGGATGAAGTGGAACAATCATACAATGGGAATTCATTAAGGA
GTTCATGGCTTCAGAGAAGGTCTAAGAATACTGAAGTTGATGTTCGGATTGTCGACGATGA
AGTTACTGTCAAACTTGTTCAGCAAAAGAGGATTAATTGTCTTTTCTCTGCATCTAAAGTTC
TTGATGACCTTCAATTGGATCTTCACCATGTTGCTGGTGGACTTATTGGTGATTACTATAGCT
TCTTGTTTAACTCAAAGATTTCTGAAGGATCAACTGTTTATGCAAGTGCAATAGCCAAGAAG
CTTATTGAGGTGGTGGACATACAGTATGCAGCTATTGCACCAACTAAC

>SlbHLH013
ATGTTTAGCTCAGAGCCAATATCTAGAGAAATGGATAAGTTGAATAGTTTCTTTTTTTCCGGT
GGTGGTGGGGGTTCTTCAAGCTTCAAAAATGGTGAAGGAATGGAGTCTGAGTTCTTTAGGA
GCAAAGAAATGATGGGTTCTGATTTTTTTCAGCAACAGTCACAGTTTCAGCAGAGTAATTCC
GGTGGGTTAACGAGGTACAGGTCAGCTCCAAGTTCGTTTTTCGCCGGAATTCTTGACGGAG
ACGGGAATAATTCCGGTGAAAATTTCATTACCGGTGATGGGTCTTCGAGTTCTGATTCGGAT
TCCATGTTCACCGCCTTATTGAACAATAACGACACCACCAACAACAATGGTACTCGAGATAT
GAATGATCAAAACCAGAAGAATCAGCTTCAGTTTGGTACTTCTTTGAAACAGGAGATTGGT
GAGGAGATTGAATTTGGGAATGAAAATGGTGTACAAAATAGATATGAGAACGGTGGTGTTT
CTTATAGTGTAGGGGTGCAAATGCAAACTAGAGCAAATTTGAGTAATGGAAATGGTGACTCT
GATCTCATCAGACAAAACAGTTCACCTGCTGGATTCTTCAATGGATTTATGAGAGAAGTTGG
GAATTTCGGAGCAAGTGTTGGGACTAACAGGGAAGCAAGTACATCAACTAATGGTTTCAAC
AATCATATAAGTTACTCAACTAATCAATCATCTACCTCAAACTTCATGCCTAGCATTGCTGAG
AATGAATCTTGGAACGACGCGTCGTTTAATTCCTTGAAGAGAAATAGAGATGGTGATCTGA
AAATGTTCTCTACTAATTTCAATGGAATGACTAATCAGAATGACGAATCAAGAAACTACACT
TCTTCTGGTTTATCTCATCATTTAAGCTTGCCTAAGACATCTTCTGAGATGGCTGCCATAGAG
AAATACCTACAGTTCCAACAAGATTCAGTGCCTTGCAAAATACGTGCTAAACGAGGCTGTG

CTACGCACCCACGAAGTATTGCAGAAAGGATGAGACGTACTCGGATTAGTGAAAGAATGAA
AAAACTGCAAGATCTTTTCCCAAATATGGACAAGCAAACAAACACAGCTGATATGTTAGATT
TGGCAGTTGATTACATTAAAGACCTTCAGAAACAAGTCCAGACACTGACCGATAAAAAGGC
GAAATGCTCGTGTACAAGTAAGCAGCTGCAATATTCAAATGGAACAACATGA

>SlbHLH014
ATAGCCAACTGTCAAACACATTTATTTTTCTTATATCTACCCTTATTCCATCTATTATCTTCTCT
CCAACACCAAAATTTTGAGAGAATAATAATAATAATAATAATAATAATATATTGTTGTTTTGAT
TCATTCATCACCAAGAAGAACACCTCCCTCTTATTTACCTTCGATATGAATCAGTGTGTACCG
AGTTGGGACCTCGATGACTCTACCGTTCCTAGAAAAAATCTTATACAAACTCAATCCAATTC
ATTAGCCGTTGATGTCCCCTCATTAGATTATGAAGTAGCGGAGCTAACGTGGGAAAATGGGC
AGTTAGCAATGCATGGGTTAGGGCCTCCACGGGCTAATAATAAGCCCATATCGAGCTACGGT
GGCACCCTTGAATCTATAGTGAACCAAGCCACCCGTTGCAACGACGACGTTCCTCTTCATCT
CCACGGGAAGTCCACCGTGGACAGGAACAAACAGAGTGGAGATGAGGTGGTTCCTTGGTT
CAACAACCACAACGCGGTTGCTTACGCTCCTCCGGCAACCGGTTTAGTCGCAATGACTAAG
GATGCGTTGGTGCCGTGTTCGAGGAACACCTCAAATTCGGACAACCAACGATCCGTGCACG
TGCCAGGGATTGACGGCTCCACCCACGTGGGGTCGTGTAGTGGCGCCACAAACAGTAGAG
ACTGGACGGTGGCGCCACGCATGAGAGTACGGCCCACAAGACGTGAGTGGAGTAGCCGTG
CGGATATGATTAGTGTAAGTGGAAGTGAAACGTGCGGAGGAGATAGCCGCCAATTAACGGT
TGACACGTTTGATAGAGAATTTGGTACGACAATGTACACTTCTACGTCGATGGGGTCACCGG
AAAACACCAGCTCCGACAAGCAGTGCACCAATAGGACGGGGGACGATCACGATTCCGTTT
GCCACAGCAGAGATCAGAAGGAAGGAGGTGATGATGAGGATGACAATGACAACAAAAAA
GGGTCTAAAAACTCCTCATCTTCTACAAAGAGGAAGAGGGCAGCCGCCATCCACAACCAGT
CTGAACGAAAAAGAAGAGACAAGATTAATCAAAGGATGAAGACACTGCAGAAGTTGGTTC
CAAATTCGAGTAAGACAGATAAAGCATCAATGCTAGACGAAGTGATAGAATATTTGAAGCA
ACTTCAAGCTCAAGTGCACATGATGAGTCGGATGAACATGTCACCAGCCATGATGTTACCAT
TGGCTATGCAACAACAATTACAAATGTCTATGATGGGTATGGGTATGGGTATGGGTATGGGCA
TGGGAGTTGCCGGAGTTTTTGATATCAACAACCTTAGCCGCCCCAATATCCCCGGACTTCCT
TCCTTTCTCCACCCTTCTGCCGCCTTCATGCAGCCTATAACCTCCTGGGATAACTCAAACTCC
GCGCCTTCTCCTCCCTCTGCTGCTATGCCTGATCCTTTAGCTGCCTTACTCGCTTGCCAATCA
CAGCCAATTAACATGGATGCGTATAGTAGGATGGCAGCATTGTACCTGCAATTCCAACAGCC
CCCAACTGGCTCTGGCCCTAAAAATTGA

>SlbHLH015
ATGGTTACTGGGAATATGTTGTGGAGTGGTGAGGATAAGGCTATGGTGGCGTCTGTTTTAGG
AAAGGAAGCTTTTGAATATTTGATGTCTGGCTCTGTTTCAGCAGAATGTTCTTTAATGGCAAT
AGGGAATGATCAGAATTTGCAGAATAAGCTTTCAGATCTCGTGGAACGCCCAAACGCCGCT
AATTTTAGCTGGAATTATGCCATCTTTTGGCAAATTTCGCGGTCTAAGTCAGGGGAATTGGT
GCTAGGGTGGGGGGATGGGTGTTGCAGAGAGCCTAAGGAAGCAGAGGAGCGTGAAGTTAA
AAAGATTCTCAATCTACGCCTCGATGATGAGGGTCAACAAAGGATGAGGAAGAGGGTACTT
CAGAAGCTGCATATGTTATTTGGTGGAACAGATGAAGATAACTATGCTTTTGGATTAGATAG
AGTTACTGATACTGAAATGTTCTTCCTTGCCTCGATGTATTTTTCGTTCCCTCGAGGAGAGGG
AGGTCCCGGGAAGTGTTTTGGATCAGGTAAGTATTTGTGGTTATCAGATGCATTGACATCTA
ATCTAGATTATTGTGCTAGATCTTTCTTAGCTAAGTCTGCTGGTATGCAAACTATTGCTTTGAT

TCCAACTGATGTAGGAGTTGTGGAATTGGGGTCAGTGAGATCGATACCGGAGAGTTTAGAG
CTATTACAGAATATAAAATCTTGCTTCTCGTCGTTTTTATCACTTGTTAGGGATAAGCAAGCA
GCAGGTATAGCAGCTGTACCTGAGAAAAACGAGGGAAACAATCCCCGCCTTTCCAACTCTG
GCGCTGTTACTGAACGAACAGATGGAAATCCTAAGATATTTGGGCATGATTTGAATTCTGGT
ACTCACTTTAGGGAAAAACTTGCTGTTAGGAAAGCGGAGGAGAGGCCATGGGACATGTAC
CAAAATGGTAACAGGATGCCATTTGTGAACGCACGGAATGGTTTAAATCCTGCTTCTTGGGC
TCAATTCAGTAATGTGAAGCTGGGAAAGCCTGTGGAGCTCTATGCTCCTCCAACCCCAGGA
CACAACCTGATGAATGGTGGGAGGGAAGAATTCCGCTTGAACAACTTTCAACATCAAAAAC
CAGCTGCTAGAATGCAAATTGATTTCACCGGAGCAACCTCGAGAACCATTGTTTCCCCAGC
ACACAATGTTGAGTCTGAACATTCAGATGTTGAAGCTTCGTGCAAGGAAGACCGTGCAGGC
CCAGTGGATGAAAAGAGGCCTCGAAAACGTGGAAGAAAGCCAGCCAATGGAAGGGAAGA
GCCTCTCAATCATGTAGAGGCAGAGAGACAGCGGAGGGAAAAGTTGAACCAGCGGTTCTA
TGCCTTACGAGCTGTTGTTCCAAATATCTCCAAGATGGACAAAGCTTCCCTCTTAGGAGATG
CCATTGCTTACATAACTGAGCTGCAGAAAAAACTAAGAGATATGGAATCTGAGAGGGAGCT
GAGATTAGGAAGCACTTCAAGGGACGCAATCACTTCAGAAGACAGCCCTAGTTCTGAGATT
CAAATCCGAGGACCCGACATCAACATAGAAGCTGCCAATGATGAAGTCATTGTAAGGGTGA
GCTGCTCACTGGAAACCCATCCACTATCTCGAATCATCCAAATATTCAAAGAGGCACAAATA
AACGTTGTTGAATCAAAACTTTCCGCGGGGAATGGTACTGTATATCACACATTTGTCATCAA
GTCTAGTGGATCCGAACAGCTGACCAAGGAAAAGCTGCTGGCAGCATTTTCCAGCGAATCA
AACTCGTTAAGGCAACTTTCACCGGTAGGGCAATAA

>SlbHLH016
ATGGAACAACTCGCGGTTTCCTCATCTCCCATGGCAGTAGCTCCTCCTCCGGTAGACGTTAA
CCAGGTGCCTTTAGGCCTACAACAGATGCTTCAATATGTTGTCAAAAGCCAACCAGAATGG
TGGGCTTATGCTATTTTCTGGCAGACCTCTAATGACGACGAGGGAAAAAACTTTTTAGCTTG
GGGAGACGGATATTTCCAAGGAGATGGTGTAGTTATTAACAACAAAGGCGGCGGCGGTAGC
AGCAGCAGCTTAAAGTCACAGGCTCAGTCCGAGAGAAAAAAAGTTATTAAAGGAATTCAA
GCTTTAATGGATGGTAATGGAGATACTGATCTAGTGGATGATGGTGATGTAACTGACACTGA
GTGGTTCTACGTGATGTCGCTGGCCCGTTCTTTCTCTGCCGGAGATGGATCTGTTACCGGTA
AAGCTTTTGGAAGTGATGATTTTTTGTGGATAACAGGTCCGGACCAATTTCAGCTTCATTAC
AGCTGTGAAAGGGCTAAAGAAGCTCAGATCCATGGGATTCAGACTCTGGTTAGTATTCCAA
CTTCAAATGGCGTGTTTGAATTAGGCTCCACTCAATTAATCAAACAGAATTTGAGCTTAGTT
CAACAGGTGAAGTCTCTGTTCCTCTGTTGTCCCCCTATTCAATTTTTAGAAAAAACAATTAG
TTTTGCCGATATTGGCCTTGTCACCGGCTTGCAACAAGATGACAATGACTATAAATTAAGAG
AAAACAGCAGAAAACCGCACCCTGTTGTAGCCAAAAAAAGAGGGAGAAAGCCTAAAGGC
GGCGAGGAGGATGCTCATATGGCGGCATTGAACCACGTGGAAGCGGAGAGACAGAGGAGG
GAGAAGCTGAACCACCGGTTCTACGCGTTGCGCTCCGTCGTTCCCAACGTATCGAGAATGG
ACAAAGCGTCATTGCTCTCAGACGCTGTGTCCTACATCAATCAACTCAAAGCCAAGGTAGA
TGAACTGGAGTTGCAGCTAATTGATCACACTAAAAAACCGAAAATCGTAACAGAATCATCA
TCAGCTGACAACCAGAGCGCCACCACCTCATCCGACGACCAAGTAATTAAAGCAGCTAATC
CCACAGCAGCCCCGGAGGTTGAAGTGAAAATAGTTGGCACAGATGCAATGATCAGAGTTC
AATCGGAAAACGTGGATTATCCATCAGCAAAACTCATGATTGCGCTTCAAAATCTACAAATG
CAAGTCCACCATGCCAGCATTTCATCCGTCAATCATCTCGTCCTTCATGATGTTGTGGTTAGA
GTTCCTCAAGGATTGAGCACCGAAGATGAACTAAGGACTGCTCTTCTTACTAGCTATGATTT

GTAA

>SlbHLH017
ATGGCTCTAGAAGCTATAACGCAACAACAACAATATCTTTATGCTTTACTCGGAACTACAAA
TTGGAGTATTGATTCTGGCTCTGATTATTACAACAATAATGAATTTGTTCCGAATCATCAAAA
TGTAACAGCACCTCAAGAGTACGAGGACTACTCAAATTGGAATTTGCCAGCTCCATTATTAG
TAGATTCCCAAAATGAATTTCATCAATGGGGTCAAAATTGTTCCTCTGTTTTAGATCATAATT
TTACAATTGTGGATCAACAGGAACAGTTGGAGTCTCCAATCGAAATGTCTAGTAGTACAACT
AGACCACGAAGAAGAAGAACAAGAACAAAGAAGAATGACGAAGAGATTGAGAATCAACG
AATTACACACATTGCTGTTGAACGAAATCGTCGAAAACAGATGAATGACTACCTCTCTGTTC
TCCGTACTCTCATGCCTGAATCTTATGTCCAAAGGGGTGATCAAGCATCAATTGTTAGTGGT
GCAATTAACTATGTGAAAGAGCTAGAGCAACAACTTCAATTCCTTAGTGGTAAGAAGCATTT
GACAAGTCAAAATCAAGAAGCAAATGGTGAAACTTCTCCATTTTCTGAATTCTTTAGCATTC
CACAGTACTCCACAACCATCACTGCTGCTGCTGCTGCTACTACTACTAGTGAAAATGTATGT
TGTGAGAATGAATATTACAGGAACCAGCAGCTACCAGCAACAGCTGATATAGAAGTTACAA
TGGTGGAAAATCATGCAAATCTGAAAATCAGATCAAAAAAGAGGCCAAGATTGCTTCCTAC
AATTATTTCAGGGCTTGAAAGTCTTAGGCTAACTGTTCTTCATCTCAATGTTTCAAAAGTTG
ACCAGTTTGTCCTCTGTTCTCTCAGTTTGAAGGTAGAGGAAGATTGCAAGATGAATTCAGT
GGAAGACATAGCAGCTGTTGTGAATCAGATTTTAAGTAGGATCCACGAAGAGGCTAATTAA

>SlbHLH018
GCCTTCTCCTTTTCCAATACAAAACTCGCCTCTTCCCCCTCTCATTTCCTTCAAACTCTTCTC
CCCCATCTCTCTCTCTCTCTCTACATAATCCTTCTCTCCTCCGCCACCTACCGCCGCCGTCTC
CGCCGTCAAACTCAAAACTCAGTTTGCATCTATTTACAGAAGAGAAGCAAACGTACAATTC
GCGAGAATCATTCCGAATTTTGCATCAGAAAATTGTTCCTTCTGATGGCGTCATCTCAAACC
GTCTCGAATCCCGATGTTAACTCCAGTCACTCGCGAGAATCCAAGCGCAAAAAGAGAAGA
AAAATTGGAGATGATGGGGAAATTGAGCAACAAACGAGCCTTGATCAATCCAGGTGGAGA
ACGGATACCGAGCAACAGATCTACTCTTCAAAGCTTCTCCAGGCTCTCCGTCACGTTCGCC
GGAGTAATGACAATCCATCTCCGGTTAATGCCGGACGCGCCGTCAGGGAGACCGCCGACAG
AGTCCTCGCCGTTACAGCGAAGGGAAGAAGTCGTTGGAGCAGAGCGATTCTCACTGGCCG
TCTCAGCCTCAGGCTTAGCCAAATCAATAAAAAGCATAAGAAAGCAAAACTGAATAGCGGC
AATATAAAATCAAAGAAACCAGCGGCGAAGAAGCGATTGCCTGCTTTGCAACGGAAAGTTC
GTGTTCTCGGCCGTTTAGTTCCCGGTTGTCAGAAACTCCCGTTTACGAACCTTCTAGAAGAA
ACTAACGACTACATATCAGCTCTACAGATGCAAATCAAAGCCATGACTTTTCTGACCGGCCT
CCTCTCCGCCGGTGGAGCAGGATCAGTTGCCGCTCACCCCGATCGGCTCGGCTAA

>SlbHLH019
ATGCCTCTCTCTGAGTTTTTGAAGATGGCTATTGGGAAGCCTGAATCTGGCCAGCAGAAGAT
CTCATCTACCTCAAATCTGTCATCTTTTCCTGAGAATGACTTGGTTGAGCTTAAATGGCAAAA
TGGTCAGATTGTGATGCAAGGACAGAACAGTAGTGCTAAGAAAAGCACTGTTCCTAATAAT
CTTCCGTCGAGTGCCTCAGGGGATCGAGACAAGTACACGGGAAATTCATCAACCTCTAAGA
TTGGGAAGTTTGGTCTGATGGACTCCATGTTGAATGATATGTCATTAACTGTGCCAACTGGT
GAATTAGATTTGATTCAAGAGGATGAAGGGGTGCCTTGGTTAGGATATCCAGCAGATGACA
GTCTGCAACAAGATTATTGTGCTCAACTATTACCTGAAATATCTGGCGTGACGGCAAATGAA

CAGTCTGGACAGAGTGTATTTGGTTTAATAAATAAGAGAGGTAGTTCTGACAAGATGATTGG
GGATTCACATAGTGTTCCTGTTCATAATGCTGTGAATTTTGAGCGAAGAAATACATCAAAGG
TTTCTCCCTCTTCCAGATTTAGCCCATTAAGTTCATTGCCATCTCAGAAAGGTCATGCGTCGA
TACCTACCCTAGAATCAGGAGTTTCAGATGTCTTTAGCAGTAAAAATAGCAATACTCCACTAT
CTGTTTTGGGGGAATCAAATCAAAGTAAAGCTTCAGCTGGTGATGCTAAAAGCAATAGAAT
TCAAAAGCAAAACATGCCTGGAAATAGGTCCAATTTGTTGAACTTCTCACATTTCTCAAGA
CCTGCTACATTAGTTAAAGCAGCTAAGCTTCAAAGTAGTACTGGGGGTTCAAATATCTCAGG
TTCACCTATTTTAGAAGCTAAGGGAAAAAAAGGAGAAGAAAAAGTGACAATTGGTGACAA
TCATGTTAGTGCAGCAGCAACTGAAAACTTCTTAACTTCTAAGAAGGATAACTTTCCCCACT
ATCCAACTAATGGGGTATCTTCCCAACTCGAGTCAAGACCATCTGGAGCCAGCTTTCATGAT
AGATCATGTCAGGCTGAACAGTCTGATAATGCATTCAGAGATTGTTCAAGTAACAATGACAA
CACCCATGATCATTTTACCAGTGCTAAGGCAACCAAGGATATTGCCGATGGTGAGAGAAATG
TTGAACATGGGGTTGCTTGCTCATCTGTATGTTCTGGTAGCAGTGCAGAGAGAGGATCAAGT
GATCAGCCTCTCAACCTAAAGAGAAAAACCCGAGATAATGAGGAGTTTGAGTGTCGAAGT
GAAGATGTTGAAGAAGAATCTGTTGGTATAAAAAAACCTTGTGCTGCTCGAGGAGGTACAG
GTTCAAAGAGAAGCCGAGCTGCAGAGGTGCATAATTTATCTGAGCGGAGGCGAAGGGATA
GGATTAATGAGAAGATGCGTGCATTACAGGAACTTATACCCAACTGCAACAAGGCGGATAA
AGCTTCGATGCTTGATGAAGCTATTGAATATTTGAAGACACTTCAACTGCAAGTACAGATAA
TGTCTGTGGGAGCAGGGCTTTGCGTCCCGCCAATGATGTTCCCTATGCAGCACATGCATGGA
GCCCAGATGCCACATTTCTCCCCAATGAGTTTAGGGATGGGTATGGGGATGGGATTTGGGTT
GGGTATGCTTGAGATGAATGGTAGATCTTCTGGCTACCCCATGTATCCAATGCCCTCTGTGCA
AGGAGGGCATTTTCCCTCACCTCCTATTCCTGCTTCCACCGCTTATCCAGGAATAGCTGTATC
TAATCGTCACGTATTTGCACATCCTGGTCAAGGACTTCCAATGTCAATTCCTCGAGCATCTCT
GGGTCCTTTGGCCGGGCAACCATCAACAGGTGCTGCTGTTCCTATGAATGTTGCAAGAGAA
GGGGTTCCGGTGGAGATACGGGGTGCACAGCCGAATTTGGATTCCAAAACTCCAGTACACA
AGAACTCACAGATAGTCCAAAATGCTGAAGCTAGCTGCCCACAGAATCAGACATGCAGTCA
GGTACAAGCAACAAATGAGGTTCTGGAAAAATCAGCACAAAAAAATGACCAACTCCCTGA
TGTCATTGGTAGTGCAGCAAATAGGTTGACCAACCGAACAAATGTGCCCGGAAATGAAGCT
GGTCCCAGTTTGTAG

>SlbHLH020
TCCATGGAAGCATGTCACAATGGAGTCAATTGGCGGCCAAACTTAGTTCCCACCAAAAATG
TGTATTCAAGTGATACTAAATGCTTTCTGCCTCACTTGATTGGGTTTAAGTCCCCACCAACTG
ATGCATCTACAAATCCTCAGAAGAGGTTTCTCATTTGTGATCAATCTGGAAATCAAACTAGA
TTTTTCTTTAGTCAAGGTCGTCCTGCTGAGGATGAAATTATTACACCGAAAGAAGTGTTTGG
TGCTTATGGTTTGCATCAAAATGAGAACCTGAATGTTGTAGTTGAGCAAAGATTTCAAGTGA
AGCCCGTTATTGGAGAGAAATCGGATGAAAGTTATGTTAATGGTGAAGAAAGTAATACGCTT
GAAGATACAGATGAGATCAATGCGTTGCTTTTCTCTTCTGATGACATTGAAGGCGACGAAGA
TGATGATTTTTATGGTGAGGATGATGAAGTAACTAGCACAGATCGCTCTCCTTGTGCAAAAC
AGGGGTGCTGCGGCTGCGGTGAGCATGAGCAACAGTCTGTGGAACTTACAGAAGAAGTTG
CCAGTTCTGATGGCACATGCAAAAGGCAAAGATTGCTAGATGGTGGGTACAAGAAATCATC
ATACATAGAATCTAGATGGCCCAATGATGATGTGGAAGCAAAATGTGTTAGAGGCTCTCTTC
CTTCTAGTGGGAAGGATAAGGATTCCAGTTTGAGCACTAGGGAAAGGAAAGTTAAAATTCG
TGAAACCTTGAGGATTCTTGAGAGCTTGATTCCTGGGATAAAGAGCAAGGACCCGTTGTTG

GTTATTGATGAAGCAATCAATTACTTGAAGTCTTTGAGGGGTAAAGCCAAAGCCTTAGGATC
TGAGCTCCCTCAAGAGTATCCCCCAGCCTCTTGTTAA

>SlbHLH021
ATGAAGCCCAAAGAGGAAACAACTAATTTGTCATCAAGCAGTATAACTTTTGCAGCTGATCA
TTGTCATAACAATATGGTTTGTCATCAAGATTCTTTTGCTAATCAGAATTATATGATGTTTAAG
GCTGCATCATGTCAAGGTGCTAATAAGAGTGTTAGCACTAATGGAAAATTAACACAAGCTCA
AGATCATATCATTGCTGAGAGGAAAAGACGCGAAAAGCTTAGCCAAAGATTCATTGCTTTAT
CTGCTCTAATTCCCGGACTCAAAAAGATGGACAAGGCTTCAGTTCTTGGAGATGCAATAAA
ATACTTGAAACAACTTCAAGAGAAAGTGAAGACGCTTGAGGAGCAAACAAAGAAAAAATC
AGTGGAATCTGTTGTATTTGTTAAGAAATATGAACTTTATGGAGATGGTGAAAATTCTTCATC
AGATGAAAATTACTCAAGTGGTACTGTGCCAGTTGATGAGGCACTCCCAGAAATTGAAGCA
AGAATATCTGAAAAAGATGTATTAATTAGAATCCACTGTGAAAAAAGCAAAGGAGTTGTTG
AGAAAACAGTTGCTGAAATTGAGAAACTTCATCTATCAGTCATCAATACATGTGCCTTGTCT
TTTGGAACTTCTGCTCTTGACATTACCATAATTGCTCAGATGGATGAGGAATTTGCAATGAC
AGTAAAGGATCTTGTCAAAAATTTGCGCTCAGCTCTCAAAGTGTTTATGTGA

>SlbHLH022
ATGGAACCTGTGGTAGCCATGTCTGAAGGTGAATGGAGTTCCTTAAGTGGAACATGTTCTAC
CGAGGAGGCGAATTTCATGGCGCAGTTATTTGGTGCCTGTCCAAATGAACAACAACTACCT
AGTAGTGGACTTCCAAATTTTTGGACAAATCATGAATCAAACATTGGAGGAAGTAGTGAAG
TTTCAATTTTTTCTTCTCAACATCATACTAATAGTAGCATCTACCATTTCCCAACTAGTACTAA
TCATTTTCAACCAATGTTATTGACAACTTCAATGACAATGGAACATTTGCCTCCAACTAACA
ATTTAATCGAAGCGGATGCTGTTGAATTTTTGAACAAACAAGTGAACAATGACAGTATTGAA
TCAGGTGAAAACATTATGTCTGAATCTGTTCTTCATGGAAAGAGCTTGCAGCTAGGAAGAG
AATATGATCAAATGCATCAACCAGAAAGCTCTAAGAAAAGATCACAATCGCCTGTTGATCAT
AAGAACAAGAGAAGCGTAAAACCAAAGAAGAACATGAAGAGTAGTGTTGCTGATGATGAG
GAGACTGGAAACAATAATAATAATAATACTGTGCTTCATAGACAGAGCTCATTTAGTTGCTGT
TCAGAAGATGAATCTAATGTTTCTAGTTATGATATCTATGGATTAGCTTCGAGCGATAACTCA
AAAGGAGTTTCTTTGCCCAATGGAAAATCCAGAGCCAACAGAGGCTCAGCAACAGATCCT
CAGAGTCTGTATGCAAGGAAAAGAAGAGAGAGAATTAACGAGAGATTGAGGATCTTGCAG
AGTCTCGTCCCTAACGGAACAAAGGTTGATATTAGCACCATGCTTGAAGAGGCTGTCCAGTA
TGTCAAATTTTTGCAACTTCAAATCAAGCTGTTGAGCTCTGATGATTTATGGATGTATTCTCC
CATAGCATACAACGGAATGGACATTGGACTTGATCTGAAGATTGGAATTCCAAATCCAAAAC
CGTAA

>SlbHLH023
ATGGCGGGTGAAGAAGAGAGTTCACTGGACGGAGGAACGTATTCGGAACTACTATTTGCCG
ACGACGATGATGGCCTAGCAGGATGTTTCAATTTCACTAATTCATCTTCCCCGAAAATGCTCT
GTTTTGGTACTGATGCTCCTATACTTGAAACTTGTTCTGTACAAACATCAGAACAAAAAACC
CCAAAATCTGAACTCACATGCAGTGGAGATTCACCATCTGCTTGTTCAAGTAGCAACATCA
GCCAGCCTAACAACTCCAATAAAAGGCGAAATGGTGCAGAGAAAGAACCGGTTGAAAAAA
CAAAAGGTAGAAATCAGAGAAATTGCAAGAGGACAAAGATGGTAGAAAATTCAAATGTGA
CAACTCATGCAAAGGTTAAGAAAGAAAAGCTTGGTGAAAGAATCACAGCATTACAACAAC

TTGTATCTCCCTTTGGCAAGACCGATACAGCATCAGTGCTACATGAAGCGATGGGATATATCA
GGTTTTTGCACGATCAGGTTCACGTCTTGTGTTCTCCTTACTTGCAGCGCCAGACCCAGAGA
CAGTCTCCCTCTTTACGTGAGGGCGGGGAAACGGAAGCATCGAGAAATGAGGTGTTGCTG
AGGAGCAAAGGACTTTGTCTAGTGCCAGTAGAGGTGAGTGTCCATGTAGCTGATACTAGTC
TCAACGGCGCTGATTTTTGGTCACCTGCCGCCATGATGAACAATAATAATAGCATTACCCAAT
GA

>SlbHLH024
ATGAATATAGATGGTGAGAATAATCATGGGTTGCCATGGGAAACTAATGATTTCTGGTCATAC
TTGAATGACAATCAGGTTGGGAGTGAAGAGACGTTTGATGGTGACAAGGTGCCCGGTCCG
ACTAAGTCTGATATTTGTCAGCAACTAACAATTGTTAATGAGGTGGTTGAAGTAACACCAGC
TGTAGGTAAGAAGAGAAGTCCACCAAATCGAAAAAGCAACGGTAAGGGAATTGCTGAACC
CAATCTTGATGTTGGTGGAGCTGAAGGCAAAAGAGAATCTGAACATGAGATACACATATGG
ACAGAGAGAGAAAGGAGAAAGAAGATGCGAACCTTGTTTGAAACTCTTCATGCGTTGGTT
CCTAATCTCCCTGCTAAGGCTGATAAGTCTACAATAGTTTATGAAGCAGTAAACCATATAGTA
AAACTGCAGAATACTTTCAAAAAACTTAAAAGTCAAAAGCTAGAAAAGCTAGAAGAATAC
AACATAGGATTGGCGGGTTCACAAAAAGTTTATAATAGTTGGGAGAAGTATGTGGTTGATCA
AGGATCAACATGTAATTCTACAGCTATTACACCAACAAATCATGGTGCTAGTCCCCTCATTCC
AACAGGTTTTATGACATGGAGTTCACCAAATGTGATACTAAATGTATGTGGTGAAGATGCAC
ATATTAGTGTGTGTTGTCCTAAGAAGTCAGGGCTATTTACCATCATTTGTTATGTTTTGGAGA
AACATAAGATTGACATTGTGTCTGCTCAAATTTCATGTGATCAATTCAGAAGCATGTTCATGA
TCCAAGCTCATGCAAAAAGTGGAAGAGACGTAGCTCAATTCTCGGAAGCATTCACAGTGG
AAGAAAGGTTGAAACAAGCTGCAACTGAGATAATGGCACTGGCAACCTCCAAATGA

>SlbHLH025
ATGAATGAAGGCGGCGAGAATAATCATGATCATTTGTGTGATGATATGTGGTCACACCTAGA
CTGGAATGACCATCAAGTTGAGAGCGGAGAGATCGAGGGTAACAAGTTGCTAGATCCGACT
GGGTCTGATACTTGCCAGCCATTAACATTTATTAATGAGGTGGTTGATGTGAGTGTTAATGTC
GCCAAAAAGAGAAGTTCAGCCAACCGAAAAAAAAAGGGTAAGAAAATTGCTGAACCAAA
TTCTAGTGTTGATGGAGCCGAAGTTAGAAGAGCATCAAAACATGAGGTACACAAATGGACA
GAAAGAGAAAGGAGAAAGAAGATGCGGACCCTATTCGAGACTCTTCATGCTTTGGTTCCCA
ACCTCCCTGTTAAGGCGGATAAGTCTAAAATAGTTTATGAAGCAGCGAATCACATTCGAAAT
CTGCAGAATACTTTCAATAAACTTGAAAGTCAAAAGCTAGAAAGGCTAGAAGAAAATAACA
TCATGTTAGTGGGTTCACAAAAAGTTGGTAATAGTTGGGAAAAGTATGGGGGTGATCAAGG
ATCAATCTGTAATTCTAAAGCTATTACACCAGCTAATCATGGTCCTACAGGTTTTATGACATG
GAGTTCACCAAATGTGGTACTAAATGTAGCTGGTGAAGATGCACATATTAGTGTTTGTTGTC
CTAAAAAGCCAGGGCTATTTACCACCATTTGTTATGTTTTGGAGAAACATAAGATTGACACT
GTGTCTGCTAAAATTTCATCTGATCAATTCAGAAGCATGTTCATGATCCAAGCTCATGCTAAA
GGTGGAAGTGGGGTAGCTCAATTCTCCGAAGGATTCACCGTAGAAGACATGTACAAGCAAG
CTGCAAATGAGATAATGTTGATGACAACCCCCAAATGA

>SlbHLH026
ATGAATGAAAACGGCGAGAATAATCATGATCATTTGTGTGATGATATCTGGTCATACCTAGAC
TGGAATGACCATCAAGTTGTGAATGGAGAGACCGAGGGTAACAAGTTGCTAGATCCGACTG

GGTCTGATACTTGCGAGCCATTAACAGTTATTAATGAGGTGGTTGAAGTGAGTGTTAATGTT
GACAAAAAGAGAAGTTCAGCCAACCGAAAAAGAAATGGTAAGGAAATTGTTGAACCAAAT
TCTGGTGTTGATGGAGCCGAAGTTAGAAGAGCATCAAAACATGAGGTACGCAAATGGACA
GAGAGAGAAAGGAGAAAGAAGATGCGAACCCAGTTCGAGACTCTTCATGCTTTGGTTCCC
AACCTCCCTGCTAAGGCTGATATGTCTAAAATAGTTTATGAAGCTGTGAACCGTATCCGAAA
GCTGAAGAATACTTTCAAAAAACTTGAAAGTCAAAAGCTAAAAAGGCTAGAAGAATATAAC
ATAAGGTTGACGGGTTTACAAAAAGTTAATAATAGCTGGGAGAAGTATGTGGGTGATCAAG
GATCAACCTGCAATTCTATAGCTATTACACCGACTAATCATGGTGCTAGTCCCCTTATTCCAA
CAGGTTTTATGACATGGAGTTCACCAAATGTGATACTAAATGTATCTGGTGAAGATGCACATA
TTAGTGTGTGTTGTCCTAAGAAGCCAAGGCTATTTACCACCATTTGTTATGTTTTGGAGAAA
CATAAGATTGACATTGTGTCTGCTCAAATTTCATCTGATCAATTCAAAAGCATGTTCATGATC
CAAGCTCACGCTAAAGATGGAAGTGGGGTTGCTCAATTCTCTGAAGCATTCACCGTGGAAG
ACATGTACAAGCAAGTTGCAAATGAGATAATGTTGATGACAACCCCCAAATGA

>SlbHLH027
ATGAATATTGCATTACCAGAAATGCTACATAACATCACTAGCAATGGAAGCTCAGAATTGAG
CGTGCTTGATAGAACGAAATGGCAGGTGCAGCAACAAGAGATGAGTTATTTTAATGGACAA
AATGATCAACTCATGAATTCTTTTCATCAAACGGCTGAAGCTCAACAATTTCATGGTCTGAT
CAATGTAAACGATCAGAGTCTTAATGAGCTTGTGACTCGGGCAATAAAGCCAGACCCCTGTA
TGGAGAACAGTTGGGGTGGTTTTGGGACCACTGGTACTAATGGTTTTGATTATGTTCCAGTT
GGAGTTGGACACGGAGGAATGTCACACCCATCTGAAATGAACTATGCTATTTCAAGAACTA
CAAGTTGCCCGCCTACCATGGCGGACAATGTTGTTAAACCCAAAGACACTAGGCTGAGTTC
TAACAGAGGCAGAGAGAGCTTCAAGAAAAGGAAAGCAGATAAGAATCAGCATCTCAAGGA
GGTTGCAGAAGAGGAAACCAAAGACAAGAAATTGAAAGAATGCATCGAAGAGGAGGATG
ATTCTTCCAAGGTAACAACAGAGAAAAAAAGCAACAAAAGGAGTGCGACTAACAGTAGCA
ACAGTAAGGAAAATTCTGATACTTCAAAGGAGAAGTCCAAAATTACCGATGATAAAAAGCT
CGACTATATACATGTCAGAGCACGTCGAGGTCAAGCCACTGATAGTCACAGTTTGGCTGAA
AGAGTAAGAAGGGAAAAGATCAGTGAGAGAATGAGATTCCTGCAAGATTTAGTACCAGGT
TGTAACAAGATAACAGGGAAAGCAGGAATGCTAGATGAAATAATCAACTACGTCCAATCTC
TCCAAAGACAAGTAGAGTTCCTATCCATGAAACTAGCTGCTGTTAATCCAAGGCTTGATATC
GATGCAGACAATTTCTTCAACAAAGATATATTTGCAACTAGCACGTCTACTTTTTCCGCAGT
GGGAGCTGGAACATCATCTGAAATGCTTAGTATGGCCCAACGCCAGTTCAATTCATTGCAGC
AAATAATGTCAAGTTCTGGATTAGAAATGGGTATTGTAAATCTAAACGAAATGGCACTACGT
AGAACCACTAGCGCTCCTGTACCAATTCCTGAAATGTTTCTCGACTCATCAAGTATCAATCA
AGTTCAGAGCTTTCAAACTTGGAACACTGATTTAGATAACATGTATGCAATGGAGCTTCAAC
AAGGAAGATCAGCACAGTTTCTTCCCCATCCGTGTACAGGTTTTGCTGAAGCTGGACATGA
CCTGAAGATGGAAATGTGA

>SlbHLH028
ATGGATCAATTCAACCATGGCGGCCTCTATCAATCGAATCAGCTTCCAAATCATTGTTTAACA
GAGTTAAATCAGCTTCCTTCTGATGTTTCCACGCCTCCCAATGGCTTGCTTTCTGAATCAAG
CAAACAAAAACCTGAGGCAGAACTCAAAGACTCTATTGCTGCAAGAAAAGTGCAGAAGGC
AGATCGAGAAAAATTAAGGAGGGACCGCTTGAACGAGCAGTTCATGGAATTAGGAAAGAC
CCTTGATCCTGATAGGCCTAAAAATGACAAAGCATCCATCCTAAGTGATACCGTTCAAATAC

TGAAGGATTTGACTGCTCAGGTCAGCAGATTAAAATCTGAGTATGCTGCACTTACTGATGAA
AGCCGTGAGTTGACCCAGGAGAAAAATGATCTCAGAGAAGAGAAGGCATCTCTTAAATCT
GATATTGAGAGCCTTAATGCCCAATATCAACAAAGAATGAGGACTATGTATCCATGGGCCGG
GATGGATCATTCCATGGTCATGCATCCGCCTTCATATCCGTATCCAATGCCCGTTCCAATTCCA
ACTGGACCAGTTCCTATGCATCCACCTCTGCAGCCCTATCCTTTCTTCGGCAATCATAATCCT
GCAGTTGTTCCAAACCCTTCATCTTTTGTTCAATACATGACTCCCAATACATTGATTGAACAG
CAACCGACTCAGTATATGTCTCCAATTATACAACCAGGTAGTATGACCAGGCAAGAATCCAG
GAACAAGTCATCAGATCAAGGAGAGAGCAGAATTGAGAAAAGTGAAGATTCCAATGAAGT
GGCAACAGATTTAGAGCTTAAGACACCTGGATCTACATCCGAGCAGGACCTTTCATCTGGA
CAAAAGAAATCCAGAAAGTTGCCAAGGAAGGATAACAGCTTCACGGATGGAAGTTCCTCA
AGTAAATGTTCATCATCCCATAGTGTACATGCTGTTTCTTCAAATAGTGTAGTTAGGGGAACA
AAGACTGGTGATTGA

>SlbHLH029
CTTCATCAATATCAATTTCTACTTGGTGTTGATGAGATTAATAATAGTTCTTTGATTAGTGGAG
AAAATATTGCTGCTAATTCTCATGATCACAATCAACATATTTGGAGCCAACTTCTCTTAAGCA
ACTCTACTACTAGTGGATCAAGTGGGAGCATTAATACTAAAAATCTATTGGAAAACAATATG
GAGATGAGTTTATTAGATCATGGATCAACAACAACCTTAATCACCTCTAAATCTTGTTTTGAT
CAAAATCAATTAGCTAATAATCATGATGATGATGATGATTATACATTTCATCCTTATAGTAATCT
TCTTGGAAGGCCACTAAATTATTATACAAATAATAATAATAATAATAATGATATGGACTCTATG
TTTCCAATGAGAAGTGATCAAATTGCTCATCATGAAGAAGTGGATAATTCAAGAAACTTGGC
TAGTATTTGTTTTGGTGATTATTTAAACAATAAGCCATTGGTGGACTTTAAACCAAGTCTCAA
AACTTTGAATTTGGGACAACATAAGAAGAATGGACTCCAACAACCTTATATAAGCAAGTCTA
GGGTTACACTATCAACATGCAACACATTGAAGAGAAGCAATGGAAGATCACAAGAATATAA
TAATCCAATTGATGGAAAAAAGAGGAAATTAGAAGATAATTTAGAAACTAATTCAAAAAGG
CTAAAGAATGAAAATTCTGACGTCACATCTACAAAGACACAAATTCCAAAAGTGAAACTTG
CAGACAAAGTTACAGCTCTCCAACAAATTGTCTCTCCATTTGGAAAGACTGATACGGCATCT
GTTTTGTGGGAAACAATCAACTATGTTAGATTCTTACAAGAGCAAATACAGCTACTAAGCCA
TGCCTACATGAAGAGCAATACATGCAAGGAAAGGTATTGGGGAGTATTTGATAGAAAAGAA
ATTGATTTGAGGAGCAAAGGACTTTGTTTAGTTCCCATTTCATGTACCCCTCAAATATATCAT
GAGACTAATAATGGATCTGATTATTTAATCCCATCATATAGAGGGTGTTTGTATAGATAA

>SlbHLH030
ATGTTACAAATGAGTGTTCTTGAAAGACAAAGAGCAGTGTTGGAACGTATTTATAATCATTC
TAAGCAGCAGCTTTCTTCTCTAGTACCTCAACAAGAACTTGCTCATTTGATTACCGGCTGCG
TTCAGGGGAATTTTAACATGTTTGGCGGGGGAGATTCTAATTTTGTTAATTTTCAAGAAATG
GCTCGTCCCTCTTTCTCCACAATATCGAATTCATCGATTACAACAGTGTCTCCGCCACCAGA
GAAAGAAAGTGACTTAAGTAGTATGATAGCTCCAAGGGAAAATGTTGTTTCTACCAAGAAG
AGAAAAGCAGAGTTTATTGAGGAAGAGGATTGTGAGAAAAGCCCGGGGAATGATTCAAAG
GAGAATTCGAAGACGTCTGAGGTTCAAAAACCTGATTACATTCATGTAAGGGCACGTCGTG
GCCAAGCTACTGATAGCCATAGTTTAGCAGAAAGAGCTAGAAGGGAGAAAATCAGTAAAA
AAATGAAATATTTACAAGATTTAGTTCCAGGTTGCAACAAAGTGACTGGCAAAGCTGGAAT
GCTAGATGAAATCATAAATTACGTTCAATCGCTTCAAAAACAAGTCGAATTTCTCTCCATGA
AGCTTGCTACTCTGAATCCTAGGCTTGATTTGAACACCGATAACATTTTCGTTAAAGATTTAC

CTTCTTACATGACCACTACTTTTCCACCAACAGTAGCAGTTCCAACACTTTCAGAATATAAC
ATGATCCAACACCAGCAAGCTGGAAGCACCGGTGATGTCGCACAAATGTTACCCCAAAGA
AGAGATTTAATGTCATTTCCTGACACTTATCTTGGTTCTTCACATGTTACGGTAGTACAACCG
CAACAACCAACTTTTGAGCCTGATCTGCAGAGCTTATTCAGTGTCGGATTTAACTAG

>SlbHLH031
CATATTCCTTCAGCATTATATTATACTTTTTGATATTTGAAGAGAGAAGAAGAAGAAGAAGA
AAAATGGAGCTTAGTGAACATGATATTTGGAGGAATTATTAGCTCTTCCAAGAAAAGAAAG
TTTGAATGATTTTTTGCCACATGGAAATGGATGGACTTTTGAATCACCACTACCTTTTTATCA
AAACCCTGAATTTATTGCCTTAAATTCTTCACTTTTGGGGCTAATTTCACCCCCAATTACTAC
TTCACACTCTAATTTTCCTGATTTTACTTCACCTGAATCCTATCAATTTCTAGATTCTTCTTTTA
CTGGTACACCATTACTTGATGACTACAGTGTTATGGAAAATCATGAAGAATTTGGTGGGATTA
TTCCTGGTGATTTTCATGGATTGCAACAAGAGTTGAATAGCTTTGGTGATGTTAAAGTGGAA
GAGTCAAATTCAAGATTAATGGGTAATGTGGGAGAAAAGAAGAGTAAAATTAGGAAGATGG
AAGGACAGCCTTCAAAAAATTTAATGGCAGAAAGGAGGAGAAGAAAGAGACTCAATGATA
GACTCTCTATGCTTAGATCTATTGTTCCTAAGATTAGTAAGATGGACAGAACATCTATACTTG
GAGACGCAATTGATTACATGAAGGAGCTTTTAGAAAAAATCCATGCATTGCGTGAAGATGAT
AATGTGAAAGACGAAATTAAGGATATTAAATTCGTAGGAAACTTCAAGGAGTTAAAGCCAA
ATGAAGCACTTGTTAAAAAGCCTCCTAAGTTTGAGGTAGAAAGGAGAAATGCAGACACCA
GAATCGAGATCTGTTGCAGTGCAAAGCCAGGGTTGTTGTTATCGACAGTGAGCACGTTAGA
AGCTCTTGGGCTTGATGTACAACAATGTGTTATCAGCTGTTTCAGTGACTTCTCATTGCAAG
CTTCTTGTTCTGAGGCAAGGGAGCATCGAACAATTTTGAGCGGTGAAGATGTAAAGCAAAC
CTTGTTCAAAACAGCAGGCTTTGGGGGAAGATGTCTTTAG

>SlbHLH032
ATGGGTTCACAGCCAAAAAAATCGAGTAGCTTGCGGCTAAGGAGATCGAGAAGAAATTCTT
CCGTGGAAAAACGGTCGAAAAATGGAGTAGTTAACAACAGTAGCAATGCCGCCGGCGATG
ATAATTCGATTTCAATTTCGGAAAAATTGGAAGCTCTGAAGCAACTTCTCCCAGTCAACAAT
GGCGAACTAAAAGCAGATCAGTTATTTGAAGAAACAGCTGATTATATTGTTCTTCTCAGAAC
TCAAATCTTCGTTTTACAGAAACTGCTCGATTTTTGTGACGATGCATCTGGTCAGTCCCAAC
ATATTAATGCTGTATAG

>SlbHLH033
AAGAATCATCTCAAAATATGTCTGGGAGAAGGTCAAGGACGCAGTCATCAGAAGGAGGCA
CCTCCAGGATTTCAGATGATCAGATCATACAACTCGTCTCCAAATTGCAGCAACTTCTTCCT
GAAATTCGTAATCGTCGCTCCAACAAGGCATCAGCATCTAAGGTGCTTCAAGAAACATGCA
ATTACATTAGGAATTTGCATAAAGAGGTGGATGATCTTAGTGATCGACTTTCTCAATTATTATC
AACCATTGATGCTGATAGTCCAGAAGCTGCAATTATTCGTAGTTTAATTATGTAATTTTCAATC
ATTTATTTATATATAATTATAATTACTATATTATATATATTTGGATGCACTAATTAATCTACTAAAT
ATATACATATATAGTTCTGTATTTCTTCTTAATTCGATCTCTAGATTTTGGTCAATATTAATTAGC
TAG

>SlbHLH034
ATGCAGAATAACTATCAATTTTCCCCTCAAATTCAAAAGCCTTTTGTCTCTCTGGAAGACCA

AGCAGCTGGTGGTAATATATTTGATATTCCTGTTCCATCCGTCTTTGATACAATGGCACTTCCT
ACTAGTTTCAAATCTTCTGTGCCCTTTCATGGTTTTGAATTTCGGTCCTCTGAGGCATGTCCA
AAGAATTTCATCATCTTTGATCAGACTGATTACCGGAGCCAGATCATGTACCACCCTGCCAT
GACCTCCAAATTCCCATATCCTGATCTGAATTATAATTCAACTTGCTTCCACGACTGCATGGA
GAGAAAAATTGCAAACAATGAGAATACAGAAGTTTCTTCATATCTGAAGGAAGATTCAGAT
GATATAAATGCATTGCTCAGCTTGGAAGAGGAAGAATGTGAGGAATATGATGAAGAAGAGG
TGAGCACTGCACGCACTGATGCAAACTATGGATGCAGTTCCCCTGAATCATACTCAAACTAT
CATTGTCAATCCAAAAAGAGCAGGACCTCTTCTTTCAGGGAATCCTCTGGTAGCAGTACTA
GCAATTGCAGTGAGAGAAAACGCAGGAAACTTAAGAAGATGGTCAAGGCATTAAAGGGAA
TCGTCCCTGGTGCCAGCAGGATGAATACTGTCACCGTTCTTGATGAAGCTGTCAGATACCTC
AAGTCACTTAAGGTGGAAGTGCAGAAGCTTGGAGTCGATAATCTGAAGACATATGCCTGA

>SlbHLH035
ATGAACTGTAGGAATTTAGGTGAGTTTTGTGAGAATGAGGCAAAAGGGGTTGTTCAAAGTT
TGGTGTTGGATAGTGAGAAAGGTGAATTAGTGAAGGCTAGTGGAAGGGTGGAGAAAAAAA
TTGGAAAATCAGAGGGGAAAACAATTGCTGCATTGAAGAGTCATAGCGAGGCAGAGAGGC
GAAGAAGGCAGAGGATCAATGCTCATTTGTCTACACTCAGGAATCTTGTGCCATCTTCTGAT
AAAATGGACAAAGCAGCACTGTTAGCTGAAGTAGTACGTCAAGTGAAACAATTGAAGGAA
ACAGCAACTCATGATAGTGAAAGGTTCTTCATACCATTGGACTCTGATGAAATAAAAGTTGA
AATAATCGCTGAAAATGCAATAGATGGGACGTGTCTTTTCAGGGCGTCCGTTTGTTGTGAAT
ACAGAACTCATCTTTTATCAGATTTAAAGCAAACTATCAATTCTCTTCATGTCAATTTAGTGA
AGTCAGAAATATCAACTTTAGGAAGTCGAGTGAAGAACGTGTTTCTCTTCACGAATTCGATC
CATGGAGGAGGAGGATGTGCCACTATTCAGGCTCGAGATATTTTCTTATCCTCTGTTCGACA
GGCGTTTAGTTCTGTACTGGATAAAGTTTCTGCGTTTCCAGAATATTCAGCCTATCCGAACA
AGAGGCAACGCGTTTCTTGCTTCGATTCTTCAAGCTTGTTATTCTGA

>SlbHLH036
ATGGAGCAGCAGTACTGTTCTAACAAACCTAGTTCCTCATCATCATGTAAAGCAGCTGATAG
AAAAACAATTGAGAAAAACAGAAGAAATCAAATGAAGGATCTATATATGAAGCTCAATTCA
CTTGTGCATCATGATCAACACACTAAGGAATTTTTGTCACTGCCTGATCAACTTGAAGAAGC
CGCGAATTACATAAAGAAACTGCAGATAGATTTGGAGAAAATGCGACTCAAAAAAGAAGC
CTTAACAGCAACAGCTGGTACTTTAAATTCGAATTCAAATTCAAGCAGTAACACTGATGGGA
GAACATTGGAAAATACATTGCCATTGCCACATATTGACATTCACAATGTGAATTCAGCTCTAG
AAGTGCTTCTAATTACTGGATATGATTATCACTTCATGTTCAACCATATCATACGTATGCTTCA
TGAAGACGGCGTTCAAATTATTAGTGCAAATTATACTCTCGTAGGTCACACCATATTCCATTC
CATACATTCTAAGGTGGGAGAAAGTGCAACGTCGTCTACAGCAAAGATTATCTCAGAGAAA
TTAAAGCAGTTTGTGGGTGCAGCTGCTACCTAG

>SlbHLH037
ATGGCTGATGAATATCAAGCAAGTGTTTGTGGAGGAAACTGGTGGAATTCAAGTAGAAGTA
TATTTGGTTCATCACTATGTGCATCTTCAGTTCCTTTAGGAAACTCTAATTTTGCATGGACAA
ACGATCATTTGTTGGATATGAAATCTTCCTGTAGGTCTAACGATGAATCTGGTAATTCCGATG
AATCTGTCGTTCTTCAAGAATTACCTAAACATGATTCCACCCTACAAATCTTGGGTAGTGGC
CTTAATTCTTCATCAACAAATGATAATTGGAGTCATACCTTGATGCATGGAAATGATAGGAGT

GAAAGTAGTTATCCTTCAATACTACAGCAAGAAGATATAAATTCAAGCATGAATTACCAACA
AGAAAGTGGTGTTGATTGTTCAAGCAACTCATTTAAGCAAGATTTCACTTTAGGAATGAATC
ATCCAATCACTAGCTCAACTAATACTCATCATGATCAAATTTCTTCAACCTTTCCAATGAATT
CATCTTTCTCTAATTATCCTTCAGCTTTACTTCAAACTTTATTTGATAACGATCCTCCTCAACA
ACAACAATTACAACAATCTTTGTTTGCTACTAATAACAACCAACCAATGAATTTCCCAACAT
CATCCTTAAACTATAGGCCTGATTTGAATGACTTTTCACCTTCTATGCCTAAATTCCCTAATTC
CTTGTTGATACCAAAACAAACTACAACACCTTCGAATCATTTTCCAAATTACTCTCTTAATGC
TACTGCCTCTCTCTACAATACTTCATCAGCCACATCCTTAAACAATATGCGCGCTACTCTTAT
GCCTTCTATGCATCCACAAATTCTCCAGTCACCAACATTTAACGACAATTCCAGAGCTCCTA
ACGTTACTCCAAAGAGTAAAGTTGAAGATTTAAGAGAGTCGAGGGTGAGTAAGAAAAGTG
TGACTAATGAAGCAACATTGAAGAGGGCAAGAATAGAGACACCATCACCATTGCCAACTTT
TAAGGTCAGAAAAGAGAAATTGGGGGACCGTATTACTGCTCTCCAGCAATTGGTTTCACCT
TTCGGAAAGACTGATACAGCTTCAGTTCTCCATGAAGCTATTGAGTACATCAAGTTCCTCCA
TGATCAAGTCAATGCTCTTAGTACTCCTTATTTGAAAAATGGATCAACCACCACTCAACACC
AACAGATTGCTGATAAGGTGAAGGAAGAAGATTTAAGGAGCCGAGGATTGTGTCTGGTACC
GATATCGAGTACTTTTCCAGTAGCAACTGAAAGTAGTACAGATTTTTGGACTCCAAATTTTG
GTGGTACGTTCAGGTAG

>SlbHLH038
ATGGAATTCCCCAGTACCCCATTTGATAATTCAAACAACTCTGAAGAAAGGGAAGTAGGAA
GAAGAACAGATAAAAGGAAGCAAATTGATGGTGAAGTTAAAGAATACAAATCCAAGAACC
TTAAGGCTGAGAGAAATAGGCGTCAAAAACTTAGCGAAAGGCTTCTTCAATTACGCTCATT
GGTCCCAAACATAACAAATATGACAAAAGAAACCATAATCACTGACGCCATCACCTACATTA
GGGAGCTACAAATGAATGTGGACAACCTAAGTGAGCAGCTTCTTGAAATGGAAGCAACTC
AGGGGGAGGAACTGGAGACAAAAAATGAAGAGATTATCGATACTGCAGACGAGATGGGTA
AATGGGGCATAGAGCCTGAAGTTCAAGTGGCTAACATTGGCCCAACTAAGCTTTGGATAAA
AATAGTCTGCCAAAAGAAAAGAGGTGGATTAACTAAACTGATGGAGGCAATGAATGCTCTT
GGATTTGATATAAATGACACCAGTGCCACTGCCTCTAAAGGAGCTATTCTTATTACTTCATCT
GTGGAGGTGGTTAGAGGTGGACTAACTGAAGCTAATCGAATCAGAGAGATCTTACTGGAGA
TCATCCACGGAATCTACTAG

>SlbHLH039
ATGGCGGAAACAGAGGAAGACGGATCAAATTGGCTCATAGAATTAGGGTTAATGGAAGACC
TTCCTTCTCTTGAACCCAATGCTCAATGGCCCTCCAACGCTTTTTCCCTTCCAAATAATCTCA
GTTCTGGATTAGAAGATTCCTATGGCAACTCAGATAGTTTGAAGGAATGCGGCTCCAAAAA
GAGGGTGAGATCTGGAGCATGTGCATCTGATTCAAAAGCACATAGGGAGAAAATGCGCAG
GGACAAGCTGAATGACAGGTTTCAAGAATTAAGTTCTATTCTGGAACCAGGAAAGCAGCCA
AAAATGGATAAATCTGTTATCCTAGGTGATGCGGTTCGTATGGTGGTGCAGTTGAGAGATGA
AGCTCAGAAGCTGAAAGAGTCGAACAACAACTTGCAGGAGAAGGTTATTGAATTGAAGGC
TGAGAAAAATGAACTCCGAGATGAGAAACAAAAGCTAAAAGCAGAGAAAGACAAACTCG
AGCAGCATCTGAAGGCCATGAACACTCAACCTGGATTTCTACCACACCCTCCTGCAATGCC
TTCCCCTTTCTCAGCCCCACATCAAGTCTTTGCAAGCAAAATGATGCCATATATTGGCTACCC
TGGAATCCCTATGTGGCAGTTTGTGCCTCCTGCTGCAGTTGATACCTCAGAAGATCATTCTCT
CCGTCCTCCAGTTGCTTAA

>SlbHLH040
ATGGATTCTACGAATCTTTATAATCATCATCAACTTCAACAGCTTGCTGGATATCCCTTTTTCA
GTACTGGAGTTTCAACTCTACATGATTGGAACTCAGGCATCACCTCGGAAGAAGAATATTAC
TACAAGCTGGGACACATGAAAAGGATCTCATCAGAAGAGCTCATGTGGAAAAGAGGCATC
GACACATTTCCATTAATGAACACCTCAATGTTCCACGACGGACATCATGAATCATCCAACGA
TCCGCTGGATGACAAAAATAACAAGGCCGGCTACATAATATCCAATGATTATTTCCTCAAAAT
GAAAGATATGAATAGTTTGAGTAACAACATGTTCAAAGAAAGTTACTTTGAAAACGAACAA
CAACATGCTTTTGATCTGAATGAGAATCTTCTGTCCGAAGATTCTTACATGAACAATGCTAAT
AATTCGAGTTATGTCTCAAGTCATGATATGGAATATTCAGATTTCCAGGGGTTGAAGTTAGCA
TTTAATGGACTAACTTTCAAGAACAGCCATGATAGTAATAGTAATTGCTTTGGCCATTTCACT
ACAGAAAGGATGTCTTCGGGTTTTGCTGATGGACTACAAGAATTAACCCATAGTCCATCATC
CAAAAAAATCACATCAAACGTTAGGAAGAACATTGGAGTTTCTTCAAAAGCAAAAAGATCT
GCTGAAGATGAAGCTAATCCATGTCAAGAAGCATCAAAGAAGTCTCGAGTTACATCGCAAT
CTCCAAGCACATTAATGCTAAAGGTGAGAAAGGAAAAACTAGGAGACAGGATTTCAGCTCT
ACACAGATTAGTGGCACCTTTTGGCAAGACTGATACTGCATCAGTATTAACAGAAGCCATTG
GCTATATTCAGTTCCTTCAGGACCAAATACTGACATTGAGTATGCCTTATTCGAAATCAACTG
AAAGGAAGCTCCATCACATAAATCTAAAGGATTCGAGCATAGAGGCAGTGCTAGATCTAGA
GAGTAGAGGATTATGTTTGGTGCCAACATCATTTTCTTCTTACATCTCTCAGTCCTGTGATTG
A

>SlbHLH041
ATGTCGAGCAGAAGGTCGAGGCAATCATCAACAGGATCCTCGAGAATTTCAGATGATCAGA
TAATTGAACTTGTTTCAAAATTGCAACAACTTCTACCGGAGATTCGCAATCGTCGCTCTAGC
AAGGCATCGGCATCGAAAGTACTTCAAGAAACATGCAACTACATAAGAAATTTGAATAGAC
AAGTGGATGATCTTAGTGATCGACTTTCTCAGTTACTCTCAACTATTGATGCTGATAGTCCAG
AAGCAGCAATCATCAGGAGTTTATTAATGTAG

>SlbHLH042
ATGGGTTCTCGTTCAAAATTAAGCCCCAGTAGCTTATCGATGAGAAGAACAAGAAGATCTAC
CCCTCACAAAAAAACATGCAAATCTCAAAATCTTGTTGACGTTGACGGCGGTTCCGTTTCT
GAAAAATTGGAGGCTCTGAAGCAGTTAATCCCAGCCAACTATGGTGAAATCAAAGCGGACC
AGTTGTTCAAAGAAACTGCTGATTACATTGTTCTTCTCAGAACTCAGGTCTTCGTTTTACAG
AAACTCGTCGATTTTTATGGATCAAATACCGATCAAAATCCTGTATAG

>SlbHLH043
ATGGATTTACAAAATATTGCTCACTCACTAGAACTTGGATTCTCCAATAGCAATATTGAAATG
ATACCTTTACAATCACCACTCCATAACTCCAATTACTTAATGAATTCTCCTCCATCTAATTTCT
CATTCATGGGCAATCCAATTGAAGAGCCAGCCGCGATGCCAATACTCTCATCGATCGATGAA
ATTATAGCATCGACTCATGGTAATGGAAATGATTACTCATGTTTGCAGAGGAGGAATTCTATG
GAGGCTATGAGGGAGATGATTTTTCGTATCGCGATGATGCAACCAATTCACATTGATCCAGA
ATCAGTTAAACCTCCCAAGAGGAAAAATGTTAAGATATCGAAGGATCCACAGAGTGTGGCA
GCCAGGCATCGGAGGGAACGAATAAGCGAAAGGATAAGGATTTTGCAAAGACTAGTCCCT
GGAGGAACTAAAATGGATACTGCTTCAATGTTAGATGAGGCTGTCCATTATGTTAAGTTTTA

AAGAAGCAACTTCAATCACTAGAGCAAGCCGCGGTTAATAATAGGCCGATGATTTCTGGATT
TTCAACAGCAATGTCATCACCAGGGGGGCCAATGAATTATAATTCAAGCTCTATTAGGGCAT
GCCAGCCTCATCAATCCATGAACTCTGGTGCACAAATGCTTAGTTAA

>SlbHLH044
ATGTCTATGGCACTAGCAAAAGAACATGTTATTATGAGTGATACAAAAATGGGCATGGTTGA
TAATTATGATCAATATTATGAAGGTGAATTTGGGATCAATGATCATTCATCCCCAGAGTTATAT
GGGATCCATGAAGAACCTCCAAAGTCTATTTTTGAAGAATGTGAAAATTCAGAAAAAAACAA
GTCCAAAAATAGCCAAGAATTTTGCTTTAAGCAGCTCTAATTCTTCCCTTTCTAGCCCTAGTA
GTTCCAATTCCAATGCTCAATCTGTTATTAACTTCAAAGGGGTTTATGGTAATTTTATGCATTC
AGCAAATGGATCTTTGTTGAGTTTTGAACAAAGTGAAAGATTTTGCCCAAATCCAAGAATG
ATTAGTAATATTAATCAAGTTGAAGGATCTGTTTGGGAAGATAATAATTTGCATTATCAGAATT
GTGTTACTCCTAAGGGAAGTAGTAATACTAGTCCTAGAGTGATTAATGACAATTCCAATAATA
ATGGAATACCATTTGGATGGTTAAATTCTGAAGCTAATGCAAGCACCACTACTCATATTGATG
AATCACGTTTCAATAAGCGCCCATCCACGGAAGAGAGCATGCAAACAAATAAGAAGCAATG
TAGTGCGGGTTCAAAAAAGGGGAAACCAAATAATAATAATAATAATTCAATTGGTACAAAGG
ATCCACAGAGTATTGCAGCCAAGAATCGTCGAGAACGGATTAGTGAGCGTCTTAAGATACT
ACAAGAACTTGTTCCAAATGGTTCCAAGGTTGATTTGGTCACCATGTTAGAGAAAGCAATC
GGTTACGTCAAATTTCTTCAATTGCAAGTGAAGGTGTTGGCAACAGATGAATTTTGGCCTAC
ACAAGGTGGAAAAGCACCAGACATTTCACAAGTAAAAGAGGCAATTGATGCTATTCTTGCT
ACTCAACGAGACAGAAACTGA

>SlbHLH045
ATGGAGCTCACTCAAGAAGATTTTTTAGAAGAAATAGTTTCTCCAAGGATAGAAAATTGGA
ACAACACTTTTGCAAATGCATGGAACATTGAATCACCAACTTTTTATCAACAAAATCCTGAA
TTTATACCTTCAAATTCTTCCCTTTTAGACCTCATAATGTCTCCTTCACAATCCAATTATTTTC
CATGTCCTGATTTTCAAGAATCATCATACCCTTTTCTTCATTCTTTCACTACTACTACCCCACC
TCAACTAGTTATTGATTCTACCACTTATAATAATAATAATATTCAAGAAAGAGCCATTATAGAG
GAAGGGCAAATTGGTCATTTTTCTACTGATTTTCATGGGCATTATGAAGACTCATTTAGTTGT
TACAATATTAACAAAGTGGTCAAAATGGAAGAAGCAACTTCAAGAATTGTGGGAGAAAAA
AAGAGTAAAAATTATAAAGTGAAGAAAGTAGAAGGACAGCCTTCTAAGAATCTTATGGCAG
AAAGGAGAAGAAGGAAAAGACTTAATGACAGACTCTCTATGCTTAGATCTATTGTACCCAA
AATAAGCAAGATGGATAGAACATCTATACTTGGAGATACAATTGATTATGTGAAGGAGCTATT
AGATAAAATCAACAGGTTGCATGAAGAAAACGAAATCAAGGACATTAAATTTCTGGGTAAT
TTCAAGGGGTTAAAGACTAATGAAGCACTTGTAAGAAATCCTCCAAAGTTTGACGTAGAAA
GGAGAAATGAGGATGAAACGAGCATAGAGATATGTTGTGGAACGAAGCCAGGTTTACTGTT
ATCAACAGTACACACAATGGAAGCATTAGGGCTTGAGGTTCAACAATGTGTTGTCAGCTGT
TTCAGTGACTTCTCAATGAGAGCTTCTTGCTCTGAGTCAGTGGATCATCGAACAATTTTAAG
TTCTGAAGACGTGAAGCAAGCTCTGTTCAAAACTGCAGGTTATGGAGGAAGATGTGTATGA

>SlbHLH046
ATGGACAATTCATCGCTAAGTCAATGGTTTTCAAGAACGGAAGAAGGTGTGTTCTACTCAA
ATAGAAATAATTCCATCGACGATTTTACCACTCAAAAATCCACAATCTCCGAAGATCAAGAG
ACTAGTGAATTGTCTATTACTCCAGACAGTGCTCGTTCTAATTCGCGATCTTATTTCCATAAA

GAAATTAGTAAATGTAAAGCCTCGAAAATTAATTTTTCCCTCATTACCCCTATGGAAAATTTC
CCAATTGGTTCAAAGAGACCTGCAACTGATCATCCTCAGCAGCCCAAACTGAATAAGATCT
CTTCATCTTCGCATCGTTTTCTTTCTTTTAATAATAACAATAATAATACGGATTCAGTATTTCCT
AGTCATCAAGGATGTAAAATCGAAGCTATGGATGATATGTATTTGTGTGATTCATCAGATCAG
ATGTTTTATCCTTATGTGAAATCGAACGGTTGTTACGATGCGAAGGATAAAAAAGGGGGAAA
AAAAATTCCTTCAGTAGAATTACAAGATCATATTATTGCTGAGAGAAAGCGCAGAGAGAAG
CTCAGCCAGAGGTTTGTGGCACTTTCAACCATTCTTCCAGGGCTAAAAAAGGTGGACAAAG
CTTCAATTCTTGAGCAAGCAATTAAACATGTGAAAGATCTTAAAGAAAAAGTGCAACTATT
GGAAGAAGAGAAAAAATCAGTTATGTTTGTGAATAAATATAAGGTGGAAACGGAAGAGTAC
ACTTCATCTGAAGAAAACAATAGTGGCAGCGATTTACCTGCAGATATTGAAGTAAGATTCTC
AGATAATAATGTATTGATCAGAATTACCTGTGCGCGGCGCAACGCATTTGTGCTGAATATCCA
TAGCGAAATAGAGAAGCTTCATCTTACCATTGTTCAAAGCTCTATGATGCCATTTGGGAAAC
AAGCAATTGATATAACACTAGTCGCACAGATGGAGGAAAGTTTCTGCATGACACTAAAGGA
TGTTGCGAAGCATGTACGAATGGTCACAGGGAGGTTGATGACCCAAGCTTAG

>SlbHLH047
ATGGATACATGGTGGTCTGAAATGGAGACTAGCAATATGAATGATCACAACTTTGTTAATCA
GAGCCAGGCGATGACACAATTTAATGAATTTCCTTTCAATTCTAAGCCCTTTTCTACTTTTAC
CCCTCCTCCTCCCTCCAATGTAATTTCTAATTACAGCTACTCCAATTCAATGTCAACCAAAAA
TCAATTCGAAAAGTTGAACACCTTTAAAGTCGTTAAGCATGAGGTTCCATCTGGAACTACAA
TAAACTTCTCTTCTTCCGTTAATTCTATGGACGATTCTGATTTTGGTGATATCGAGGCCGCCAT
GGGATTTGGTGCTGCTATTACGACTACTACTGATCAGAAGAAAAGTTACAATAGAACAAGT
GTTCAGGCACAAGATCACGTTCTAGCGGAAAGAAAGCGAAGAGAACGCTTAACTCAGCGT
TTTATAGCTTTATCTACCCTCATTCCAAACCTCAAGAAGTTGGACAAAGCAACAGTACTGGG
AGATGCTATCCAGTATATAAAAGAGCTGGAAGAACAAGTAAAAACCCTTGAGGAAAAAAA
CAAGAAATGTAGTGAGGAACCGGTGATTCCACCAGCAAAGAGACCTCGATTGGTCTCCAGT
TGTGCCGATTCATCGTCGTCCGACGAAATATCCAGTGTTAGTACAGTCTGCACAGATCGATC
ATTGCCAGATATTGAAGTTAGAGCTTCAGATGGAAATATTTTGATAAGGATTTATTGCAAAAA
ACAAAATGGGATGATGAAGGAAATATTCAATGAAGTGGAAAAACTTCATCTTTCTATCATTA
GTTGCAGTGTTATGCCTTTTGGCTACAACACTTCCCACATCACCATTATTGCTCAGATGGATC
ATAAATTAACAAGCAATACACCAAATCATGTTGCAAATAGAATCCGAGCGGCGATGGTGAA
AGAAGAAGCCAACTCATTTACAGCATAG

>SlbHLH048
ATGGATATTTCATCAGCTACATGGTGGTCTGAAATGGATGTTATGAATATGAACGAACTTCAA
TACATTGATCAGACATTTGATGATTTTGCATTCTCGGATAATATTCAAGTTTCTCAAATTGGTG
AAATATTTGAGGAGAAGCCAGCCTTGTGCAGTTCAATTACCCAAAATACTAGTACCTCTCCG
CCTTGTTCGTCTCCCAGTGTTATTTCTTTTTCCAACTCCAATTCGCCATCAGCCACTCCTACA
ACTACTAATGCCCAAAACTACTTTAAAAATTTGAATACATCGTCTCTTAAGACTGAGGTTCC
ATCAGGAACTACTATTAATTTCTCCTCATCAAATACTTCTTCTGATTCTGATTACGATGATAGT
AAGCAACTATTCCAGGCCATGGGATTTGGTGCTGGTCAGTCGAAGAAGATGAATTATAGTAG
AACGCCTTTACAGGCTCAAGACCACGTTTTGGCTGAAAGAAAGAGAAGAGAACGACTCAC
TCAGTATTTCGTAACTCTGTCCACCCTCATCCCTAACCTAAAGAAGTTGGACAAGGCATCAA
TACTTGGAGATGCGATAACATATATAAAACAACTTGAAGAACAAGTAAAAAGGCTAGACGA

AGAAGCTAACAAGCAACCAGTTAAGAGAAGTCGGTTGCACTCAAATTATGATAATTTTTCA
ACTTGTAATGAAAACTCCAACAAATCAGTTGTTCCAGAAATTGACGTTAGAGTTTCAGATG
GAAATGTGTTGATTAGAGTTTGTTGCAAAAAGCAAGCGGGAATAATAAAGGAAATATTCAG
CCAAGTAGAAATGTTTCAACTCACTATTACTAGTAGCAGTGTCATTCCATTTGGTTATGATAC
AACACACATAACTATTGTTGCTCAGATGGATCATCAGTTGAACATGGCAACAGAACAAGTC
GCGAACAACATACGTTTGTCTATAATGAAGCTTATCAATAGCCATAAATAA

>SlbHLH049
ATGGCCAACAACAATGTTTATTATCACAATGCTAATTTTTCTTTGACTGATCCTGATCCTGAA
CCAGATGATATTTCTGTGTTTCTTCGTCATATTCTACTTCCTTCTTCGTCTTCGTCTTCATCTTC
ATCTAATTTTATGGCCCTGAAGAGTAATGAAATGCAATATTCCTCATCATTACCTCATCTTATG
CCTAATAATAATCAACAGGGTAATTTGTCATCCATGATGAATTCATCAGCTTGCGGCATTTTC
TCGTCTTCCTATGGGGTTTGTAATGGTGCAACTACTGTTTCTTCCTCATCTGTTGGGACCATA
GATTACGATCCGGATGAGTATGAATGTGAAAGTGAGGATGGTACAGAGGATTTGGGGGCGG
AAGCTTCAGTTCAACCGCCATCTCGTAACACTTCCAAGAGAAGCAGAGCAGCTGAAGTGC
ACAATTTGTCTGAAAAGAGGAGAAGAAGCAGGATTAATGAGAAGATGAAAGCATTGCAAA
AACTTATTCCAAATTCAAATAAGACTGACAAGGCTTCAATGCTTGATGAAGCCATTGAGTAT
CTTAAGCAGCTTCAGCTCCAAGTACAGATGTTGACAATGAGAAACGGGTTAAACATGTATC
CTCTTGGCCTGCCACGAATGCTACAACAAAATCAACTCTCCCATCAAAAAGTAGGTTTGTG
CGAGGGGAATGCATTCACAAATGCCAAAGTGGCTGGGAATCTACAAGTTAACCAAGACGCC
TCGTTAAATGCTATTTTCAACCCAACTGAAAATTGTACAGAAACTAAGGTAACACCACCGAT
AACTATGTCAAATATAAACCGGTCAGACAGTGCGTTTGAGCTTGAGTCATCGATGAATATTC
ACCTTGATCCCTTTCAGCTCTCAAGATCTACCAGCAAGGAAATTTGGAGGGAGGATGACTT
ACCTTTATATGGTATGAATGAGCTAACGACCAAAACTGCATCAACTGGATCAAATCTGGCAT
TCTCAGTTCCCCTAGATACGGATGCATCTAACCTGAAGAGAAGCACTCGGGAGGCCTGCTT
GCTTCGATATCAGTTTGGTGCTGTGAATGAAACCAATCTGGATTGTGACCAACTTCTTTCCC
AACAACTGTATAGCAACTTCTGA

>SlbHLH050
ATGGCGGACAACCCACCGGAGGTATATGCTGCCGATGATTTTCTCGAGCAAATTCTGGCGAT
TCCCTCTTATGCTAGCCTGCCGGTAACTGATTTAACCGCCGGTGCCTCATCGGAGAATTCAA
CATCTGGTGTCTCTCAGCTCCAGCAGCAGCCGTTGTTCCCATTGGGGTTAAGTTTGGATAAT
GGCTTTGCTGACGCCAACAACACTGGAGGTTTTCAAGTGAAGACTGAAAGAGAAGCAATG
AACATGGGGAATTTGTATCCAGGTCTAGAACATTTGCAATCTCATGCTGTTTGCCTAAGTGTT
CCTCAAGTTCACCAAGTCCAGCCTTTTCAAGGCCACCCTACATCAAGCGCAATAGTAACAAT
ACCACACCAACCTGCAATTCGTCCTAGGGTTCGGGCACGAAGAGGACAGGCCACTGATCC
ACATAGTATTGCTGAGCGGTTAAGGAGAGAAAGGATATCAGAACGAATAAAGGCTTTACAG
GAACTTGCCCCCAGCTGCAATAAGACCGACAGGGCAGCAATGCTTGATGAGATTCTGGACT
ATGTGAAGTTCTTAAGGCTTCAAGTTAAGGTACTGAGCATGAGTAGGTTGGGAGGAACTAG
CGCGGCGGCACAAGTTGTTGCTGATATTCCATTGCAGTCTGTTGAGGGAGACACCTGTGAA
AGTCATTCCAACCAGCGCGTCTGGGAGAAGTGGTCTGATTCTGAAACAGAGCAAGAGGTA
GCAAAGCTCATGGAGGAAGATGTTGGAACAGCCATGCAGTACCTTCAATCCAAATCACTCT
GCATCATGCCTATTTCACTTGCTGCACTTATCTATCCGACTCAGCAAAGTGACAACCAATCAA
TGGTCAAGCCAGAACAAGCGGCCCCATTGTAG

>SlbHLH051
ATGTTTTCTTTACAAGGAAGTGATGACTTATTAATTCAAATTCTCTCAAATACATGTCAACAG
AACAAGAGTTCACTAGATGCAGTAATGGATTTTGCTTCTACGGATACAATTACGCGAACTCA
TATTCCTCCGAATATTAATTCACAGAAGAAGATAATATCGAAAAGAAAACTGAGTACTCATA
GTAATAAAAACGATGGAATTGCTTCAGATGATCATTTCAAGCTTAAAAAAATTATACATCGA
GATATTGAACGTCAAAGAAGACAAGAAATGGCTGCGCTCTATTCTTCTCTTCGTTCTCTCCT
TCCTCTACAATATGTCAAGGGAAAGCGTTCAGTATCTGATCATATGCATGAAGCTGCGAATTA
CATAAAAGAAATGCAAGGAAACATAAAGGAATTAGAGAAACGGAGAGACTTGCTGATGAA
GTCAATTCGACATGGAAATGAAAATGCTGACAAAAATAACAGATTCACAGATTGTACTGTC
ACAGTTAGCCCCTGGCTGCAGGAAGGTATAGAGATCTCAATCAGCGTCGATTGTGAAGGGA
AAACTTTTCCTCTTTCAAAAATTCTGGGTGAGCTTTTGAAACAAGGGCTTAATGTGGTGAGT
TGTGTTTCTGCAAAAGCGGATCAAAGGTCACTCTACTCAATTCACACTGAGGCGTGTGATAT
GAACAACATTGATCACTTGGCATTGCAACAAAAAGTGATTGATATGATCAACCTGGACTTAT
AG

>SlbHLH052
TATTCCCTTTTCTACATCTCCTCTTTTATTTTATTTTTCCTCTCTAAAAATTTCCTTTCATACGC
TCACTCACATATTATTACATTACCTCTACTTTTTTCAACGTTGAAAATGGATGGTGACCAAAA
TTTATCTGATTTATTCGACGATTCCGAATGCGATATCTTCGGTATTTTAGAGGCTTTAGAAGGC
GGTGGAGGTGGAGGTGGTAATAGTGGTATTACTTCGAAATTTAATGATAATATCAACAACCA
GACTGCAACAATCGCAACCACAATCACAACCACAACGTCTGATGAAATTACGGGTTTGGTG
TCAGAAGAAGGAAAGAAGAGGAAGTTAATATCTCAAAAGTCCACGGGTTCATGCGCGACT
TTACAAGAAGAGGAAACTATAGAAAATAAGATTTCTCATATAACAGTTGAAAGGAATCGCC
GCAAACAAATGAATGAACATCTCTCCGTGCTACGCACTTTGATGCCTTGTTTTTATGCTAAA
CGAGGTGATCAAGCATCAATAATTGGTGGGGTTGTTGATTACATAAACGAGCTACAACAAGT
TCTCCAATCCTTAGAAGCCAAAAAGCAACGCAAAGTTTATAGTGAAGTTTTAAGTCCAAGA
GTACTCCCACCACAACTAGTCCCAATTAGTCCAAGACTATTAACACCTTCACCATTAAGTCC
AAGAAAACCACCACTTAGCCCTAGAATGAACTTACCAATTAGCCCAAGAACCCCACAACCC
ACAAGCCCATATAAACCTAATGCTAATGCTAACAAGCCACCTGAACCATCTCCTACTACTTCA
TCTAATTCTTCCATTGATAGTCATGTCAATAATGAGCTTGCTGCTAATTCAAAGTCAGCCATT
GCTGACGTTGAGGTGAAATTCTCCGGCGCCGGCGCCAATGTCATATTGAAGACCGTCTCTC
CACGTATTCCTGGCCAGGCTGTCAAGATTATTGCTGCTTTAGAACAACTCGCACTTGAAATA
CTCCATGTTAGCATTAGTACAATTGATGGTACCATGCTCAACTCCTTCACCATTAAGATTGGA
ATTGAATGCCAACTAAGTGCTGAAGAACTGGCTCATCAGATTCAGCAGACATTCTGCTAA

>SlbHLH053
ATGGCTGATGAATATTTTCAAGATGGAGTCTATGGAGAAAGCTTGTGGATTAATTCAACAAA
AAATATTTTTAGTTTATCATCATCAACTAATTCAATTCTTGATCCAATTATTGGACGATATAATT
TTGCATGGCCAAATGATGATCAATTTTTGGACATGAAAAATATGTCAAATAATAATGATGATT
ATTATTCTAGTTCTGATGATTCTATAGTTTTTCAAGAATTTCCCAAAAATCATAACTTGGGGAT
TAATGAAAATTCTTCTTCACCAGATTGGAATCACTCAATCAATGATTCCATGTTACAAGAAA
AACTCAACTCAAGAGAGAATTATCCTAATCATAATGAATTGAAGAGGAACTTTTCCAGCATA
ATTAGTGAAGATAATTCCTTAATTAAACCAATTATGAATCAAGATTTCACTAATTACTATTCAG

ATTTGCTTCAAACATTGTTTGCTAATACTGATCTTCATGAAGAGCAACCTCAACAACAATCTT
TCAATTATCCATCATCATCAATAAATCATAGACAAAAATTGAAGGATTTTGTACCTTCTTTGC
CTAAGTTTGATATTGAAGAAATTGGAGAGTCTAAATTAAGCTCAATTACCAAGAATAACACA
AATGAACAAACAAACAAACGGCAGAGAATTGAGACACCATCATCATTGCCAACTTTTAAGG
TGAGAAAAGAGAAACTGGGTGACAGGATAACTGCCCTCCAGCAATTGGTTTCACCTTTTGG
AAAGACTGATACAGCTTCAGTTCTCCAAGAAGCAATCGAATACATCAATTTTCTTCATGATC
AAGTCAATGTATTTAGCAATCGATATATGAAAAATGGACCACCCACACAACATCAACAGGTT
AAGGAATTACAAGAAGGATTAAAACAAGGGTTAAGGAGCAAAGGACTATGTTTAGTACCAA
TATCAAGTACATTCCCATTAGCAGCTGAGACTACTATGGAATTTTGGACACCAACATTAATGG
GAACTACAGTCAGATAG

>SlbHLH054
ATGAGCATTCTTGAAAGACAAAGAGCTATTTTTGAACATTTGTACCAATGCCAACAACAAC
AACAAACTTCTAATTCTTTGCCAAATCAAAATCTTGCTCAACTCAATAGTTTAATGAGTGAA
AATACAATGGAATTCAGTCAATTTCAGAATTTTCCCTTCAAGATTGACACACAAATTGATTTT
GGAAGTGAAAAGAGGAATTGGACAAGCAAGAAGAGAAAATCAGAGGTTTATGAAGAATAT
GAATGTAAAGTTGAAAGACTTGATGGAGAAGCAGGGGAAGTAAAGACAGAGATGATCGTG
AAAACTGAAAAAGGGAAGAATTCGAAGGAGAATTTAGAGGCTAAAAATACTGATTTTATTC
ATGTTAGGGCTCGTCGCGGCCAAGCTACTGATAGCCACAGCTTAGCTGAGAGAGCAAGAAG
GGAGAAAATAAGCAAGAAGATGAAATGTTTACAAGATTTAGTACCAGGTTGTAACAAAGTG
ATTGGCAAAGCAGGAATGCTTGATGAAATCATCAATTATGTTCAATCTCTTCAAAAACAAGT
GGAGTTTCTTTCCATCAAACTTGCCACTTCCAATGTGAACACAGACAATTTATTTGCCAAGG
AGTTACCTAATCCAACATTTTTACAACAACAAGGAAACATTAATATTGGTGTTACACAAAGA
AGAGAAAATTGTTTGATGTCTTTCCCAGAAGCTGTTCTTGATTCTTCAAATGTTCTGGCATTA
CAACAACTACCAAATCTTGAAACTGATCTACAATGCCTTTTTGGTGTACGGTTTCAGAAGTA
A

>SlbHLH055
ATGGATTTAAATGAGATTACACAATCCATCAACAACATTGAAATGATTCAACTGCTACCAATC
CACAACTCATCATCAATTGATCATGTGATAATGAGGGAGATGATTTTTGGTATAGCGATGATG
CAGCCCATCAAAATTGATCCGGAATCAGTAAAACCGCCTAAGAGAAAGAACGTAAAGATAT
CAAAGGATCCACGGAGCGTTGCAGCCAGACATCGGAGGGAAAAAATAAGCGAAAAGATAA
GAATTCTTCAAAGACTTGTTCCTGGAGGAACCAAAATGGACACAGCTTCTATGTTAGACGA
GGCTGTCCATTATGTTAAGTTTCTTAAGAAACAACTTAAATCGTTAGAACAAGCAACAACTG
CTTGCTCTAACGATAGAAATAATAATAGGTTTGTTAGTGGATTTAACCAATGTCCTCATAATT
GCTACTAA

>SlbHLH056
ATGGAGAATTCTTTTAGTTCAGAATATTGTGATGTGAATGATTTTTTAGTTCAAAATTACTTAC
CTCAATGCTCTCACGAGGGAAGAGAAAGTGCTTCTAGGAGTCACAGTGAAGCTGAAAAAA
GACGCAGAGACAGAATTAATGCACAGCTTTCTACTCTCAGAAAACTCATTCCTACTTGGGA
AAAGATGGATAAAGCAGCTCTACTAGGAAGTGTAGTTGATCATGTTAAAGATCTGAAAGAC
AAAACAGCAGAAATCAGCAACGTTTTAAACACTCCAACTGATACCGATGAAGTAAGCATTG
AGCATTTAAATGAAGAAGAAGATAACAAAGGATGCCTTATTAAGGCCTCTTTTTGTTGCGAT

GATCGACCTGAATTATTCTCAGAGTTGCAACGGGGAATTAAGAATCTGCAGTTAAGAATGAT
GGAGGCTGATATAACTAGTTTGGGTGGCAGAATTAAATGTGTTTTTATGCTCTCTCCAAATGA
CAATTATGTTTGCATCAATTCTCTCGAAAAATCACTAAAAGCTGTCCTCTCTAGAATTGCTAT
ATCTCCTTCCACATCAAATTACAGAATTAAAAGCAAGAGGCAGAGGTTCTTCTTGCCTCCTC
AATTTTCTTAA

>SlbHLH057
ATGGCTGCTTTTTCATCACACCAATTACAACACAATAACCCATTTCTTCTTGATTCAGTATTTT
TGCCAACTTCTCCTATTAAAATGTCTGGCTTTTTTGAGGAACCAAACAATTCTTGTATAGTAC
AACAGTTTTACCAACAAGAATTCCCTTCCAATTTAATTTCTCATGAAAATAGCTTTTGCCTTG
ACCCTAAAAGTAGCAGCAGTATAAGCTTAGATATGGATGCTTCCTCTGTTACTGATAAAATTG
AAAGTGGAATTAATAATAATAAGGCTAATGTTAGTCCTTTGGATAAGAAAAGAAAATCTAGT
GAAGGGTCTTCTTCTATGACTTCTGCTCATTCTAAGAATGAGAAACAGGGTGATAATGGGAA
AAAGAAGAAAATTATCAGCAAGTTAGTAGCCAAAGATGAAAAGAAAGCTAATGAAGAAGC
ACCAACAGGGTACATTCATGTTAGAGCAAGAAGGGGCCAGGCAACTGATAGCCATAGTCTT
GCTGAAAGGGTGAGGAGAGAGAAAATAAGTGAAAGGATGAAGATATTGCAATCTCTTGTTC
CTGGTTGTGACAAGGTAACTGGAAAGGCCCTTATGTTGGATGAGATAATTAATTATGTCCAA
TCTTTGCAAAACCAAGTTGAGTTTCTCTCAATGAAACTTACTTCTTTGAATCCAATGTACTAT
GACTTTGGAATGGACTTAGATGCACTCATGGTCAGACCTGATGACCAGAGTTTAAGTGGCTT
GGAGACACAAATGGCAAATATTCAGCAAGGTAGCACAACTACTACATCACAGGCAGCTGAA
GTTATTGCTAACACTAATAGTGGCTACCAATTTTTGGATAATTCAACATCACTCATGTTTCAA
CAATCCCATTTCCCTAATTCTATTCCTCAGGGTATTGGACAGCTCTTATGGGGTGCAGATGAG
CAAACACAAAAAATAATAAATCAGTCTGGATTTAGCAACAACTTTTGTTCTTTCCATTAA

>SlbHLH058
ATGAAAATTCTGCAAGATTTGGTTCCTGGATGTAATAAGGTTATCGGAAAAGCACTTGTTCT
TGACGAAATAATAAATTATATCCAGTCACTACAGCGTCAAGTTGAGTTCTTGTCCATGAAGC
TTGAAGCAGTCAATTCAAGGATGAACCACCCTATAGAAACCTTTCCTTCAAAAGATCTAGC
ACCATCAGCTTTTGATACGAGTGGAATGATTTTCGGCACTCAAGCACCGAGAGAATATGCTC
AAGGGACACAATCCGAGTGGCTCCATATGCAGGTTGGCAACAGCTTTGACAGAGCAACATG
A

>SlbHLH059
ATGGAAAACACTAGTCCAGAAAAGCTAGACAGGAAAACACAAGAAAAGAACAGAAGAAT
TCAAATGAAGTATCTTTCTTCTAAGCTCTTTTCTCTTATTCCTCCACACCACCACCAATACTC
AGCTAAGGATATGGTGACGCAACAAGACCAAATTGATCAAGCCATTACTTACATTGAAAAAT
TGAAAGAAAGAGTAGATGTATTAATGAGAAGGAAGGATAAGATTATAGCACAAGGTACAAG
TGATGATTCAAAGAAATTCATGCCTTCAACATCTTGTAGCAATATTAAATTACCTATGATTGA
AGTTAGAGAGTTGGGTTCAACTATAGAGGTTATTTAGTTAGTTGCTTGCAAAAAAAGTTCA
CCATGCAAGAGGTGATAATCATCTTAGAGGAAGAAGGAGTTCAAGTTGTTACCGCTAATTTT
TCAACAATCGGCGATAAGGTTTACTATACTATTCATGCTCAGGTGAAAATTACGAGATTAGG
GGTTGATGCATCAAGGGTCTATTTGAGATTGCAAAATCTGATTTGCTAA

>SlbHLH060

ATGGAAAACAACAGTGTTAATAATATTGTTAGCACTAGCTCAGTACAAAAGCTAGACAGGA
AAACACAGGAAAAGAATAGAAGAATCCAAATGAAGTATCTTTATGCTAAGCTCTTCTCTCTT
ATTCCTCTCAATCATTCTAAGGAGGTGCTGACACAACATGACCATGTTGATCAAGCCACTAC
TTTCATTGAGGAATTAAAAGAAAGAGTAGAGGTATTAAAAAGAAGGAAGGATGAGGTGGTT
GCACAAATCATAGGTGATGATTCAAAGAAATCCATATCTACAACAACTTGTACAATAAAAGT
TAGAGAACTGGATTCAACTCTAGAGGTCATTTTAACAAGTGGTTTGCAAAAGAATTTCACAT
TGCAAGAGGTTATTAAAATCATAGAGGAAGAAGGTGCACAAGTTGTTACCGCTAATTATTCA
ACAATTGACGGTACAATTTATTATACGATCCACGCTCAGGTAAAAATTACAAGATTAGGGATT
GATGCATCAAGGATCCATTTTAGATTACAAAAGCTGGTTAGCTAA

>SlbHLH061
ATAACGGAAATGGAAAGCAGCAATATTAATATTGTTACAGCTTCAGAAAAGATAGAGAGGA
AAACACAAGAAAAGATTAGAAGAATCCAAATGAAGTATCTTACTTCTAAACTTTTTTCTCTC
ATTCCTCCCCACCATCACCAATCTACTAAGGAGGTTTTGACAAAGCAAGACAAAATTGATGC
TGCAATTACTTACATTAAGCAATTAAAAGAAAGAATAGAAGTATTAGAGAGAAGGAAGGAA
GAAGTGGTTGCACAAGAGACATGTGATGATTCAAAGAAATCCATGCCTACAACAACTTGTA
GTATTAAATCACCAATGGTTGAAGTTAAAGAGTTGGATTCAACTCTAGAGGTAATTTTAGTG
AGTGGTTTGCAAAAGAACTTCATATTGCAAGAGGTTATAAAAATCATAGAGCAAGAAGGAG
CACAAGTTGTTTCTGCTAATTATTCAACAATTGACGATACAATTTATTATATGATCCACGCTCA
GGTGAAAATTGCTAGATTAGGGATTGATGCATCAAGGGTCCATTTGAGATTACAAAAGATGG
TTTGCTAA

>SlbHLH062
ATGGAGCTGCCTCAGCCCAGACCTTTTGGAACAGAAGGGAGGAAGACGACTCATGACTTT
CTTTCACTGTATTCACCTGTTCAACAAGATCCAAGACCTCCCCAAGGTGGCTACCTGAAGA
CTCATGACTTCTTGCAACCACTGGAGCAAGCAGAGAAGACATTAAGGGAAGAAGAAACCA
ATGTTGAAGTAGCAACTGTGGAGAAGCCTCCGCCACCAGTAGCTGCTACTCCTTCAGGGGA
GCATATTCTTCCTGGTGGCATAGGCACCTTCAGTATTTCTTATTTGCACCAAAGGATACCAAA
ACCAGAGGCAAGCTTGTTCTCTGTAGCACAAGCAAGTAGTACCGACAGAAATGATGAAAAT
TCAAACTGTAGTTCTTTCACAGGGAGTGGTTTCACACTGTGGGATGAATCTGCAGTCAAGA
AGGGAAAGACAGGGAAGGAGAATTCTGGGGGTGATCGACACGTCCTAAGAGAAGGAGGT
GTGAACACTGGAGGTGTTCAGCCAACGACATCATTAGAGTGGCAGTCACAATCATCTTCAA
ATCATAAGCACAACACTACGGCCTTAAGTTCGCTCTCATCTGCTCATCAATCATCGCCCCTTA
AGAGCCAAAGTTTCTTGCATATGATAACATCAGCAAAGAGTGCACAAGATGACGATGATGA
TGATGAAGATTTTGTGATTAAGAAAGAACCACAGTCGCACCTTAGAGGTAGTTTATCTGTAA
AGGTTGATGGAAAAGGCAACGATCAAAAGCCAAGCACCCCACGTTCAAAACATTCAGCAA
CAGAGCAACGGAGAAGAAGCAAGATCAATGACAGATTTCAGATGTTGAGGGGAATCATCC
CTAATAGTGATCAGAAAAGAGATAAAGCGTCTTTCTTACTGGAGGTTATTGAATATATTCATT
TTCTACAAGAGAAAGTGCACAAGTATGAGGAATCATACCAAGGCTGGGACAATGAACCTCC
GAAGTTGCCATTGAGCAAGTGCCATAGGACGACTCATGGCGTTAGCAATCTTCCTCAACGC
ATTATCAATGCATCCAGTGCTTCACTGACGTATGCTGGAAAGTTTGATGAGAGCATAATGGG
GATTTCTTCTGCTAATCCTATCAATGTGCAAAAACTGGAACCAAACATAAGCAGCACAGGTT
TAAAAGATAAAGGTCAGCAGCCTGGTTTAACAAATAAGCCGACAACAGTTCCTATGCACCC
AAACACGTTTTCATTTTCTGGGACTAGTAGCACGGCAGCGCTTTATTCATCAAAGCTGATAG

CTGATACTGATAAGTTGGAGTCAAAGTCTCATTCTCAATTTTCACTTAGCAGATCGCATATGA
CTGATTATGCTATTCCAAATGCTAATCCAGAAAGGCTGGAGCTGCCTATAGAAAGTGGTACC
ATCAGCATCTCTAGTGCCTATTCTCAAGGGCTATTGAACACCTTGACACAAGCGCTGCATAG
TTCTGGAGTAGATTTGACCCAAGCCAATATCTCTGTGCAAATTGATCTTGGAAAGAGAGCAA
ATGGGAGAGTAAATTCATCAGCTTCCACTGTTAAGGGTGACAACGTTTCAACAAGCAATCA
ACCAATTCCAAAATCTAGAGTTACAACCACAAGGGAGGAGCCTGATCACGCCTTCAAAAGG
CGGAAAACAAGCTAA

>SlbHLH063
ATGAGTCATTGTACGGTACCAACTTGGAATCCAAGTTATCAAAGACAAGAACATGTCGTAG
AAGCCGAAGAAGCTAACAAATATCCTCACCTACATAACCAGCAGATTCAAATTAATCATCTT
TTGCCCATGTCTAGTAAGTGTGAAGAAGTTGCAGAGCTGACATGGGAAAAAGGGCAGTTA
GGAATGCATGGACTAGGGGGGATCCTTTCCATTTCACAAGCAAAACAGACACTGGGAAGA
ACCGGCGATACATTAGAGTCCATCGTGCATCAAGCCACATATCACGCGAAGAATCAGACTTC
AATACATCAAAATTATGCCCAAAATGAAGATCAGGATCTGAAAACTGGGGTGCTGTATAGCG
GAGGAAAATGGGGCGAGAGTTCACAACAAATGGCGCCTCCTCGAGCAACAGTGTTGGCAA
AAAAGAGGATGAGACCATCAGAATCTGACCCTCAATATGGTGGAGCTGAAGATCATGAGTA
TGCAGAAGGTAGCGCGTGTGCAAGTGCCAGCGCTACCTTCTATAGGGAGAATGATACCACC
ATGGTTACATGGCCTTCCTTTGACGAGTCTTCGCGTAGCATCAAGTCTAAAACCGCTTGTGA
TGAGGATTCTGCTTGTCATGGTGGTGGCTCGGAAAACAAGGAGGAAGAACATGAAACAAA
AAGGTCCAACTCATCAAGACGTAGCCGAGCAGCAGCTGTTCATAACCAGTCAGAGCGGAG
ACGTCGAGATAGAATTAACCAAAGGATGAAAGCTCTACAGAGATTGGTACCAAATGCAAGT
AAGACAGATAAAGCATCAATGCTTGATGAAGTGATAGATTACTTAAAGCAGCTACAAGCAC
AAGTTCAATTGATGAGTAGTACTCCAAGAAACATGGCACCACAAATGATGATGCCTCTTGG
AATGCATCAACATATTCAAATGTCGTTACTAGCAAGAATGGGCGTTGGCGTTGGTCTTGGCA
TGGGTATGGGAATGTTTGACATGACTGCTTTAGCTCGCGCTGCTGCTGCCTCTGCCACAACT
CATCCAAATCAAATGACTACAGCTCCCATCAATATTCCATTCACCCCATCAGGAGCCTTCGC
GCTGCCTGCAGCCCCGGCTAATTCTGTTAGCCCTGCCAGTGCCACCACTAGTACTACCACCA
ATTCCATTCCTTTTACGAACCCCTATAGTGCTTTTCTACCTCAATCGATGGACATGGAGTTCT
TCAACAACATGGCAGCTTTGTATCGGCAGCAATTAGCTAATCAATCTACACAGATCACAGGC
AGCAAGTTGAACCAGGAAAACCAGACAGAATAG

>SlbHLH064
ATGGATACAGAAAATCCTGCTCCAATAGTTGAAAAGGATACTACAGATGCGGAGACATCATT
GGATAGCTCTCATCTCAGGAAGAAGATTCAGAAGAAAGTTCCAAAAAGAATTCACAAAGC
TGAGAGAGAGAAAATGAAGAGAGAGCATTTAAATGAGCTTTTTCTTGGTTTGGCTGATGCT
CTTGAACTATCTGAGCAGATGAATGGAAAAGCCTCTGTATTGAGTGAAGCTGCTCGATTTGT
AAAGGACATGCTTTCTCAGATCAAGCATATGAGGACAGAAAATACGACTTTGCTGTCTGAAT
CTCAATATCTTAGCGTGGAGAAAAAGGAGCTTGAGGATGAAAATACAGTTCTGGAGGCTGA
AATTAGCAAACTGCAGAATGAGGTCAAAGCAAGGGAAGCTGAGACTAGTCTTGACCTAAA
TCTAGCTCCTCCTGAAATTCATCACACAGAGTTTGCCTCACAAACTAACTATATGAGATTGC
CTGCTTCGGAGCACGGATTTCAACAGTCACAGATGATGAACCCCGTGTACGTCTTTCCCTTG
AGTTCTAATCCTCAGGCTTATCCAGCGCCTGATGCTGCAGATCCCATGGCTATGCCCTCATCT
ACTGTGAAGAAACCACAGCCTAGATACCCCACTCCAAATGATGTATGGCCATCCCAAATCCT

TGAAAAGAGGCCTCAGTTATTGCGACAGGAGGTTCAAGATGGTGCGTAA

>SlbHLH065
ATGATAACTGGAGTGAATGATATGGGTTGGATTGATGGTAAGGAGGATGGAGGAACTGGTT
CTTGGGTTAATCAGAACAATGAAAATCATCAACAGAACAATGGTGGTTTTCCCAATGAAAA
TCATCAGCTGAACAATGGTGGTTTTACTAATTTCCAAGGTATGGTTGATGATGGAGGTGTGG
ATTGGTTTATGGGTGGTGGTGATAGTAACAATCATCACATCAACAACAACAACAACAATGGT
GGTGGTGGTGGTGGTGGTAGCAACATGCAGAGCCACATTTCTTACTCTACAAGTTTCACTG
AAGCTGAAAACAGTTTGCTTCTGCAACCTGTTGATTCTTCAGCTTCCTGCTCGCCTGTTTCT
GGTAACGTTTTCAACAACATTGACCCTTCTCAGGTGAACTTCTTCATGCCCCAAAAATCTAC
TATTCCTTCTTCACTCACTGGGTTATCCAACAACCCAATGGATAACAGCTTCAATTTGGGTAT
GTTGAATCAAGCTGGGAACGGGATGATGAATACTGGGTATCATCATCTGGGTTCACCAAACC
AAATGGGGACGAACAACCTGAGTTCTTATACTCAGTTTTCTTCTCCCAATCTGCTCCAGCTA
CCTCAGGTAGCTGGTGGGTACAGTTCGATGGGATTTGGGGCTAACAATTCTGCTAATGGGAA
CACTTTGTTTCTCAATCGGTCTAGAACTCATAAACCTTTAGACAATTTTGCTTCCATTGGAGC
ACAGCCAACTCTTTTCCAGAAAAGGATTGCAAAGAACTTGGTTAGTAATGGTGAAAATTTA
GGTACAGAAATCGGTCAATCTTCCAGTAATCTGACTGATAAAAAAAGGAAGTCCAGCATGA
ATGATGAGTTTGAAGATGTTAGCATGGATGGAACATTGAATTACGACTCGGATGAGTTCATG
GATATCTCTAACAAAATGGAAGATGGGATCAAGATTGGGGACAGCTCTAATGCAGCCAGCA
CTGTCAGTGGCGCTGATCAAAAGGGAAAAAAGAAGGGACCTCCAGCTAAGAACCTGATGG
CGGAGCGACGCCGCAGAAAGAAGCTGAATGACAGGCTTTACATGCTGCGATCGGTTGTCCC
AAAGATAACCAAGATGGACAGGGCTTCAATCTTAGGAGATGCAATTAAGTACTTGAAGGAG
CTTCTGCATGACATCAATGAGCTGCATAATGAGTTGGAGTCCACACCAGCTAACAACTCTTC
ATTGAGTCCCGCAACCAGCTTCCATCCTCTGACACCGACTGCATCAGCTCTGCCAAGTCGTA
TAAAGGAGGAACTTGTTCCAAGCCCCCTGTCCAGCCCTACTGGACAACCTGCTAGGATTGA
AGTGAGGGTCAGGGAAGGAAAAGCGGTGAACATTCATATGATCTGCAGCCGCAAACCAGG
GGTTTTACTCTCAACAATGAAGGCTCTGGACAGTCTTGGACTGGACATTCAACAAGCTGTTA
TCAGCTGCTTCAATGGGTTTGTGTTGGATGTGTTCCGAGCTGAGCAATCGAACGAAGGTCA
AGAGATGCATCCAGATCAGATCAAAGCTGTGTTGATGGAGACTGCTGGCTTTCAAGGGGGG
ACGATATAA

>SlbHLH066
TATCACAAACTAAAAAAACAAACACAACATAAAAGGACCTGTTCCTTCCTACACACCCAAA
AAAAAATGGCTGATCATCAATTCAACTCAGACATACAAAATTTTATCACAAATTCACCATTTT
CTCTATTAAATTTCGATTCGAGTGTCGATTTAATGAACCAATTTCCAGATATGATGACCATTCC
TTGTTCATCAGACATGTCAAGTTTCAATATACAGAGTTCAATGGAATTTTCGAATGATAATGT
TTTCACTCAAGTAAATGATCAGTTTCCAGGAAGTTTACAAGAAATATTTCAAGGAAATATAC
AACAAGAAAGCAAAAATGAGGAAATTAATGATAGTAAAAAAAGAAAAATTAGTGATACTCC
AGAAAGCAGTTCAGCTTCTGCTACGGGGAATAAAAGAAGAAATACTAAAGGAAGAGGAAA
TAGAGTGAAAGTTGATGAAAAGGAAGAAGAAAAACCAAGAGAAGTGGTTCATGTTAGAGC
TAAAAGAGGCCAAGCAACTGATAGTCACAGCTTAGCAGAAAGGGTTAGAAGAGGAAAAAT
TAATGAAAGACTTAGATGCTTGCAAGATATTGTTCCAGGATGCTACAAGACAATGGGCATGG
CAGGAATGTTGGATGAAATTATTAATTACGTGCAATCCTTGCAAAATCAAGTCGAGTTCCTC
TCCATGAAACTTACCGCAGCAAGCTCATACTACGACTTCAACTCGGAATCCGACATTCTTGT

GTCATTACAGAGAGCAAAGGCATATGAAGCACTAAAAATGCAGAAAATGATGAAGAAGGA
AATTGAATGTGAAGTGATGTCTACAAATCAAGTTGGTCTTCATTTTGGAAGTTATCCAATGTT
GCCATACAACACCTGA

>SlbHLH067
ATGGCTGGAAACCCTAGTAATAACTGGTGGAACAACATGTTGATGAACACAAGCATGCATC
CACATGAATCTCATGAACTTTCTTCTTCTACGACCACGACTCAACATTTCTATGGATCTTCAA
ATTTTTTGGCTGATAATAATCCAAGTCAAGATCATTTACCTCGCTCATGGAGCCAGCTACTTC
TGTCTGGATTATCTTGTGATCAAGAAAAACCTGATATAAGTGATCATTTTCAACATCAATACA
AGAAGCTGGAGAATTGGGAAGAAATCCAAAATTTGAATTCCATCCATAACAATAATATTATT
CCATCTAATTCAAGTTTTAGGGTTCCTATTTTTGATGTAAAACCAGAAGAATTAGTGAGCCA
AAGACTGTATAGTAATTACCATCATGATTTATCACCAGCTAGCTCTTGTGTTACCACTAATTTA
AACCATAATAATATTTTTAACTTCTCATCATCTCCTGCTAACAAAATTACCAACAGTAACAAA
GTGGTTGAAGTCAAGCATCAAGACCATTCATCTGAGTGTAACAGCACAAGCAATGGTGGGG
TAACTAAGAAGGCTAGAGTTCAACATTCTTCAGCTCAACCCTCTCTCAAGGTGAGAAAAGA
GAAACTAGGAGATAGGATAACAGCACTCCACCAACTTGTTTCTCCATTTGGAAAGACTGAC
ACAGCCTCCGTCTTGTCAGAAGCCATTGGATACATCAGATTCCTTCAAGCACAAATTCAGGC
ATTGAGCTCTCCATACATGGGCAATGTAGCAGGAAGCATGGGTCACACTCAACAACAATCT
GCTGATTTGAGGAGTAGAGGGTTGTGCTTGGTTCCTATATCTTGTACACAACATGTTGGAAG
TGACAATAACAATACTGTTGGAGATTATTGGGCCCCAGCTCTTGGAGGAGGAGGATATTTAT
GTAACAATTTTAGTGTTGCATAA

>SlbHLH068
ATGGAGAATTTGGATTGGAATGAAACTCCGTTTAATTTTGGATCTTCATTTGATCCAATACAT
GTAATATCAAATTGGAACATGCCACAACGCCAAGAAGCTGCTAATAGATTGGCTGCTGATTC
TATGGCTGCTAAAGCCGGAGCAGCAGATCTAACCCGGGATTGTACCGATATAGCCTTTTCTT
CTTCCCCAATACTCAATATGCCTAACAATTACATCACTGATAATAATCCATTAGGCCTAATGTC
CGATTTCGGTGTCGAAATTGCTAAGCCAATCTCCAATACTGTATCTCTAGAATCCATTGATTG
TTTACTGTCAGCAACCACCACCAACAATACAGACACGTCAATCGAAGACGATGGCATGTCC
GTAATTTTTGCTGATTCGAATAGCTTGTGGAATTCTGGAGAGTCTGCCGGAATTAATCCACA
GCTGTTGAACTGTCTGCCTACTGATCATAAGATACTGGAAGAAGAACAAAATCAAAGAAAA
AGAAAATCTTATGAACTTGATGAAGCAGTTTCACAATCATCTCTGGGGAATTCTTCGAACGA
GTTCAATCTATTCCAGTCAAATTCATTAGGAGATTGCGGTAATTTCCAGCTTATTTCAGAAAA
ACAATCCAAATCGAAGAAGATGAGGTTAATTGAGAGCTCTAATAAGCGTCCAAGTTCATCA
AACATCAATTTCCAACAAGCTAGTTCATCAGTATCGTCAATTGATCAAGAGCCTGATCCGGA
GGCGATCGCTCAGATGAAAGAAATGATATACCGAGCTGCAGCATTCAGGCCGGTTGATTTCG
GTGCTGAAGTACTAGAAAAGCCTAAACGGAAGAATGTAAAGATATCAACGGATCCTCAGAC
GGTGGCGGCGAGGCAACGAAGAGAAAGGATAAGTGAAAGGATTAGGGTTTTGCAAAGGCT
AGTACCAGGAGGAAGTAAAATGGATACAGCATCAATGCTTGATGAAGCAGCAAATTATTTG
AAATTTTTGAGGACACAAGTGAAAGCATTGGAAGCAATAGGCCAAAAACAAGATCCTTTTA
CTTCAATAACACAATTTAATTACCCAATTCCCATGCAATTACCACATTTTCCACTACAAAACC
CTAACCAAATTCATCGACCAAAGAGTTGA

>SlbHLH069

ATGGATCCTCATTCTACTATAATGAGTGCGTTTCAAACTGCGACTAATTTGGCGGAGATCTGG
CCCTATCACCATCTTCTCGACCACACTACTAATCACGCCGCCACAAAGCGACGCGACGATGA
TGAATCTGCTATTGCAGTTTCAACTAGTGGAAATGCCTTGACTGAATCTGATAGTAAGCGGC
TGAAGGCCACAAGATCAAACGAGAATGGGGAATATTCAGGAGGGAATTCAGGAAAATCTT
CAGACCAACCTGCAAAGCCACCAGCTGAACCACCTAAGGACTACATCCATGTGCGAGCGA
GGAGAGGTCAAGCTACCGATAGTCATAGCCTAGCAGAAAGAGCCAGAAGAGAAAAGATTA
GTGACAGGATGAAAATCCTACAAGACTTGGTCCCTGGTTGTAACAAGGTTATTGGAAAAGC
TCTTGTCCTTGATGAGATAATCAATTATGTCCAATCATTACAACGTCAGGTTGAGTTCCTATC
AATGAAGCTTGAAGCAGTTAATACAAGAGTAACCCCAACCATCGAAGGAATTCCTACTAAA
GACTTTGGGCAGCAAACATTCGAGACAAACGCTATGGCATTTGGTTCACAAGGTACAAGGG
AATATGCTGGGGGGACATCACCAGACTGGTTGCACATGCAGATAGGTGGTGGATTTGAAAG
AACGACATAA

>SlbHLH070
ATGAATATTGTTAGCATGGATGATGCTGAGCCAAAAGATTGGCAATTCAGTGGTAGTAATCT
GATAAATGCATCTAATCGAATTATCGATTCGTTCTGTGTTAGTGTTTGGGGTAATCCTATAAGT
TTATCAAACGTAGGCTTATGTGGTACTAATATTCCAATGAATCCTGGTGCTGCAACTGTGTTG
GATTGGACTCAACCAAATGCTATGTCGAAAGGAGGCACGTTTAGTATGGTTCCTCAAAGCTT
AACTCAATTGACAGCTGATTCAGAGAAAGTTGGTGATATGATGAACCCCTTTGCTATTCCTA
ATTCCTTTAATACGTATCACAACGGACTGATCTTCGGAGAATCACAAGATGTATTCGATTCGC
CTTCTACTGCAATCAGTCAAAAGAAACAGATGAGAAATGCAGTTGAAAGTTCTGATCAGGA
TGTTCCTTTACCTCTTGAATATGAACACACAGATAGAAGCCCCCTCAAGAATGATAAAAATG
TAAACTTTGTTAAGTCTCAGGGTGAAGCAAAGGAATGTGTTGGCATCTCTGAAAATGAGTC
TGAATGTAGTGGACATCAAGAAGAAGTGGAGGGTGGATATTCTTCTGCTAGGTGCCTTGGT
TCAAGGAAAAGGAAAAGAAGTGGTCAGGATGCAGAATTTGATCAAATGAATGGAGCACAA
CAACAACCAGCTGAACTGGCAAAAGAGCAAAACTTGAATTCCATTCTAAGGAGGGAGAAG
ATCAGCGAACGGATGAAATTTCTTCAGGATCTTGTACCTGGTTGCAACAAGCGAGAGCCGG
TGATTTCGCAGGTCACAGGGAAAGCAGTAATGCTTGATGAAATCATTAATTATGTACAATCT
CTTCAAAGACAGGTTGAGATGAAGCTTGCAACTATAAACCCGCGGTTGGATTTTAATATTGA
TGCGCTCCTTGCGAAAGAAATTCTGCATTCACGAGCAGGTACTTCATCCTCACTTGCTTTTG
CACCTGATCTTACAGTGCCGTATCAGTCCTCACATCAACTGCAACGAGGCCTGGTTCATTCA
GGTCTTCCTGGTTCGGGAAACTCCATCGATACATTACTTAAATCTATCTATCCTTTTTTAGCTG
TTACGAGTGGTGGCTACAAAGAGCGTTCATCGCAGTTACCTAATGATGAGCTACATAATGTT
GTCGAGATGGGTCTCTGCACGAGTGCACCCCTGCATATTCGTGAAGGTTCTTTGCCATCAGG
CCAGATGAAAGAAGAACCCTGA

>SlbHLH071
ATGCCACACCTTCTAGCAGTAATACCAAAGGTCTATGGGAAGAGTAATGAGAAGGCTAGTG
CCCTAAGGTCTAAACATTCAGTGACTGAGCAGCGTCGAAGGAGCAAGATCAATGAGAGATT
TCAGATATTGAGAGATCTGATACCCCATACTGATCAAAAGCGAGACACTGCATCATTCTTGT
TGGAGGTAATTCAGTACGTACAGTATTTACAGGAGAAGGTACAGAAGTATGAAGGACCATAT
CAGCCTTGGAGCTCAGAGCCTACAAAGCTTATGCCATGGAGAAACAGTCATTGGCGCATGC
AAAGTTTACCTGCACAGCCACATGCCTTGAAGAATGGTAGTGGCCCAGAATCAACATACCT
GGGAAGGTTTGATGAAAATCTCGCTACTGTTACTTCTACGATGCAACCAAATCAGCAGAATC

CAATTGAATCTCATACTAGCAGGGATGTATCATTTAAAGCACTTGATCAACAAAATGAACTA
GCTAACAAGTCAATCACAACACCCATTCCTCTTCAGGCCGGTATGAAGATGTCAGTGCCAA
ACAATAGTGCTTTCTCAGAGCCTCAACCCAGACCAGTGTCTGATCAATGTCCTAACACAATT
GATGCTCTGAATCATGACGAGGATGACGTAATAGATGGAGGCAGAATCAGCCTTTCAAGTTC
ATACTCGCAGGGGTTTCTAACTTCGTTGTCGCAAGCGCTGCAGAGTACTGGTCTAGATCTTT
CAAAGGCTACTATCTCAGTGCAGATTGATTTTGGGAAGCGCGCAAACCAAGCAATGACCTC
AGGGCCATCTATTGCGAAGGATGACGAGAATCCTACTCTCTCTGGTCATCAACATACGGATC
ATTTCAGAGAGGCAAGCAATGACGAAGATATGAACCAAGCTCAAAAGAGGCTGAAGATATA
G

>SlbHLH072
ATGTCAAATCGAAGAACACGCGGTTCGAGACAATCATCAGGAGCTTCTAGAATAAGTGATG
ATCAAATTGCTGATCTCGTATCAAAGTTACAATTACTTATCCCTGAAAGCCGGAGCACTAGG
AGTTCCGATAAGGTTGAAGCTTCCAAAGTGTTGCAAGAGATATGTAATTACATAAGAAGTCT
ACACAGAGAAGTGGAAGACTTGAGTGATAGATTATCAGTGCTTTTGGAATCTACTGAAAGT
GACAGTGCTCAAGCTGCTATTATTAGAAGCCTATTTATGTGA

>SlbHLH073
ATGTATGGAAGTGGAAATAGATCATCAATTCTACCATGGAATAGCTTCAACACACTTGAAGA
TCCATTTATTTTTCATGGATCATCATGTTATGGAGATTTATTCAACAAAATTATACCACAATTT
CCATGTCAAGATCATAATGAAGTGCAAAAAATGATGAATTCTCAAGAAATAATGGATGCAAA
GGCACTTGCTGCCTCAAAAAGTCACAGTGAAGCTGAAAGAAGACGTAGAGAAAGAATCAA
TAATCATCTTGCTAAATTACGTAGCCTTCTCCCAAACACTACTAAGACAGATAAAGCATCACT
ATTGGCTGAGGTGATACAACATGTAAAGGAGCTAAAGAGACAAACATCACAAATAGCCCAA
ACAAATCCACTAATCCCAACTGAGATTAATGAATTAACAGTTGATTATTGCAATAATGAAGA
AGGAAATTTCATGATCAAAGTTTCATTATGTTGTGAAGATAGGTCTGATCTTTTACATGACCT
AATCAAGACCTTAAAATCTCTAAGGTTAAAAACACTCAAGGCTGAAATAACAACACTTGGT
GGACGTGTGAGAAATGTCTTGTTCATAACAAGGGATCAACAACAAGACAACGACGATACGT
GGCCTATCAATGATAACAATAATGATAATGATGATGATCAGATGAAATATTGTCTAAGATCAA
TTCAAGAAGCACTTAAAGAAGTTATGGAGAAATCTAATGGAAATGATTCTGGTAATTCAGGG
AGTATTAAGAGACAAAGAACTAGTAATAATAATAATATACACTACTAAAAATAAATAGGAATT
GTGTCGTTACGTGGCTAA

>SlbHLH074
ATGGGTGATTCTTCTTCTTCTACTCCTCTAGATTTTCATGCTTTGAATTCTACTTGTAACAATA
ATTCCTCTATTTTGATGAATTCAAATATGGAACTTCTAAATAGCATAAGCCAACAACTTGAAA
ATGATCAAAATTTCTCCTCAAATAATATTCATCAACAACATGGTTTTTTGTCTTTGTCATCAAA
TGATCAAAACTTCTCTAATCATCATCAACATGAACTCAACATCATGTCTAATTTTCATAATGAT
CATCATATGAATAATATTAGCCATGATGTTTATGATCCCGCCGTCGCGGTTGCTGCTGCTCAAT
TTTTTACTTTGGGAGGTCCAAGTTATGGATGTACTAGCTCAATTCCAGAATCAGAATCCATGT
TGAATAGTAGTAATAATAACATTAATATTCCAACTCCTCATCCTCTAGTCTCTGGAAATACTAC
TAGCAAGAATACAAGTGAAGGGAGAAAGAGAAAAAGGAACAATCAAAAGGAAGTTGAGA
AACCAAGAGAAGTTGTCCATGTTAGAGCAAAGAGAGGCCAAGCTACTGATAGTCATAGTTT
GGCTGAAAGACTTCGAAGGGAGAAAATAAATGAAAAACTCAGATGCTTGCAAGAACTTGT

TCCTGGATGTTATAAGACTATGGGAATGGCAGTGATGTTAGATGTAATAATCAATTACGTCCG
GTCATTGCAAAATCAAATTGATTTTCTTTCAATGAAACTATCAGCAGCAAGTTTGTTTTATGA
TTTCAATTCATCAGAGATGGATGATATGGACTCAATGCAGGGAACAAATGGGTATGCAGCAG
CTCAAGGAATGGGGAAAAATATTGTTGGAGAAGGGTATGGAGGATTTCCTCAATTTCAAAC
ATCTTGGCCTCTTTAA

>SlbHLH075
ATGCTTGCAAGATTTGGAGTATGTTATGTTGCGGAAATTGAGACTAATAATGGAAGTGGGAT
TACATCAAGAAGAAATGAAGATGGTTTGTACCAATATCCAAAAGCAAGTTGTGTAGAAATG
CATAAAATGAATGAGAGGAGACGTAGATACAAAATTGCGAAAAAGATGAAGGTACTAGAG
ACCCTAATCCCTAATTGCAACAAGTCAGATAGAGCCTCAGTACTTGATCAAGCCATACAACA
CATTCAAGCCTTACAACACCAAATCCAGGTGATGTCAATGGATAGAATCCGTGGATCGACTC
TAGTAGCTGCAGGGAGAAACCAAATAATGCAGAGTACTCTGCATTTTAATCCTTACATAGGA
GCAATTGGCTATTTTTTCAACTTTAGCAATATTTTGTGCTCCAATTTTTCCCCAATGTTGTCGA
CGGGAAGTGAATTTCCATTTTTGCCCCTCGCCGTCGCCTGTAACTTACTGCATCCCGGGCCT
ATAATGGAAGTATTTACGCGAGGCTCTGCATCCGTTGCTCCACTAGAAAAACGCGTTTAG

>SlbHLH076
ATGTATCAATTTCCAAGTTTTTATGAACTAGGTAACACATGTTCAGATTCATATAATAATTTTC
TTCATGAAATTATAACTTCATCATCAAGTGAAATGTTTAATAATATTAATAATTTAGAAAGTTC
AAGTGTTAGTCCAAGATCAATGGCTGAAGCTAAAGCAATTGCAGCTAATAAAAGTCACAGT
GAAGCTGAAAGAAGGAGAAGAAAAAGAATCAATGGACATCTTGCTACTCTTAGGAATCTTC
TTCCTAACACTATCAAAACAGACAAGGCCTCTTTACTAGCAGAGGCAGTAAGGTGTGTAAG
GGAACTAAAGCAGACAACATCAGAACTAGGAGCAACAACAACAACAACAACAATGTCTGA
AAACGACGACGATGATGATCACACGACATTAATGACGAAAATAATGTTTCCGAGTGAATCG
GATGAATTAAACCTAAGTTATTGTAACGAGAGTAATAATAACAATACTGATAATAACAACGA
CGATAGAAATTTAATAATAAAAGCATCAATGTGTTGTGAGGATAGGCCAGAAATAATGATGG
AATTAAGAAGGGCATTGAGTACAGTAGAAGGAAAAATTGTAAGGGCAGAAATGTCAACAG
TAGGAGGAAGAATTAAGTGTATTTTGTGGCTAGAAATGTTAGAGAATGGATGCAAAGAAGG
GCTATTTGTGCAATTAAGAAGAGCATTAAAAGTGGTTATGGACAAGGCAAATTTTGGCCCAC
AAAATATGGGCCAAGATTTATTGGGAAATAATAAAAGGCCACGTTTACTAGGGGGACCAATC
AATTATGCTACCTAA

>SlbHLH077
ATGGACTGTCTTTCAGAGATCTTCTCTTCCAAAAAAATCAAAGATCATGATCAACAACAATT
GATTCAGATTCCTTCTACTCCATGCCAACCACACCATCAAGATCTTCCCACAAATGATCCTAA
TTATAAAAGGAAACGATCGATAAGTGATCATAAAGAAAGAGATTCTTTAACCAGAAAGAAG
TCGGTTCAAAAAAAAGTTTTGCATAGAGATGTTGAAAGGCATAGAAGACAAGAAATGTCTA
ATCTTTATGCTACTCTTAGATCACATCTTCCTCATATCAAGGGAAAACGTTCATTACCAGATG
ACATGCAACAAGCAGTGAATCATATCAAAAACTTAGAGAATAATATCAAAGAACTTGAAATA
AAGAGAAAAAAACTTGAAGATTTGGTCTTGTGTTCTTCCAAGGAGGATAAACATTTTGGTG
ATTATGTGAAAATAAACTTATGTGATTGTGGTGTGGAGATTTTGATCAATGAAAGGATTCCCC
TCTCAAGAGTACTTGAAGAGCTAGTAAAGAGACAACTCAATGTTGTTAGTTGTGTCTCAAC
CAAAGTAGATGAAAGATTACTCCATAGGATTCAAATTGAGGAGAGTGATGTTTCATGCATGT

GCATGGATGGTCTAGAACAAAAGCTTGCAGAGATGATTGTGTTCCACTCTTGA

>SlbHLH078
ATGTTCACGAGGTCAAGATCAATCTTGATGGCGTTAGAAACAGTGATTTACAATCAAGAAA
CATTTGGGTACGGTTGCAAAGAGTACTACAACAATAATTTAGGTAACTTCAGTTATAATTATG
AGTTGGGATTAACACAAGGCGAAGGAGAAATGAGTTTTACAAATTTGTTGGATCATAATAAT
AATAATAATCGTGAAGAATCTTCATCATCACCAACGGAACTAATGTACAACAATAATGCAAG
AGAGTACAACATTTGGGATCCAAACTCTTCGCTAGAAGATCATCACCTTTTTATGGAAGGTT
CACCGGCGGCTGAACTTCCGGCGGCGGCGGTCAGATCCGCCGCCACCTCCGGCCGCCGTA
AAAGAAGACGGACTAGGAGTTGTAAAAACAAAGAGGAATTGGAGAATCAAAGAATGACTC
ACATTGCGGTGGAACGGAACCGCCGTAAACAGATGAATGAGTATCTTGCTGTTATTCGATCT
TTAATGCCACCTTCGTATGTACAAAGGGGTGACCAAGCTTCAATAATTGGAGGAGCCATAAA
CTTTGTGAAGGAGCTAGAACACCATCTTCAAACCCTAGAAGCTCAAAAGAGAAGTCATCCT
CAAAAACAAGAGCATTCAGATAATCATGGCTCATCATCAACTCCACCATTTGCTGATTACTTT
GCGTTTCCGCAGTACTCAACTCATTCCAAGAGTACATCTCCGACAGCAGCAGCATCAGATGT
TGCTGCTGCCGGATCATGTAACTCTCCGTTAGCCACGGAGAAAATGTCGGCGTTGGCCGAC
ATTGAAGTTAGCATGGCGGAGAGTCATGCTAACCTCAAGATACTATCAAAGAGAAGACCAA
AACAACTCTTGAAAATAGTGGCTGGGCTTCAGTGTCTGTGGCTCACTGTCCTCCATCTCAAT
GTCACTACTGTTGACCACATGGTTCTTTACTCACTCAGTCTCAAGCTAGAGGAAGGGTGCC
AGTTAACTACTGCAGATGAAATTGCTGACTCTGTTAATCAATTGCTTGGTAGAATCCAGGAG
GAAGCTGCTTCAAGTTCATGA

>SlbHLH079
ATGACTCCTCAGTCAAATGAGATTCCTTTAAATGGGGATTCTCCATATTTCACATTTCCTGGT
CTGCCAGTGTCAAATGCAAGCTGGCCAGCAGAAACTTGCAATTGGTTATATTATTCGCCTCT
GTTTTATCAGGGCTTTAATCCTGTTTCGACCACTTTACCTAAAGAGAAGTTAGCTCCTAGAG
CACTTGAAAATCTGGAAGGTAGCAAACATCCTAATGGAGGCACCACATCTACTCAGAAGAG
ATTCCTTGTGTTTGATCAATCTGGTGATCAGACAACTTTGATCTATAATTCTGCTAATGGTACT
CACGTACAATGCCCGGCTTCTCTGAATCCAAAAGCCCCAGCCCTTTATAAGGAAGATCCAG
AGATCAAAAGAAATGAAACTTCTCCATTTGGGCATTTCTTCGGTGATGAATATTATGAAGAA
AACAACAGAGATGATGTTGAAAGTGAAATGCATGAGGATACTGAAGAACTGAATGCCCTAC
TCTACTCTGATGATGATTACAATTATTCCGAGGATGATGAAGAAACAAGCACTGGTCACTCG
CCTAGTACTATGACCACTCATGATATGCGCGAGTGTTTTGACGGAAGGGGTGAAGAAGTTG
CTAGTTCTGCTGGGGTGACTAAAAGGCATAAACTGCTAGATGGTAGCTACGATGCACCAGA
GCTCAGGGATACTGCAACCTCCGCAAAAGCCTATACGTGCTCCGACTTAGAGGATGATGCA
CAATCCAGTTGTGGCAATGGTCTCGAACAAGACTCAGGAGCACCAGATTCTCCATCTGGGA
AAAAGAGGCTAAGAAAAGATAAAATCCGCGAGACAATAAGCATTTTGCAGGAAATAATCCC
CGGAGGGAAAGGAAAAGACTCAATGGTTGTTATTGATGAAGCAATCCATTACTTGAGATCC
CTGAAGATGAAAGCCAAGTCCCTAGGACTTGATTCTCTTTGA

>SlbHLH080
ATGTTATCTTCATTACAAGGATGTAATAATGTTTTTTTGCAGCTTGTTGATCATCAGAAGAAT
GAAAAATCAAATACAACAAAGAAAAGTAAATCAAAAGATGCAGCTGTAACACATGCTGTAG
CTGAACGTAAACGTAGAGAGAGAATCAATTCTCATCTTCATACTCTCAAGAAACTCTTCCCT

CATCTCCCTAAAAAGGACAAGCCAAGAGTGTTAACAGAAGCAGTCACTCAACTAAAGGAA
CTAAGGAAGAATGTTGCACAACAACTAGAGTTGTCATCTTTATTCATACCTAGTGAAAATGA
TGTAGTTATAATAAATTATTGTGACAATATTAATGACGAACGAACCGTCAAAACTACGATTTG
CTGTGAGGACAGACCGAGTCTGAACCGGGACTTATCGTCCGCGATTCAATCGGTTCAAGGA
CGGGTGATCAAGGCGGAGATGGCCACGGTAGGGGGGCGGACCAAGGCGGAGTTGGTGGT
GGTGTTGGGCAAGGCAAATGGTGGAGAAAAAGATGTTGGACAACTTAAACGGGCTTTAAA
GGCTGTTGTGGAAAATAGGGCTTTGGGCTTTGGAAGTAATGTGATGTTGGGCCGAAGATTT
GGGTGA

>SlbHLH081
ATGGAGTATTACAATGAGAATGGGTTCTTAGAAGAATTATTATCTCTAAGAAGTGAGTCATG
GGATACTACAAATGTAGTCCCTATGGAAATGTCTCATGATTTTTACAGTAATGTCCTCAACTA
TGATAATATTCCACTTCCTTGTACTACTACTTCTAATTCCTTTGAAGGGTATTCATGTAATTTG
CCATTTGACCAACAAAATTTGATAAATTGTGGTACTTCATTTTGTAGTCCATTTTGTGATGAA
TTGTCACCACCATCTTCATTTCCTTCTCAAGATGATTTTTCATCAATTTTGGATGATGAAGTT
GGGAACTATAGTTTTCAAAATTTGGAAATGGGGAATAATAGTTGTAATAATGATAATAATAGT
AATATTGTTATTCCTTGTAAATTGGAGGAAAATCAAGTGTGTGAAGGTGCAGGAGGAAGAG
TTGGTGGAGCTTCAAGTTTTAACATTGGTTTTTGTCCAGAAATTAGAAAGTCAAAATCAAAG
AAAATGGATGGTCAACCTTCAAAAAAATTTAATGGCTGAGAGGAGGAGGAGAAAAAGGCTT
AATGATAGACTCTCTATGCTTAGATCAGTTGTTCCAAAGATTAGCAAGATGGATAGAACTTC
AATACTTGGGGATACTATAGACTACATGAAGGAGCTACTAGAGAAAATCAATAATTTGCAAG
AAGAAATGGAACTTGGACCAAATCAACTAAGCTTGATGAGCATTTTCAAAGATGTAAAACC
AAATGAAATGCTTGTGAGAAATTCACCTAAGTTTGATGTGGAAAGGAGAAGTGTTGATACA
AAAGTTGAGATATGTTGTGCAGGGAAGCCTGGTCTGTTGTTATCAACAGTGACCACACTAG
AGGCTTTAGGTCTAGAGATTCAACAGTGTGTAATCAGCTCATTTAGTGATTTTGCAATGCAA
GCTTCTTGTTCTGAGGAAATGGAGCAGAGAGGAGTTGTGAGTTCAGAAGACATAAAGCAA
GCATTGTTCAGAAATGCTGGATATGGAGGGAGGTGTCTGTAA

>SlbHLH082
ATGGGTACTAAGGAAAATGGTTCTTTCAATTGCCCTTCTACTGGGATGAATAGAGCAGATTC
AATGCCTAATGTTGACCCTTTTAGTGGTTCTGGTTGGGATCCACTTCTTTCACTGAATCAGA
AGGGGGGATTTAAAGGATCTTCAGTTGTTGGTCACAATGAGTTTGTCAATTTGCCCTATCAA
TCATCTCAGTTTGTTCACTATCCATCTGATTCAAATCTAGCTGAAATGGTCCCAAAGATTCCA
GCTTTTGGAAATGAAAGTTACTCAGAATTGGTCAATACTTTTCCTTTACAAGAACAGCTTAG
AGGGGCAAATTGTTATGCTAACTATGTTAAGAACAGAGGGATTTCCACTGAAGGAGAGTGC
CAAATTTCTGGTGAAGGTGCTGTGGAGGTTTCACCTAATGGGAAGAGGAAAATATCAGAAA
ACCATTCTTTATCCAATGCCAACAAGAATGTTGAAGGAGAGCTGCAGAAGGCTCCATCAAG
AGATAGTTCAGACTGTTCAAAAGAACAAGATGGTGGAAAAAGACACAAAACAGACCAAA
ACGTTAGCTCTAACTTAAGGAACAAGCAAGCAGGGAAACAAGTTAAGGATGACTCTGATG
GTGGAGAACCTCCTAAGGATAATTATGTTCACGTTAGGGCTAAAAGAGGGCAAGCCACAAA
CAGCCACAGCCTTGCAGAAAGGGTGAGGAGAGAAAGGATCAGTGAGAGGATGAGATTGCT
TCAAGAATTAGTTCCGGGCTGCAATAAGATAACTGGAAAAGCTGTGATGCTTGATGAGATTA
TCAACTACGTGCAATCGCTGCAACAGCAAGTTGAGTTCCTGTCAATGAAACTTGCTACTGT
AAATCCGGAACTGAACTTCGATATTGATCGTATTTTATCAAAAGAGATGCTCCATCAGCAAA

CTAGCAATGCAGCTCTTCTTGGTCTTGGTCCAGGGCTAAGTTCCTCTCTTCCTTTTCCTGGAA
TTTCTCATGGAAGTTTTGCTGGTATCCCCGCAACGACACCACCCTTCCATCCCTTGCCTCAG
AATGTATGGGACAATGAGCTTCAAAGCCTTCTTCAAATGGGATTTGATTCAACTTCATCTATG
AACAATATGGGACCAAATGGACGGTCAAAGTTGGATCTGTAG

>SlbHLH083
ATGGAACATGTTGGAGCTTTTTTTGATGAAGAATGGGAGTCATTGAGCAAATTATTCTCTAG
TACTGAAACTGCAGATTTCATGCTGCAATTGCAAGGTGATCATGGAAGTATGTTCTCGATGA
ATGGTAGCGATAATGCTGGCTCGAGCTATAGGACTGATAATCCACAAGTAGCATTCTCTCAAT
TGTCTGATGAAGTTAACAATAACTTTCAATATTTATCTCAAGAAAGTAGCATTACTAGCTGCG
GAAGTGATCATGGAATGTTTTTTACTAACCCGAGCCACGACCATCTGCAGCATTCTAACAAC
ATTGATGATGTTAACAATAAGCTTCTTCAAGTAGCAATCAACAACAACCTTCCTGATGAGTA
TCAGTCCATTGATTTCTTCGATATGGACAGCAAAAATCTTGAAAATTTGTGTATTAATCAAGA
TTTTCAAGCTGAAATGGTATGCGATCAATTGGACAATGCTGGGATATCCCCTGTTCCACACA
AGGAAATGCAATTGAAAAGGAAATGTGACAAAATAGCACATGAAAATCCCAAGAAGAAAT
CGCGGGGTTCACAAGATGCACAGAAAAGTACTAAGAAAAAGATGCAGCCAAAAAAGGGG
AAAAAGAATCAGAAAATGACTCAAATTAACAATGAAGAAGGAGAAGAAGAGACTAACAAC
AATGCAGATAACCAAATTGCTCAAAGCTCGAGTTGCTGCAGCTCTGAGGATGATTCTAATGC
ATCTCAAGAACTAAATGGAGGCACGGTATCTTCAAACCCCAAAGGAAAGTCAAGAGCTAG
CAGAGGAGCTGCAACAGATCCACAAAGCCTTTATGCAAGGAGAAGAAGAGAAAGAATTAA
CGAAAGATTGAGAATCTTGCAGAACCTTGTTCCTAATGGCACAAAGGTTGACATTAGCACA
ATGCTTGAAGAAGCAGTTACCTATGTGAAGTTTTTGCAGCTTCAAATTAAGTTACTGAGCTC
AGATGAACTTTGGATGTATGCACCACTTGCTTATCATGGAATGGACATTGGCATCTATCAAAA
AATGCTACCAAATATGCAATGA

>SlbHLH084
ATGCTAAATTGTCTGTTACAACATACACTTAGAAGTGTCTGTACTTGCTCTGACTCTTCTTCT
AATGCTTCTGAGTGGGTCTATGCTGTTTTCTGGAGAATTGTCCCAAGAAATTATCCTCCACCT
AAATGGGATCATGGAGGAGGCTTGCTTGATCGCGCTAAAGGGAACAAGAGAAACTGGATT
CTTGTTTGGGAAGATGGTTTTTGTGACTTTTATGAATGTGAAAGATCGAAAAGGGAACATGT
AACGATAAATTTTGGTCCTGAAATCTTCTTCAAAATGTCTCATGAGGTTTATTCTTTTGGTGA
AGGATTGGTGGGCAAAGTTGCAGCAGATAATAGTCACAGATGGGTGTCTAAAGATGCCCCA
AATGAAAAAGATTCAAACTTTACTTGCTCATGGAATATGTCTATTGAAGCTCAACCAAGAGC
CTGGGGAGTTCAGTTTAATTCAGGCATTCAGACTATTGCTATTATATCTGTTAGGGAAGGCAT
AATTCAGCTTGGCTCATTTAACAAGGCATTTGAAGATCATAATCTAGTATTAAACATTCAGAG
GAAATTCAGCTACCTTCAGAGCATACCGGGTATTTATGCAATTCAGAGACCATTTTTGCCAAT
CCAACATCCATATACTTACAAGCCAAACAATGTCACTCTAGTTAACGAGACGGATAATCAAA
TGGATGACAAAAACCAGATAATTGGGTCGAAAAGAGTTCATGAGTTTCCGTTCAAGTCTAT
CAACTTTGGCTATAATAGCCCACAAACTATGGCTAGTCTACCTTTGTGGTCGATGCCTATAGC
AGCACCGTCTTGTTATGCTAATGCAGCACATGAGATGAGTTCTTTACATGACAGAGTTACGA
GGAACACTACAAGCAAGGATGTCAAGGTTGTCGATGAATTAGGCCATTTGAAGTTTGAGAC
AGATGAGGAAAATGATCAATTTTCCTTAAACCAGAACTTAGGCCTGGAAAATAAGGTAGTT
GAGGTTGGTTTTAGACAATTAGGAAACGGAGGGGCTGCTCCAAATCCAAATTAA

>SlbHLH085
ATGGCGGATCCGTACAGAACGAACCCTCACGCTTCTTCATCTCTCGAATCCGAAGATATGTC
TTCCTTTTTTCTTAATTTTCTTCAAGGAACGCCCGCTTCCTCTTCTGCGACTGCTGCGGCTGG
TTTTTATAACCGATCGGGACCGGCGCCGGTGGCTGAGTCATCTTCTAGCCTCAATTTCTCCG
ATCCCGGCCGATTTTATGCTGCCGAGTTCAAGGAAGGCGTTGAGAATGTGTTTGCTTCTGCT
GGCCTCGGGGAATGTGACGGTATGAATTCAGCTAATAGGAGAGAGTTTTTGGAAGATGATA
AGGTTGATAACTTCGGTTTTAGTAGCGAGGAATGTGATGGACTTGACATGCCATCTGATCCG
ACTCATCCACGTTCTTCAAAGAGGAGTAGATCTGCTGAAGTTCATAATTTGTCCGAGAAGA
GGAGGAGGAGTAGGATCAATGAGAAACTGAAAGCATTGCAGAACCTAATTCCGAATTCTAA
CAAAACTGACAAGGCATCTATGCTTGATGAAGCCATTGAATATCTGAAGCAACTTCAACTGC
AAGTTCAGATTCTTACCTTGAGGAATGGGTTGAGTTTATACCCAGGATATGTTCCCGGGTCTT
TGCAGTCGGTGCAACTTCCATCTGGTAATGAATTTGATGGGAGAAGTTTTATGCTGAGTGCA
AATGGAGGAGCTACACTTCCTGTGAATCGAGAAATGCCACAAACTGCATTTGAAATTTCCA
ATCAGAATCCGTCAGGCAAACCAACAATAACCTCACATAACACAGAAAACGCTGTAGCTTT
AGAAACTACAATTCAGAATCATTATGGACTTCTTAACCATTTAGCATCTTCTAAGGACATGTG
CCGAGACAATACACTATCAAGATTGCATTTAGATATGAGTTGCTCTGGAAACAATTCATCATC
AGGAGTGTCGTCTTAA

>SlbHLH086
ATGGATATTGATATGCTTAAATCATCAGCAACTTCTGAAGATCAAATGGAAATGATGCTCATG
ATGCAATTGGAGAAATTTCCTGAGTTTTCTACCGGTAACTGTTCTGAATTACCAATGATGGA
GTTTAGTCCACAAGGAAGTTGCAACAGCAGCAACAATTTCCAACAAATGGATCAAAATTCA
CCTAATTTTCTCAACATGCCTTCTACTATTTCATTCACAAACTCACCTCCAATTCATCAAAATT
CACCTAATTTTATACCTAATTCGGGTGGATTTAATTCGAATTCGATGAACCGAAGCAATATGG
CAGCAATGAGGGAGATGATATTCAGAATTGCAGCAATGCAGCCGATTAACATCGATCCTGAA
TCAGTGAAACCACCAAAGAGAAGGAACGTTAAGATATCAACGGATCCACAGAGCGTTGCG
GCGCGACACAGGAGAGAAAGGATAAGTGAAAGGATAAGAATATTACAGAGATTAGTACCA
GGAGGAACAAAAATGGATACTGCATCAATGTTAGATGAAGCAATACATTATGTGAAATTTTT
GAAAAATCAGGTGCAATCACTGGAAAGGGCAGGTGCAACTAGGCCAGCTAATGGTACTGC
TCTTACTGCTGCAGCTGCAGGATTAGGATTCCCTGTACCAATGTCTTTGAGTGGGAATTATAA
TTTGCCTGTCAGTACAAAAAAATTATCATCATCAAAATATTCAACAATATGCAGATGTCTAA

>SlbHLH087
ATGAGTAGTACTACTCATCAAGATATGGGATTTCATCAATGGGCTAATAATAACATTAAGCAA
GAAAATTCATTGGATAATTCATATCAAAGATTTACTCAAATGTTGAAAAGTCCAGAAGGAGG
AGGAGAATTGAGTGATATGAATGCTAAGCTTTTGCTTGGTACACTTTCTAATACTGGCCTTCA
ACTTTATCATGGAGATAATAATAATCTTCTGTATTCTTCAAATTCTAGTAGTATTAGTACTATTA
ATAGAGGGAGATTTAGTCAAATATATCCCACAATAAATGTATCCAATTTGAATATAAATCATCA
AGCAAATTCTTGTAGCTCTTTGGATATGAATTTACAGCCATTGGATCTCATCAATTCTACAAG
ATATGGAGGGAGTTTTAGCCAAACATATGGCTTAACAACAAATCATTTTCAGCATTCATCAA
GTGAAAGTCCAGTAAATAGCTCCACAAGTATATCAGCCTTTAGTAATGGAATGCCTGAAGCT
AAAAGGACCAGCAATACTTTGGAGACTAATAAAGGTCCTCAAAATGCACCAAAAAAAATCA
AGAGTTGATTCACGCGCATCATGTCCACCCTTTAAGGTGAGAAAGGAGAAATTAGGAGATA
GAATAGCAGCTCTTCAACAATTGGTAGCACCTTTTGGCAAGACGGACACAGCATCTGTACT

AATGGAAGCTATTGGTTATATCAAATTTCTTCAAAACCAAGTTGAGACACTAAGTGTGCCAT
ACATGAAATCATCACGAAGCAAAGCCAGCAGATCATTGCATGGAGGTGGTGGAGAGATGAA
TAATGAAGAAATGAAAAGAGATCTTAGAAGCAGAGGTTTGTGTTTAGTGCCCTTAACATGTT
TAACTTATGTTACTGAAGGTGGAGGAGGTGTTTGGCCGCCCCCTAATTTTACTGGAGGCACT
TAA

>SlbHLH088
ATGGAAAAAGCATTAGAATGGTTAAGACCACTTGTTGATTCCAAAAATTGGGAATATTGTGT
TGTTTGGAAGTTTGGTGATGACCCTTCAAGGTTTATAGAATGGATGGGGTGTTGCTGTTCTG
GTGCAAATGGAGTTGATGTCAATGTTAAGAGAGAAAATGGAGGGAAACAGACATTTAGTTC
TCTGTGTAGAGATATTCAAGTGCAGCATCCTATTAGAACAAAGGCTTGTGAGGCTTTAGCTC
ATTTTCCTCATTCTATTTCTCTTTATTCAGGGATTCAGGCAGAGGTTGTAACATCAAATGAAC
CAAAGTGGATTAATCATGCTGAGATTTCCAATTCAAATTTGTCACATGAATTAAAGGGTACC
CTCGTATTGATCCCCGTTGCTGGTGGATTGGTTGAGCTTTACAATTCAAAGATGATATATAAA
GATCAAAAGACGATTAATTTCATCATCAATCGGTTCAAACTTGGTTCAGAAGAAGCCAATAG
TTCCGTAGCACAGAAAGAAGATCAAGTTCTTGATTTCTTTCCCTATGAGAAATCAAACTTCT
GTGCTCCTCTCCTGCAATATGCCACAAGTTTTCCTTCAAGTGCACCTCATATTTCTCAAGTAT
CTGAGTCTAGTGCTAATCCCAGCATTCAAGGATCATCGACCGGTTCCATCCCTTCAAATGAA
CTTACATTGTGTCATTCACCTCCTGACCATTTATCGCGAAATGTACCATTAAGTCAATCTACT
GAAGGGTATTTTGAGCACACAGAGCTTCAGTGCAGTGGAAACTTATCAAGAATGGAAGAC
ACTATTTTTCCTTGGAAACAAGAAAACTATATAGTTGCAGGAGATATGTTCAGTATGGGTAA
GAAAAGGCAAAAAGGACCTTATCAGTCGAAGAATCTTGTCACGGAGAGAAAACGAAGGA
ACAGAATCAAAGACGGTCTTTTTACACTTCGAGCTTTAGTTCCCAACATCACTAAGATGGAC
AAAGTCGCAATACTAGGAGACTCGATTGACTATATAAATGAACTGCAAGAAAAAGTGAAGT
TATACAAAATTGAGCTCAACAAAATAGAAGCGGAAGTTACCAACAATGAGAGTACTCCTGA
AATGGTCCTATCAGACATGACTGAAATGTCCAAAGTTACCGGACAAACAAATGAGAAAACA
CAAATTTCAGTTAATACAACTGATAGGACGAGAATGGAGGTGGAAGTGAATCAAATTGGTG
CAAGAGAGTTCTTGTTGAAGGTCTCTGGATCACGTAAACCTGGTGGATTTACGCAGTTGAT
GGAGGCCATGAATTACTTAGGACTTGAGCTAGTAAATGTGAGTTGTACCACGTCTGGAGGG
GAGATCGTGAGCGTTTACATAGTAGAGGCAAATGTAGACAGATTTATTGATGCACAGAAACT
GAGATCTTCACTAATTGAGCTAACAAGTTGA

>SlbHLH089
GCTTCTTATTATAAAAAAAGAAAAAAAAATATATTTCATATTCTTCTAAAAATAATGGAAGAA
CAACTAAATTCTTTAGCTATTACTCATCTTCTTCAACACTCTTTAAGAAGTTTATGTATTCATG
AAAATTCTCAATGGGTTTATGCTGTCTTTTGGAGAATCTTGCCTAGAAATTACCCTCCTCCAA
AGTGGGATAATCAAGGTGGAGCATATGATAGGTCAAGAGGAAATAGAAGAAATTGGATTTT
GGTATGGGAAGATGGTTTTTGCAATTTTGCAGCATCAACGGCTGAGATTAATGCAAATGAAT
GTCCAGGATCATCTTCTAATAATAATAATAATAATTATGGAGAATATCAACATTATCAAGG
TCTTCAACCTGAACTTTTTTTCAAGATGTCACATGAAATTTACAACTATGGAGAAGGTATAAT
TGGAAAAGTGGCAGCAGATCATAGTCATAAATGGATCTATAAAGAACCAAATGAACAAGAA
ATTAATTTCTTGTCTGCATGGCATAACTCTGCTGATTCTCACCCTAGAACTTGGGAAGCTCAG
TTTCGTTCTGGTATTAAGACTATAGCCTTGATTGCTGTGAGAGAAGGTGTCATTCAATTAGGA
GCTGTTCATAAGGTCATTGAAGACCTAAGCTATGTGGTGTTACTAAGAAAGAAGTTTAGTTA

CATAGAAAGCATTCCGGGAGTGTTATTACCACATCCCTCTTCCTCAGCCTATCCTTTCAAGGT
AGACGGATACGGGGCGTCACCAGACGCGTGGCATTTTCAGACTAATTTACCAACACCAACA
CCAACACCAACTGAGTTATACGAACATTTCAATCAACATCAACACATGAGGATCACGCCTTC
AATGAGCAGCTTAGAAGCACTTCTTTCCAAGCTGCCTTCAGTCATTCCAGCAGATGTTGCTG
CTGGAATGACAGGGGGTTCAATACCTACTACATATTGTCACGAGTACCAACAACAACCCCAA
TATAGACCTAATGTTGAAATATTGGGGTTAGAGAAAGTTGCAAAAGAAGAGTATGAGGACG
AGGAGGAGGAGAAGGAGAATAATAATAACGAAGAGAAAACTCGAAATAATAATAATAGTAA
TGAGAGATTAGATCATAATGGTGGTGAGAGTAGTAGTTCAATGTCATCTTATAGTCAACATCA
TCATAATTATCATCATCAACATTATGGTTATCATCATGATTTGAATGTAAGTAGTAGTATGCCTA
ATAATGGATATTAA

>SlbHLH090
ATGGATCCACAAGCTTCCATGATGAACCACGCCGGAGGATTTCAGTCTCCGCCGTTTAATTT
ATCTGAGATCTGGCAGTTTCCGATCAATGCAGGTGAAGGTGAGACGCCGTATAGTTTTCCGT
TGTCTACGGCGGCTGCGCCGCAGAATGTGAGTGATGATGTTCGGAATAATGATCCTATGGTT
CTAGACCGGAGAACTAATAATTACAGCGGTGGCGGCGGTGGTGGTGCAGCTAGGAAGCGA
AACGAGGATGATGAATCAGCTAAAGGAGTTTCCACTAGCGGCAATGGCTTGACTGAATCTG
CTAGTAAGCGGATGAAGGTTACAAGATCAAATGAGAATTGCGAAGCTAGAGGTGACGGGG
AAGGGAATTCAGTGAAATCTGCAGAACAACCTGCAAAGCCTGCTGAACCGCCAAAAGATT
ACATTCACGTGCGGGCAAGGAGGGGTCAAGCTACTGATAGTCACAGTCTAGCAGAAAGAG
CTAGGAGAGAGAAGATAAGTGAGAGGATGAAAGTCCTCCAAGATATAGTCCCTGGTTGTAA
TAAGGTTATTGGCAAAGCTCTTGTTCTTGATGAGATAATAAATTATATTCAATCATTACAACA
CCAGGTTGAGTTCTTATCAATGAAGCTTGAAGCAGTTAATTCAAAAATGCCAAGCATAGAG
GGATATCCATCTAAAGATTTCGGACAGCAGCCATTTGATACAAATGCTATGGCATTCAGTTCA
CAAGCTACAAGGGAATATACCAGGGGAACATCACCAGATTGGTTGCATATGCAGCTTGGTG
GAGGCTTTGAAAGAACAACATAA

>SlbHLH091
ATGTCAAGCAGACGATCACGTTCCAGAATAAGCGATGATCAAATCGCTGATCTTGTTTCCAA
GTTGCAACAACTTATCCCTGAAATTCGTAATAGACGTTCTGACAAGGTTTCAGCTTCAAAAG
TGCTTCAAGAAACTTGCAACTATATAAGAAATTTACACAGAGAAGTGGATGGATTAAGTGA
GAGATTATCACAACTTTTGGAATCAACTGATAGTGATAGTGCTCAAGCTGCTATTATTAGAAG
CTTACTTATGTAG

>SlbHLH092
ATGGATGATCTTGCTCTTATCTTTTCTCATCATAATAATGGTGAGTCATCAAGAAAACAACTA
CATAAAGAAAAAGAAGATGAAAAGGCAAATTCTTTAGCTTGTATAATTTCTTTCAACAATAA
TTTTGAAAATTCTTTGTCAATCCCAAAAGATGAGGCAAATAATACTATAAGTTTCTCAAATAT
TAATAACATTATTGGTGCAAAATTTGGAGAAAGAAGGAGTGGGGAGCAAGCACTTGAACAT
TTGTTGGCTGAAAGAAAGAGAAGAAAGAGGATATCTAAGTTGTTTGTTTCCTTAGCCTCTCT
CATTCCTGGCCTCAACAAGATGGACAAGGCATCAATTCTTGAAGGAGCAGCCACTCTCATT
AGACAACTTGGTGAAAGGGCAAAAGAAGATGATCACCATCAATCTACCATAGGCATGATGA
CTAAGAATAATTTATTACCAGAAGTTGAAATAAAGAGTTTAGAAAAAGAGTTGCTCATTACA
ATATTATTATACAAAAACCAACAAAAAAGAAATATTGATGAAATATTAAGTGTGATCCAAAG

ACTTCATTTAACTATCAAGACCACCAATTTCATGCCATTTGGCACCACCTCAATGCATATCAC
AGTTATTGCTCAGATGAATGATGAGTTTTGTGAAACAACAGATTTTCTTGCTGAGAAATTAA
GATCATTGATAAGTAAAGTGTGA

>SlbHLH093
ATGCATCCACAAAGTTTCATGAACCCGGTTCACTTCCAAAGCAACTCAGAAATGTTACCATG
GTCAATTCCACCGGTTCAACCATTTATGAACCCGGTTCACCACCATGATCAGTCATTTCTCCT
CCCTCCATCACCATCGGCTTACGGTTTATTTAATCGTAACACTAACACAGATCAACAACACC
TCCGGTTCATCTCAGATAGTTTAGTAGGTCAAGTGGTTCATCATCATCATCATCATAATCAAC
CCGGCTCAATAGCACCTTTTGGTTTACAAGCTGAGTTACAAAAAATGAGTGCACAAGAAAT
AATGGATGCTAAGGCATTGGCTGCATCTAAAAGCCATAGTGAAGCTGAGAGAAGACGTAGA
GAAAGAATCAATAATCATCTTGCTAAATTGAGAAGCCTTCTTCCTAATACTACTAAAACAGA
CAAAGCTTCATTGCTAGCAGAAGTGATACAACATGTGAAAGAGTTAAAAAGACAAACATCC
TTAATATCAGAGACAAGTCTTGTCCCAACTGAAATTGATGAATTAACAGTTGATAATGCAAC
ATCTGATGAAGATGGTAAGTTTATAATAAAGGCCTCTTTGTGTTGTGAAGATAGATCTGATCT
TTTGCCTGATTTAATCAAGACATTGAAAGCACTAAGGTTAAAAACATTAAAAGCTGAAATTA
CAACACTTGGTGGACGTGTTAGAAATGTGTTGTTTATAACTGGAGACGATTATTATTGTAATA
ATAATAATAATCGAGAGGTAGATACGTGTATAAGTGGAGACGATGAAGATACTGAGATGATG
CAGCAACAACAACAACAACAACCACAGTATTGTATAAGTTCAATACAAGAAGCACTTAAAG
CTGTGATGGAGAAATCAAGTGGTGATGATTCTGCTTCTACAAGTGTTAAGAGACAAAGAAC
TAACAATATCAACATCCTTTCTTAA

>SlbHLH094
ATGGATATAGAAATCAAGAATGACAGTGAACCAGAAAAGAGGAATGATCAAGAAGTGTCAA
TGAACTATCAGTCACCAAATGTTTCTTCAGAATGGCAATTAAATGGGAGTAATTTGACTAATT
CATCAATGGGAATGGTGGATTCGTTTTGTCCTACTACTTGGGATCAGCCTACCACCAATTCAT
CAAATTTAGGCTTTTGTGATGCTAGTGTTCAAATGGATTTAGGACCTTTTAGAGCTGGTGTTG
ATAGTACACTTGGTCCTAATTGGACTCCATCAAATGCAGTGTTGAAAGGGGGTATGTTTCTA
CCTCCTGTTCCGATGATGCTTCCGCAAAGCTTAGCTCAGTTTCCGGCTGATTCGGGGTTTATT
GAAAGAGCTGCAAGGTTTTCTTGCTTTAGTGGAGGGAACTTTGGTGATATGATGAACCCTTT
TAGCATCCCCGAGTCGTCGATGAATCCTTATTATAGGGGATTGTCATCAATGCAGGGTCCTCA
AGAGGTTTTGGCAAATAATGGGTTGAAATCACCTCAGAAGCTGCAGCATTTGAGTAATGTG
GCTGAAAGTTCTAAGGATGTTTCTTTAACTCATAGGGACACACAAAGAAGTCCCCTCAAGA
ATGAGAAAAAGAGTGAAAATGTTGCTAAGTCTCAGGATGAAGCAAAAGAAGTTGCTGGAG
TCTCTGGTAATGAGTCTGATGAAGCAGGATGTAGTGGCCGTCAAGAAGAAACTGAGGGTGC
TGGTGAGGAGTCCTGTGGAAAGAATATTGGCTCAAAGAAAAGGAAAAGAGGTGGTCAGGA
TACTGAACCTGATCAAATGAAGGGAGCACAGCAGCCACCATCCGAAATTCAGAAAGGGGA
ACAGAACTTGAATCCAATTGCCAGCAAGCCTGGTGGAAAGAATGGTAAACAGGGGTCTCA
GTTTTCAGATCCAACCAAAGAAGAATATATACATGTTCGTGCTCGAAGAGGCCAGGCAACA
AATAGCCATAGTCTTGCAGAAAGAATAAGGAGGGAGAAAATCAGTGAACGGATGAAGTATC
TTCAGGATCTTGTGCCTGGTTGCAACAAGGTCACCGGCAAAGCTGTGATGCTGGATGAAAT
CATTAACTATGTACAGTCTTTGCAAAGGCAGGTTGAGTTCCTCTCAATGAAGCTTGCAACAG
TAAACCCGCGGCTTGATTTTGATATTGATGGTCTCCTGGCAAAAGATATCCTCCAGTCTCGA
GCAGGTCCTTCATCCTCACTCGCTTTTCCGCCTGATATGACCATGGCATATTCTCCTTTACATC

CACCGCAAGCTGGACTGCTTCAATCTGGTCTTCCTGGTTATGGATTTCCTAGTGAAGGATTT
CGTAGAGCCATCAATCCTCATTTAGCCACTACTAGTTGTGGGCCTGGTGACTACAAGGATCC
GTCATCTCAGGCACCGAATGAATGGGATAATGAACTACATAATATTGTCCAAATGGGGTTAA
ATTCAAGTGTACCTTCATCTAGTCAAGATTTAAGTGGTTCTCTACCAGCAGGACAAATGAAA
GCAGAACCATGA

>SlbHLH095
ATGGATCAAGGAAAGGAACTAGCTAGAACGGCAATTGCAACATCGATGCCTCTTCAGGCTA
GTATGCCAGTCCCTTTTCAGAACGATAGTGCCTTCTCTGACTCACTACCTACACCTGCTTCTG
ATGAATGCCCTAGAACCACTAATGCTCTGAATGATCAGGAGTTTATGGTAGAAGGCGGCACA
ATTAACTTTTCGAATACTTACTCTCAAGGGTTACTGAATTCGTTGACACACGCGCTACAGGC
TACTGGCCTTGATCTTTCACAGGCCAGTATATCGGTGCAGATTAATCTAGGGAAGCGAGCAA
ACAAGGAAAGGGCCTTGGGGCCATCTGTTGCTAAGGATACAGAAAATCCTCCACCTGCACC
TGGCGATCAATTTTTGGAGTTTCGAGATACAAACAATGGTGAAGAGTTGAACCAAGCTCAA
AAGAGGCTGAAGAAATAG

>SlbHLH096
ATGGAAAACATGGTTGAAGAGAATAACAATTATTTGGAAACAACCATTCTCTTTCAACAAGA
TAGCTACTTGGACGAGCCAATTATGTCGTCATACTATGATTCAACCTCGCCAGAAGGGTCAC
AATCATCGAAGAACATTGTATCAGAAAGGATTAGGAGGAACAAACTCAAAGAGAAGTTATT
TGCACTTAGAGCTCTTGTTCCAAAAATAACCAAGATGGATAAAGCCTCAATAGTGAAAGAT
GCAATTGAATATATTGTAAAACTGCAGAAGCAAGATAGGAGAATTCGAGGAGAGATATCAA
AGCTTGAATCCGAAACCTCAAACAAGAATAGCACCCATCTTCAACATGAAACCTTTGATTTT
TCAAACCCTAAAACACTTGATGAGCACCAGTATGGATATCATTCTTCCCCTATTGATGTTCTT
CAGTTGAGGGTATCTTCGATGAGAGATAGGATAGTGGTGGTGAACCTCACTTGCATCAAAA
GAAAAGATACAATGATAAAACTTTGTGATGTATTCGAATCTTTGAATATCAAAATAATTACTA
CAAATATTATTGCTTATTCTGAGACACTTCTCAACACAACTTACATTGAGGCTGATGTGGAA
GAGAGCAATCTTTTGATGTTGAGGATTCAAAGTGCTATAGCTTCTCTAAATAATTCAGATAGC
CCTCTGAGCTCTTAG

>SlbHLH097
ATGACTATGTTGTGGAGTGATGAGGATAAGACTATGGTGGCAGCGGTATTAGGAACAAAAG
CTTTTGATTACTTGATGTCTAGTTTAGTTTCTGCTGAATGTTCTTTAATGGCAATGGGGAGTG
ATGAGAATTTGCAGAATATGCTATCAGATCTTGTGGAACGTCCGAATGCAAGTAATTTTAGTT
GGAATTATGCAATTTTTTGGCAGATTTCGCGGTCTAAGTTGGGGGAGTTGGTGTTAGGATGG
GGAGATGGTTGTTGTAGGGAAGCTAGGGAAGGAGAGGAATCTGAACTTACTAGGATTCTCA
ATATACGCCTCGCGGATGAGGCTCAACAAAGGATGAGGAAGAGGGTTCTTCAGAAGTTGCA
TATGTTTTTTGGTGGAACAGATGAAGATAACTATGTGTCTGGATTGGATAAAGTCACTGATAC
TGAAATGTTCTTTCTTGCTTCAATGTACTTTTCGTTTCCTCGAGGGCAAGGTGGTCCGGGGA
AATGTTTTACTGCTGGTAAACATGTGTGGTTATCGGATGTTATGAGGTCGTCTGTAGATTATT
GTTCTAGGTCTTTTCTGATGAAGTCTGCTGGTATGCAAACAGTTGTTTTGATCCCAACTGATA
TAGGGGTTATGGAATTGGGATCTGTACGAACTATACCAGAAAGCTTGGAGCTTGTACATTCA
ATAAAATCTTGCTTCTCGTCGTTTTTGGCTCAAGTTAGGGCTAAGCAAGCGGCACCTTTAGC
AGCAGTTGTAGCTGAGAAAAAGAATGGAAACAATTCTGTGTTTCCCAGCTCTTTTCCTTTTG

ATCAGTCAAAGGAAAATCCGAAGATCTTTGGGCAGAATTTAGAATCTGGTTCTACCGAGTTT
AGGGAAAAACTTGCTCTCAGGAAACCGGTGGATGGGCCACTGGAAATGTATAGAAATGGA
AACAGGGCTCCCATCATAAATACACAAAATGGTGTACGCCCCGTATCATGGGCTTCATTTGG
TAATGTAAAGCCAGGGAACTCGGTGGATCTCTATAGTCCTCAGGCACCACCAAACAACTTA
CGAGAGTTTGTCAATGGTGGAAGAGAAGAGCTCCGGTTAAATAGCCTCCAGCATCAAAAGC
CTGGTGGAATGCAAATAGATTTCACCAACTCAAGACCCGTTGTTTCTCCAGTGCCCACTGTT
GAATCGGAGCATTCAGATGTTGAAGTTTCATGCAAGGAAAAACATGCAGGACCAGCTGATG
AAAGGAGGCCTCGAAAGCGTGGAAGAAAACCAGCCAATGGAAGGGAAGAGCCCCTCAAT
CATGTAGAAGCTGAGAGGCAACGGAGGGAGAAGCTGAACCAGCGGTTTTATGCATTACGA
GCTGTTGTTCCAAATATTTCCAAAATGGACAAAGCTTCCCTCTTAGGAGATGCCATTGCTCA
CATCACTGACATGCAGAAGAGGATCAGGGACGCGGAATATAAGCTGGAAAAGCGAGGAAG
CACCTCTGTGGATGCAGCTGATATCAACATTGAAGCCGCCAGTGATGAAGTCATCGTAAGA
GCTAGATGCCCACTGGGAACCCATCCTGTAGCAAAAGTCGTTGAAGCGTTCAAAGAGACAC
AGGTCAGCGTTGTTGAGTCAAAACTTGCCGTGGGAAATGATACAGTATATCATACATTTGTG
GTCAAGTCCAGTGGACCCGAACAGCTAACGAAGGAAAAGCTAATGGCTGCATTTGCTGGT
GAATCAAACTCTCTCTAG

>SlbHLH098
AGAAAGAGGCCAAGAATTAAGACAAGTGAGGAAGTTGAGAGCCAAAGAATGACACATATT
GCTGTGGAAAGGAATAGAAGGAAGCAAATGAATGAACATCTTCGTGTATTGAGGTCTCTCA
TGCCTGGCTCCTATGTTCAAAGGGGAGATCAAGCATCTATTATTGGTGGAGCAATTGAATTT
GTTAGAGAATTGGAACAACTCTTACAATGCCTTGAATCACAAAAAAGAAGAAGAATCTATG
GAGATACTCCAACAAGACCATTAGGAGATTCATCAACACCACCATCAATGCCAATGAATCAA
AACCCTAGTGCTATAAATCCTCATCATCATCAATCACCAATACTATTTCCTCTTCCAAATGAGT
ATAATATTGAAGATGAAATTCAAGAAGAAGTAGCTGAGAGTAAGTCTTGTTTGGCTGATGTT
GAAGTGAAACTTTTAGGGTTTGATGCAATGATCAAGATTCTATCAAGAAGAAGACCAGGAC
AACTCATAAAGGCAATTGCTGCATTGGAAGATATGCAACTTAGTATTCTTCATACAAATATTA
CAACCATTGAACAAACTGTTCTCTACTCATTCAATGTCAAGATTTCTGGTGAAACTAGATAC
ACAGCTGATGATATAGCAAACTCAATCCAACAGATATTCAGTTTCATTCATGCTGAAATAGCC
CCATATGATATCAACTAG

>SlbHLH099
ATGAATTACTGTGTTGTTCCTGATTTCAAAATGGATGATGATTATTATGATATTCCTTCTGCTAT
TTTCAACAAGAAATCCACAATAGCAGATGAAGAAATCATGGAGCTAGTATGGCAAAATGGT
GGAGTAATTATGCAAAGCCAAAATCAAAGATCTGTAAGGAAATCTAACCTTTTTCCCGAGCA
ATCAGCTGTAGAACAAACGGTAGCAGTTTCAGCTCCACTGTATATGCAAGAGGATGAAATG
AACTCATGGCTTCAATCTCCACTTGATGATTCCTCCTTCGATGATTTTCTAAATACTACACCG
AGTTGTGACGCCGTGACCTCCGCCGCTGCAGCACCGCCGGGAGAAATTGGTACTTCGAAG
GTGGAGATCCGTCCGCCTCTGGTTCCGCCGTGTTCGCGGCCGATTAGATGTACAGAGGGTG
AATTACCACATCGATTACAGAATTTTGGGCATTTTTCACGGTTGTCTGGTGAAGCGGTTTTAC
GAAATGGTACTACATCGAGTTCTGGTCACTCAGTTAGGGCATCGACGATTGTGGATTCAAAC
GAGACTCCGGTAGCAGCACGCGTATCAGAAAATGTAACACCAGTTACCGCCATGAATGTCA
GAGGAAGAGAACTGACGGCGACGTCGATGGCGACAACAAGCGGCGGTAGGGAAGTGACG
ATGGCATGCGAGTTGGCATTGACAGCATCTACACGTGGTTCAGGAGGTAGTGTAAGCGCCA

GAGCAGGACCGCCACAGCCGTCGCATACGGAGGCGGATACGGCGGCGTATGATCGGAAAC
GGAAAAGTAGAGAATCGGACGACAATGAAGGTCAGAGTGAGGATGTTGAATATGAGTTTG
CTGATGCAAGGAAGCAAGTAAGAAGTTCTACATCGGCAAAGAAATCTCGCGCAGCAGAGG
TCCATAATCTTTCTGAAAGGAAACGCCGAGACAGAATAAATGAGAAGATGAAGGCTCTACA
AGAACTCATACCTTGCTGCAATAAGTCAGACAAAGCTTCAATGCTGGATGAAGCAATTGAG
TACCTTAAATCATTGCAATTGCAAGTACAGATGATGGCCACGGGATGCAGCATGGTTCCTAT
GATGTATCCTGGAATCCCGCAATACATGCCAACTATGGGAATGAATATGAGCATGGGGATGG
ATATGAAAATGGGCAGGAACCGGCCATTAATTTCATATCCACCGCTAATGCCAGGTCCAGCA
ATGCAGAATGCAGCTGCAGCAGCACAAATGGCTCCTCAATATCCTCTTCCGGCATATCACTT
GCCACCATTTCCTGCACCTGATCCATCCAGAATCCCAGTTGCGAACCAGCCAAACTCACATG
TTGGACATAATATTAGTCAGCCAAGACTTCCCAATTTTAGTGACCCATACTATCAATATTTG
GTCTACAGCAAGCACAACTGATGTTACCCCAGAATCAGGAAGTGGAACAGCTGAGCAGCA
GTAAACTTAACAGCTGCATAGAAGGAAGTAGAGGGAATCATCAATCTGGTGAGCATACTATT
TAG

>SlbHLH100
ATGGGTATTTTTTCTCATACTCCTAACAAGGGAAAGTTTGATTTAAGCGATTCCTTCCAATGC
GATATAAGCAGACAAAGAGTCACGACTTATAACGTAAGGCATGTCAAAAGGTGCAAAAGTG
TTGTGATGAAGAGAAGGATACGCGTAGAACGTTCTAGAAGGTTCGAGGATGAGATTGGAAG
TAAAGTCAAGATACTCAAGAAGTTGATTCCAATTAACTGTGAAGATTTGGGGCTGGAAGGG
ATTTTCAGAGAGACAGCTGATTACATTTTGGCTTTAGAAATGAGAATTAAGGCTATGCAAGA
TATGGTAAATGTATTGTCACATTCTAATCACTAA

>SlbHLH101
ATGGATAATTTCCATTCAATATTTCCTTTACAACCGGATGATGATGCGAATTATGATTCCCATC
AACAACAGCTCATTTTTCCACATGAAACCTATACCAACCCTTGTAATTTCATATACCAACAAG
ATCTGGTAAATGATTTTGCTTCTTTTGATTCATTGGAGTTAAGAGACAACAATCTTACATTTA
ACAACAATACTAACAACAATCAGAAAGGGAAATCATGTGGCGTTGAGGATAAGAAGAAAC
ACAAGAAAGTGATGCATAGAGAAATTGAAAGGCAAAGAAGACAAGAAATGTCTACTCTTTA
TGCTTCTCTTCGTCAACAACTTCCTCTTGAAAATATTAAGGGGAAGCGTTCCACATCAGATC
ACATACTTGAGGCAGCGAATTACATAGAACAACTACAAAAGAATGTTAAGAATTTGGAAGA
AAAGAGAGAAAAGTTGATGAAAGATTCAACTGGTTTAAGCAATGTTGATGGCATTAACAAA
AGTGGAAGATCAACATCTAGACACTGCTCCCCACCTGCTATTGTGACAGTGAAAGAATGCT
TGGATGGGATGGAGATTTTGGTTAATTGTGGTCTCGGGTTCCGCCTTTCTAGGGTTCTTCAA
GTGTTACTTCAAGAAGGGCTTAGTATAGTGAACTGCAGCTCTACAAAAACAAATACTTCAA
GTCTACTTCACACTATTCGAACTGAGGTTTCCAGTACTGATCAGCCAAGGATTATTAATGTG
GACGTAATCCAACAAAAGCTCACAGATATTATTCATGATGATACAAGTTTCAAGTGA

>SlbHLH102
ATGGTATCAAGGGAGCAGAAAAGAGCCTCAGTATTGCATGACAAATTGCAACTTCTTCGAT
CAATCACTAAATCTCATGCTCTTAGTGAAAGCTCAATCATTGTAGATGCATCAAAATATATTC
AAGAACTCAAACACAAAGTGGAAAGGCTAAATCAAGACATAACTACTACTACCCCTCAAAC
TAATTCAAACAACAACACTTCTTCATGGCCACAAATTGAAGTGGAAACCCTAGAAAAAGGT
TTCTTAGTAAATGTATATTCAGAAAGAAGTTGCCCTGGTTTGCTTGTTTCCATATTACAAGTT

CTTGAAGACCTTGGCCTTAACGTACTTGAAGCTAGGGTTTCTTGCACCGACACCTTTCGTTT
ACAAGCATTTGGTGGAGAGGATGAAGAAACCATGATTAATGCCCAAGTGGTGACAGAGGC
AGTTTTTGAAGCTATTAAGAACTGGAGTGAAAGCAAAGAACAAGGCTAATTAATCAAATATA
TAATATAGTTTAATTAGCTATTATTAATTATTACTATATTCACTCTTTCGTTTTTCTCTTTAATTTT
AACTTTCCCGATCATCTTCCTAGCTAGCTATGATAGTTATATTAATTATCGATAA

>SlbHLH103
ATGATGATAAGTGATGAGGCCAAATATGATTTTTCATTTGACCAAATAAAGTTTTCTGCTTCT
AGGAGCCATAGTGAAGCTGAGAAAAGGCGCAGAGACAGAATTAATGCTCAGCTTTCTACTC
TTAGAAAACTCATTCCCACTTCTGAAAAGATGGATAAGGCAGGTCTACTAAGAAGTGTTGTT
GAGCATGTTAAAGATCTGGAAGGAAAAGCAAAAGAAATGAGCAATGTATTGAACACTCCA
AGTGACATTGATGAAGTAGTAATTGAGGAAGAAGATGAAAGTTCCAACAACAACAACATAG
TTGTGAAGGTTTCTTTTTCGTGTGATGATCGGCCTGAATTGTTCTCAGAACTAAACAGGGGT
CTCAAGAACCTGAAACTAACAACAATGGAGGCTAAAATAACTAGTTTGGGTGGCAGAATTA
AATGCATTTTATCCCTTCAATCCATTAATGTTGTTTGCACTACTCATTCTATCAAACACTCTCT
TAGGCTGTTGCTTGCTAGGATTGCTACTTCCCCTTCTACTTCCAATTTTCGAATCAAAAGCAA
GAGGCAGAGGTTCTTCTTGCTTGCTACCTGA

>SlbHLH104
CAAAATAAAAAAAAAATGGAGAGTGGTAATGCATCAATGGAAAATAATAATGTTAATGATAT
TGGGCTTATTAATTTCTTGGATGAGGATAATTTTGAACAATTCATTGAGCTTATTAGGGGTGA
AACTGCTGATCCTATTGTAAATTTTTGCCCAAACTATGATTGTGAACATATGACAGGTTGTTT
TTCCGCAGCCAATGCCCAATTTGAGCCAATATTATCGTCGATGGATTTCTATGATACAACATT
GCCAGATCCTATTTCGCTATATAACTGCGAAATCAAGCTGGATAATAATGATGATGAAGACGA
TGATGAATCTTCTGGCACAACGGCTACCACCAAAATGACTCCAACAAGCAAAGGCACGAG
GACTGACCGGTCCAGAACTTTGATTTCGGAGCGCAAAAGGAGAGGAAGAATGAAGGAAA
AGCTTTACGCTTTGCGATCCTTAGTTCCTAATATCACAAAGATGGATAAAGCCTCCATCATTG
GAGATGCAATATTATACGTACAAGGACTACAAACCAAAGCAAAGAAACTTAAAGTTGAAAT
AGCAGAGTTTGAGTCATCAAGTGGAATATTTCAAAATGCAAAGAAAATGAATTTCACAACC
TATTATCCAGCAATCAAGAGGATAACAAAGATGGACATTAATCAAGTAGAAGAAAAAGGAT
TTTACGTGAGATTAATTTGCAACAAAGGGCGACACATTGCAGCTTCTCTTTTCAAAGCTCTT
GAGTCTCTCAATGGATTCAATGTTCAAACTTCCAACTTGGCTACTTCTACCAATGATTACATT
TTCACCTTCACTCTTTATGTGAGAGAATGTCATGAGGTGGACATAAACTTTGGCAATTTGAA
GCTATGGATAGCTAGTGCTTTTCTTAATCAAGGGTTTGACTTCGAGACATCTCCGTTGGTCTA
A

>SlbHLH105
ATGAATTTTCAGGAATTTGGAGCTGCATTTGATCCAATCGACATCCTTTCTAGTTGGAATATC
TCTCAGCGTCAAGAAGCTGCGACGAGATTGGCTGCGGACTCTGTTGCTGCTAAATCAGGAG
CAGGTTCAATTGGGGATTGTTCTAAACGTAAAATTGACTCCTCTTCTTCTAATTTTCCTCCAG
GGAACAATTATTTGCCTTATTTCGGTAATAATCAAGTCCCAGTTTGTAGCCTAATGGCGGATC
TTAGTTCTATTTCCTTATTCTCTAAAACCAAATCCCCTAATTGCTTGCTCTCTGCAACAAATAC
AAGCAACACCGACACATCAGGCGAATTAGATGATGTGATTTTTTCTGATGATTCTGAAAGCT
TGTGGAATATCCACACTAGCAATGTAGTTTCATCTGGAGAATCTGCAATTGATGCCTACAAAT

CGAAACCCATTGATGCCGATGACAACAACGATATTCACTACTCTGTTAATCAGCTTCATGCA
ACAGTCTCGTGTAACAATTTGATAAGATCTTCAGAAGCAAAATATTACAATATGGACAAGAG
AAGCCACGATGCTCTGCTCGAATCAGATTCCTCGAACAGAGACTATGTAATCCAGCTCATTT
CGGAAAATGATCAACCTAAGTCCAAGAAATCCAGATCAGAATACAAGCTTCCAAGTTCATC
AAACATCAATTTCCAGCAAGCAAGTTCTTCGGCTTGTTCAATAGTTGATCGTGATTCAGAAG
CAATTGCACAAATGAAGGAAATGATTTATCGCGCAGCAGCATTCAGGCCAGTGAGCTCTGT
CACGGAAGATGTGATGGTAGAGAAGCCGAAGAGGAAGAACGTAAGAATATCAATGGATCC
ACAAACAGCTGCAGCGAGACGAAGGAGGGAAAAGATAAGCGAAAGAATAAGGATATTGCA
GAAGTTGGTACCAGGAGGAAGCAATATGGACACTGCATCCATGCTTGATGAAGCTGCAAAC
TATCTCAAGTTCTTAAGGACACAAGTGAATGCCTTTGAATCCTTTGGCTTTAATATAGACCCA
AGTATCACTAACAATAACTTTACTACTTCTTTATCTTCAATACCATTAATCAACTACCCATTTC
CCTTGCAACCACATTTTCCTATGCAAAACCTCAATCCAGTCCATCACCCTAAGTGTTGA

>SlbHLH106
ATGGAAAATACAACTTTCAATAACAACATAATTGGTGATGAAGAAGCACCATTTGAATTTAA
GAATCTACTATCTGAAATGTTCACATCTTTACCTAATCAAAATCCCACTACCTCCAATTCATTA
GAAAATGTTCCTAAAATTGCATTTTCTTGTTCTTCTCCTTCTTCTAATAATTCATCGTCTTCAT
CGCAAAATATTATTTCATTTGGGAATAAAGCTGATTCTTTTTTCCTAGAGGATAATGAATTAG
ATTATGATATGATAACGGAGAAAGTGATAATGAGCAACGTCAACATATCGAATTCATCGATGG
CATCTAAGAGGATATGTAGAAGTCCTTTGCAATCTCAAGATCATCTTTTGGCTGAAAGGAAA
CGTCGTGAAAGGTTCTCCCAACTTTTTGCTCTCTTGGCTAAAGCTATACCGCAACTCAAGAA
GTTGGACAAAGCTTCAATTCTTGAAGATGCTATAAAGTACATAGGAGAACTTCAAGAACGT
GTTAGTTCTTTAGAGGAAGCTGCTCGGACGATAAAAAGTAGTACAAACTTGATTGAATCAA
CTCATCCCACACTAGTACACAAACAATATTCTCATGATGATCATGTTGATGATCTTGAATCCA
AAAGACATGAGAATATTAATGATATAAAGGTTCAAATTCTGGACAAAAATGTACTCATAGGA
ATCCATTGCAATAAGCAAATGAGGAGTATTTTCTCTATTATTGCTGGAATAATGGAAAAGCTA
CATCTTACTATTCACCACATAAGAGTTTCCCCATCCAATCATACTTCTCTTCATTATATCTCCAT
TCTTGCTGAGATTGATGAGAATGTTGATATTAAAGTGCAGGATGTTGAAAAGGCGTTTGAAT
TACATCTTCTTACCATACAAGACTCGACACAAGAATAA

>SlbHLH107
ATGGAAAGTAATTATTACGATTCTTTTCAAGGCATGGAATATAGTGGTAATTATAACTATGAA
GATGAGAGCTTCTTTGAATTCAAGCCAAATATTTCTCAAGGTATCTATGATTTATCAAGTGCT
TACAACACAAATATTGAAGAAAAAAGTGGTCAAAAGTCAATTTCTTCTAATAATTCTTCCTC
TAATTCTGGGGGATTTCTCATTTCATTTTCTTCAAATCAAGAAGAAGATATTGGTGCAATGAT
AAGCTCAGAAAATTCATGCCAAGAATCATTTTTATTAGGGGAAAATAATAATAACAATAATAA
TAATAATAATGTTATGTATAAGAGGAGTCCACAACAAGCACAAGATCATGTTATTGCAGAGA
GGAAGAGGAGAGAAAAAATGGGAGACCTTTTCATCTCTTTGTCAAAGATTGTACCTGGTCT
TAAGAAGTTAGATAAATCATCTATCCTTGGAGATACGATTGAGTACATGAAAGAGCTTCAAG
AACAAGTAAAACTTCTTGAAGAGTCAAAGAAAAACACATCATCATCTTTGGAACATAATGA
TTCTAACAAGGAACAAGTATTAGGATCAAACAAGATTAAGGTAAGGATTATGGACAAAAAT
GTACTTATTAACATACATTGCAACAAACAAGATGGAATGTTGGGAAGATTGCTAGTCCAAAT
GGAGCAATTGCATCTTTCAGTCCATGACATGAGGATTATGCCTTTTGGTCCTACTAATCTTGA
AATATCACTTCTTGCTCAGATGGAGGATGGATGCTGCATAAATGTAGAGGATATTGTAAAGG

CCATCCAAATCAACATCCTGGACCTCGTAAATAATTAA

>SlbHLH108
ATGCAACGTGGAACTACCGGTGACGGGGGTGGTGGACTTTCCCGTTTCCGGTCAGCTCCGG
CGACATGGTTAGAAGCTCTACTTGAATCCGACACTGAGAGCGAAGTTATTCTTAACCCTTCT
TCTCCCATTTTACACACTCCCAATAAACCACCACCTCACCCATCAACTCCGAAGCTTAAGCT
GGAAACCGGCGGAGCTACTAGGTTCACTGGCGATCCGGGTCTGTTTGAATCCGGTGGTAGT
AGCAATTTTCTCAGGCAGAATAGTTCTCCGGCGGAGTTTCTTTCACATATCAGCTCTGATGG
TTACTTCTCAAACTATGGAATTCCGTCCAGTTTGGACTATCTTTCTCCATCCGTTGATGTTTC
GCAGTCCGCTAAGCGAACCCGAGACGATGATTCCGAAAGTTCCCCAGAAAATTAG

>SlbHLH109
ATGGCTGGAAACCCTACTAATTGGTGGAGCATGATCATGAATGGAAACATGCATCCACAATT
ATCTCATGATCATGATCAACATAATTCTTCTTCTTCTAATTCTAATTCTAATTCTTCACATTTTT
ATGGAGCTCATAATCCAAATCAAGATTTTCCTCGTTCATTGAGCCAACTTCTTATGAATGGAT
TTTGTGGTGATGAAGAAAAGTTTGGTATTAGTCCTTTTCATGCCGCCGGGAATTCGGAAGAC
CAAAGTTTGAATTCCGTCCATCCAACATCATCAACAGCTAATTTTAGGGTTCCTGTTGCTGAT
GTAAAGCCAGAATTGTATCATCATCATCATGGCCACAACGAATTTCAAACATCTTCATCGCTA
CAAGAAAAACTCGAATTAGATACTGATCATTTTACCAGGCCTTTAATTGCTTCTAATTGGTCT
CAACAAGATTTGTCTTCACCAGCTAGCTCTTGTATTACAACTGGGTTAAGTCACAACCTTTT
GAATTTCTCTAACAACAAAGGGGAACATAAGCATCAACATCCAGATCATAATTCAACTGAGT
GTAACAGCACAAGCAGTGGTGGGATTAGCAAGAAGGCTAGGGTTCAACAATCTTCAGCTC
AACCATCTCTAAAGGTAAGAAAGGAGAAGCTAGGAGATAGAGTAACATCACTCCACCAACT
TGTTTCTCCATTTGGAAAGACTGATACAGCCTCCGTCCTATCAGAAGCTATTGGCTACATCA
GATTCCTTCAGGCTCAAATTCAGGCATTAAGCTCACCATACTTGGGCAATGCATCAGGAAGC
ATGGGTCACATTCCCCAACAATCACTGAATTTTTTGGATGGTAATTTTCTGAAAAGGAGACC
AGCAAATAAGCAGGATTCTCAGCACAAACCGATGGATTTAAGGAGTAGAGGGTTGTGCTTG
GTTCCTATTTCTTGCATGGCAAATATAGGAAGTGATACCGGCGCCGATTATTGGGCTCCAGCT
CTCGGAGGAGGATTTTGA

>SlbHLH110
ATGCTCTCAAGAGTTAACAGTATGCAGGTTTGGGATATGGAAGGCAAACAAGAAGAAGAA
AAAGAAGAAAATTTTAGCAACAAGGAGAATACCACTAATGTTGAGCTTGAAAACAAACAA
GATATGGAATTGGGTGCACTTTCTACATTCAAATCTATGTTAGATGGTACAGATGTAGATTGG
TATCACAACAACATGCAAAACCATACTGAAAATATCTGTTTCACTCAGAATTTCACTGAATT
AGCTGAAAACAGCATGTTTCTACAGCCAGTAGTACCAGTGGATTCTTCATCTTCTTGTTCAC
CATCTTCAGTTTCTGTTTTCAACAATCTAGACCCATCTCAGGTACATTACTTGTTAGCCCAAA
AGGCTATTAACAACAACCCTTTGGATTATAGTTTTAATTTGGGTTGTGAAAATGGTTTTCTTG
AAGCTCAAGGTATGGGTGGTTTGAATAAAGGGGGATTTCTTCTTGCTGGTGGTGGGTTTCAT
GATTTGAGCTCACAGAACCAAATGGGTAACCCCAATTTGAATTCATTTACTCAATACCCATC
ATCCCATTTGCCCCAAAATACTACTACTACTGGGTTTAGTCCACTAGGGTTTGTTGATGGTTC
TGCAAATGAGAACTCTCTGTTTCTCAATAGGTCCAAGTTGTTAAAGCCACTTGATAATTTTG
CTTCAAATGGAGCACAACCAACCCTTTTTCAGAAAAGGGCTGCTCTTAGGAAGAACTTAGC
TAATACTACTGGTGGCAGTTTGGGGGATTTCGGAGGTGAAATTGGCCAGAATTCCATGAATG

GAGAGAATGAAAGGAAGAGAAAGTGGGGTAGTGGGGAAGAACTTGATGATGTGAGCTTTG
ATGGGTGCACATTGAGTTATGATTCAGATGACCTTACTGAAAATGTTACTAATAAGGTGGAT
GATACTGTTAAGAATGGTGGAAATAGCTCAAATGCAACTAGCACTGTCACCTGTGGGAATC
AGAAAGGAAAGAAAAAGGGACTTCCGGCTAAGAATTTGATGGCTGAACGACGACGTAGGA
AGAAGCTAAACGATAGGCTTTACATGTTGAGGTCTGTTGTACCAAGGATTAGCAAGATGGA
CAGGGCTTCAATCTTAGGGGATGCAATTGAGTACTTAAAGGAGCTTTTACAGAAAATCAATG
ACCTGCATAATGAACTGGAGTCCACGCCACCTAGCTCTTCACTGACCCAAACAACAAGCTT
CTATCCTCTAACACCAACTGGACCAGCTCTGCCAGGCCGCATCAAGGAAGAACTCTATCCG
AGTTCATTTGCAAGCCCACTGTCTAGCCCGACTGGACAACCTGCAAGGGTTGAAGTAAAGG
CAAGAGAAGGAAGAGCTGTGAATATCCATATGTTTTGCAGCCGCAGACCAGGCCTCTTACT
CTCAACAATGAGGGCACTCGACAACCTCGGACTGGACATCCAACAAGCTGTTATCAGCTGC
TTCAATGGATTTGCCTTAGATATCTTTAGAGCTGAGCAATGCAAAGAAGGTCAAGACTTTCA
TCCTGATCAAATCAAAGCAGTACTGCTGGATTCCGCTGGTTGCCATGGGATGATATGA

>SlbHLH111
ATGGAGTTACCTCAATCCAGGCCTTTTGGAACAGAAGGGAGGAAGACGACGCATGATTTTC
TTTCACTATATTCACCAATTGAACAAGATCCAAGACCTTCTCAAGGTGGCCACCTACAAACT
CATGATTTCTTTCAACCTCTAGAACAAGCAAGGAAGACTGTTGGGAAAGAAGAAAATAAA
GTTGAGGTAGAGGCTATAGAAAAGCAGCCTCCATCAGCAGCACATATTCTTCCTGGTGGCAT
TGGCACATATAGCATTTCTTATTCACAGCAAAGGTTTCCAAAGCCAGAAGCAAACACATTTG
CTGTTACACAAACTAGCAGTACAGATAGAGACGATAGAAACTCAAACTGCAGTTCTTACTC
AGGAAGTGGTTTTGCATTATGGAATGAATCTGCCATGAAGAAGGGAAAGACTGGGAAGGA
GAATCTCGCTGGAGATAGACATGTCGTAAGAGAAGCAGGTTTAAACATTGGAGGAGGGAAA
TGTACGACATCATTAGAACGGCAATCACAATTGTCTTCAAATCATAATCATAACACTGCAAC
CTTGAGCTCACACTCATCTCCTCAGCAACCATCAGCAATGGAGAATCAGAGTTTCATACATA
TGATAACATCGGCTAAGAATGCACAGGAAGATGATGATGACGACGAAGAAGAATTTGTTGT
TAAGAAAGAGTCACCTTCACCATCCAGAGGCAATTTATCAGTAAAAGTCGATGGGAAAAGC
AGTGATCAGAAGCCAAATACTCCACGCTCGAAGCACTCAGCAACAGAGCAAAGAAGAAGA
AGCAAGATCAATGACAGATTTCTGAAGTTGAGAGAGATCATCCCTCACAGTGACCAGAAGA
GAGATAAAGCATCATTCTTGCTGGAGGTTATCGAATATATTCAATTCCTACAAGAGAAAGTG
CACAAATATGAGGGATCCTACCAAAGTTTGGATAGTCAACCTTCAACATTACCTTGGAATAA
GTGCCATAGTATGGCTCAAGGTTTCATTGATCATTCTCAAGGCACAAACAGTGCATCTAGTC
CTGCACTTATACGTGCTGCAAAGTTTGACGAGAACAAAATTGGAATCTCTGCTACAGGGCC
TGTCACTGAACAGACACAAGAGCCAAACAGAACTTCCAGTGTGAAAGAAAGCAGTTTTCT
GTCTGAATTGACAAACAGGGCCGCAACACTTTGTATGCAACCAAGCACATTTCCGTTTGGT
GGACATACTAGCATAGCATCGCTAAAGTCAATACTGGCACCTGATGCTGGCTCGTTGGAACT
GAAATCTCAGCCTCAATGTCCATCAAACAGATTGAATATGGCTAATTATGCTGTTACAAATGA
CAAGCTAAAAGGTCAAGAGGTTTCTACAGAAAGTGGTACGCTCAGCATCTCCAGTGTCTAT
TCTCAAAGATTGATGAACACACTGAAACAAGCATTGCAGAGTTCCGGAGTAGACTTGTCAC
AAGCCAACATGTCAGTGCAAATTGATCTCGCCAAAAGAGCTGATGATAGATCAAATGCTTC
AACATCCAATTTTAAGGGTGACAACATTTCCTCAAGAAATCAACCAACTCCACTATTCATTG
ATACAAGCACAAGAGAGGAGTCTGTCCATGCCTTCAACGGCTCAAAACAAGTTAA

>SlbHLH112

ATGAGTCATCATACTTGGAATTTTAGTCACCAAAAACAAGAACAACAAGTAGTAGAAAAAG
AAGAAGAGGAGAACAGATATACTCGTGGCCACGTGCATAATCAGCAGAATCAAGTTGACCC
CATGTCCAATAAGTGTGAAGTTGCAGAGTTAACATGGGAAAATGGGCAAGTAGCCATGCAT
AGACTCGGAAGCAACCTCTCGAATGAGCAAACAAAACACACATGGGGAAAGGCTGGTGAC
ACACTAGAGTCAATTGTGCATCAAGCCACTTTCCAAAAACAACATCATAGTTATATAATGGG
AAGTGATGGACAAAATCAGGCAAACATCAACAGGGAAAAAAATGTATCTTATGGTGCTCAA
CAAACAAGAGGAGTATTAAAGAGGATGAGATCATCAGATTCTGACCCCCAGTTATATATTGG
TGGAATATCATTAGAACATTTAAACGCTCGTGCAAGTGCTAAGGACAATGATATCACCATGAT
AACATGGCCTTGTAATGAGGATTCTGCCTGCCATGGTGGCTCGGAAAACAAAGAGGAAGA
ACGTGAAACAAAAAGCTCCAACCCATCAAAACGTAGTCGAAGAGCTGCTGTCCACAACCA
ATCAGAGCGGAGACGCCGTGACAGAATCAATGAAAAGATGAAAGCTCTGCAGAAGTTGGT
GCCAAATGCTAGTAAGACAAATAAAGCATCCATGCTGGAGGAAGTGATAAAGTACTTAAAA
CAGCTTCAAGCACAAATCCAGTTGATAAGCTATGCTAAAAACATGGAGCAGCAAATGATGA
TGATGTCTCTAGGAATGCAACCAGCACATATTCAAATGCCTTTACTAGCAACAATGGGCATG
TGTAGTAGTACTACAGGAATACTTAACAATATGACTTCTAATTTAGCTCCAGCTCCTTATCAA
TCTCTTATAGGCGGCCGCGCTCCCCTCATTTATCCAACCTCTTCAATGCCCACTCTTTTTCCT
CCCTTCATGTCACCACCCTTCGCCACGGCATCATCCATTCCTAGTACTCCACCACAACCTATT
AACGCCGAATCCATCAGCCCCAAACTCACTAAATATGCAGCTCCCCCTAATATTGCAGCCAG
TACTTCATTCCCTTTTAGCCACCCCTATAATGCATATCTGCCTCATTCAATGAAGATGGAGTTC
AATAATGAGATGGCAGCTCAATATCTGCAGCGGGGCAATCAAGAAAATGTCAATATTCAAGG
GCAAAAAAAATGA

>SlbHLH113
ATGCACCAACGCAAGTTGTTTTGATAGATTCTTGACTCAAGGAAAAGTGAATAAAAGTAGA
TCGAAATAAATAAATAATGGAAGTGAAAATTAGATCATGTCAAAATATTATAGAAAACATGA
CAAAACATTCTGAAAAAAAGATGTGCAATAATAGAAGTACTAGAGACACCGTAGAAATTGA
AGCCTACGTAATAAGACTGCGGACTACTAGTATAGAAAGATAATTAACACAATGCTTTAGCT
TTACTGCCTACTAATTTTCTATCCTAATTTGTGTTTTTCACAACCTTGTATTTAAGGTCACGTC
CTCAATAAGCTGAAAATACGTCATGTCATATCTAATCGCTAGTCCAAGGCTCCCAGCTTACC
AATCATGTAATAAAAAGAGAGCATTCTATCTAA

>SlbHLH114
ATGGAAGCTAATAGTAACTCTTTTCATGTAGATTCTGTTTTTCATGTGCCCATTAAGATGTCT
GGTTTTTTTGAGGAACCAAACAATAATATAACAAGTAGTAGTACACTACCAAATTGTGTTTC
TCAATTTTATTTGCAAGAGCTTTCTGTCAATATGAGTAATAATGTTCATGAAATTAGCCATAAT
GAACCTTCTCATGTGACAAACAAAACCAATTCTTCCTCCCTCTGCTCTACTCAATCTAAGAA
TGTAAGAGACGGTGATGATGGGAAAGGGCAAAAGAAAAGAAATGGTAATGTAAAAAGAGA
GAAAAAAACAAAGGAAAATAAGAAGAAAGCCCCTGAAGAGGCCCCTACAGGATATGTTCA
TGTTAGAGCAAGGAGGGGCCAGGCAACTGACAGTCACAGTCTTGCTGAAAGGGTGAGGA
GAGAGAAAATAAGTGAAAGGATGAAGATATTGCAAGCACTTGTTCCAGGTTGTGACAAGGT
AACTGGGAAGGCCCTTATGTTGGATGAGATAATCAACTATGTCCAGTCTTTGCAAAACCAAG
TTGAGTTTTTATCCATGAAGCTTGCTTCTTTGAACCCCATGTACTATGACTTTGGCATGGACT
TAGATGCTCTCATGGTCAAACCTGATCAGAGTTGGAGTGGCTTGGAAGGACCATTATTAGAG
AACACAACTAGTAACTACCCTCACTTGGATAGTTCAACATCACTTATGTTTCAACAATTGCAT

CTACCAAATTCCGTTTCTCAGGGTAGTGGACATGTATTATGGAGTGTAGATGACCAAAGACA
AAAGATGATTATTAATCATTCAGAATTGATCAGTAATAACAACAACTTGAGTGTCCCTTTCCA
TTAA

>SlbHLH115
ATGGGGTACTTGCTGAAAGAGGTTTTGAAAACTCTTTGTGGAGTAAACCAGTGGTCTTATG
CTGTTTTCTGGAAGATAGGTTGCCAAAACACCAAGATTTAATTTGGGAAGAATCTTATTAT
GAAACATCAACATTGTCTAATATTCATGGAACTTCTGGAGTTGAGAATCCAGAACTAGCTTT
CCAAGATTGGAGCACTGGTTGGGCTTTTGGTGGAGTTCAAAATTCTCAGCTTCAGAATCAA
GCAGGGGAGAACTTGCATTTGCTTATAAACAAAATGATGATGGACAATCAGTTCAATCTAGT
AGGAGAAGGACTAATTGGCCGGGCTGCAGTTACTGGGAAACATCAGTGGGTTCTTTCGGAG
GGTTTAAGTAGAAATGTTCATCCACCAGAGGTTTTGAGAGAACTTCGCCAACAATTTTCAG
CTGGCATACAGACAATTTCAGTTATTCCTGTTCTTCCTCATGGCGTTGTTCAATTTGGTTCATA
TTTGCACATAATGGAGAATATGGGATTTGTGGAGGATGTGAAAACACTAATGAGTCAACTAG
GATGTGTCCCTGGTGTTTTATTATCTGATGAGAATGCAACAAAAGAACCAGCACTAGAAACT
TCCAGATCAGTTTACCTTGGGAGTTCAGTCTCAACGGAATATTGTGGGAGAGCCAAAGTAA
TGAACTCTGCTTCAATAATTGACAAGGGTAACTCAATTCAGACTGAGGGTTTTGTTGGTCAA
ACTTCTTTTTCTTTGGTTGATGCAACGTTTCAAGACTCCAATTTCACTCAAACTTTTGCTGAC
TGCCATGATAACCACTTGCACAAGAAAATTTCGCCACAAGTGAAACCATGCATGTATATGAA
CAACCAGCTGACAAATAGTGTCATCAAAACTGAGGTAATTCCTCCAAACACCGACATGTGG
AAGAAGCAACAAGATTCACAGTACATTCCAAAGCCACCGTTCTGCCAGGAATCTTCTGTTG
GCTCATTACCTCTAGATAGTGACAGCATAATGTTAACTGAACAACAGATCTCTGGTGAAAAT
AGTCTTGCAAAAAGCAATTTAACTCTACCAAATTTCTTAGGATCATCTCATGGAAGATCCCA
TCATGCAGTGATGTACAAGTCAATTCCACATCCCAATTTTATTGCAGATGCCAGCAGACCAC
CACAGAAAATCATATCTTGCACAGAGCATATTGGAGATGGGCTTCAAATAGGTTCGTCTGAT
TTGATGGCATCTTCTAAATATGATGTGAACCATGTGATCAATAATCATTCTTTGGATGGACAA
GGTGCAGAATATTTGTTGGATGGGAGCAAGAGAATGGTAGAAAATGATTTGTTTCAAGCAC
TTGGCCCTATATTAACACAGAATGAGAATCCAAGTTCAAGTGAGTGCATTCAGGATTTTTATA
GTGAAAAAATTGAACACGGGGCGCGGTTTCCATTATTTGACAGTGCATATGGAGATGTGCAT
GTCCAATGTCAGTCTGGGGATGACTTGTTTGATGTTTTGGGTGCTGATTTTAAGAAAAATCA
TCTAAATGGCAGCTGGAACAATGGCCAGTGCAAAGAACCAAACTCCAACACAAAGGATTG
GATTAAAAATAGCTCGACTTCTACAATAAGTCAGGATGCTTCTTCTACCATCAACCAGGGAA
ACTCAGATAGTTGTATGTTCTCCATGACTGGCTTTGACCGTATTTTAGATACTATGGTATCAA
GTCATTCTGCTAAGCAGAGCCTGGATGACAATGTTTCTAGCCGTACGACTATAACAAATTTG
AGTAGCTCTTCTGCCCCTAATGCTTCATGCTCCTATGATCGAGTTGGGGTCTCCAGTCAAATT
CAGGGAGAGCAGTTTGTGTCTCCCAAGACACTTTTAAAATCAGGAGCAATAAGTTCTTCAT
ACAAATCTGAATGCTCCAAAGAAGACACAGGAATGTACTCTCAAAGTAGTTCAATTTATGG
ATCAACGATCAGTTCATGGGTGGAAAGTGGTTATGACACGAAGCCATCAAGCAGTGTTTCA
ACAGGCTATTCTAAAAAGCCAGACGAAATGAGCAAAACAAGTCGCAAAAGGCTTAAACCA
GGAGAGAATCCTAGGCCAAGGCCAAAAGATCGGCAAATGATCCAAGATCGTGTGAAGGAA
CTTCGAGAAATTGTGCCTAATGGGGCAAAGTGTAGCATTGATGCACTGTTCGAACGCACGA
TCAAACACATGCTTTTCTTGCAAAGTGTCACAAAACATGCAGACAAACTAAAACAGACTGG
AGAGTCAAAGATTATCAGTAAGGAAGGAGGATTGCTTTTAAAGGATAATCTTGAAGGTGGA
GCAACATGGGCATATGAAGTAGGCTCACAGTCTATGGTCTGCCCTATTATAGTTGAGGATCT

GAATCAACCTCGTCAAATGCTTGTGGAGATGCTTTGCGAGGAACGGGGCTTATTTTTGGAA
ATAGCTGACATTATAAGAGGTTTGGGCTTGACAATCCTGAAGGGTGTGATGGAAACAAGGA
ACGACAAAATATGGGCACAATTTGCTGTAGAGGCAAACAGAGATGTCACAAGGATGGAGAT
ATTCATCTCACTTGTTCACCTCTTGGAGCAAACAGCAAAAGGTGGAACAGAACCTGTCAAT
GCTGCTGATAACAACACAGCAATGGTGCATTCATACCACCAAGCAGCGGCAAAACCTGCAA
CTGGTAGATCTTGTAGTTTGCTGTGA

>SlbHLH116
ATGGATAATGACTGCTTCTCTAATGGTGGAATTCAACCACCATTCCATTTGACCCCAAAATT
CCATTAAACTCTCTACATTCACCTCATTCAGATTACTTTCTAAACACCCATTGGGATAATAATT
CTACAGATAATCAATACACTCATTTTGATTCAGCTTTGAGTTCGATAGTTTCATCTCCAGTAC
CCTCCAACTCTGTAAATTCCAATTCATCACTCTGTGAATTGATTGGGAAATTGGGTTCAATTT
GTACTTCTCCTTCAACCCCATTTACTAGTAACTGTGATTCGACGAGAACTTCGTGTTATACTA
CACCCATGAGCTCTCCTCCGAAATTACATATACCCATAATGAACCAAATTGGAAAAGATAAA
GTACCCAATTTGGGGAATTCAGTTGTTATGAATTCTCCTCCATTTCCCTCTCTTTCAGCTGCT
AAGTTCTCTTGTTTTGGTAGCCGAAGTTTTAATGGCAGAACGAGTCAGTTTGAGCTGAATAA
TGAAGATTCGCGGTATGGATCTGGTACTGGGGTTATGGGGATAGGAAATTTAACTAGAATAT
CGAGTAGCCCTTGTGTTGTGCAAAACAAAAACTCAAGCCTAATGATGTGTGAGAGATTGAA
TTTGGGCAAAATTTCAGGTCGTAATGAAGAGTGTTCTGTTTCTGAGCAAGACCCAAATGGT
GAAATGGGGTCAAAAACAAGAAATGTATTGAATTCTAAGAAAAGAAAAGCAGTGAAATCA
AAGGATTTTGTGCCAATTGTAGATGAAACTGGAAAAAAGAGAGCAAAATCAACACAAGGT
AATGGGAGCAACAATGGAACTGTTAAAATGGAGGAACAAAAGGGCAATGAAGATGATGGA
GCTGAGAAAGAAACAAAGGAAAATCGAAAGATTGCAGAGCCACCAAAGGATTACATTCAT
GTTAGAGCAAGAAGGGGTCAAGCTACTGATAGCCATAGTTTAGCTGAAAGAGTCCGCAGAG
AAAAGATCAGTCAAAGAATGAAGCTTTTGCAAGATCTTGTACCAGGCTGTAACAAGGTGAC
TGGAAAAGCATTGATGCTCGACGAAATTATAAATTACGTCCGGTCACTCCAACACCAAGTTG
AGTTCTTGTCTATGAAGTTGGCTTCAGTGAACCCAAGAACGGATATTCACATCGATAGTCTC
CTTCACACAGAAATAAGTCAACCAAGTGGTTCTTTGCACCAACATGTTTTCCCAGTAGATGG
ATATGCTGAAAATCTTGCTCAGCTTCCCACAATCTGTGAGGATGATCTCCAAAGCATTGTCC
AGATGGGATTTAATCAGAACTCTAACCAAGATCTGATATTGCAGTCACAGACATTCCCTGTG
CCTAATTCTGAATCTCAAATGAAAATTAAGATGTAA

>SlbHLH117
ATGGCAGAGAAATTTTTTCTGAAGGGAGAGGATAAGGTTAATATGGAGGGGGTATTAGGCA
GCGAAGCAGTAGAATTTTTCTCTTGGTCAGCTTCAAATCACATGCTGACAGAATTTACTTCA
TCAAGGGGGGATTTGGGAGTGCAGCAGGCGCTCTGCAAGATTGTTGAGGGGTCTGATTGG
ACTTATGCAATCTATTGGCAAGTTGCAAAGTCGAAATCTGGAAAATCAGCTTTAATATGGGG
CGATGGACATTGCAGAGAAACAAAGATAGGGCAAGGTGAAGGTGCAAATGATTCCGCGCA
TCAGAAAATGATGGACGGAAACAAGAAAAAGATGGTTCTTCAAAAGATTCACACTTGCTTT
GGAGGGTCAGAAGATGATAATATTGCTGCCAAGTTGGAATCTGTTTCGGACGTGGAGGTGT
TTTATCTCACATCAATGTATTATATCTTCCCATTCGATAAGCCTTCTAGTCCTTCTCAATCATTT
AATTCTGCTAGATCAATATGGGGTTCTGATTTAAAAGGTTGCTTAGAGCATTTCCAATCAAGA
TCTTATCTAGCAAAGTTGGCTCGATTCGAGACACTAGTGTTTGTTCCGCTGAAATCAGGGGT
TGTGGAGCTTGGTTCTGTGAAGTCTATTCCGGAAGATCAGAATTTGATTCAGATGGTGAAAA

CATCGGTGGTGGTATCTAATCCTCCGCAGCCCAAAGCAAATACAAAGATATTTGGTCGGGAA
CTCAGTCTAGGTGGCGCTAAGTCAGGTCCCATCAGCATAAATTTTTCTCCGAAGGTGGAAG
AAGAGCTGAGTTTTGCTTCAGATTCGTACGAAGTACAAGCAGCGCTAGGTAGTTCTCAGGT
TTATGGGAACTCATCAAACGGGTATCGAAGTGATGAAGGTGAAGGGAAACTTTACAAGGAA
GAATTAGATGAACGGAAACCAAGAAAGAGGGGCAGAAAGCCTGCCAATGGGAGGGAAGA
AGCATTGAATCATGTTGAAGCAGAGAGGCAAAGGCGTGAGAAGCTAAACCAGAGGTTTTAT
GCTTTAAGAGCAGTTGTTCCGAATATCTCAAAGATGGATAAAGCATCGCTGCTTGGAGATGC
AATTGCTTATATCACAGATCTCCAGGCAAGAATTAGGGTTTTAGATGCTGAGAAGGAGATGG
TAGGCGACAAGCAAAAGCAGCAGGTTATCCTGGAGATTGATTTTCATCAAAGACAAGATGA
TGCAGTTGTAAGAGTAGGCTGCCCTTTGAATGCTCACCCTGTTTCTAGAGTTTTGAAGACAT
TTCAGGAACATCAAGTGGTGGCACAAGAATCCAATGTCTCATTAACAGAAAACGGCGAACT
TGTACACATGTTCTCTATACGAGCTCCTGGCCCTGCTGCTGAGGATTTGAAGGAAAAGCTGA
CAGCTGCTCTGTCTAAATGA

>SlbHLH118
ATGCAAGCCATGAATTCACTTCTTAGTCAACAACAGCAGTCACAGATATCACTTCAAGACCT
TCAAAATGGCGGAAATGGCGGTTCTACCGGTGGTGTTGGTGGTTTAAGTCAACACAGTATG
GGTCACTCTCACTTTGATCCGACGTCGTCTCATGACGATTTTCTCGAACAGATTCTCTCTTCT
GTTCCTTCTTCTTCTCCTTGGCCTGACCTTTCCAAATCATGGGATCCACATCACCATCTCTCA
TCTCCGCCGCATAACCCTAGCTCCGGCGAAGATCAACCTCCTTCTAATCCATTTCACTCACA
GTTCCATTACGACGACCAAGCTTCTTCTCTACTAGCTTCAAAGCTCCGTCAACATCAGATCA
CTAGCGGCGGCGGCGCCGCTGCAGCTGCTAAAGCACTTATGCTACAGCAACAGCTTTTGCT
CTCTAGAACACTCGCCGGAAACGGACTCAGGTCCCCAAACGGAGCTTCCGGAGATAACGG
CCTCCTTTCCCTACCCCTAAACCTCAGTAATGGTGACCAAAACGACGGCGTCGCTAATCCAA
CTAATGACAATTCCGTTCAAGCTCTTTTCAATGGATTCACCGGATCTCTTGGTCAAACCTCC
AATCAACCTCAACATTTTCATCATCCTCAGGGAGGATCGATGCAATCGCAGAGTTTCGGAGC
TCCGGCGATGAACCAAACTCCGGCAGCAAGTGGTTCAGCTGGTGGCGGTGGAGGTTCAAC
GCCGGCAGCACAACCAAAACAACAACGAGTTAGAGCTCGTAGAGGTCAAGCTACTGATCC
TCACAGCATAGCTGAAAGATTACGTAGAGAGAGAATTGCAGAGAGATTGAAGGCTTTACAG
GAGCTGGTACCCAATGCCAATAAGACAGACAAGGCTTCAATGCTGGATGAGATCATCGACT
ATGTCAAATTCCTACAGCTCCAAGTCAAAGTTTTGAGTATGAGTAGATTAGGTGGTGCTCCC
TTAGTTGCTGATATGTCCTCTGAGGGAAGAGGTGAAGGAAATGTTGGGAGGGGAGGTAACG
GAAGGGCGTCGTCATCTTCAAACAACGAGACAATGACGGTAACAGAGCACCAAGTAGCTA
AATTAATGGAGGAAGATATGGGATCGGCTATGCAGTATCTGCAAGGAAAAGGCTTATGCTTA
ATGCCTATATCCTTAGCTACAGCCATTTCCACTTCCACCACCAGAATCTCTAACAACCCACTA
CTTGCCCCCGAAGCCGGTGGCTCAACTTCTCCTACCCTATCGGCTTTGACTGTCCAGTCAGC
CACCGCCGGAAAAGACGCCACTTCTCTATCGGAAACTTAA

>SlbHLH119
ATGGTGAAAAAAAGAACAAAAGTTCTTAAAAGTCTTGTTCCTGGTGGAGAATACATGGATG
ATGCTTCTTTAATTAAAGAAACCCTAGATTATATTATTTCATTAAGAGTTCAAGTTGATGTTAT
GAGGCATCTAGCTAATAATGCTAGTTATGAGATTAATGATCCCAAAACAAGGTTGTAG

>SlbHLH120

ATGGACAACATTGGTGATGAATATAAAAATTATTGGGAAACAACTATGTTTTTACAATCAGA
AGAGCTCGATAGCTATTTCGATGAGCCAATTTCATCGTATTATGATTCAAGTTCACCGGATGG
GTCACAATCATCGATGGCATCGAAGAACATTGTATCAGAGAGGAATAGGAGGAAGAAATTG
AATGAAAGGTTATTTGCACTTAGAGCTGTGGTCCCAAACATAAGCAAGATGGATAAAGCTTC
AATAATCAAAGATGCAATTGATTATATTGAAGAATTACATAATCAAGAGAGAAGAATTCGAG
GAGAAATTTCAGAGCTTGAATCTGGAAGATCATCGTCAAAGAAGAATAGTAACGATGTTGA
ATTTGAACAAGATGAAAGTTTCGATTCAAAGCCTAAAAGATCGAGAAGATTTGAGATGCAA
TATGGATATGATTCTTCAGGATCAACTACTAGATCACCACCATCTTCTTCCCCTGTTGATGTTC
TTGAGTTGAGGGTATCTTCAATGGGAGAAAAGACAGTTGTGGTGAGTCTTACTTGCAGCAA
AAGAACAGACACAATGGTTAAAGTTTGTGAAGTGTTTGAATCTTTGAATATCAAAATAATTA
GTGCAAATATCACTGCTTTCTCTGGGAGGCTTCTCAAGACAGCTTTCATTGAGGCTGATGAA
GAAGAGAGGGATCTTTTGAAGTTGAGGATTGAAACAGCTATAGCTTCTCTAAACGATCCCG
ATAGCCCGATGAGTTCTTAG

>SlbHLH121
ATGAATAGAGGTGATGTTATGGAGAAATCACCAGTACAACAAATTATGGGTGGAAGCCCTA
AGTGGTGGAATATGATGAATAATATGAGGCCTCCCATATCATCATCTCAACAAGCTGCTGTTG
CTGCTACTACTACTCATCATGTTCCTAATTCCTTGTTGCCTCCTAATATTCTTTTTCCTCATTTC
TCTTCTTCACTAGTTCCCATGTCATCTACTACTAATTCATGGAATGATAGTAACCAAAGTCAG
CTACCTGAATCATGGAGTCAATTACTTCTAGGTGGCTTGGTGGAAGAAGAAGACAAGTCCG
TACATATGGTTAAGAAGTTGGAGAACAATTGGGATGAACAGTCGTTCCTGAGCCAGCATGA
TTCTGTTATAGACGTTAACCAAAAGGATCTCAGAAATAGTTATAATATGTATGAAGATGGAAA
TAATGTAGAGTTCCATCACCAAACTGCAGAATTAGCTGCTAAACCAACTTGGTCAGCACAG
ATGATACCAGTCTCATCTCCCAAGTCTTGTGTTACAACTTTGAGCAGCAACATGCTTGATTTT
TCTAACAAAAACACACATCCAACTCCAGATCATTCATCTGAGTGCAACAGCAGAGCACCTA
ATAAGAAGGCTAGGGTTCAACCATCTTCAACTCAGTCTACCTTCAAGGTGAGAAAGGAAAA
ATTAGGGGACAGAATAACAGCTCTTCACCAACTTGTCTCCCCATTTGGGAAGACTGACACA
GCTTCTGTCCTGTTAGAAGCAATTGGGTACGTCAGATTCCTACAAAGCCAAATTGAGGCCCT
TAGCTTGCCCTACCTAGGTAGTGGATCAGGAAATATGAGGCGCCAGCAATCTGTTCATGAGA
GAAATAATTTATTTCCTGAAGACCCTGGTCAGCTATCCAATGACAATTCCCTGAAAAGGAAG
GCAAATAGTGAACAGGATTATCAAGAAGATAAGAAAAAAGATCTGCGGAGTAGAGGTTTGT
GTCTAGTTCCGCTATCATGCACACTGCAAGTTGGGAGTGACAATGGAGCAGATTACTGGGC
TCCCGCATTCGGAGGAGGTTTCCGGTGA

>SlbHLH122
ATGGACTCCATCTTCTTTCTTGAAGAAGGAGACCGCACCGTCTTTCTATTAAAGATAATGGA
GTCATTTGGTTGCACTTACATATGCCTTTGGCAATACTTTCAACCTTCCAACACTTTCATGTC
CTTGGGTGGAATCTACAATGGAGAAAATGTCGTTGCCCAGAGATTATTTGAAGAATATAAGC
ATTCATGGCTTATCATGGATAACGGCCGCATTCCAGGACTGGCCTTCAAGAACAATGTTCCT
TATATGGAACTGAAGTTTGCTGATCTTCAATCTCATGCATCCAATCCAGTTCAGCTTCAATTC
TATTACACAACTATATGTATGGGATGCAGCATAGGAGAAATTGAATTTGGAATGACTTCTAGC
CCTCAAGTAAATTTGGAAATGGGGATGAAAAACTTATTTCCGGAATACTTCTCAACAAGATT
AGTACTTGCTCGTCCTCAGACATTGCTGACAAATATTGATCAAAACAGACCATCGCCATCTT
CATCATTCTCTCTCGATAGCCCAGGAGAATACTCATCGCTTCTATTCAATGTTGCAACTACAT

CTTACGTACCAGATGCCTTTCCAGAGCAGACCGTAAGACCAGTTTCTACCAGCGCGATGCCA
TTCCATCAACAACAACCTATTCAGACACTAACTCAACTCAGAGGCATTCAATTCCCTGGGGT
AGAAACTGATGATGCTGCATTGACAAGAGCGTATCTTGCTGTTATGACTTCACCTTCTTCTTC
ATCTTCATCTCATCAATCGCGAGAAAATATTGATGTACCAATAACGGATTATCATTATCAAAA
GTCCACTGCATTCAGAAGGTTTGGACCAGGTTTAGGTCGTCCTAGTAATGTGCAAATAGGCA
CATCCAGAACAATTCGTCGAGAAAACATATTGAGAAGATCAATTATATTCTTCAGGAATTTA
GATATGATGAGAAGGAAAGAACAAATCCAGGCGAATCAGCGTGCCCTTACTAGCACTCAAG
TTCATCATATGATTTCAGAAAGAAAAAGACGTGAAAAGCTTAACGATAGCTTTCAACTACTT
AGATCTTTACTCCCTCCTGGTACAAAGAAGGACAAAGCATCTGTTCTTGCTAGTACAACAG
AATACATAACTTGTTTGAAAGATCAAGTGGAAGAACTTAGCAAAAAGAATGAAATAATGCT
GAATGCACAGGCGTTAGATAAATCATCGATGATGAAATCGAATGACGTTGGTGATGGAAATG
ATGAAAGGGTAGTTGTTGAAATAATAAAAAATGTAAGTAGTGAATCAGAATCAAGAACAGT
GGAATTGCAAGTTTCAGTAAGATCAGGAGAATGCAACGTGTTGGATTTGGCCACTCGTTTG
CTCGAGTTCTTGAAAACTCAAGACAATTTAAGCTTACAGTCAGTAGCAGCAAACACTAGGC
CATCCATGGTTACTCACGTAAGTTTGACAATTACAATTCAGGGTAGTGAATGGGACGAGTCT
GGTTTCGAGGAAGCAGTGAAAAGGGTTGTTGATGACTTGACATAG

>SlbHLH123
ATGCTGCAAGATATTAAAGAGGGGTCTGTGATGACAGTTGGTTCAAGCCACTGTGGCAGCA
ATCAAGTTGATACTAGCCGGTTTTCAAGTAGTGCAAATAGAGGGCTGTCTGCAGCAATGATC
ACTGATTATACCGGAAAAATCAGTCCACAAAGTGATACAATGGACCGAGACACATTTGAAC
CAGCTAATACATCTTCGTCTTCAGGAAGATCGGGTAGTAGTTATGCAAGAGCATGCAATCAA
TCTACAGCGACCAATAGCCAGGGCCACAAAAGGAAGAGTAGAGATGGTGAAGAACCAGAA
TGCCAGAGTAAAGCTGATGAGCTAGAATCAGCTGGAGGAAACAAGTCAGCCCAAAAATCT
GGAACTGCCCGAAGGAGCCGTGCTGCAGAAGTGCATAATCTCTCTGAAAGGAGACGGAGG
GATAGAATCAATGAGAAAATGAAGGCCTTGCAAGAGCTTCTTCCTCACTCTACTAAGACAG
ACAAAGCATCAATGCTGGATGAGGCTATTGAATACTTGAAATCACTTCAGATGCAACTGCAG
ATGATGTGGATGGGAAGTGGCATGGCATCAATGATGTTCCCTGGTGTCCAACACTACATTTC
CAGAATGGGAATGGGGATGGGTCCGCCTTCGGTGCCTTCCATGCACAATGCTATGCATTTAG
CTAGGCTTCCTTTGGTTGATCCAGCAATCCCTTTGACACAAGCTGCCCCTAATAATCAAGCA
GCTGCAATGTGCCAGAATTCAATGTTGAATCAAGTTAACTATCAACGCCATTTGCAGAATCC
CAATTTTCCAGATCAATATGCTAGTTACATGGGGTTCCATCCACTTCAAGGCGCTTCTCAGCC
TATAAACATTTTTGGCTTAGGTTCACATACAGCACAGCAAACTCAGCAGTTACCGCATCCAA
CTAATAGTAATGCACCTGCCACTTGA

>SlbHLH124
ATGGAGGTCGATTCTAGTGGGAATCCTAATTGGTTATTTGATTATGAGTTGATGACGGATATT
ACTTCTGCTGCATCTGTTACCGTCGCTGAGTTTCAGTCTCCGGCTACTATTGATTTCAGCTGG
CCTGCTCAAACGATCTATGCTTCTTCTAATCTCATTACTGAAACAGATTACACATTTGCGGAT
TCAGAAGTTAGCAAGGAGGCAAGCTCACGAAAGCGGTTAAAAAGTGAATGTTGCAGCTCT
CCGAGATCTAAGGCATGCAGAGAGAAATTGCGGAGGGACAGACTGAATGAGAGGTTCCTC
GCATTGAGCTCTGTCCTTGATCCTGGAAGGCCACCAAAAACTGAGAAAGTTGCAATTCTAA
GTGATGCTCAAAGGATGCTGATTGAGCTGCGAACTGAAACCCAGAAGCTGAAGGAGTCAA
ATGAGGAGCTGCAAGAGAAGATAAAAGAACTTAAGGCAGAGAAGAATGAGCTCCGAGATG

AAAAGCAAAGGCTAAAGGAAGAAAAGGATAATTTGGAGCAGCAGGTTAAAAGCTTAGCTT
CTAAAGCAGGATTTCTCTCCCATCCTTCTGCCATGGGAGCTGCATTTACTGCACAAGGACAA
GTTGCTGCAGGCAACAAATTGATGCCTTTCATTGGTTATCCCAGTGTTGCGATGTGGCAATT
CATGCAACCTGCTGTTGTTGACACATCTCAAGATCATGTGCTCCGTCCTCCAGTTGCTTAA

>SlbHLH125
ATGTTACGGGCAGGAACCTACATTTCCACCGTTGGATCAAAATCTAATCAACGGATATCCCT
TGTATTTTTCTGCGTGTTTTGTGTGAGGGCTATGGAGAGAAAATTACCTCTCGTTCAACCGG
AGTTTAATGCCGGAGAAGCGTTATTGTTGCCACTTATGGAGTCTGATGAAGCCTTCATCAAC
GGCATGTATAATGGATTGAGCTATCAGTCTCTGTTGAGTTTGAACCTCGACCACAGCTACTAT
GGCGAAGTGAAACCTTTTTCAGCTAATTCATCTAATTTCGCCCTTCTTTCACCAATGATATG
CTCTGCGCTGGTACTACTGCTTCTTCTCAAATCCAGCAAAGTTCGGGGACTTTTCATGAGTT
CTCTACTGGAGATTTTGCATGGCTTGATAAGAAGACGGAGATCAATCCAGATGCTGATCAAA
AGTTGAAATACCCGAAACTAGAACCAGTAACTGATAATCTCCAGTTATATCCATACAACAAT
GAGGTTTTGTTGTGTGAGCCGTTCAACTTCTTCTCTAACGGTGTTGGATACCCCAGTCTTCC
AGATCTCCGTTGCATGGAACAACCAAACTATTTGGATTTCTCTTCATCACTGGTCACAACAG
AAAACCGAGTCGTCGAACCGGTGAGGCCTGTTTCAGAAGTAATGAGTAACCTCCTAAAAA
ACAGGCAGCCGTATAGTTCATCTACAACTCGATTGCGAAGGCAGAAGCTAAGCGAGAAAAT
AAGATGTTTAGAGAAGCTATTGCCATGGGACAAGAAAATGGATACGTCAACTATGCTAGAA
GAGGCGTACAAATACGTGAAGTTTCTACAGGCGCAAATTGACGTCCTACAAAGCATGCCTC
CGCTGGTAGAAGGAGGTGCTTCTTCCGATCAGGATAGAAATGGAAAAAGTACAAGCTATGG
ATTAAATCATGAAGCGAAGGCAATTAGCGTTTTTGGGACACTAGCGAGGCTAAACAGGCAG
CAGCTTTTACAGGTTTTACTGAACTCGCCTGTTGCTCAGACGTATCTCTACTCAAAAGGCTG
CTGCGTTTACTCCGTAGAACAGCTGGTTCAGTACAGGACAATTGCTCAGAGAAACGCTTTC
TACCGACGATCTTTGTTTCTTTCCGGCATGCTCTCTTGA

>SlbHLH126
ATGGAATTTTTAAGTTCTTTTAATGGGTTGAATGAGGTTTATGGGGGGTTTCAGGGTATAATA
GGAAATGGGTTGTCATCATCTTCTTCATTAGTTTTGGATAATGAGAGTGGTGAGCTTGTGAA
GGCAATGGTGAAACCAGGGGGAAAAGGGGTTAATCCAGAAAAAGCTTTGATTGCTTTGAA
GAATCATAGTGAAGCTGAAAGGAGGAGGAGGGAGAGGATTAATGGTCATTTGGGTACTCTT
AGGAACCTTATACCTGGCACTAATAAGATGGACAAGGCTGCTTTACTTGCCAAAGTCATCGG
CCACATAAAAGAATTGAGAGTTAATGCAGCAGAAGCTACCAAAGGTGTTCTAGTTCCAACA
GACATTGATGAAGTAAAAGTTGAACAACAAGCAGAGGGATCTGATGGAGCCACTTATTCTG
TCAAGGCATCTTTGTGCTGTGACTATAAGCACGAGCTTATCTCTGATTTACGTCAAGCTCTG
GATACTCTCCCCCTAAAAACTCTGAGGGCAGAGATTGCTACACTAGGGAGCAGAATGGTTA
GCGTTTTCGTGATTACTGAGGGCAATGAAGGGAATACTGAGGGTACTGAAAGGTGCCAGCT
TCTTATAACTTCTGTTCGTCAGGCTTTGAGGTCAGTGCTTGATAAATTTATGCATCCGAGGA
ATTCTCTTCGAGAAGTACACTATCAAGCAAGAGGCGAAGAGTTTCCCTCCTCAATTCTTCGA
GCTCATCTTCTTTAGGGGATTTCTGGTGA

>SlbHLH127
ATGGAGAGTAGTTGTAATAGAGAAGAATCAAGTGCCACATTTTCAAGAAAAGATAAGAATG
TGTGTAAAAGGCAAAAGAGAAGGAGAGAAAGTGGCTATATTGTTAAAAATAATGAAGAAG

AAGAAGAAGAAGTGGAAGAGAAGATTTTGGCATTGCAAAAGATAGTACCAGGAGGTGAAA
CACTTGGAGTTGATAGGTTATTTGAAGAAACTGCTGGATATATTTTGCAATTGCAATGTCAAC
TTAAAGCACTCAAAGTACTTGCCAACTTTGTTGAAGGAAATGACAAACAAAGGATGAAACT
TGGAGGTTAA

>SlbHLH128
ATGGATGATTCCGAATTTGACTTTGGCCAATTAGATCAACTATTCAACTTTCTTTCTTCTCCTT
CTCCTCCTCCTCCGCCTCCAACTACTCTTCAAAGTTATCAAGATTCATCATTTTCATTACAAA
AACAAAACTCAAATATTGTTTTTACAAGTACTCATAATGTACCTAAAAAGAAACCAATTTTG
ATCACCAATATCAATATTATTGATGATCAAGTAATTCAAGATTTACCTAAAGAGAAAGAGATG
GTGGTGGAGAAGAAAGTTATGAGAAGAGATGTTGAAAGGCAAAGAAGGCGCGATATGGCT
AAGCTTTATCAACGTTTACGCCTTCTTATTCCATCTAAGTATCTCATGGGAAAGAGATCTATAT
CTGATCATCTAGAAGAGATAGTGGACTATGTAAAAGATTTGAAGAAGGACATAGAGGAGTT
GGAAAGTAAAAGAGAAAAATTGAAAGAAATGAAAAATATTACTAATATTAGCTCTCCATTG
GCACCAAATTCTTCATCTATGAAATTAAATGATGATGATGATGAAGATAAAATAATAGTGAAA
TCATGCAATGAAGGAGTGGAAATTTCAATAAAAGGAGTGCTTTCTATTTCAAAAGTGCTTAA
AGTTCTTATGAAAGAAGGATTTATTGTTAATAGTTGTGTTTCCTCCACAATTAATCAAAGGCT
TATTCACATTATTCAAACTAAGGTGAATAAAAGAGGAGATATTGATCTTGCATTACTACGATC
CAAGTTGATGGGGAAAAAGAGCTATATCTGA

>SlbHLH129
ATGGTGGGCTCAGGAACAGCAGATAGAAGCAAAGAAGCAGTTGGGATGATGGCCCTTCAT
GAGGCACTAAGAAGCGTCTGTCTTAACACAGACTGGACTTATTCAGTGTTCTGGACCATTC
GTCCTCGACCGAGAGTTAGAGGTGGTAATGGTTGTAAAGTTGGAGATGACAATGGTAGCTT
GATGTTGATGTGGGAAGATGGATTCTGCAGAGGTAGAGGAACAGATTGCTTGGAAGAGATG
GATGGTGAAGATCTTGTGAGAAAAGCCTTCAGCAAAATGTCCATTCAGTTATATAATTATGG
AGAAGGGTTGATGGGTAAAGTTGCATCTGATAAGTGTCATAAATGGGTTTTCAAGGAACCTA
CAGAATGTGAACCAAACATATCTAATTACTGGCAGAGTTCATTTGATGCTCTCCCACCTGAG
TGGACTGACCAGTTTGAGTCAGGGATTCAGACTATTGCTGTGATTCAAGCTGGACATGGCCT
ACTGCAACTGGGATCCTGCAAGATTATACCGGAAGACCTCCATTTCGTGTTAAGAATGAGGC
ACACGTTCGAGTCACTAGGCTATCAATCTGGCTTTTATTTGTCACAACTATTTTCTTCAACAA
GGACTAGTTCACCTTCGTCTGCAATTCCTCTTAAGCAGCCGACCATGCCAATTCGCGCTCCT
CCTCCACTTTTCAACTGGGGACCAAGGCCAATGCCTTCAGCGTCTTCGCTACTATCGTCTCC
CAACTTCCAGAACTCCGCGAGACTTGGTATTCCACAGTCGAAAGATGAATCACATATGTTCC
TTCAACTTCCTCATTCATCCGAACCACGAATGGAAGACATGATGGGAGCTGCTGCTGATCAT
GAGAGTGATATCAAGTGGCCTAATGGATTAACTTTCTTTAGTGCTCTCACTGGTAGAAATGA
TGATTCCAGGATCCTGTTCAATCCTGATAGCTTAGGTTCCAAACCGGATCATAATCAGCATCC
GCTTAGTCTTGATGGGAAGACATCAAATCCGAACTCAGATGCTTCTAGCTTGCACAACAAC
GGAGGTGCTAATCCAAACGATTTCTTGAGCCTGGATAGCCACCCTGATAGCATTCGGAAGAT
GGACAAGTTCAAGAGAAGCTATACGCTTCCTGCTAGGATGGCTTCGTCTTCTAATTCATCGA
CTTCACTTGATCAGCACGCTAATAATCCCGGAGAATATAGGAATGAAGGAGGAATGTACCCT
GATGTGATGGAGAGATTCTTGGAATGA

>SlbHLH130

ATGGCGGAAGAATTTCAACTAGGTAGAGGAAATTGGTGGGAATCATCAACTTCAGCTTCAT
CAACAACAACAACATCATCATCTAGAAATAAGTTTATCGATAGCGGAATTTCATCATGTACA
ACTCCTTCAGCATCGAGTACAACCGGTCTAAGCAGCATGGCAAGCAACTTTGTTAATTGGC
CAATAGAAATTCATGAGGATATTAAAGTTAGATCCGAAAATAGTTCTATGGTTTTCTCGGGCA
CGGATTCCCATAAACGTTACGCCTCCGGAGGAGGAGGTGGAGGCGTGGGGCAAGGTGTTTT
ATCTGTTGATGATCCTAATTTACAAATAATGGGGTTAGGTCTATCTTCTCAAGGACTTGATTG
GAATCAACCTTTTTTTCGTAGTGAAAAATCGGGGAGTGGTTTTCGTTCGTTAATTCAAGAAG
GGTTGAGTTCAAATGCAAATTATCAACAAGAAGGAACATGTCAAGAACAAGATCATAATAA
TTGGAGTACGCAAAAATTATATCATGGGAATTCTGATGATTCTTCAGTAAATGATTATAATAA
ACAATTATTTAGTGGTCATAATAATAATCTTGAAAATTCAGCAGTACCTTATGGAAGTCCATC
AAATATGTTACAAGGGTTATTAATTTCTGACCTTAATAGTCAACAACAAGAATCAAATAATTT
TTCAAGTGTTAGTAGATCTTTATATTATAATCCTTCATATAATAACCAACCAAATTGTGATGTC
AATATTCCTACTTCTACTTCATCATCATCTTGGTCTAAATTTCCTCAATTTCTTCGAACATCTG
ATCCTTCAAAAGTAGTACAACAGTGGTCACTGTCACAATCACCACCGCTATCTCATAGCCAG
TTGTCACATTTCTCTGGTGGCACGTCTTTTTGGAACGCCACATCAGCTGCCGCGGAGGACG
TCCGATCGGGTTTCCTCCCTCAGCTCCCCACCAACCCAACGGTTGATGAGAAACCAAAGCA
TACAGGTGAAGTTAGGAATACAAGCACAGTAACAAAGAAAAATAGCAGTGAAACATCAAA
TAAAAGACCAAGGAATGAAGCACCATCTCCATTGCCAGCTTTTAAGGTGAGGAAAGAGAA
AATGGGAGACAGAATCACTGCACTTCAACAATTAGTCTCACCTTTTGGAAAGACTGATACA
GCTTCTGTGCTATCGGAAGCAATCGAGTACATAAAGTTCCTCCATGACCAAGTTAATGTATTA
AGCACCCCATACATGAAAAGTGGAGCTTCCATACAACATCAACAGAATACTGGTGATAAATC
CAATGTAAATCCAGAAGGAGGAAAACAAGATCTTAGAAGTCGAGGATTATGTTTGGTCCCA
GTTTCTAGTACCTTTCCAGTAACGCATGAAACAACAGTTGATTTTTGGACTCCCACCTTTGG
AGGAACATTTAGATAA

>SlbHLH131
ATGGTTTCACCGGAGAGTACCAATTGGCTTTATGATTACGGATTCGAAGATAGTTGCGTCCC
TGATTCGAATTTCTCAGCTTCTGCATCTGGGTTTAACTGGTCTGTGCAGAATTTGAATGGTTC
AAGGAATGTTAGTTCTGAAATCGATGGGTCAATTGGTGAATCAGATTACCCCAAGGAAAGT
GGTTCTAAGAAACGGGCAAGGGTTGAATCATGTGCTCCAACAAGTTCCAAAGCTTGCAGA
GAGAAACTGCGAAGAGATAGGCTGAATGACAAGTTCATGGAATTGGGTGCACTCCTTGAGC
CTGGAAGACCCCCTAAAACAGACAAATCCGCTATTCTTGTTGATGCTGTTCGCTTGGTGACC
CAGTTACGTGATGAAGCTCAAAAGTTGAAAGACTCAAACTTGAATCTGCAAGAAAAGATC
AAGGAGTTAAAGGTTGAGAAAACCGAGCTTCGAGATGAAAAACACAGGCTGAAAGCTGA
AAAGGAGAAGCTAGAGCAACAACTAAAGACTACAAGTGCACAGCCTAGTTACTTGCCTCC
TGCTATACCTTCTGCATTTGCTGCTCATGGTCAATTTCCAGGAAGCAAGCTGGTGCCAATCAT
GAGTTACCCTGGTGTCGCGATGTGGCAATTCATGCCTCCTGCTGCTGTTGATACTTCACAGG
ACCACGTCCTCCGTCCTCCAGTTGCTTAA

>SlbHLH132
ATGACGGACTATAGATTATGGAGTAATACCAATACTACTAATACATGTGATGATACTATGATGA
TGGATTCTTTTTTATCTTCCGATCCATCCTCTTTTTGGCCTGCTTCCACTCCCAATCGTCCGAC
TCCGGTGAACGGAGTCGGAGAAACGATGCCGTTTTTCAATCAAGAGTCACTACAGCAAAG
GCTTCAGGCTTTAATTGACGGTGCTCGTGAATCATGGGCATATGCTATTTTCTGGCAATCGTC

AGTTGTTGATTTTGCGAGCCAAACTGTATTGGGTTGGGGAGATGGGTATTATAAAGGAGAAG
AAGATAAGAATAAACGGAGAGGGTCGTCTAGTTCAGCAGCTAATTTTGTTGCTGAGCAAGA
GCATAGAAAGAAGGTGCTTCGGGAGCTGAATTCATTAATATCCGGTGTACAAGCTTCCGCCG
GAAACGGAACTGATGATGCAGTGGATGAGGAAGTGACGGATACTGAATGGTTTTTCTGAT
TTCAATGACCCAATCGTTTGTTAACGGTAACGGGCTTCCGGGCTTGGCGATGTACAGTTCAA
GCCCAATTTGGGTTACTGGAACAGAGAAATTAGCTGCTTCTCAATGTGAACGGGCCAGGCA
AGCCCAAGGTTTCGGGCTTCAGACGATTGTGTGTATTCCTTCACCGGAGTCCAGGGAGATAT
TGAATTTTGGTGATAGTAGTAAGAGATTTTCAGGGCAATCACAGTTGGGTCCTGGGCCTGGG
CTCATGGAGGAGAACAAGAACAAGAACAAGAACAAGAAAAGGTCACTTGGATCAAGGGG
AAACAATGAAGAAGGAATGCTTTCGTTTGTTTCGGGTGTGATCTTGCCAACTTCAACAATG
GGGAAGTCCGGGGATTCTGATCACTCAGATCTCGAAGCCTCAGTGGTGAAGGAGGCCGTTG
TAGAACCTGAAAAGAAGCCGAGGAAGCGAGGGAGGAAACCAGCCAATGGAAGGGAGGA
GCCATTGAATCACGTGGAAGCGGAGAGACAGAGGAGGGAGAAATTGAATCAAAGATTCTA
CGAGTTGAGGAGCCAAATTGAATGTTTAAGGAAGGAATTAACCAACAAGGGATCATCAAAC
TATTCCGCCTCCCCTCCATTGAATCAAGATGTCAAGATTGTCGATATGGACATTGACGTTAAG
GTGATTGGATGGGATGCTATGATTCGTATACAATGTAGTAAAAAGAACCATCCAGCTGCCAG
GCTAATGGCAGCCCTCAAGGACTTGGACCTAGACGTGCACCACGCTAGTGTTTCCGTGGTG
AATGATTTGATGATCCAACAAGCCACAGTCAAAATGGGGAGCCGGCTTTATGCTCAAGAAC
AGCTTAGGATAGCATTGACATCAAAAATTGCTGAATCGCGATGA

>SlbHLH133
ATGGAAAATTTAAATATATCTACTTCATCAACTCCTTCACAACCAAACACACTTCAAAAAAC
CCTTCAATATATAATCCACAATCGTCAAGAATGGTGGGTTTATGCCATTTTTTGGCAAGCATC
CAAGGACGTTAATAACCGTCTCATTTTATCGTGGGGCGATGGCCATTTTCGCGGAACTAAAG
ACACAACAGGTTCCACGAAAACAGGTCATGGTCAATATCATCAATTTCAGAAGAAATTTGG
TTTCAATGATATTAGTGAAACAAATAATAACGTTACGGATACAGAGTGGTTTTATATGGTGTC
TATGCCACAATGTTTCGTGGCCGATGATGACCTCGTTATACGAGCTTACACCTCGGCCTCAC
ACGTGTGGCTAGCTAGCTATTACGAATTGCAAATTTATAATTGTGAGAGAGCTAAAGAAGCT
AATTTACATGGAATACGTACGATTGTGTGTATTTCTACGACTAGTGGTGTTGTTGAATTGGGT
TCCTCTGATGTTATTCAAGAAAATTGGGAATTTGTTCAATTCATTAGGTCTCTATTTGGATCA
AATAATAACATGAATACGACTTCTCATCTACCTGTCAATCAAGTAACCTTGGGAGATGATCAT
AAGGTTGCAAAATGTGGATCTAATATAATAGTAAAGCAAGAAATGACTATCGGAAATTTATTA
TCTGAATCGGGTATTTCTGATTTCGAAAATGATGACTCTTTAACCATCAACAATGTTATGAAT
GGGTCAATAAAACGAGCAAAGAAGGGTGATTCGAGCCATATAAGACGAGAAATGGCTATGG
ATGTTCACGTAGAGGCAGAGAGAAAAAGAAGGGAAAAGTTAAATCATAGATTTTACGCTCT
ACGGAGCGTAGTCCCATACGTGTCCAAAATGGACAAGGCTTCATTGCTAGGTGACGCAGTT
ACTTATATCAATGAACTTAAAGCTAAAATTAAAAATTTAGAATCCAAATTAATCGAGCCCCAG
AAAAAACATATACTTATGGAACAACACGATTCTCATAGTGCATCCTCCACTATTGTAACTGAT
CATGGGGCAAACAACAAGTCATTATTTTCTAGCAACGGGGTACGAAACGGGATGGAAATTG
AAGTGAAAATTATTGGATCTGAAGGTGTGATTCGGGTTCAATCTCTGGATATGAACTATCCAT
GTACTAGATTGATGAACGCGATGAAAGAAATGAAATTTCAGATTTACCATGCAAGTATTTCC
AGTGTTAAAGATTTGATGCTTCAAGATATCGTGATTAGGGTTCCTGAGGAGTTTTCGAATGA
GGAAACATTGAAATCTGCTATCATCTCAAAATTAAGTGTTATGGAGAATTAA

>SlbHLH134
ATGATGCAACACAAAAGAAGTCCAATTTCCCTCGAGCATAGCAGCAGCCTCACATCTCTTAC
ACCAAAACGACTAAAGGCCGATATGCTCATTTCCTCCAAGGAGAAGAAGGAGAAGTTTGGT
GAACGTATAGTTGCTCTTCAACAGCTGGTGTCACCATATGGCAAGACAGATACGGCTTCTGT
TCTCCTTGAGGCAATGGGATACATAAAATTCCTCCATGAACAAGTGAAGGTGTTGAGTGCA
CCATACCTCGGAACTATGCCAATGTCGAAGACACAGGAATCACAACCTTACAATTTGAGAA
GCCAAGGCTTATGTCTTGTGCCGGTATCATATACTGTTGGTGTGGCAAGCAGCAATGGTGCT
GATATTTGGGCTCCCATTAAGACCTCACAAAAGTTCTAG

>SlbHLH135
ATGGAACTCATGCATCTAATGGAGAGGCTTAGGCCTATTATGAGCTTAAAAGGCTGGGATTA
CTGTGTTTTATGGAAGTTGAGTGAAGATCAAAGGTTTCTTGAATGGATTTGTTGCTGCTGTG
GCGGAGCTGAGAAAAATATGCATGGCTGTGGACAAGAGATATTTTTTCCTGATTCTTCTACT
TCAACTTGCAGAGATGTTATGTTTCAGCATCCAACAACAACTGCTTGTAATTTACTGGCTCA
GGTCCCGCCTTCTCTGGCGCTAGACTGCGGAGTTTATGCACAGACCTTACTATCTAATCAAG
CAAAATGGATGAACTTTGTTCCTTTCTCGGAATCAAATATCTCTAATGAAATAATGGGAACTA
GAGCTCTGATTCCATCTCCTCTTGGATTGCTCGAGTTGTTCAGTACGCAACAACTAGCAGAA
GATGAGAAAGTGATAGAATTTGTCTCAGCTCAATGCAATATCTACTTGGAGCAGCAAGCTAT
GATGAATTCAACTTTCTCAAATGGAGTTGAAGAGAACAACACATCAAAGCCTTTCCCAACA
GAAGGAGAAAGAGACAGAGACGATCATATAAAAGATTCCCAAAATCATTACAAACAGAGA
GTCTCCCCTGCAGCTACATCAGATCACTTGTCATTTGATTTCCCACTTAAACGAAAACAATT
GGATTCTTGTTCGATGAACTTCCTTCCACCGTTCAGTACTTATAGCACACCAGAAGTGGATA
ACAACACAGGAGGGAACATGTTGTTTGATCAGAGCACAAGTGATATGACACATTTTTCGGA
GAATAGGTACATGAGTGAGATGGATGCTTATTTACAGAAGCAAATGATGAGAAGCAGTAGT
ACTCAAGCTGGAATTGATGATGAATCAATCAAACATGATAACGGAAGATCAAATTCGGGATC
TGATAGTGATCAAAATGAGGAAGAAGATGATCCCAAGTATAGAAGGAGAAATGGAAAAGG
TCCTCAATCCAAGAATCTTATGGCTGAAAGGAAAAGAAGGAAGAAACTAAATGAAAGGCT
CTATGCTCTTAGAGCTTTGGTTCCCAAAATCTCCAAGTTGGATAGAGCATCTATCCTCGGAGA
TGCTATTGAATATGTGATGGAATTGGAAAAGCAAGTGAAAGATCTGCAGCTTGAGGTTGAA
GAACATTCAGATGACGACGGTACTGGTGGAGGAAGGAATTCGGACCAGATTCACCCCGTAG
TTTTAAGCCATAATGGAACTAAAAACAGGCCTAAATCAGATAACGGAAAACTTACAAATGG
AAGCCAAAGAGAAATATCAACTAATTCTAATGGCAGCACTGACCCTTCCAGAAAAAATCAA
GATGTAGAAGAGAACGACAAATTGCAGCAAATGGAGCCACAAGTGGAAGTTGCTCAATTA
GATGGGAATGAGTTCTTTGTGAAGGTGTTTCGTGAACACAAGGCTGGTGGATTTGTGAGGA
CTTTGGAGGCTTTGAACTCATTGGGCTTGGAAGTTACCAATGTTAATGCAACTAGGCATACT
TGTTTGGTATCAAGTATCTTCAAAGTTGAACAGAAAAGGGATAATGAAATGGTTCAAGCTGA
TCATGTGAGGGACACCTTGCTAGAGCTGACAAGAAACCCTAGTAGAGGTTGGTCTGAAATG
GGTAGAGCATCATCAGATAATATAAATAATAATAATGCAAATGGCACTACAGATTATCATCAA
CATCAACTTCATGATCATCACCTAGACAATAATAATCAACATAAGCAAACCAATTCTCATCAC
TTCCATACACACCACCATCACTAA

>SlbHLH136
ATGATGACCATGCAGCAGCTTCTTTTTGATTTCAACTATGATGTTGAACAGAACTTTTCTGAT
GAAAATCAAGATTGCTATTTCGATCCAGACGAGTTTATTTTACCCATTGAAATGAATAACAGT

TGTTGTTTCATGCCAGAGTACTCTGTTTTGGAGAAACAACCAAAGGACAATCATTCTTGTTT
CATTCCAGAGTACTCTGTTTTTGAGAACATCCCAAAACGTCAAAAGATTTTTCAAGATGATT
TCTTTCCAAATCCAAATAGTAATACGATAACCCCATCCACTCACAATTCTTGTTTCATGCCAG
AATACTCTGTTTTTGAGAAACAACAAAAACTCTTTCAAGATAACTTCCATGAGGAGGGGTT
TTTGCCAAATCCTCCAATGTTTGAGGATTTTGCGCTTCCAGAAATTCCTGTGCCTGTTTTTAG
TGCTGGAGTTGTCGCGAAGAAAGGCGGTAGTAGTAATAACGAGAAGAAGATGTCAGCACA
GAGTATGGCGGCGAGGCAGAGGAGGAAGAAGATTAGTGATAAAACACAAGAATTGGGCAA
ATTGATACCTGGTGGACACAGAATGAACACTGCTGAAATGCTTCAAGCTACTTACAAGTATA
TTAAATTGTTGCAAGCACAAGCTGGAATTCTTGCCTTCATCGGATCATATCAGGAAAATGAG
AAATCATTTGAAACATCATACTTGCAGAAACTTGTTGGATCTTCCTTAGTTCAAGAGAAGTT
GTATTCAAGTGAAAGTTCTATACAAATATACCATATTTCAATCCGACCCGGCCCATATCCACA
TCAACATAGGGCACTAAACTTTACAAAAACCCTAACACAGACAGAGTCGCCATTTCACCTG
CCGTGGGCACAGAAAAGCTCTATCAACCCTATTCTCTTCTCACTTTTACAATATCAAGAGAT
GGGTGAGGTAGTTAAGGGAGCTGTTCCAGTTCCAGAGTCAGTATTGAAGAAGCAAAAAAG
GAGCGAGGAATGGGCCCTTGCAAAGACACAAGAGCTTGTAGCTGCAAAGAAGAAAAGTG
CTGAGAACCGGAAATTGATCTACAACAGAGCTAAGCAGTATGCTAAGGAGTACGACCAGCA
GGAAAAGGAGTTGATCCGTTTGAAGCGTGAGGCTAGATTGAAGGGTGGTTTCTATGTTGAC
CCTGAAGCTAAGCTGTTGTTTATCACTAGAATCCGTGGGATTAACGCTATGCCTCCACAGAC
CAAAAAGATATTGCAGCTTCTACGGTTGAGACAGATCTTTAATGGTGTCTTTTTGAAAGTCA
ACAAAGCCACTGTCAACATGCTTCACAGGGTTGAACCTTATGTTACCTATGGTTACCCTAAC
CTAAAAAGTATTAGAGAATTGATCTACAAGAGAGGTTATGGGAAAGTTGACAAGCAGAGAA
TTGCTTTGACTGACAACGCTGTCATTGAGCAGGTATTGGGTAAGTATGGAATTATCTGCATG
GAAGACCTAGTCCATGAGATCATGACTGTTGGACCCCATTTCAAGCAGGCCAACAACTTCC
TATGGCCGTTCCAACTCAAGGCACCTTTGGGTGGACTGAAGAAGAAAAGGAATCACTATGT
TGAAGGAGGTGATGCTGGTAACCGTGAGAACTTTATCAATGAACTTATTAGGAGGATGAACT
AA

>SlbHLH137
ATGATGACCATGCAGCAGCTTCTTTTTGATTTCAACTATGATGTTGAACAGAACTTTTCTGAT
GAAAATCAAGATTGCTATTTCGATCCAGACGAGTTTATTTTACCCATTGAAATGAATAACAGT
TGTTGTTTCATGCCAGAGTACTCTGTTTTGGAGAAACAACCAAAGGACAATCATTCTTGTTT
CATTCCAGAGTACTCTGTTTTTGAGAACATCCCAAAACGTCAAAAGATTTTTCAAGATGATT
TCTTTCCAAATCCAAATAGTAATACGATAACCCCATCCACTCACAATTCTTGTTTCATGCCAG
AATACTCTGTTTTTGAGAAACAACAAAAACTCTTTCAAGATAACTTCCATGAGGAGGGGTT
TTTGCCAAATCCTCCAATGTTTGAGGATTTTGCGCTTCCAGAAATTCCTGTGCCTGTTTTTAG
TGCTGGAGTTGTCGCGAAGAAAGGCGGTAGTAGTAATAACGAGAAGAAGATGTCAGCACA
GAGTATGGCGGCGAGGCAGAGGAGGAAGAAGATTAGTGATAAAACACAAGAATTGGGCAA
ATTGATACCTGGTGGACACAGAATGAACACTGCTGAAATGCTTCAAGCTACTTACAAGTATA
TTAAATTGTTGCAAGCACAAGCTGGAATTCTTGCCTTCATCGGATCATATCAGGAATCATTTG
AAACACCAAATTTGCAGAAACTTGTTGGATCTTCGTTGGTTCAAGAAAAGTTGTATTCAAG
TGAACATTGCTTAGTTCCAAAAGTGTTTGTTGAAGCACTAGAAAATAATCAAGAATTCCAA
AATTCACAAATCCTTGAGGAAATCAAAACTTTGATGAAGGAAGGAAAATGA

>SlbHLH138

ATGGCTTTAGAAGCACTTTCTACCAATGAACTCCTCAACTTCATTATCTACGATACAATCTCT
ACCACCACCACCACCACCAATAGCAACAACAATCTTGATGAAAATGCCTTATTTTTTGACAA
TCATGAAGAAAATAATGCCTTTCTATTAAAGCCTCAAGATTTTTGTACTACTACTCCCTTAGA
GCAACAATATTCCATCGCCGCCTCAGTGCCGCGACCAGAGTCTTCTAGAGAGAAGAATAAT
TTGTCGGTCGCGGCACAATCAGGAGGCGGCGGTGGAGGGAGAAAGAAAAGGAGAAGAAG
ACCTAAAATTTGTAAGAATAAAGAGGAAGCTGAAAATCAAAGAATGACTCACATTGCTGTT
GAGAGAAATAGGAGGAAACAAATGAATGAACATCTTTCTGTTTTACGTTCTCTCATGCCTGA
ATCTTATGTTCAAAGGGGTGACCAAGCTTCAATCGTTGGTGGTGCAATTGAATTCGTAAAGG
AATTAGAACACATTCTACAATCTCTTGAAGCACAAAAATTCGTATTATTACAACAACAACAA
GAAGGCGGTACTAGTAATGATAATGATGATTGTGACGGTGGAAAGAGAGAAGTTTCGAAGG
CGGATTATGTTGGGACCCCATTTGCTCAATTTTTCTCGTATCCACAATATACATGTTGTGAATT
ACCAAATAAATATACATCAAAGAGCAAGGCAGCTATAGCTGATATTGAAGTAACTTTAATTG
AAACACATGCAAATGTTAGAATATTATCAAGAAGAAGATTTCGTCAGCTATCAAAATTGGTC
GCTGCGTTTCAGTCATTGTACATTTCTGTCCTTCACCTCAATGTCACCACTTTAGATCCATTG
GTTCTTTATTCAATAAGTGTTAAGGTGGAAGAAGGTTGCCAACTAAATTCAGCAGATGACAT
AGCAGGTGCAGTCCACCACATGCTAAGAATAATTGAGGAGGAAGCAGCTACACTTTAA

>SlbHLH139
ATGACTGAATACAGCTTGCCCACCATGAATTTGTGGAACAATAGTACTAGCGATGATAACGT
TTCTATGATGGAAGCTTTTATGTCTTCTGATCTTTCTTTTTGGGCTACTAATAATTCTACTTCT
GCTGCTGTGGTTGGTGTCAATTCAAATCTTCCTCATGCTAGTAGTAATACTCCCTCTGTTTTT
GCACCATCTTCTTCTACATCTGCATCTACTTTATCCGCAGCTGCGACTGTGGATGCTTCCAAA
TCTATGCCGTTTTTCAACCAAGAAACCCTTCAGCAGCGTCTTCAAGCTCTTATTGATGGTGC
TAGAGAGACGTGGACTTATGCTATCTTTTGGCAATCGTCGGTTGTTGATTTCTCAAGTCCGT
CTGTGTTGGGTTGGGGAGATGGTTATTACAAAGGGGAAGAAGATAAAGCAAAAAGGAAATT
ATCGGTGTCATCACCTGCTTATATTGCTGAGCAGGAGCATCGGAAGAAGGTTCTACGGGAG
CTGAATTCGTTGATTTCCGGGGCACCACCCGGAACGGATGATGCGGTTGATGAAGAAGTTA
CCGACACCGAATGGTTCTTTCTTATCTCCATGACCCAATCGTTTGTTAATGGAAGTGGGCTTC
CTGGTCAGGCGTTGTATAGTTCCAGCCCGATTTGGGTCGCCGGAACTGAGAAATTGGCAGC
TTCACACTGTGAACGTGTGAGGCAAGCACAAGGGTTCGGGCTTCAGACGATTGTCTGTATT
CCTTCAGCTAACGGCGTGGTTGAATTGGGCTCGACGGAGTTGATTGTTCAAAGTTCTGATCT
TATGAACAAGGTTAGAGTATTGTTTAACTTCAGTAATGATTTGGGTTCTGGTTCATGGGCTGT
GCAGCCGGAGAGCGACCCATCGGCGCTCTGGCTCACTGATCCATCGTCCTCAGGTATGGAA
GTTAGAGAGTCTTTAAATACAGTTCAAACAAATTCAGTTCCATCTAGTAATAGTAATAAGCA
AATTGCTTATGGAAATGAGAATAATCATCCATCTGGAAATGGTCAGAGTTGTTACAATCAGC
AACAACAGAAGAATCCTCCTCAGCAACAAACACAAGGATTCTTCACGAGGGAGTTGAATT
TTTCGGAATTCGGTTTCGATGGAAGTAGTAATAGGAATGGAAATTCATCGGTTTCTTGCAAG
CCTGAATCAGGAGAAATCTTGAATTTTGGTGATAGTACTAAAAAAAGTGCTTCCAGTGCCAA
TGTGAACTTGTTTACAGGTCAGTCCCAATTTGGGGCTGGGGAGGAGAATAATAACAAGAAC
AAGAAAAGATCAGCTACTTCCAGGGGAAGCAATGAAGAAGGAATGCTTTCATTTGTTTCAG
GTACAGTTTTGCCTTCTTCGGGCATGAAGTCAGGTGGAGGCGGAGGCGAAGACTCTGAACA
TTCAGATCTCGAGGCTTCAGTGGTGAAAGAAGCTGATAGTAGTAGAGTGGTAGAGCCTGAA
AAGAGGCCAAGGAAGCGAGGTAGAAAGCCAGCGAATGGACGGGAGGAGCCATTGAATCA
CGTCGAGGCAGAGAGGCAAAGGAGGGAGAAATTGAACCAAAGATTCTACGCGCTTAGAGC

TGTTGTACCAAATGTGTCTAAGATGGACAAGGCATCACTCCTTGGAGATGCTATTTCCTATAT
AAACGAGTTGAAATCGAAGCTTCAAAATACAGAGTCAGATAAAGAAGACTTGAAGAGCCA
AATAGAAGATTTAAAGAAAGAATCAAGGCGCCCCGGTCCTCCTCCACCACCAAATCAAGAT
CTCAAGATGTCTAGCCACACTGGAGGCAAGATTGTAGACGTGGATATAGACGTTAAGATCAT
CGGATGGGATGCAATGATTCGTATACAATGTAATAAAAAGAATCATCCAGCCGCAAGGCTAA
TGGCAGCGCTCATGGAATTAGACCTAGACGTGCATCATGCCAGTGTTTCAGTTGTCAACGAT
TTGATGATCCAACAAGCCACAGTGAAAATGGGTAGCAGACATTACACTGAAGAGCAGCTTA
GGGTAGCGTTGACATCGAAAATTGCTGAAACACACTAA

>SlbHLH140
ATGGCTATGGGACACCAAGATCAAGATGGAGTCCCAGGCAACTTGAGAAAGCAACTTGCTC
TTGCTGTTAGAGGCATTCAATGGAGCTATGCAATCTTCTGGTCAACTGCAGTTACACAACCA
GGGGTGTTGAAATGGATTGATGGGTACTATAATGGGGATATCAAGACCAGGAAGACTGTTCA
GGCGGGGGAAGTTAATGAAGACCAGCTGGGGTTGCATAGAACTGAGCAATTGAAAGAACT
TTATAGTTCCCTCTTAACAAGTGAAAGTGAAGAAGACCTGCAGCCACAGGCCAAAAGGCC
GTCGGCCTCATTATCTCCAGAAGATCTCACTGATACAGAGTGGTATTTCCTAGTATGCATGTC
ATTTGTCTTCAATGTTGGCCAAGGGTTACCAGGGAAGACCTTAGCAACAAATGAAACTGTT
TGGCTGTGCAACGCTCACCAAGCCGAGAGTAAAGTCTTTTCTCGTTCTTTGCTAGCAAAGA
GTGCATCTATCCAGACTGTTGTATGCTTTCCCTATTTAGGAGGCGTAATTGAGCTGGGAGTCA
CCGAGCTTGTCACGGAAGATCCTAACCTCATTCAGCAAATAAAAAATTCCTTTCTAGAGGTT
GATTACTCCGTTATTTTGAAGAGGCCTAATTATGTCTCCAACGATGCAAAAAATGACACGAA
TATCGGTAGCCAAAAGCCTGATCATAATGCACTTGAAAATGATGCTTATCCAGTTGAAATAA
ATTCACCTCATGATAGTTCAAATGGTTTTGTCGCCAATCAAGAGGCAGAAGATTCACTGATG
GTGGTAGACGGTATTGGGGAAACTTCACAAGCTCAAAGCTGGAGGTTCATGGATGATAATAT
CAGTAATGGTGCGAATAATTCCTTGAATTCCAGTGACTGCATCTCTCAAAACAATGCAAATT
GTGAAAAGTTGTCCCCTCTTTCGAGTGGAGAGAAGGAAACTAAGCCTTGCCCACTAGACC
GTCAAGAGAACGATCAGAAGAAACCGCATCTTTTAGATCACCAAGGAGATGATGCTCAATA
TCAAGCTGTCCTTTCTACTCTTTTAAAGAGTTCTGACCAATTAACTTTGGGACCACATTTTAG
AAATATGAATAAAAAGTCAAGTTTTGCTAGTTGGAAAACCGATATTCAGATGCCGAGATTTG
GAACTGCACAAAAACTATTGAAGAAGGTACTTCTTGAAGTTCCTAGAATGCATGCTGGTGTT
ATACATAAATTCAGCAGAGAAAATGGTAAAAAGAATAGCCTTTGGAGACCAGAAGTTGATG
ACATTGATAGAAACCGTGTTATTTCAGAGAGAAGGCGAAGAGAAAAGATAAATGAGAGATT
TATGCATCTTGCATCGATGCTGCCAACTAGTAGCAAGGTTGACAAAATATCACTACTCGATG
AGACAATAGAATACATGAAAGAGCTTGAAAGGAGAGTTCAGGAGCTGGAGGCGAGATCAG
CAAGACGATCAAATGATACTGCAGAGCAGACATCTGATAATTGTGGCACTAGCAAATTCAAT
GACATCAGGGGATCACTACCGAACAAAAGGAAGGCATGTGATATGGATGAAATAGAACCTG
AAAGCTCCAATGGATTACTGAAATGTAGTTCAGCTGATAGTATTGTCATCAATATGATCGATA
AGGAAGTCTCGATCAAGATGAGTTGTCTTTGGAGCGAGAGCTTGTTGCTTAAAATTATGGA
GGCACTGACTGACCTACATATGGATTGCCATACAGTTCAGTCTTCCAACCTTGATGGGATTCT
ATCCATTGCTATCGAATCCAAGTCAACCGGATCGAAAACACTAGCAGTCGGAACAATTAGA
GAAGCACTTCAGAGAGTAGTATGGAAATCTTGA

>SlbHLH141
ATGTGTGATTCAATGGCAAAAGAGACACTCAAGAGGCTTTGTAGAAGCCATGGATGGTCTT

ATGGAGTTTTCTGGGGATTTGATCAGACTAATTCTTTATTATTGGCATTGCAAGATGTATACTA
TGAGGAACAAATGGGATCTGTGATTGATGAGATGCTTCTGCAAGTCCATATTCTTGGTCGAG
GGATAATTGGCCAAACAGCATTTAGTAAGAAATATAAATGGATGTTTACAGCTGCTAATCATG
AAAGGCAGATCTCCATTAGATCATCTAATAATTCCAACTTATTCCTGGATGATAATGAGTTTG
AACAACAATTTTCAGCTGGGATCAAGACAATTGCTGTATTATCTGTCGAACCGCTTGGTGTT
CTGCAATTTGGTTCTACTAATAAGCTTCAAGAGAGCACATGTTTTGTGGAGCAAGCAAGAA
CACTTTTCCAGGGGATAGGAGGATCACCGACTTCATCAAGCTGCGAGAATCTGCATTTTGTG
AATTCCACTGTTTTTCCAACTGCAAACAATGAAAGTTTGATGAAGGAATCTCATTTTCTAGA
AAATCTCATTCAATCGGTAACATGCAATGCGGAAAGTCAGATTATGAATTCTGATGTAGCTAC
AGCATTTTTGAGTGAAAACCAGTTTCAGGATGTGAATCAGTTCAATAATTGCAGCAGCCAAT
TTGATACTCAACTACAGCAAGCAATGTTCCCATCTGCTGGTTTGTTTACCAGCTTCCACGATT
CCTGTTTGACGTCAACCTGGGAAGATCTCCCATCAGATATGAGCATACAAGATTTCAGTTAT
GTCCTTCCCACGGGGATAAACCAATTTGAATATGGCACAGGTGCAACTCAAAGCTTTCATGA
CAACACAACATTTGGTTCCTTAGGTGGATTTGGAGTACTTGCAAATGAGGATACTACTGGCC
CTCTAAATGGATACATTGTCCAATGTCCCATCAACCAGAGGAATGATGGAGCAGTTAGTACT
ATCAGTGATAACATATTAGATACGACGGGTATCATCTCAGCCTCTGCTGGTATAAATGAACAG
TTTAGGTTCAATTCAGACAGTGATGCTTCTGTTTCGATTCAAAGTTCTATCACCAACGCCTTT
GAAACTGTTGAAAAAGCAAACTGTTCAAACATGTCTGCCATTGAGAAAATGACTAATTTGG
TTGGAGTTAAACATGATTCTAAAAAACCATGTAACTGGGGTGATGTTTCGAACCCTGTTGTT
AGCACTTCCAACTCTGAATGGACCTATAGTAATGCTAATGAACTCAGATCCAGGCCAGCTAA
CAGGTTATTCTCCAAACTGGGACTAGACCAGTTTCTGGATGGTGCCTTGAGCAGCTCTTATT
CATTTGCTGGATCTTTTTCCGACGGACAGTTGTCAGAAACAAATAAGAGAAGAAGAGTTGG
AAGTTCTTCAGAATGCAATTATCTTCAAAAGCCCCTTGGTTTTTCTAACTTTGATAAGAATGC
AAAGTTGGTGCAGCCTGAATGTGGTCTAGATAGGACAAGCAACCTTGAAGCCAAAAGTGA
GATCATCACAAAATTGGATGCAAGTACATTAATTGGAGACAGATGCAGCATCAATAATTGCA
GAGGCAATGAAAAATCCTCAAAGCCTACCAAGAAGAAGGCTAAACCAGGAACTCGGCCTA
TTCCTAAAGACCGGCAGCTGATCTATGAACGTCTATCTGAGTTGCGAGGGCTTATCCCTAAT
GGGGAGAAGATGAGCATTGATCGCTTATTACACAGAACGGTAAAGCACTTGCTTTTCTTGC
AAGGTGTCACTAAACATGCTGAAGGACTCAAAAAGGCTGAAAGTTTGAAGGACTCAGAAA
CTCGGTTAAACTCCAAGTCTAATGGTAATGGAGTTACATGGGCATGTGAAATTGGCGATCAA
ACAATGGTTTGTCCGCTGATAGTTGAGGACCTTAGTACACCTGGTCAAATGCTTATAGAAAT
ACTCTACAATGAACAAGGCTTCTTTCTGGAGATGGTAGATATAATCCGAGGCTTTGGCTTGA
ACATATTGAAGGGAGTGATGCAGTCACGCGAGACAAAGATGTGGGCACACTTTGTTGTTGA
GGCTGAGGGTAACCGGCTAGTAACAAGACACGAGATTTTTTCATCTCTTGTTCAACTTTTAC
ACTTGACAAGTGCTAGTAAAGTTGGTCTGAATAATCAGCTTCAGTATACATCCGGTGGAAGG
AATACTCTAATAAATGATTGTCCAAACTCTGCTGTGCCGATATCTGGCTGCTTACCTGAAACG
ATCCGATGTGTAAGATAG

>SlbHLH142
ATGCAAGACTTCATCTCAACTTCTTCTTCTTTTTCTTCAACTTCACTACTTCAAAAGCGCCTT
CATTATATAATCCACAATCGTCAAGAATGGTGGGTTTATGGCATATTTTGGCAAGCGTCGAAA
GACGCAAATGGTCGTCTTATTTTCTCGTGGGGGGACGGCCATTTCCGCGATTTAGCGCTGGC
CAAAGTCCATAACGCTAATGTTAGCGATATGGAAATGTTTTATGCGGTGTCAGCGCCAAATT
GTTTTTTGTCGGAGGACGACCTAATCGTCCACGCTTATAATTCTGGTTCCTATGTTTGGCTTA

ATAATTACTACGAATTACAAATTTATAATTACGATAGAGCTAAAGAAGCTCACTTGCACGGTA
TTCGGACTCTGCTTTGTATTTCTACTCCTCATGGTGTTGTTGAATTGGGTTCCTCTCAAGTTA
TCCAAGAAAATTTGGAATTAGTCCAATTAATAAAGTCATTATTTGGACAAATTAATGATCATG
GTTTTAATTTTGTTCCTTTAGGAGATCCTATGGATACGAAAACAATAACTATGGGGTCCGATT
CAGGAAATTCGGATGAGTCTTCGGCTATGAATAAGGATTCACCTAAAAAACGAGCAAGAAA
ATCCACAACAGCGAAAAATCATGTTGAAGCAGAAAGGCAGAGACGGGAAAAGCTGAATCA
TCGGTTCTATGCTCTAAGGAGTGTGGTCCCTAACGTGTCTAAAATGGACAAAGCTTCTTTGT
TAGCTGATGCGGTTACTTATATTAATGAGCTCAAAGCCAAAGTGGAAGAACTCAAGGCTAA
AATTGAGGTGTCAACTAAAAAATTAATTCAGAAGAGAAACTGCGTATCATCATCTGCAGTAG
TTGATGGTACGAATATTAATATTAATATTAATTCATCGTTCGTGGACGGAATGGAGGTGGAGG
TGAAAATTATTGGAGTTGAAGCTATGATTCGGGTGCGTTCACCGAATGTGAACTATCCATGT
GCGAGATTGATGAACGTTTTACGAGAGTTAGAGTTTCAGATTCACCATGCAACCGTTTCAAG
TATGAAGGAGATGATGCAACAAGATGTTGTCATTAGGGTTCCTCATAATGTCACTAATGAAG
AAGCCATCAAATCTGTTATCCTCACAAAGTTAAGTTTTGCTTAG

>SlbHLH143
ATGAATTCGAAAGAAAAGAAAGAGAGAGTTTACTCGTCAGCACCAAAGAAAGTTATGAAG
CTATCAACTGATCCACAAAGCATAGCTGCTCGCGAAAGAAGGCACAGAATAAGCGATCGTT
TTAAAATTTTACAAAGTTTAGTCCCCGGTGGTTCTAAAATGGACACTGTTACTATGTTAGAA
GAAGCAATTCACTATGTCAAATTTCTCAAAACACAAATTTGGCTTCACCAAACGATGGTTAA
TTTAGTCGATATTAATCATGAAATGGTTGGATATTACCCTCTCGTTGATGATGATCAGAATATA
CACAAAAATAATATTAGTTCAATGGACTATCAACAAATGCAACAGGTACAAAGTTATGATAA
CGATGCCTTTCAACAAGTTGAGTTTCCGTTTGAAGAAACTAATATTTCTGGTGATGTTTTTAT
GTACTATAATTAG

>SlbHLH144
ATGAGTGCTGCTTCCTTGAGGCATTTCCTTGAAAGTCTCTGCTTCAAATCTCCATGGAATTAT
GCAGTGTTCTGGAAGCTTCAGCACCAGTGTCCTATCATTTTGACATGGGAAGATGGATATCT
GGATGTTCCGGGTGCTAGAGAACCTTACAGAAGTCAAAATGGCAATTATTATAGCAAAAAC
TTGAGTGACTTATCCCCAAACTGTGGGTCAAGATCACATAATGGATATTTGAGTGCACATTC
AATTGGCTTAGCCGTGGCTGAGATGTCAAGTACTTATCACATTGCAGGAAAAGGGGTGGTA
GGTGAAGTGGCTTCTTTGGGCATCCCCCGCTGGATTTCTTCCGACAGTGTAGCACCTGCAG
AGCTTGGGTTTGGTTCTGTTGCTGAGTGTCCAGATAAGTGGATGCTTCAGTTTGTCGCTGGC
ATTAAGACTATATTGCTGGTACCTTGTATACCTTATGGTGTTCTTCAATTAGGATCAGTGGAA
ACGGTAGCTGAAAACATGGAGATGGTTACCATTCTGGCTGAAGAATTTGATGCCCATCTCAA
ATTTGTGGAGAGCTTTTTACCTGGTGGAGAAAGCTGTGAATTTCTTCTACAATCTACTCTCT
CAGAGACTTTGAATATACCATCTGCAACTACCACAAATAAGGTGAACGAAGATGATGTAGCT
GCTGATATACCAATTGTGGAAGATCACAAATCATCAGCTGTATTTCCAATGACTTCACTAATT
GATGTTCAACATCCCTTTCAGTTATCTGGACAACACATGCAAAATGTCCTCGAGAATGAAAA
TGAAAGCAAAATTGGCAAATTTGTTGAACACATGCCAAATGTTCTTGAAAATGCATATAAAT
GGGAAATTCCAATGCAACATGTGGACATGATTAATCTAGTGAAGCAACTTGCTCATGGATAT
TCTGATGATAATAGATCAGGGATAACCGAAAGAAGCATCGTTAGATCTTCTTGCCATACAAA
AGATATAGATGCCTTTTCTTACTCTAGCTGCAATGTCGGCGGAGTCGGTGTTTCCAATGAGGT
AGATTTTCACTTTGATGGAGACATGCTAGATCCACGATCTCTTGGAATGGATTGCCACAACA

CTATTCTTGGAAATGTAAGTAATTCTTTTAGCTGCTCGACAGAACGTGAACTACACGAAGCC
TTTGGAAGTACAATTCACAACTTGTCTGGCTTTAGTGCAAATCCATCTAGCAAATCCATCTAT
GCTGCAGATTGTACATTTAATAGTGAGCCATCAGATGGATGGCATCTGAAAGAAGATAATGC
AGAGAATCTTCTGGAAGCCGTTGTTGCCAGTGCTTACTGTTTTACTGATGATTACTCGCTCA
ACAAAATGGCTGGTCTGGAATCCTTAAACATGTCATCAGGAAAACCTGTCCCTTCTCGCAA
AAGGCTAAATCAATCTGCAGAAAGTGATTCGGTTGGAGATGCAGTTACACGAAGCACTCTT
ACATCGGCTTCTGCTGGGGTAGACAAATATGCTTCGACTAACCGTCCACATTCTGCTTCTTC
ATTTGATTATGTTGTCAGTACATTTGATGAGGGACATCATCAGACAAAAGTCTTCAGCTCTTT
GGATTGTCACAAGGAGTCAAAAATATCTAATACTAACAAGAAAAGGAGACGGTCTGGTGAT
AGTCATAAGCCCAGACCACGAGATAGGCAGTTAATTCAGGACCGGCTTAAGGAATTGCGGC
AACTTGTCCCTAGTGGTGCCAAATGTAGCATTGATGGTTTACTAGATAAGACCATAAAGCAC
ATGCTGTTTCTGCGAAGTGTTACTGATCAGGCTGATAAATTAAGGTTCCAGGCTCAGACAGA
GGTAGCCCCTGACAAGAACCTTCAGTCACCTCCAATAAAATCTAGCAATCAACAAGGAACC
TCTTGGGCTCTAGAATTGGGAAGCGTGGACCAGATATGCCCAATAATTGTCAAAGATTTGGA
ATACCCTGGACACATGCTTATAGAGATGATGTGTGACGACCATGGACGTTTCTTAGAAATTT
CTGATGTTATTCACCGTTTGGAGCTGACGATTTTGAAGGGCGTGATGGAAAAGCGTTCAGA
GAGCACCTGGGCTCACTTCATTGTTGAGGCTTCAGGAAGCTTCCATAGACTGGATATCTTCT
GGCCACTGATGCAGCTCCTACAGCAAGTGCCAAGTTCTGTTTCACGCAACATCTAG

>SlbHLH145
ATGAATGGAGGCGGCGAGAGTAATCATGTTTTCTCATGGGAAATTGATGATGTCTTGTCATAT
CTGAACTTGAATGATAATCAAATTGGGAGTGGAACGACGTTTGAGGGTGACATGATGACAA
ATCCAATTAGATTTGATACTTATCAGTCGTTAACAGTTGTTAATGAGGTGGTTGAATCGAGTA
CTAATGTTGCTAAGAAGAGAAGTCCACCCAATCTAAAAAAGAACGGTAAGGGAATTGTTGA
ACCAAAATCCAGTGTTGATGGAGTCAGAGATAAAGGAAGTTTAGAGCATGAGATACACATA
TCGACAGAGAGAAAAAGGACAAAGAAAATTTGGATCTTGTTTGAGACTCTCCGTGCTTTGA
TTCCTAATATCTCTGCTAAGGCTGATGAGTGCACCATTATTGATAAAGCAGTGAACCACATCC
TAAAGCTGAAGAATACTTTTGAGAAGCTTAAACGGGAAAATCTAGAAGGGTTTCAAGAACA
TAACATAAGATTGATTAGTTCACAGAAATTCCCAGATGTTGGTAATAGTTGGGAGAAATTATT
TGGGTGA

>SlbHLH146
ATGAAAAAGAAAAAAATCAATGGAGGTGGTGAGAATAATCATGTTTTTCCATGGGAAACTA
ATGATGGATGGCCGTACCTAAACTTGAATGGCAATCAAATTGGGATTGCAGTGACATTTGAG
GGTGACAAGTTACAAGATCCAACTAGATTTGATACTTATCCACCATTGACAGTTGTTAATGA
AGTGATTGAACCAAGTACCAATGCTGGAAAGAAGAGAAGTCCACCCAACCGAAAAAAGA
ACGGTAAGGAAATTGTTGAACCAAATTTTTGTATTGATGGAGCCGGAGATAGAGGAGGATT
AAATCATGATTTACACATATGGATGGCGAGACAAAGGACAATGAAAATTGGAATTTTTTTCA
ACACTCTTCGTGCTTTGATTTCTAATATCCCTGCTAAGGCTGATAAGTCTACAATTGTTGAGA
AAGCAGTAAACCACATCCAAAAACTGCAGAATACTTTGGAGAAGCTTGAACAGGAAAAAC
TAGAAAGGCTTCAAGAACATAATGTAAGGTGTAGGAGTTCACAAAAATTCACTAATACTTGT
AACAATTGGGAGAAATATCTGGGTGATCAAGGATCAACACATAATACATCTTCTACTACATC
AACAACTCATGGTACCAATCCCCTTATGGTGAATAATAACATTCCAACAGGTTTTGTGACAT
GGAGTTCACCAAACGTGATACTTAATGTTTGTGGTGAAAATGCACATATTAGTGTGTGTTATC

CAAAGAAGTCAGGGCTATTCACCTTCATTTGTTATATTTTGGGGAAACATGGGATCGAGATT
GTGTCTGCTCAAGCTTCATCTGATCAATTCAGAAGCGTGTTCAAGATCCAAGCTCACGCTAA
AGGTGGAACTGGCATAGCTCAATACTCTGAAGCATCCAGAGTTGAAGAAATGTACAAGCAA
ATTGCGATTGAGATAATTTCATTTGCAACCCCCAAATGA

>SlbHLH147
ATGGGATCCAAAGAGCAAAAAAAAGCATTGCTCAATCAACAATTGGAGCAACTTGGTTCAA
TTACTAATTCCCCTTCTGTGAATAAGACATCAATCATTGCCCATGCATCGAAGTATATAGAAG
AATTAAAGGAAAGGATTGACAAGTTAAATGAAGATGTTTCAACTTCACAACCTTATCATCAT
GACCATGAAGATGCCTTACCAGAGGTTATCGTAGAAACCCTAGAGAAGGGGTTCATAATAA
ATGTATTTTCAGAAAAGAATTGCCCTGGTTTGCTAGTCTCAATATTGGAAGTCTTTGAAGAG
CTTGGCCTTGAATTATTGGATGCTAGGGTTTCTTGTTCAGATTGTTTTCGACTTGAAGCTGTT
AGTGATAAGGAAGGAGAAATTGAATGCATAGATGCCCAAATGGTAAAACAAGCAGTGTTGG
AAGCTATAAGGAATTGGAAACAACTCAATCACCAAGATTAA

>SlbHLH148
CTATCTTTTTTCTCTCTCTCCAGTCTCCACCTATATAACTTCCTCTCTCTCTATTTATTTCACTT
CCCTCTCCCTAATTTTTTTCTTAATATTTTTAAACTTCAATTTAATCATTCATTTTCCATGGAAA
AAGACTACTTCATCAATGGAGGAATCCCTAATCCAGCCCTTCAGTTTGAACCTACTATGTCAT
TCCCTTCTTGGAATCCCTTACATTCAGGGCAATCTTTGTTTAACCCCAACTGGGATCATTCAA
CACCTCAATTTGACTCAACTTTGAACTCGATTGTATCTTCTTCTCCTGCTGAGTTGATTGGAA
AACTGGGTACTGTTTGCAGCTCCCCTCAACCGATTTTGCACAATAACAGTTATAGTAGACCC
ATGATGGGAAAAGATAATATACCCAATTTGGGAAGTTCACTTCCTCCTCCATTACCTGCTGAT
CCAGGTTTTGCTCAAAGAGCTGCTAAATTTTCCTGTTTTGGGAGTCGTAGTTTCAACGGCAG
AACGAGTCCGTTGGGGTTGAACTATACGGAATTATCACATAGATCTGCTCAAACATTGGGGA
ACGGAAAGTTGCCTAGAGTTTCGAGTAGCCCTTCTCTCAAGCAAGGTGGATCCCCTTTGCA
ACTCAAGAATTCGGGTCAAACCCGGATGGAAATGATGTCCAATAACTCTAACGAGTCTGTTT
CAGAGCCAAGTGGGGAAACAGCATCGAAACTTCCAACTGAATTGAATTCTAGTTCTAGAAA
AAGAAAAACAGTTTCAAGAGGGAAAACAAAGGAAGATTCCCCAACTGAAGGAGTTAATG
GAAACAGGGGAAGTGAAGCTGATGATAATGCAAGAGCAAAGCGATGTAAGCAAGTAGAAA
GTAATGGGATAGAAAATGGAAGAGTACACATGGAAGAATCAAAGGATGATGAAACCCAAA
AACAAGTTATGGAATATCAAAAGCCTACTGAACCACCAAAGGACTATATTCATGTTAGAGCA
AGGAGAGGACAAGCTACTGATAGCCATAGTTTAGCAGAAAGAGTGCGACGGGAGAAGATT
AGTGAAAGAATGAAGCTTTTACAAGATCTTGTACCTGGCTGTAACAAGGTGACAGGAAAA
GCACTAATGCTTGATGAAATTATAAACTATGTACAGTCACTCCAACGACAAGTCGAGTTCCT
ATCCATGAAGTTGGCAACAGTGAACCCAAATTTGGATTTCCCCCTCTCCAAGGATATATGTC
AACCAAATGGCTCTGTGGCCCATCCTGTTTTCCCAATAGATAAAACATCATCTTCTTACCAAC
AAGGCCGGAGCGATATTCCTAATGGAGCACTAAGTCAATGCTCAGTGGACACATCAGATAA
CTCACTATGCCGTAGCCTGGGAATGCAATTGCCTCCCCTTGATGGATTTGCTGAATATCTTAA
TCAGTTCCCTGAGGATGACCTGCAAAGCTTTGTGCAGATGGGATTTACCCAAAACCCCAAC
AAGGATATGACACTGCAATCACAAGGGCCTCATCAAACGTCTCACATGAAAATTGAGATGT
AG

>SlbHLH149

ATGAATCATTCTGTTCCTGATTTTGATATGGATGATGACTTCTCTCTTCCTGCTTCTTCTGGTA
TTACCAGAACTAAGAAGTCCGCAATGGCGGAAGAGGAAATCATGGAACTACTGTGGCAAA
ATGGTCAAGTGGTTATGCAGAGTCAAAATCAAAGATCTCTTAAGAAACCTCACATCGGTAA
CGGCAGTGGAGGTGGTGGTGATGCGGTTATTCCCTCTGACCAAGCTGTCAGTAGAGAGATC
CGTCATGTAGAGGAAACTACTCCACATCATCTGTTTATGCAGGAGGACGAGATGGCCTCGTG
GCTTCATTACCCACTCGATGACCCCTCCTTCGAACGTGATCTCTACTCCGATCTCCTATATCC
CACGCCCACTTCTACCTTCACCACCGCCGCGCTGCCACGAGAAAACCGTACGTCTACGTTT
GAGATCCGTCCACCTCCGCCACAACCATCGCCTGCAGCGCCGATCGGAACGGCTCCTCGAC
CGCCTATACCTCCTTCTAGGCGCACCGTCACAGAAAATTCAAATCGATTCCAGAACTTCGGA
CACTTCTCGCGATTGCCTAAAGCAAGGCTAGAACCTGGTCAGGCTAACTTAAGCAAGTCAC
CAAGAGATTCAACGGTCGTGGATTCAAACGTAACTCCAATTACAGGGCAGGAATCTAGAGT
TACACTCATACCTGATAATGTGGTAGCAGTACCCGGCGGAAATGTAGGATGTAGTACAGTAA
ACGGCAGCACCGGAACTGCGACAGCGTCAACGGCAATTAGGGAACCGACGACTACATGTG
ATATTTCAATGACGTCATCTCCGGGTGGCTCAGGAAATAGTGTAAGCGCCAGTGCCGAACC
ACCAGCACCAGCACCAGCACCGTCGCATAAGGGGGCGGCACCAACGGCAACGGCAGCAG
CGGATGATCGGAAACGGAAAGGGAGAGAAATGGAGGACGAAGGTCAGAATGAGGATGCT
GAATTTGAGTCTCCTGATACGAAAAAGCAAGCACGCGGTTCAACATCCACAAAGAGATCTC
GTGCTGCGGAGGTCCATAATCTTTCAGAAAGGAGACGTAGAGACAGAATAAATGAGAAGAT
GAAGGCCCTGCAGGAACTCATACCACGCTGTAACAAGACGGACAAAGCTTCAATGCTTGAT
GAGGCAATTGAGTATTTGAAATCACTACAATTGCAAGTGCAGATGATGTCCATGGGATGTGG
CATGGTCCCGATGATGTATCCTGGAATGCAGCCGTACATGCCGCCAATGGGAATGGGCATGG
GTATGGGGATGGGGATGGATATTGGCATGAACAGGCCAATGGTGCCATATCCACCTCTATTAC
CAGGTACAGCGATGCAGAATGCAGCTGCAGCAGCACAAATGGGTCCTAGATTTTCTATCCCT
CAATTTCATTTGCCACCAGTTCCAGTACCAGATCCATCCAGAATGCAAGCCTCAAGTCAGCC
AGATCCAATGCTAAATTCACTTGTTTCACATAATTCGAACCAGCCAAGACTTCCGAATTTTA
GTGATCCATATCAGCAGTTTTTTGGTCTCCAGCAAGCGCAAGTTGCATTACCGCAGAATCAG
GCAGTAGAACAGCCCAGTAATAGTAAATCCGGCAGCAGCAAAGAAGTTGGAAATCCAGGC
AATCATCAATCT

>SlbHLH150
ATGGGAGAGCAGAATCAGCCTAATTTCGTAGGAACACAATGGGCATTTCTTGGAAGTACAA
ATGATTCTTTTTATCTAGCGGGCCATGAATTTAACAATCAGTACATGACTGAAGTTGGAATTG
ATGGGATCCATTATGTTCCTGTGCCTTCACCTCAGTGGATCCATGACCAGCAAACACCACAT
AATTATGTCGAATATCTTGCAGAGAATGTGGTCTCTGAGGTAGATGCTTCGGTAGAAGCTAC
TGGTTTCAGCCTACTCGAGGGCTCTGCTGACATTATTTCTCAGGAGATAAGATATCCGAGGC
CTTATTATGAAGGTGTGCCACCCAACTGTTTAAATAGCTCAGAACATCAACGAGGTGCCTTG
ACATGGGAGTTAAGAGGAAATGTCAGGCATGCTTCTGGGCCTTGCTCTGATGCATCAGCTG
CTGGGTCATCGTATTCTTCCAAAAGCTCTAGAAAAGCAAGAGCTGCATTATCTGATCGGCAT
CGCAGGATGAAAATTGCTGAAAGAATAGATGCATTAGGAGAACTGTTTTCGTGCTCTAAAC
AGGGTGGAAAAGCATCTCAAATGGATGAAATTATTGATCATATCAAATATCTGCAGTTCCAG
ATGAAGGATCTCAGTAGGAGCAGATTGGGTGGTGAACCTACTTCTATCCCGTTTGTATTTCTT
GAGGGATGTGGCCATTACATTCTTGATGAACAGCAGATTGAACCTCTGGAAGACACAATGG
GAAAGTTGCTGGAAGTGAATCCTTCATTGGCAACCCAATTGCTGGAGAGTAAGAGCCTGTT
TGTGATGCCTTTGGCTCTGGCAGAAGGACTGCATCATCATGAGTAG

>SlbHLH151

ATGGAGATTATACAGCCTAATAGCCTGCAGTTACAAAACATGTTGCAAAATTCCGTCCAGTC
GGTTAAATGGACTTATAGTATTTTTTGGCAATTTTGTCCAAAACAAGGGGTGTTAGTGTGGA
GAGATGGATATTATAATGGAGCTATAAAGACTAGAAAAACTGTGCAGCCAATGGAAGTTACT
GCTGAAGAAGCTTCTCTTCATAGAAGCCAACAACTTAGAGAACTTTATGATTCACTTTCTGC
CGGTGACTCAAATCCCCCGGCGAGAAGGCCGTCCGCAGCATTGTCGCCGGAGGATTTGACG
GAATCTGAGTGGTTTTATCTCATGTGTGTTTCTTTCTCTTTTCCTCCACCTATTGGATTACCAG
GCAAGGCTTATTCAAAGAAACATCACATATGGATAATGGGAGCAAATGATGTTGATAGCAAA
GTCTTCTGTAGAGCTATTCTTGCTAAGACTGTAGTTTGTATTCCTCTCTTGGACGGTGTTGTG
GAACTCGGAACTATAGAAAAGGTTCAAGAAGATATTGGATTTATACATCGCGTAAAAAGTTT
CTTCAATGAGCCACAACAAGCTCAGCCACCAAAGCCAGCTTTATCTGAGCACTCCACTTCG
GATCCCGCCGCCTTTTCGGAGCCACATTTTTACTTCAGCAATACTCCGTCATCTGCGGGTATT
TGTCCAGCGGATCAAGACGGTAGAATTACTGGAGAAGAAGAAAATGAGGACGAGGACGAG
GACGAGGCTGAGGATGACGAGGATGAAAATGATGAAGCTGAATTAGACTCGGATGGTATAG
CAATTCAAAGTGGGGCTGGAGCGGCTAATCCTATGGCGGCTGAGGCTAGTGAGCTCATGCA
GCTTGATATGTCTGAAGCTATACGGCTCGGCTCACCGGATGATGGCTCTAATAATATGGACAC
TGATTTATATTTGGATGGTATTAGCCAAGCTGGAAATACGGCTGACTCTTTCAAAGCTGAGA
CTGCAATTAGTTGGGCTAACTTCCAAGACCTTCAACATTTACCAGGTATACCTAGTTATGATG
AATTATCACAAGAAGATACACATTATTCTCAAACAGTTTCAGCTGTACTTGAACACCTCTCG
AACACAAGTTCCAAGTTTGCCTCTTCTGCTACCATAATGGGCTCTATTTCTCCTGATTCAGCC
CAATCCGCTTTCACATTATGGCCCGTCACTTGTAGCCCAAATCTCTCCCACTGTCGTCGCCA
CGATATCGGCGATGGCAGTGGGACCACCTCTCAATGGCTGCTCAAAAGCATACTATTCACTG
TCCCATTTCTCCACAGTACTAAAAAATTATCAGAAGCTCTGTCTCCAAAGTCACGAGACGCT
GCTGCGGCCGACTCTTCGGCCGCAGCGTCTCGATTCCGCAAAGGATGTACGATAAATAGTT
GTACACAACAAGAAGAGACGAGCGGGAACCATGTACTAGCGGAACGACGTCGTAGAGAGA
AGCTGAATGAGCGTTTTATTATTTTAAGGTCCTTGGTACCCTTCGTTACGAAAATGGATAAAG
CATCGATACTTGGCGATACAATAGAGTATGTGAAGCAGTTACGTAAGAAAGTTCAGGATCTC
GAAGCGCGTGATCGCCACACGGAAATTACCAAAAAATCAGATGAAAAGAGTGGTTCACCA
ATAGTAAAGGCGTTTCCGGTGAAGGGTAAGAGGAGAATGAAGAGTACGGTGGAGGGAAGT
ATAGTCGGAGCACCGGCAAAGATGACGGGTTCTCCACCGATGGAAGAGGAGGTTTTGCAA
GTGGAAGTCTCGATCATCGAAAACGATGCACTGGTGGAGCTCCGGTGTCCGTACAAAGAA
GGGTTGTTGTTAGATGTAATGCAAGTTCTAAGGGAACTAAAGGTGGAAGTTGTAGCAATCC
AATCATCTCTTAGTACTGGACTCTTATTGGCTGAGTTAAGAGCTAAGGTAAAAGAAAATATAT
ATGGAAGGAAGGCAAGCATATTGGAAGTGAAAAAGTCAATAAATCAGATAATCCCTAGAGT
TAATTAA

>SlbHLH152

ATGGATGATGTTTCAGCACCTGGAAGGAGGTTTAGAGATGATGTTGTTGATAGCAGAGCTTC
TTCTTCTGTCAAACCTGGTTTTCATGGACAACCGATGCCTTCAATGCCGCATCCCCCTGCAA
TACGTCCAAGGGTGAGAGCTAGGCGAGGACAAGCTACTGATCCACATAGCATAGCGGAGA
GGTTACGTAGAGAGAGGATAGCAGAAAGAATTAGAGCATTGCAAGAGTTGGTTCCCAGTGT
CAATAAGACTGATAGAGCTGTAATGCTTGATGAAATTGTAGATTATATCAAATTCCTACGGCT
GCAAGTGAAGGTGTTGAGCATGAGTAGGTTAGGAGGAGCTGGTGCAGTAGCACCACTTGTT

ACAGACATTCCAATATCATCAGTAGAGGAAGAGAGCAGTGAAGGTGGAAATAATAACCAAC
CAGCTTGGGAAAAGTGGTCAAGTGATGGCACAGAAAGGCAAGTGGCTAAACTCATGGAAG
AAAATGTTGGTGCTGCAATGCAATTTCTTCAGTCTAAAGCACTATGTATAATGCCTATTTCTC
TTGCATCAGCAATTTATCACTCTCAGCCACCAGATACATCAAGTCTTGTTAAGCCAGAAACA
AATCCTCCTTCATAG

>SlbHLH153
ATGGCAAGCCAACTGCAACAAGCACTGAGGAGCCTTTGTTGCAATACTCCTTGGAAGTATG
CTGTGTTCTGGAAGCTCACACATCGAGCTCGAATGATGTTGACTTGGGAGGACGCTTACTAT
GATAACGATGGGTTTCCAGGGAAAAAATCACCTGATAGTACGGCAGGCAACCTTTATGATG
GACATTATTCCAACAATCATCTTGGAGTAGCTGTGGCAAAGATGTCCTATCATGTTTATTCTC
TCGGAGAAGGTATTGTTGGGCAGGTTGCAATTACTGGAAAACATCTATGGCTTTCAGCAAA
CAAAGTTGCAGCTATCACCAACTTGGCGCCTGAACACTGTGATGGGTGGCAAGCTCAATTT
TCTGCTGGGATTAAGACCATTGTTGTTGCTGCTGTTGCTCCACATGGAGTTGTACAACTTGG
ATCCTTGGATAGTATCCCTGAGGATTTGAGGGCGATCAAGCATATTAGAGATGTCTTTTCTGA
GCTCCAGGAGTTGATGACGAGTTGCTTGCGGAGTTCGATGCAGCACAGCATGGAAAATTCT
TGCTTGTCAGAGATATCGACAAGAACCTCAGGTTCAGAGATTTTTCAAGACTGCGTAAATAA
TCTAGGTAGAAGTGTTTGTGAGGATCGGAGAAATATGTGGTCTCCTCTATATACATCTTTTGA
AAAATCTGTTGATCATTCTTGTATCTTCTTGCAACCTGGAGGTTACCCAAATAAAATACTTGA
AGTGGTAAATAACCAAAGACTTCACAGGAGTTCAGTTCAAGGATCTGATGACTCTACAAAT
CTGTTTTGTGCTGGCTATGAGCTATACGAAGCATTAGGGCCAGTTTTCCAGAAAGGGAATTC
TTCCAAGGACTGGGAGGCAGGGAAACGGAAGAAATGGCTGTTGACATGCTTGAGGGTAT
TGGCACCAGCAGTCTAGTAATGAGCAACACTGGCAATGAGCATCTTCTGGAAGCAGTAATA
GCTAATGTTAACCGCCATGACAATGATTGTAGCAGTGTCAAGTCATTCTGTAAATCTGTTGAT
TCCCTCTTGACCACTGAAATAACTGCCGAACCTTGTAGCAGTGATATTGGCACAATCAGTTC
CACAGGCTATTCATTTGATCGGGAAACATTGAACAGCTTCAACTCATCAGGTACATGTAGTA
TTCGGTCTTCTAGGGGTCTTTCATCAACCAGTTGTAGCAGAGGTAGTGGACATGTTGAGAG
GCCACTTGAACCCGTTAAAATGCATAAAAAGAGAGCTAGACCTGGTGAAAGTTGCCGACCT
AGACCCAGGGATAGACAATTGATCCAAGATCGCATCAAGGAGCTCCGTGATCTTGTTCCAA
ATGGTTCGAAGTGCAGTATAGACTCACTTCTAGAACGAACAATCAAACACATGCTCTTCATG
CAAAGTGTAACCAAGCATGCTGACAAGCTAAGTAAGTGCTCTGCATCAAAGCTGGCTGACA
AAGAATCGGGTATCTGTGGATCTTCCTCCCATGAGGTTGGTTCAAGCTGGGCAGTGGAAGT
TGGAAATAACCAAAAAGTTTGTCCGATGAGGGTTGAAAACTTAGGCATGAATGGTCAAATG
CTTGTCGAAATCTTTGAAGACGGAAGCCATTTCCTTGACATAGCAGAAGCCATCCGGAGCT
TGGGTCTTACAATTTTAAAAGGTCTGGCTGAGGCTTATGGTGAGAGGACACGCATGTGTTTT
GTGGTTGAGGGGCAGAATGATAGAACCCTGCATCGCATGGATGTATTATGGTCTCTTATGCA
GCTACTGCAGGCAAAGATCAACTTGTAG

>SlbHLH154
GTCAGCACAAATACTTCAATTATTATGCCTCAGTTTCAGAGAAGTAGTGATTATTTATGTAAA
TCTTTGATTAGTACGAAAAGTACTTCAATTATTACGCCTCAGTCCCACACAAGTTATGATCGG
CTGTATAAATCTTCAGTAAATAGAAGTAGTACATCAATTATTATGCCTGGTCAACTCCAGACA
AGTTGTTGTGATCGACTATATAAATCTTTTACTAAGGAGGGGTTAAGAAGTCAAGATGAATC
AAATAAGCTCAAGAAAGCTGTGCATAGAAATATTGAAAGGCATAGAAGGCAAGAAATGGCA

AATCTTGTTACTTCTCTCAGATCTCTAATTCCCATTGAGTTCATCAAGGGTAAACGTTCAGCA
TCAGATCATATGCATGCAGCCGTGAATTATATAAAGTATCTTCAGAAGAATATACAAGAGTTG
GATAATACAAGAAAATGTATTATTTCATTGGATAATCAAAATAAAAGTTCAACAATTGTTGAT
ATTTTTCAACAAAATAATAATTGTGTCACAGTGAATAAATGTGAAGATGGTATGGAAATTCTG
ATTAATGTCAATAATAGCAATAAAGAGGATATATTTTCTTTATCCAAAGTGTTGAGATGGCTA
CTTAAACAAGGGCTAAATGTGGTTAGTTGTGATAATTCCAAAAAAGATGAAAGGACTTTGAT
CAGGATCCAATGTCAGGTGAGTGATATATCATCAGGTCTCACTGTAGTTGGGTTGCAAAAGA
AACTGACTGATGTGATTAATTAA

>SlbHLH155
ATGGCAGCAAACCAACCGGAAGGCTATGCCGACGATTTCCTCGAGCAAATTCTCGCTATTCC
TCCCTACTCTGGCTTGCCGGTTGCTGATGTTGGCACTCCATCAGAGACGACGTCGTTTACCT
CGGCGTCTGCCGTATCTCATCTTAACTCTGCCGCTGCTGCTGGCCTACAGCAACCTTTGTTT
CCGCTGGGATTAAGCTTGGATAACGGCCGTGATGACGTCGGCGATGCAGGTCCTTATGCAGT
GAAGCATGAAAGAGATGGAATGAATATCGGAAATCTATATGCAGGTCTAGAACACTTGCAAT
CTCATGCAGTTCGTCACTCTGTGCCTTCTGTTCACCATGTCCAGCCTTTTCAAGGCCCACCA
ACAACGAGCACAACAGTAACTGTGCCACACCCACCTTCAATTCGTCCTAGGGTTCGAGCTC
GGAGGGGACAAGCCACAGATCCACATAGCATTGCTGAGAGGCTGAGAAGAGAGAGGATAT
CAGAAAGAATAAAGGCTTTGCAGGAACTGGTACCCAGCTGCAATAAGACAGATAGGGCCG
CAATGCTTGATGAGATTCTGGACTATGTGAAGTTCTTAAGGCTTCAAGTTAAGGTACTGAGC
ATGAGTAGGCTGGGAGGAGCCAGTGCAGTGGCACAACTTGTTGCTGACATTCCATTACAAT
CTGTGGAGGGGGACAGTGGTGAAAGTAGATCCAACCAGCATATATGGGATAAGTGGTCCAA
TGTTGACACAGAACGAGAGGTTGCTAAGCTCATGGAGGAAGACGTCGGTGCAGCCATGCA
ATACCTTCAGTCTAAATCACTCTGCATCATGCCCATATCACTTGCTGCACTCATCTATCCTACT
CAACAACCGGATGACCAGTCCCTGGTCAAGCCTGAAGCAGCAGCCCCATCATAG

>SlbHLH156
ATGGAAGAGTTCTTTCAAGTTTCTTGGTTTTGTGATGATGAAAATGAAACTAATAATAATTTT
GTGGTGAACCAAAGTGCTTTTGTGAGTTTTGGGAGCAAATCAAATGAAGGATTTGGAGTTT
CTAGCTATGGAAATGTATCGATGAATCATCGAAACATGAACAAGAGGATGATTGAGTTCTTG
AAGAAGAATTGGAGTCCCAAAAATGGACAAGTGAAGATAGAAAAAGAGAAAGTTCATAAA
CATATGATTAAAGAGAGGATTAGAAGAGAAAAACAAAAGCAAAGTTATTTGAACTTGTACA
AATTGCTTCCAATGGGTACCAAGAATGAGAAAAATGCTATAGTCCAAACAGCAACAAGGAG
AATTGAAGAGCTACAAAAATACAAGGAAAATTTAGAGAAGAGAAATGATGAAATTCAATTG
ATTTTAGCACAAAGTGATAAAAAAGAGGAGGAATTTGAGAAGGCTAAAATCAAAGCAAAA
GTTGGTAATCCAATTTGTGGTGTAGATTCAATGCTAGAGGTTCTCAAGTGTTTGAGGAATTG
TGAAACCAAGGCAAATTCCATTCAATCAAGTTTCTCTCACCAAGAATTTTCAACATTGATTG
AAATAGAAACTAAGAGTGGAGCAGCAGAGATAGAAAAAGCAGTACAAAATACTCTATTTGA
AGTTGAAAGGAACCTTCGTGCCCATTGA

>SlbHLH157
ATGGATCGATTTGATTCATTTCGAGATGCCAATTGGGACCTAATTGATTTCAATAGCTTTATAG
ATGAAGAATCGCCGATAGATTTCTTTTGGAACGATCAAACCCAGGATTTGAGTGCTGTAGCA
GAAGTCGAGGCACCTCTCAGCAGTGCTGCATTACAGGAGTGCATAGAAACAGAATGCCCTC

GGAAAAGAGGTCGTAATGAGTCTTGCAGCAAACAAGGGAACAAAGCTTGCCGTGAGAGAT
TAAGAAGGGAAAAATTGAATGAAAGGTTTTCAGACTTATGCTCTGTTCTGGAACCCGGGAG
ACCTGTAAAAACAGACAAAATGGCCATACTTGGTGATGCCATTCGTGTTCTAAACCAGCTG
AAGACTGAATCTGAGGAATACAAAGAGATGAACCAAAAGCTTATGGAAGAGATTAAGACTT
TGAAGGAAGAGAAGAATGAACTTCGTGAAGAGAAACTTGCACTGAGGGCTGACAAAGAG
AGGATGGAGCAAGAGTTGAAAGCTACAGCTTCTCCAGCAAGTTTTATTCCCCCTCATCCAG
CAGCATATCAGCCGGCTGTAAATAAGATGGCCGTTTTCCCTAGTTATGGTTATGTGCCAATGT
GGCAGTATCTACAACCATCTTCGCGGGACACATCTCAAGATCATGAGCTCAGACCTCCTGCT
GCTTGA

>SlbHLH158
ATGGTTAATATTGATGAAGAAAAGGCCTTGATGGTGTCACAAAAAGAAGAGGAAATTAATG
TGACATTTGTTGGTGAAAATAGCAAAGGAAATGAAAGCAAGAAGAAGAGAAAAAGAACA
AAAATAAGAATGAAGAGTAGTGAGGAAGTTGAGAATCAAAGAATGACACATATTGAAGTTG
AGAGGAATAGAAGAAAACAAATGAATGAACATCTTCATGTGTTGAGGTCTCTCATGCCAAG
TTCATATGTACAAAGGGGTGATCAAGCTTCAATTGTTGGAGGAGCAATAGAATTTGTAAGAG
AATTGGAACAACTTTTACAATGTCTTGAATCACAAAAAAGAAGAAAACTCTATGGAGATAA
TCATCAAGATTCATCATTATTAATGGAAATTCAAAATCCATCTAATAATTTAGTATATGCTCCA
AATAACAATGAAATTGGGATTATTCAAGAAGATATAGCTGAGATTAAGTCATGTTTGGCTGAT
GTTGAAGTGAAAATTATTGGTATTGATAATGCTATGATAAAGGTTTTGTCAAAAAGAAGGCC
AGGGCAACTCATTAACACTATTTCTGCATTACAAGATTTGCAACTTAACATTATTCATACAAA
TGTTACAACAATTGAACAGACTGTTTTGTATACTTTCAATGTTAAGATTTGTGGTGAAACAA
GGTTTTCAGCTGATGATATAGCAAATTTGGTGCAGCAAATATTCAGTTTTTTTCATGCAAAT
AG

>SlbHLH159
ATGGAAAAGGGAAATTTGTTCATAAATGATGATAACACTACCTATGAACATCTTCAAAATTG
TTTCTTTAATCCCAATTTAGATAACAACAATTCTGATCCATTTGAATCAGCATTGAGTTCAAT
GGTATCTTCTCCTATTTCAATTCCTAATAATAATAGTGGTAGTGATAATTTTGTATTAAGGGAA
TTAATTGGTAGACTAGGAAGTATTTGTAACAATACTAATAATAATAGTAGTAGTACTAATAATT
CTTGTTATAGTACCCCTTTAAATTCTCCACCAAAATTGAATCTTTCTATGAGAGGTAATTTACC
ACCAACACAATTTACTACTGATCCTGGATTTGCAGAAAGAGCTGCTAGATTTTCTTGTTTTG
CTACAAATTTAGAAAGTAATCATTCCATCAAGATTCAAGATGTCAATTTGGTTCAAAGAAAT
TCTGAATTTGGAGATTCAAGAGAAAATTCATCACTTTCTGAGCAAATGATTGGTCAAAATGA
TACAAATTCAAGAAAAAGAAAATCAATTTCCAAGGGAAAATCATCCAAGATTGTCAATGAT
AAAAATGAATCAAATGCCAAAAGAAGCAAGTCTGAGGAAAATGAAAATAAAGTAACAAAA
AAAGAGGAAAATGCTGTTTTAGAAGAAAATAAAGATAACCAAAAGGCAACAGAACCACCA
AAGGACTATATTCATGTGAGAGCTAGAAGGGGACAGGCCACAGATGCACATAGTCTAGCTG
AAAGAGTGAGAAGGGAGAAAATTAGTGAAAGAATGAAGCTTTTACAAGATCTTGTACCAG
GCTGCAATAAGGTGACTGGAAAAGCTGTAATGCTTGATGAGATTATTAACTATGTACAATCA
CTTCAACGTCAAGTTGAGTTCCTTTCAATGAAATTAGCTACTGTGAATCCAAGAATGGACTT
TAATATGGAAGCACTTCTTTCCAAGGATATGTTTCAATCTAGGGGATCATTGGGACATAACAT
GTATCAATCAGAAACATCAACCCAAGCATTTCCTTATGGATTTCAATCTCAACCAAACCAAA
ATTATCATAAAGGAACAGAGTTTCCTTTCCAAATTAACTCATTGAATCCTAATTTGATCAGAA

ATTCAAGTATGCAATTGCCTCCTCTAGATGGTTTTGTTGAGCCTACCCCTCAGGTCCCCACAT
TCTTTGAGGATGATCTCAATAGTGTTGTTCAAATGGGATTTGGACAAAATCAGAACCAAAGT
TTTCCGGGTGTAGCAGGCAATGTACCTAATTCTCAAATGAAAGTTGAACTATGA

>SlbHLH160
CTGATCCACTCTCTTTCTCACAAGACTTTTCATAAAACAAGTGTAGCACTTTTTCATTTTTTT
AATTCCCAACTTGCTCTCTTCCCTCACCCCACCCACCCACCTCTCTCTCTATTAACTTCTCAA
CCTCCAAATCTTAACAATCCAATTCGAACAAATTATCAAATGTATCCATCATCAACATCTTCT
TCATCACAAGGTTCAATGAGCCACACCAGCACCACCGCTGGCGGCGCCGGCGGTGGTCTC
ACTCGCTACGGTTCAGCTCCGGGATCATTTTTAACTACAGCAGTTGAAGCCGTCGTTAACGG
TAATCACGAGTTCGCTTCTCACGGATCTCATCACTCACACCTTGGTCCTTCACGGTTTTTTCA
GTCCAATCTAGCATCTACTTCGTTAAATTCCGAATCTACAAGTAAAGCGAAGGAACAATCGA
ATTTACAGAGGTCAATTGGTTTCAATGACTTAACAATCGGCGGCGGAAGCGGCGCTGGAGG
AGGAGTTTTGCCGACGACTTCAACGACGCCGTTGGTTCGACATAGTAGCTCACCGGCGAGG
TTCCTTAATCAACTTGCTACTGCTGCAGGTGATACTGTTTCAATGGGGAGAGGAAGCTATAA
CCCAAAAGGCGGTGGAGATAGTGGCCGGGGAATAACAAGGCTGAACTCTCAGCTCAGCTT
CACAACGCAAGAAGCTCTTTCTCAAATAGCAGAGGAAAATGAGGATATTGAAGGGACCAGT
ATAGCCAATGGCCACAGAAAGTCAACACATTCTTATGCCAGTGCAAGTAGTTTCGCGATGG
GTTCTTGGGAAGATAACAACTCTATAATGTTCTCTGTCACACCTAGCAAACGATCCAAGCAG
ATTAGCAATGACATTGTCAATGGGCTCGATGACGGGGAAACTCAGTTTCAGTTTGGCTTGTC
TCAGACAGCTCTAGAAATAGCATCTATGGATAGATTGCTGCACATCCCCGAGGATTCTGTTC
CTTGCAAAATTCGTGCCAAGCGTGGTTGTGCTACTCATCCTCGCAGCATTGCAGAAAGGGA
GAGAAGAACCAGAATTAGTGGGAAACTAAAGAAGTTACAAGATCTTGTTCCAAACATGGA
CAAGCAAACGAGCTATGCTGACATGCTGGATCTAGCAGTGCAGCATATTCGAACCCTTCAA
GATCAGGTTCAGAATCTGAATACAGAACTTGAAAACTGCAAATGTGGATGTAGGAAATCAA
GTCAATAA

>SlbHLH161
ATGGTTCTACCGGAAAACACCAACTGGTTGTATGACTATCATTATGAAGATATAGTCACCCCT
GATGTTAATTTCTCAGTTTCTGGTTATTCTTGGTCTATGCAGGGGTTTAATGGTTCAACTAAT
GCTAGTGTTGATATTGATGGCTCACTTGGTGAATCAGACTGCGTGAAGGAAAGCGGTTGTA
AGAAAAGGACAAGAGCTGAATCATGCACCTCATCAAGTTCCAAAGCTTGCAGGGAGAAAT
TAAGGAGAGAGAAACTGAATGAAAAGTTCATGGAATTGGCGGTGCTTCTTGAGCCCAGCA
GGCCTCCAAAAACAGACAAGAGTGCCATTCTTGTTGATGCTGTTCGCGTGGTGACACAGTT
ACGTGGTGAGGCTCAAAAATTGAAAGACTCAAATTTGGATCTACAAGAAAAGATAAAGGA
GTTGAAGGTTGAGAAAAATGAGCTTCGAGATGAAAAGCAGAGGCTGAAGTCCGAGAAAG
AGAAGCTAGAGCAACAACTAAAGACTATGAGTGCACAACCTAGCTTCCTGCCTCCTGCCAT
GCCTGGTGCTTTTGCTTCTCCAGTTCAAGCTGCAGGAACCAAGCTGGTGCCAATCATAAGTT
ACCCTGGAGTTGCCATGTGGCAATTCATGCCTCCTGCTGCAATAGATACCTCGCAGGATCAC
GTGCTCCGCCCTCCAGTTGCTTAA

>SlbHLH162
ATGGCGGAAGAATTTCAACTAGGGGGAGGGAATTGGTGGGAAAATAATACGTCCACATCAT
CAAGGAATAGGTTCGATAGTAGTGGTTCATCCATTACACCTACAACTTCCACATCGGCCACT

ACTACTGACTATAGTAGTAATTGGCCGATACAAACTAATCATGTGGACATCAAACCTAGGAC
TTTCTTGGATTCTGTGTCGGTGTCTCAATCTTCCGATCATGATGATAAGGGTGAAGGAGGAG
GAGGAGGTGGCGGCGGCGTTTTATCTTCTCATGATTCAAATTTTCAAATTATGGGTTTGGGC
CTTACTTCTCAACCACATGATTGGAACCATCAATCTTTATTGAATTATGAGGATTCTTCGTCA
CAAATGAATATTAGGGGTTTTTCATTAGATCAAAATAATTCAATATTTAGTCATTCATCAAATT
ACGAAGTAACTGATAATCATCAAGTGTTGCATTCGTCTACGACGAATTCTTGGTCTAAATTTC
CTCAATTTCTCCGAGCATCACCTTCGCCGGAGCAGCCTCCATTGCCGCCACCGGCGCGGCC
GTTGCCGCCACCTCACAGCCAGCTGCATTTCTCAAATAATACTCCATTTTGGAATGCAAGTG
CAGCTTCCATGAATGATGTTCCTTCAAGTTTATTTCCTTCTAATTTGCATAATATTCCTAATAA
CACAACAATTGATGAGAAAGCAAAGAATATGGGAGATGTTAGAGATATAAATAGAATTATAT
CAAAGAAAACCACCACTATTGAAACATCAAATAAAAGACCAAGGAATGAAACTTCAACTC
CTTCTCCGATAATTAAGGTGAGAAAAGAGAAAATGGGAGACAGAATCACTGCACTTCAACA
ATTAGTCTCACCTTTTGGAAAGACAGATACAGCTTCTGTATTATCAGAAGCAATTGAGTATAT
AAAGTTCCTCCATGATCAAATTGGTGCATTAAGCGCCCCATATATGAAAAGTGGAGCATCCA
TGCAACATCTTCAGAGTGATAATAAATCCGAAGATATCGGAGAAGGAAGAAATAAAGATTT
GAGAAGTCGAGGGTTATGTTTGGTACCAATTTCAAGTACTTTTCCAGTAACTCATGAGACTA
ATGTTGATTTATGGACACCTACTTTTGGATCAACTTTTAGATAA

>SlbHLH163
ATGGAAAGTTATAACCTAAAGTCTAAATTATGTCCAAAAATAAAAAGGAAAAATGTGGAGA
AAAATAGAAGGAATTATATGAAGAATCTCTATAATCAACTTCATTCCATGCTCCCTACTACTA
AGGAGGCAATGTCAGTGTCAAATCAAATAGATGCAGCAGTTAATTACATAGAAAACTTGAA
AATAAATTTGGAGATGAACAAGAAACACTTGGAAGAGTTGAAAATGGGCCTAAAGAAGGC
CCAATCATTCAATCCAACTAATGAGCCCGGCCCAACTATAAAGTCAACCCCTCATATTGAATT
CCATGAAATGGGCCCAAACATGGTCGTTGTTTTAATAACTAGACTTAATAATATAATAACATC
ATTAGATTATGCCATGAGGAAGGTGTTGAAGTTATGTTTACAAGCTTTCAACTCAATGGAAA
GTACTCTACATTGCATATTTCTCATGAAACTAAGGTGGATATGA

>SlbHLH164
ATGGAAGGTTGCCAGAGGTCAAGTTCAGCTCCGGCAGGGGTAAAATTGGAAAGAAAAGAT
GTGGAGAAAAATAGAAGGAATTATATGAAGAATCTATGCAATCAACTTTATTCATTGATCCCT
TCTACTCATCACTCTCGGGAAACAATGGGATTGCAAGATAAAATAGATGCAGCAATAAAGTA
TATAAAAAGTTCAGAAATGAAATTGGAAAAAAAAAAGATACACTTGGAAAAATTAAGTAGG
CTGAGTGGTAGAAAGAGGCCCAAATCATCCAATTCAACTAATGGGCCTAGCCCAAGTACAG
GGTTATCATCATCACCTCAGATCCAATTCCATGAAATGGGCCCAAATATGGTTGTTGTTTTAA
TAAGTGGCCTTGATAATATAGCCACTTTTAATAACATCATTCGATTGTGCCATGATGAAGGTG
TTGAAGTTGTGTATGCAAACTTTACACTCAATGGAAACTCTATGCTGCAAATTTCTCATGAA
ACTAAGATCAACATGAGTTCAACAATGGAATGTAGAGCTGCAAATTTATGTGATAAGCTGAA
GGAATTACTTCATGGAAAATCATATGACAATGAATTGGAATCCCAATTATATTTATGGGATTAT
ATAGTCGAATCTGAATTATTAAAATTTTATGACGTGGAATTTCTACCATCAACAAGTCAAAAT
TCAAATATGTACAATTAA

>SlbHLH165
ATGGAAAGGCGTATGAAGTCGAAATTACCTCCGGCAGGTCCAAAAATTGAAAGGAAATATG

TGGAGAAAAATAGAAGGAATTATATGAAGAAACTCTATAATCAACTTCATTCCTTGATCCCT
ACGTCTAAGGAAACAATAATGACAGTGCCAGATCAAATAGATGCAGCAGTGAATTACATAG
AAAACTTGAAAATGAATTTGGAGATGAACAATAAGTACTTGGAAGAATTGAAAATGGGCCT
AAAGAAGGCCCAATCATTCAACCCAACTAATGAGCCCGGCCCAATCACAAAGTCACAACCT
CAGATTGAATTCCATGAAATGGGCCCAAACATGGTTGTTGTTTTAATAACTAGCCTTAATAAT
ATAGCCACTTTTAATAACATCATTCGATTATGCTATGTGGAAGGTGTTGAAGTTGTGTCTACA
AGATTTGAACTCAATGGAAACTCAACACTACAAATTTCTCATGAAACAACTAAGATCAATAG
AAGCTCAGCGATGGAATTAATAGGTACAAATTTGTGTGATAAGATGAAGGAGTTAATTTATG
GACCCTCTTATATGGAATCTCAATTACATTTATGGGACTACAAAATTGAATTTGATACATTGG
AGTACTACTTATTACCAACAAGAAGTAAAAATCCAAACATGTATAGTTACATGCAAAATTAA

>SlbHLH166
AAAGATGTGGAAAAAAATAGAAGGAATTATATGAAGAATTTATGTAATCAACTTTATTCATTG
ATCCCTTTTCCTAATGCCTCTACCTCGAGCTCTAAGGAGACAATGGCAGTGCCAGATCAAAT
AGATGCAGCAATTAACTACATAGAAAGTTTGAAAATGAATTTGGAGAAGAACAAAAAATCA
ACCCCTCAAATTGAATTCCATGAAATGGGACCAAATATTGTAGTGGTTTTAATAATTGGCTTA
GATAATATAGCCACTTTCAATAATATTATTAGATTATGCTATATGGAAGGTGTTGAAGTTGTGT
CTACAAGATTTGAACTCAATGGGAACTCTACAATACAAATTTCTCATGAAACTAAGGTGGAG
ATGATCAATATAAATTCAAATTCAAGAATGGAATTTAGAGGTACAACTTTGTGTAATAAGATG
GAGGAGTTGATTTATGGAGCATCTTTCAACAATGATATCGAATATTCCCAAATACATTTATGG
GACTATATAATTGAATGTGATGCATTAGAGGTATTAAAAACTCCAAATCAAAATCCAAACATA
CAAAATGTTCAAGAAACTTCTAGGCCTTTGTGGGACTAA

>SlbHLH167
ATGGAAAGACACTTGAAGTCTAAATTAGCTCCAAAATTGGAAAGGAAATATGTGGAGAAAA
ATAGAAGGAATCATATGAAGAATTTATGTAATCAGCTTCATTCCATGCTCCCTACTCATTCATC
TACCTCTAAGGAAACAACAATGACAGTGCCTGATCAAATAGATGCAGCAGTGAAACACATT
GAAACCTTGAAAATGAATTTAGAGAAGAACAAGAAGCACTTGGAAGAATTGAAAATGGGC
CCAAATAAGGCCCAATCACTCAATCAAACTAATGAGCCCGGCCCAATCACAAAGTCACCAC
CTCAGATTGAATTCCATGAAATGGGCCCAAACATGGTCGTGGTTTTAATAAATAGCCTTGATA
ATATAGCCACTTTTAATAACATCATTAGATTGTGCCATAAAGAAGGTGTTGAGGTTGTGTCTA
CAAGCTTTAAACTCAATGGAAACTCTACACTACAAATTTCTCATGAACCTAAGGTACAAATC
AATAAAAGTTCACCAATGGAATTTAAGGCTACAAGTCTTTGTGATAAGATGAAGGAGTTGAT
TTATGGACCATCTTGCAACAATGACATTGAATCAAACCAACATCTATGGGACTACATTATTGA
ATCTGGACTTATAGAATTTAATACAATAGAGTTACCACCAATAGAAAATCATATGAAAAATAT
TTATGAAACTCCAAGCTTTTTCTAA

>SlbHLH168
ATGGATGAACCAATTTTTTCCCCTTCATCATCACAATCAAGTCTTCAACATAGGCTTCAATAT
ATTGTCAAGAATCAAACAAATTATTGTTCTGATTGGGCATATATTATTTTCTGGCAATCGTCG
AATAATCGTTCTTGTTTAACATGGGGTGATGGCCATCTAAACATGAAGATAACGAATAATAA
AGACGTCGAATGGTTCTACTTGATGTCTTTAGCTCAGTCTTTTTGTGTTGGTGAAGGAGTTG
TTGGAAAATGCTTTAGTAGTGGCTCTTTAGTGTGGCTAGCAGGGGATCAACAATTTGAGTTT
TGTCATTGTGAAAGGGCTAAAGAAGCTCATTATGTTCATGGTATCAATACTTTTGTATGTATT

CCAATTTCTAGTGGTGTGCTCGAACTGGGCTCGAGCACGATGATTAAACAGGATTTGAACTT
GGTTCAACAAGTCAAGTCTATGTTCTTTGGTTATGAAACAATTGATCAATTTGATGATTTTGG
TCTTTTCAATTGTTTGGAACTATATGGTGAAGAAGCAAAGAAAGGTGAAGTGGTAGTAGGT
ACAACACCACATGAAAACAAAGCTGGACTAAAGAACAAGACTTCGAAGAAAAGGAGGAG
AGAGATTTGCGAGACACAAGGAAACCACGTAGAGGCGGAGAGGCAGAGAAGAGAAAAAC
TTAACTCTCGATTCTACGCGTTGCGCGAGGTGGTTCCAAACGTAACAAAAATGGACAAAGC
CACATTGCTATCAGATGCTGTGACATATATTACCCAACTGAAAGCTAAAGTGGATGAATTAG
AATCAAAACTTCATAGTAACAATTATCACTATTATTATCCAGAGATGAAGATCAAACACAAAA
TGGAAAATCATGATATAAATGTTGTGGACAACCAAAGTTCCATAACAACTTCCAGGGATCAT
ACAATGGAAATTGAAGTGAAGATGGTAGGTCAAGATGCCATGATTAGGGTTCAATCAGAGA
ATGTGAATTATCCATCAACAAGATTGATGTGTGCTCTTCAAGAAGTTGAACTACATGTCTAC
CATGCCAACATCTCAAGTGTCAATGATTTTATGCTACATGACATAGTTGTTAAAGTTCCTCAA
GGATTGGAGACTGAAGATGAAGTAAAATATGCTCTACTTAGAAGCTTAGACCAACAAACAT
GTTCATAA

>SlbHLH169
ATGGATGAATTAATGGTTTCTTCTTCATCATCATCATCATCATTTTTTATACCATCTTTACTTTC
TCAAACTTCATCAAACCTTCAACAAAAACTTCAAAATATTCTCAAGATTCAAACAGATTCTT
GGTCATATGCTATTTTCTGGCAAACTACAAATGATGATGATGATGGTCATCTTTTTTTAGCTTG
GGGTGATGGTCATTTCCATGGTACTAAATCTAAAACAGGGGTTCAATCTAGTGAACAATCTA
CAGAGAGAAAAAAATGTTATTAAAGGGATTCAAGCTTTAATTTGTGAAAATGGTGATGAAAA
AGTTGATGATGATGATGATGATGATGAAGTTACTGATGCTGAATGGTTTTATGTCATGTCATTAGC
TCAATCTTTTTCCATTGGTGATGGTGTACCTGGCAAAGCTTTTAGTACTGCTTCTATCATATG
GTTAACTGGTTCACAAAACCTTCAATTTCATACCTGTAAAAGAGCTAAAGAAGCTCATCTTC
ATGGAATTCAAACTTTTGTTTGTATTCCAACTTCCAATGGAGTTATTGAAATGGGTTCCAATC
AACTAATCAAAGAAAACTGGGTGTTGATTCAACAAGTTAAGTCCATTTTCAACAACTCAATT
CCTCATATTGTCAATTGTTTAGAACAAAACACAAACATCAACCCGAAAACAGAGGAATTAG
TTTCAGTTTCAGTTTCCGCTGAATGTAATGATTCAGATTCCGATTGTCAACTACTGGTGGAG
AAGAAAACCCCGAAGAAGAGAGGTAGAAAACCAGGGGCAACACGTGAAACACCGTTGAA
CCACGTTGAAGCAGAGAGACAGAGGAGGGAGAAGTTAAACCATAGATTCTACGCTCTGCG
TTCTGTTGTGCCTCATGTTACCAAAATGGATAAAGCTTCGTTGTTATCAGATGCTGTATCGTA
CATCAACGAATTGAAATCGAAAGTTGCTGAATTGGAAACTCAGCTTACTAGAAAATCGAAG
AAACTGAAAATCGAGTGTACGGATAGTTTCAGTATAGACAACAACAGTACTGCTACAACAA
TAACAAATTCAGTGGATCAAATTAGGCATAATTCATTTGGTGTCCACAGCAATTTAAAAGTT
GAAGTTGAAGTTAAGATTTTGGGACCTGATGCCATGGTAAGGGTACAATCTGAAAATGTTAA
TTATCCATCAACGCGATTGATGCGCGCTCTTCAAGATCTTGAACTTCATGTTCACCATGCCAG
TATTTCGAGTGTCAACGATATTATGCTACAGGACATCGTTGTTAAAGTTCCTATAGGATTGAG
TACTGAAGATCGATTAAAAAACGCTCTAATTAGAAGCATACAACATCAGTAG

>SlbHLH170
ATGGATTATGAAGTAGCAGAGCTAAAATGGGAAAAGGGAGAGGTAGTGATGCATGGGTTAG
GTCCTCCAGGCGTGCCTTGTTATTATAAGCCTTTATCGACTCCTTCTCCAACAAAATACACGT
GGGACGATAAGCCACATGCTGCTGCAGGTGGCACACTTGAATCCATAGTGAACCAAGCTAC
GACTCATAATATTGACATCGGTGACGAGGGTGGTGATGATGATGATTTAGTGAGTTGGTTTG

ATGATTGTCTTCCTGAAACGTCCATGGATATTGTGGCCGTAGTTCCAACAAGTTGTACTAACT
ATAATCAACAAGTGCCCCCGTCCACACGTGTTGCATCATGCAGTGGTGATGCAGAGATGGC
ACGTGTGGGAATGGGATCTAGCTTTGAGGAAATATCGGAAGACTTTGAGAATCAAGAGGCT
AAGAACTTGATCGGCTCAATGGTATACGAGGGCAAAAATAACACTGTGAGCCCGGGAGAG
ACAAGTTTGGGTGAGGAAAGAGTACTTACAACAACATCTACCTTTAAGCATAATAAAAGGA
AGACACTGAATAATCATGATAGCAGAGGTCAGGAGTCGAGAGATAATGAGGATGAGGATGA
GAAAAAAAGATCCAAAATTTCTTCATTTTCAACAAAAAGGTGCAGAGTTGCTGCTACTCAC
AACCAGTCTGAACGAAAAAGAAGAGACAAGATAAACCAAAGGTTGAAGACATTGCAGAA
GTTAGTTCCAACATCGAGTAAGACTGATACGGCATCAATGTTAGATGAGGTGATAGAATATT
TGAAGCAACTACGAGCTCAAGTTAAAGCCATGAGCATGATGATTCATGTTAACATGCAGCC
ACCCCCTATGATGTTACCAAATATGGCATTCCAACAACAACAACAACAATTTCAAATGTCAA
TGATGGGGATGGCTAGACCCATCGATGTCAATGCCCTTAGCAGCCCCAACATAACAACAATC
CCATCGATTCTCCATACCACCGCACCCTCTAATTTCAATAACCCTCCTATTGCCTCCCCTGGA
GCTGATCCTTTAGCTTCCTTGGTCGCAGTACGCCAATTATCACAGCCTATGACGATGGATGCT
TATAGCAGGATGGCAGCATTGTACCAACAATATCTACAGTCAAATGCAAATCTTGGCTTTAA
AAATTGA

>SlbHLH171
ATGGAGCATGTGGTATCAATGTCTGAAGGAGATTGGAGTTCGTTTAGTGGAATGTGTTTTAC
TGAGGAGGCGGATTTCATGGCACAGTTACTTGGTAACTGTTCGTTTCCAAATGAGTTACCAA
GTAATTCTGGTTATTGGAATATTGGTCATGAATCAAATATTGGATCTTCAGGAGGAAGAGAA
CATAGTAGTTTCTTCTTTCCTCCCCTGAGTCATGAGAGTCACTACTCAAGTAATTCTCGGCCT
ATCTTGATGAGAAATGATAGTTCAATAACAACAGAACGTGGTCTGATGGATACTAATAATCC
AATTGAAGCAGATGAGTACTTCGCTAATAACATGGAGTTTGATGCGAACATGGCTGAACCTC
TTCTTGATGGAAAAGGCTTGCAGCTAGGAAGAATAGACTATGAAGATCATAGCCCAACAGA
GAGTTCTAAGAAAAGAGTGCGGTTACATTGTCATGTACCAAAGAACAAGAGAAGTACAAA
ACTTCAAACAGAAGGTAAGACTGTCGAGATGGATATGAAAAGCAAGGCTGTGCTTCAGAG
GCAAAACTCTATGGTCAGTTGCTGTTCAGAAGATGAATCTAATGTTTCTCTCGAGTTGAGAC
GGAAATCCAGAGCAAGCAGGGGTTCAGCAACTGACCCCCAGAGCATGTATGCCAGGAAAA
GAAGAGAGCGAATTAATGAGAGATTGAAGACCTTACAGAGTCTCATACCTAATGGAACCAA
GGTTGATATTAGTACCATGCTTGAAGAGGCAGTTCAGTATGTTAAGTTTTTGCAACTCCAAA
TCAAGCTGTTGAGCTCTGATGATCTATGGATGTATGCTCCCATTGCCTACAATGGAATGGACC
TTGGGCTTGATCTGAGGATTGGCAATCCAAAATGA

>SlbHLH172
ATGGAGCCTGTATTTGAAGATGAATGGAGTGGAATGTGTTCCACTGATCAGGAGGCTGATTT
CATGGCTCAGTTGCTTTGGCAGCCTAATAACATTGACAATATGTATTTTTCTAGTTATAATGGT
TGTAATAGCTCTCAGATTTCCTTTCCGAGTCTTGAAAGCTACTATCAGAGTCATGTTCAGTCA
ATCTTGACGAGAAATGGAAGTTCAATAACAAAGGAAAATGGCATGGTGGAAGGTGAAAAC
ACAAGTTCACCCTCTCAGGTGCTTGCTACTTACAATCCAATTGAGGCGGATGACTTCCTCAA
CCAAGATGTGAGCATGGAGTCTGGTGAAAACACGGCTAAAGTATTGGATAGTCCACCAGAG
AGCTCAAAGAAAAAAAGATTGTGCAACCTGCTAGGGGATGTCCCAAAGAACAAGAGAAGC
GTAAATCTGAAGAAGGCTAGTGAGATGGATGGAAAAAACAAGGCTGCACTTCAGCGGCAG
AACTCGATAAGTTGCTGCTCAGAAGATGAATCTATTAATGTACCAAAATCCAGAGCTAGCAG

AGGCTCAGCAACTGACTCCCAGAGCTTGTATGCTAGGAAAAGAAGAGAGAAAATTAATCA
AAGATTAAGAATTTTGCAGAGTCTAGTCCCTAATGGAACAAAGGTTGATATTAGCACCATGC
TTGAAGAGGCAGTCCAGTATGTCAAATTTTTGCAACTCCAAATCAAGCTTTTGAGCTCTGAT
GACCTATGGATGTATGCTCCCATTGCATACAACGGAATGGACATTGGCCTAGATCTAAAGATA
GGCACTCCAAAATCGTAA

>SlbHLH173
ATGCAACAACAACAAGAAGACTTTCAAGAAAACTTGGAATCTTGCAATTATTTCTCCAAAA
ATAAATCATCATCAAGTGATAATAATATGATGGGAGAATATTATGGTTTTGATATTGAAGCTAT
TAAAAACTTTTGTAGCTCTTCCCCATTTTACCCTCAACATAATCATGTGGAATTTCAAGAAAC
TATAGAGAGTAATAATAATAGTCCAGAAAGTAGAGCTAAAAATCATAAAGAAGCTGAAAGA
AGAAGAAGAGAAAGGATTAATTCTCATCTTCATAGCCTCAGAACCCTACTATCTTGCAATTC
TAAGTTGGATAAAGCTTCATTACTAGCCAAAGTTGTTCAACGTGTAAGGGAACTCAAAGAA
CAAACTTCACAAATTATGCAAAGTGAAACAAACTTATTATTCCCTTCAGAAACTGATGAAAT
CACTGTGTTATCATCAAATGATTGTTTAGCTGATGGAAGATTACTCATCAAGGCCTCTCTTTG
TTGTGAGGATCGATTCGATCTCATCCCTGACCTAATTGAAACCCTAAAATCACTTGGATTGA
GTCCTATAAGAGCTGAAATGGTCACATTAGGAGGGAGAATCCATAATGTGATTGTATTAGCT
GTTGATCATAAAAAGGAGAGTAATAATAACAATACTGATCATGATGAATCGTCGTTATTGTTC
TTGAGAGATGCTTTGAGGTCTATAGTTCAACGTTCGAGTTATGGTACAGGTGAACGAGGTAA
AAGACGAAGAGTATTAGGTCAAGGAACAAGGAATTACTAG

>SlbHLH174
ATGGATTCATCAGAGTTTGAGAAAAACCAGAACAATTCTGGGTTATTAAGGTTTCGTTCAGC
CCCAAGTTCATTTCTTGAAAATTTCATTGATGGAGTTGGGAATAATGGAAATATGGGTAGTG
AGAATAAAGGGTTGTCATCAAAATTCAATCTTGATGGTTTAAATAACCAATTGGTTTCTCAG
AATTCTCTTGATAGTAAAGTTTTTGCAAATTTGAGTAGTAATTCACAGTTGCCACCTCAATAT
CCAAGGCAAAATTCTACTGTTCATATGGTGGGGTCAATGGAAGGGGGTGGTGGGTACAGGG
TGATGGGGTCAGTATTGGGAAATAGTAATAATCATCATCAGGGGCAAAATAAATTGGCATCA
AATCTTATGAGGCAAAACAGTTCTCCAGCTGGTTTATTCTCTCAACTCAATTCTCTAAATGGC
TATGCTACGTTGAAAGGCGGTGCTGGAGGTTATAGAATGGCAACTGGTGCCAATGGAGATC
CAAATCCATCTTCAAGCAGGTTGAAAGATCATTTTGGCTTCTCATCAGTGACACCTTCTTCA
TTAGGAATGTTGTCTCGAATCTCAGAAGTCGATGATGAGAGCAGCATAGCAACTGATCTCGA
TGATGATGAGAAGCGTGGAAATGGCAACTCAGAACCTCAACTCTACAATATGGGATTTCCTT
TTAATTCTTGGAGTGATCATTCACAGTTTCAGCAGACACTCACAGGCCACAAAAGGGAGTT
GGATAGTGAGGGGAATAAGCTATTTGCTAATGCTCAGATTGAAGAGCTTGGAAACCGGCCC
CCAATTTTGTCACACCACCTAAGTTTACCAAAGACACCAGCTGATATAGCAGCTATGGAGAA
GTTATTGCATTTTCAAGACACTATTCCCTGCAAGATCCGCGCCAAACGTGGCTGTGCTACTC
ATCCTAGAAGCATTGCAGAAAGGGTGAGAAGAACACGTATCAGTGAACGAATGAGAAAGC
TACAAGAGCTAGTTCCCAACATGGACAAGCAAACAAACACCGCGGACATGCTAGACTTGG
CTGTCGAGTACATTAAAGGCCTCCAAAAACAATACAAGGTACTCACTGATTGTCGCGCTAA
CTGCAAATGCTCAGCAATGCAGAAACCAGATTGA

>SlbHLH175
ATGGATAATCTGATAGAGTGTATTGAAGCAGGAGAAGCTAGATCCGAGAATTGCGGAGCTA

GAATGGGAGAAATTAAAGATGGGTTTCATCAATTTGGTGAGGAAATTTTGTCTGTTACGTCG
GAAGGTGGAAGTTCTTTCACTGCTTTACTTGGGCTTCCGCCGAATCAAGCGGTGGAGCTTT
TGGTTCAGTCACCGGAAACTGACAAAATAGCATCAGATAAGCTTGCTATCAGTGAACCTCA
CTATCGTTATCCTCCTCCTCCTCCGATTTTTCCTTCAGATATCGCTTTGATCGATAGAGCTTCT
AAGTTCTCCGTCTTCGCCGCCGCCGGTAACTCCCCGGAGTCCAATTCAACCCTTTCGAATTC
CGGGTCGAAATCACTGTTCGTCAAGCAAGAACCCCTAGATTCCGAATGTAACCACAACTCA
TCTCCGGCGACTTCCAATCCACTAGTCCATCAGAAATCAACAAAGAGAAAGGAACGCGAG
AAAAAGGTAAAAGAAACTTCTAAAAAGGGGAAAAAATCAGCTAACGATACCTCAGAAGAC
GGCGGCGAGAAGCTACCATATGTTCACGTCCGAGCGCGTCGAGGCCAAGCCACTGATAGCC
ATAGTTTAGCAGAAAGAGCAAGGAGAGAGAAGATTAATGCTAGGATGAAGCTACTACAAG
AACTGGTCCCAGGATGTAACAAGATTTCAGGTACTGCAATGGTGTTGGATGAGATCATCAAC
CATGTACAGTCCCTACAGCGCCAAGTGGAGTTCTTATCAATGAGACTTGCTGCGGTTAACCC
GAGAGTTGATTTCAACCTCGAAAGTCTTTTTGCAGCAGAACGGAGTGGTTCCCATGTGGAG
AGTAACTTACAAGACATGGTTGTGCCGCCTATCTGGGCTGAGGGACAAAGCAGCGGAAAC
AGAAACCAATATCAACATCTATGGCTTATTGAAGGGTTCCATCAGCCTGCCTGGGGAAGGTT
AGAAGACAATTCTAGCTTTGTTACACCAGAGAACTCGCTGTTGACATATGATTCCTCAGCAA
ATTCAGCATCTTTACATCCAAATCAGCTGAAAATGGAGCTATGA

>SlbHLH176
ATGCTAGCCATTTCTTCTCCTATGATTTCTACTAATTATATTAATTTTGGTTGGCTTTTGGAAGA
TTCAAATAGCCAACAAATTAACATTAATCCCATGGAACCTCTTCATTCATCATCACGAAAAA
ACCTTCAACACTCTGATTCAAATAAATTTGATGAGATTACTATCAACGGTGGTGATCATCATC
AACCCGATCAAACGGTGAAGAAGCTTAATCATAACGCAAGTGAACGTGATCGTAGAAAGA
AAATCAACGACTTATATTCTTCTCTTCGTTCTTTACTTCCTCCTTCTGATCATATGAAAAAGCC
AAGTATTCCATCAACAATATCAAAAATTCTAAAGTACATACCAGAGTTACAAAGTGAAGTGG
AAAGATTGGTTCAAAAGAAAGAAGAATTTACATCAACATATGTTTTCAATAGACAAAAATTA
GGTGATTTTACCAAACAAAAAAGAACAAAAGGAGGAATTGAGAATTCTTCATTTGTTATTTC
AACAAGTAAATTAAGTGATAAAGAAATAGTTGTTCAAATATCAACTTTGAAGATCAATAAAG
GTTCAATTGGTGAGGCTATTTCACAATTAGAAGATGAAGGACTTGTTCTACTAAATGCAAAT
TCTTTTGAAACTTTTGAAGATAGAGTATTCTATACCTTGCATTTTCAGGTTGAAGGAAGTATG
GTGGTTGAGATTGACATGTTAAGAGACAAACTCTCATCTTATTTTGAGAAGGAAGACAAGT
TATAA

>SlbHLH177
ATGTTAGCCATTTCTTCTTCTTCTCCTCCTTTATTTTCTACTACTACTAATAATTTTGGTTGGCT
TTTGGAAGATCTTATAAGCCATGAATTAACAAATAGTGGAGAAACTTCAAATTCATCTCAAA
AAAGCCTTCAACATTGTGATTCAAATAAATTTGATCAAATTATTATCAACAGTGGTGATCAGT
ATCAACCTGATCAGACGGTTAAGAAGCTTAATCATAACGCAAGTGAACGTGACCGTAGAAA
GAAAATCAACAGCTTATATTCTTCTCTTCGTTCTTTACTACCTCCTTCTGATCATACGAAAAA
GCTAAGCATTCCATCAACAGTATCAAGAATTCTAAAGTACATACCAGAATTACAAAGTGAAG
TTGAAAGATTGGTTCAAAAGAAAGAAGAATTTACATCAAAAAATATTTTGAATAAACCAAA
AAGAATAAAAGGAGGAATTGAGAATTCTTCATTTGTTATTTCAACAAGTGAATTAGGTGACA
AAGAAATAGTGATTCAAATATCAACTTTGAAGATCAATAAAGGTTCAATTAGTGAGGCTATT
TCACAATTAGAAGATGAAGGACTTGTTCTACTAAATGCAACTTCTTTTGAGACTTTTGAAGA

TAGAGTATTCTACACATTGCATTTTCAGTTTGAGAAGTTACCAGAGATGATGAAAGATTTCA
CTGTGGGCTTAGCTTTGATTGAGCAGTTACCAAATATCGCGCCGTTTTTGTTGGAGTCAGCT
GCCTATAGGATCATGCTTTTAATTGTGAACAAGGATGATGATCATTACGATCTTGATATGGATT
GTTTTTTCAATAATGGGATTGTAGACAATCTAGTTGAATGTTGTAGATCAAGAAATTGGCGTA
GTCTTCGTAGGCAAGCTGCTGAGACATTACGTGAATTGGTTAATTGTGGAACGATGGCACA
GAATCAGGAATTGCTTAGACGTGGTGTCGTTACAATTTTTCTGCAAATGCTTAATGATGAGG
AAGCTATAGACCTTGATGATGAGGAGTGGTTTGATGACTTATTAGTTGTTGAGGCTGTGAAG
ACTTTGGCTTTATTGTCAACTAAAGTAGATGTCAGGGGTGACTTGGTTACTGCATTTGTTGA
GCAAAATCTGTGTAGAAGGCTGATGTTACTTTTCAGGAATACTAGACACACCATAGTTGAAA
ATGTGTTGACATTTGTGGAGAACTTTCTTACTATAGGAACTGAAGAGCAAATGCAGGTTCTT
CTTGACAACCAAGTTTTGCATCATCTCAGACTTGTAATTGTCGAGCCTGTGGTGTCCGATTTA
TACCTCCTAGGTGGACAATATAAGGTAGTAGAGATTTTGGGCAATTTTGCTCGTCGTTCAAG
AAGGTTGAGGGATCTTGTTATTGACAGCGGTGAAGATCTCATCTCATTATTTCGAGTCCTTC
GTTCCCTAGCAGATGATTCCATCGTTGCATTTGACGCTTCACCAGCAATCAGTGCTGCCAGG
ACAATAGGATACTTGTGTTTTGGTAGTCCACCTCCACCATTTGATAAGTTGAGACCATCACTA
CCAATCCTCCGGTTTCTCATAAATCAACTGGAACCTCATATAGGAGAAAGAGCTTGTCCTGT
TATTAAGCATGCCTGCTTGATTATTGCATGTCTGGCACGCGGTGGATTTGATCCTATAAATGC
TTTGATTGATGAAAATATGTGCCCGATCCTGGTGATGCTTTTGGCACATCCTCACAGTGAGG
TTGTTGCAAGTGTGCTGAAGGTTGTAGAGAACTTCCTCAAGAATGGAACTGATAATCAAAT
TCAGGTTCTCCATGACAGCCAAGTTCTTCAACATGTTTTAGAAATTGTAATGAATCATGACA
ACCTACCTCCTTTACATCTGAGGAGTGTTTGTCGTGCCATCGCCAATATAGTGAATTACAGG
AGCAGTCAAATCCAGAGAATGGTTGATGCAGGTATCTTCCCTTCGATTATTCAGATTTCGATT
AACCAAGAGGTTGGTGATACCAAATATGAGGCTATATACGCCATCTCAAGCGTTGTCACGAG
GGGATCTCACGAGCAGATTAGGGTCTCCGCGGTATTCTGA

>SlbHLH178
ATGGAGAAGAAGCTAAATCATAATGCAAGTGAACGTAATAGAAGGAAGAAGATGAATTTTC
TTTATTCAACTCTTCGTTCTTTGCTTCCTCCTCCTACTAATAAACATCAAAAGAAAAAATTAA
GCTTTCCAGCAACAGTATCATATGTACAAGAATACATCCCAGAGTTGAAGAAAGAAATAGA
GAGGCTAAGCAAAACAAAAGATTTGCTTTTATCAAAGAAATCAAATTATTCATTACTCAAAA
TTGATGATAATAATAAGAGAAAATTAATTATTGGTGGAACTTCTTGTAATTCTTCAACAACAT
CAATTTGTGCAAGTCAACTAAGTAATTCACAAGTTTTGGTACAAATTTCAACAACTCAAGA
AAATAATTTTCCAATTTCACAAGTATTTGCAAGTGTAGAGGAAGATGGATTAATTTTGCTAAA
TGCATCATCCTTTAAATCTTTTGGAGACAAGATTTTTCACAGCTTGCATTTTCAGATGCAAGG
ACCAATTGAAATGGACATTCAGGTTTTGAAGACTAAGCTTTTAGTAATGTGTGAAAAAAGA
AGAAAAAATTCATATATAGTTTAA

>SlbHLH179
ATGGATATCAACCACATTAATAAACTCACTACCTCCACTTGGGATCCAACCATGTCCAATATG
GACAACCAACAAGTCTTTCGCGATCAACAACAACAACAACAACCATGCTTGTCCAGTATAC
CAAACGACCATATCTACCACGAACATCATCATCATCAACAACAATTTCATTTCGAACATAAC
CCTATTTGGCCTAGTTTTCCTCTTCAAAACCCTCAACACCATCACCTTCCCTCCTCCTCAACC
CAACAACAACAACAACAACAAGAAGAAGTAGTAGTTGTCCCTTTTGATCATGTGTTAAACA
ACCATGTTCAAACCCTAATAGAGGATCAAGAACACGATGATCAAGACGAAGATGAAGAGG

AAGAAGAAGAGCTAGGAGCCATGAAAGAAATGATGTTCAAAATTGCATCTATGCAACCAGT
AGACATTGATCCATCCACTATACGAAAACCTAAAAGGCGTAACGTACGTATAAGCAATGATC
CACAAAGCGTAGCAGCCAGGCTACGCCGCGAAAGGATAAGTGAAAAAATCAGAATCTTGC
AAAGATTAGTCCCTGGTGGAACAAAAATGGACACTGCATCCATGTTAGACGAAGCAATTCG
CTATGTTAAGTTCTTAAAAAGACAAATCCGTCAATTACAATCATCGAATCATAACCTCCCACC
AGCACAGATACCAGTATCGTCATGCCCTAATAATGAAAATTGGGCAAATAATATTGTCACAC
CAAGTACAAAAGGTCTGATTCTTGGCTCCTCTAGTAGTACTACAACCAATAATGTTACAACT
TTTGTTGGTAACACTACATTGGATCCACCTTATGAGGTAATTGGTAATTAG

>SlbHLH180
ATGGCTCTTGAAACTGTTGTTTTCCAACAAGATCCTTTCAATTATAGCCACAAAGATTGTAAT
TTTTACAATCTTGAAACTTTTCATGATTATGGTAATTTTGGCTATGAGGGGTATAATTGGAATT
CTTCAATACCACAAAGTTACAACGACGACGATAACAACAACAATATCAATAATAATAATAGT
AATTCTTCGCCGGATAAATATTTTCCGGTGGAGAGTACGGTGGTTTCAGGACGGAGAAAAA
GGCGGCGGACAAAATGCGCGAAAAACGAGGAAGAAATACATAATCAAAGGATGACTCACA
TTGCCGTCGAGAGAAATCGTCGACGGCAAATGAACGATTACCTCGCCGTACTCCGGTCGTT
AATGCCGCCGTCTTACGCTCAACGGGGTGACCAAGCATCAATTGTTGGAGGCGCAATTAATT
TTGTTAAAGAACTTGAACAACTCCTCCAATTTTTGGAAGCACATAAACAAGTTATTACGACT
AACCAGCAACATATTCAGTATAGTTCGTTCTCTAAATTTTTCACGTTCCCTCAATATTCTACG
GGTAATAACAATCACCCGTTGGCGGCCACCACCAGCAACGAGGGGTCGGAAGAGAGACGG
TCGGCGGTCGCGGATATCGAGGTGACCATGGTGGAAAGTCATGCAAATGTTAAGGTGTTATC
AAGAAGAAGACCAAAACAATTGTTGAAAATTGTTAATTGGTTACAAGCAATGTGCCTGACT
ATACTTCACCTTAGTGTTACAACAGCTGATCATATGGTACTTTACACATTTAGTGTCAAGGTG
GAAGAAAATTGTGAGCTAAATACAGTGAGTGAGATAGCAAGTGCTGTACATGAAATGGTGG
CCATGATTAAGGAGGAGGCTATGCCTTGTTGA

>SlbHLH181
ATGGATCATAGTCGTGTTGAGCAGCAACCAATTACTTCTGGTTTAACTAGGTATCGATCTGCG
CCAAGTTCTTATTTCTCTACTCTGTTAACTACTACTACACCTGTTACTGGTGGGGGTTGTGGT
TATGCAAGAGATGATTTTTCCAATTTACCAAATTCTACTGCTTCTTCTTCATCTACTGATATAG
AGCAAGTTTTTGCTAGATTTGTAGCTAGTATTGGTAACTCCAACTCTAACTCTGGGAATTTTC
CTACAAATCAACCAATTCATGAAAACCCCACGAGTAATATGAATGTTAGATCGGATTTTGTT
GTACCAAAACAAGAGATTACACCGCGGTTAGTACGACATAGAAGTAGTGATCACTCTCTTG
TTTCAAATTATCAAAATCAGAATCAACACTTCATTTCACCTGTGAAAAGGGAACCAGAAAC
TCTGCTACAGAGTACTGATCATGTTTCAGCTTCACAACTGAATTACCAAAGCCAACCTCAAC
AAATTGAGAAGTTAAATGCAGTTACAGATAAGCCATTTGTATTCGCGAATTCGGCTAGTTTG
ACACCTGGTAGTAGTAGTGGTGGTGGTGGTAATTCGAATCTTACCCGTTATAATAGTTCGCCT
GCTGGATTCTTTGCTCAAATCAATATTGAAAATGAATATGGTGCTATACGAGGCATAGGGCGT
TACGAAACTGGTACTAATGCTGCTGCAGATACTTCATTGTCGACTCCAAAAAGTTTCAAGAC
TCAAGTTGGATTCTCATCCGGGAAACCTCCTGCTTCTCCCAGGCTTATGGCTCCAATATCTG
AATTTGGAACTAAAGTCTTAGAGGAAAAGAGCATGGGGAATGAACATAAAAACGACGAGC
ATTGCATTACAGATTTCCCCATGCCTTCTTGGGAAGATTCACACATTTTGTCCGATGATTTCC
TTAAAACTGAAGACATTGAGATTGAGCCATTCTCTAATGAAGATGCATCCCATAATCAGAGT
AGTGAAGGGCTGGCTCGCCCGCCAATTCCTTTATCTCATCATTTGAGTTTGCCCACAAGTAC

CATGGAGAAGCTCTTGCAAGATTCAGTTCACTTGAATGTCAGGGCAAAGAGGGGTTGTGCC
ACTCACCCTCGTAGCATTGCAGAAAGGGTAAGAAGAACACGAATAAGCGACAGAATGAGG
AAGCTGCAAGAGCTTGTTCCCAACATGGACAAGCAAACAAACACAGCAGATATGTTGGATT
TTGCAGTAGATTACATTAAAGAGCTGGAGAAACAAGTCAAGATACTAGCAGAAAGGCGCGC
CAAATGTACGTGCATAAATAAGTAA

>SlbHLH182
ATGGGGTATTTGCTGAAAGAGGTTTTAAAGACTCTGTGTGGAGTCAACCAATGGTCTTATGC
TGTTTACTGGAAGATCGGTTGCCAGAACACAAAGCTTTTAATTTGGGAAGAATCCTATTATG
AACCATCAACATACTCCGGTATTCATGGAGTTCCGATGGTTGAGAATCCGGAACTGCCGTTC
CATGATTGGGGCGTTTGCTGGGGTCCGGGGGAGGTACGTAATCCTCAGCTTATGAATCAAGC
AGGGGAGAGAGTGCAATTGCTTGTAAACAAAATGATGATGGAAAGTCAAATCAATATAGTA
GGAGAAGGACTAGTCGGACGGGCCGCAGTTACTGGGAGTCATCAGTGGTTTCATTCAGAG
GGTTTAAGTAGAGTAGTTCATCCACAAGAGGTCCTGAAAGAGCTCACGGGACAATTTTCTG
CTGGCATCCAGACAATTGCAGTTGTTCCTGTTCTTCCTCATGGTGTACTCCAATTTGGATCAT
GCTTGCACATAATGGAGAACATGAGTTTTGTGGAGGATGTGAGGATACTGATAAGTCAACTA
GGATGTGTCCCTGGTGTCTTGTTATCCGATGAAACTGCAATGAAAGATCCGATGGTGAATAC
TGGCACACCAGTTTATATGGGAAGTTCAGTTCTTGTAGATTCTTCTGTGAGCACAAACGTAA
TGAACTCTGCTCCGGTTATTGCTAGCTGCAGTTATCAGGGCAACTCAAGTCAGAATGATGGT
TTCATTCGTCAAACTTCTTCCTCTTTGGATGCACAAGTTCAGGATAGAATAATGCAGTCTATC
GATTCATCATTTCAAGCCTCCAATATGACCCAGCGTTTTGTTGAATCTCATGATGATCGTCAG
TTTCACAAAAAAATTATTCCAGAGGTGAAATCACACCTGTCTCCTAATAGCCAGCTAATAAA
TAGCGTGATTAAAGCGGAGGTAATTTCTCCAAGCTCCAACTTGTGGATGAGTCAACAAGCT
CCTTTACACATTCCGAGGCCTCCCTTTCACCAGCAATCTTTTACTGACTCATTAACTGTAGAT
AGCAGCAGCTTAAGCAATGTATCCCAGCTTAATGGTTTCACTGCATCTGATCCAAGACCTAA
TGATGTCCTGATCTCTAGTTATCATGGAAATTCAATTTCTCCATCCAACGGAGAAAATGAACT
ATGTAAACGAAGGGATGGGCATCACCGATCAATTCCATGCCCCAATTCAATTGCTGATGCCA
ATGGACTGTCAAACATAATCAGTTCTTGCACAAAGTCGACTGGAAATGGGCTTCAAACTAC
GTCAAAATTTAATGTCGGGGATGATTCGAGTACTAGTCATATTTCTGATGGTCAGAATGCACA
ATTTATGTGGGATGAGAGTAATGGAATAGTCGAAAATGATTTGTTTCAAGCACTTGGAATTAT
GCTAACACAGAATGAGCACCCATGCAGTACAAGCAAGTCGGTGCAAGAGGTTTGTGTTGA
AAAACATGAATATGTGGGGCAGAGTGCATTACTTGAAAATAACAAGTATGAAGATTCCTGTG
TCCAACGTCACTCGGGGGATGACCTGTTTGATGTTCTGGGTGCTGATTTTAAAAATAAGCTT
TTCAATGGAAGCAGGAACAGTTATCAAAGTAATGGACCAGACTCAAACACATGGGATCGGG
TTAAGAGTAACTCTACTTCTGTGCTTAGTCAGCAGGCTTCTTCGATAGTCAATCAAGGAAAG
TCAGATAGTGGTTCATTCTTCGCTGCTGGTTTTGAGCGTCTGTTGGATTCTATATCGTCCAAG
CCTTCTGCCAAGCAGAATATGGATGATGATGTTTCTTGTAGGACAACATTGACAAATTTGAG
TAACTCCTCTGCTCCAAATGTGTCATCGTCCTATGGCCGAGCTGGTTTCTCGAGTCAAATTC
AAGGAAATGTATTTGGGAAACCCAAAACCCTTGCAAAACCAGGATCAACAGTTTCTAGGTC
ATTTAGATCTGAGAAAGAAAAGACAGGAGCTTATTCGCAGAGTAGTTCAATTTATGGGTCTC
AAATTAGTTCATGGGTGGAACAAGGTCATGATACAAAGCCAACTAGCAGTGTTTCTACGGG
ATATTCTAAGAAGCCTGATGAGACGAGCAAGACCGGTCGCAAAAGGCTTAAACCAGGAGA
GAATCCTAGACCAAGGCCAAAAGATCGGCAAATGATCCAAGATCGTGTGAAAGAACTGCG
AGAAATTGTTCCAAATGGGGCAAAGTGTAGCATTGATGCACTACTAGAACGCACGATCAAG

CACATGCTTTTCCTGCAGAGCGTCACAAAACATGCGGACAAATTAAAACAGACTGGAGAGT
CAAAGATTATCAGCAAGGAAGGAGGGTTGCTTTTGAAAGATAATTTTGAAGGAGGAGCGAC
ATGGGCATATGAAGTTGGTTCACAGTCTATGGTCTGCCCGATTATAGTTGAGGATCTGAATCA
ACCTCGTCAAATGCTCGTGGAGATGCTTTGTGAAGAACGCGGTCTATTTCTGGAAATAGCTG
ACATAATAAGAGGATTGGGCTTGACCATCTTAAAAGGGGTTATGGAAACGAGGAACGACAA
AATATGGGCGAAATTCGCTGTAGAGGCAAATAGAGACGTGACGAGGATGGAGATATTCATCT
CACTTGTTCGCCTCTTGGAGCAAACATCAAAAGGCGCAGAGGAATCTGCTAAGGCCATTGA
TAACAACACAGCAATGGTGCATTCGTATCATCAAGCAGCGTCCATACCTGCAACAGGTAGTA
GACCCTGTAATTAA

>SlbHLH183
ATGGAGTTCTTGAAAGTGGCTAGTTATGTAAACTTGATGGCTATTGATGCAAACACAACAAT
GGTTCATTCATGTCCACTTACTCGAATAACCTTAAGACTGAGAACTCAGGGTGATGAATGGG
ATGAGTCTACTTTCCTGGAAGCAACAAAAAAGGTTATTGGGGACGTGACATAA

>SlbHLH184
ATGCAAGCTATGAACTCATTTCAAAGCACCGGCGAAAATGGTGCTTCGTCCGGCGAACACA
TGAGTCATTCCCATTTCGATCCGTCGTCGTCTCACGACGACTTCCTCCAACAGATTCTCTCTT
CCGTTCCTTCTTCTTCTCCCTGGCCAGAAATCTCCGGCGACGGTCACCCCTACAATTTCGAC
GACCATCAGTCAACTCTTCTGGCTTCCAAACTCCGGCAGCATCAGATCAACGGCGGTACCT
CTGCTGCTGCAGCTGCTAAAGCGTTGATGCTTCAGCAGCAGCTTTTGCTTTCCAGAGGAAT
CGCTGGTAATGGCGGCTCTGGGATAAACGGCGATCAAAACGACGACGGTTTGAATTCGGGG
AATGATATTTCAGTTCAAGCTCTTTACAATGGATTCGCTGGATCTCTTGGTCAAACCTCTAAT
CAATCTCAGCATTTTCACCATTCTCAGGCCCAGAGTTTCGGAGCTCCGGCGGCGTCGTTATC
GATGAATCAAACGCCGGCGGCGAGTGGTTCAGCTGGTGGAGCGCAGCCAAAGCAACAGAA
AGTTAGGGCTCGTAGAGGACAAGCAACTGATCCTCACAGCATTGCTGAAAGATTACGGAGA
GAGAGAATTGCAGAGAGAATGAAGTCTTTGCAGGAGCTGGTACCTAATGCCAATAAGACAG
ACAAAGCTTCAATGTTAGATGAGATCATCGATTATGTCAGGTTCCTCCAGCTCCAAGTCAAA
GTTCTGAGTATGAGCAGATTGGGTGGTGCTGCAGCTGTTGCTCCCCTAGTTGCTGATAGATC
CTCTGAGGGAGGAGGTGATTGTGTACAAGGAAATGTAGGTCGAGGTGGCAGTAATGGAAC
GACGTCGTCTGCAAACAACGACAGCAGCATGACGATGACGGAGCACCAGGTAGCTAAGCT
AATGGAAGAAGATATGGGATCAGCCATGCAGTATCTTCAAGGAAAAGGCTTATGCCTTATGC
CAATTTCCTTAGCCACAGCTATTTCCACCTCCACGTGTCACTCCATGAAACCCAACAACCCA
CTACTACTCGCCGGAGGCTCCGCCATTAACGGCGTTGGTGAGACCGGCGGTGGACCGTCTT
CTCCTACCTTGTCGGCTTCCACTGTTCAGTCAGCTACGATGGGCAATGGCGGGACTTGA

>SlbHLH185
ATGATGATCAAGAAAATGTATAGTGGTGTGCAAGGTTGTAATTCAGCATCAAGATTGGAAAA
AAAATATGTGGAGAAGAAAAGAAGGAGCAATATGAAAAACCTTTTTAATCATCTTTGTTCTC
TCCTCCCACCTCATCCATCTCAGTTGCAGGAAGCAATGGGATTGCCAGATCAAATAGATGCA
TCAATAACTTACATAAAAAACTTGGAAATCAAATTAGAGAAAAGCAAAATGCAATTGGAAA
AATTAAGGACTAAAAAGAGGTCCAATTTATTATGCATGGCTAATCATGATACAAATCCAAGTA
TTAGCAAATTATCTTCACCTCAAATTGAAGTCCAAGAAATGAGCCCAACAATGGACTTAATC
TTGATTAGTGGCCTTGATAACTTAGTTATGTTTTATAACATAATTAGGTTATTCCATGAGGAAA

ATTTTGAAGTAATTAATTCCAACTTTTCTCTAGATGGGAACTCTATGATGCAAATTTTCCATG
AAAGT

>SlbHLH186
ATGATTCGAGGTGACGAGAATAATCATGATTTTCCATGGGAAACTAATGATTTTTGGTCATAT
CTGAACTTGAATGACAATCATGTTGGGAGTGGAGAGACGTTTGATGGTGACAAGTTGCCAG
ATCTGACTAGGTCTGATACTTGTCAACCGCTAATAGTTGTTAAAGAGGTGGTTCAAACAACA
ATTGGTGTTGGTAAAAAAAGAAGTCCACCAAACCGAAAAAGGAATGGTAAGGAAATTGCT
GAATCAAATTCTGATGCTGATAGAGCCGAAACTAAAAGAAAATCAGAACATGAGATACACA
TATGGATGGAGAGAGAAAGGAGAAAGAAGATGCAAACCCTGTTCGAGACTCTTCATTCGTT
GGTTCCTAATCTCCCTGCTAAGGCAAAAGGTGGAAGTGACACAACTCAATTCTCAGAAGAA
TTTACAGTGGAAGAAAGGTTTATGCAAGCTGCAATTCAAATAATGCCCTTGACAACCCCCA
AATGA

>SlbHLH187
ATGGCTCATCATTATTTCAATCCAGATATGCAAATCATTATTCCACCATCCTCTGTATTTAACA
CTGACACAAATTTGAATTTTCATAACCAATTTCCAGAACAAAATTACTTGAATACCATTTCAC
TGGAAATGTCTAATTTCAATTTTCATAAGCACTCTGATGAACATATTTTGAACCAAACCTCTG
ACTTCCCTGTATATTTAACAGAAAATTTCCAACAAGATATGTTCAACCTTCCAGTACCAACTA
GAAATGATATAAATGAAAGCAAGAAAAGGAAAACAATTGAAACCCCAGAAAGTAGCTCTG
CTTACTCATCCCCTGCAGTTTCTTCGAGAAGAAAAACCGGCAAAGGAAGAGGAAAGAGAG
TAAAAAGGGATGAAAATGAAGAAGAGAAACTAAGGCAAGTAGTGCATGTAAGAGCCAAGA
GAGGCCAAGCAACTGATAGTCACAGTTTAGCAGAAAGGGTTAGAAGAGGAAAAATTAATG
AAAGACTTAGGTGTTTACAAGATATTGTTCCAGGATGCTACAAGTCTATGGGCATGGCAGTA
ATGCTGGATGAAATAATTAACTATGTGCAATCTTTACAAAATCAAGTTGAGTTCCTCTCAATG
AAGCTATCTGCAGCTAGCACATATTATGACTTCAATTCAGAGACAGACATTTTGGAGACAAT
GCAGAGAGCAAAGGCATATGAAGCAAACATGATGCAGAAGCTAAAGAAAGAAGGATGTGA
AGGGATGGGTTCTAATCAAGTTGGCCCTTTAATTGACCGCACTTTTGGCTGCTATCCCAAGT
TGTCTTACAACACCTAA

>SlbHLH188
ATGACTACTACAAGAAGTTGCATCATCAGAAGTAGATTTGCTTATCGGTTTCTTCACTCCCTT
AGAAAAATGAACCAGCAGGATAAAACCAATTCACGAAGGGTAAAACATGCTGCTTATGCAT
CCATGGCATCTGTTGTCGGGTCAAAGAGAGCTTGGAGTCGGGCCGTGCTTTCAAAGATCCG
TAACAGATCTCTCTTGCTGAAGAAGAAGAAGAAGAGAAGAAGAAGATCATCAGACGAGTT
TGGTGAACTTCGAAAAATAGTGCCTGGTGGTCAACTTATGGATTTCTACAATTTATTAGATGA
AACTGCGGATTATATCAACTCTCTTACTTCACAGGTACATGTTATGAAAAATATTCTTAATCTA
CTTTCAACTTGA

>SlbHLH189
ATGTTGCACGAAGAAAAGGAAAATAAAGATGAAAGAATGATACATATAGCTATGAAGCGAA
ATCAACGAAAACAAATGAATAATTATCTTACTCTCCTTCAATCTTTGATCTCATCTTCACATG
TTCAAAGGGCAGACCAAGCATCAATAATTGGAGGAGCCATAAACTTTGTAAAAGAGCTAGA
GCATAATCTCCAAACTCTACGATCGAATTGTTCAAGTGAATTAAGTAGAGGGTCATGTCAAC

CTCAAATACTCACAAAGAGAAGACCAAAACAACTCATGAACATAGTAGTTGGCCTTCAATG
CTTGTGGCTTACAATTCTTCACCTTAATGTCACTACTATTCATAACCAAATTGTTCTTTGCTCC
ATAAGTGCTAAGCTAGAGGAAGGGTGCCAATTGACAACAATAGAAGAAATCTCTCATGCTG
TTAACCAATTATTGGGTAGAATTCAAGAAGAGGAGGCTACTTCAAGTTCAAACTAA

>SlbHLH190
ATGCCTCCGTATGCAAATGAGGTTCCAGTAAAAGGGAGTTCTCCCTTGTCGTTTTCGGGGTT
GCTAGAACCAAAGGCAAGCCAACCAACTGAAGCTCACAATTGGTTCTACTGTTTACCACAG
TTGCAACAGGGTTTTGCTCCTGTTTTGAGCACTGTACCGAACGAGAAGTTTGCTCCTCAGT
CTGTTGACAATTACGGGGTAAATGAAGAAGCCAATGCTGGCCCCGGATTCGCGCAGAAAAG
ATTTCTTGTTTTTGATCAATCTGGTGATCAAACAACTTTACTTTACAGTTCTCCAAATGGTAC
TCCTGTGCAATGCCTACCCTCTTGGCATCCAAAATCCGCTGCACCTTGTCATCTGATTAAGG
AAGGCCAACAGATTTAGGGAACGGAATTTGTCCATCTGGGAAATACTCCGGTGGTGAATAT
TATGAAGAAAATCATAGAGATGATGTAGAAAGTGAATTGCATGAGGATACTGAAGAATTGAA
TGCACTACTCTACTCGGATGACGATGATAGTTATTCTGAGGATGGTGAAGAAATGAGCACAG
GGCACTCGCCTAGTACTATGACAGCTCATGATCTACCATCGTGGCATGACGAGATGGGTGAA
GAAGTTAATAGTTCAGAATGGCCAAGCAAAAGGCGTAAGCAGTTAGATGGTGGCTGTGATA
TACCACCATCACTCGTGGATACTGCAACGTCTGCAAAACCCTTTACTTGTTCCGACTTAGAA
GATGATGCAGAATCCAGTTGTGGGAACAGTCATAATAATCAAGTTTCAGAATTGGTTTCTCT
ATCTGGGAAAAAGAGGCCAAGAAAAGATCAAATTCTTGAGACGATAAGCATTTTGCAGAA
AATAATCCCCGGAGGGAAAGGAAAAGACTCGATGGATGTCATTGATGAAGCAATCTGTTAC
TTGAGATCCTTGAAG

>SlbHLH191
ATGGAGGATTACTATAGAGAGAATGAATTCTTATTAGATGAATTATTTTCTTTAAGAAGTGAT
TTATGGGAAAGTACTTGTCTACCTATGGAAATTAATAGTAGTAGTAGTAATTTATTTTGTAACA
ATATTAATTATAATTGTTTTGGGGAAATTCCTCTTCCTTCTACTACTACAACTACTACTACTACT
ACTTCTTTTGAAGACTACTACAATAATTTTCCAATAAACCAAAGCCAAAATTATTCATTATTA
CATAATGAATTTTATACTACACAAAAAGTAGATGTATTGTCTCCACTTGAACTCACTAATTCT
TTCAATTCCCAATTAGATGATTTTTCATCAATATACTTTGATGTTGGAAATTTTGGTGATTGTA
AATTGGAGAGAACTCAATCAACTGCATCGTCAGCTGCTATAGCTCCTGCTAATTTCAATATCG
GCGCGTGCCTTGATAAAAAGTCGAAAAAAAAGAAGGTGAATGGGGAACCTTCTAAGAATT
TAATGGCTGAAAGAAGAAGAAGAAAAAGGCTCAATGGTCGTCTTTCTATGCTTAGATCTATT
GTTCCTAAAATTAGCAAGATGGATAGAACATCAATCTTAGGGGACACTATCGACTATACAAA
GGAACTTCTTGAGAAAATCAACAATTTGCAACAAGAAATGGAACTAGGGTCAAATCAACTA
AGCTTGATGAGCATTTTCAAGAATGAAAAGCCCATCGAAATGTTTGTTAGAAATTCACCTAA
GTTTAATGTGGAAAGGAGGAATAATATTGATACAAGAGTGGAGATTAATTGTGCAAACAAAT
CTAGTCTTTTGCTATCAACATTGACCACACTAGATGCCTTAGGCCTTGAACCTCAACAATGT
GTTATAAGTTGTTTCAATGATTTTGCAATGCAAGCTTCTTGCTGTGAGGAAATGGAGCAAAG
AGGAGTTACAGATGCAGAAGAAATAAAGCAGGCACTATTCAAAAATGCTGGATATGTAGGA
AAATGTCTCTAA

>SlbHLH192
ATGGAGCAAATGGGAGCATCTTTTGATGAAGAATATTGTCAATCTTTGAGCAAAATGTTCTT

GAATGAAAATTCATCAGATTTTATGTTTCAATTACATGGTGAAGATAATATTATTGAAGGGAG
TTTTTTTTCTTCTTCTAATTCTCATACTAGTAACAACAATATAGATTATTTTTCTCAAGAAAAT
AGTATTGATAGTAGAGGAAGTGATGCTATGTTTTTTCTCAACAACAACACAAGTCATGAATA
TTTACAATATAATTATGATGTTGAGTCCACTAATTTTTATATGACGGGCAACAAGATGAATCTT
GAAAATTCATTGTCTAATGATGATTATGTTATGAAAGAAAATATTGGCAATAATTATAGCCAAT
TGGATAAAGAAATGTTGCTAAAGAGAAAGTTTGATAAAGTTGAAGTTCAATCAACAACACA
AGAGGAAACACACAAGGAGTTTGAAAATCCCAAGAAGAAATCAAAGGTGTCAAGAGATCA
TGGACAAAAAAACAAGAAGAATAGCCAACCAAAAGCCAAAAAGAACATCCAAATGAACA
ATGAAGAAGTTGGAGACAAAGAGACTAATAATAATGGACAAAGCACAAGTAGTTGTAGCTC
TGAGGATGATTCAAATTTAAAAACCAAAACAAGGGCAAGTAGAGGGGCTGCAACAGATCC
ACAAAGCCTTTATGCAAGGGTTGACTTAAGCACAATGCTTGAAGAAGCAGTCCATTATGTG
AAGTTTTTGCAGACACAAATTAAGTTACTGAGCTCAGATGATATGTGGATGTATGCACCAAT
TGCTTATAATGGAATGGGGATTGACCTCTAA

>SlbHLH193
ATGGTGGCTATGAGGGAGATGATTTTTAGAATAGCTGCGATGCAACCAATTCAAATCGATCC
ATCATCTGTAAAAGCGCCCAAGAGAAGGAACGTGAAGATATCAAGCGATCCTCAAAGCGTG
GCGGCGCGCCATAGGAGAGAAAGGATAAGTGAAAAGATAAGGATACTACAAAGATTAGTAC
CAGGTGGAACAAAAAATGGACACTGCATCAATGTTAGATGAAGCAATTCATTACATGAAATTC
TTGAAGAAGCAAGTTCAATCTCTTGAAAAAGTTGAAATTAATAGGCCTATGTCAATGTCATT
AAGTGGGAATTACTTACCTAATTATAATTACCCTTATCATCAAAGTGTTCAACCCTAG

>SlbHLH194
ATGGAGAAGAAAAATTTGTTCTTGAACAATGTCAACACAATGAATGAGTTGAATTGTACTA
GTACTAGTTTTTACAATCCCAATTGGGAGAATTCTTCAATGGATTATCAAAATGACAATATTT
TGTCTAGTAATAGTAACAATTTTGGTGAAATTTCACCAAATTCTTTTGTTGGTAGTAATAACA
ATTCTTGTTATACTACTCCATTGAATTCTCCTCCAAGACTCAATCTTTCCAACTTTGATCATCA
AATCAAGGGAAATTTTCCAAATACTAGTAATAATTTGCCACATTTTTCTACTAATCTTGGAAA
TTTTTCTTGTTTTGGTGGCAATGATTCACAATTTGTCCAAAATTTGGAAAGTTGTAAACTTTC
AAGAAACAAGTCTATGAAAGAAAGTGAATTTGGAGATTCAAGAGAGAATTCTTCAATTTCT
CAACAAATTCAACTTGAAGAAGTTGGAATCAAATGTCAAAATGATGCAAATTCCAAGAAAG
GGAAATCAATTCCAAAGAGGAAAGCTAAAGAAATCACTCCCAAGAATGATAATGTTTCAAC
ACAAAACAATGAATCAAGTTCAAAAAGAGTCAAATCTGATGAAAAAAATGAAGAAAATCA
AAAGCCTCAAGATTCATTAAAGGATTATATTCATGTTAGAGCTAGAAGGGGTCAAGCCACAG
ATGCACATAGTCTAGCTGAAAGGGTGAGAAGAGAAAAAATTGGTGAAAGAATGAAGTTTTT
ACAAGATCTTGTACCTGGCTGCAATAAAGTGACTGGAAAAGCTGTGATGCTTGATGAGATTA
TTAACTATGTACAATCCCTACAATGTCAAGTTGAGTTCCTCTCGATGAAGTTATCTAATCTGA
ATTCAACAACTGACTTCAACGCGGAATCTCTTACGTCTAAGAATATGTTTCAATCCGTCGGA
TCGTTGCATCATAACATGAATTCATCAGAATCCTCAGTACAAGAATTTCCATATGGATTTCAA
TCTCAACAAGGGTCAAATATACAAAGTTTCTTAACTAAAGAAACAGAATTTCCCTTTAAGAT
TAATCCTCATTTGGATGGTTTTGTGGAGCAAACACCTCAGGTTCCAACATTCTTTGAAGATC
ATGATCTTCATAGTTTTATCCATATGGGGTTTAGCCAAATTCAAGCACAAAACTATCCTGGCA
ATGTCTCCACAGCACAAATGAAGGCTGAACTATGA

>SlbHLH195
ATGGATCATAGAAGTCATCAGCAGCAACAGATGACTTCTGGTTTAACTCGATATCGATCCGC
GCCAAGTTCTTATTTTACCAACCTATTGAGTAGTAATAATCCAGCTGGTGTTTCTGGTGGGAG
TGGGAATTGTGGTTACGCAAGAGATGATTTTGATCAGTTGCTCAATCCTCACGCTTCGAATA
GTGGTATAAAGCAAGTTTTTGATAGATTTGTAGCTAATATTGGTCCCCAAGATTCGAATCCGG
ATGGCCTAATTGATGATACTCAGCAAAATCAAAATCCCATGAGCAATATGGATGTTAGATCTG
AAGTTCTAGCTCCTATGAAACAAGAACACGAAGCTCAAATGAATTACCAATGCCAAGAACA
ACAGAGTCAGAATTCACAGTTTGTTGCACCTGTGAAACAAGAAATTGCTCAGCAGAATAGT
GATTATTCTTTGGCTTCACAGATGAATTATCAAAATCACAATGCAACAGACTTTACTTCAGG
AATGGATAACTTTGGTAGATATAATCGTTTGAACCACACGAAAATGGATGGTGGATTTGGTA
CTGGTAGTAGTGACTCAAATCTTACTCGTTATAACAGTTCACCTGCTGGATTCTTTGCACTAG
TCAATATTGAAAATGAATATGGTGCATTGAGAGGCATAGGGAGTTATGGAGCTGGTAGTGGT
ACTAATGCTGCTGGAGAAGTATCATTTTCCAATCCAAGCAGGTTTAGTAGTCAAACAGCTCT
CCCATCCGGCCAACCTACTTCTTCGGGGCTATTAGCTCCAATCTCTGAATTTGGAGCTAAAA
GCATAGAAGAGAGCAGACGAGGCCATGAAAGTTTTGGTAAAGGCCATAAAAGTGATGAGA
GTTACATGGCAGGTTTCGCCTTGCCTTCTTGGGATGATTCACAAATTTTGACTGATGATTTCC
TACAAGTACCAGAAGATGATGAGTCCGGATCATTCTCCAATGTAAATGCATCTGATAATCAG
AGTAGTGAAGGTCGAGCTCGTCCTCCCACTCTATTATCTCATTTGAGTTTACCTCAAACATCA
GCGGAGTTATCCGCCATGGAGAAGCTCTTGCAAGATTCAGTACCATGTAAAGTCAGAGCAA
AGAGAGGCTGTGCCACTCATCCTCGTAGCATCGCCGAAAGGGTAAGAAGAACACGAATAA
GCGAAAGAATGAGGAAGCTGCAAGAGCTTGTCCCCAATATGGACAAGCAAACAAACACAG
CAGATATGTTGGACTTTGCAGCTGACTACATCAAAGAACTAGAGACACAAGTCAAGGCACT
ATCGGAAACGCGTTCAAAGTGTACATGCTTGCATGAATAG

195 amino acid sequences

>SlbHLH001
MDMGSKNDTQGQKRNKDATNFQSPNISLDWQLSGSNLTNASMGMIPNSNPLVDSVFPTIWDR
PPNSSHLGFYGNNNANAQISPCIMNQHETAAIGSVPTRGSMSWNPLNSMLKGAMFVPPIPGMIP
QSLAQLPADLGFIERAARFSCFSGGNFNDMMNRPLSVPESTKPCYRGPAPTWRTEEVLASSGLN
SPSAVDPWKQNIRSGVDGSKDVSLPHENKTHEQSPLKIEKKNEIFARSRDEGKESVGLSGNESD
EAECSGRQEEMGSAGLESSPKSLGSRKRKKYSQGTEHDRMKRVQQLPAEPDKELIETQKGDGR
LHSPSSKHGGKNSKQRSQSSDPPKEDYIHVRARRGQATNSHSLAERVRREKISERMKFLQDLVP
GCDKVTGKAVMLDEIINYVQSLQRQVEFLSMKLSTVNPRLDFNLDGVLTKDSQAGPSSALAFS
PDNMTMTYASLHGWQSGLLQSGLPGDGNYIDAFRRSNTQLSSMSGGYRDPSSQVPSVWDDQL
HNVVDMGFTSTAPLDCQDLSSLPPDQMKTEP

>SlbHLH002
MNGGGESNHVFPWEIDDVWSYLNMNDNQIGSGAMFEGDMMPDPLRFDTYHPLTVVNEVVG
SSTNVAKRRSPPNSKKNGKKIVEPKSSADGVGYREGLEHEIHIWTERERTKKIGILFETLRALIPN

ISAKADECTIIDEAVNHILKLKNTFEKLKQKKLDDLQEYNIRLMSSQKFPDVGNSWENYLGDQ
GTTSNSSFIKPKIHGATPLMVNNNIPTSFMTWSSPDMILNVCGKDAHISVCCPKKLGLFTFICYV
LGKHNIEIVSAQVSSDQSRTMFMIQAHAKCGNDIAQFSEAPTVEEIYKQVAIRIMSFETPK

>SlbHLH003
MNGGGENNHVFPWETNDGWPYQNLNGNQIGSGVTFEGDKLQDPNRFDTYEPLTAVNEVIEAR
GLNHDLHIWTARQRTKKIGILFDTLRALIPKIPAKAGKSTIVEKAVNHIQKLQNTFEKLEHEKLK
RLQEHNVRCMSSQKFTNAGNNWEKYQGDQGSTHNSSSIASTTHGTNPLMVNNNIPTGFVTWS
SPNVIVNVCGENAHISVCCPKKSGLFTFIGYVLGKHQIEILSAQVSCDQFRSMFMIQAHATGGT
GIAQVSEASRVEEMYKQVAIEIMSFATPN

>SlbHLH004
MNEDGENNHDHLCDDIWSYLDWNDHQVESGETEGNKLLDPTGSDTCEPFTVINEVVEVSVNV
AKKRSSANRKKNGKEIAEPNSGVYGAEVRKTSKHEVRKWTERERRKKMRTQFENLHALVPNL
PAKADMSKIVYEAVNRIRKLKNTFKKLESQKLKSLEEYNIRLTGSQKVDNSWEKYVGDQGSTC
NSIAIIPTNHGASPLIPTSFMTWSSPNVILNVSGEDAHISVCCPKKPRLFTTICYVLEKHKIDIVSS
QISSDQFRSMFMIQAHAKDGSGVAQFSEAFTVEDMYKQAANEIMLMTTPK

>SlbHLH005
MWILFKTLHALVPNLPSKADKSKIVYGTVNRIRKLENTFKKLESQKLKRLEEYNIRLTGSQKVG
NSWENYVGDQGSTCNSTAITPTNHGASPLIPTGFMTWSSPNVILNVYGEDAHINVLS

>SlbHLH006
MKKKKMNEGGGNDHDADVWSYLDLNDYQVGSGEKFEGDKVLDLTRSDACQPLTIVNEMFE
VNIYAAKNRSPPNREKNGKGIAEPKSGVDRGGGKRESKHKIHKWTERERRKKMLTLFDTLHDL
VPNLPTKADQSTIVGEAVNHILELQNIFTKLKSQKLERLEEYNIRLMSSQKVDNSWEKYVGDQ
VSTNNSTVITPTTHGPSPLIPTGFMTWSSPNVILNVCGEDAHISVCFPKNPKLFAIICYVLEKHKI
DILSAQVSSDQFRSMFMIQAHAKGGSELALFSEVFTVEDMFKQAAIEIRALTTSK

>SlbHLH007
MEPMVVEMSSTVISNPVTSSDRVISRRKKSKKSLRNQTQNSSNNNNNNSETPTNTTEWKTQAQ
QQVYSSKLLKALREVRISSPAAAATTTTSSVPAPKGGRAVREVADRVLAVTAKGRSRWSRAILT
NRLKLKFMKKHAKRQKMAVSSTSRLPRKPRLGILKLKTKNLPAFQKKARVLGRLVPGCRKQPL
PVILDEATDYIAALEMQIRAMSALADLLSGASSSTTAPLDQLSSSRPPPI

>SlbHLH008
MWQEMMMKEEAEEEEIERLFMGVMGGYKDAFMPPLTDFSTINEMGSSSSYGNKRHKKEAPL
ADDNMHKERNRRGKMAELYSLLQSLVPTISHIHKATREKIVAESIDYIKRLEEEVLRLKNLKKS
VVVYKPALSQWRNRVSSVNATVSKGLAFFGIQFQLTQGLMTNIFSVLDKHQAEVLAANISVSD
HQLTTLTITVTIGNNESNTVESIRRELLLF

>SlbHLH009
MGIISQQPLLSSPEFLNKCDLSDPLWTKVLEKGVYIRRRSCSWLCKKHNGARIMKRRVRVERSR
RSSNEAEKNVKMLEKLIPNCEPLSSSMGLERLFRETADYIWALEMKVKVMKMIVNVLSTPDIN

>SlbHLH010
MSHIAVERNRRRQMNENLKVLRSLTPCFYIKRGDQASIIAGVIEFIKELHLVLQSLEAKKRRKSL
SPSPGPTTPRPLQLSPTPESSPFITHNNNFKELGACCNSPVADVEARICGSNVMLRTISKRIPGQIV
KIINVLEKLSFEILHLNISTMQDTVLYSFVIKIGLECQLSVEELALEVQKSFTSSDVLCINEI

>SlbHLH011
MYASQSLSFHPTSDQEDEKSFVQNHIAASGFVELQQQQQQFHSSENNLNMQLEFGHDSNNTRF
HPNWEEISFNPYNNQQLSYPISNPSLGLLGGFHQRTELASTSTNLFYEPPQMNMPLNLCTPQSSL
FKELFHLSPHGSSYGLGSSGTGSLFSLGHDQEEVTGNLYHDGSFHELTGDMMINSAAIKKRILG
KDIKHHASEKQRRVHFSDKFQALRTLIPNPSKNNRATIIADAIGYIDELKMRVNELKVQVDIKKE
RIKRRRSMVEEYGAVIMEDNQDDQQVMMNKSTNWHHQIKSSKNSNTEVDVRIMEDEVIVKF
VQHKQMLKGVNCLLLVSKALDELQLDLQHVAGGLIGDHYSYLLNSKICEGCTVYASVIANKVI
DVLDKEHADIN

>SlbHLH012
MYVEESVCYDPATHHVQHEGLTEDVFVIQEHTYHNNNDSSQQDVAVAAAAAALEIEFQHQLN
LEMEQCYNNNNNNNTHNNNNNIVNEGLSCDQANWGEMNFPPYQNQQHNDNGNSNNNFHQ
QDFSNPISETPYLTTPDLLNMFPLPRCTQSSLLPQKSPNLLTSLGLIGDIDGGGASTSSAICDPSSL
LLPLNLPPQPPLLRELFHSFPHGYGLRNLRSNNNTSFFNGLEETDQGLYQENGETRPFQNGIFEF
SGGMNDIAKNRDGIKETKHFATERQRRVHLNDKYKALRSMVPNPSKNDRASIVKDAIDYINEL
KRGVNELKLMAEKKRCNKDRIKRQKTEGGTTISMDGSDAKQIMDEVEQSYNGNSLRSSWLQ
RRSKNTEVDVRIVDDEVTVKLVQQKRINCLFSASKVLDDLQLDLHHVAGGLIGDYYSFLFNSKI
SEGSTVYASAIAKKLIEVVDIQYAAIAPTNSY

>SlbHLH013
MFSSEPISREMDKLNSFFFSGGGGGSSSFKNGEGMESEFFRSKEMMGSDFFQQQSQFQQSNSG
GLTRYRSAPSSFFAGILDGDGNNSGENFITGDGSSSSDSDSMFTALLNNNDTTNNNGTRDMND
QNQKNQLQFGTSLKQEIGEEIEFGNENGVQNRYENGGVSYSVGVQMQTRANLSNGNGDSDLI
RQNSSPAGFFNGFMREVGNFGASVGTNREASTSTNGFNNHISYSTNQSSTSNFMPSIAENESWN
DASFNSLKRNRDGDLKMFSTNFNGMTNQNDESRNYTSSGLSHHLSLPKTSSEMAAIEKYLQFQ
QDSVPCKIRAKRGCATHPRSIAERMRRTRISERMKKLQDLFPNMDKQTNTADMLDLAVDYIKD
LQKQVQTLTDKKAKCSCTSKQLQYSNGTT

>SlbHLH014
MNQCVPSWDLDDSTVPRKNLIQTQSNSLAVDVPSLDYEVAELTWENGQLAMHGLGPPRANNK
PISSYGGTLESIVNQATRCNDDVPLHLHGKSTVDRNKQSGDEVVPWFNNHNAVAYAPPATGLVA
MTKDALVPCSRNTSNSDNQRSVHVPGIDGSTHVGSCSGATNSRDWTVAPRMRVRPTRREWSS
RADMISVSGSETCGGDSRQLTVDTFDREFGTTMYTSTSMGSPENTSSDKQCTNRTGDDHDSVC
HSRDQKEGGDDEDDNDNKKGSKNSSSSTKRKRAAAIHNQSERKRRDKINQRMKTLQKLVPNS
SKTDKASMLDEVIEYLKQLQAQVHMMSRMNMSPAMMLPLAMQQQLQMSMMGMGMGMG
MGMGVAGVFDINNLSRPNIPGLPSFLHPSAAFMQPITSWDNSNSAPSPPSAAMPDPLAALLACQ
SQPINMDAYSRMAALYLQFQQPPTGSGPKN

>SlbHLH015
MVTGNMLWSGEDKAMVASVLGKEAFEYLMSGSVSAECSLMAIGNDQNLQNKLSDLVERPNA
ANFSWNYAIFWQISRSKSGELVLGWGDGCCREPKEAEEREVKKILNLRLDDEGQQRMRKRVL
QKLHMLFGGTDEDNYAFGLDRVTDTEMFFLASMYFSFPRGEGGPGKCFGSGKYLWLSDALTS
NLDYCARSFLAKSAGMQTIALIPTDVGVVELGSVRSIPESLELLQNIKSCFSSFLSLVRDKQAAG
IAAVPEKNEGNNPRLSNSGAVTERTDGNPKIFGHDLNSGTHFREKLAVRKAEERPWDMYQNGN
RMPFVNARNGLNPASWAQFSNVKLGKPVELYAPPTPGHNLMNGGREEFRLNNFQHQKPAARM
QIDFTGATSRTIVSPAHNVESEHSDVEASCKEDRAGPVDEKRPRKRGRKPANGREEPLNHVEAE
RQRREKLNQRFYALRAVVPNISKMDKASLLGDAIAYITELQKKLRDMESERELRLGSTSRDAIT
SEDSPSSEIQIRGPDINIEAANDEVIVRVSCSLETHPLSRIIQIFKEAQINVVESKLSAGNGTVYHTF
VIKSSGSEQLTKEKLLAAFSSESNSLRQLSPVGQ

>SlbHLH016
MEQLAVSSSPMAVAPPPVDVNQVPLGLQQMLQYVVKSQPEWWAYAIFWQTSNDDEGKNFLA
WGDGYFQGDGVVINNKGGGGSSSSLKSQAQSERKKVIKGIQALMDGNGDTDLVDDGDVTDT
EWFYVMSLARSFSAGDGSVTGKAFGSDDFLWITGPDQFQLHYSCERAKEAQIHGIQTLVSIPTS
NGVFELGSTQLIKQNLSLVQQVKSLFLCCPPIQFLEKTISFADIGLVTGLQQDDNDYKLRENSRK
PHPVVAKKRGRKPKGGEEDAHMAALNHVEAERQRREKLNHRFYALRSVVPNVSRMDKASLL
SDAVSYINQLKAKVDELELQLIDHTKKPKIVTESSSADNQSATTSSDDQVIKAANPTAAPEVEV
KIVGTDAMIRVQSENVDYPSAKLMIALQNLQMQVHHASISSVNHLVLHDVVVRVPQGLSTEDE
LRTALLTSYDL

>SlbHLH017
MALEAITQQQQYLYALLGTTNWSIDSGSDYYNNNEFVPNHQNVTAPQEYEDYSNWNLPAPLL
VDSQNEFHQWGQNCSSVLDHNFTIVDQQEQLESPIEMSSSTTRPRRRRTRTKKNDEEIENQRIT
HIAVERNRRKQMNDYLSVLRTLMPESYVQRGDQASIVSGAINYVKELEQQLQFLSGKKHLTSQ
NQEANGETSPFSEFFSIPQYSTTITAAAAATTTSENVCCENEYYRNQQLPATADIEVTMVENHAN
LKIRSKKRPRLLPTIISGLESLRLTVLHLNVSKVDQFVLCSLSLKVEEDCKMNSVEDIAAVVNQI
LSRIHEEAN

>SlbHLH018
MASSQTVSNPDVNSSHSRESKRKKRRKIGDDGEIEQQTSLDQSRWRTDTEQQIYSSKLLQALR
HVRRSNDNPSPVNAGRAVRETADRVLAVTAKGRSRWSRAILTGRLSLRLSQINKKHKKAKLNS
GNIKSKKPAAKKRLPALQRKVRVLGRLVPGCQKLPFTNLLEETNDYISALQMQIKAMTFLTGLL
SAGGAGSVAAHPDRLG

>SlbHLH019
MAIGKPESGQQKISSTSNLSSFPENDLVELKWQNGQIVMQGQNSSAKKSTVPNNLPSSASGDR
DKYTGNSSTSKIGKFGLMDSMLNDMSLTVPTGELDLIQEDEGVPWLGYPADDSLQQDYCAQL
LPEISGVTANEQSGQSVFGLINKRGSSDKMIGDSHSVPVHNAVNFERRNTSKVSPSSRFSPLSSL
PSQKGHASIPTLESGVSDVFSSKNSNTPLSVLGESNQSKASAGDAKSNRIQKQNMPGNRSNLLN
FSHFSRPATLVKAAKLQSSTGGSNISGSPILEAKGKKGEEKVTIGDNHVSAAATENFLTSKKDNF
PHYPTNGVSSQLESRPSGASFHDRSCQAEQSDNAFRDCSSNNDNTHDHFTSAKATKDIADGER
NVEHGVACSSVCSGSSAERGSSDQPLNLKRKTRDNEEFECRSEDVEEESVGIKKPCAARGGTG

SKRSRAAEVHNLSERRRRDRINEKMRALQELIPNCNKADKASMLDEAIEYLKTLQLQVQIMSV
GAGLCVPPMMFPMQHMHGAQMPHFSPMSLGMGMGMGFGLGMLEMNGRSSGYPMYPMPSV
QGGHFPSPPIPASTAYPGIAVSNRHVFAHPGQGLPMSIPRASLGPLAGQPSTGAAVPMNVAREGV
PVEIRGAQPNLDSKTPVHKNSQIVQNAEASCPQNQTCSQVQATNEVLEKSAQKNDQLPDVIGS
AANRLTNRTNVPGNEAGPSL

>SlbHLH020
MVTTSELQYYQQDPAWTSPNLNYATTLLQPGHHLGVPSLYTPAICAANAVSPRRSCFIPCLPNS
MEACHNGVNWRPNLVPTKNVYSSDTKCFLPHLIGFKSPPTDASTNPQKRFLICDQSGNQTRFFF
SQGRPAEDEIITPKEVFGAYGLHQNENLNVVVEQRFQVKPVIGEKSDESYVNGEESNTLEDTDE
INALLFSSDDIEGDEDDDFYGEDDEVTSTDRSPCAKQGCCGCGEHEQQSVELTEEVASSDGTCK
RQRLLDGGYKKSSYIESRWPNDDVEAKCVRGSLPSSGKDKDSSLSTRERKVKIRETLRILESLIP
GIKSKDPLLVIDEAINYLKSLRGKAKALGSELPQEYPPASC

>SlbHLH021
MKPKEETTNLSSSSITFAADHCHNNMVCHQDSFANQNYMMFKAASCQGANKSVSTNGKLTQ
AQDHIIAERKRREKLSQRFIALSALIPGLKKMDKASVLGDAIKYLKQLQEKVKTLEEQTKKKSV
ESVVFVKKYELYGDGENSSSDENYSSGTVPVDEALPEIEARISEKDVLIRIHCEKSKGVVEKTVA
EIEKLHLSVINTCALSFGTSALDITIIAQMDEEFAMTVKDLVKNLRSALKVFM

>SlbHLH022
MEPVVAMSEGEWSSLSGTCSTEEANFMAQLFGACPNEQQLPSSGLPNFWTNHESNIGGSSEVSI
FSSQHHTNSSIYHFPTSTNHFQPMLLTTSMTMEHLPPTNNLIEADAVEFLNKQVNNDSIESGENI
MSESVLHGKSLQLGREYDQMHQPESSKKRSQSPVDHKNKRSVKPKKNMKSSVADDEETGNN
NNNNTVLHRQSSFSCCSEDESNVSSYDIYGLASSDNSKGVSLPNGKSRANRGSATDPQSLYARK
RRERINERLRILQSLVPNGTKVDISTMLEEAVQYVKFLQLQIKLLSSDDLWMYSPIAYNGMDIGL
DLKIGIPNPKP

>SlbHLH023
MAGEEESSLDGGTYSELLFADDDDGLAGCFNFTNSSSPKMLCFGTDAPILETCSVQTSEQKTPK
SELTCSGDSPSACSSSNISQPNNSNKRRNGAEKEPVEKTKGRNQRNCKRTKMVENSNVTTHAK
VKKEKLGERITALQQLVSPFGKTDTASVLHEAMGYIRFLHDQVHVLCSPYLQRQTQRQSPSLR
EGGETEASRNEVLLRSKGLCLVPVEVSVHVADTSLNGADFWSPAAMMNNNNSITQ

>SlbHLH024
MNIDGENNHGLPWETNDFWSYLNDNQVGSEETFDGDKVPGPTKSDICQQLTIVNEVVEVTPAV
GKKRSPPNRKSNGKGIAEPNLDVGGAEGKRESEHEIHIWTERERRKKMRTLFETLHALVPNLPA
KADKSTIVYEAVNHIVKLQNTFKKLKSQKLEKLEEYNIGLAGSQKVYNSWEKYVVDQGSTCN
STAITPTNHGASPLIPTGFMTWSSPNVILNVCGEDAHISVCCPKKSGLFTIICYVLEKHKIDIVSA
QISCDQFRSMFMIQAHAKSGRDVAQFSEAFTVEERLKQAATEIMALATSK

>SlbHLH025
MNEGGENNHDHLCDDMWSHLDWNDHQVESGEIEGNKLLDPTGSDTCQPLTFINEVVDVSVN
VAKKRSSANRKKKGKKIAEPNSSVDGAEVRRASKHEVHKWTERERRKKMRTLFETLHALVPN

LPVKADKSKIVYEAANHIRNLQNTFNKLESQKLERLEENNIMLVGSQKVGNSWEKYGGDQGSI
CNSKAITPANHGPTGFMTWSSPNVVLNVAGEDAHISVCCPKKPGLFTTICYVLEKHKIDTVSAK
ISSDQFRSMFMIQAHAKGGSGVAQFSEGFTVEDMYKQAANEIMLMTTPK

>SlbHLH026
MNENGENNHDHLCDDIWSYLDWNDHQVVNGETEGNKLLDPTGSDTCEPLTVINEVVEVSVN
VDKKRSSANRKRNGKEIVEPNSGVDGAEVRRASKHEVRKWTERERRKKMRTQFETLHALVP
NLPAKADMSKIVYEAVNRIRKLKNTFKKLESQKLKRLEEYNIRLTGLQKVNNSWEKYVGDQG
STCNSIAITPTNHGASPLIPTGFMTWSSPNVILNVSGEDAHISVCCPKKPRLFTTICYVLEKHKIDI
VSAQISSDQFKSMFMIQAHAKDGSGVAQFSEAFTVEDMYKQVANEIMLMTTPK

>SlbHLH027
MNIALPEMLHNITSNGSSELSVLDRTKWQVQQQEMSYFNGQNDQLMNSFHQTAEAQQFHGLI
NVNDQSLNELVTRAIKPDPCMENSWGGFGTTGTNGFDYVPVGVGHGGMSHPSEMNYAISRTT
SCPPTMADNVVKPKDTRLSSNRGRESFKKRKADKNQHLKEVAEEETKDKKLKECIEEEDDSSK
VTTEKKSNKRSATNSSNSKENSDTSKEKSKITDDKKLDYIHVRARRGQATDSHSLAERVRREKI
SERMRFLQDLVPGCNKITGKAGMLDEIINYVQSLQRQVEFLSMKLAAVNPRLDIDADNFFNKDI
FATSTSTFSAVGAGTSSEMLSMAQRQFNSLQQIMSSSGLEMGIVNLNEMALRRTTSAPVPIPEM
FLDSSSINQVQSFQTWNTDLDNMYAMELQQGRSAQFLPHPCTGFAEAGHDLKMEM

>SlbHLH028
MDQFNHGGLYQSNQLPNHCLTELNQLPSDVSTPPNGLLSESSKQKPEAELKDSIAARKVQKAD
REKLRRDRLNEQFMELGKTLDPDRPKNDKASILSDTVQILKDLTAQVSRLKSEYAALTDESREL
TQEKNDLREEKASLKSDIESLNAQYQQRMRTMYPWAGMDHSMVMHPPSYPYPMPVPIPTGPV
PMHPPLQPYPFFGNHNPAVVPNPSSFVQYMTPNTLIEQQPTQYMSPIIQPGSMTRQESRNKSSDQ
GESRIEKSEDSNEVATDLELKTPGSTSEQDLSSGQKKSRKLPRKDNSFTDGSSSSKCSSSHSVHA
VSSNSVVRGTKTGD

>SlbHLH029
MSSEKSILEYSNLANNNNPNFYLMSQNWWSQVDNNTIFHPISTNSSLSSCNTNRSSSCEEDVISI
SNNNNNNGNSVTNNNNNNNNLHQYQFLLGVDEINNSSLISGENIAANSHDHNQHIWSQLLLSNS
TTSGSSGSINTKNLLENNMEMSLLDHGSTTTLITSKSCFDQNQLANNHDDDDDYTFHPYSNLL
GRPLNYYTNNNNNNNNDMDSMFPMRSDQIAHHEEVDNSRNLASICFGDYLNNKPLVDFKPSLK
TLNLGQHKKNGLQQPYISKSRVTLSTCNTLKRSNGRSQEYNNPIDGKKRKLEDNLETNSKRLK
NENSDVTSTKTQIPKVKLADKVTALQQIVSPFGKTDTASVLWETINYVRFLQEQIQLLSHAYMK
SNTCKERYWGVFDRKEIDLRSKGLCLVPISCTPQIYHETNNGSDYLIPSYRGCLYR

>SlbHLH030
MLQMSVLERQRAVLERIYNHSKQQLSSLVPQQELAHLITGCVQGNFNMFGGGDSNFVNFQEM
ARPSFSTISNSSITTVSPPPEKESDLSSMIAPRENVVSTKKRKAEFIEEEDCEKSPGNDSKENSKTS
EVQKPDYIHVRARRGQATDSHSLAERARREKISKKMKYLQDLVPGCNKVTGKAGMLDEIINY
VQSLQKQVEFLSMKLATLNPRLDLNTDNIFVKDLPSYMTTTFPPTVAVPTLSEYNMIQHQQAGS
TGDVAQMLPQRRDLMSFPDTYLGSSHVTVVQPQQPTFEPDLQSLFSVGFN

>SlbHLH031
MELSEHDILEELLALPRKESLNDFLPHGNGWTFESPLPFYQNPEFIALNSSLLGLISPPITTSHSNF
PDFTSPESYQFLDSSFTGTPLLDDYSVMENHEEFGGIIPGDFHGLQQELNSFGDVKVEESNSRL
MGNVGEKKSKIRKMEGQPSKNLMAERRRRKRLNDRLSMLRSIVPKISKMDRTSILGDAIDYM
KELLEKIHALREDDNVKDEIKDIKFVGNFKELKPNEALVKKPPKFEVERRNADTRIEICCSAKP
GLLLSTVSTLEALGLDVQQCVISCFSDFSLQASCSEAREHRTILSGEDVKQTLFKTAGFGGRCL

>SlbHLH032
MGSQPKKSSSLRLRRSRRNSSVEKRSKNGVVNNSSNAAGDDNSISISEKLEALKQLLPVNNGEL
KADQLFEETADYIVLLRTQIFVLQKLLDFCDDASGQSQHINAV

>SlbHLH033
MSGRRSRTQSSEGGTSRISDDQIIQLVSKLQQLLPEIRNRRSNKASASKVLQETCNYIRNLHKEV
DDLSDRLSQLLSTIDADSPEAAIIRSLIM

>SlbHLH034
MQNNYQFSPQIQKPFVSLEDQAAGGNIFDIPVPSVFDTMALPTSFKSSVPFHGFEFRSSEACPKN
FIIFDQTDYRSQIMYHPAMTSKFPYPDLNYNSTCFHDCMERKIANNENTEVSSYLKEDSDDINA
LLSLEEEECEEYDEEEVSTARTDANYGCSSPESYSNYHCQSKKSRTSSFRESSGSSTSNCSERKR
RKLKKMVKALKGIVPGASRMNTVTVLDEAVRYLKSLKVEVQKLGVDNLKTYA

>SlbHLH035
MNCRNLGEFCENEAKGVVQSLVLDSEKGELVKASGRVEKKIGKSEGKTIAALKSHSEAERRRR
QRINAHLSTLRNLVPSSDKMDKAALLAEVVRQVKQLKETATHDSERFFIPLDSDEIKVEIIAENA
IDGTCLFRASVCCEYRTHLLSDLKQTINSLHVNLVKSEISTLGSRVKNVFLFTNSIHGGGGCATIQ
ARDIFLSSVRQAFSSVLDKVSAFPEYSAYPNKRQRVSCFDSSSLLF

>SlbHLH036
MEQQYCSNKPSSSSSCKAADRKTIEKNRRNQMKDLYMKLNSLVHHDQHTKEFLSLPDQLEEA
ANYIKKLQIDLEKMRLKKEALTATAGTLNSNSNSSSNTDGRTLENTLPLPHIDIHNVNSALEVLL
ITGYDYHFMFNHIIRMLHEDGVQIISANYTLVGHTIFHSIHSKVGESATSSTAKIISEKLKQFVGA
AAT

>SlbHLH037
MADEYQASVCGGNWWNSSRSIFGSSLCASSVPLGNSNFAWTNDHLLDMKSSCRSNDESGNSD
ESVVLQELPKHDSTLQILGSGLNSSSTNDNWSHTLMHGNDRSESSYPSILQQEDINSSMNYQQE
SGVDCSSNSFKQDFTLGMNHPITSSTNTHHDQISSTFPMNSSFSNYPSALLQTLFDNDPPQQQQL
QQSLFATNNNQPMNFPTSSLNYRPDLNDFSPSMPKFPNSLLIPKQTTTPSNHFPNYSLNATASLY
NTSSATSLNNMRATLMPSMHPQILQSPTFNDNSRAPNVTPKSKVEDLRESRVSKKSVTNEATLK
RARIETPSPLPTFKVRKEKLGDRITALQQLVSPFGKTDTASVLHEAIEYIKFLHDQVNALSTPYL
KNGSTTTQHQQIADKVKEEDLRSRGLCLVPISSTFPVATESSTDFWTPNFGGTFR

>SlbHLH038
MEFPSTPFDNSNNSEEREVGRRTDKRKQIDGEVKEYKSKNLKAERNRRQKLSERLLQLRSLVP

NITNMTKETIITDAITYIRELQMNVDNLSEQLLEMEATQGEELETKNEEIIDTADEMGKWGIEPE
VQVANIGPTKLWIKIVCQKKRGGLTKLMEAMNALGFDINDTSATASKGAILITSSVEVVRGGLT
EANRIREILLEIIHGIY

>SlbHLH039
MAETEEDGSNWLIELGLMEDLPSLEPNAQWPSNAFSLPNNLSSGLEDSYGNSDSLKECGSKKR
VRSGACASDSKAHREKMRRDKLNDRFQELSSILEPGKQPKMDKSVILGDAVRMVVQLRDEAQ
KLKESNNNLQEKVIELKAEKNELRDEKQKLKAEKDKLEQHLKAMNTQPGFLPHPPAMPSPFS
APHQVFASKMMPYIGYPGIPMWQFVPPAAVDTSEDHSLRPPVA

>SlbHLH040
MDSTNLYNHHQLQQLAGYPFFSTGVSTLHDWNSGITSEEEYYYKLGHMKRISSEELMWKRGI
DTFPLMNTSMFHDGHHESSNDPLDDKNNKAGYIISNDYFLKMKDMNSLSNNMFKESYFENEQ
QHAFDLNENLLSEDSYMNNANNSSYVSSHDMEYSDFQGLKLAFNGLTFKNSHDSNSNCFGHF
TTERMSSGFADGLQELTHSPSSKKITSNVRKNIGVSSKAKRSAEDEANPCQEASKKSRVTSQSPS
TLMLKVRKEKLGDRISALHRLVAPFGKTDTASVLTEAIGYIQFLQDQILTLSMPYSKSTERKLHH
INLKDSSIEAVLDLESRGLCLVPTSFSSYISQSCD

>SlbHLH041
MSSRRSRQSSTGSSRISDDQIIELVSKLQQLLPEIRNRRSSKASASKVLQETCNYIRNLNRQVDD
LSDRLSQLLSTIDADSPEAAIIRSLLM

>SlbHLH042
MGSRSKLSPSSLSMRRTRRSTPHKKTCKSQNLVDVDGGSVSEKLEALKQLIPANYGEIKADQLF
KETADYIVLLRTQVFVLQKLVDFYGSNTDQNPV

>SlbHLH043
MDLQNIAHSLELGFSNSNIEMIPLQSPLHNSNYLMNSPPSNFSFMGNPIEEPAAMPILSSIDEIIAS
THGNGNDYSCLQRRNSMEAMREMIFRIAMMQPIHIDPESVKPPKRKNVKISKDPQSVAARHRR
ERISERIRILQRLVPGGTKMDTASMLDEAVHYVKFLKKQLQSLEQAAVNNRPMISGFSTAMSSP
GGPMNYNSSSIRACQPHQSMNSGAQMLS

>SlbHLH044
MSMALAKEHVIMSDTKMGMVDNYDQYYEGEFGINDHSSPELYGIHEEPPKSIFEECENSEKTS
PKIAKNFALSSSNSSLSSPSSSNSNAQSVINFKGVYGNFMHSANGSLLSFEQSERFCPNPRMISNI
NQVEGSVWEDNNLHYQNCVTPKGSSNTSPRVINDNSNNNGIPFGWLNSEANASTTTHIDESRF
NKRPSTEESMQTNKKQCSAGSKKGKPNNNNNNSIGTKDPQSIAAKNRRERISERLKILQELVPN
GSKVDLVTMLEKAIGYVKFLQLQVKVLATDEFWPTQGGKAPDISQVKEAIDAILATQRDRN

>SlbHLH045
MELTQEDFLEEIVSPRIENWNNTFANAWNIESPTFYQQNPEFIPSNSSLLDLIMSPSQSNYFPCPD
FQESSYPFLHSFTTTTPPQLVIDSTTYNNNNIQERAIIEEGQIGHFSTDFHGHYEDSFSCYNINKV
VKMEEATSRIVGEKKSKNYKVKKVEGQPSKNLMAERRRRKRLNDRLSMLRSIVPKISKMDRT
SILGDTIDYVKELLDKINRLHEENEIKDIKFLGNFKGLKTNEALVRNPPKFDVERRNEDETSIEIC

CGTKPGLLLSTVHTMEALGLEVQQCVVSCFSDFSMRASCSESVDHRTILSSEDVKQALFKTAG
YGGRCV

>SlbHLH046
MDNSSLSQWFSRTEEGVFYSNRNNSIDDFTTQKSTISEDQETSELSITPDSARSNSRSYFHKEISK
CKASKINFSLITPMENFPIGSKRPATDHPQQPKLNKISSSSHRFLSFNNNNNNTDSVFPSHQGCKI
EAMDDMYLCDSSDQMFYPYVKSNGCYDAKDKKGGKKIPSVELQDHIIAERKRREKLSQRFVA
LSTILPGLKKVDKASILEQAIKHVKDLKEKVQLLEEEKKSVMFVNKYKVETEEYTSSEENNSGS
DLPADIEVRFSDNNVLIRITCARRNAFVLNIHSEIEKLHLTIVQSSMMPFGKQAIDITLVAQMEES
FCMTLKDVAKHVRMVTGRLMTQA

>SlbHLH047
MDTWWSEMETSNMNDHNFVNQSQAMTQFNEFPFNSKPFSTFTPPPPSNVISNYSYSNSMSTKN
QFEKLNTFKVVKHEVPSGTTINFSSSVNSMDDSDFGDIEAAMGFGAAITTTTDQKKSYNRTSV
QAQDHVLAERKRRERLTQRFIALSTLIPNLKKLDKATVLGDAIQYIKELEEQVKTLEEKNKKCS
EEPVIPPAKRPRLVSSCADSSSSDEISSVSTVCTDRSLPDIEVRASDGNILIRIYCKKQNGMMKEIF
NEVEKLHLSIISCSVMPFGYNTSHITIIAQMDHKLTSNTPNHVANRIRAAMVKEEANSFTA

>SlbHLH048
MDISSATWWSEMDVMNMNELQYIDQTFDDFAFSDNIQVSQIGEIFEEKPALCSSITQNTSTSPPC
SSPSVISFSNSNSPSATPTTTNAQNYFKNLNTSSLKTEVPSGTTINFSSSNTSSDSDYDDSKQLFQ
AMGFGAGQSKKMNYSRTPLQAQDHVLAERKRRERLTQYFVTLSTLIPNLKKLDKASILGDAIT
YIKQLEEQVKRLDEEANKQPVKRSRLHSNYDNFSTCNENSNKSVVPEIDVRVSDGNVLIRVCC
KKQAGIIKEIFSQVEMFQLTITSSSVIPFGYDTTHITIVAQMDHQLNMATEQVANNIRLSIMKLINS
HK

>SlbHLH049
MANNNVYYHNANFSLTDPDPEPDDISVFLRHILLPSSSSSSSSNFMALKSNEMQYSSSLPHLM
PNNNQQGNLSSMMNSSACGIFSSSYGVCNGATTVSSSSVGTIDYDPDEYECESEDGTEDLGAE
ASVQPPSRNTSKRSRAAEVHNLSEKRRRSRINEKMKALQKLIPNSNKTDKASMLDEAIEYLKQ
LQLQVQMLTMRNGLNMYPLGLPRMLQQNQLSHQKVGLCEGNAFTNAKVAGNLQVNQDASL
NAIFNPTENCTETKVTPPITMSNINRSDSAFELESSMNIHLDPFQLSRSTSKEIWREDDLPLYGMN
ELTTKTASTGSNLAFSVPLDTDASNLKRSTREACLLRYQFGAVNETNLDCDQLLSQQLYSNF

>SlbHLH050
MADNPPEVYAADDFLEQILAIPSYASLPVTDLTAGASSENSTSGVSQLQQQPLFPLGLSLDNGFA
DANNTGGFQVKTEREAMNMGNLYPGLEHLQSHAVCLSVPQVHQVQPFQGHPTSSAIVTIPHQP
AIRPRVRARRGQATDPHSIAERLRRERISERIKALQELAPSCNKTDRAAMLDEILDYVKFLRLQV
KVLSMSRLGGTSAAAQVVADIPLQSVEGDTCESHSNQRVWEKWSDSETEQEVAKLMEEDVGT
AMQYLQSKSLCIMPISLAALIYPTQQSDNQSMVKPEQAAPL

>SlbHLH051
MFSLQGSDDLLIQILSNTCQQNKSSLDAVMDFASTDTITRTHIPPNINSQKKIISKRKLSTHSNKN
DGIASDDHFKLKKIIHRDIERQRRQEMAALYSSLRSLLPLQYVKGKRSVSDHMHEAANYIKEM

QGNIKELEKRRDLLMKSIRHGNENADKNNRFTDCTVTVSPWLQEGIEISISVDCEGKTFPLSKIL
GELLKQGLNVVSCVSAKADQRSLYSIHTEACDMNNIDHLALQQKVIDMINLDL

>SlbHLH052
MDGDQNLSDLFDDSECDIFGILEALEGGGGGGGNSGITSKFNDNINNQTATIATTITTTTSDEITG
LVSEEGKKRKLISQKSTGSCATLQEEETIENKISHITVERNRRKQMNEHLSVLRTLMPCFYAKRG
DQASIIGGVVDYINELQQVLQSLEAKKQRKVYSEVLSPRVLPPQLVPISPRLLTPSPLSPRKPPLS
PRMNLPISPRTPQPTSPYKPNANANKPPEPSPTTSSNSSIDSHVNNELAANSKSAIADVEVKFSG
AGANVILKTVSPRIPGQAVKIIAALEQLALEILHVSISTIDGTMLNSFTIKIGIECQLSAEELAHQIQ
QTFC

>SlbHLH053
MADEYFQDGVYGESLWINSTKNIFSLSSSTNSILDPIIGRYNFAWPNDDQFLDMKNMSNNNDD
YYSSSDDSIVFQEFPKNHNLGINENSSSPDWNHSINDSMLQEKLNSRENYPNHNELKRNFSSIIS
EDNSLIKPIMNQDFTNYYSDLLQTLFANTDLHEEQPQQQSFNYPSSSINHRQKLKDFVPSLPKFD
IEEIGESKLSSITKNNTNEQTNKRQRIETPSSLPTFKVRKEKLGDRITALQQLVSPFGKTDTASVL
QEAIEYINFLHDQVNVFSNRYMKNGPPTQHQQVKELQEGLKQGLRSKGLCLVPISSTFPLAAET
TMEFWTPTLMGTTVR

>SlbHLH054
MSILERQRAIFEHLYQCQQQQQTSNSLPNQNLAQLNSLMSENTMEFSQFQNFPFKIDTQIDFGSE
KRNWTSKKRKSEVYEEYECKVERLDGEAGEVKTEMIVKTEKGKNSKENLEAKNTDFIHVRAR
RGQATDSHSLAERARREKISKKMKCLQDLVPGCNKVIGKAGMLDEIINYVQSLQKQVEFLSIK
LATSNVNTDNLFAKELPNPTFLQQQGNINIGVTQRRENCLMSFPEAVLDSSNVLALQQLPNLET
DLQCLFGVRFQK

>SlbHLH055
MDLNEITQSINNIEMIQLLPIHNSSSIDHVIMREMIFGIAMMQPIKIDPESVKPPKRKNVKISKDPR
SVAARHRREKISEKIRILQRLVPGGTKMDTASMLDEAVHYVKFLKKQLKSLEQATTACSNDRN
NNRFVSGFNQCPHNCY

>SlbHLH056
MENSFSSEYCDVNDFLVQNYLPQCSHEGRESASRSHSEAEKRRRDRINAQLSTLRKLIPTWEK
MDKAALLGSVVDHVKDLKDKTAEISNVLNTPTDTDEVSIEHLNEEEDNKGCLIKASFCCDDRP
ELFSELQRGIKNLQLRMMEADITSLGGRIKCVFMLSPNDNYVCINSLEKSLKAVLSRIAISPSTSN
YRIKSKRQRFFLPPQFS

>SlbHLH057
MAAFSSHQLQHNNPFLLDSVFLPTSPIKMSGFFEEPNNSCIVQQFYQQEFPSNLISHENSFCLDP
KSSSSISLDMDASSVTDKIESGINNNKANVSPLDKKRKSSEGSSSMTSAHSKNEKQGDNGKKK
KIISKLVAKDEKKANEEAPTGYIHVRARRGQATDSHSLAERVRREKISERMKILQSLVPGCDKV
TGKALMLDEIINYVQSLQNQVEFLSMKLTSLNPMYYDFGMDLDALMVRPDDQSLSGLETQM
ANIQQGSTTTTSQAAEVIANTNSGYQFLDNSTSLMFQQSHFPNSIPQGIGQLLWGADEQTQKIIN
QSGFSNNFCSFH

>SlbHLH058
MDPPIINEGSFSAANPSSYSLAEIWPFAAAANGGGNGELGGGGLGLRMSSFTGLLEAAANSINE
STLTEQSRRSGGAGGGDGSGSGGGNVGVRKRDVNSEDDFSKFVSTSDANDLFSDLTKQLIDIHI
NSNHVQDGSVAKRLKVSQSKEENGVSKVEAESSSQTANKGTEQSSKPEPPKDYIHVRARRGQA
TDSHSLAERARREKISERMKILQDLVPGCNKVIGKALVLDEIINYIQSLQRQVEFLSMKLEAVNS
RMNHPIETFPSKDLAPSAFDTSGMIFGTQAPREYAQGTQSEWLHMQVGNSFDRAT

>SlbHLH059
MENTSPEKLDRKTQEKNRRIQMKYLSSKLFSLIPPHHHQYSAKDMVTQQDQIDQAITYIEKLKE
RVDVLMRRKDKIIAQGTSDDSKKFMPSTSCSNIKLPMIEVRELGSTIEVILVSCLQKKFTMQEVII
ILEEEGVQVVTANFSTIGDKVYYTIHAQVKITRLGVDASRVYLRLQNLIC

>SlbHLH060
MENNSVNNIVSTSSVQKLDRKTQEKNRRIQMKYLYAKLFSLIPLNHSKEVLTQHDHVDQATTFI
EELKERVEVLKRRKDEVVAQIIGDDSKKSISTTTCTIKVRELDSTLEVILTSGLQKNFTLQEVIKII
EEEGAQVVTANYSTIDGTIYYTIHAQVKITRLGIDASRIHFRLQKLVS

>SlbHLH061
MESSNINIVTASEKIERKTQEKIRRIQMKYLTSKLFSLIPPHHHQSTKEVLTKQDKIDAAITYIKQL
KERIEVLERRKEEVVAQETCDDSKKSMPTTTCSIKSPMVEVKELDSTLEVILVSGLQKNFILQEV
IKIIEQEGAQVVSANYSTIDDTIYYMIHAQVKIARLGIDASRVHLRLQKMVC

>SlbHLH062
MELPQPRPFGTEGRKTTHDFLSLYSPVQQDPRPPQGGYLKTHDFLQPLEQAEKTLREEETNVEV
ATVEKPPPPVAATPSGEHILPGGIGTFSISYLHQRIPKPEASLFSVAQASSTDRNDENSNCSSFTGS
GFTLWDESAVKKGKTGKENSGGDRHVLREGGVNTGGVQPTTSLEWQSQSSSNHKHNTTALSS
LSSAHQSSPLKSQSFLHMITSAKSAQDDDDDDEDFVIKKEPQSHLRGSLSVKVDGKGNDQKPS
TPRSKHSATEQRRRSKINDRFQMLRGIIPNSDQKRDKASFLLEVIEYIHFLQEKVHKYEESYQG
WDNEPPKLPLSKCHRTTHGVSNLPQRIINASSASLTYAGKFDESIMGISSANPINVQKLEPNISST
GLKDKGQQPGLTNKPTTVPMHPNTFSFSGTSSTAALYSSKLIADTDKLESKSHSQFSLSRSHMT
DYAIPNANPERLELPIESGTISISSAYSQGLLNTLTQALHSSGVDLTQANISVQIDLGKRANGRVN
SSASTVKGDNVSTSNQPIPKSRVTTTREEPDHAFKRRKTS

>SlbHLH063
MSHCTVPTWNPSYQRQEHVVEAEEANKYPHLHNQQIQINHLLPMSSKCEEVAELTWEKGQLG
MHGLGGILSISQAKQTLGRTGDTLESIVHQATYHAKNQTSIHQNYAQNEDQDLKTGVLYSGGK
WGESSQQMAPPRATVLAKKRMRPSESDPQYGGAEDHEYAEGSACASASATFYRENDTTMVT
WPSFDESSRSIKSKTACDEDSACHGGGSENKEEEHETKRSNSSRRSRAAAVHNQSERRRRDRIN
QRMKALQRLVPNASKTDKASMLDEVIDYLKQLQAQVQLMSSTPRNMAPQMMMPLGMHQHI
QMSLLARMGVGVGLGMGMGMFDMTALARAAAASATTHPNQMTTAPINIPFTPSGAFALPAAP
ANSVSPASATTSTTTNSIPFTNPYSAFLPQSMDMEFFNNMAALYRQQLANQSTQITGSKLNQEN

QTE

>SlbHLH064
MDTENPAPIVEKDTTDAETSLDSSHLRKKIQKKVPKRIHKAEREKMKREHLNELFLGLADALE
LSEQMNGKASVLSEAARFVKDMLSQIKHMRTENTTLLSESQYLSVEKKELEDENTVLEAEISK
LQNEVKAREAETSLDLNLAPPEIHHTEFASQTNYMRLPASEHGFQQSQMMNPVYVFPLSSNPQ
AYPAPDAADPMAMPSSTVKKPQPRYPTPNDVWPSQILEKRPQLLRQEVQDGA

>SlbHLH065
MGWIDGKEDGGTGSWVNQNNENHQQNNGGFPNENHQLNNGGFTNFQGMVDDGGVDWFM
GGGDSNNHHINNNNNNNGGGGGGGSNMQSHISYSTSFTEAENSLLLQPVDSSASCSPVSGNVFN
NIDPSQVNFFMPQKSTIPSSLTGLSNNPMDNSFNLGMLNQAGNGMMNTGYHHLGSPNQMGTN
NLSSYTQFSSPNLLQLPQVAGGYSSMGFGANNSANGNTLFLNRSRTHKPLDNFASIGAQPTLFQ
KRIAKNLVSNGENLGTEIGQSSSNLTDKKRKSSMNDEFEDVSMDGTLNYDSDEFMDISNKMED
GIKIGDSSNAASTVSGADQKGKKKGPPAKNLMAERRRRKKLNDRLYMLRSVVPKITKMDRAS
ILGDAIKYLKELLHDINELHNELESTPANNSSLSPATSFHPLTPTASALPSRIKEELVPSPLSSPTGQ
PARIEVRVREGKAVNIHMICSRKPGVLLSTMKALDSLGLDIQQAVISCFNGFVLDVFRAEQSNE
GQEMHPDQIKAVLMETAGFQGGTI

>SlbHLH066
MADHQFNSDIQNFITNSPFSLLNFDSSVDLMNQFPDMMTIPCSSDMSSFNIQSSMEFSNDNVFT
QVNDQFPGSLQEIFQGNIQQESKNEEINDSKKRKISDTPESSSASATGNKRRNTKGRGNRVKVD
EKEEEKPREVVHVRAKRGQATDSHSLAERVRRGKINERLRCLQDIVPGCYKTMGMAGMLDEII
NYVQSLQNQVEFLSMKLTAASSYYDFNSESDILVSLQRAKAYEALKMQKMMKKEIECEVMST
NQVGLHFGSYPMLPYNT

>SlbHLH067
MHPHESHELSSSTTTTQHFYGSSNFLADNNPSQDHLPRSWSQLLLSGLSCDQEKPDISDHFQHQ
YKKLENWEEIQNLNSIHNNNIIPSNSSFRVPIFDVKPEELVSQRLYSNYHHDLSPASSCVTTNLNH
NNIFNFSSSPANKITNSNKVVEVKHQDHSSECNSTSNGGVTKKARVQHSSAQPSLKVRKEKLG
DRITALHQLVSPFGKTDTASVLSEAIGYIRFLQAQIQALSSPYMGNVAGSMGHTQQQSADLRSR
GLCLVPISCTQHVGSDNNNTVGDYWAPALGGGGYL

>SlbHLH068
MENLDWNETPFNFGSSFDPIHVISNWNMPQRQEAANRLAADSMAAKAGAADLTRDCTDIAFS
SSPILNMPNNYITDNNPLGLMSDFGVEIAKPISNTVSLESIDCLLSATTTNNTDTSIEDDGMSVIF
ADSNSLWNSGESAGINPQLLNCLPTDHKILEEEQNQRKRKSYELDEAVSQSSLGNSSNEFNLFQ
SNSLGDCGNFQLISEKQSKSKKMRLIESSNKRPSSSNINFQQASSSVSSIDQEPDPEAIAQMKEMI
YRAAAFRPVDFGAEVLEKPKRKNVKISTDPQTVAARQRRERISERIRVLQRLVPGGSKMDTAS
MLDEAANYLKFLRTQVKALEAIGQKQDPFTSITQFNYPIPMQLPHFPLQNPNQIHRPKS

>SlbHLH069
MDPHSTIMSAFQTATNLAEIWPYHHLLDHTTNHAATKRRDDDESAIAVSTSGNALTESDSKRLK
ATRSNENGEYSGGNSGKSSDQPAKPPAEPPKDYIHVRARRGQATDSHSLAERARREKISDRMKI

LQDLVPGCNKVIGKALVLDEIINYVQSLQRQVEFLSMKLEAVNTRVTPTIEGIPTKDFGQQTFET
NAMAFGSQGTREYAGGTSPDWLHMQIGGGFERTT

>SlbHLH070
MNIVSMDDAEPKDWQFSGSNLINASNRIIDSFCVSVWGNPISLSNVGLCGTNIPMNPGAATVLD
WTQPNAMSKGGTFSMVPQSLTQLTADSEKVGDMMNPFAIPNSFNTYHNGLIFGESQDVFDSPS
TAISQKKQMRNAVESSDQDVPLPLEYEHTDRSPLKNDKNVNFVKSQGEAKECVGISENESECS
GHQEEVEGGYSSARCLGSRKRKRSGQDAEFDQMNGAQQQPAELAKEQNLNSILRREKISERM
KFLQDLVPGCNKREPVISQVTGKAVMLDEIINYVQSLQRQVEMKLATINPRLDFNIDALLAKEIL
HSRAGTSSSLAFAPDLTVPYQSSHQLQRGLVHSGLPGSGNSIDTLLKSIYPFLAVTSGGYKERSS
QLPNDELHNVVEMGLCTSAPLHIREGSLPSGQMKEEP

>SlbHLH071
MRLVLLRRMPHLLAVIPKVYGKSNEKASALRSKHSVTEQRRRSKINERFQILRDLIPHTDQKRD
TASFLLEVIQYVQYLQEKVQKYEGPYQPWSSEPTKLMPWRNSHWRMQSLPAQPHALKNGSGP
ESTYLGRFDENLATVTSTMQPNQQNPIESHTSRDVSFKALDQQNELANKSITTPIPLQAGMKMS
VPNNSAFSEPQPRPVSDQCPNTIDALNHDEDDVIDGGRISLSSSYSQGFLTSLSQALQSTGLDLS
KATISVQIDFGKRANQAMTSGPSIAKDDENPTLSGHQHTDHFREASNDEDMNQAQKRLKI

>SlbHLH072
MSNRRTRGSRQSSGASRISDDQIADLVSKLQLLIPESRSTRSSDKVEASKVLQEICNYIRSLHREV
EDLSDRLSVLLESTESDSAQAAIIRSLFM

>SlbHLH073
MMNSQEIMDAKALAASKSHSEAERRRRERINNHLAKLRSLLPNTTKTDKASLLAEVIQHVKEL
KRQTSQIAQTNPLIPTEINELTVDYCNNEEGNFMIKVSLCCEDRSDLLHDLIKTLKSLRLKTLKA
EITTLGGRVRNVLFITRDQQQDNDDTWPINDNNNDNDDDQMKYCLRSIQEALKEVMEKSNGN
DSGNSGSIKRQRTSNNNNIHY

>SlbHLH074
MGDSSSSTPLDFHALNSTCNNNSSILMNSNMELLNSISQQLENDQNFSSNNIHQQHGFLSLSSN
DQNFSNHHQHELNIMSNFHNDHHMNNISHDVYDPAVAVAAAQFFTLGGPSYGCTSSIPESESML
NSSNNNINIPTPHPLVSGNTTSKNTSEGRKRKRNNQKEVEKPREVVHVRAKRGQATDSHSLAE
RLRREKINEKLRCLQELVPGCYKTMGMAVMLDVIINYVRSLQNQIDFLSMKLSAASLFYDFNS
SEMDDMDSMQGTNGYAAAQGMGKNIVGEGYGGFPQFQTSWPL

>SlbHLH075
MLARFGVCYVAEIETNNGSGITSRRNEDGLYQYPKASCVEMHKMNERRRRYKIAKKMKVLET
LIPNCNKSDRASVLDQAIQHIQALQHQIQVMSMDRIRGSTLVAAGRNQIMQSTLHFNPYIGAIG
YFFNFSNILCSNFSPMLSTGSEFPFLPLAVACNLLHPGPIMEVFTRGSASVAPLEKRV

>SlbHLH076
MYQFPSFYELGNTCSDSYNNFLHEIITSSSSEMFNNINNLESSSVSPRSMAEAKAIAANKSHSEA
ERRRRKRINGHLATLRNLLPNTIKTDKASLLAEAVRCVRELKQTTSELGATTTTTTMSENDDDD

DHTTLMTKIMFPSESDELNLSYCNESNNNNTDNNNDDRNLIIKASMCCEDRPEIMMELRRALS
TVEGKIVRAEMSTVGGRIKCILWLEMLENGCKEGLFVQLRRALKVVMDKANFGPQNMGQDL
LGNNKRPRLLGGPINYAT

>SlbHLH077
MDCLSEIFSSKKIKDHDQQQLIQIPSTPCQPHHQDLPTNDPNYKRKRSISDHKERDSLTRKKSVQ
KKVLHRDVERHRRQEMSNLYATLRSHLPHIKGKRSLPDDMQQAVNHIKNLENNIKELEIKRKK
LEDLVLCSSKEDKHFGDYVKINLCDCGVEILINERIPLSRVLEELVKRQLNVVSCVSTKVDERLL
HRIQIEESDVSCMCMDGLEQKLAEMIVFHS

>SlbHLH078
MFTRSRSILMALETVIYNQETFGYGCKEYYNNNLGNFSYNYELGLTQGEGEMSFTNLLDHNN
NNNREESSSSPTELMYNNNAREYNIWDPNSSLEDHHLFMEGSPAAELPAAAVRSAATSGRRKR
RRTRSCKNKEELENQRMTHIAVERNRRKQMNEYLAVIRSLMPPSYVQRGDQASIIGGAINFVKE
LEHHLQTLEAQKRSHPQKQEHSDNHGSSSTPPFADYFAFPQYSTHSKSTSPTAAASDVAAAGSC
NSPLATEKMSALADIEVSMAESHANLKILSKRRPKQLLKIVAGLQCLWLTVLHLNVTTVDHMV
LYSLSLKLEEGCQLTTADEIADSVNQLLGRIQEEAASSS

>SlbHLH079
MTPQSNEIPLNGDSPYFTFPGLPVSNASWPAETCNWLYYSPLFYQGFNPVSTTLPKEKLAPRAL
ENLEGSKHPNGGTTSTQKRFLVFDQSGDQTTLIYNSANGTHVQCPASLNPKAPALYKEDPEIKR
NETSPFGHFFGDEYYEENNRDDVESEMHEDTEELNALLYSDDDYNYSEDDEETSTGHSPSTMT
THDMRECFDGRGEEVASSAGVTKRHKLLDGSYDAPELRDTATSAKAYTCSDLEDDAQSSCGN
GLEQDSGAPDSPSGKKRLRKDKIRETISILQEIIPGGKGKDSMVVIDEAIHYLRSLKMKAKSLGL
DSL

>SlbHLH080
MLSSLQGCNNVFLQLVDHQKNEKSNTTKKSKSKDAAVTHAVAERKRRERINSHLHTLKKLFPH
LPKKDKPRVLTEAVTQLKELRKNVAQQLELSSLFIPSENDVVIINYCDNINDERTVKTTICCEDRP
SLNRDLSSAIQSVQGRVIKAEMATVGGRTKAELVVVLGKANGGEKDVGQLKRALKAVVENRA
LGFGSNVMLGRRFG

>SlbHLH081
MEYYNENGFLEELLSLRSESWDTTNVVPMEMSHDFYSNVLNYDNIPLPCTTTSNSFEGYSCNL
PFDQQNLINCGTSFCSPFCDELSPPSSFPSQDDFSSILDDEVGNYSFQNLEMGNNSCNNDNNSNI
VIPCKLEENQVCEGAGGRVGGASSFNIGFCPEIRKSKSKKMDGQPSKNLMAERRRRKRLNDRL
SMLRSVVPKISKMDRTSILGDTIDYMKELLEKINNLQEEMELGPNQLSLMSIFKDVKPNEMLVR
NSPKFDVERRSVDTKVEICCAGKPGLLLSTVTTLEALGLEIQQCVISSFSDFAMQASCSEEMEQ
RGVVSSEDIKQALFRNAGYGGRCL

>SlbHLH082
MGTKENGSFNCPSTGMNRADSMPNVDPFSGSGWDPLLSLNQKGGFKGSSVVGHNEFVNLPY
QSSQFVHYPSDSNLAEMVPKIPAFGNESYSELVNTFPLQEQLRGANCYANYVKNRGISTEGECQ
ISGEGAVEVSPNGKRKISENHSLSNANKNVEGELQKAPSRDSSDCSKEQDGGKRHKTDQNVSS

NLRNKQAGKQVKDDSDGGEPPKDNYVHVRAKRGQATNSHSLAERVRRERISERMRLLQELVP
GCNKITGKAVMLDEIINYVQSLQQQVEFLSMKLATVNPELNFDIDRILSKEMLHQQTSNAALLG
LGPGLSSSLPFPGISHGSFAGIPATTPPFHPLPQNVWDNELQSLLQMGFDSTSSMNNMGPNGRSK
LDL

>SlbHLH083
MEHVGAFFDEEWESLSKLFSSTETADFMLQLQGDHGSMFSMNGSDNAGSSYRTDNPQVAFSQ
LSDEVNNNFQYLSQESSITSCGSDHGMFFTNPSHDHLQHSNNIDDVNNKLLQVAINNNLPDEY
QSIDFFDMDSKNLENLCINQDFQAEMVCDQLDNAGISPVPHKEMQLKRKCDKIAHENPKKKS
RGSQDAQKSTKKKMQPKKGKKNQKMTQINNEEGEEETNNNADNQIAQSSSCCSSEDDSNASQ
ELNGGTVSSNPKGKSRASRGAATDPQSLYARRRRERINERLRILQNLVPNGTKVDISTMLEEAV
TYVKFLQLQIKLLSSDELWMYAPLAYHGMDIGIYQKMLPNMQ

>SlbHLH084
MLNCLLQHTLRSVCTCSDSSSNASEWVYAVFWRIVPRNYPPPKWDHGGGLLDRAKGNKRNWI
LVWEDGFCDFYECERSKREHVTINFGPEIFFKMSHEVYSFGEGLVGKVAADNSHRWVSKDAPN
EKDSNFTCSWNMSIEAQPRAWGVQFNSGIQTIAIISVREGIIQLGSFNKAFEDHNLVLNIQRKFS
YLQSIPGIYAIQRPFLPIQHPYTYKPNNVTLVNETDNQMDDKNQIIGSKRVHEFPFKSINFGYNSP
QTMASLPLWSMPIAAPSCYANAAHEMSSLHDRVTRNTTSKDVKVVDELGHLKFETDEENDQF
SLNQNLGLENKVVEVGFRQLGNGGAAPNPN

>SlbHLH085
MADPYRTNPHASSSLESEDMSSFFLNFLQGTPASSSATAAAGFYNRSGPAPVAESSSSLNFSDPG
RFYAAEFKEGVENVFASAGLGECDGMNSANRREFLEDDKVDNFGFSSEECDGLDMPSDPTHP
RSSKRSRSAEVHNLSEKRRRSRINEKLKALQNLIPNSNKTDKASMLDEAIEYLKQLQLQVQILT
LRNGLSLYPGYVPGSLQSVQLPSGNEFDGRSFMLSANGGATLPVNREMPQTAFEISNQNPSGKP
TITSHNTENAVALETTIQNHYGLLNHLASSKDMCRDNTLSRLHLDMSCSGNNSSSGVSS

>SlbHLH086
MDIDMLKSSATSEDQMEMMLMMQLEKFPEFSTGNCSELPMMEFSPQGSCNSSNNFQQMDQN
SPNFLNMPSTISFTNSPPIHQNSPNFIPNSGGFNSNSMNRSNMAAMREMIFRIAAMQPINIDPESV
KPPKRRNVKISTDPQSVAARHRRERISERIRILQRLVPGGTKMDTASMLDEAIHYVKFLKNQVQ
SLERAGATRPANGTALTAAAAGLGFPVPMSLSGNYNLPVSTKNYHHQNIQQYADV

>SlbHLH087
MEPANLHQQYQYHQLQFQDQFPLIGISPNSSSSSNNSCYGGVSTTNTWTPCTTTNTTILNSHGS
GLINSYSSGDIINTTKYSSSSDHPLNLVNSMSSTTHQDMGFHQWANNNIKQENSLDNSYQRFTQ
MLKSPEGGGELSDMNAKLLLGTLSNTGLQLYHGDNNNLLYSSNSSSISTINRGRFSQIYPTINVS
NLNINHQANSCSSLDMNLQPLDLINSTRYGGSFSQTYGLTTNHFQHSSSESPVNSSTSISAFSNG
MPEAKRTSNTLETNKGPQNAPKKSRVDSRASCPPFKVRKEKLGDRIAALQQLVAPFGKTDTAS
VLMEAIGYIKFLQNQVETLSVPYMKSSRSKASRSLHGGGGEMNNEEMKRDLRSRGLCLVPLTC
LTYVTEGGGGVWPPPNFTGGT

>SlbHLH088

MEKALEWLRPLVDSKNWEYCVVWKFGDDPSRFIEWMGCCCSGANGVDVNVKRENGGKQTF
SSLCRDIQVQHPIRTKACEALAHFPHSISLYSGIQAEVVTSNEPKWINHAEISNSNLSHELKGTLV
LIPVAGGLVELYNSKMIYKDQKTINFIINRFKLGSEEANSSVAQKEDQVLDFFPYEKSNFCAPLL
QYATSFPSSAPHISQVSESSANPSIQGSSTGSIPSNELTLCHSPPDHLSRNVPLSQSTEGYFEHTEL
QCSGNLSRMEDTIFPWKQENYIVAGDMFSMGKKRQKGPYQSKNLVTERKRRNRIKDGLFTLR
ALVPNITKMDKVAILGDSIDYINELQEKVKLYKIELNKIEAEVTNNESTPEMVLSDMTEMSKVT
GQTNEKTQISVNTTDRTRMEVEVNQIGAREFLLKVSGSRKPGGFTQLMEAMNYLGLELVNVS
CTTSGGEIVSVYIVEANVDRFIDAQKLRSSLIELTS

>SlbHLH089
MEEQLNSLAITHLLQHSLRSLCIHENSQWVYAVFWRILPRNYPPPKWDNQGGAYDRSRGNRRN
WILVWEDGFCNFAASTAEINANECPGSSSNNNNNNNYGEYQHYQGLQPELFFKMSHEIYNYGE
GIIGKVAADHSHKWIYKEPNEQEINFLSAWHNSADSHPRTWEAQFRSGIKTIALIAVREGVIQLG
AVHKVIEDLSYVVLLRKKFSYIESIPGVLLPHPSSSAYPFKVDGYGASPDAWHFQTNLPTPTPTP
TELYEHFNQHQHMRITPSMSSLEALLSKLPSVIPADVAAGMTGGSIPTTYCHEYQQQPQYRPNV
EILGLEKVAKEEYEDEEEEKENNNNEEKTRNNNNSNERLDHNGGESSSSMSSYSQHHHNYHH
QHYGYHHDLNVSSSMPNNGY

>SlbHLH090
MDPQASMMNHAGGFQSPPFNLSEIWQFPINAGEGETPYSFPLSTAAAPQNVSDDVRNNDPMV
LDRRTNNYSGGGGGGAARKRNEDDESAKGVSTSGNGLTESASKRMKVTRSNENCEARGDGE
GNSVKSAEQPAKPAEPPKDYIHVRARRGQATDSHSLAERARREKISERMKVLQDIVPGCNKVIG
KALVLDEIINYIQSLQHQVEFLSMKLEAVNSKMPSIEGYPSKDFGQQPFDTNAMAFSSQATREY
TRGTSPDWLHMQLGGGFERTT

>SlbHLH091
MSSRRSRSRISDDQIADLVSKLQQLIPEIRNRRSDKVSASKVLQETCNYIRNLHREVDGLSERLS
QLLESTDSDSAQAAIIRSLLM

>SlbHLH092
MDDLALIFSHHNNGESSRKQLHKEKEDEKANSLACIISFNNNFENSLSIPKDEANNTISFSNINNI
IGAKFGERRSGEQALEHLLAERKRRKRISKLFVSLASLIPGLNKMDKASILEGAATLIRQLGERA
KEDDHHQSTIGMMTKNNLLPEVEIKSLEKELLITILLYKNQQKRNIDEILSVIQRLHLTIKTTNFM
PFGTTSMHITVIAQMNDEFCETTDFLAEKLRSLISKV

>SlbHLH093
MLPWSIPPVQPFMNPVHHHDQSFLLPPSPSAYGLFNRNTNTDQQHLRFISDSLVGQVVHHHHH
HNQPGSIAPFGLQAELQKMSAQEIMDAKALAASKSHSEAERRRRERINNHLAKLRSLLPNTTK
TDKASLLAEVIQHVKELKRQTSLISETSLVPTEIDELTVDNATSDEDGKFIIKASLCCEDRSDLLP
DLIKTLKALRLKTLKAEITTLGGRVRNVLFITGDDYYCNNNNNREVDTCISGDDEDTEMMQQQ
QQQQPQYCISSIQEALKAVMEKSSGDDSASTSVKRQRTNNINILS

>SlbHLH094
MDIEIKNDSEPEKRNDQEVSMNYQSPNVSSEWQLNGSNLTNSSMGMVDSFCPTTWDQPTTNS

SNLGFCDASVQMDLGPFRAGVDSTLGPNWTPSNAVLKGGMFLPPVPMMLPQSLAQFPADSGFI
ERAARFSCFSGGNFGDMMNPFSIPESSMNPYYRGLSSMQGPQEVLANNGLKSPQKLQHLSNVA
ESSKDVSLTHRDTQRSPLKNEKKSENVAKSQDEAKEVAGVSGNESDEAGCSGRQEETEGAGEE
SCGKNIGSKKRKRGGQDTEPDQMKGAQQPPSEIQKGEQNLNPIASKPGGKNGKQGSQFSDPTK
EEYIHVRARRGQATNSHSLAERIRREKISERMKYLQDLVPGCNKVTGKAVMLDEIINYVQSLQR
QVEFLSMKLATVNPRLDFDIDGLLAKDILQSRAGPSSSLAFPPDMTMAYSPLHPPQAGLLQSGL
PGYGFPSEGFRRAINPHLATTSCGPGDYKDPSSQAPNEWDNELHNIVQMGLNSSVPSSSQDLSG
SLPAGQMKAEP

>SlbHLH095
MKSGKGHHVEEEEDEYDFGSNRDATPSSNSTKDGKNSDKANAIRSKHSVTEQRRRSKINERFQ
MLRNLIPHTDQKRDTASFLFEVIQYVQYLQDTVQKYEGSYQPWSSEPTKLMPWRNTQWRAQS
FPANPQALNNGTDAGPTYSGRFDENLLTVTSSMQANQRNPLESHSGGDVKSMDQGKELARTAI
ATSMPLQASMPVPFQNDSAFSDSLPTPASDECPRTTNALNDQEFMVEGGTINFSNTYSQGLLNS
LTHALQATGLDLSQASISVQINLGKRANKERALGPSVAKDTENPPPAPGDQFLEFRDTNNGEEL
NQAQKRLKK

>SlbHLH096
MENMVEENNNYLETTILFQQDSYLDEPIMSSYYDSTSPEGSQSSKNIVSERIRRNKLKEKLFALR
ALVPKITKMDKASIVKDAIEYIVKLQKQDRRIRGEISKLESETSNKNSTHLQHETFDFSNPKTLD
EHQYGYHSSPIDVLQLRVSSMRDRIVVVNLTCIKRKDTMIKLCDVFESLNIKIITTNIIAYSETLL
NTTYIEADVEESNLLMLRIQSAIASLNNSDSPLSS

>SlbHLH097
MTMLWSDEDKTMVAAVLGTKAFDYLMSSLVSAECSLMAMGSDENLQNMLSDLVERPNASNF
SWNYAIFWQISRSKLGELVLGWGDGCCREAREGEESELTRILNIRLADEAQQRMRKRVLQKLH
MFFGGTDEDNYVSGLDKVTDTEMFFLASMYFSFPRGQGGPGKCFTAGKHVWLSDVMRSSVD
YCSRSFLMKSAGMQTVVLIPTDIGVMELGSVRTIPESLELVHSIKSCFSSFLAQVRAKQAAPLAA
VVAEKKNGNNSVFPSSFPFDQSKENPKIFGQNLESGSTEFREKLALRKPVDGPLEMYRNGNRA
PIINTQNGVRPVSWASFGNVKPGNSVDLYSPQAPPNNLREFVNGGREELRLNSLQHQKPGGMQ
IDFTNSRPVVSPVPTVESEHSDVEVSCKEKHAGPADERRPRKRGRKPANGREEPLNHVEAERQ
RREKLNQRFYALRAVVPNISKMDKASLLGDAIAHITDMQKRIRDAEYKLEKRGSTSVDAADINI
EAASDEVIVRARCPLGTHPVAKVVEAFKETQVSVVESKLAVGNDTVYHTFVVKSSGPEQLTKE
KLMAAFAGESNSL

>SlbHLH098
MEKEENCQANNTLPTSFQMAIIGESSSNNTNNQMVDYMVDSQSIQQQQHNQSSLGFLPSNPSL
DKLSFADVMQFADFGPKLALNQTKVLEQDVGIDDPVYFLKFPVLNEKKNDNDNDDDREEGL
MISGGKEKSENNNNNVEKNQEGKSNNKRKRPRIKTSEEVESQRMTHIAVERNRRKQMNEHLR
VLRSLMPGSYVQRGDQASIIGGAIEFVRELEQLLQCLESQKRRRIYGDTPTRPLGDSSTPPSMPM
NQNPSAINPHHHQSPILFPLPNEYNIEDEIQEEVAESKSCLADVEVKLLGFDAMIKILSRRRPGQL
IKAIAALEDMQLSILHTNITTIEQTVLYSFNVKISGETRYTADDIANSIQQIFSFIHAEIAPYDIN

>SlbHLH099

MNYCVVPDFKMDDDYYDIPSAIFNKKSTIADEEIMELVWQNGGVIMQSQNQRSVRKSNLFPE
QSAVEQTVAVSAPLYMQEDEMNSWLQSPLDDSSFDDFLNTTPSCDAVTSAAAAPPGEIGTSKVE
IRPPLVPPCSRPIRCTEGELPHRLQNFGHFSRLSGEAVLRNGTTSSSGHSVRASTIVDSNETPVAA
RVSENVTPVTAMNVRGRELTATSMATTSGGREVTMACELALTASTRGSGGSVSARAGPPQPSH
TEADTAAYDRKRKSRESDDNEGQSEDVEYEFADARKQVRSSTSAKKSRAAEVHNLSERKRRD
RINEKMKALQELIPCCNKSDKASMLDEAIEYLKSLQLQVQMMATGCSMVPMMYPGIPQYMPT
MGMNMSMGMDMKMGRNRPLISYPPLMPGPAMQNAAAAAQMAPQYPLPAYHLPPFPAPDPSR
IPVANQPNSHVGHNISQPRLPNFSDPYYQYFGLQQAQLMLPQNQEVEQLSSSKLNSCIEGSRGN
HQSGEHTI

>SlbHLH100
MGIFSHTPNKGKFDLSDSFQCDISRQRVTTYNVRHVKRCKSVVMKRRIRVERSRRFEDEIGSKV
KILKKLIPINCEDLGLEGIFRETADYILALEMRIKAMQDMVNVLSHSNH

>SlbHLH101
MDNFHSIFPLQPDDDANYDSHQQQLIFPHETYTNPCNFIYQQDLVNDFASFDSLELRDNNLTFN
NNTNNNQKGKSCGVEDKKKHKKVMHREIERQRRQEMSTLYASLRQQLPLENIKGKRSTSDHI
LEAANYIEQLQKNVKNLEEKREKLMKDSTGLSNVDGINKSGRSTSRHCSPPAIVTVKECLDGM
EILVNCGLGFRLSRVLQVLLQEGLSIVNCSSTKTNTSSLLHTIRTEVSSTDQPRIINVDVIQQKLTD
IIHDDTSFK

>SlbHLH102
MVSREQKRASVLHDKLQLLRSITKSHALSESSIIVDASKYIQELKHKVERLNQDITTTTPQTNSN
NNTSSWPQIEVETLEKGFLVNVYSERSCPGLLVSILQVLEDLGLNVLEARVSCTDTFRLQAFGG
EDEETMINAQVVTEAVFEAIKNWSESKEQG

>SlbHLH103
MLRSIQLREMMDDSSCSKYTHETLSSWCHNLQELPLIPTNSCCWSQQFSASRSHSEAEKRRRD
RINAQLSTLRKLIPTSEKMDKAGLLRSVVEHVKDLEGKAKEMSNVLNTPSDIDEVVIEEEDESS
NNNNIVVKVSFSCDDRPELFSELNRGLKNLKLTTMEAKITSLGGRIKCILSLQSINVVCTTHSIK
HSLRLLLARIATSPSTSNFRIKSKRQRFFLLAT

>SlbHLH104
MESGNASMENNNVNDIGLINFLDEDNFEQFIELIRGETADPIVNFCPNYDCEHMTGCFSAANAQ
FEPILSSMDFYDTTLPDPISLYNCEIKLDNNDDEDDDESSGTTATTKMTPTSKGTRTDRSRTLISE
RKRRGRMKEKLYALRSLVPNITKMDKASIIGDAILYVQGLQTKAKKLKVEIAEFESSSGIFQNA
KKMNFTTYYPAIKRITKMDINQVEEKGFYVRLICNKGRHIAASLFKALESLNGFNVQTSNLATS
TNDYIFTFTLYVRECHEVDINFGNLKLWIASAFLNQGFDFETSPLV

>SlbHLH105
MNFQEFGAAFDPIDILSSWNISQRQEAATRLAADSVAAKSGAGSIGDCSKRKIDSSSSNFPPGNN
YLPYFGNNQVPVCSLMADLSSISLFSKTKSPNCLLSATNTSNTDTSGELDDVIFSDDSESLWNIH
TSNVVSSGESAIDAYKSKPIDADDNNDIHYSVNQLHATVSCNNLIRSSEAKYYNMDKRSHDAL
LESDSSNRDYVIQLISENDQPKSKKSRSEYKLPSSSNINFQQASSSACSIVDRDSEAIAQMKEMIY

RAAAFRPVSSVTEDVMVEKPKRKNVRISMDPQTAAARRRREKISERIRILQKLVPGGSNMDTAS
MLDEAANYLKFLRTQVNAFESFGFNIDPSITNNNFTTSLSSIPLINYPFPLQPHFPMQNLNPVHHP
KC

>SlbHLH106
MENTTFNNNIIGDEEAPFEFKNLLSEMFTSLPNQNPTTSNSLENVPKIAFSCSSPSSNNSSSSSQNI
ISFGNKADSFFLEDNELDYDMITEKVIMSNVNISNSSMASKRICRSPLQSQDHLLAERKRRERFS
QLFALLAKAIPQLKKLDKASILEDAIKYIGELQERVSSLEEAARTIKSSTNLIESTHPTLVHKQYS
HDDHVDDLESKRHENINDIKVQILDKNVLIGIHCNKQMRSIFSIIAGIMEKLHLTIHHIRVSPSNH
TSLHYISILAEIDENVDIKVQDVEKAFELHLLTIQDSTQE

>SlbHLH107
MESNYYDSFQGMEYSGNYNYEDESFFEFKPNISQGIYDLSSAYNTNIEEKSGQKSISSNNSSSNS
GGFLISFSSNQEEDIGAMISSENSCQESFLLGENNNNNNNNNNVMYKRSPQQAQDHVIAERKR
REKMGDLFISLSKIVPGLKKLDKSSILGDTIEYMKELQEQVKLLEESKKNTSSSLEHNDSNKEQ
VLGSNKIKVRIMDKNVLINIHCNKQDGMLGRLLVQMEQLHLSVHDMRIMPFGPTNLEISLLAQ
MEDGCCINVEDIVKAIQINILDLVNN

>SlbHLH108
MQRGTTGDGGGGLSRFRSAPATWLEALLESDTESEVILNPSSPILHTPNKPPPHPSTPKLKLETG
GATRFTGDPGLFESGGSSNFLRQNSSPAEFLSHISSDGYFSNYGIPSSLDYLSPSVDVSQSAKRTR
DDDSESSPRKLVSQLKGESSGQLHGSGGSLDAEMENLMDDLVPCKVRAKRGCATHPRSIAERV
RRTRISDRIRKLQELVPNMDKQTNTADMLEEAVEYVKFLQRQIQELTEHQKKCTCSMKDQ

>SlbHLH109
MAGNPTNWWSMIMNGNMHPQLSHDHDQHNSSSSNSNSNSSHFYGAHNPNQDFPRSLSQLLM
NGFCGDEEKFGISPFHAAGNSEDQSLNSVHPTSSTANFRVPVADVKPELYHHHHGHNEFQTSSS
LQEKLELDTDHFTRPLIASNWSQQDLSSPASSCITTGLSHNLLNFSNNKGEHKHQHPDHNSTEC
NSTSSGGISKKARVQQSSAQPSLKVRKEKLGDRVTSLHQLVSPFGKTDTASVLSEAIGYIRFLQA
QIQALSSPYLGNASGSMGHIPQQSLNFLDGNFLKRRPANKQDSQHKPMDLRSRGLCLVPISCM
ANIGSDTGADYWAPALGGGF

>SlbHLH110
MLSRVNSMQVWDMEGKQEEEKEENFSNKENTTNVELENKQDMELGALSTFKSMLDGTDVD
WYHNNMQNHTENICFTQNFTELAENSMFLQPVVPVDSSSSCSPSSVSVFNNLDPSQVHYLLAQ
KAINNNPLDYSFNLGCENGFLEAQGMGGLNKGGFLLAGGGFHDLSSQNQMGNPNLNSFTQYP
SSHLPQNTTTTGFSPLGFVDGSANENSLFLNRSKLLKPLDNFASNGAQPTLFQKRAALRKNLAN
TTGGSLGDFGGEIGQNSMNGENERKRKWGSGEELDDVSFDGCTLSYDSDDLTENVTNKVDDT
VKNGGNSSNATSTVTCGNQKGKKKGLPAKNLMAERRRRKKLNDRLYMLRSVVPRISKMDRA
SILGDAIEYLKELLQKINDLHNELESTPPSSSLTQTTSFYPLTPTGPALPGRIKEELYPSSFASPLSSP
TGQPARVEVKAREGRAVNIHMFCSRRPGLLLSTMRALDNLGLDIQQAVISCFNGFALDIFRAEQ
CKEGQDFHPDQIKAVLLDSAGCHGMI

>SlbHLH111

MELPQSRPFGTEGRKTTHDFLSLYSPIEQDPRPSQGGHLQTHDFFQPLEQARKTVGKEENKVEV
EAIEKQPPSAAHILPGGIGTYSISYSQQRFPKPEANTFAVTQTSSTDRDDRNSNCSSYSGSGFALW
NESAMKKGKTGKENLAGDRHVVREAGLNIGGGKCTTSLERQSQLSSNHNHNTATLSSHSSPQ
QPSAMENQSFIHMITSAKNAQEDDDDDEEEFVVKKESPSPSRGNLSVKVDGKSSDQKPNTPRS
KHSATEQRRRSKINDRFLKLREIIPHSDQKRDKASFLLEVIEYIQFLQEKVHKYEGSYQSLDSQP
STLPWNKCHSMAQGFIDHSQGTNSASSPALIRAAKFDENKIGISATGPVTEQTQEPNRTSSVKES
SFLSELTNRAATLCMQPSTFPFGGHTSIASLKSILAPDAGSLELKSQPQCPSNRLNMANYAVTND
KLKGQEVSTESGTLSISSVYSQRLMNTLKQALQSSGVDLSQANMSVQIDLAKRADDRSNASTS
NFKGDNISSRNQPTPLFIDTSTREESVHAFKRLKTS

>SlbHLH112
MSHHTWNFSHQKQEQQVVEKEEEENRYTRGHVHNQQNQVDPMSNKCEVAELTWENGQVAM
HRLGSNLSNEQTKHTWGKAGDTLESIVHQATFQKQHHSYIMGSDGQNQANINREKNVSYGAQ
QTRGVLKRMRSSDSDPQLYIGGISLEHLNARASAKDNDITMITWPCNEDSACHGGSENKEEER
ETKSSNPSKRSRRAAVHNQSERRRRDRINEKMKALQKLVPNASKTNKASMLEEVIKYLKQLQA
QIQLISYAKNMEQQMMMMSLGMQPAHIQMPLLATMGMCSSTTGILNNMTSNLAPAPYQSLIG
GRAPLIYPTSSMPTLFPPFMSPPFATASSIPSTPPQPINAESISPKLTKYAAPPNIAASTSFPFSHPYN
AYLPHSMKMEFNNEMAAQYLQRGNQENVNIQGQKK

>SlbHLH113
MSSRRSRSSRHSGGSRISEDQINDLVNKLQQLLPELRNRSSDKVSASRVLQDTCNYIKSLHREV
DDLSDRLSELLESSDTTQAALIRSLLMQ

>SlbHLH114
MEANSNSFHVDSVFHVPIKMSGFFEEPNNNITSSSTLPNCVSQFYLQELSVNMSNNVHEISHNE
PSHVTNKTNSSSLCSTQSKNVRDGDDGKGQKKRNGNVKREKKTKENKKKAPEEAPTGYVHV
RARRGQATDSHSLAERVRREKISERMKILQALVPGCDKVTGKALMLDEIINYVQSLQNQVEFLS
MKLASLNPMYYDFGMDLDALMVKPDQSWSGLEGPLLENTTSNYPHLDSSTSLMFQQLHLPNS
VSQGSGHVLWSVDDQRQKMIINHSELISNNNNLSVPFH

>SlbHLH115
MGYLLKEVLKTLCGVNQWSYAVFWKIGCQNTKILIWEESYYETSTLSNIHGTSGVENPELAFQ
DWSTGWAFGGVQNSQLQNQAGENLHLLINKMMMDNQFNLVGEGLIGRAAVTGKHQWVLSE
GLSRNVHPPEVLRELRQQFSAGIQTISVIPVLPHGVVQFGSYLHIMENMGFVEDVKTLMSQLGC
VPGVLLSDENATKEPALETSRSVYLGSSVSTEYCGRAKVMNSASIIDKGNSIQTEGFVGQTSFSL
VDATFQDSNFTQTFADCHDNHLHKKISPQVKPCMYMNNQLTNSVIKTEVIPPNTDMWKKQQD
SQYIPKPPFCQESSVGSLPLDSDSIMLTEQQISGENSLAKSNLTLPNFLGSSHGRSHHAVMYKSIP
HPNFIADASRPPQKIISCTEHIGDGLQIGSSDLMASSKYDVNHVINNHSLDGQGAEYLLDGSKR
MVENDLFQALGPILTQNENPSSSECIQDFYSEKIEHGARFPLFDSAYGDVHVQCQSGDDLFDVL
GADFKKNHLNGSWNNGQCKEPNSNTKDWIKNSSTSTISQDASSTINQGNSDSCMFSMTGFDRI
LDTMVSSHSAKQSLDDNVSSRTTITNLSSSSAPNASCSYDRVGVSSQIQGEQFVSPKTLLKSGAI
SSSYKSECSKEDTGMYSQSSSIYGSTISSWVESGYDTKPSSSVSTGYSKKPDEMSKTSRKRLKP
GENPRPRPKDRQMIQDRVKELREIVPNGAKCSIDALFERTIKHMLFLQSVTKHADKLKQTGESK
IISKEGGLLLKDNLEGGATWAYEVGSQSMVCPIIVEDLNQPRQMLVEMLCEERGLFLEIADIIRG

LGLTILKGVMETRNDKIWAQFAVEANRDVTRMEIFISLVHLLEQTAKGGTEPVNAADNNTAMV
HSYHQAAAKPATGRSCSLL

>SlbHLH116
MDNDCFSNGGIQPPFHFDPKIPLNSLHSPHSDYFLNTHWDNNSTDNQYTHFDSALSSIVSSPVPS
NSVNSNSSLCELIGKLGSICTSPSTPFTSNCDSTRTSCYTTPMSSPPKLHIPIMNQIGKDKVPNLG
NSVVMNSPPFPSLSAAKFSCFGSRSFNGRTSQFELNNEDSRYGSGTGVMGIGNLTRISSSPCVVQ
NKNSSLMMCERLNLGKISGRNEECSVSEQDPNGEMGSKTRNVLNSKKRKAVKSKDFVPIVDE
TGKKRAKSTQGNGSNNGTVKMEEQKGNEDDGAEKETKENRKIAEPPKDYIHVRARRGQATD
SHSLAERVRREKISQRMKLLQDLVPGCNKVTGKALMLDEIINYVRSLQHQVEFLSMKLASVNP
RTDIHIDSLLHTEISQPSGSLHQHVFPVDGYAENLAQLPTICEDDLQSIVQMGFNQNSNQDLILQ
SQTFPVPNSESQMKIKM

>SlbHLH117
MAEKFFLKGEDKVNMEGVLGSEAVEFFSWSASNHMLTEFTSSRGDLGVQQALCKIVEGSDWT
YAIYWQVAKSKSGKSALIWGDGHCRETKIGQGEGANDSAHQKMMDGNKKKMVLQKIHTCFG
GSEDDNIAAKLESVSDVEVFYLTSMYYIFPFDKPSSPSQSFNSARSIWGSDLKGCLEHFQSRSYL
AKLARFETLVFVPLKSGVVELGSVKSIPEDQNLIQMVKTSVVVSNPPQPKANTKIFGRELSLGG
AKSGPISINFSPKVEEELSFASDSYEVQAALGSSQVYGNSSNGYRSDEGEGKLYKEELDERKPR
KRGRKPANGREEALNHVEAERQRREKLNQRFYALRAVVPNISKMDKASLLGDAIAYITDLQAR
IRVLDAEKEMVGDKQKQQVILEIDFHQRQDDAVVRVGCPLNAHPVSRVLKTFQEHQVVAQES
NVSLTENGELVHMFSIRAPGPAAEDLKEKLTAALSK

>SlbHLH118
MNSLLSQQQQSQISLQDLQNGGNGGSTGGVGGLSQHSMGHSHFDPTSSHDDFLEQILSSVPSSS
PWPDLSKSWDPHHHLSSPPHNPSSGEDQPPSNPFHSQFHYDDQASSLLASKLRQHQITSGGGA
AAAAKALMLQQQLLLSRTLAGNGLRSPNGASGDNGLLSLPLNLSNGDQNDGVANPTNDNSV
QALFNGFTGSLGQTSNQPQHFHHPQGGSMQSQSFGAPAMNQTPAASGSAGGGGGSTPAAQPK
QQRVRARRGQATDPHSIAERLRRERIAERLKALQELVPNANKTDKASMLDEIIDYVKFLQLQV
KVLSMSRLGGAPLVADMSSEGRGEGNVGRGGNGRASSSSNNETMTVTEHQVAKLMEEDMGS
AMQYLQGKGLCLMPISLATAISTSTTRISNNPLLAPEAGGSTSPTLSALTVQSATAGKDATSLSET

>SlbHLH119
MRNPNSLKQEFLKKWIKGLQICNATKKNMSIMERKKAIKLSADIAMASTRKSTIYWSHALMK
NALKDDTNKIIMKNILGSNDDNNNNNNNNLKKTSMGINSMSQNGIIRSKKIIKKSCKISRRARKII
SPNIIAKKMVKKRTKVLKSLVPGGEYMDDASLIKETLDYIISLRVQVDVMRHLANNASYEINDP
KTRL

>SlbHLH120
MDNIGDEYKNYWETTMFLQSEELDSYFDEPISSYYDSSSPDGSQSSMASKNIVSERNRRKKLN
ERLFALRAVVPNISKMDKASIIKDAIDYIEELHNQERRIRGEISELESGRSSSKKNSNDVEFEQDE
SFDSKPKRSRRFEMQYGYDSSGSTTRSPPSSSPVDVLELRVSSMGEKTVVVSLTCSKRTDTMVK
VCEVFESLNIKIISANITAFSGRLLKTAFIEADEEERDLLKLRIETAIASLNDPDSPMSS

>SlbHLH121
MNRGDVMEKSPVQQIMGGSPKWWNMMNNMRPPISSSQQAAVAATTTHHVPNSLLPPNILFPH
FSSSLVPMSSTTNSWNDSNQSQLPESWSQLLLGGLVEEEDKSVHMVKKLENNWDEQSFLSQH
DSVIDVNQKDLRNSYNMYEDGNNVEFHHQTAELAAKPTWSAQMIPVSSPKSCVTTLSSNMLD
FSNKNTHPTPDHSSECNSRAPNKKARVQPSSTQSTFKVRKEKLGDRITALHQLVSPFGKTDTAS
VLLEAIGYVRFLQSQIEALSLPYLGSGSGNMRRQQSVHERNNLFPEDPGQLSNDNSLKRKANS
EQDYQEDKKKDLRSRGLCLVPLSCTLQVGSDNGADYWAPAFGGGFR

>SlbHLH122
MDSIFFLEEGDRTVFLLKIMESFGCTYICLWQYFQPSNTFMSLGGIYNGENVVAQRLFEEYKHS
WLIMDNGRIPGLAFKNNVPYMELKFADLQSHASNPVQLQFYYTTICMGCSIGEIEFGMTSSPQV
NLEMGMKNLFPEYFSTRLVLARPQTLLTNIDQNRPSPSSSFSLDSPGEYSSLLFNVATTSYVPDA
FPEQTVRPVSTSAMPFHQQQPIQTLTQLRGIQFPGVETDDAALTRAYLAVMTSPSSSSSSHQSRE
NIDVPITDYHYQKSTAFRRFGPGLGRPSNVQIGTSRTIRRENILRRSIIFFRNLDMMRRKEQIQAN
QRALTSTQVHHMISERKRREKLNDSFQLLRSLLPPGTKKDKASVLASTTEYITCLKDQVEELSK
KNEIMLNAQALDKSSMMKSNDVGDGNDERVVVEIIKNVSSESESRTVELQVSVRSGECNVLDL
ATRLLEFLKTQDNLSLQSVAANTRPSMVTHVSLTITIQGSEWDESGFEEAVKRVVDDLT

>SlbHLH123
MGFDHELVELLWRNGEVVLHSQTHKKQPGYDPNECRQFNKHDQPTIRVAGNQTNLIQDDETV
AWLNCPIDDSFDKEFCSPFLSDISTNPHLGEEPDKSIRQSEDNNKVFKFDPLEINHVLPQSHHSGF
DPNPMPPPRFHNFGSAQQKHHIVGGDQKGVNFPPPIRSSNVQLGGKEARSNLMLQDIKEGSVM
TVGSSHCGSNQVDTSRFSSSANRGLSAAMITDYTGKISPQSDTMDRDTFEPANTSSSSGRSGSSY
ARACNQSTATNSQGHKRKSRDGEEPECQSKADELESAGGNKSAQKSGTARRSRAAEVHNLSE
RRRRDRINEKMKALQELLPHSTKTDKASMLDEAIEYLKSLQMQLQMMWMGSGMASMMFPG
VQHYISRMGMGMGPPSVPSMHNAMHLARLPLVDPAIPLTQAAPNNQAAAMCQNSMLNQVNY
QRHLQNPNFPDQYASYMGFHPLQGASQPINIFGLGSHTAQQTQQLPHPTNSNAPAT

>SlbHLH124
MEVDSSGNPNWLFDYELMTDITSAASVTVAEFQSPATIDFSWPAQTIYASSNLITETDYTFADSE
VSKEASSRKRLKSECCSSPRSKACREKLRRDRLNERFLALSSVLDPGRPPKTEKVAILSDAQRM
LIELRTETQKLKESNEELQEKIKELKAEKNELRDEKQRLKEEKDNLEQQVKSLASKAGFLSHPS
AMGAAFTAQGQVAAGNKLMPFIGYPSVAMWQFMQPAVVDTSQDHVLRPPVA

>SlbHLH125
MGKEKLVNEFLIFILDVNGLSRYSVLVNDNGKVLQRYPYLPNQEELVSDEVLSMLRAGTYISTV
GSKSNQRISLVFFCVFCVRAMERKLPLVQPEFNAGEALLLPLMESDEAFINGMYNGLSYQSLLS
LNLDHSYYGEVKPFSANSSNFRPSFTNDMLCAGTTASSQIQQSSGTFHEFSTGDFAWLDKKTEI
NPDADQKLKYPKLEPVTDNLQLYPYNNEVLLCEPFNFFSNGVGYPSLPDLRCMEQPNYLDFSS
SLVTTENRVVEPVRPVSEVMSNLLKNRQPYSSSTTRLRRQKLSEKIRCLEKLLPWDKKMDTST
MLEEAYKYVKFLQAQIDVLQSMPPLVEGGASSDQDRNGKSTSYGLNHEAKAISVFGTLARLN
RQQLLQVLLNSPVAQTYLYSKGCCVYSVEQLVQYRTIAQRNAFYRRSLFLSGMLS

>SlbHLH126

MEFLSSFNGLNEVYGGFQGIIGNGLSSSSSLVLDNESGELVKAMVKPGGKGVNPEKALIALKN
HSEAERRRRERINGHLGTLRNLIPGTNKMDKAALLAKVIGHIKELRVNAAEATKGVLVPTDIDE
VKVEQQAEGSDGATYSVKASLCCDYKHELISDLRQALDTLPLKTLRAEIATLGSRMVSVFVITE
GNEGNTEGTERCQLLITSVRQALRSVLDKFYASEEFSSRSTLSSKRRRVSLLNSSSSSSLGDFW

>SlbHLH127
MESSCNREESSATFSRKDKNVCKRQKRRRESGYIVKNNEEEEEEVEEKILALQKIVPGGETLGV
DRLFEETAGYILQLQCQLKALKVLANFVEGNDKQRMKLGG

>SlbHLH128
MDDSEFDFGQLDQLFNFLSSPSPPPPPPTTLQSYQDSSFSLQKQNSNIVFTSTHNVPKKKPILITNI
NIIDDQVIQDLPKEKEMVVEKKVMRRDVERQRRRDMAKLYQRLRLLIPSKYLMGKRSISDHLE
EIVDYVKDLKKDIEELESKREKLKEMKNITNISSPLAPNSSSMKLNDDDDEDKIIVKSCNEGVEI
SIKGVLSISKVLKVLMKEGFIVNSCVSSTINQRLIHIIQTKVNKRGDIDLALLRSKLMGKKSYI

>SlbHLH129
MVGSGTADRSKEAVGMMALHEALRSVCLNTDWTYSVFWTIRPRPRVRGGNGCKVGDDNGSL
MLMWEDGFCRGRGTDCLEEMDGEDLVRKAFSKMSIQLYNYGEGLMGKVASDKCHKWVFKE
PTECEPNISNYWQSSFDALPPEWTDQFESGIQTIAVIQAGHGLLQLGSCKIIPEDLHFVLRMRHT
FESLGYQSGFYLSQLFSSTRTSSPSSAIPLKQPTMPIRAPPPLFNWGPRPMPSASSLLSSPNFQNSA
RLGIPQSKDESHMFLQLPHSSEPRMEDMMGAAADHESDIKWPNGLTFFSALTGRNDDSRILFNP
DSLGSKPDHNQHPLSLDGKTSNPNSDASSLHNNGGANPNDFLSLDSHPDSIRKMDKFKRSYTL
PARMASSSNSSTSLDQHANNPGEYRNEGGMYPDVMERFLE

>SlbHLH130
MAEEFQLGRGNWWESSTSASSTTTTSSSRNKFIDSGISSCTTPSASSTTGLSSMASNFVNWPIEI
HEDIKVRSENSSMVFSGTDSHKRYASGGGGGGVGQGVLSVDDPNLQIMGLGLSSQGLDWNQP
FFRSEKSGSGFRSLIQEGLSSNANYQQEGTCQEQDHNNWSTQKLYHGNSDDSSVNDYNKQLFS
GHNNNLENSAVPYGSPSNMLQGLLISDLNSQQQESNNFSSVSRSLYYNPSYNNQPNCDVNIPTS
TSSSSWSKFPQFLRTSDPSKVVQQWSLSQSPPLSHSQLSHFSGGTSFWNATSAAAEDVRSGFLP
QLPTNPTVDEKPKHTGEVRNTSTVTKKNSSETSNKRPRNEAPSPLPAFKVRKEKMGDRITALQ
QLVSPFGKTDTASVLSEAIEYIKFLHDQVNVLSTPYMKSGASIQHQQNTGDKSNVNPEGGKQD
LRSRGLCLVPVSSTFPVTHETTVDFWTPTFGGTFR

>SlbHLH131
MVSPESTNWLYDYGFEDSCVPDSNFSASASGFNWSVQNLNGSRNVSSEIDGSIGESDYPKESGS
KKRARVESCAPTSSKACREKLRRDRLNDKFMELGALLEPGRPPKTDKSAILVDAVRLVTQLRD
EAQKLKDSNLNLQEKIKELKVEKTELRDEKHRLKAEKEKLEQQLKTTSAQPSYLPPAIPSAFAA
HGQFPGSKLVPIMSYPGVAMWQFMPPAAVDTSQDHVLRPPVA

>SlbHLH132
MTDYRLWSNTNTTNTCDDTMMMDSFLSSDPSSFWPASTPNRPTPVNGVGETMPFFNQESLQQ
RLQALIDGARESWAYAIFWQSSVVDFASQTVLGWGDGYYKGEEDKNKRRGSSSSAANFVAEQ
EHRKKVLRELNSLISGVQASAGNGTDDAVDEEVTDTEWFFLISMTQSFVNGNGLPGLAMYSSS

PIWVTGTEKLAASQCERARQAQGFGLQTIVCIPSPESREILNFGDSSKRFSGQSQLGPGPGLMEE
NKNKNKNKKRSLGSRGNNEEGMLSFVSGVILPTSTMGKSGDSDHSDLEASVVKEAVVEPEKK
PRKRGRKPANGREEPLNHVEAERQRREKLNQRFYELRSQIECLRKELTNKGSSNYSASPPLNQD
VKIVDMDIDVKVIGWDAMIRIQCSKKNHPAARLMAALKDLDLDVHHASVSVVNDLMIQQAT
VKMGSRLYAQEQLRIALTSKIAESR

>SlbHLH133
MENLNISTSSTPSQPNTLQKTLQYIIHNRQEWWVYAIFWQASKDVNNRLILSWGDGHFRGTKD
TTGSTKTGHGQYHQFQKKFGFNDISETNNNVTDTEWFYMVSMPQCFVADDDLVIRAYTSASH
VWLASYYELQIYNCERAKEANLHGIRTIVCISTTSGVVELGSSDVIQENWEFVQFIRSLFGSNNN
MNTTSHLPVNQVTLGDDHKVAKCGSNIIVKQEMTIGNLLSESGISDFENDDSLTINNVMNGSIK
RAKKGDSSHIRREMAMDVHVEAERKRREKLNHRFYALRSVVPYVSKMDKASLLGDAVTYINE
LKAKIKNLESKLIEPQKKHILMEQHDSHSASSTIVTDHGANNKSLFSSNGVRNGMEIEVKIIGSE
GVIRVQSLDMNYPCTRLMNAMKEMKFQIYHASISSVKDLMLQDIVIRVPEEFSNEETLKSAIISK
LSVMEN

>SlbHLH134
MQHKRSPISLEHSSSLTSLTPKRLKADMLISSKEKKEKFGERIVALQQLVSPYGKTDTASVLLEA
MGYIKFLHEQVKVLSAPYLGTMPMSKTQESQPYNLRSQGLCLVPVSYTVGVASSNGADIWAPI
KTSQKF

>SlbHLH135
MERLRPIMSLKGWDYCVLWKLSEDQRFLEWICCCCGGAEKNMHGCGQEIFFPDSSTSTCRDV
MFQHPTTTACNLLAQVPPSLALDCGVYAQTLLSNQAKWMNFVPFSESNISNEIMGTRALIPSPL
GLLELFSTQQLAEDEKVIEFVSAQCNIYLEQQAMMNSTFSNGVEENNTSKPFPTEGERDRDDHI
KDSQNHYKQRVSPAATSDHLSFDFPLKRKQLDSCSMNFLPPFSTYSTPEVDNNTGGNMLFDQS
TSDMTHFSENRYMSEMDAYLQKQMMRSSSTQAGIDDESIKHDNGRSNSGSDSDQNEEEDDPK
YRRRNGKGPQSKNLMAERKRRKKLNERLYALRALVPKISKLDRASILGDAIEYVMELEKQVK
DLQLEVEEHSDDDGTGGGRNSDQIHPVVLSHNGTKNRPKSDNGKLTNGSQREISTNSNGSTDP
SRKNQDVEENDKLQQMEPQVEVAQLDGNEFFVKVFREHKAGGFVRTLEALNSLGLEVTNVNA
TRHTCLVSSIFKVEQKRDNEMVQADHVRDTLLELTRNPSRGWSEMGRASSDNINNNNANGTT
DYHQHQLHDHHLDNNNQHKQTNSHHFHTHHHH

>SlbHLH136
MMTMQQLLFDFNYDVEQNFSDENQDCYFDPDEFILPIEMNNSCCFMPEYSVLEKQPKDNHSC
FIPEYSVFENIPKRQKIFQDDFFPNPNSNTITPSTHNSCFMPEYSVFEKQQKLFQDNFHEEGFLPN
PPMFEDFALPEIPVPVFSAGVVAKKGGSSNNEKKMSAQSMAARQRRKKISDKTQELGKLIPGG
HRMNTAEMLQATYKYIKLLQAQAGILAFIGSYQENEKSFETSYLQKLVGSSLVQEKLYSSESSIQ
IYHISIRPGPYPHQHRALNFTKTLTQTESPFHLPWAQKSSINPILFSLLQYQEMGEVVKGAVPVPE
SVLKKQKRSEEWALAKTQELVAAKKKSAENRKLIYNRAKQYAKEYDQQEKELIRLKREARLK
GGFYVDPEAKLLFITRIRGINAMPPQTKKILQLLRLRQIFNGVFLKVNKATVNMLHRVEPYVTY
GYPNLKSIRELIYKRGYGKVDKQRIALTDNAVIEQVLGKYGIICMEDLVHEIMTVGPHFKQANN
FLWPFQLKAPLGGLKKKRNHYVEGGDAGNRENFINELIRRMN

>SlbHLH137
MMTMQQLLFDFNYDVEQNFSDENQDCYFDPDEFILPIEMNNSCCFMPEYSVLEKQPKDNHSC
FIPEYSVFENIPKRQKIFQDDFFPNPNSNTITPSTHNSCFMPEYSVFEKQQKLFQDNFHEEGFLPN
PPMFEDFALPEIPVPVFSAGVVAKKGGSSNNEKKMSAQSMAARQRRKKISDKTQELGKLIPGG
HRMNTAEMLQATYKYIKLLQAQAGILAFIGSYQESFETPNLQKLVGSSLVQEKLYSSEHCLVPK
VFVEALENNQEFQNSQILEEIKTLMKEGK

>SlbHLH138
MALEALSTNELLNFIIYDTISTTTTTTNSNNNLDENALFFDNHEENNAFLLKPQDFCTTTPLEQQ
YSIAASVPRPESSREKNNLSVAAQSGGGGGGRKKRRRRPKICKNKEEAENQRMTHIAVERNRR
KQMNEHLSVLRSLMPESYVQRGDQASIVGGAIEFVKELEHILQSLEAQKFVLLQQQQEGGTSN
DNDDCDGGKREVSKADYVGTPFAQFFSYPQYTCCELPNKYTSKSKAAIADIEVTLIETHANVRI
LSRRRFRQLSKLVAAFQSLYISVLHLNVTTLDPLVLYSISVKVEEGCQLNSADDIAGAVHHMLRI
IEEEAATL

>SlbHLH139
MTEYSLPTMNLWNNSTSDDNVSMMEAFMSSDLSFWATNNSTSAAVVGVNSNLPHASSNTPSV
FAPSSSTSASTLSAAATVDASKSMPFFNQETLQQRLQALIDGARETWTYAIFWQSSVVDFSSPSV
LGWGDGYYKGEEDKAKRKLSVSSPAYIAEQEHRKKVLRELNSLISGAPPGTDDAVDEEVTDTE
WFFLISMTQSFVNGSGLPGQALYSSSPIWVAGTEKLAASHCERVRQAQGFGLQTIVCIPSANGV
VELGSTELIVQSSDLMNKVRVLFNFSNDLGSGSWAVQPESDPSALWLTDPSSSGMEVRESLNTV
QTNSVPSSNSNKQIAYGNENNHPSGNGQSCYNQQQQKNPPQQQTQGFFTRELNFSEFGFDGSS
NRNGNSSVSCKPESGEILNFGDSTKKSASSANVNLFTGQSQFGAGEENNNKNKKRSATSRGSN
EEGMLSFVSGTVLPSSGMKSGGGGGEDSEHSDLEASVVKEADSSRVVEPEKRPRKRGRKPANG
REEPLNHVEAERQRREKLNQRFYALRAVVPNVSKMDKASLLGDAISYINELKSKLQNTESDKE
DLKSQIEDLKKESRRPGPPPPPNQDLKMSSHTGGKIVDVDIDVKIIGWDAMIRIQCNKKNHPAA
RLMAALMELDLDVHHASVSVVNDLMIQQATVKMGSRHYTEEQLRVALTSKIAETH

>SlbHLH140
MAMGHQDQDGVPGNLRKQLALAVRGIQWSYAIFWSTAVTQPGVLKWIDGYYNGDIKTRKTV
QAGEVNEDQLGLHRTEQLKELYSSLLTSESEEDLQPQAKRPSASLSPEDLTDTEWYFLVCMSFV
FNVGQGLPGKTLATNETVWLCNAHQAESKVFSRSLLAKSASIQTVVCFPYLGGVIELGVTELV
TEDPNLIQQIKNSFLEVDYSVILKRPNYVSNDAKNDTNIGSQKPDHNALENDAYPVEINSPHDS
SNGFVANQEAEDSLMVVDGIGETSQAQSWRFMDDNISNGANNSLNSSDCISQNNANCEKLSPL
SSGEKETKPCPLDRQENDQKKPHLLDHQGDDAQYQAVLSTLLKSSDQLTLGPHFRNMNKKSSF
ASWKTDIQMPRFGTAQKLLKKVLLEVPRMHAGVIHKFSRENGKKNSLWRPEVDDIDRNRVISE
RRRREKINERFMHLASMLPTSSKVDKISLLDETIEYMKELERRVQELEARSARRSNDTAEQTSD
NCGTSKFNDIRGSLPNKRKACDMDEIEPESSNGLLKCSSADSIVINMIDKEVSIKMSCLWSESLL
LKIMEALTDLHMDCHTVQSSNLDGILSIAIESKSTGSKTLAVGTIREALQRVVWKS

>SlbHLH141
MCDSMAKETLKRLCRSHGWSYGVFWGFDQTNSLLLALQDVYYEEQMGSVIDEMLLQVHILG
RGIIGQTAFSKKYKWMFTAANHERQISIRSSNNSNLFLDDNEFEQQFSAGIKTIAVLSVEPLGVL
QFGSTNKLQESTCFVEQARTLFQGIGGSPTSSSCENLHFVNSTVFPTANNESLMKESHFLENLIQ

SVTCNAESQIMNSDVATAFLSENQFQDVNQFNNCSSQFDTQLQQAMFPSAGLFTSFHDSCLTST
WEDLPSDMSIQDFSYVLPTGINQFEYGTGATQSFHDNTTFGSLGGFGVLANEDTTGPLNGYIVQ
CPINQRNDGAVSTISDNILDTTGIISASAGINEQFRFNSDSDASVSIQSSITNAFETVEKANCSNMS
AIEKMTNLVGVKHDSKKPCNWGDVSNPVVSTSNSEWTYSNANELRSRPANRLFSKLGLDQFL
DGALSSSYSFAGSFSDGQLSETNKRRRVGSSSECNYLQKPLGFSNFDKNAKLVQPECGLDRTSN
LEAKSEIITKLDASTLIGDRCSINNCRGNEKSSKPTKKKAKPGTRPIPKDRQLIYERLSELRGLIP
NGEKMSIDRLLHRTVKHLLFLQGVTKHAEGLKKAESLKDSETRLNSKSNGNGVTWACEIGDQ
TMVCPLIVEDLSTPGQMLIEILYNEQGFFLEMVDIIRGFGLNILKGVMQSRETKMWAHFVVEAE
GNRLVTRHEIFSSLVQLLHLTSASKVGLNNQLQYTSGGRNTLINDCPNSAVPISGCLPETIRCVR

>SlbHLH142
MQDFISTSSSFSSTSLLQKRLHYIIHNRQEWWVYGIFWQASKDANGRLIFSWGDGHFRDLALA
KVHNANVSDMEMFYAVSAPNCFLSEDDLIVHAYNSGSYVWLNNYYELQIYNYDRAKEAHLH
GIRTLLCISTPHGVVELGSSQVIQENLELVQLIKSLFGQINDHGFNFVPLGDPMDTKTITMGSDS
GNSDESSAMNKDSPKKRARKSTTAKNHVEAERQRREKLNHRFYALRSVVPNVSKMDKASLL
ADAVTYINELKAKVEELKAKIEVSTKKLIQKRNCVSSSAVVDGTNINININSSFVDGMEVEVKII
GVEAMIRVRSPNVNYPCARLMNVLRELEFQIHHATVSSMKEMMQQDVVIRVPHNVTNEEAIK
SVILTKLSFA

>SlbHLH143
MNSKEKKERVYSSAPKKVMKLSTDPQSIAARERRHRISDRFKILQSLVPGGSKMDTVTMLEEAI
HYVKFLKTQIWLHQTMVNLVDINHEMVGYYPLVDDDQNIHKNNISSMDYQQMQQVQSYDN
DAFQQVEFPFEETNISGDVFMYYN

>SlbHLH144
MSAASLRHFLESLCFKSPWNYAVFWKLQHQCPIILTWEDGYLDVPGAREPYRSQNGNYYSKNL
SDLSPNCGSRSHNGYLSAHSIGLAVAEMSSTYHIAGKGVVGEVASLGIPRWISSDSVAPAELGFG
SVAECPDKWMLQFVAGIKTILLVPCIPYGVLQLGSVETVAENMEMVTILAEEFDAHLKFVESFL
PGGESCEFLLQSTLSETLNIPSATTTNKVNEDDVAADIPIVEDHKSSAVFPMTSLIDVQHPFQLSG
QHMQNVLENENESKIGKFVEHMPNVLENAYKWEIPMQHVDMINLVKQLAHGYSDDNRSGITE
RSIVRSSCHTKDIDAFSYSSCNVGGVGVSNEVDFHFDGDMLDPRSLGMDCHNTILGNVSNSFS
CSTERELHEAFGSTIHNLSGFSANPSSKSIYAADCTFNSEPSDGWHLKEDNAENLLEAVVASAYC
FTDDYSLNKMAGLESLNMSSGKPVPSRKRLNQSAESDSVGDAVTRSTLTSASAGVDKYASTNR
PHSASSFDYVVSTFDEGHHQTKVFSSLDCHKESKISNTNKKRRRSGDSHKPRPRDRQLIQDRLK
ELRQLVPSGAKCSIDGLLDKTIKHMLFLRSVTDQADKLRFQAQTEVAPDKNLQSPPIKSSNQQG
TSWALELGSVDQICPIIVKDLEYPGHMLIEMMCDDHGRFLEISDVIHRLELTILKGVMEKRSEST
WAHFIVEASGSFHRLDIFWPLMQLLQQVPSSVSRNI

>SlbHLH145
MNGGGESNHVFSWEIDDVLSYLNLNDNQIGSGTTFEGDMMTNPIRFDTYQSLTVVNEVVESST
NVAKKRSPPNLKKNGKGIVEPKSSVDGVRDKGSLEHEIHISTERKRTKKIWILFETLRALIPNISA
KADECTIIDKAVNHILKLKNTFEKLKRENLEGFQEHNIRLISSQKFPDVGNSWEKLFG

>SlbHLH146

MKKKKINGGGENNHVFPWETNDGWPYLNLNGNQIGIAVTFEGDKLQDPTRFDTYPPLTVVNE
VIEPSTNAGKKRSPPNRKKNGKEIVEPNFCIDGAGDRGGLNHDLHIWMARQRTMKIGIFFNTLR
ALISNIPAKADKSTIVEKAVNHIQKLQNTLEKLEQEKLERLQEHNVRCRSSQKFTNTCNNWEKY
LGDQGSTHNTSSTTSTTHGTNPLMVNNNIPTGFVTWSSPNVILNVCGENAHISVCYPKKSGLFT
FICYILGKHGIEIVSAQASSDQFRSVFKIQAHAKGGTGIAQYSEASRVEEMYKQIAIEIISFATPK

>SlbHLH147
MGSKEQKKALLNQQLEQLGSITNSPSVNKTSIIAHASKYIEELKERIDKLNEDVSTSQPYHHDH
EDALPEVIVETLEKGFIINVFSEKNCPGLLVSILEVFEELGLELLDARVSCSDCFRLEAVSDEGEIE
CIDAQMVKQAVLEAIRNWKQLNHQD

>SlbHLH148
MEKDYFINGGIPNPALQFEPTMSFPSWNPLHSGQSLFNPNWDHSTPQFDSTLNSIVSSSPAELIG
KLGTVCSSPQPILHNNSYSRPMMGKDNIPNLGSSLPPPLPADPGFAQRAAKFSCFGSRSFNGRTS
PLGLNYTELSHRSAQTLGNGKLPRVSSSPSLKQGGSPLQLKNSGQTRMEMMSNNSNESVSEPS
GETASKLPTELNSSSRKRKTVSRGKTKEDSPTEGVNGNRGSEADDNARAKRCKQVESNGIENG
RVHMEESKDDETQKQVMEYQKPTEPPKDYIHVRARRGQATDSHSLAERVRREKISERMKLLQ
DLVPGCNKVTGKALMLDEIINYVQSLQRQVEFLSMKLATVNPNLDFPLSKDICQPNGSVAHPVF
PIDKTSSSYQQGRSDIPNGALSQCSVDTSDNSLCRSLGMQLPPLDGFAEYLNQFPEDDLQSFVQ
MGFTQNPNKDMTLQSQGPHQTSHMKIEM

>SlbHLH149
MNHSVPDFDMDDDFSLPASSGITRTKKSAMAEEEIMELLWQNGQVVMQSQNQRSLKKPHIGN
GSGGGGDAVIPSDQAVSREIRHVEETTPHHLFMQEDEMASWLHYPLDDPSFERDLYSDLLYPTP
TSTFTTAALPRENRTSTFEIRPPPPQPSPAAPIGTAPRPPIPPSRRTVTENSNRFQNFGHFSRLPKAR
LEPGQANLSKSPRDSTVVDSNVTPITGQESRVTLIPDNVVAVPGGNVGCSTVNGSTGTATASTAI
REPTTTCDISMTSSPGGSGNSVSASAEPPAPAPAPSHKGAAPTATAAADDRKRKGREMEDEGQN
EDAEFESPDTKKQARGSTSTKRSRAAEVHNLSERRRRDRINEKMKALQELIPRCNKTDKASML
DEAIEYLKSLQLQVQMMSMGCGMVPMMYPGMQPYMPPMGMGMGMGMGMDIGMNRPMV
PYPPLLPGTAMQNAAAAAQMGPRFSIPQFHLPPVPVPDPSRMQASSQPDPMLNSLVSHNSNQP
RLPNFSDPYQQFFGLQQAQVALPQNQAVEQPSNSKSGSSKEVGNPGNHQSG

>SlbHLH150
MGEQNQPNFVGTQWAFLGSTNDSFYLAGHEFNNQYMTEVGIDGIHYVPVPSPQWIHDQQTPH
NYVEYLAENVVSEVDASVEATGFSLLEGSADIISQEIRYPRPYYEGVPPNCLNSSEHQRGALTW
ELRGNVRHASGPCSDASAAGSSYSSKSSRKARAALSDRHRRMKIAERIDALGELFSCSKQGGK
ASQMDEIIDHIKYLQFQMKDLSRSRLGGEPTSIPFVFLEGCGHYILDEQQIEPLEDTMGKLLEVN
PSLATQLLESKSLFVMPLALAEGLHHHE

>SlbHLH151
MEIIQPNSLQLQNMLQNSVQSVKWTYSIFWQFCPKQGVLVWRDGYYNGAIKTRKTVQPMEVT
AEEASLHRSQQLRELYDSLSAGDSNPPARRPSAALSPEDLTESEWFYLMCVSFSFPPPIGLPGKA
YSKKHHIWIMGANDVDSKVFCRAILAKTVVCIPLLDGVVELGTIEKVQEDIGFIHRVKSFFNEP
QQAQPPKPALSEHSTSDPAAFSEPHFYFSNTPSSAGICPADQDGRITGEEENEDEDEDEAEDDED

ENDEAELDSDGIAIQSGAGAANPMAAEASELMQLDMSEAIRLGSPDDGSNNMDTDLYLDGISQ
AGNTADSFKAETAISWANFQDLQHLPGIPSYDELSQEDTHYSQTVSAVLEHLSNTSSKFASSATI
MGSISPDSAQSAFTLWPVTCSPNLSHCRRHDIGDGSGTTSQWLLKSILFTVPFLHSTKKLSEALS
PKSRDAAAADSSAAASRFRKGCTINSCTQQEETSGNHVLAERRRREKLNERFIILRSLVPFVTK
MDKASILGDTIEYVKQLRKKVQDLEARDRHTEITKKSDEKSGSPIVKAFPVKGKRRMKSTVEG
SIVGAPAKMTGSPPMEEEVLQVEVSIIENDALVELRCPYKEGLLLDVMQVLRELKVEVVAIQSS
LSTGLLLAELRAKVKENIYGRKASILEVKKSINQIIPRVN

>SlbHLH152
MANNPSEGPSDDFFDQILGFPAYNGAEPNLAGNDAGAIPPAMMLQLNSGDGSSQFTGVGLGVG
LGGGGFHGHGGGGSFPLGLSLEQGKGGFLKMDDVSAPGRRFRDDVVDSRASSSVKPGFHGQP
MPSMPHPPAIRPRVRARRGQATDPHSIAERLRRERIAERIRALQELVPSVNKTDRAVMLDEIVDY
IKFLRLQVKVLSMSRLGGAGAVAPLVTDIPISSVEEESSEGGNNNQPAWEKWSSDGTERQVAKL
MEENVGAAMQFLQSKALCIMPISLASAIYHSQPPDTSSLVKPETNPPS

>SlbHLH153
MASQLQQALRSLCCNTPWKYAVFWKLTHRARMMLTWEDAYYDNDGFPGKKSPDSTAGNLYD
GHYSNNHLGVAVAKMSYHVYSLGEGIVGQVAITGKHLWLSANKVAAITNLAPEHCDGWQAQF
SAGIKTIVVAAVAPHGVVQLGSLDSIPEDLRAIKHIRDVFSELQELMTSCLRSSMQHSMENSCLS
EISTRTSGSEIFQDCVNNLGRSVCEDRRNMWSPLYTSFEKSVDHSCIFLQPGGYPNKILEVVNNQ
RLHRSSVQGSDDSTNLFCAGYELYEALGPVFQKGNSSKDWEAGKREEMAVDMLEGIGTSSLV
MSNTGNEHLLEAVIANVNRHDNDCSSVKSFCKSVDSLLTTEITAEPCSSDIGTISSTGYSFDRETL
NSFNSSGTCSIRSSRGLSSTSCSRGSGHVERPLEPVKMHKKRARPGESCRPRPRDRQLIQDRIKE
LRDLVPNGSKCSIDSLLERTIKHMLFMQSVTKHADKLSKCSASKLADKESGICGSSSHEVGSSW
AVEVGNNQKVCPMRVENLGMNGQMLVEIFEDGSHFLDIAEAIRSLGLTILKGLAEAYGERTRM
CFVVEGQNDRTLHRMDVLWSLMQLLQAKINL

>SlbHLH154
MDNNYLWEIFTDNKQNISEDHVDHNHHLLQIQIPTFQLNGFSQQDNQDLIEDHVGSSIITSQFQT
SCDQPYKSSTVSTNTSIIMPQFQRSSDYLCKSLISTKSTSIITPQSHTSYDRLYKSSVNRSSTSIIMP
GQLQTSCCDRLYKSFTKEGLRSQDESNKLKKAVHRNIERHRRQEMANLVTSLRSLIPIEFIKGKR
SASDHMHAAVNYIKYLQKNIQELDNTRKCIISLDNQNKSSTIVDIFQQNNNCVTVNKCEDGMEI
LINVNNSNKEDIFSLSKVLRWLLKQGLNVVSCDNSKKDERTLIRIQCQVSDISSGLTVVGLQKK
LTDVIN

>SlbHLH155
MAANQPEGYADDFLEQILAIPPYSGLPVADVGTPSETTSFTSASAVSHLNSAAAAGLQQPLFPL
GLSLDNGRDDVGDAGPYAVKHERDGMNIGNLYAGLEHLQSHAVRHSVPSVHHVQPFQGPPTT
STTVTVPHPPSIRPRVRARRGQATDPHSIAERLRRERISERIKALQELVPSCNKTDRAAMLDEILD
YVKFLRLQVKVLSMSRLGGASAVAQLVADIPLQSVEGDSGESRSNQHIWDKWSNVDTEREVA
KLMEEDVGAAMQYLQSKSLCIMPISLAALIYPTQQPDDQSLVKPEAAAPS

>SlbHLH156
MEEFFQVSWFCDDENETNNNFVVNQSAFVSFGSKSNEGFGVSSYGNVSMNHRNMNKRMIEFL

KKNWSPKNGQVKIEKEKVHKHMIKERIRREKQKQSYLNLYKLLPMGTKNEKNAIVQTATRRIE
ELQKYKENLEKRNDEIQLILAQSDKKEEEFEKAKIKAKVGNPICGVDSMLEVLKCLRNCETKA
NSIQSSFSHQEFSTLIEIETKSGAAEIEKAVQNTLFEVERNLRAH

>SlbHLH157
MDRFDSFRDANWDLIDFNSFIDEESPIDFFWNDQTQDLSAVAEVEAPLSSAALQECIETECPRKR
GRNESCSKQGNKACRERLRREKLNERFSDLCSVLEPGRPVKTDKMAILGDAIRVLNQLKTESE
EYKEMNQKLMEEIKTLKEEKNELREEKLALRADKERMEQELKATASPASFIPPHPAAYQPAVNK
MAVFPSYGYVPMWQYLQPSSRDTSQDHELRPPAA

>SlbHLH158
MVNIDEEKALMVSQKEEEINVTFVGENSKGNESKKKRKRTKIRMKSSEEVENQRMTHIEVERN
RRKQMNEHLHVLRSLMPSSYVQRGDQASIVGGAIEFVRELEQLLQCLESQKRRKLYGDNHQD
SSLLMEIQNPSNNLVYAPNNNEIGIIQEDIAEIKSCLADVEVKIIGIDNAMIKVLSKRRPGQLINTI
SALQDLQLNIIHTNVTTIEQTVLYTFNVKICGETRFSADDIANLVQQIFSFFSCK

>SlbHLH159
MEKGNLFINDDNTTYEHLQNCFFNPNLDNNNSDPFESALSSMVSSPISIPNNNSGSDNFVLRELI
GRLGSICNNTNNNSSSTNNSCYSTPLNSPPKLNLSMRGNLPPTQFTTDPGFAERAARFSCFATNL
ESNHSIKIQDVNLVQRNSEFGDSRENSSLSEQMIGQNDTNSRKRKSISKGKSSKIVNDKNESNA
KRSKSEENENKVTKKEENAVLEENKDNQKATEPPKDYIHVRARRGQATDAHSLAERVRREKIS
ERMKLLQDLVPGCNKVTGKAVMLDEIINYVQSLQRQVEFLSMKLATVNPRMDFNMEALLSKD
MFQSRGSLGHNMYQSETSTQAFPYGFQSQPNQNYHKGTEFPFQINSLNPNLIRNSSMQLPPLDG
FVEPTPQVPTFFEDDLNSVVQMGFGQNQNQSFPGVAGNVPNSQMKVEL

>SlbHLH160
MYPSSTSSSSQGSMSHTSTTAGGAGGGLTRYGSAPGSFLTTAVEAVVNGNHEFASHGSHHSHLG
PSRFFQSNLASTSLNSESTSKAKEQSNLQRSIGFNDLTIGGGSGAGGGVLPTTSTTPLVRHSSSPA
RFLNQLATAAGDTVSMGRGSYNPKGGGDSGRGITRLNSQLSFTTQEALSQIAEENEDIEGTSIA
NGHRKSTHSYASASSFAMGSWEDNNSIMFSVTPSKRSKQISNDIVNGLDDGETQFQFGLSQTAL
EIASMDRLLHIPEDSVPCKIRAKRGCATHPRSIAERERRTRISGKLKKLQDLVPNMDKQTSYAD
MLDLAVQHIRTLQDQVQNLNTELENCKCGCRKSSQ

>SlbHLH161
MVLPENTNWLYDYHYEDIVTPDVNFSVSGYSWSMQGFNGSTNASVDIDGSLGESDCVKESGC
KKRTRAESCTSSSSKACREKLRREKLNEKFMELAVLLEPSRPPKTDKSAILVDAVRVVTQLRGE
AQKLKDSNLDLQEKIKELKVEKNELRDEKQRLKSEKEKLEQQLKTMSAQPSFLPPAMPGAFAS
PVQAAGTKLVPIISYPGVAMWQFMPPAAIDTSQDHVLRPPVA

>SlbHLH162
MAEEFQLGGGNWWENNTSTSSRNRFDSSGSSITPTTSTSATTTDYSSNWPIQTNHVDIKPRTFL
DSVSVSQSSDHDDKGEGGGGGGGGVLSSHDSNFQIMGLGLTSQPHDWNHQSLLNYEDSSSQM
NIRGFSLDQNNSIFSHSSNYEVTDNHQVLHSSTTNSWSKFPQFLRASPSPEQPPLPPPARPLPPPH
SQLHFSNNTPFWNASAASMNDVPSSLFPSNLHNIPNNTTIDEKAKNMGDVRDINRIISKKTTTIE

TSNKRPRNETSTPSPIIKVRKEKMGDRITALQQLVSPFGKTDTASVLSEAIEYIKFLHDQIGALSA
PYMKSGASMQHLQSDNKSEDIGEGRNKDLRSRGLCLVPISSTFPVTHETNVDLWTPTFGSTFR

>SlbHLH163
MESYNLKSKLCPKIKRKNVEKNRRNYMKNLYNQLHSMLPTTKEAMSVSNQIDAAVNYIENLK
INLEMNKKHLEELKMGLKKAQSFNPTNEPGPTIKSTPHIEFHEMGPNMVVVLITRLNNIITSLDY
AMRKVLKLCLQAFNSMESTLHCIFLMKLRWI

>SlbHLH164
MEGCQRSSSAPAGVKLERKDVEKNRRNYMKNLCNQLYSLIPSTHHSRETMGLQDKIDAAIKYI
KSSEMKLEKKKIHLEKLSRLSGRKRPKSSNSTNGPSPSTGLSSSPQIQFHEMGPNMVVVLISGLD
NIATFNNIIRLCHDEGVEVVYANFTLNGNSMLQISHETKINMSSTMECRAANLCDKLKELLHGK
SYDNELESQLYLWDYIVESELLKFYDVEFLPSTSQNSNMYN

>SlbHLH165
MERRMKSKLPPAGPKIERKYVEKNRRNYMKKLYNQLHSLIPTSKETIMTVPDQIDAAVNYIENL
KMNLEMNNKYLEELKMGLKKAQSFNPTNEPGPITKSQPQIEFHEMGPNMVVVLITSLNNIATF
NNIIRLCYVEGVEVVSTRFELNGNSTLQISHETTKINRSSAMELIGTNLCDKMKELIYGPSYMES
QLHLWDYKIEFDTLEYYLLPTRSKNPNMYSYMQN

>SlbHLH166
MKSNLKSKLGPKLERKDVEKNRRNYMKNLCNQLYSLIPFPNASTSSSKETMAVPDQIDAAINYI
ESLKMNLEKNKKSTPQIEFHEMGPNIVVVLIIGLDNIATFNNIIRLCYMEGVEVVSTRFELNGNS
TIQISHETKVLFLISLGYICYVWT

>SlbHLH167
MERHLKSKLAPKLERKYVEKNRRNHMKNLCNQLHSMLPTHSSTSKETTMTVPDQIDAAVKHI
ETLKMNLEKNKKHLEELKMGPNKAQSLNQTNEPGPITKSPPQIEFHEMGPNMVVVLINSLDNI
ATFNNIIRLCHKEGVEVVSTSFKLNGNSTLQISHEPKVQINKSSPMEFKATSLCDKMKELIYGPS
CNNDIESNQHLWDYIIESGLIEFNTIELPPIENHMKNIYETPSFF

>SlbHLH168
MDEPIFSPSSSQSSLQHRLQYIVKNQTNYCSDWAYIIFWQSSNNRSCLTWGDGHLNMKITNNKD
VEWFYLMSLAQSFCVGEGVVGKCFSSGSLVWLAGDQQFEFCHCERAKEAHYVHGINTFVCIPI
SSGVLELGSSTMIKQDLNLVQQVKSMFFGYETIDQFDDFGLFNCLELYGEEAKKGEVVVGTTP
HENKAGLKNKTSKKRRREICETQGNHVEAERQRREKLNSRFYALREVVPNVTKMDKATLLSD
AVTYITQLKAKVDELESKLHSNNYHYYYPEMKIKHKMENHDINVVDNQSSITTSRDHTMEIEV
KMVGQDAMIRVQSENVNYPSTRLMCALQEVELHVYHANISSVNDFMLHDIVVKVPQGLETED
EVKYALLRSLDQQTCS

>SlbHLH169
MDELMVSSSSSSSSFFIPSLLSQTSSNLQQKLQNILKIQTDSWSYAIFWQTTNDDDDGHLFLAW
GDGHFHGTKSKTGVQSSEQSTERKNVIKGIQALICENGDEKVDDDDDDEVTDAEWFYVMSLA
QSFSIGDGVPGKAFSTASIIWLTGSQNLQFHTCKRAKEAHLHGIQTFVCIPTSNGVIEMGSNQLI

KENWVLIQQVKSIFNNSIPHIVNCLEQNTNINPKTEELVSVSVSAECNDSDSDCQLLVEKKTPKK
RGRKPGATRETPLNHVEAERQRREKLNHRFYALRSVVPHVTKMDKASLLSDAVSYINELKSKV
AELETQLTRKSKKLKIECTDSFSIDNNSTATTITNSVDQIRHNSFGVHSNLKVEVEVKILGPDAM
VRVQSENVNYPSTRLMRALQDLELHVHHASISSVNDIMLQDIVVKVPIGLSTEDRLKNALIRSI
QHQ

>SlbHLH170
MDYEVAELKWEKGEVVMHGLGPPGVPCYYKPLSTPSPTKYTWDDKPHAAAGGTLESIVNQA
TTHNIDIGDEGGDDDDLVSWFDDCLPETSMDIVAVVPTSCTNYNQQVPPSTRVASCSGDAEMA
RVGMGSSFEEISEDFENQEAKNLIGSMVYEGKNNTVSPGETSLGEERVLTTTSTFKHNKRKTLN
NHDSRGQESRDNEDEDEKKRSKISSFSTKRCRVAATHNQSERKRRDKINQRLKTLQKLVPTSSK
TDTASMLDEVIEYLKQLRAQVKAMSMMIHVNMQPPPMMLPNMAFQQQQQQFQMSMMGMA
RPIDVNALSSPNITTIPSILHTTAPSNFNNPPIASPGADPLASLVAVRQLSQPMTMDAYSRMAALY
QQYLQSNANLGFKN

>SlbHLH171
MEHVVSMSEGDWSSFSGMCFTEEADFMAQLLGNCSFPNELPSNSGYWNIGHESNIGSSGGRE
HSSFFFPPLSHESHYSSNSRPILMRNDSSITTERGLMDTNNPIEADEYFANNMEFDANMAEPLLD
GKGLQLGRIDYEDHSPTESSKKRVRLHCHVPKNKRSTKLQTEGKTVEMDMKSKAVLQRQNSM
VSCCSEDESNVSLELRRKSRASRGSATDPQSMYARKRRERINERLKTLQSLIPNGTKVDISTMLE
EAVQYVKFLQLQIKLLSSDDLWMYAPIAYNGMDLGLDLRIGNPK

>SlbHLH172
MEPVFEDEWSGMCSTDQEADFMAQLLWQPNNIDNMYFSSYNGCNSSQISFPSLESYYQSHVQ
SILTRNGSSITKENGMVEGENTSSPSQVLATYNPIEADDFLNQDVSMESGENTAKVLDSPPESSK
KKRLCNLLGDVPKNKRSVNLKKASEMDGKNKAALQRQNSISCCSEDESINVPKSRASRGSATD
SQSLYARKRREKINQRLRILQSLVPNGTKVDISTMLEEAVQYVKFLQLQIKLLSSDDLWMYAPIA
YNGMDIGLDLKIGTPKS

>SlbHLH173
MQQQQEDFQENLESCNYFSKNKSSSSDNNMMGEYYGFDIEAIKNFCSSSPFYPQHNHVEFQET
IESNNNSPESRAKNHKEAERRRRERINSHLHSLRTLLSCNSKLDKASLLAKVVQRVRELKEQTS
QIMQSETNLLFPSETDEITVLSSNDCLADGRLLIKASLCCEDRFDLIPDLIETLKSLGLSPIRAEM
VTLGGRIHNVIVLAVDHKKESNNNNTDHDESSLLFLRDALRSIVQRSSYGTGERGKRRRVLGQ
GTRNY

>SlbHLH174
MDSSEFEKNQNNSGLLRFRSAPSSFLENFIDGVGNNGNMGSENKGLSSKFNLDGLNNQLVSQN
SLDSKVFANLSSNSQLPPQYPRQNSTVHMVGSMEGGGGYRVMGSVLGNSNNHHQGQNKLAS
NLMRQNSSPAGLFSQLNSLNGYATLKGGAGGYRMATGANGDPNPSSSRLKDHFGFSSVTPSSL
GMLSRISEVDDESSIATDLDDDEKRGNGNSEPQLYNMGFPFNSWSDHSQFQQTLTGHKRELDS
EGNKLFANAQIEELGNRPPILSHHLSLPKTPADIAAMEKLLHFQDTIPCKIRAKRGCATHPRSIAE
RVRRTRISERMRKLQELVPNMDKQTNTADMLDLAVEYIKGLQKQYKVLTDCRANCKCSAMQ
KPD

>SlbHLH175
MDNLIECIEAGEARSENCGARMGEIKDGFHQFGEEILSVTSEGGSSFTALLGLPPNQAVELLVQS
PETDKIASDKLAISEPHYRYPPPPPIFPSDIALIDRASKFSVFAAAGNSPESNSTLSNSGSKSLFVK
QEPLDSECNHNSSPATSNPLVHQKSTKRKEREKKVKETSKKGKKSANDTSEDGGEKLPYVHVR
ARRGQATDSHSLAERARREKINARMKLLQELVPGCNKISGTAMVLDEIINHVQSLQRQVEFLS
MRLAAVNPRVDFNLESLFAAERSGSHVESNLQDMVVPPIWAEGQSSGNRNQYQHLWLIEGFH
QPAWGRLEDNSSFVTPENSLLTYDSSANSASLHPNQLKMEL

>SlbHLH176
MLAISSPMISTNYINFGWLLEDSNSQQININPMEPLHSSSRKNLQHSDSNKFDEITINGGDHHQP
DQTVKKLNHNASERDRRKKINDLYSSLRSLLPPSDHMKKPSIPSTISKILKYIPELQSEVERLVQK
KEEFTSTYVFNRQKLGDFTKQKRTKGGIENSSFVISTSKLSDKEIVVQISTLKINKGSIGEAISQLE
DEGLVLLNANSFETFEDRVFYTLHFQVEGSMVVEIDMLRDKLSSYFEKEDKL

>SlbHLH177
MLAISSSSPPLFSTTTNNFGWLLEDLISHELTNSGETSNSSQKSLQHCDSNKFDQIIINSGDQYQP
DQTVKKLNHNASERDRRKKINSLYSSLRSLLPPSDHTKKLSIPSTVSRILKYIPELQSEVERLVQK
KEEFTSKNILNKPKRIKGGIENSSFVISTSELGDKEIVIQISTLKINKGSISEAISQLEDEGLVLLNA
TSFETFEDRVFYTLHFQFEKLPEMMKDFTVGLALIEQLPNIAPFLLESAAYRIMLLIVNKDDDHY
DLDMDCFFNNGIVDNLVECCRSRNWRSLRRQAAETLRELVNCGTMAQNQELLRRGVVTIFLQ
MLNDEEAIDLDDEEWFDDLLVVEAVKTLALLSTKVDVRGDLVTAFVEQNLCRRLMLLFRNTR
HTIVENVLTFVENFLTIGTEEQMQVLLDNQVLHHLRLVIVEPVVSDLYLLGGQYKVVEILGNFA
RRSRRLRDLVIDSGEDLISLFRVLRSLADDSIVAFDASPAISAARTIGYLCFGSPPPPFDKLRPSLPI
LRFLINQLEPHIGERACPVIKHACLIIACLARGGFDPINALIDENMCPILVMLLAHPHSEVVASVL
KVVENFLKNGTDNQIQVLHDSQVLQHVLEIVMNHDNLPPLHLRSVCRAIANIVNYRSSQIQRM
VDAGIFPSIIQISINQEVGDTKYEAIYAISSVVTRGSHEQIRVSAVF

>SlbHLH178
MEKKLNHNASERNRRKKMNFLYSTLRSLLPPPTNKHQKKKLSFPATVSYVQEYIPELKKEIERL
SKTKDLLLSKKSNYSLLKIDDNNKRKLIIGGTSCNSSTTSICASQLSNSQVLVQISTTQENNFPIS
QVFASVEEDGLILLNASSFKSFGDKIFHSLHFQMQGPIEMDIQVLKTKLLVMCEKRRKNSYIV

>SlbHLH179
MDINHINKLTTSTWDPTMSNMDNQQVFRDQQQQQQPCLSSIPNDHIYHEHHHHQQQFHFEHN
PIWPSFPLQNPQHHHLPSSSTQQQQQQQEEVVVVPFDHVLNNHVQTLIEDQEHDDQDEDEEEE
EELGAMKEMMFKIASMQPVDIDPSTIRKPKRRNVRISNDPQSVAARLRRERISEKIRILQRLVPG
GTKMDTASMLDEAIRYVKFLKRQIRQLQSSNHNLPPAQIPVSSCPNNENWANNIVTPSTKGLIL
GSSSSTTTNNVTTFVGNTTLDPPYEVIGN

>SlbHLH180
MALETVVFQQDPFNYSHKDCNFYNLETFHDYGNFGYEGYNWNSSIPQSYNDDDNNNNINNN
NSNSSPDKYFPVESTVVSGRRKRRRTKCAKNEEEIHNQRMTHIAVERNRRRQMNDYLAVLRSL
MPPSYAQRGDQASIVGGAINFVKELEQLLQFLEAHKQVITTNQQHIQYSSFSKFFTFPQYSTGN

NNHPLAATTSNEGSEERRSAVADIEVTMVESHANVKVLSRRRPKQLLKIVNWLQAMCLTILHL
SVTTADHMVLYTFSVKVEENCELNTVSEIASAVHEMVAMIKEEAMPC

>SlbHLH181
MDHSRVEQQPITSGLTRYRSAPSSYFSTLLTTTTPVTGGGCGYARDDFSNLPNSTASSSSTDIEQV
FARFVASIGNSNSNSGNFPTNQPIHENPTSNMNVRSDFVVPKQEITPRLVRHRSSDHSLVSNYQN
QNQHFISPVKREPETLLQSTDHVSASQLNYQSQPQQIEKLNAVTDKPFVFANSASLTPGSSSGGG
GNSNLTRYNSSPAGFFAQINIENEYGAIRGIGRYETGTNAAADTSLSTPKSFKTQVGFSSGKPPAS
PRLMAPISEFGTKVLEEKSMGNEHKNDEHCITDFPMPSWEDSHILSDDFLKTEDIEIEPFSNEDA
SHNQSSEGLARPPIPLSHHLSLPTSTMEKLLQDSVHLNVRAKRGCATHPRSIAERVRRTRISDRM
RKLQELVPNMDKQTNTADMLDFAVDYIKELEKQVKILAERRAKCTCINK

>SlbHLH182
MGYLLKEVLKTLCGVNQWSYAVYWKIGCQNTKLLIWEESYYEPSTYSGIHGVPMVENPELPF
HDWGVCWGPGEVRNPQLMNQAGERVQLLVNKMMMESQINIVGEGLVGRAAVTGSHQWFHS
EGLSRVVHPQEVLKELTGQFSAGIQTIAVVPVLPHGVLQFGSCLHIMENMSFVEDVRILISQLGC
VPGVLLSDETAMKDPMVNTGTPVYMGSSVLVDSSVSTNVMNSAPVIASCSYQGNSSQNDGFIR
QTSSSLDAQVQDRIMQSIDSSFQASNMTQRFVESHDDRQFHKKIIPEVKSHLSPNSQLINSVIKA
EVISPSSNLWMSQQAPLHIPRPPFHQQSFTDSLTVDSSSLSNVSQLNGFTASDPRPNDVLISSYHG
NSISPSNGENELCKRRDGHHRSIPCPNSIADANGLSNIISSCTKSTGNGLQTTSKFNVGDDSSTSH
ISDGQNAQFMWDESNGIVENDLFQALGIMLTQNEHPCSTSKSVQEVCVEKHEYVGQSALLEN
NKYEDSCVQRHSGDDLFDVLGADFKNKLFNGSRNSYQSNGPDSNTWDRVKSNSTSVLSQQAS
SIVNQGKSDSGSFFAAGFERLLDSISSKPSAKQNMDDDVSCRTTLTNLSNSSAPNVSSSYGRAGF
SSQIQGNVFGKPKTLAKPGSTVSRSFRSEKEKTGAYSQSSSIYGSQISSWVEQGHDTKPTSSVST
GYSKKPDETSKTGRKRLKPGENPRPRPKDRQMIQDRVKELREIVPNGAKCSIDALLERTIKHML
FLQSVTKHADKLKQTGESKIISKEGGLLLKDNFEGGATWAYEVGSQSMVCPIIVEDLNQPRQML
VEMLCEERGLFLEIADIIRGLGLTILKGVMETRNDKIWAKFAVEANRDVTRMEIFISLVRLLEQT
SKGAEESAKAIDNNTAMVHSYHQAASIPATGSRPCN

>SlbHLH183
MLIDQNFHSSSSSSSEYSSLFLNGAKTTHENQTLTRFPKIEGEDDVIRQAYLAVISSSSPSSSSRGH
IQENLTRDHRLVTRNMTAFTRFRSPNNYASIGATRSCSQNMLKRSITFFKNLYTINRQEGTQVNR
AMSTQVHHMISERRRREKLNENFQHLRSLLPPETKKDKASVLASTTEYLSSLKDQMEKLYKRN
EILEAQLLIKKENSQFQQNESGRIDVYITNIEEKIVDLQVIAKGKYNTLDLVICLMEFLKVASYV
NLMAIDANTTMVHSCPLTRITLRLRTQGDEWDESTFLEATKKVIGDVT

>SlbHLH184
MQAMNSFQSTGENGASSGEHMSHSHFDPSSSHDDFLQQILSSVPSSSPWPEISGDGHPYNFDDH
QSTLLASKLRQHQINGGTSAAAAAKALMLQQQLLLSRGIAGNGGSGINGDQNDDGLNSGNDI
SVQALYNGFAGSLGQTSNQSQHFHHSQAQSFGAPAASLSMNQTPAASGSAGGAQPKQQKVRA
RRGQATDPHSIAERLRRERIAERMKSLQELVPNANKTDKASMLDEIIDYVRFLQLQVKVLSMS
RLGGAAAVAPLVADRSSEGGGDCVQGNVGRGGSNGTTSSANNDSSMTMTEHQVAKLMEEDM
GSAMQYLQGKGLCLMPISLATAISTSTCHSMKPNNPLLLAGGSAINGVGETGGGPSSPTLSAST
VQSATMGNGGT

>SlbHLH185
MMIKKMYSGVQGCNSASRLEKKYVEKKRRSNMKNLFNHLCSLLPPHPSQLQEAMGLPDQID
ASITYIKNLEIKLEKSKMQLEKLRTKKRSNLLCMANHDTNPSISKLSSPQIEVQEMSPTMDLILIS
GLDNLVMFYNIIRLFHEENFEVINSNFSLDGNSMMQIFHESKVIGDSTMVYRRVKELLNGSSSN
DDIIETLLHSWDNEIQSEMLGLINLM

>SlbHLH186
MIRGDENNHDFPWETNDFWSYLNLNDNHVGSGETFDGDKLPDLTRSDTCQPLIVVKEVVQTTI
GVGKKRSPPNRKRNGKEIAESNSDADRAETKRKSEHEIHIWMERERRKKMQTLFETLHSLVPN
LPAKAKGGSDTTQFSEEFTVEERFMQAAIQIMPLTTPK

>SlbHLH187
MAHHYFNPDMQIIIPPSSVFNTDTNLNFHNQFPEQNYLNTISLEMSNFNFHKHSDEHILNQTSDF
PVYLTENFQQDMFNLPVPTRNDINESKKRKTIETPESSSAYSSPAVSSRRKTGKGRGKRVKRDE
NEEEKLRQVVHVRAKRGQATDSHSLAERVRRGKINERLRCLQDIVPGCYKSMGMAVMLDEIIN
YVQSLQNQVEFLSMKLSAASTYYDFNSETDILETMQRAKAYEANMMQKLKKEGCEGMGSNQ
VGPLIDRTFGCYPKLSYNT

>SlbHLH188
MTTTRSCIIRSRFAYRFLHSLRKMNQQDKTNSRRVKHAAYASMASVVGSKRAWSRAVLSKIRN
RSLLLKKKKKRRRRSSDEFGELRKIVPGGQLMDFYNLLDETADYINSLTSQVHVMKNILNLLST

>SlbHLH189
MLHEEKENKDERMIHIAMKRNQRKQMNNYLTLLQSLISSSHVQRADQASIIGGAINFVKELEH
NLQTLRSNCSSELSRGSCQPQILTKRRPKQLMNIVVGLQCLWLTILHLNVTTIHNQIVLCSISAKL
EEGCQLTTIEEISHAVNQLLGRIQEEEATSSSN

>SlbHLH190
MERDFVSWFRRQYPDLQSAHSNFSSRGLNIGQQNSVPIYMPPYANEVPVKGSSPLSFSGLLEPK
ASQPTEAHNWFYCLPQLQQGFAPVLSTVPNEKFAPQSVDNYGVNEEANAGPGFAQKRFLVFD
QSGDQTTLLYSSPNGTPVQCLPSWHPKSAAPCHLIKEGQQILGNGICPSGKYSGGEYYEENHRD
DVESELHEDTEELNALLYSDDDDSYSEDGEEMSTGHSPSTMTAHDLPSWHDEMGEEVNSSEW
PSKRRKQLDGGCDIPPSLVDTATSAKPFTCSDLEDDAESSCGNSHNNQVSELVSLSGKKRPRKD
QILETISILQKIIPGGKGKDSMDVIDEAICYLRSLKVKAKSLGLDTL

>SlbHLH191
MEDYYRENEFLLDELFSLRSDLWESTCLPMEINSSSSNLFCNNINYNCFGEIPLPSTTTTTTTTS
FEDYYNNFPINQSQNYSLLHNEFYTTQKVDVLSPLELTNSFNSQLDDFSSIYFDVGNFGDCKLE
RTQSTASSAAIAPANFNIGACLDKKSKKKKVNGEPSKNLMAERRRRKRLNGRLSMLRSIVPKIS
KMDRTSILGDTIDYTKELLEKINNLQQEMELGSNQLSLMSIFKNEKPIEMFVRNSPKFNVERRN
NIDTRVEINCANKSSLLLSTLTTLDALGLEPQQCVISCFNDFAMQASCCEEMEQRGVTDAEEIK
QALFKNAGYVGKCL

>SlbHLH192
MEQMGASFDEEYCQSLSKMFLNENSSDFMFQLHGEDNIIEGSFFSSSNSHTSNNNIDYFSQENSI
DSRGSDAMFFLNNNTSHEYLQYNYDVESTNFYMTGNKMNLENSLSNDDYVMKENIGNNYSQ
LDKEMLLKRKFDKVEVQSTTQEETHKEFENPKKKSKVSRDHGQKNKKNSQPKAKKNIQMNN
EEVGDKETNNNGQSTSSCSSEDDSNLKTKTRASRGAATDPQSLYARKRRERINERLRILQGLVP
NGTKVDLSTMLEEAVHYVKFLQTQIKLLSSDDMWMYAPIAYNGMGIDL

>SlbHLH193
MVAMREMIFRIAAMQPIQIDPSSVKAPKRRNVKISSDPQSVAARHRRERISEKIRILQRLVPGGT
KMDTASMLDEAIHYMKFLKKQVQSLEKVEINRPMSMSLSGNYLPNYNYPYHQSVQP

>SlbHLH194
MEKKNLFLNNVNTMNELNCTSTSFYNPNWENSSMDYQNDNILSSNSNNFGEISPNSFVGSNNN
SCYTTPLNSPPRLNLSNFDHQIKGNFPNTSNNLPHFSTNLGNFSCFGGNDSQFVQNLESCKLSR
NKSMKESEFGDSRENSSISQQIQLEEVGIKCQNDANSKKGKSIPKRKAKEITPKNDNVSTQNNE
SSSKRVKSDEKNEENQKPQDSLKDYIHVRARRGQATDAHSLAERVRREKIGERMKFLQDLVPG
CNKVTGKAVMLDEIINYVQSLQCQVEFLSMKLSNLNSTTDFNAESLTSKNMFQSVGSLHHNM
NSSESSVQEFPYGFQSQQGSNIQSFLTKETEFPFKINPHLDGFVEQTPQVPTFFEDHDLHSFIHMG
FSQIQAQNYPGNVSTAQMKAEL

>SlbHLH195
MEEKSMDHRSHQQQQMTSGLTRYRSAPSSYFTNLLSSNNPAGVSGGSGNCGYARDDFDQLLN
PHASNSGIKQVFDRFVANIGPQDSNPDGLIDDTQQNQNPMSNMDVRSEVLAPMKQEHEAQMN
YQCQEQQSQNSQFVAPVKQEIAQQNSDYSLASQMNYQNHNATDFTSGMDNFGRYNRLNHTK
MDGGFGTGSSDSNLTRYNSSPAGFFALVNIENEYGALRGIGSYGAGSGTNAAGEVSFSNPSRFS
SQTALPSGQPTSSGLLAPISEFGAKSIEESRRGHESFGKGHKSDESYMAGFALPSWDDSQILTDD
FLQVPEDDESGSFSNVNASDNQSSEGRARPPTLLSHLSLPQTSAELSAMEKLLQDSVPCKVRAK
RGCATHPRSIAERVRRTRISERMRKLQELVPNMDKQTNTADMLDFAADYIKELETQVKALSET
RSKCTCLHE
