# Supplementary material for: Genome- and Transcriptome-Wide Characterization and Expression Analyses of bHLH Transcription Factor Family Reveal Their Relevance to Salt Stress Response in Tomato
Source: Plants (Basel). 2025 Jan 12;14(2):200. doi: 10.3390/plants14020200 (PMC11768425; doi:10.3390/plants14020200)
Supplement: Supplementary file 1 [file plants-14-00200-s001.zip › Supplementary file S2 Gene and protein sequences of Arabidopsis 169 AtbHLHs.pdf]

Supplementary file S2. The nucleotide sequences and amino acid sequences of 169 AtbHLHs identified in the genomes of Arabidopsis.

169 nucleotide sequences

>AtbHLH001
CTTCTAGTCTTTCGAGTTCAAGCTCAAACAACAACAAAATTAATATTCAAAACAACATTAAT
AAAAAAAAAGTATGGAGTGACCAAAGAAAAAAAAAACTCTCAAACATTTCTCTGTTTGTT
CCGGCGAAAACGGCAACTGTTTCATCAAATGACAAACACAAAAACCTTAACATCTAGTTTG
TATCCTCTCTGATACTTCAAAAAAAATGTAAAGAGTTACAAGTTTCCCTTTTTCTTTCTTTTT
TAGGCTATAAATTTAACATAGTATCTTATTTCTTTCTAAATCAGGACAAAAAGTTTGATTTTTT
TCTTTTCTTTTCTTTTTCCCTGTGTGTGGTTTCATGGGATATAGGGATGAAGAAACAATGGCT
ACCGGACAAAACAGAACAACTGTGCCAGAGAATCTGAAGAAACACCTCGCAGTTTCAGTT
CGAAACATTCAATGGAGTTATGGTATCTTTTGGTCTGTCTCTGCTTCTCAGTCTGGGTAAGTT
TCTTAAATCATATTCATTGAACACTAGATATTGGAGATTTTGAAAGATATTGATGAAGTAAATT
TCTTGATTTTAATTGGAAAATTAGATAAAACTTATGTGTTCACTTCAAATCAAAACTAGTGTT
TAGTTCTACAACTCAAGACAAGGTTCTCTAGGTTTTTCTTGATTCCATTGATTTTTTCTTATAA
ACTCATATAAGGTTTCTTGATCTTCTTTAAAAGTTCAATTTTTTTTCTCTTCTTCTTGAAGTTC
TTGATTAGTTCAAGAAAATGTTTTGTTTGTTTTCTTTATTCGATTTGATATTTGTTTTTGTTTTT
CTATATATAAAAAGAGTTTTAGAATGGGGAGATGGATACTATAATGGAGATATCAAAACGAG
GAAGACGATTCAAGCTTCGGAGATCAAAGCTGATCAGCTTGGTCTACGGAGGAGCGAGCA
GCTTAGCGAGCTTTACGAGTCTCTCTCCGTCGCTGAATCTTCTTCTTCAGGCGTTGCTGCCG
GATCTCAAGTCACCAGACGAGCTTCCGCCGCCGCACTTTCACCGGAAGATCTCGCCGACAC
CGAGTGGTACTATTTGGTTTGTATGTCTTTCGTCTTCAACATTGGTGAAGGGTAATTCCGTCT
TTTCCTCTATCAGCATTGACATTTATTATGTGTCATGATCGTGTTGATCTTTGCCAGTTTTAAA
CAACGTGCCAAAATATACTTTTAATACTAGTAGGTCCAAGGATTTCGACTTTTATGGTTCAGT
TTAGGTTTGTTAGATTTCAGGGTTCTGCAGGAAAAAAGTATGAGCTAGAACCAATTTTTTAG
TTTCAGTTTGGTTTCAAAATTTAATGTCCGGATTTGGTCGGTTTCAGATTACAAAAGAAAAA
GTTAAAACATAATTAATTTTAAATAATAATGTGTATATATATGCATTAGTATGTTTAACTCTATT
CTACATCTAATAAATTTTATTAATTCGGTTTGGTTGCAGTATATTTGTGTTCGATTCGAGTTTA
GTTCGGTTTATTTATCATCACAAAAGTTTGTTTCTGTCCGGATTAATTAAAAAATTCGTTTCAT
TTTGGTCTTTATGAACACGGCTAATTAACTTTCAATAATTATGAATATGTTAGGAAGAAAAAA
TGTGGAAAAATTAAGCTTAGAATGCATTGCTGCTTTCTAAATGAACCTGTCTTTTTGCAGAA
TGCCTGGACGGACGTTTGCAAACGGTGAACCGATATGGTTGTGCAACGCTCATACGGCGGA
TAGTAAAGTGTTTAGCCGTTCTCTTCTAGCAAAAGTAAGTTGTAGTATTGATGTAGTTGCTAT
GTAACACTTTTTTGCGATTTGAAAACACTAAAATCGTTCGTTTTTGTTTGCGTTTTGTGGTGG
TTTAGAGTGCTGCGGTTAAGACAGTGGTTTGCTTCCCGTTCCTTGGAGGAGTCGTTGAGATT
GGTACCACAGAACATGTAAGCCAATATTTGTGTTTTCTGCTTCCAATGTTTTAGAATAATTATT
ATGAAAATCCTTTATTAAGAAAAAAAAGAATAATTATAAAATTACCACAATGTATTCTAACATT
TCAATTTGACCCTTCAATCTGTCGAATAACGGTATAGACACCAAGAACACACAAACTAATAT
TTTAGTAGTCTCGATTTTTAAAGATTTCCACGGTTTGGTTATGACAAAACGCGAAAATACGG

CTTTTGTACTTTTTACTAGCTCGTTGGTATAGTTATAATTGAAAACTTTTTCAAGGCTTTTTCT
TGGTGTGTTTCGTCTCGTAGATTACGGAAGACATGAATGTAATACAATGCGTGAAGACATCA
TTCCTCGAAGCCCCTGATCCGTACGCTACAATATTACCAGCAAGATCCGATTATCACATCGAC
AACGTTCTTGATCCGCAACAGATTCTAGGCGACGAGATTTACGCGCCTATGTTCAGTACGGA
GCCTTTTCCAACAGCTTCTCCGAGCAGAACTACCAACGGTTTCGATCAAGAACATGAACAA
GTAGCAGATGATCATGATTCTTTCATGACCGAAAGAATCACTGGAGGAGCTTCTCAGGTGC
AAAGCTGGCAGCTCATGGACGACGAGCTTAGTAACTGCGTTCACCAGTCGCTAAATTCCAG
CGATTGCGTCTCTCAAACGTTTGTTGAAGGGGCGGCTGGACGGGTTGCTTACGGTGCAAGA
AAGAGTAGAGTTCAAAGACTAGGGCAAATTCAAGAGCAACAGAGAAATGTGAAGACATTG
TCATTTGATCCAAGAAACGACGACGTTCATTACCAAAGTGTGATCTCAACGATTTTTAAGAC
CAACCATCAGTTAATTCTCGGACCGCAGTTTCGAAACTGCGATAAACAGTCAAGCTTCACT
AGGTGGAAGAAATCATCGTCATCATCATCAGGAACCGCCACGGTCACGGCACCATCACAAG
GAATGTTAAAGAAAATTATTTTCGATGTTCCGCGAGTGCACCAGAAAGAGAAGTTAATGTT
GGACTCACCAGAAGCCAGAGATGAAACTGGGAACCATGCGGTTTTAGAGAAGAAGCGCCG
CGAGAAATTGAACGAACGGTTCATGACCTTGAGAAAAATCATTCCGTCAATCAACAAGGTA
TATTTATATAATCGATTCTTCAAAATTTCAAATCCATATATATCGCGAGGTAACTAATCTTATGT
TTAACATTTTCGATAGATCGATAAAGTATCGATTCTTGACGATACGATAGAGTATCTTCAAGA
ACTCGAGAGACGGGTTCAAGAACTAGAATCTTGCAGAGAATCAACCGATACAGAGACTCG
TGGGACGATGACGATGAAGAGGAAGAAACCATGCGACGCAGGAGAAAGAACATCAGCTAA
TTGCGCAAATAATGAAACAGGAAATGGGAAGAAGGTGTCGGTTAACAATGTTGGTGAAGC
CGAGCCAGCAGATACCGGTTTTACTGGTTTAACCGATAATTTAAGGATCGGTTCGTTTGGTA
ATGAGGTGGTTATTGAGCTTAGATGTGCTTGGAGAGAAGGAGTATTGCTTGAGATAATGGAT
GTGATTAGTGATCTCCATTTGGATTCTCATTCGGTTCAATCCTCGACCGGAGACGGTTTGCTC
TGCTTAACCGTCAATTGCAAGGTATACTCTCTAACCTACTTTTGGCTTAACCAGGATTTGGTT
AACCTAGATTGACATGAAATGTGTTGGTTTCAGCACAAGGGGTCAAAAATAGCGACACCAG
GAATGATCAAAGAAGCACTTCAAAGGGTTGCATGGATCTGTTGAAGACTACTTAGTTAAAA
TTGACAGCAAAGAAAAAACATTCCCGGTTTGGTTTCTATTCTTTGGTTTTCTTCTAACCGGG
TTTTAGGAATTAATGTTATGTTTATCATTTGTTTTTTGTTTTTTTTTTGTGTCTTTTTTTCCGTT
GCTTAACGTAGGTGAAGAGGAACATACACTATGCGTATTTTGTTTGAGGTAGATTATTTTAAG
GGTATTAGTAATAGTAATAGCCAGTTTAGATGATTTTGTGTTCTTTTGTTGTTTAAGCACAAG
GTTGATGTGAAATTACGGGGAAAAAAACATTAATGTGTAAATGAAAATTAGTGGTCAAAAA
GCTTCACACGAAACGGGAGAAGATTCTGCATTTTGGCATTTTCATGGATAACAAAGAGGTA
AAGTTTATGATAAGAAATCCAGATTAATT

>AtbHLH002
TTCGGACACCGTCCTTTTCTCTCAACTCAAAGCTCAATTCAACCACAACAGATAGTAACTTG
AAAGTCACAAATATTTAATATTTTCCCATTATAGAAGGTTAAAAAAAATAAATATATTCTGGG
TTTTGTTGTCTTCTTCTTTCTTCCTCAGGAAACCAAACCAATGTCATGTAAAAATCACTAACT
CATGACTAGTCATAACCTTTAAATCACTTCTGCTTTTTTTCTGTTTCTCTTTTGATTTATTCAA
AGACAACAAAGTTTTGTTTTTGACAAAAAATCTGATCTTTGTTTGTTCGTTTGTTTGTTACAT
GGGGATGAAGAAACAATGGCAACCGGAGAAAACAGAACGGTGCCGGACAATCTAAAGAA
ACAGCTCGCAGTTTCAGTTCGAAACATTCAATGGAGTTATGGAATCTTCTGGTCTGTCTCTG
CTTCTCAACCAGGGTACAATCTCGTAATCTTTATCCTTCAAATTAACTTTTCTTGAAAATAAA
GTTTTGTTTGAGACATTCTTGACTTTAGTCCAAAACTCAGATAAAACTTTATCCTTACTTCAA

GATTTCGATTTTTGTTATGTTTTCTCGATTAATCCAATTTTTCTTGGCTATCTTTTTTGTTTTCT
CTAAACGAAGAGTGTTGGAGTGGGGAGATGGATATTACAATGGAGACATAAAGACAAGGA
AGACGATTCAAGCAGCAGAAGTCAAAATTGACCAGTTAGGTCTTGAGAGAAGTGAGCAGC
TTAGAGAGCTTTATGAATCTCTCTCCCTCGCTGAATCCTCAGCTTCCGGTAGCTCTCAGGTC
ACTAGACGAGCTTCCGCCGCCGCTCTCTCACCGGAGGACCTCACCGACACCGAGTGGTACT
ACTTAGTATGCATGTCTTTCGTCTTCAACATCGGTGAAGGGTAATTCCGTCTCTTCCTCATAA
TCCTTTCATTTCACCTTTTTTAAAAACAAAAACCTATCTTTCAGAAAAATTTCTTTTATGAAG
AATATCGTTTTTAAATTATAGTATTATTTTATTCGTAGACATTATAAGAAAATGTAAACGATACC
AAAATTCTAAAAAAAAAGTATAAGGGTATGTTTTATTTTGTCTCATAAAAAAAGACAAGAAC
ACCCTTTATTAGCTTGTCTCCTTCATTTCTTCTTCCATGTGAGCTTATTCAGAAATCCGACATG
TACGAGTTTAATTATTAACAATGCAATTATCCTTTATATTTACTTAATTTTTTTTATGTTTGTAG
GTTTTTTTAAAGTATACCTCTTTTTAATCTTAGAAGACATACATAGATAGAAATGCGAAAGCA
CAACGGAAATAAGTTTTTACTTCATTTGCATAATTGCATTATACATTTGCTATTCAAAGAAAG
TAGTAGAAAACTTTTGTTCCCATTAAGATAAAGATTTTGAGATACAAAACATAAAGGGTATT
TCCGTCTTTCTAATGTAGAATCCCCGGAGGAGCGTTATCCAATGGAGAACCAATATGGCTTT
GTAACGCTGAAACCGCCGATAGCAAAGTCTTCACTCGTTCTCTTCTAGCTAAAGTAAGTTTT
TTGCAATTGGAAACGCTAACGCTAGAATTCAAGAACGCTATAACTGAAAACGCTCTTTTTTT
TTTTTTTTTCGTTTGGCGTTTTGCTCTTTAAAGAGTGCTTCGCTTCAGACAGTGGTTTGCTT
CCCGTTTCTTGGAGGAGTCCTTGAGATTGGCACGACCGAACATGTAAGCCTATATTTCCTATT
TTTTTTCCAACACTATCAAAACATTCTTAAAAGTACCCAAAACTTATCTTTTGTTCTTTTAGA
TTAAAGAGGACATGAACGTGATACAAAGTGTTAAGACGTTGTTCCTTGAAGCTCCTCCATAT
ACTACAATATCGACAAGATCAGACTATCAAGAAATTTTTGATCCCTTAAGTGACGATAAATA
CACTCCGGTGTTTATAACCGAAGCTTTTCCAACAACTTCTACTAGCGGGTTTGAGCAAGAAC
CTGAGGATCATGATTCGTTCATCAACGATGGTGGTGCGTCTCAGGTACAAAGCTGGCAGTTT
GTGGGTGAAGAAATCAGTAACTGCATTCACCAATCGTTAAATTCAAGCGATTGCGTTTCCCA
AACGTTTGTTGGAACAACCGGGAGACTTGCTTGCGATCCAAGGAAGAGTAGGATTCAACG
GTTAGGTCAGATTCAAGAACAGAGTAACCATGTAAATATGGACGACGATGTTCATTACCAAG
GCGTGATATCGACGATTTTCAAAACAACGCATCAGCTAATACTCGGACCGCAGTTTCAGAAC
TTCGATAAGCGGTCTAGCTTCACAAGGTGGAAGCGATCATCATCTGTGAAAACATTGGGAG
AGAAATCGCAGAAGATGATAAAGAAGATACTCTTCGAGGTTCCTTTGATGAACAAGAAAGA
AGAGTTGTTACCGGACACACCAGAGGAAACCGGGAACCATGCCTTGTCCGAGAAGAAACG
CCGCGAGAAATTGAATGAACGGTTTATGACATTGAGATCAATCATTCCCTCAATTAGTAAGG
TAACCGATTCTAGCAAATCTGTTTCAGTAACGTTTTATGTTTATTTCTTGATAATAGGAAGAC
ATGTTTAAATATAATGCTCTGAAGGAACTAAGCTTGAGTCTCTTGCTGATTCTACAGATTGAT
AAAGTGTCGATTCTTGATGATACAATTGAGTATCTTCAAGATTTACAGAAACGGGTTCAAGA
GTTGGAATCTTGTAGAGAATCTGCTGATACAGAGACACGGATAACGATGATGAAGAGGAAG
AAACCGGATGATGAGGAGGAAAGAGCATCAGCGAATTGTATGAACAGCAAAAGGAAGGGG
AGTGATGTGAATGTAGGAGAAGATGAACCAGCTGATATCGGTTATGCTGGTCTAACGGATAA
CTTAAGGATCAGTTCATTAGGTAACGAGGTGGTTATTGAGCTTAGATGCGCTTGGAGAGAAG
GGATATTGCTTGAGATAATGGATGTGATTAGTGATCTCAACTTGGATTCTCACTCGGTTCAGT
CGTCAACCGGAGACGGTTTACTCTGCTTAACTGTCAATTGCAAGGTACAGTCATTAAACCA
AGCTTGATTTATGTTACTCAACCGGAATATGAGCAAACCGAAATGTGATATGTAATGTTTAAT
GTTGATTGCAGCATAAAGGGACAAAAATAGCAACAACAGGAATGATCCAAGAGGCACTTC
AAAGGGTTGCATGGATATGTTAAGGATTCAAGGTTTAGATTTGACAAAATTAGCTTTTTCTCT

GGTTTGGTTTCCCAATTTTCGGTAATCAGTGATTCTAACCGAGTTTTTGTGATTTTGAAGTTT
TGGAAGATCCTTAAGAATTCTTGAAAAATCGTTCCTTTTGGCTCATTACGTAGGAGAAGACA
ATAAGAAATTACTTACTTAGCAATTAGCATACTATGAAATTCAATTATGCGAAATATGAAAAC
CACTCGTAGTAGTTCGTGCACTATCATTTTTAAAAGTAAGTAAACTAGAACTTTTTTTTTGTT
GTTGAAAGTAACTTTTGTATAAGTATTTGTAACGAGATCATATTTTGTGACTAGTTCAAGTTC
GACCAAAAACATTATAGTAAAATCTAAAGAGATACTACATAGCCCGATTCCAGTATCTTAAGT
CTTCAATCTCACT

>AtbHLH003
CATAGTCTCTTCCTCTTTTCTTCTTTCTTCGTCAATTTCAGAACAAGTCACTACAGATCTGAA
GTGAACCAAGCTCAGGTTTTGTCTTCTCTTTGATCATTCCTTTCTCAGCAATATAAATTAGAG
TTATATCCTTTATAAAGGATTTTGCTTTTTCACCAACAAACCCTAAATTCGGTGTCTCAGCAA
GAATCACGTGATTCTCGTTCCTCTTCCTCACGAAACCCATCATCTTCTATCTCATTTGTAAGC
AGCGATCTCAATCCAAATACCTTTAATTTTATTTGATTCCATACCCTAATTTTTGTATATTCCAT
GTCAATGAACGTTTCAGGAGAAATGGGTCAAAAGTTTTGGGAGAATCAAGAAGATCGAGC
GATGGTTGAATCCACCATAGGCTCTGAAGCTTGCGACTTTTTCATCTCAACAGCTTCAGCTT
CCAACACTGCCTTGTCCAAGCTTGTCTCACCACCAAGTGATTCCAATCTCCAACAAGGGTT
ACGTCACGTTGTTGAAGGATCTGATTGGGATTATGCTCTTTTCTGGCTAGCGTCCAACGTTA
ATAGCTCTGATGGTTGTGTCTTGATCTGGGGAGATGGTCATTGCCGTGTCAAAAAGGGTGCT
TCAGGTGAGGATTACTCTCAGCAAGATGAGATCAAAAGACGTGTGCTTCGCAAGCTTCACT
TGTCGTTCGTTGGTTCAGATGAAGATCATCGTTTGGTGAAATCAGGAGCTCTTACTGATCTC
GACATGTTTTATCTGGCTTCTTTGTACTTTTCCTTTAGGTGTGATACCAATAAGTACGGTCCT
GCTGGAACCTATGTGTCTGGGAAGCCTCTTTGGGCTGCAGATTTGCCTAGCTGCTTGAGTTA
TTATAGGGTTAGGTCTTTCTTAGCTAGGTCAGCTGGTTTTCAGACTGTGTTGTCTGTACCAGT
GAATTCTGGAGTTGTGGAGCTTGGTTCTTTAAGACATATTCCAGAAGATAAGAGTGTGATTG
AGATGGTGAAATCAGTGTTTGGTGGGTCTGACTTTGTTCAGGCTAAAGAAGCTCCTAAAAT
CTTTGGTCGACAGCTGAGTCTTGGTGGAGCAAAACCTCGGTCTATGAGTATTAATTTCTCCC
CGAAGACCGAGGATGACACGGGTTTCTCATTGGAATCGTATGAGGTGCAAGCGATCGGAGG
CTCTAATCAAGTGTATGGTTATGAGCAAGGGAAAGATGAGACATTGTATCTAACTGACGAGC
AAAAGCCGAGGAAGAGAGGGAGAAAACCAGCAAATGGAAGAGAAGAGGCTCTAAACCAT
GTGGAAGCGGAACGGCAGAGGAGGGAGAAGCTGAACCAGAGATTCTACGCTTTGAGAGC
GGTGGTGCCTAACATCTCCAAGATGGACAAGGCTTCGCTCCTTGCAGACGCAATCACTTAC
ATCACGGATATGCAGAAGAAAATCAGGGTGTATGAAACAGAGAAGCAGATAATGAAGAGG
AGGGAGAGTAATCAGATAACTCCAGCAGAGGTTGATTATCAACAGAGGCATGATGATGCAG
TTGTAAGGCTAAGCTGTCCGTTGGAAACTCATCCAGTTTCAAAGGTGATACAAACGTTGAG
GGAGAATGAAGTTATGCCTCATGATTCCAACGTGGCCATCACAGAGGAGGGTGTGGTTCAC
ACATTCACTCTCCGGCCTCAGGGTGGCTGCACCGCTGAGCAGTTGAAGGACAAGCTCCTTG
CCTCTCTATCACAGTAACTATCACAGCAGTAACTGCTATGTAATAAGTGTAACCGTGTTGGA
GGTTGTATCAATGTACTATTGCAAGCCAACCAAAAAAAACTCCAGCTTAGTAGGATCGTGTA
ATTTTCCTTATATGTAATGTTGAGATTTGTCTTTTACATATAAAGATTTGAGCAGAAAGCTTCA
CACTT

>AtbHLH004
AAATAACTCAATCAATAATTCATATTTGGTGAAAATAAAAGTACTATTTCTTTTTCTTTAGAGT

TAAAAAAAAAAAAATGAATATAAACAGATTCCTTTTCCCGAAACAATCAAACCAAACACAAA
TATTTTGCTTAAAGAACTTACTTCTCTCCTCTGTCTCTCACCGGAAAAACAAAAAGTCTTCT
CTTTTATAACTACGTCAGAGAACTGTTATGTCTCCGACGAATGTTCAAGTAACCGATTACCAT
CTCAACCAATCAAAAACGGATACAACAAATCTCTGGTCAACCGACGACGATGCATCGGTAA
TGGAAGCTTTCATCGGCGGCGGCTCCGATCATTCTTCTCTTTTTCCTCCACTTCCTCCTCCTC
CTCTTCCTCAAGTCAACGAAGATAATCTCCAGCAACGTCTCCAAGCTTTAATCGAAGGAGC
AAACGAGAACTGGACTTACGCCGTGTTCTGGCAATCATCTCACGGTTTCGCCGGAGAAGAC
AACAACAACAACAACACAGTGTTGTTAGGTTGGGGAGATGGTTATTACAAAGGAGAAGAA
GAGAAGTCTAGAAAGAAGAAATCAAATCCAGCTAGTGCAGCTGAACAAGAGCATCGTAAG
AGAGTGATTAGAGAGCTCAACTCTTTAATCTCCGGTGGTGTAGGAGGAGGAGATGAAGCTG
GAGATGAAGAAGTTACAGATACTGAATGGTTCTTCTTAGTTTCAATGACACAGAGCTTTGTC
AAGGGTACTGGTTTACCTGGTCAAGCTTTCTCAAATTCAGACACGATTTGGTTATCTGGTTC
TAATGCTTTAGCTGGATCAAGTTGTGAGAGAGCTCGTCAAGGTCAGATTTATGGGTTACAAA
CAATGGTGTGTGTAGCGACAGAGAATGGTGTCGTTGAGCTTGGTTCGTCGGAGATTATTCAT
CAAAGTTCAGATCTTGTTGATAAAGTTGACACCTTTTTCAATTTTAACAATGGTGGTGGTGA
ATTTGGTTCTTGGGCGTTTAATTTGAATCCAGATCAAGGAGAGAATGATCCAGGTTTGTGGA
TTAGTGAACCTAATGGTGTTGACTCTGGTCTTGTAGCTGCTCCGGTGATGAATAATGGTGGA
AATGACTCAACTTCTAATTCTGATTCTCAACCAATTTCTAAGCTTTGTAATGGAAGCTCTGTT
GAAAACCCTAACCCTAAAGTTCTGAAATCTTGTGAAATGGTGAATTTCAAGAATGGGATTG
AGAATGGTCAAGAAGAAGATAGTAGTAATAAGAAGAGATCACCGGTTTCGAATAATGAAGA
AGGGATGCTTTCTTTTACCTCTGTTCTTCCATGTGACTCGAATCACTCTGATCTTGAAGCTTC
AGTGGCTAAAGAAGCTGAGAGTAACAGAGTTGTGGTTGAACCGGAGAAGAAACCGAGGA
AACGAGGGAGAAAACCGGCGAATGGAAGAGAAGAGCCTTTGAATCATGTAGAGGCAGAG
AGACAGAGAAGAGAGAAGTTGAATCAGAGATTCTATTCTTTAAGAGCTGTGGTTCCTAATG
TGTCTAAGATGGATAAAGCTTCTCTATTAGGAGATGCTATTTCGTATATCAGTGAGCTTAAGT
CTAAGTTGCAAAAGGCTGAATCTGATAAAGAAGAGTTGCAGAAGCAGATTGATGTGATGAA
TAAAGAAGCGGGAAATGCGAAAAGTTCGGTAAAAGATCGAAAATGTTTGAATCAAGAATC
GAGTGTGTTGATAGAGATGGAGGTTGATGTGAAGATTATTGGTTGGGATGCAATGATAAGGA
TTCAATGTAGTAAGAGGAATCATCCTGGTGCTAAGTTCATGGAAGCACTTAAGGAGTTGGAT
TTGGAAGTGAATCATGCGAGTTTATCGGTAGTGAATGATCTTATGATCCAACAAGCGACTGT
GAAAATGGGGAATCAGTTTTTCACGCAAGATCAACTCAAGGTTGCTCTAACGGAGAAAGTT
GGAGAATGTCCATGAATTGAAGTCAGCATCTTTAGGGCTAATACACCGGAGAATACTGCGA
AAAGTCGAAAACAACGATCATAGTATAAGCCGCGGTAAAAAGTGTTAAACCTTTCACACAA
GTTTCTCTAGTGAATGTAGTTGTAAACTCTATTGTGTAAGGGTAATTTTGTAGTACCCACTTG
TTGCTATTGAATGCTTGTTAGAGAGGATTCTTAGTGTAGTATATGATTAGGTTGGGGTTTGTT
GTTTCATGAGATAAATAAATGTGTTTGATCAATGGTTAAGTCTTTGGTTTGTTGGTGTATGTAT
GTAAATAAGGCTTTTGTTAGAAATAAGACAAATGGGACTGAAGTTGGAGTTTTTTGTTGTCT
TTTGAGTTAAAGTTTGA

>AtbHLH005
ATTTACAATTGTTTTAGAAAAACAATAAAAATAAAATATAGTATTGTAGAGAGAATCATCTTC
TCCAAACAATCACTAAACGCCATTTGCTTAGCTTTTTCCCTCTTTTGATTTTTCCTCCCTACA
CGTCACCTCTCTCTTCTTCTCACCGGAAAAAAGTCTTCACCTTTCACCGGAAAATATCACCT
TTTCAACCGGCGTATGTTCACATGAACGGCACAACATCATCAATCAACTTCTTGACCTCCGA

CGATGACGCGTCGGCGGCGGCTATGGAAGCTTTCATTGGAACAAACCACCACTCATCTCTC
TTTCCTCCACCACCACAACAACCACCTCAGCCTCAGTTCAACGAAGATACTCTTCAACAAC
GTCTCCAAGCTTTAATAGAATCCGCCGGAGAAAACTGGACTTACGCTATCTTCTGGCAGATC
TCACACGACTTCGATTCATCCACCGGAGATAACACAGTGATCCTCGGCTGGGGAGATGGTT
ACTACAAAGGAGAGGAAGATAAAGAGAAGAAGAAGAACAACACCAACACGGCGGAGCAA
GAGCATCGGAAAAGAGTAATACGTGAGCTTAACTCGTTAATCTCCGGCGGAATTGGGGTTT
CCGATGAATCAAACGATGAAGAAGTAACAGATACTGAATGGTTCTTCTTAGTTTCGATGACT
CAAAGCTTCGTTAACGGTGTTGGTCTCCCCGGAGAATCTTTCTTAAACTCTCGTGTGATTTG
GTTATCCGGGTCTGGTGCTTTAACCGGGTCGGGTTGTGAAAGAGCGGGTCAAGGTCAGATT
TACGGGTTAAAGACGATGGTGTGTATCGCGACTCAAAACGGCGTCGTTGAGCTTGGTTCGT
CGGAGGTTATAAGTCAAAGCTCAGATCTGATGCATAAAGTTAACAACTTGTTTAATTTCAAC
AACGGTGGTGGAAACAATGGTGTTGAAGCTTCTTCGTGGGGTTTTAATCTGAATCCAGATC
AAGGAGAGAATGATCCAGCTTTGTGGATTAGTGAACCGACGAACACCGGAATCGAATCTCC
GGCGAGGGTTAATAATGGTAATAACTCGAATTCTAATTCTAAGTCTGATTCTCATCAAATTTC
TAAGCTTGAGAAGAATGATATTAGCTCTGTAGAGAATCAGAATCGTCAAAGTTCGTGTCTTG
TCGAGAAAGATTTGACCTTTCAAGGTGGGTTGTTGAAATCTAATGAGACTTTGAGTTTCTGT
GGTAATGAGAGTAGTAAGAAGAGAACTTCGGTATCTAAAGGGAGTAATAATGATGAAGGGA
TGCTTTCGTTTAGTACTGTGGTTAGATCAGCTGCGAATGATTCGGATCATTCTGATCTTGAAG
CATCTGTTGTTAAGGAAGCGATTGTTGTTGAGCCACCGGAGAAGAAGCCGAGGAAACGGG
GGAGGAAACCGGCGAATGGGAGAGAAGAGCCGTTGAATCATGTTGAAGCAGAGAGGCAG
AGAAGAGAGAAGTTAAACCAGAGATTCTACTCTTTGAGAGCTGTTGTTCCTAACGTTTCGA
AGATGGATAAAGCTTCGCTTCTCGGAGACGCGATTTCGTATATCAATGAGCTTAAGTCGAAG
CTGCAGCAAGCGGAGTCTGATAAAGAGGAGATTCAGAAGAAGCTAGATGGGATGAGTAAG
GAAGGGAATAATGGGAAAGGTTGCGGGTCAAGGGCAAAAGAACGGAAAAGTTCGAATCA
AGATTCTACGGCGAGTTCTATAGAAATGGAGATTGATGTTAAGATCATAGGTTGGGATGTGA
TGATACGTGTACAATGCGGCAAGAAAGATCATCCCGGTGCTAGGTTCATGGAAGCACTTAA
GGAATTGGATTTGGAAGTGAATCATGCGAGTTTATCCGTTGTGAATGATTTGATGATTCAAC
AAGCTACGGTGAAGATGGGGAGCCAATTTTTCAATCATGACCAGCTCAAAGTTGCTTTGAT
GACGAAAGTCGGAGAAAACTATTGAACAAAGTTGGCATATCAAAGAGGGGAAACTAGTGT
TTTCATCGATGAAATGGGATCGAATTTAGCGGGTAATGTATGGTCTTTTGTAAAGTAGTAAGT
ACTCATTTTGCATCACTCGCTTCATGTTGTTAAATGTTGTTGTTCATTAGGAGAACTTGCTTA
GTTCTTAGCTTAGTGTTATCATAAGCCTTAAGTGAGAAGGAAGTTGGAGACTAGAAGACCA
GAGAGAGTTAGCGAAATAATAAAATCTTTGGTTGTACTTGTATGTATATATATGTATGGAGTA
AGATTAAGTGAGATCCTAAATTCTATTGAAGTTTTTTATTCTATGATTGAATTTTTTTGTTAG
TTTGTAACTTTGTAGTGTACATAAAGGTCCAAATTTATGAACTGTAGTGTACCAAAAAGCTG
AGGAAAATAAGTGGTTGTAAATGGGTTTTGCCTAGAGATAGTGCTCTTATCTTTGTAAGGGG
GTATGAGGAAACTGTTTATAATGCCAAAAATGTGGAGACATGTGTGATTTGTTTGTAAAAGA
AACTAAAAGTTAAACCAAAGATTTGTTAGTTTTAAGTAATGAGTAG

>AtbHLH006
TAAGTAATTAATCTGTTATAAAAAATATATTCTAAAAGTTGACAAAACGACAAATATAAATCG
AAAAAATAGGTGAAAAGTTATAGTAAAGTGTTTTTCCACTGAGCTCACAGTTCACTCTTTGG
TACAAAAGAAACAATCAGTTAAAATCTAAGTCAATCATTAAAGAGCGTGTGATACACGTGC
GACCAAACAGCCGTACGATCTCACTCACTTCCGGTCTTAATTCTTACCGCGTCGATCCAACA

GTCGATGGGGTTAAATCGGGATGTGAGTTCATCGAAGCTTCTCTCTCTCTTACAACCATCCA
CGTTTCCCAATGAAATCTCGCCACGTAATATCCCTAACAACAAATTTACACATTCACCCTCAA
GATCAGTTACACATATAATAATCGCAGACGCTCTCCATTTTTCTCCACTACGAAGACTTTCTC
CTATCTCTCTCTCTCTCATTAAAAACGTGTTTTTTTTTACCGGTCACCGGTTTATGGAATGAC
TGATTACCGGCTACAACCAACGATGAATCTTTGGACCACCGACGACAACGCTTCTATGATGG
AAGCTTTCATGAGCTCTTCCGATATCTCAACTTTATGGCCTCCGGCGTCGACGACAACCACG
ACGGCGACGACTGAAACAACTCCGACGCCGGCGATGGAGATTCCGGCACAGGCGGGATTT
AATCAAGAGACTCTTCAGCAACGTTTACAAGCTTTGATTGAAGGAACACACGAAGGTTGG
ACCTACGCTATATTCTGGCAACCGTCGTATGATTTCTCCGGCGCCTCCGTGCTCGGATGGGG
AGATGGTTATTACAAAGGTGAAGAAGATAAAGCAAACCCGAGACGGAGATCGAGTTCGCC
GCCGTTTTCTACTCCGGCGGATCAGGAGTACAGGAAAAAAGTGTTGAGAGAGCTTAACTCG
TTGATCTCCGGTGGTGTTGCTCCGTCGGATGACGCTGTTGATGAGGAGGTGACGGATACGG
AATGGTTTTTCTTGGTTTCGATGACGCAGAGCTTCGCTTGCGGTGCGGGATTAGCTGGTAAA
GCGTTTGCAACGGGTAACGCGGTTTGGGTTTCCGGGTCAGATCAATTATCCGGGTCGGGTT
GTGAACGGGCTAAGCAAGGAGGAGTGTTTGGGATGCATACTATTGCGTGTATTCCTTCGGCG
AACGGAGTTGTGGAAGTCGGGTCAACGGAGCCGATCCGACAGAGTTCGGACCTTATTAAC
AAGGTTCGAATTCTTTTCAATTTCGACGGCGGAGCTGGAGATTTATCGGGTCTTAATTGGAA
TCTTGACCCGGATCAAGGTGAGAACGACCCGTCTATGTGGATTAATGACCCGATTGGAACA
CCTGGATCTAACGAACCGGGTAACGGAGCTCCAAGTTCTAGCTCCCAGCTTTTTTCAAAGT
CTATTCAGTTTGAGAACGGTAGCTCAAGCACAATAACCGAAAACCCGAATCTGGATCCGAC
TCCGAGTCCGGTTCATTCTCAGACCCAGAATCCGAAATTCAATAACACTTTCTCCCGAGAAC
TTAATTTTTCGACGTCAAGTTCTACTTTAGTGAAACCAAGATCCGGCGAGATATTAAACTTC
GGCGATGAAGGTAAACGAAGCTCCGGAAACCCGGATCCAAGTTCTTATTCGGGTCAAACAC
AATTCGAAAACAAAAGAAAGAGGTCGATGGTTTTGAACGAAGATAAAGTTCTATCATTCGG
AGATAAAACCGCCGGAGAATCAGATCACTCCGATCTAGAAGCTTCCGTCGTGAAAGAAGTA
GCAGTAGAGAAACGTCCAAAGAAACGAGGAAGAAAGCCAGCAAACGGTAGAGAAGAGCC
ACTAAACCACGTCGAAGCAGAGAGACAAAGACGCGAGAAACTAAACCAAAGATTCTACGC
GTTACGAGCGGTTGTACCAAACGTTTCAAAAATGGATAAAGCTTCGTTACTCGGTGACGCA
ATCGCTTACATCAACGAGCTTAAATCCAAAGTAGTCAAAACAGAGTCAGAGAAACTCCAAA
TCAAGAACCAGCTCGAGGAAGTGAAACTCGAGCTCGCCGGAAGAAAAGCGAGTGCTAGT
GGAGGAGATATGTCGTCTTCGTGTTCTTCGATTAAACCGGTGGGGATGGAGATTGAAGTGA
AGATAATTGGTTGGGACGCAATGATTAGAGTTGAATCTAGTAAGAGGAATCATCCGGCGGCG
AGGTTGATGTCGGCGTTGATGGATTTGGAGTTGGAAGTGAATCACGCGAGTATGTCGGTGG
TTAACGATTTGATGATTCAACAAGCGACGGTGAAGATGGGTTTTAGGATCTATACGCAAGAA
CAGCTCAGAGCAAGTTTGATTTCAAAAATCGGTTAAAAGGGTGTGTTTTGGGAAGTTTAGA
AAGTTATGGGGTCAAATCATAATTAATTCGTTTTAGTGGCTTCAGTAATTTTGTAGATTTTAGT
TTTGTAAGAAAAAAATCTTAAAATAGAGCGACAAGTTTCTTCTTTTGCTCTATGTTTGAGTC
TGTATCGTTTTATTGTTGTATCTCCTCAATGAGTAAACTTGTATATATTGATATGAGTAATATGA
GTTAGTTACTGAAAAAGTTAATATTTCTACACAATTTCATATGATAAAAAAGAAAAAGAAAT
CGAGTATAAATGGTCTAAAATAAGAGACTAATGGTCATGATCACTGATTTTGTACATATGGTC
AAAAATGTCTCTCCTAACACATCAAACTTAGGAAGAGAGACAAATCTTAAGTAAGTTAAGT
CGAGAGGGAGACCATATGCACAGTTTTCTTAAGTCAGTGTGATAATGTTTCCTCTTGTCTAAT
CAATAAGACACAACACACATAAACCTTAGTATAATACTACATGGATCTTCGAATACACAGAC
AGAGTCACAGACAAGAAAAAGCAAATCACTTGCTTTTGTCTTGATCATTTCCAATATCTACA

AAAATAGAAATCTTGAAGAGATTATTGAATCTGAAATCTGCTTTGTTTGTGCATTCTCTTCCT
CGATCACCTCGCAAGATCCTCTAGCAACCTGACCAATCCTCAAGAATCAAGTTTTCTTCTTT
CTAGCTTCAGCTTTCCAATCTCGACGATGCGGCCTTATCTTCCGTTTAACCGACTCACAAGA
ACTATCTTGAGCCTTGTTCATAATCAAATGAGCTCTTCTAGCTTCTTTTTTCCAATTTCTATCG
CAATAAACCCGCCGCACAAAGTTCTCAACATAACCACTAGGTCCATTAAACTCTAGTCCCTC
GTCTTCTTCTTCTTCTTCTTC

>AtbHLH007
CATAAAGTGAGTCTTTTTGTCATATCCTTTCTCGTCTGTGTCTCTTCGTCCTCATTAGCTTAAG
CATCATCATTCTCTCACCTAACAACTCCACACAAGATTCTTCGCATGGAAAGTTGTTCTTCG
ACTTCTCTTCTCTAACTCGCTATCTTTTAACTCACCCAGCTCCACTGAGTCGAAAATTTCAA
ACCTTTACTCGTTTCCTTCATGGCTAATAACAACAACATCCCACATGATAGCATCTCCGATCC
ATCTCCTACCGACGATTTCTTCGAGCAGATCCTCGGGCTTTCCAACTTCTCCGGTTCTTCAG
GTTCTGGTCTCTCTGGAATCGGCGGCGTGGGTCCACCTCCGATGATGCTTCAGCTTGGTTCA
GGCAACGAAGGGAATCATAATCATATGGGTGCCATTGGAGGAGGTGGACCTGTAGGGTTTC
ATAATCAGATGTTTCCGTTGGGATTAAGTCTCGATCAAGGGAAAGGACATGGCTTTCTTAAA
CCTGATGAAACTGGTAAACGTTTCCAAGACGATGTTCTTGATAATCGATGTTCCTCTATGAA
ACCTGTAAGTTTCTCTTCAGAATCTTCACTTTGTCTTTTTCTTTTGTGTTCTCGAGAAAATAA
AGTGAAATTCAAATCTTTAGGGTTTCAAAATGGTGTCGTTTTTGAGAAAACCCAGCTTAGGT
CTAAATTTGATAGTTACCGAGAGTTTTTGGCTCAGATTGGTGAGATTTGATGTTTTTGAAGA
AACCCAAGTTGAAGATTTTGATGTGTTTTAAAGTCATAACACTAAAATTAGTTTTGCAAGAA
ACTTTAGTATAAAAGAGTGTTCTTTTATGTCCAAATTATATTGTAAGAAAATTTGCTCTTTAGT
TTCTTTTCTCATGTGGATAATTCCGTCATAGTTTGAATTGTTGCTATAACTACTCTAGTGGCGA
CCGTTCTTGTGAGAAGCGGTTTTTAAGCTTTTCTTAATGTTATTACGGCATTGTTTTGTGTTTT
GTTGAAGATTTTCCATGGGCAGCCAATGTCACAGCCAGCTCCACCAATGCCGCATCAACAG
TCTACTATTCGGCCTAGAGTTAGGGCTAGGCGAGGTCAAGCTACCGATCCACATAGCATCGC
TGAGAGGGTAAGATGTTTGATACACTTTTTTTATCTTCTGGTCTCTTTTTTCGATTTTTATGTG
ATAATGTTTGATCAAAGCTTTAATTATAGCTTTTGGTTGAGAAAGTTCGATTAGTTCTTACTTA
CTAAGAACGGTAAGACCTAACCTGAATGTATTTGCTTTGCAACAGCTCCGAAGGGAAAGAA
TAGCAGAACGGATCAGGTCGTTGCAGGAACTTGTACCTACCGTTAACAAGGTTGGTATCTTT
TTCTCTAGAGCTTCAGCTACTGTTTTCATGTCGCGAAGAGAGCTTGAAAGAGATTTTAAAAG
TAGGGAGAAAACTAGAGTTGTCAATGAGAGAAAAGTTTTGTGATTGGCATATAGAAACTGC
GGTATTCACATCTGTTTCTTTTGCTTTCCTTTTGCAGACAGATAGGGCTGCTATGATCGACGA
GATTGTCGATTATGTAAAGTTTCTCAGGCTCCAAGTTAAGGTAAACACCCAAGTCATTTTCA
GTTTTCTATTCTCTGGTTTTTGAAAATGCTATCATTAAAAGTTTGTAGAAAGATTATTTAAAA
CAGCCAGAAGGATTTAGTGGTGTAAATCCTTGGTCCCCACCACCACCTACTCTCTTAAACCA
AACTTCCAATCTTAATAAAAGATTATAATTGTTTAATCTGGCAATAAAGAGTAATCAAAGAGT
ACATGATTGTGTGTTTTTTGGCATTAGTGTCTTTCACTTTTTGCTTGCTTCCCTCCCATGGAG
AATCATCACAATTGAGGACTATTGTAATAAAGCTAATGAGAACATTGAAGAATATTTTATGCA
ATGATGGGCTTTGATAATATTTTTCCTTCATAAGCTTTATCTCTGGTCAACTATGGCAGGTCCT
GAGCATGAGCCGTCTTGGTGGAGCCGGTGCTGTCGCACCACTAGTCACTGAAATGCCATTA
TCTTCATCAGTTGAGGTTAGACTAGAGAGAGATACTTCATTAAAAAAAGAACAGAACTTTG
GTAATGGTTTTTCTTGCACTGACTTGTGGTGTAAAAACTTTCTTCAGGATGAGACGCAGGCC
GTGTGGGAGAAATGGTCAAACGATGGGACAGAGAGGCAAGTGGCTAAGCTGATGGAAGA

AAACGTTGGAGCAGCGATGCAACTTTTGCAATCAAAGGCTCTTTGCATAATGCCGATCTCAT
TGGCAATGGCGATTTACCATTCTCAGCCACCAGACACATCTTCTTCAATCGTCAAACCAGAG
ATGAATCCTCCACCGTAGATTTTTGTTCATCCAACGGTCCCCAGCTGATGATTGACATTTTGC
TCTGTTTCCCACTACTAGACTTTTGTGACTCATGAAAGGTAAGTAAAAAGGCATTGGAGATG
GAATCTAAGTAGGATTTGTGCAGTAAAGAAGTAAAACGGGATCTGTCAAAAGAAGGAAAA
AGCTCTCGCTTGCTTGGCTAGTATTTATCATTTTGATGAAAGTAACTCTTTTTTGTTCAAAGA
CTTTAGTGTGATTTTCAGGACCAAGGGCTTTGAGGGTAGTGCTAGCTGTAGTAATAGTAATG
AAGGTGTGGGATCGTGTTTCTGAATTATGTAAAAAAGGAAGAAAAAACAAATGTTGGTATT
ATATTATGGTTTTGCCTCTCTTTTTTTTCTTGCACTAATTTGGATAGACCATTTAACTATTCAA
AATGATATAGTTG

>AtbHLH008
CCTGTATCATGTAACAGAGACAGACGAAGTGTGCAGAGGTCGGTGAGAAGCAATTTGGTCA
CCATGCTCCAACTCTCTTCTCTAGGTCCCTTTTGCTTTAATTTTCTCGTTTAATAACAATATCT
TATGAAAGCAGTTACCATATCAATTGAAGGATTGTATAATACCGTAATTTATTTATATATAGTAC
ATCTAATTAAACATTAATTATTTCTACTAATAGCCTAAAACCACTACTAATACAACAACTAGTA
CTTGTTTACGATAATTATTAAACTTGTCTCGATACCTACAACATGCCCAAAAAAGTAATCAAT
ACTTTGTTTAATCACAAAGATCTCAGTGAGATGGTTCTATAGATAGTATTTCTGATTTGTTAGT
ATATTAGAGAATTGACCATCTATTAAAATAAGAGAATGAACACTATTTGATAAAACAATACAT
ACGCAATTATCATTGTCTTTAATTTTTATCAAAGTAATCATACGATCGACTATTTGTATGCAAA
AATTCATCATTTTATTTGCAAAAAAAAACAAACAAACATGGTAGAATTGTATTTGGTGTGTG
TTTTAACAAATTGTTGCAATGTTTAGTGCAACATATATAATCCACTATATATAATCATTGACATA
ATTAATATTTGATTATATGAAGCAATTTCTCTTTAATTATGTGTTTATAAATATAGGGAGAAAA
GAAAAATAGAGAGAAAGGTAAAGAGTAGAGCTAATGATTCGGGCGACGCTAAAAGGTTGA
ATGTTCGAGAGAAACGGAGTCTCAGAGACTGTGAAAACAAAAAATCAAAATCCTCAGTTA
CAGACGATTTGGTCCCCTCTCTTCTCTCTCTGCGTCCGTCTTGTGCGTCGATTTGTCTCCATC
TCCTTCTCTCTCCGCCTACTTTCTCAGGACTACTCCAATTCGCCGACTTTTTCTCCTCTGTTC
TCTGCATTTGGATTGATGCCACTTTAATACTTTGAGGTTCTCACTCTCTCTTTCTCTCTTCGCT
GACTTCGATTGCTTCAGTACCCTTTTTCTCAGGTCAGCTACGATGATAGCTTTATTCAGACAA
GAAGATCAAAGTTTTGATCTTTTTGTTTTGGTTTTCTTAAGAGATGGTTAATTAGGTCTTGAG
TGCGCTGTTTTGAATCGTTCTTCAGTGTGATTAAACAATTGGTTTTGATTTGACTAATCATGT
TGCCATTAAACTAAGTGAAAATCTCTGCTTTTTTTATATTGCATATTGATGATTGAGACATTGA
TTTCTTTTCTATTTCTTTATTTCCCAGTTTCTAATTTGGGAACTGAAAAGTTTTCTAATTTAAG
CATTACAAAGTGTACAATTTTTGTTTCTGGCAAGTTTTTCTTGGCCTAATTCGTCTATTTTATG
AATTCACCTTTTTAGGATCAAGACTTCGTAAAAGATTGTTTCTGTAAAACGCAACACCATGC
CTCTGTTTGAGCTTTTCAGGCTCACCAAAGCTAAGCTTGAATCTGCTCAAGACAGGAACCC
TTCTCCGTGAGTCCCATTCACTTGTCTCTCTTTAAGTCTGCCTTGTTTCAAGTTCATCAAATT
CATGTGTTTTTTTGTTGTGTGTGAAAAATAACAGACCTGTAGATGAAGTTGTGGAGCTGGTG
TGGGAAAATGGTCAGATATCAACTCAAAGTCAGTCAAGTAGATCGAGGAACATTCCTCCAC
CACAAGCAAACTCTTCTAGAGCTAGAGAGATTGGAAATGGCTCAAAGACGACTATGGTGGA
CGAGATCCCTATGTCAGTGCCATCACTAATGACGGGTTTGAGTCAAGACGATGACTTTGTTC
CATGGTTGAATCATCATCCCTCCCTTGATGGATATTGCTCTGATTTCTTGCGTGATGTGTCGTC
TCCTGTTACTGTCAACGAGCAAGAGAGTGATATGGCGGTAAACCAAACTGCTTTCCCGTTG
TTTCAGAGAAGAAAGGATGGCAATGAATCAGCTCCTGCTGCTTCTTCGTCGCAGTATAACG

GTTTCCAATCGCATTCTCTGTATGGAAGTGATAGAGCTAGAGATCTTCCTAGCCAACAAACC
AATCCGGATCGGTTTACTCAGACGCAGGAACCACTAATTACTAGTAACAAGCCTAGTTTGGT
CAACTTTTCACATTTCTTACGCCCTGCAACTTTTGCGAAGACTACTAATAATAACCTTCATGA
CACTAAAGAAAAGAGTCCTCAAAGCCCGCCAAATGTGTTTCAGACCAGAGTTCTTGGAGCT
AAAGACTCTGAAGATAAGGTTCTTAACGAGTCTGTTGCTTCTGCTACGCCTAAAGATAACCA
AAAGGCTTGCCTAATATCAGAGGACTCATGTAGAAAAGACCAAGAGAGTGAAAAAGCAGT
TGTATGTTCTTCTGTTGGCTCGGGTAATAGTCTCGATGGCCCATCCGAAAGTCCTTCACTTTC
TTTAAAGAGAAAGCATTCGAATATTCAAGACATTGACTGTCATAGTGAAGTGAGTTAAACTC
ATGTCATTTTATGCACATTACAATTTCGTTTCTTTGTTGTTTTCTTCAGTTTTTTTATTGAATAT
TGTTGAGCAGGATGTGGAAGAAGAATCAGGAGATGGAAGAAAGGAAGCAGGTCCATCTCG
AACGGGTTTGGGTTCAAAGAGAAGCCGCTCTGCAGAAGTGCATAATCTGTCTGAAAGGGT
GAGTGAGTGAAGCCCCTAAAGGGTTTTTGTCCATTCTCAAACTCACAAAAGATTGCTATATT
TGGTAACTTACTGTCCTCAATTTTCAGAGACGGCGTGATAGGATCAACGAGAAGATGCGTG
CCCTGCAAGAACTCATTCCAAACTGTAACAAGGTCAGCTTCTTCTCCTTTACAAATACCATT
CATGATTCATTTTTGTTTGAAAGATAAGGAGCAATGCCAAATAAACTAAAACTTGCATCTTA
GAACGAATTGCTAAAGGTTTTTGATTGGTTTCCGTATTTATTAAACCGGAAAAACAATTGAA
ACCATATTGTAAGCAAGATTGTTCAAGCCGAGAATTTTCGTATTTAATCAGCTTTTGTAACAT
TTATATGGTTTAAAGGTGGACAAAGCTTCGATGCTAGATGAAGCCATCGAGTATCTCAAGTC
ACTCCAACTTCAAGTGCAGGTAACACTCTCATTACTCTCTAGAGTCGATAAAAATCGTGCAA
ACTAATAATCTAAACACAATAATCGCCAACAGATCATGTCAATGGCGTCTGGTTACTATCTGC
CACCGGCGGTTATGTTCCCACCGGGTATGGGGCATTACCCGGCAGCAGCTGCTGCAATGGC
AATGGGTATGGGAATGCCTTATGCAATGGGCTTGCCTGATTTGAGCCGTGGTGGTTCATCGG
TTAACCACGGACCACAGTTCCAAGTCTCGGGGATGCAACAACAACCAGTGGCGATGGGTAT
TCCACGTGTCTCTGGTGGTGGTATCTTTGCCGGTTCTTCGACGATTGGCAATGGCTCGACTA
GAGATTTATCTGGTTCTAAAGATCAAACAACGACGAATAACAACAGTAACTTGAAACCAAT
AAAGAGAAAACAGGGGTCTTCTGATCAGTTTTGTGGATCGTCGTGACATAAATACAAACTT
ATGAACTGATGTATTGTTTATTTGTAGCATTCAAACAAATCTATTTTATAATCCTAAACAAAAT
TAATTTGTGTTCGTTATAACAGTGAAGAGCTTTGTGCTTATTGGTGGGTTCTGTTCTGTTTCT
TGTTTTGAATTAAAAAATGTTTTTAATATTTTCAAAGGTTTTGTTCCCTTTTTAATGTAGCGTG
GGGTTCACTATATAGATGGACCTGGTTAAACAAGCGTGATGGGCTTCTCAGCCCATTTTGTT
AAATAACTTAATTATGACA

>AtbHLH009
TAATTATTTTAAAATCATTCTATAATGATTAGAGTAAATAAACTATTAGGACTCTGAATTATAA
AATTCGATTTTATATATGCTCCTCCTTGTATCTCTTAATCATAAGTTATCATTAGCTCTGTTCAC
TAGTGCTATAAAATATATTCTGAGGTTAATAAAACTTTTTTTTCTTTTTATTTTGAAATGTCTC
CAGAGATCTGACATGGAACACCAAGGTTGGAGTTTTGAGGAGAATTATAGTTTGTCCACTA
ATAGAAGATCTATCAGGTATTATTTAATAATCTTTGATCTAATGTTTTCATTCATTAAAAAAAA
AACTTTATAATATAGTCTGTGTGTGTGTGTATTGATGTCTAGGCCACAAGATGAACTAGTGGA
GTTATTATGGCGAGATGGACAAGTGGTTCTGCAGAGCCAAACTCATAGAGAACAAACCCAA
ACCCAGAAACAAGATCATCATGAAGAAGCCCTAAGATCCAGCACCTTTCTTGAAGATCAAG
AAACTGTCTCTTGGATCCAATACCCTCCAGATGAAGACCCATTCGAACCCGACGACTTCTCC
TCCCACTTCTTCTCAACCATGGATCCCCTCCAGAGACCAACCTCAGAGACGGTTAAGCCTA
AGTCCAGTCCTGAACCTCCTCAAGTCATGGTTAAGCCTAAGGCCTGTCCTGACCCTCCTCCT

CAAGTCATGCCTCCTCCAAAATTTAGGTTAACAAATTCATCATCGGGGATTAGGGAAACAGA
AATGGAACAGTACTCGGTAACGACCGTTGGACCTAGCCATTGCGGAAGCAACCCATCACAG
AACGATCTCGATGTCTCAATGAGTCATGATCGAAGCAAAAACATAGAAGAAAAGCTTAATC
CGAACGCAAGTTCCTCATCAGGTGGCTCCTCTGGTTGCAGCTTTGGCAAAGATATCAAAGA
AATGGCTAGTGGAAGATGCATCACAACCGACCGTAAGAGAAAACGTATAAATCACACTGAC
GAATCTGTATCTCTATCAGATGTATGCTTCCTTTAATTCTAACTCATGATAATTACTTCTTGAA
AACGTAATCTTGGTCTATCTCAGAGAGTAATACATAATACATTTTGCAGGCAATCGGTAACAA
GTCGAACCAACGATCAGGATCAAACCGAAGGAGTCGAGCAGCTGAAGTTCATAATCTCTCC
GAAAGGGTACTTCATCGGTTCGTCTATATAATATATAATATATAGTTTAATGTTAGAGAATCGTA
TAACATTTATACAAAGAAAACAGATACAAAACTCTGTTTTTATTTTCTTCTTTTCATAACTTA
AATAGAGGAGGAGAGATAGGATCAATGAGAGAATGAAGGCTTTGCAAGAACTAATACCTCA
CTGCAGTAAAGTGAGTTGATACAAATCAAATCTAAAAGTGAATCAGATTAAGAAATGAAGA
TTGATTCATTCATTGGTGTGTTTTTGCAGACTGATAAAGCTTCGATTTTAGACGAAGCCATAG
ATTATTTGAAATCACTTCAGTTACAGCTTCAAGTGATGTGGATGGGGAGTGGAATGGCGGCG
GCGGCGGCTTCGGCTCCGATGATGTTCCCCGGAGTTCAACCTCAGCAGTTCATACGTCAGAT
ACAGAGCCCGGTACAGTTACCTCGATTTCCGGTTATGGATCAGTCTGCAATTCAGAACAATC
CCGGTTTAGTTTGCCAAAACCCGGTACAAAACCAGATCATCTCCGACCGGTTTGCTAGATAC
ATCGGTGGGTTCCCACACATGCAGGCCGCGACTCAGGTAAGGCCAATACGAAGCCGTGTTT
ATTAAACGACGATTTTGGTAAAATGTTTTTTGGGTTTACTACATTAATACGAGATAAGAAAAA
ATGTTAAACCGACGTAGTTTTAGTTGACACGTTGTGTGGTTTTTTATTAGATGCAGCCGATGG
AGATGTTGAGATTTAGTTCACCGGCGGGACAGCAAAGTCAACAACCGTCGTCTGTGCCGAC
GAAGACCACCGACGGTTCTCGTTTGGACCACTAGGTTGGTGAGCCACTTTTTTACTTCCTTA
TTTTTGGTATGTTTCTTTTTTATATCTATCTTTCTGAACATACTTAAAACGTTCAAGGATGTATT
ATTATAGAGTAAACGTGCAACTTCATTACGTTATTTCTGTATATGTGAGTTTATGTATGTCAA
AATGACATGATGAGATTTTTTGTAAACAACATCTTAAAAACAGGACATGTGATTTTTGTAATC
GTAAAAACTTTGGGATGCAGTTTATTTTCTAATCAAAACAAGGA

>AtbHLH010
GGGAGAGAGAGACTCTTGTTTAAAAAACTTACTTCTTCTCCTGAAAATTGAGAAGACAGAC
GTAGAGAAGAATTAATTGTAACAAAGGTTCTAATTTCCCAGCTCTGATTACTCATTATCTTA
CATCTCTTTCTATCTATTGTCAAATTCAAACCCTTAACCCAATGTTTAATCTTTTGGATCCTAA
CAAACATTTCTATGGTTTCGTTTCCAAAATTATAAAAGAGCTAACTTTTTTTGAAATTAGTTT
CAAATTTTTGGTTTTTCTTCTTCTTAAAGATTGTTGAATGTTACTTTTGATTTGCAGGTGTTAA
AGAATCATCTAGAGTACTCGTTGAACCATTTCCATGGAAGGTAACAAAATTTCATTATTTGGT
CTAATTTCTCATGAAAGTTGCTACCTTTTTCGTTCTGGAAGATCTAAATTAGTGTTTGAGTTA
CAAACGCAGAAGAAAGAGAAAGCCTTTACGAGGAAATGGGTTGTTTCGATCCCAACACTC
CAGCAGAAGTTACGGTGGAGAGTAGCTTCTCTCAAGCCGAACCACCACCACCACCACCGC
AAGTTCTGGTTGCCGGAAGCACAAGCAACAGCAACTGCAGTGTTGAGGTAGAAGAGCTCT
CTGAATTCCATCTCAGCCCACAAGATTGTCCTCAAGCTTCTTCAACACCTCTCCAGTTTCAC
ATCAATCCTCCTCCTCCACCACCTCCTCCTTGTGATCAACTCCACAACAATTTGATTCACCA
AATGGCTTCACATCAACAACAACACTCTAATTGGGATAATGGGTATCAGGATTTTGTGAATT
TGGGACCTAACTCTGCAACAACTCCTGATTTGCTTAGTCTCTTACACTTGCCTAGATGCTCAT
TGCCTCCTAATCATCATCCTTCCTCTATGCTACCTACCTCTTTCTCAGACATTATGTCTTCTTC
TTCTGCTGCTGCTGTTATGTACGACCCTCTCTTCCATTTGAATTTCCCTATGCAGCCTAGAGA

TCAGAACCAGCTAAGAAACGGCTCTTGTTTACTTGGAGTTGAAGATCAAATTCAGATGGAT
GCTAATGGAGGAATGAATGTTTTGTACTTTGAAGGAGCAAACAACAACAATGGTGGATTTG
AGAATGAGATTCTTGAGTTTAACAATGGAGTTACTCGTAAAGGAAGAGGATCAAGAAAGTC
GAGAACTTCCCCTACAGAACGTGAGAGACGAGTTCACTTCAATGATCGTTTCTTCGACTTA
AAGAACCTCATTCCAAATCCCACAAAGGTATATACTTTACTTTGCTTTGATTTCAATTCATGT
TGTTAATGTGTATGAGGAGACTATGAAATGAGAGACATTGTTGGTTTAATCTCTGTAGATTGA
TAGAGCATCGATAGTTGGAGAGGCGATAGATTACATCAAAGAGCTTTTAAGGACAATAGAG
GAGTTTAAGATGCTTGTGGAGAAGAAGAGATGTGGGAGATTCAGGAGCAAGAAGAGAGCT
AGAGTTGGTGAAGGAGGAGGAGGAGAAGATCAAGAAGAAGAAGACACTGTGAATTA
CAAGCCACAGAGCGAAGTAGACCAGTCTTGCTTCAACAAGAACAACAACAACTCACTGAG
ATGTTCATGGCTTAAGAGGAAATCGAAAGTCACTGAGGTCGATGTACGTATAATCGATGATG
AAGTAACCATCAAGCTTGTTCAGAAGAAGAAGATTAACTGTTTGTTGTTCACCACCAAAGT
TCTTGATCAGCTTCAGCTAGATCTTCACCATGTTGCAGGTGGACAGATTGGTGAACATTACA
GCTTCTTGTTCAACACTAAGGTAATGAAAATGTCTCACTAATATAGTTCTGTTATTAACTCCA
AACCAATAAACTAATCTTTTTCAATTTGGTTTGAGGCAGATTTGTGAAGGATCTTGTGTGTAT
GCAAGTGGAATTGCAGACACATTGATGGAGGTCGTGGAGAAGCAGTACATGGAAGCTGTT
CCTAGCAACGGCTACTAGATTCCAGACACACTTATGGAGTAGTTTATTGCTTTTTAATAACTT
TTGTGATACCCCTCTAATGACTGATTAAGTTATTAATCACTTTGGTAATTTTCAAGTTTATTTA
GCTAGAGTTAAAGTTAGAGCTAGAGCTGAGGCTCTTTGTCTGGATTTTGTCTAACTCTATATA
TTTCTTCTAGCTTTATCGGTTTCGTTTTTCGACCAC

>AtbHLH011
GTGGGGAGCACAAGTTATTGATTTGCCTCGTCGGCGTCCACCACCATTATCGCCGGATTTTT
ATATATTCACGTAGAAGAAGAATGAGAAGACGAAAACCAACTTCTACTTCTTCTCAGTGGCT
CTCTCTTTATCTTTCTTGGAGTTTAGTTAGAGATTTTAACGTTGCAAATGGATCAACCAATGA
AACCAAAAACTTGCTCTGAATCTGATTTTGCTGATGATTCCTCTGCTTCTTCTTCTTCTTCTT
CGGGACAAAATCTCAGGTTTTAGTGCTTAAAGATGTTAACTTTACATGGCTGTGTCATGTTTA
TTCATAGTTTCGTCTAATTACAGAGGAGCTGAGATGGTGGTGGAAGTGAAGAAGGAAGCAG
TTTGTTCCCAGAAAGCAGAGCGAGAGAAGCTTCGTAGAGATAAGCTTAAGGAACAGTTTCT
TGAGCTTGGAAATGCACTTGGTATATATCTTTTCACAAGATGAATACAGATTTTCAAAATTTT
CTTTGAATTGAGTTTAGAGTAATGGTTTCTTTCTACAGATCCGAATAGGCCTAAGAGTGACA
AAGCCTCAGTTCTCACTGATACAATACAAATGCTCAAGGATGTAATGAACCAAGTTGATAGA
CTAAAAGCTGAGTATGAAACACTATCTCAAGAGTCTCGTGAGGTGATTTGATTAACTTTGTC
CATTCTCTTATTGGATTTATAGTTGAATATGCTCTGCTCAAGTGCTCATCTTCCAGTTCGTTTG
ATCAGCTAATTCAAGAGAAGAGTGAGCTGAGAGAGGAGAAAGCGACTTTAAAGTCTGATAT
CGAGATTCTTAATGCTCAATATCAGCATAGAATCAAAACCATGGTTCCATGGGTACCTCATTA
CAGTTATCATATCCCCTTCGTAGCCATAACTCAGGGTCAGTCCAGTTTTATACCTTATTCAGC
CTCTGTCAATCCTCTAACCGAACAACAAGCATCGGTTCAGCAGCATTCTTCTTCTTCTGCCG
ATGCTTCAATGAAACAAGATTCCAAAATCAAGCCGTTAGATTTGGATCTGATGATGAACAGT
AACCATTCAGGTCAAGGAAATGATCAAAAAGATGATGTTCGTTTAAAGCTCGAGCTTAAAA
TCCATGCCTCTTCTTTAGCTCAACAGGTGAGTGATCTCTTCAATAGTTTTGCCAACAAGCTC
TTTCATGGACTGACCAGAGTTTACTTCCATGCAGGATGTTTCTGGAAAAGAGAAGAAAGTA
AGCTTGACAACCACTGCAAGCTCATCGAATAGTTACTCATTATCTCAAGCTGTTCAAGATAG
TTCCCCCGGTACCGTAAATGACATGTTGAAGCCATAAACCAATAAACATATTCCCCTGAACT

TGTGTTTAATACCGTGATTGAGAAGGTACCATGATTAAACTTGTTGTAGATTATCCACATGAT
TAACGATGTATTCTTATCACAAGCAAATAAAACACAAAAGCATTTGCTTATATCTCTTTGCAA
CTGAACTATTGTCTATGACCGATGTTATAAGACTTGAGAAACATATTCAAACCTAACGGCAG
AT

>AtbHLH012
AAACCCTTCTTTAATAAATTTTGCCTTTTGGTTGTCTCCTCTTTATCAATCACTTTGGTTCCAT
ACTTTTAAATTCAGTTCTTCACGATATCTAGATATGTCTTTGACAATGGCTGATGGTGTAGAA
GCTGCAGCAGGAAGAAGTAAAAGACAAAACAGCTTATTAAGAAAACAACTTGCTTTAGCT
GTAAGAAGTGTTCAATGGAGCTACGCAATCTTCTGGTCGTCTTCACTTACTCAACCTGGGTA
AGTATAATCTAATATTCATCATGTACCAAAATTATCAGATCTTGTTGTTCTCATCTTTGCATTTC
CATTTTCTCTTCTTTTAAATCTTGGGTTACTTATGAGTACTATGTTCTACTAATTTAGGGTTTT
GGAGTGGGGAGAAGGATGTTACAATGGAGATATGAAGAAGAGGAAGAAGAGTTATGAATC
TCATTATAAATATGGGTTGCAAAAAAGCAAGGAGCTTCGGAAACTTTATTTGTCTATGCTTG
AAGGAGACAGTGGTACTACTGTTAGTACTACTCATGATAATCTCAATGATGATGATGATAATT
GTCACAGTACAAGTATGATGCTGTCACCAGATGACCTCTCTGATGAAGAGTGGTACTATTTA
GTCTCCATGTCCTATGTCTTCTCTCCTTCACAATGGTTTGTTCTCTCACTCTCTCTCTCTCTTC
TCCTATGAATTTGTACACTAAAATATATTAGCTCTTGTGAACTATTCATATAGAAAAACCAATT
TGCAATAAAGAAGTAATGAGAAATGTCCTTTCATTTTAAGATGAAACATCCATCACTTTGGG
TAAAGAGTTTAGTACCACCAAAACCATTTGGTAAATAGTGGAGTGATTATTCCTTTGGGGTT
CCTTTTTGTTACACTTTCACCTTTTGCATTTTAAGTAATCCTAGATTAAGTTTTTTTTTTCTCTT
TTATGATCATTAATTATTGTCATTTTGGCTTTTTTGGTTGTTTATGTATATACTTTAGTTTGCCT
GGAAGAGCTTCAGCGACGGGTGAGACCATATGGCTCTGCAACGCTCAATATGCGGAGAACA
AGCTCTTCTCTCGTTCTTTGTTAGCAAGAGTAAGTTCTCAGAAACTTCTAATTTCCTCTTCTT
GAATCTTGATATATCAAGATCGAATCTGACTAAAAATCTCCTTTGATCTTCTTTGCTTTTCATT
ACTGTTCATATGGCTTCTCTCCTTTAGAGCGCATCAATTCAGGTACGTTATAATGTTTGTTATA
ACTGGATTCTGATTGTTAGAAATAAAAGTTTAGTTCTCTTATTGTTAGAAATGAAAGTTTAGT
TATCTATATATCAATTTGGATAGAAGATTTATAGAATGTATCTGTGTTGTTATCTCCAGACTGTT
GTGTGTTTCCCTTACTTGGGCGGAGTCATTGAGCTGGGCGTCACTGAATTGGTAACTCTCCA
TCTTAATATATAAAGTTTACTTTTGTTGCTTGCTTCACAAGAATTAAATATACCTTACTTTGT
TTAATTTTGTTCATGATCAATGAAAGATTTCAGAAGACCATAACCTGCTTCGAAACATCAAA
TCTTGCTTGATGGAAATATCTGCACACCAAGACAACGATGACGAGAAGAAGATGGAGATTA
AGATCAGTGAAGAGAAGCATCAGCTTCCATTAGGTATTTCTGATGAAGACTTGCATTACAAA
AGAACCATTTCAACAGTACTCAACTACTCCGCAGATAGATCAGGTAAGAACGATAAGAACA
TTCGTCATCGTCAGCCAAATATTGTTACTTCTGAACCTGGCTCAAGTTTCTTGCGGTGGAAG
CAATGTGAGCAGCAAGTCTCGGGTTTTGTTCAGAAAAAAAAGTCACAGAATGTGTTGCGG
AAGATATTGCATGATGTCCCTTTGATGCACACAAAGAGAATGTTCCCAAGTCAGAACTCTGG
TCTGAATCAAGATGATCCTTCAGATAGAAGAAAAGAGAACGAAAAGTTCAGTGTCCTTAGA
ACTATGGTTCCCACTGTCAACGAGGTAATAACTCTTTTTCTAAATCCTTAGAGACAAGACTG
TGTTTCAGAAGCCAACACTCTGTTTTGAATCTTTGTGCAGGTTGATAAAGAATCGATACTAA
ACAACACAATCAAGTACCTGCAAGAACTGGAGGCAAGAGTAGAAGAGCTAGAATCTTGTAT
GGGATCAGTTAATTTTGTAGAAAGACAAAGAAAGACGACAGAGAACCTTAACGACTCTGT
GTTGATCGAAGAGACATCAGGGAACTACGATGATAGCACGAAGATCGATGACAATTCAGGA
GAAACCGAACAAGTCACTGTTTTCAGAGATAAGACACATTTGAGAGTTAAACTCAAAGAA

ACAGAAGTTGTGATCGAAGTAAGATGTTCTTACAGAGACTACATAGTTGCGGACATCATGG
AAACTCTGAGCAATCTTCACATGGATGCTTTCTCTGTTAGATCTCACACGCTCAATAAGTTC
CTCACATTGAATCTCAAGGCCAAGGTAAATTAATCTTACTGATGTTTGGAAAAGGCAGATTT
GGGTTTTCTTGACTTACACGTAACGTTTTTGGGTTTATGCATGCAGTTTCGCGGGGCTGCAG
TTGCGTCCGTAGGAATGATTAAGCGAGAGCTGAGAAGAGTCATTGGTGATTTGTTTTAATGT
TCTTCTTAGTAAAAATTCTTATATTTTCTTAAACTTTTGATGAAATGTTATATTTATCTAGACTT
TCGTGAACCGATATGCGATGTGCCATTATCTTTACATCAAGTTTTCAGGGTTTTTGTATGTAA
AGTTTGCCAAAGTTTGGTTGGAATTTTCGACAACGTTGTCTCTTCCTCTTCTACAAAACCAA
GATCTATACTTATTCATAATTCATGGGCGATTTGTATCTTTCATTAATGATTGTTTTGAGCTATT
ATATTATTTGTATTGATAGAAAAAAATAACCAATTG

>AtbHLH013
ATGAAAGTCTTTTTGTTTTGTTTTTTGTTTTGTTTGTTGGGGTTTGGTCTTTTAATAAAAAAG
AGCACGAAGAACATGCGTCGGACTCTCTCTCTTCCTTGAATTCATTCAATTGGCCACGGCGA
CAAAACAGGGAGCCGATACAGCGAAACTGATACATCTTTTTTATTTTCTGGATTCTTAGGG
TTCTTTTTCGGGGAATTTAGGTAATTTGTTTGTCCATTTTGGTGGGGTTTGGGAGATTTAAAA
TTTGTGTTTTTTTTTGGGTCGGATTCTCAAAAATCTGATTTTGAGGAGAAAATCTCGGTGGA
AGAGACTTGATTCAAATCTTCTTTACTGGTAAAAATATATTCTTCTCAATTCCTCATTTTCTGT
TGGTGGAGTTTGATTTTTGTCATTTAATTGTTGTTAAAGAAAATAGGGGCAAGATCTGAGAT
CTCTGGGTGTTCCATGCAAAACTGTTATACTCCGCCGTATATTTGCATCTGGGTCACACTTCT
AAACCCTATCATTTGACTTAAGAATCGATTTCTGAAGATCTGGGTTTCTTAAAAGTCTTTCCT
TTTTGTTGGGATCCAAACAACAAAAGAAAAGAAAGCAGAGATGAATATTGGTCGCCTAGTG
TGGAACGAGGACGATAAAGCGATTGTTGCGTCATTACTGGGCAAACGAGCTCTCGATTACT
TGCTTTCCAACTCTGTTTCCAATGCTAATCTCTTGATGACTCTAGGAAGCGACGAGAATCTG
CAGAACAAGCTCTCGGATCTCGTCGAGAGACCCAACGCTTCTAATTTCTCTTGGAACTACG
CCATTTTCTGGCAGATTTCCAGGTCAAAGGCCGGAGATTTGGTTCTCTGTTGGGGCGATGGA
TATTGCAGAGAGCCTAAAGAAGGAGAGAAATCAGAGATCGTTAGGATTCTAAGTATGGGAA
GAGAAGAAGAAACGCATCAGACTATGAGAAAGAGAGTATTGCAAAAGCTTCATGATTTGTT
TGGTGGCTCAGAAGAAGAGAACTGTGCTTTAGGACTAGATAGAGTTACTGACACTGAGATG
TTTCTTCTTTCTTCTATGTATTTCTCATTCCCTCGAGGTGAAGGTGGTCCAGGCAAGTGTTTT
GCCTCTGCTAAACCTGTTTGGTTATCCGATGTCGTCAATTCGGGTTCTGATTATTGCGTTAGA
TCGTTTCTTGCTAAATCTGCTGGCATTCAGACTGTTGTTTTGGTTCCTACTGATCTTGGTGTT
GTTGAGTTAGGCTCAACTTCATGTTTGCCTGAAAGTGAAGACTCAATCTTGTCTATAAGATC
ATTGTTTACCAGTAGTTTGCCTCCGGTTAGAGCTGTTGCTTTACCTGTTACTGTAGCTGAAAA
GATTGATGATAACAGAACGAAGATATTCGGCAAGGATTTGCACAATTCAGGGTTTCTTCAAC
ATCATCAGCATCACCAACAACAACAACAACAACCACCGCAACAGCAACAGCATAGACAGT
TTAGAGAGAAACTCACGGTTAGAAAAATGGATGATAGAGCTCCCAAGAGATTAGATGCTTA
TCCGAATAATGGGAACAGGTTTATGTTTTCGAACCCAGGCACCAACAACAACACTCTTCTG
AGTCCTACTTGGGTTCAACCTGAGAACTACACGAGGCCAATCAACGTGAAGGAAGTTCCA
AGCACGGATGAGTTCAAGTTTTTACCTTTGCAGCAATCATCGCAGAGGCTGCTTCCTCCTGC
TCAAATGCAGATAGATTTCTCTGCTGCGAGTTCAAGGGCTTCAGAGAATAATTCAGATGGAG
AAGGAGGAGGGGAATGGGCAGATGCAGTGGGTGCTGATGAATCTGGTAACAACAGACCAA
GAAAACGCGGAAGGAGACCTGCCAACGGAAGAGCGGAAGCTTTGAACCATGTAGAAGCA
GAGAGGCAGAGGCGTGAGAAGCTTAACCAGAGGTTTTACGCTCTGAGATCCGTTGTTCCCA

ACATATCCAAGATGGACAAAGCGTCCTTGTTGGGAGACGCGGTTTCTTATATAAACGAGCTT
CACGCGAAGCTAAAGGTCATGGAAGCAGAGAGAGAGAGGTTAGGGTATAGCTCAAATCCA
CCTATCAGCTTGGATTCAGATATAAATGTTCAAACCTCAGGTGAAGATGTCACAGTGAGGAT
AAACTGTCCGTTGGAGTCTCATCCGGCTTCCAGAATCTTCCATGCGTTTGAAGAGAGTAAA
GTAGAAGTGATAAACTCGAACCTGGAAGTTTCTCAGGACACAGTGTTGCATACGTTTGTAG
TCAAATCTGAAGAATTAACGAAAGAGAAGCTGATATCGGCCTTGTCTAGAGAGCAAACAAA
TTCAGTTCAGTCAAGAACATCATCAGGTAGATAGCCTTTTGAGAGACAGAAGAAGGTGGAA
GTAAGTTTGTAATCAAGAACCGGTTTTGAATGGTTCTTTTTTTCTTCTAGGTATCTTTTGTTTT
TACCTTTTTTTGGTTATATACCTGTAAGAATCCTCCAAACTAGGATTTTAGAAGTTTTATTGAC
AAAAGTAGACATTATCATTTACTCGTTTTACAGTGGACAAAGCTGGGAATTAAGTAATTAAC
GATAATGTTGTATCTGTTAAAGTCGTAGACTAGCTCATGAATCAGTCTTTATTCATTCTGATAT
GTCCTATGTGTTGGATTTGGTGAAGTGTTTAATCGACAAGTCAGATAAGTG

>AtbHLH014
CTTATTCATCCTTTTCCACTTGTCTTCTCTAACCCTCTAAATTCTCTTTGGTCGGGATCACTGG
AAAGAAATGTATAATCTCACTTTCTCTCCATCATTGTCCTCTTCTTTGCTCTCTTTTACACAAC
AAACTCCTGCAGCCATAGTGTCCTCTTCTCCTCCGGATTTAGTACTTCAACAAAAACTCCGG
TTTGTCGTGGAAACCTCACCGGACCGATGGGCTTATGTCATTTTTTGGCAAAAGATGTTTGA
TGATCAATCGGACCGGTCTTACTTGGTTTGGGTTGATGGTCATTTCTGTGGAAACAAGAACA
ACAATTCCCAAGAAAATGTTCATTTTTCTTTGCCTCTTATGTTATCTAATCAAAATATCCAAAA
TTTATTTAAATCAACTTCTTAAAAAAGTTCTATTAAGAAAATAATATAATTTTCCTTACCCTTG
ATTTTTTGTTTTTGTTTCTTTCTTAAGTATACAACAAACAGCATCGAGTGTGAGCTGATGATG
GACGGTGGAGATGATCTGGAGTTGTTCTATGCCGCATCGTTTTACGGTGAAGATAGATCGCC
GAGAAAGGAAGTATCCGATGAATCTTTGGTTTGGTTAACCGGTCCCGACGAGCTCCGGTTT
AGCAACTACGAGAGGGCTAAAGAAGCCGGTTTTCACGGGGTACACACATTGGTCTCCATAC
CCATCAACAATGGCATTATCGAACTTGGTTCGTCTGAATCAATTATACAAAACCGGAACTTTA
TAAACCGAGTCAAGTCCATATTCGGGTCGGGTAAAACAACAAAACACACTAACCAAACCG
GTTCTTACCCTAAACCGGCGGTATCAGATCATTCGAAAAGCGGAAATCAGCAATTCGGGTC
GGAAAGAAAACGGAGGAGGAAACTTGAGACGACAAGGGTAGCTGCGGCGACGAAAGAG
AAACATCATCCGGCGGTTTTGTCCCACGTGGAAGCGGAGAAGCAACGGAGAGAGAAGCTA
AACCACCGGTTTTACGCTCTACGTGCCATCGTACCAAAAGTGTCACGAATGGACAAAGCGT
CACTTTTATCCGACGCAGTTTCGTACATAGAAAGTCTTAAATCCAAAATCGATGATCTCGAA
ACTGAGATCAAGAAAATGAAGATGACGGAGACAGATAAATTAGACAACAGCAGCAGCAAT
ACGTCTCCGTCTTCCGTTGAGTACCAAGTTAACCAGAAACCTTCAAAATCAAACAGAGGCT
CTGACTTGGAAGTGCAAGTGAAGATCGTCGGAGAAGAAGCCATAATTAGGGTTCAGACAG
AGAATGTGAACCATCCTACGTCGGCATTGATGAGTGCGTTAATGGAAATGGATTGTCGTGTA
CAACACGCGAACGCGTCGCGCTTGAGTCAGGTTATGGTTCAAGATGTTGTTGTTTTGGTTCC
AGAAGGATTAAGATCGGAAGATAGGCTTAGGACCACACTTGTCCGAACCCTAAGCCTATAA
CAATTTCTTTGTTTTGTGACGTCAACATAGGATGTGATAATAATTTTTGTTTTTTTTTGCGACC
TTTTGTTTTATTTGATAAGTACTAGAAAAATCAAATTCCTTCTAGCGATTAATGTTGTTTTATC
AAAAGTTTGATCTCGTTGATACGTAGTTTATGTGTTACTTTTTTACTTTATTTCTCGTACGTAC
GTACTAGCTCGCTAAAAGCGCAAAGTGTTTGGTATATTGTCTTACTAAAACAATATAGAGAC
TGTATATTTTAAAAGAAACAACTCATTAATTGATCATTTGGAA

>AtbHLH015
TAAAGTTAGTTGACACAATAGTAAATGCGATACATGCTAGAGTCACGGAAGAGGCAAAAAC
AAACTCCAAAGAGAGACATATAAGAAAAAGACAAAAACCCTTAAGAAGCTTATCACATTCT
TAAAGCACGAAAATTCAGAATAAGATTCAGTCATCCTTTAGCACAAAAAAAAAAAAAAAA
AGATCCAGTCATCCAATACTAGTTCCCCAGAAATAAAGGTGATATATTTTTTTTTTGAGAAG
CCTATCCAGTACCAACCTCAATTCTCTAGGTTAGATATAGTCTGGGTAGTATATATGTTTCTTC
ACTGATTAGTAATCTCTGTGTGTAGTACACTTACTCACTAGTGCTACTCTTTTGGATCTTTCT
GGGGTTTTTCTTTTGGTTATAAAACCAGAAGAAGAAGATAACTGTTTGTTATTTTAATACTTA
CCATTATCATCATCTTTGTAGAGAGAGAGATATGCATCATTTTGTCCCTGACTTCGATACCGAT
GATGATTATGTCAACAACCATAATTCTTCTTTGAATCATCTTCCTAGAAAATCCATTACTACTA
TGTAAGTTCTTTTATTCAAACTTATATTAAAAAAAAAAAAAAAAGTTTGATTAGTTCAAGATCTG
AGTATTTATAGTATTGAAAACTTTAGGGGTGAAGATGATGATCTTATGGAGCTTTTATGGCAG
AACGGTCAAGTTGTTGTTCAAAACCAGAGACTTCACACCAAGAAACCTTCTTCTTCTCCAC
CGAAGCTTCTTCCTTCTATGGATCCTCAGCAGCAACCTTCTTCAGATCAGAATCTTTTTATTC
AAGAAGATGAAATGACTTCTTGGCTTCATTATCCTCTCCGTGACGATGATTTCTGCTCAGATC
TTCTCTTCTCCGCCGCACCTACTGCGACGGCTACCGCGACGGTGAGTCAAGTCACCGCCGC
GAGACCGCCAGTATCTTCGACGAATGAGTCGAGGCCGCCGGTGAGGAACTTCATGAATTTC
TCGAGGCTGAGAGGGGATTTTAATAACGGTAGAGGTGGTGAATCTGGACCGTTGCTTTCGA
AGGCGGTTGTGAGAGAATCTACGCAGGTAAGTCCTAGCGCAACACCGTCGGCGGCGGCGA
GTGAATCCGGTTTAACACGGCGGACGGATGGTACTGACAGTTCCGCCGTAGCTGGAGGCGG
CGCGTATAATCGGAAGGGAAAAGCAGTGGCTATGACGGCGCCGGCGATCGAGATAACCGGT
ACATCGTCATCTGTAGTGTCAAAGAGCGAAATCGAACCGGAGAAGACGAACGTCGATGATA
GGAAACGAAAAGAGAGAGAAGCCACCACTACTGATGAAACTGAATCCCGTAGCGAGGTGA
GTCAAAAAAACACAGAACAGAGTCGTTGTTAGCTATCGTGCGCAAGTCAAATACGGCGCAT
TTTAGTCTGACTTTGTAGTTTTCAGCGCATTTTTAATTAACTTTTTTGGTGTTTTCTTGTGGTG
CAGGAAACAAAACAAGCACGTGTATCAACAACATCTACCAAGAGATCTCGTGCTGCTGAA
GTTCATAATCTCTCTGAAAGAGTACGTTTGTGTTTTTTCTTATGTTTTTGTAATCTTTTTCGAT
AAAGATTTCGCAATGAATCTGAAGAATGTCGCGGATTTGGTAACAGAAACGGAGAGATAGG
ATCAATGAGAGAATGAAAGCTTTGCAAGAACTTATACCTCGCTGCAACAAGGTCTTCGACG
ATCTTTTTTCTTTCTCTTCAAGTTATTGTATACGTTAACCTTGTTACTGATTGTTGTTTCTTTGA
TCCTCAGTCAGATAAAGCTTCGATGCTAGATGAAGCTATTGAGTACATGAAATCTCTTCAGC
TTCAAATACAGGTTCATTTTCTTTAAAGGATTCTAAGTCTTGAAAGCCCTTTTTAAGTTCTTG
AAAATGTACACTTTACCATGAAAACTCCAGTTTATATGACTGAGATTAATGCAGATGATGTCA
ATGGGATGTGGAATGATGCCAATGATGTATCCGGGCATGCAACAGTACATGCCTCATATGGC
GATGGGTATGGGTATGAACCAGCCTATTCCTCCTCCTTCCTTCATGCCATTCCCCAACATGTT
AGCCGCTCAAAGACCTTTGCCTACACAAACTCACATGGCCGGGTCAGGACCGCAATACCCT
GTTCATGCTTCTGACCCGTCAAGAGTCTTTGTACCGAACCAGCAGTATGATCCAACCTCGGG
CCAGCCTCAGTATCCAGCTGGTTACACGGATCCATATCAGCAGTTCCGCGGTCTCCACCCGA
CCCAACCACCTCAGTTTCAGGTACTAAACAGTCATCTCTTTCCCAAAAATGACTAGTTCCTC
TGCTTACAAGTCACAAGTTCTTATCTTTCTTATTTTAGAATCAAGCAACATCGTACCCAAGTT
CGAGCAGGGTGAGTAGTAGTAAGGAATCTGAGGATCACGGAAACCACACAACAGGTTAAT
AATGTCCATGGAGCAACAAGAAGATCTGTTTTCACAAGCAAACACAATTTCTTATCCGACC
CGACCCAACCACCTCAGTTTCAGGTACTAAACAGTCATCTTTTTCCCAAAAATGGCTAGTTC
CTCTGCTTACAAGTCACAAGTTCTTATCTTTCTTATTTTAGAATCAAGCAACATCGTATCCAA

GTTCGAGCAGGGTGAGTAGTAGTAAGGAATCTGAGGATCACGGAAACCACACAACAGGTT
AATAATGTCCATGGAGCAACAAGAAGATCTGTTTTCACAAGCAAACACAATTTTGAGAAAT
TGACAGAGAGACCTAACATGTATATATATCGCCATCTGTTTCTTGTTTTTCTTTGGTTTGTTTT
GTCCTCTCTTCTCAGGTTGTATACTTAGAGAGCTGTACATGTAATGATCCAGAGATCTAGGAA
TCAATACATAGAGGTTGCAGAGGCTTAGTGCAAAGAATAAATATGAGAAAGTTAGGTTCATA
TACA

>AtbHLH016
TAAGCTGTCAACAAAACATTCAACAAGTCTTAAGCAAGCCATTAAAGAAGGGACCTGAAC
AATTTAATATATATGGTTAACTATTTAAGTATCATGTCCCCACTTGTCCATTACGTGATTACTAC
AAAAGTTCACATAAAAGTTCCTTTTTATATATATAGGAAAGGGAAGAAGATCTCAATAACAC
AAAACCTTTTAAACTAGTAAAATACACAGATTTTAGGATGAGCCAATGTGTTCCAAACTGTC
ACATCGATGATACTCCGGCAGCAGCCACCACCACCGTCCGCTCCACCACAGCCGCAGACAT
CCCCATGTACATACCTTAATTTATCATCTTGATAGTATAAGTGGCCACAATATAATGATAGCTT
TTCTTGTTTGTCTACAGATTAGACTACGAGGTAGCCGAGCTGACGTGGGAGAACGGGCAAC
TAGGCTTGCACGGCTTAGGTCCACCGCGAGTGACGGCTTCGTCGACCAAGTACTCCACAGG
CGCCGGTGGAACGTTGGAGTCGATAGTGGACCAAGCTACTCGCCTCCCTAACCCTAAGCCC
ACGGATGAGCTCGTCCCGTGGTTCCATCATCGCTCCTCCAGGGCCGCGATGGCAATGGACG
CGCTTGTCCCTTGCTCCAACCTAGTACACGAGCAGCAGAGCAAGCCTGGTGGCGTTGGCTC
CACCCGGGTGGGGTCATGTAGCGATGGTCGTACCATGGGCGGTGGAAAACGAGCAAGAGT
GGCACCGGAGTGGAGCGGCGGCGGGAGTCAGCGGCTGACCATGGACACTTACGACGTAGG
TTTCACCTCAACATCAATGGGCTCGCACGATAACACAATCGACGATCATGACTCCGTCTGCC
ACAGCCGCCCACAGGCAAGTCATCATATATATTAACTTGTTCTAGTTATTGATGTCTCTAGCT
TTATAAGTAAATGATAATAATTAAGGAAAAACGATAGATGGAGGACGAAGAAGAGAAGAAA
GCCGGAGGAAAATCATCAGTTTCAACCAAGAGAAGCAGAGCTGCTGCTATTCATAACCAAT
CCGAACGTGTAATGATTCACTCAACCATTATTTTATTTTTCAAAAAAAGTTTTATGTTATTCGT
CTACTGTTACACATGCATGACCCCCTGCGTTTTATCCTTGGTTGATTTTATTACAGAAGAGGA
GAGATAAAATCAATCAAAGGATGAAGACTTTGCAAAAACTGGTTCCCAATTCCAGCAAGGT
ACGCAAGTCGTTTTACATAAACTAGTGTACGTGGCTAAATAAAAGTCATATGCGTACGTATA
GTAAAAAAATAGATGCTAGCTAGGTATACATGAAAATAGAGGGGATAATATGAAAAAGGGTG
GTGGACCAAAGTGAAGTGAATGCATCTAAAGAAATGGACCTCATACGATGATTCTACTCCA
CACACTTATGTTCATCAATCTCTTATTTGCTTGTCCCCTAAAAACTTATTTACATCACTTTATT
ACTTGGTCCGGTTGGTCCATTCATATACACCTTTAATTATTATATGCCTTGTGGCTGTCGACTC
AAAAATCAACACAATCTACATTACACACATGCTCCGTAACATTGGTTTGGTTAATTATAGTAG
ATACCAAAGAGGCTTGATATATAAATCGTTAATTTTGGAACTATAGACGGATAAAGCATCTAT
GTTGGATGAAGTGATAGAGTATTTGAAGCAACTTCAAGCACAAGTGAGCATGATGAGCAGA
ATGAATATGCCTTCTATGATGCTTCCTATGGCCATGCAGCAACAACAACAACTACAAATGTCT
CTCATGTCCAATCCCATGGGTTTAGGGATGGGCATGGGGATGCCCGGTCTCGGTCTCCTCGA
CCTTAATTCTATGAACCGAGCTGCTGCAAGCGCTCCTAATATCCATGCCAACATGATGCCAA
ACCCATTTTTGCCCATGAATTGTCCATCGTGGGATGCTTCTTCCAATGACTCTCGATTTCAGT
CTCCTCTCATCCCCGATCCTATGTCTGCCTTTCTTGCATGCTCTACTCAGGTTGACCCTTTTTC
TATATATAGATATACAACTTGGGTCTAAACAATGCTTCAGTACCATTTATATTTTTATATATTTG
GAATTGCAGCCAACGACGATGGAAGCGTATAGCAGGATGGCTACATTATATCAGCAAATGCA
ACAACAACTTCCTCCTCCTTCGAATCCAAAATGATTATTACTCAAACACCTCTATATAGTTTA

CGTCTATATATGTGTTAGTCACATACATACATATATATATTCCATCATAATTATTTATTTATATGTA
TAGGCTTCTCATGAATTATGATATTATACGTATTACGTACTTTAAAAATATGTGGTCGTAATTG
GATAGAGAAAAATAAATAATGGTTATGTTTGAGTGAATTAGTAACATCACTCCACGTCTCCA
CCACATCCACAAATGAACTCCAAATTTTCTAGATCAATATACGAACAGTAGATAATCCTTCGT
TTTCACGAATTTAATTATGTGACTGAGGATAAGTGAAAAGTGGAAATGGA

>AtbHLH017
GCATAAGTAACCATGGTTGGGTTTTTAAAAAGAGCTTTCATACAAACTTCAATTACCCCAGC
AAGTCTTGTCTCCTTCTCTCTCTCTCTCCCTCTCTAACTGTATCTTCTTCTTCTTCTTCAACCT
TCAGACTTTTCTAGTTGCAACTCTCATTCAATTGTTCAATTTCTCTGTAAAAATCAAAGCTTT
TTGGAATCTTTCTTCGGCGAGATATTTCTTCACCGGCGCAAGCTTCTCTTGAGCGTGATTCAT
CACTGGGTTTGCATTTTTTCCTGGTGGGTATTCTTCAAATTGTTCAAAGCTCCGACCTTTTCT
TCCTTAGAATCTGATTACTGGAATCTTGAAGCTGGAGAGACTCGAGTTTATATGGTTTCAAG
CATCGTTGAGTCAATCATTGAGTTGTGTAGAAGAAAGGTTTGGTTTTCTTCTCCGTTTAAGT
GGTTTTGGGTTTGGTAGGATTTGGTGTAATCTGGGAGATAAAGAAAGATGAATATGAGTGAT
TTAGGTTGGGATGATGAAGATAAATCGGTGGTTAGTGCTGTTTTAGGGCATTTAGCTTCTGAT
TTTCTTCGAGCAAACTCTAATTCGAATCAGAATCTCTTTCTTGTTATGGGAACTGATGATACT
CTGAATAAGAAGCTCTCTAGTCTCGTTGATTGGCCAAACTCGGAGAATTTCAGCTGGAACT
ACGCTATTTTCTGGCAACAAACCATGTCTAGATCCGGACAACAAGTCTTAGGTTGGGGAGA
TGGGTGTTGTCGAGAGCCTAATGAGGAAGAGGAATCAAAAGTTGTTAGGTCTTATAATTTTA
ACAACATGGGGGCAGAGGAAGAGACATGGCAAGATATGAGGAAGAGAGTGTTGCAGAAG
CTTCATAGGTTGTTTGGTGGATCTGATGAAGACAATTATGCTTTGAGCTTAGAGAAAGTTAC
TGCTACTGAGATTTTCTTCTTAGCTTCCATGTATTTCTTCTTCAATCACGGTGAAGGCGGTCC
TGGGAGGTGTTATTCTTCAGGGAAACATGTGTGGCTCTCTGATGCAGTTAACTCTGAGTCTG
ACTATTGTTTCAGGTCTTTTATGGCGAAATCTGCGGGAATCAGAACGATCGTTATGGTTCCTA
CTGATGCTGGTGTTCTTGAGCTTGGTTCTGTTTGGTCTTTGCCTGAAAACATTGGCTTGGTT
AAGTCTGTTCAAGCTTTGTTCATGAGGAGAGTTACGCAACCAGTAATGGTGACTTCAAACA
CTAACATGACTGGAGGGATTCACAAGCTTTTCGGGCAGGATTTGAGTGGAGCTCACGCGTA
TCCTAAGAAGCTCGAAGTGAGAAGAAACTTGGATGAGAGATTCACTCCTCAAAGTTGGGA
AGGCTATAATAACAATAAAGGTCCAACATTTGGTTACACACCTCAGAGGGATGATGTGAAA
GTGCTAGAGAATGTGAATATGGTTGTAGATAATAACAATTACAAGACGCAGATTGAGTTTGC
GGGATCATCAGTTGCTGCTTCTTCGAATCCATCTACAAACACTCAGCAAGAAAAATCAGAAT
CTTGTACAGAGAAAAGACCAGTGAGCTTGTTAGCAGGAGCAGGAATAGTTTCTGTTGTTGA
TGAGAAGAGACCGAGAAAGAGAGGGAGAAAGCCTGCAAACGGAAGAGAAGAGCCATTG
AACCATGTGGAAGCTGAGAGGCAGAGACGCGAGAAGCTTAACCAAAGATTCTACGCTTTA
CGATCAGTTGTTCCAAACATTTCTAAAATGGACAAGGCTTCTCTACTTGGAGACGCAATTTC
TTACATCAAAGAGCTTCAAGAGAAAGTCAAGATAATGGAAGATGAAAGAGTAGGAACAGA
TAAGAGCTTATCAGAATCAAACACAATAACAGTAGAAGAAAGTCCAGAAGTTGACATTCAA
GCTATGAATGAAGAGGTTGTTGTAAGAGTAATCTCGCCTTTGGATTCACATCCAGCTTCAAG
AATCATACAAGCAATGAGAAACTCAAATGTTAGTCTAATGGAGGCTAAGTTATCATTAGCTG
AAGACACAATGTTTCACACTTTTGTGATAAAGTCTAACAACGGGTCGGATCCATTGACGAA
AGAGAAGCTTATAGCAGCGTTTTACCCCGAGACCAGCTCGACGCAACCGCCATTGCCTTCT
TCTAGTTCACAGGTCTCTGGTGATATATAAGTTTCACATGATGGTGAAGCTTTTGTCTATTCT
TGTTTCAGTATAGATAACTTTCAGGTTCAACTAGAAATAGATAAAATCCTTTTTTCTTTTCTCT

TTCTTATAGTGAAAGGAATCCGGTTTAAGTATATTCATTAACTTTATTTAGACCATTGATTCAC
ATATTTGTATCTTGGGAAAATATTTCCTATACTTTAGAAAATCAAATAAAGTATGAAAGATATT
TGACTTCTC

>AtbHLH018
CGTGTTTAGCCATTAGCACATGAGCAAGTCCAAATCTCCCTCTCTCTCTCTCTATCTATCTCT
CTATAGAAGGTAAACTTCAACTTCATCTTTCTTAGACTGCTTAAATTTCTCAAATTCTTGTAA
AGCTTTTGGAATTCAAGAAATATTAATGTTATATAATTTCATTTTCTTGGGTTAATAGTTTCCT
CATATGATTACCAATTTTCAGATTTTTTAACTAAGAAGCTAGCGATCATGGCCACAGCGATGA
ACGTTTTCTCTACCAAATGGTCCTCCGAATTGGTATATTTCTATACACACATACATACATATTT
ATGTGTGCCTCTCTTTCCTCATATGATTTTGTATATATATAAAATCTATATTCATGTTCTTTTAAC
CTACTTTTTCTTAGGCAAATTTCACGAATCTTTATTATTTGCTTTTCGTATAGGATATAGAAGA
ATATAGTATCATCCACCAATTCCACATGAACTCACTCGTCGGAGATGTTCCACAGTCTCTCTC
ATCTCTTGATGATACCACCACTTGTTATAACCTTGATGCTTCTTGTAATAAAAGTTTGGTAGA
AGAAAGACCTTCAAAGATCCTCAAGACCACTCACATATCACCAAACTTACATCCTTTTTCTT
CTTCTAATCCTCCTCCTCCAAAGCACCAGCCCTCTTCTAGGATTCTTTCTTTTGAAAAGACA
GGTTTACATGTTATGAATCACAACTCTCCAAACTTAATATTTAGCCCCAAGGACGAAGAAAT
TGGATTACCAGAGCATAAGAAAGCCGAGCTGATAATAAGAGGGACAAAGAGAGCTCAATCC
TTGACTCGAAGCCAATCAAATGCTCAAGATCACATACTGGCAGAGAGAAAACGGAGAGAG
AAGCTTACTCAAAGATTTGTAGCTCTTTCCGCGCTAATTCCTGGCCTAAAGAAGGTACACAT
ATGTAAAAATAAATCTTCTCTTCTTCTTCTTGTTTAATAAAGAGGCGCATACCAACCAAATGA
TAGTGTAGTCTTTTCTTAAATAATTCTATTTTGCTAGGCAAGAAACCAATTTTCATGGACAGT
ATTCACTCTTTTCTTATACCCACAATGAAAGTTTTGACTTCAGATCTCCCGGTTATGAGACTA
AATTATTTGACCAGGACCGACTTTGTGAAACTCGAAATATACTAAAACAAATCCTCTATATAT
AAGAATTATTACTTTTTTACAAATCATTTGCAGTTCCGGCTTGAAGGTTTTTCATTTCGATTA
AATATAAATATATTTACATGCTATATATTGAAATATGCATCTATGTCATTATAGATGGACAAGGC
TTCTGTGTTGGGAGATGCAATAAAGCATATAAAGTACCTCCAAGAGAGTGTGAAAGAGTAT
GAGGAACAAAAGAAGGAAAAGACAATGGAATCAGTGGTTCTTGTAAAGAAGTCTAGTCTG
GTTTTAGATGAAAATCATCAACCATCATCATCATCTTCCTCAGATGGAAATCGCAATAGCTCG
AGCTCAAATCTTCCAGAAATAGAAGTTAGGGTTTCAGGAAAAGATGTTCTTATTAAGATCCT
ATGCGAGAAGCAAAAGGGTAATGTGATCAAGATTATGGGGGAGATTGAAAAGCTTGGTTTG
TCTATCACCAACAGCAATGTCTTGCCCTTTGGACCCACTTTTGACATCTCTATTATCGCTCAG
GTAACTATATATTTTCTCATATAAGCTACAACATAACTTTATTCTAGTATTCAACATAAATATAT
ATATCGTGTGACAACATAAATCAATTCTATAGATAATGTACATGTTTCAGAATTAGTATGACTT
TGGACTTAAATATTCTAGTATTCGAAAATTCTAATGATATAGTTTATTCTTTGCAGAAGAATAA
CAATTTTGATATGAAAATCGAGGATGTTGTGAAGAACTTGAGTTTTGGCTTATCAAAGCTCA
CTTAATTGGTTTCACGTTACATACATATACACATTCATCATCGATTTCTCCGATCGAAGAATCC
AAAATCAGTTTTTCCATGAAAGTGGTTTTTTAGTTGTTAAGTTTGTTGTATGGAGATTCTTAA
GTCATTTAAAGATCCTTGTTCTTGTGTTGTTAAGTGTGCTTTAAGATGCATATCATCAAATGTT
TAGTAATTATTTCTCTCCAGTTTCATTTGGGACGGAATTTTTTTCGCAGTTGTTGGATATATAT
TTCCTGCGATGTAAAGCATTTCGTTAGTTT

>AtbHLH019
ATAATTTAAGCCGCGTCTCGTTGTCGAATATATAGTAAGAATACCTGAAAACACTTTCTATTC

ATAAACCCTTTACGACAAAACAATAAAGACGTCTCTCGATTTATCTTTGTCTTAATCAGGAA
ACTATTTGACATTTCTTTATCGATTATTACTTCATATTCGGGTATGGATGAAGATTTTTTCTTAC
CCGATTTCTCACTAGTAGACATTGATTTCGACTTTAATATTTACGAAGAAAATAATTTATCACC
TGATGAATCTTTATCAAATTCACGAAGAGCTGATCAATCGTCAAAGTTTGATCATCAAATGC
ATTTTGAGTGCCTCAGAGAGAAGCCAAAGGCAGCGGTGAAGCCTATGATGAAGATCAACA
ACAAGCAACAACTGATTTCTTTTGATTTCTCTAGTAATGTTATTTCTTCTCCAGCCGCGGAGG
AGATCATTATGGATAAATTGGTTGGTCGTGGAACCAAAAGAAAAACATGTTCTCATGGCACA
AGATCTCCGGTTCTTGCGAAAGAACATGTTTAGCCGAGAGAAAGCGCCGTGAGAAGCTTT
CTGAAAAATTCATTGCTCTTTCAGCTCTCCTTCCCGGGCTTAAGAAGGTATTGCAAATTTAA
ATTGTAAATTATCGATAAATTTGTTATATATTTAACTATAGATATTTTCATAGTGAAGAAAAAAT
AATAATTCATCAGTGTCAATTGGTTTTGAGAATACAAAACTATCTTGTGGTTTTTTAGGCGGA
CAAGGTAACGATTCTTGATGACGCGATCTCGCGTATGAAACAACTCCAAGAACAACTAAGA
ACGCTCAAGGAAGAAAAAGAAGCAACGAGACAAATGGAATCCATGATTCTTGTAAAGAAA
TCTAAAGTGTTTTTCGACGAGGAACCTAACCTATCATGTTCTCCTTCAGTTCATATTGAATTC
GATCAAGCATTACCAGAAATCGAAGCGAAAATTTCTCAAAATGACATTCTCATCCGAATCCT
TTGCGAGAAAAGCAAAGGGTGCATGATCAACATACTAAACACAATTGAAAACTTTCAATTG
CGCATTGAAAATAGCATCGTATTGCCATTCGGAGACTCGACTCTCGACATCACAGTCCTTGC
ACAGGTGATCATTAATAACTATATATATATGCAATACTAAAATGGATAACTCGTAGTTCATCCA
ATATAATATTGATGTATTAATGAAACTGATAATCTACAATTATGCTTTCTTAACTTTTTGTGTGG
CAGATGGATAAAGATTTTTCGATGAGTATACTTAAGGATCTTGTACGGAACCTGAGACTCGC
AATGGTTTAGGTCTTCTTTCAAGTTTTAGTAACCTGAGACTGTACATTGACACAATCGTTTCG
TGGATTTGCTATCGTTTTCTTCTTGAGATTTTTTCACACGTGCCCACATGTTTGATGTGTTTTT
TTAGCAAAGCTATTTCCGATAACGACCGTTGAGAATTAATTTAGATGTAACATTTTTTGTTTG
TTAATAATGAATTTAAAATATAACAGAATTGTTTTTTTATTAAGTGGCAGAAATAAATGTGGC
CTTATCCGCTGTCCAAAAAAAAAAAAACAAATGTGGCCTTATCCTTAGCATGGTCGTAATGCC
TTCTGACTTGTTAAAGTATTTTGTAATTATTG

>AtbHLH020
GCCAAATTCATATAACAATTATAAACCATATGCTCTTTTTCCATGCAGATATTAATTGTATACTG
CAAAATAATGATTATAAAAAACAAAATCATATAGATCCACTATAAAGAGTTCACTCGACCAC
ATTCACATAACAAAACAAAATCATACATACTCCAGAACAAAGGAAAATTTAAAATATATCTC
CTTATATACTCCTAAAGTTTTCTTTCCTTTAGCTTCTTGTTTAGTTTTATTCTTTCAACATTAAC
TTTTTTTTACTCTCTTGTCAAAAGAGAAAAAGAGTTCTTGTAATGGATGATTCAAGCTTTATG
GATTTGATGATCGACACTGACGAGTATCTGATCGATGACTGGGAATCCGATTTCCCGATATGC
GGGGAAACTAACACCAACCCCGGTTCCGAGTCTGGATCTGGAACCGGGTTTGAGTTGCTAG
CAGAAAGACCAACGAAGCAAATGAAAACCAACAACAACATGAATTCAACATCTTCTTCTCC
GTCGTCTTCTTCTTCTTCTGGTTCTCGCACGTCACAAGTGATCTCATTTGGGTCTCCAGACA
CGAAAACCAACCCGGTCGAGACATCTTTGAACTTCTCCAACCAAGTAAGCATGGATCAGAA
AGTGGGGTCGAAAAGAAAAGATTGTGTTAACAATGGAGGAAGAAGAGAACCACATCTATT
GAAAGAACATGTTTTGGCTGAACGAAAACGTCGACAAAAGCTCAACGAGCGTTTGATTGC
TCTCTCTGCTCTTCTTCCTGGCCTCAAAAAGGTCAACATCTATAAACCATCTCTTTCTGATTG
TACTTTAACTATTTGTGTTTGGTTTAATATTTATTATATAGTATATTCTTTTATAAATAACCCACC
AGAACCGTAAATATATATTCGGAAAAAGAAACTCATTTTTCTTTAAATGTAAATGTAAACTCC
AACCTCAACAAACATAAGAAAATCTTGTTCTTATTTACATTTTGAAAATGAAAATCTGTTTTT

TTTCTTAAACGTTTTTTTTCTCGTAAAAATCGTGTTTAATATGATAGGCTTGTAATTTGTTTGT
ATGTGGATGCCACTACTCATAAAATCCGGATGGTGACACAATGTTCTCTAGTAAATATTGTAC
TAAATGTTTTTTTTTTTTTTGTGTGTGTGTGTGTAGACGGACAAGGCAACAGTTCTTGAAGA
TGCCATCAAACATTTGAAACAACTTCAAGAGCGGGTAAAGAAGCTGGAGGAGGAGCGAGT
GGTGACCAAGAAAATGGATCAATCAATTATATTGGTGAAGAGATCTCAAGTGTATTTAGATG
ATGATTCTTCATCTTATTCTTCTACTTGCTCTGCTGCGTCTCCTCTGTCTTCTTCTTCGGACGA
AGTTTCGATCTTCAAACAAACAATGCCTATGATCGAAGCCCGAGTTTCAGACAGAGATTTGT
TGATTAGAGTCCATTGTGAGAAGAACAAAGGGTGTATGATTAAGATCTTAAGTTCCTTGGAG
AAGTTTCGTCTTGAAGTAGTCAATAGCTTCACTTTACCATTTGGAAATTCAACTCTCGTTATA
ACCATTCTCACTAAGGTATGTTATCAATCTTCTAATATTTTTTATTTATCATATTCTATCTTTTT
TTATATATTATCATTTACTGTTGATTTTTATGTATTAATGTGATTTTTAAATCTAATTTGCAGATG
GACAACAAATTTTCTCGACCAGTTGAAGAAGTGGTGAAGAACATAAGAGTTGCGTTAGCTG
AATAATATTTTAAAAAACGTTTATGTGAATTTCGCAGTGGTTAAACGTGTTAGATTTGGTCTC
CAATTTTGACCAGATCACTCGGTTTAGTTGAATAATGAGTAGTTGGATAATAAAACCAGATAT
ATGCACGAAATATGGGATTTTGTCGTTTGTTGAGTGCAAAAGCTGGATTGGTGTGTGTACCT
AATTATGGCTGTATCATTGGTTTTGATACTTTGATATATATTTTTCCCCAATATCATTGGGTTGG
TTCAATAATGAAGTACCTAAGTGTTCATTAATTTTATTGGCTTC

>AtbHLH021
ATGGAGAGTAATATGCAAAACTTGTTGGAGAAATTGAGGCCTCTTGTGGGTGCAAGGGCTT
GGGATTATTGTGTTCTTTGGAGACTAAACGAAGACCAAAGGTTGATTTGATTCTTTTAATAA
GTAGTTAAGCTTTAAAGAGTTTTTATCTCTATCTATCCATCTTTCTTTCTCTTTCGTCACAAAT
ATTTGTTATTGTGGTTTTGGAGGAGACAGGTTTGTGAAGTGGATGGGATGTTGTTGTGGTGG
GACAGAACTCATAGCGGAGAATGGAACAGAAGAGTTTAGCTACGGAGGTTGTAGGGATGTT
ATGTTTCATCATCCTCGGACCAAATCTTGTGAATTTCTTTCCCATCTTCCAGCTTCCATACCTC
TTGATTCCGGGTATGATCATCTATAATATATATACAAGCTACATATATTCTTTCATAAGAAACGC
CTAGAGATGGAAAAATATGGATTTTGCTATAGGATATATGCGGAGACTCTTCTGACTAACCAG
ACTGGTTGGTTGAGTGAGAGCTCAGAACCAAGTTTTATGCAGGTACTATTACCCGATTCTTG
GAGTTTTGAGTAATCACAATCTCATGAACAAGAAGTATTAATTTTCGGTTTTTCTTATATCCT
GTAGGAAACAATCTGTACTAGGGTTTTGATTCCTATACCGGGAGGACTAGTGGAGCTCTTTG
CGACCAGACATGTATGTTTATGTCTCAATAATGTTAAGTTTATGGTTTTTTGTATTGTGGTTGT
TTGTGATGATGTGTGCGCGTATAGGTCGCTGAAGATCAGAACGTGGTGGATTTTGTAATGGG
ACATTGCAACATGTTGATGGACGATTCTGTAACGATAAACATGATGGTAGCAGACGAGGTCG
AATCGAAGCCGTACGGGATGTTATCCGGTGACATCCAGCAAAAGGGTTCCAAAGAAGAAGA
TATGATGAATCTCCCTTCGTCTTACGATATCTCTGCTGATCAGATCCGACTCAATTTCTTGCCT
CAGATGAGTGATTACGAGACACAACACTTGAAGATGAAGAGTGATTATCATCATCAAGCCTT
GGGGTATCTCCCAGAGAATGGTAACAAGGAAATGATGGGTATGAACCCATTTAACACGGTG
GAAGAAGATGGGATTCCAGTGATTGGAGAGCCTAGCTTGCTTGTGAATGAACAGCAAGTTG
TTAACGATAAGGATATGAATGAGAACGGTAGGGTGGATTCAGGGTCGGATTGCAGCGACCA
GATTGATGATGAAGATGATCCAAAGTACAAGAAGAAGTCAGGAAAAGGATCTCAAGCCAA
GAACCTGATGGCTGAGAGACGGAGGAGGAAGAAGCTAAACGATAGGCTTTATGCTCTCCG
GTCGCTTGTTCCCAGGATAACCAAGGTATCTTGGTTTTCGTCTCTGGGTTTACTTCTTGGTTT
CTGAGTTATAATGCGTTCTCTGAAACTAGTTTTTGTGGCATGGAAGTTGGACAGGGCGTCGA
TTCTTGGCGATGCAATCAACTACGTTAAGGAGTTGCAGAATGAGGCAAAGGAGCTTCAAGA

CGAGCTTGAAGAGAACTCAGAGACTGAGGATGGATCTAATAGGCCACAAGGAGGGATGAG
CCTGAACGGGACAGTGGTCACTGGGTTTCACCCGGGGCTTTCATGTAACTCCAACGTTCCT
AGCGTGAAGCAAGATGTTGATCTTGAGAATTCCAATGATAAAGGACAAGAAATGGAGGTTA
GAGCATCATAACAAGAAACTAACTCTCACCTTTCTTCTAATCTGAACTCTAAAATCAAAACC
TCATGATATCGGTTTATGTTGGGTTGCGGAGTTTCAGCCACAGGTGGACGTGGCTCAGTTAG
ATGGCAGAGAGTTTTTCGTAAAGGTGATTTGCGAATACAAACCAGGAGGCTTCACAAGGCT
AATGGAGGCACTGGATTCTCTTGGACTAGAAGTCACAAATGCTAACACGACTCGCTACCTC
AGCCTGGTCTCCAATGTCTTCAAAGTCGAGGTATGATCTAAAGGACCTGCGGAATGGAGAA
ACAAAAACCAAAGCAAAATATACAAAAACTATATGTTTCTGTTTTCCCATGTTACAGAAAAA
TGATAACGAGATGGTCCAAGCTGAACACGTAAGGAACTCGTTGCTGGAAATAACCCGGAAC
ACATCTAGAGGATGGCAAGATGATCAGATGGCTACAGGCTCTATGCAAAACGAAAAGAACG
AAGTTGATTATCAACACTATGATGATCACCAGCATCACAATGGTCATCATCACCCATTTGATC
ACCAGATGAATCAGAGCGCTCATCATCATCACCACCACCAACACATCAACCATTACCACAA
CCAATAAGGACCAATTCCCCAAAGCTATAAAGATGTCTGCTTTACAAGCAAAAGAAACATT
CTGCATCAACAAACTTTGAAGATCTTTTCATTTCAAGATATATAAGGATAAAAGGAATGTCA
GTTCGAGAACAGTTCCTGTATCCAATACCAGGAATAAACCAAAGCGAAACAACTTTACTTA

>AtbHLH022
ATGGGTGGAGGAAGCAGATTTCAAGAACCAGTGAGGATGAGCCGTAGGAAACAAGTAACA
AAAGAGAAGGAAGAAGATGAAAACTTCAAATCTCCAAATCTTGAAGCAGAGAGACGTAGA
AGAGAGAAGCTTCATTGTCGGCTTATGGCTCTGCGATCTCATGTCCCCATTGTCACCAACGT
AAGTCCCAAATCTCGATTATTATTTTATGGGTATTTTGGTGATGATTGTTGTTGTTTTTGTTTC
GTTTTCAGATGACTAAAGCAAGTATTGTTGAAGATGCGATTACTTACATAGGAGAGCTTCAA
AACAATGTTAAGAATCTCTTAGAGACATTTCATGAAATGGAAGAAGCTCCTCCTGAGATTGA
TGAAGAACAAACGGATCCAATGATAAAACCTGAAGTTGAAACTAGTGATCTTAACGAAGAG
ATGAAGAAACTCGGAATCGAGGTTTTTAAAATTTCTCTAATCGATTTTTGATTCATTTTCTTTA
TCTTTCTTGATGATTTTTTATTTTTGGGACAGGAGAATGTGCAATTGTGTAAGATTGGGGAGA
GGAAGTTTTGGTTAAAGATCATAACAGAGAAGAGAGATGGGATCTTTACTAAATTCATGGA
GGTTATGAGATTTCTCGGATTCGAGATTATCGATATTAGTCTAACAACTTCAAATGGAGCAAT
TCTTATTAGTGCCTCTGTTCAGACACAGGAACTCTGTGATGTTGAACAGACAAAAGATTTTC
TTTTGGAAGTTATGAGAAGCAATCCATAA

>AtbHLH023
TGTCCATATGAGTTTCTGGGCGACTTCTATCAGAATTTTTGTTCATAATTTTTTTTATTGTATC
AAGGGATGACTTGGAAACCGAAGATGCTTATATTATCCCATGATCTAATCTCACCAGAAAAA
TACATCATGTAAGTTCATTTTCGAATTTCTTTCTCTCTTACTTGCATGTTTATTCCAATATGAG
GTGTATATTTTAGTGGTTTTGGTTTTGTTTGAGTTTAGGGGTGAAGATGATATCGTGGAGCTC
TTAGGGAAGAGCAGCCAAGTAGTTACAAGTAGCCAGACACAAACACCCTCTTGCGATCCTC
CTCTCATTCTCCGGGGCAGCGGAAGCGGGGACGGAGAAGGAAATGGTCCTCTACCGCAGC
CTCCGCCTCCCCTGTACCATCAGCAGAGTCTCTTTATCCAAGAAGACGAAATGGCTTCTTGG
CTTCACCAGCCTAATCGCCAAGATTATCTCTACTCCCAACTTCTTTACTCCGGAGTAGCCTCG
ACTCACCCGCAAAGTTTGGCCTCCCTAGAACCACCACCACCACCTAGGGCTCAGTACATTC
TGGCGGCGGATAGACCGACCGGTCATATTTTGGCCGAGAGAAGGGCGGAGAATTTTATGAA
TATCTCGAGGCAAAGAGGGAACATATTTCTTGGCGGTGTTGAAGCTGTACCGTCGAACTCG

ACCCTGTTGTCTTCAGCCACTGAATCAATACCAGCGACTCACGGCACCGAGAGTCGAGCAA
CAGTCACTGGCGGAGTATCTCGTACTTTTGCAGTTCCTGGTCTTGGTCCGAGGGGAAAGGC
GGTGGCGATTGAGACGGCGGGAACACAATCTTGGGGGTTGTGCAAGGCCGAAACAGAGCC
GGTTCAGAGACAACCAGCGACGGAGACGGATATCACCGATGAACGGAAGAGAAAAACGA
GAGAGGAAACAAATGTCGAAAACCAGGTGAGTCAGAGCTCCCATATATAGGTTAACTTTAC
GCAAACATGTCAGCGCGTGTTAGTCACTTGACTCGTGCAGTTTAGCAGTTGAAGCTTAATTT
GTATCTTTCTGTTTCTTGGCGTACAGGGAACTGAAGAAGCTCGTGATTCGACGTCTAGTAAG
AGGTCACGAGCTGCAATAATGCATAAACTCTCCGAAAGGGTTCTTATGCTTCCATTGGAATT
TCAAGATTCTGAGTTTGAATAATGGACTATCTGTTTCATCTCCTTTAAAACTTGTTTTTTTTT
GTTAAACAGAGACGGAGACAAAAGATTAACGAGATGATGAAGGCTTTGCAAGAACTCCTT
CCTCGCTGCACAAAGGTTATTACAGTTTCTTTTTTAATTAACTAGTTGTTATTTAACTTTAACG
TTGATTCTGATTCTGTGTCTACTCTGATCAGACTGATAGATCTTCCATGCTGGATGATGTTATA
GAGTACGTGAAATCTCTACAGAGCCAAATACAGGTATGTCCTCAAAAACAGAATGTGAGTT
TGAACATGGAAACAGTTTGAATGTCCGCAGATTCATTTCCTGGAAGATTTATAAAGTTTAGA
TGAGATGGCCTAATAGCCCTGTTCTTGAGGTCTTTGCTTCCCAAATTTTGTAGGGTAAACATT
TAAGGATCTTTAGGTCATGAGTGAGATTTCATATCTGAATCGAGGTTTTTATGCCAGATGTTC
TCAATGGGACATGTTATGATTCCACCGATGATGTATGCGGGGAATATACAACAACAGTACAT
GCCCCACATGGCCATGGGTATGAATCGGCCTCCTGCATTCATACCTTTCCCTAGGCAGGCTC
ATATGGCGGAAGGTGTAGGTCCTGTTGATTTATTTAGAGAGAATGAAGAAACAGAGCAAGA
GACAATGTCTCTTCTCCTTAGAGAAGACAAAAGAACAAAACAGAAAATGTTTTCTTGAACT
GAAACTTGTTAGTTCTTTTATTAAGACAAGACACACTCTTATATACATGTTCACATAACTACT
CTACGTTGGTAACAGTTGTAACTTCTCCAACAAGTTACGTCTCTTACTCGCCTCCTTTCGTA
GTCACATGACGTCCGTTGACTTAACATACAAACTATAACATATATGATATTACAAGATTTAATC
ATGGACTCAATTCTCAACAGGTCCATCATCATATCCACCACCGCGCTACCCTTTTCCAAACAT
TCAGACCTTTGACCCATCCAGAATTCGTTTACAAAGTCCGCAGCCTAATCTGGTGTCGAACC
AACCTCAGATGAATCCCTATAGCCAGTTTGTTGGTCACCACCGGATCCAACAACCTCGTCCT
CCATTGCAGGTAATTCTTCACCAGTATCCCATTGTGCTTATTCCTCTCTGTCCCTACGAACGC
TTACAAGATCCTTTTCTAAAAAAAAATGTTTTTATAGAGTCAAACAAGACCACAGGTGAGTT
TCAGCCAAGCAAGTAGTAGCAAAGAACCTGATGATCAGGACAACAAACCAACAAGTTAGT
ACGAATCAGGGAATAAAGGCTTTTCTTGCTAGATAAGTTAATAGTCAAGATTGTGAATTTCA
ATGGGTAAAGTCATCCTACGTTCACAGTCTCACAGATAAATTTATAACACATTTAATAATAGA
TTTTTTAGAAATTTGCAAAATTAAGGGATAGATTTGGCACAAACTCATTAACGTCACACAAA
ATATGTAAAAAAATTAAAAGGACGAGGCAGAAAAAGATCATGTTGTTTTCTAACGTCGATAC
AAAAAACTCTGA

>AtbHLH024
TCTCTCCATGCCCATAAAATCTCAAAGACTGTTTAAAAAAAAAAAATGTTTTAGCTTTAACTG
CTTTTTTTTTGTTGTTGGTGTAATGATATCACAGAGAGAAGAAAGAGAAGAGAAGAAGCAG
AGAGTGATGGGAGATAAGAAATTGATTTCATCTTCTTCTTCTTCCTCGGTTTACGATACTCGT
ATCAATCATCATCTTCATCATCCTCCGTCTTCTTCCGACGAAATCTCTCAGTTTCTCCGGCATA
TTTTCGACCGTTCTTCTCCTTTACCTTCTTACTACTCCCCGGCGACGACTACAACGACGGCG
TCTTTGATTGGTGTGCACGGGAGCGGTGACCCACATGCAGATAACTCGAGAAGTCTCGTTT
CTCATCATCCACCGTCAGATTCTGTGCTTATGTCGAAACGTGTCGGAGATTTCTCTGAGGTT
TTAATCGGCGGAGGATCAGGCTCAGCCGCCGCGTGTTTTGGTTTCTCCGGTGGTGGTAATAA

TAACAACGTTCAAGGAAATAGCTCTGGGACTCGAGTATCGTCTTCTTCCGTTGGAGCTAGTG
GCAACGAGACAGATGAGTATGACTGTGAAAGCGAGGTTTTTCTCAATCTCTGTTTTTATC
TTTTGCATGTTTTAAAAGTTGATTCTTTGTCTCTTTCCCGAGAAAATGTTTCTACAGAGATTT
CTCTTTAACGGATCTGTACTTTATTTTCCCCCTTAAAATCCGCAATTTTTTTGGTTGTGATTTT
GAGTGAAGTTTTGTCATTTGTGAGTGTAATAATGAATGCACACCGAGTGTTTGATAATTTGC
TTCAATGAAAATACAGGAAGGAGGAGAAGCTGTAGTTGATGAAGCTCCCTCTTCCAAGTCA
GGTCCTTCTTCTCGTAGTTCATCTAAAAGATGCAGAGCTGCTGAAGTTCATAATCTCTCTGA
GAAGGTTTGGTTTAGAGTCTCTCCCAAAAGTAGAAACTTTCTTATTTGAGATTTATGTTTATC
TTTCTTGTAACAGAGGAGGAGAAGTAGAATTAATGAAAAAATGAAAGCTTTACAAAGTCTC
ATCCCTAATTCAAATAAGGTAATGATACTATATATAGAGAGACATCTACATGAATCATTCTATT
TTCTGTAGATTCTTTTTGTGTCTGATTATTGCAATGTGTTTATTGGGGAAGACGGATAAGGCT
TCAATGCTTGATGAAGCCATTGAGTATCTGAAACAGCTTCAGCTCCAAGTTCAGGTCACTAA
AGATACCTTATGTTTATCATTATAACCAAAATCCTAAAACCAGTCTATATTCTGACATAAGTCT
TTTTTTGTTCTGATATTTGCAGATGTTGACTATGAGAAATGGAATAAACTTGCATCCTTTGTG
TTTACCTGGAACTACATTACACCCATTGCAACTCTCTCAGATTCGACCCCCTGAAGCAACCA
ATGATCCTCTGCTTAATCATACCAATCAGTTTGCTTCGACTTCTAATGCACCGGAAATGATCA
ATACTGTGGCTTCTTCATACGCTTTGGAACCTTCTATTCGCAGTCACTTTGGACCTTTCCCTC
TCCTTACTTCACCCGTGGTGCGTAGTTGAATTACACATTTTTCATTCTATAACCATAAAGCTA
GTTTGCTTACTAATTTTTTTTTGTTACTTAAGGAGATGAGTCGGGAAGGTGGGTTAACTCATC
CAAGGTTGAACATTGGTCATTCCAACGCAAACATAACCGGTAAAGTCTTGTTGTTTTGGAAT
CCTAAGGAAGTTTTAGGACATTTACAATGTTAATAACGTTACGATATTTGGGGATTTGCAGGG
GAACAAGCTCTGTTTGATGGACAACCTGACCTAAAAGATCGAATTACTTGAACAGTGTCCC
AACTTCGGGATCTCTATGTGTTCTTGTTTCTTAGAACGCAAGCCATAAAGCTGTCTGACAAT
GGAGATGCATTTAACTGTCTTTATTTTTTCTAAGGTTCTATTTAAAATCAATTGTTGGTGACA
CGAATTCTAGAGTCTAATCTTTTGGATCTAATAGCTATATATAAAAAGGACTGAAATTTTATTA
CAAAGTAATTGACTAATTGTTAGATTGTTGTATGTTTATGGTGGATGTAAATTTTCTAGATCAT
CTTCGTATTGTATTTGAGAGTTTGATACTCGATGGCTTTGGATATGTAAATTACCCCGATAAGC
TTCTAGAGAAGTTTTCGATTCTACAAGATGGATTTGGTATCTTTAAGGTTGACCATGTTCTTT
TGGTATCTCTTGTTCTCAAATTGATCATTTACAATACAATTTTTTAAAATC

>AtbHLH025
ACACAAATCTCTCTTCTCTCTCTCTCTTCTCCTAGAAGCTGGTGAGTTTTATTTCTTTGTGAT
TCAAAAAACATAAATCTCAACCCTTACTAAATGTTTCTTGATCTTGGGTTTTTACTATACGTC
AAATCTTGTATTTGAAACCCAAGATGATCTGCTAATTTATCTTTTTTCTTGGTTAAATACAGTT
TCTTGTTTATAAATCATACACTAATATTCTTTTTACCATTTTCAGATTTTCAGAGGCAACAGCA
ATGAGTATCTTATCCACAAGATGGTTTTCTGAGCAGGTATATATGCGCATAAACACATACACT
CATGTATATATGTTGTCATCTGGGACTGTTTTTCATTGTTTTTTGTTCTTTTTTCTTTTGGGTGT
GATGTTATATTTTAGGAAATAGAAGAAAATAGCATAATTCAACAGTTTCACATGAACTCAATA
GTGGGAGAGGTCCAAGAAGCTCAATACATTTTTCCACACTCTTTCACGACCAACAATGATC
CCTCTTATGATGATTTGATCGAAATGAAACCACCAAAGATCCTTGAGACAACTTACATATCA
CCTTCTTCTCATCTTCCTCCAAATTCAAAGCCCCATCATATTCACCGTCATTCTTCCTCAAGA
ATTCTCTCTTTCGAAGATTATGGTTCAAATGATATGGAACACGAGTACTCTCCCACCTACCTA
AACTCAATCTTTAGCCCAAAATTAGAGGCTCAAGTGCAACCACACCAGAAGAGTGATGAGT
TTAATCGAAAAGGGACCAAGAGGGCTCAACCGTTTTCAAGAAACCAATCCAATGCTCAGGA

TCACATAATAGCCGAAAGAAAACGTAGAGAGAAGCTTACTCAAAGATTCGTAGCTCTTTCC
GCTCTAGTTCCTGGCCTTAAAAAGGTATACATACCATCAGATCTTCCTTCTTGATTACCTTTTT
CAAAAAAATACATGTTTTTGAATCTATAGGTGTAATTTTTTTCTAATTGCGTGCATAGATGGA
CAAGGCTTCTGTGTTGGGAGATGCATTAAAGCATATAAAGTATCTCCAAGAAAGAGTGGGA
GAGTTGGAGGAACAGAAGAAAGAAAGAAGATTGGAATCAATGGTTCTTGTGAAGAAGTCT
AAGCTGATCTTGGACGATAATAATCAATCATTCTCTTCTTCTTGTGAAGATGGCTTCTCGGAC
TTGGATCTTCCCGAGATCGAGGTAAGATTCTCGGATGAAGATGTTCTAATCAAGATCCTTTG
CGAGAAGCAAAAGGGTCATCTTGCCAAGATTATGGCCGAGATTGAGAAACTTCATATCTTGA
TAACTAATTCAAGTGTATTGAATTTTGGACCAACTCTTGATATCACCATTATAGCTAAGGTAT
GCAAATGTATATTCAGGCATACACTTATACATATAAATGAAGAACTTGTTCTTAATTTTAGAGT
AACATCGCTTGATGTTTATTTTTCAGAAGGAGAGTGATTTCGACATGACACTCATGGATGTT
GTAAAGAGCTTAAGGTCTGCTTTGTCGAATTTCATATGATTGTATTAACTTTCATGCTTCTCG
TCAATTTCTACTGTGGGAGATCTTGTTGAACACATGGACGAAACATAAGTGGTTTGCCATGA
AAATGTTTTTTTTTTTTTATAAGTTTGATTGGAGGTTTTCTTATCTGTTAATTATCTATAATTCT
ATATATAAATAAGATTAAATTAGTTGAAAATTCACTTCTCTTTTTTTCTCTATTTGGTTCTTTCC
CCCTTTATGTATTATATTGCTGTTTCCAAAGATGTAAAGCACTTCCGTTTATGTATTTGTATTAT
ATGGTTTAT

>AtbHLH026
AAACGAACCACACATACTTTGAAACGTGGCACAATATAAGTGGGTCACCAATGTAGCTGAC
AACTATACCATCTATCTGATCCGATCGCTCTTATAACATAAGCGTATGGGATACCATTTTCTCG
GACAAAGCTGAAATCCCTAAAGAAAAAACACTTCTCCAAACTTTTCATCTCCGATATCTCTT
TAACTAACATGTAAGTATAATTAAACAGGAGGTTTCAGAGTTTAGGTTTCTTGCCTTGTTATT
CTCTTTATTTATTTCTTTAATAATTTATCAATGATTTGTATCGTAAACCCTAATAGTTTACTACTT
TTTTCTTGGCAGGTCGAATAATCAAGCTTTCATGGAATTGGGATGGAGAAACGACGTCGGAT
CACTTGCTGTGAAAGATCAGGGCATGATGTCAGAAAGAGCAAGAAGTGATGAAGATCGTCT
AATCAACGGTCTAAAATGGGGCTACGGCTACTTTGATCATGATCAAACTGATAATTATCTTCA
GATTGTTCCAGAGATTCATAAAGAAGTAGAAAATGCGAAGGAGGATTTATTGGTTGTTGTCC
CTGATGAACATTCTGAAACTGATGATCATCATCATATTAAAGATTTTTCAGAGAGATCAGATC
ATCGATTTTATCTGAGAAACAAACATGAGAACCCCAAAAAACGTCGTATCCAGGTCTTAAGT
AGTGATGTAAGTCGATACATAGATATATTTTCATGTTACCTTATGTATTATCATTTTTCACTGTT
TTTAATTTTTTCTTTCTATAAATAAAATTAATTAATTTAGGATGAATCGGAGGAGTTTACAAGA
GAAGTTCCTTCAGTTACTCGAAAAGGTTCCAAGAGAAGAAGAAGAGACGAGAAGATGAGT
AATAAGATGCGTAAGCTACAGCAACTCGTACCTAATTGTCACAAGGTTGGTGTGTATATAGA
TCCTTCTATATAATGTTTCAATTTAACTATTGAGTGTCTAGTCTTTTCCGTATAAGTTTCCGCTA
CTTCCGTTGCATATATGAAGAAATCTTGCCTTAAGTTTCCAATTCTGATAACGGGTGGTGCTT
TTTTACGGTTTTCAGACGGACAAGGTTTCGGTTCTCGACAAGACCATAGAGTATATGAAAAA
CCTTCAACTTCAACTTCAGGTCTATATTTAACTTTATTCACTATTTTTATTTCACACTTTATTCA
GTGTTCTTCTTCCATATATTTGAATTATTGAGGCTTGAGGTTTTAAGTGTTTATCTAATAGTCT
GAGTTTTTTTTTGGTTAATTTAACTAGTTGACATAAACTGTTCTTGGTATTCGATCTGACTCG
CCAACTTATGAGATATGGATTATTGTTTTACAATATGGGTTTCAAATTGATCAAACAAATATAG
CCACTTTTTGGTGTTTAGTGACGAAAAAAATGGAATGATATATAGTAGGCAGTAGATAACCT
AGATGGTGAAGTCTATAGAATTTCTTTAATATTTCGAATTCATCTCTTTTCATGTGATTATTTA
GTGCCAACTAAATGTTTTATATTTTATAGAACTATGTACCCATTTTGATCATTCGAATATGTATA

TGTGCTATTTATATTCAGAAACTAAGTTGTAATCAAAGTTGATGTTATAATTGCAGATGATGTC
AACAGTGGGGGTGAATCCTTATTTCTTCCGGCGACATTAGGATTTGGAATGCACAACCACA
TGCTGACGGCAATGGCTTCGGCTCACGGCCTAAATCCGGCGAATCACATGATGCCATCGCC
GCTAATTCCGGCGTTAAATTGGCCATTACCACCGTTTACTAATATTTCATTCCCACATTCATCT
AGTCAATCTCTATTTCTTACAACATCATCACCAGCTTCTTCTCCTCAGTCTCTTCACGGTTTG
GTTCCTTATTTCCCAAGTTTCTTGGATTTTTCTTCCCATGCGATGAGAAGACTATGATAAGTA
AGTAGCTCGATAAAAGTTTATGTGAAATGATCGTTGACTAATTAAGAGAGAAAAGAGTTCC
AAATTAGGACAGTTTGTGTGAACCATTTGTGTCATATACTTTTTTTCCGGGTGGTTTTGTATA
TTAATAATAATCAATAAGTTAAAACTTTTTGTATAAATTCTTGCATGTAACAATTTTTTCATTTA
TCTGATATTTTGTAAAATATATTGAGACATGCTTATAAGAGCATTGAACGTAGGAACAAGATT
TCCCTGATTCTTAAGCGTCTCAAACGTTTACACTTGGTTACATGCATCCCAACTAGCT

>AtbHLH027
CACTCTTGTTTTGATAGTACACTCAATAGAAAGACACAAAGAAAGAAAGAAAGGACAAAC
AGAAATAACAAAACCTTCCATTTCAAGAAAGCATCATCTTTGTGTGTCCGAAAAATGGAAG
ATCTCGACCATGAGTACAAGAATTACTGGGAAACCACAATGTTCTTCCAGAATCAAGAACT
CGAATTTGACAGGTGCTTACTAGAATCTTGGTCCATTTTTTTTTTTTTTGTTTCTTTAGTTTTT
TTTTCCGATAAAGATTAATCTTGCTTTAGTTGGTAATTAATGAAAATTGTTATAAATTATAAAA
TTTAAGTTGGCCGATGGAGGAAGCGTTTTCGGGTTCCGGCGAGTCGAGTTCGCCAGACGGA
GCGGCAACGTCGCCGGCTTCTTCGAAGAACGTTGTCTCCGAGAGAAACAGACGGCAAAAG
CTTAATCAGAGACTTTTTGCTCTCCGGTCAGTTGTTCCCAATATAAGCAAGGTATGATTAATT
ATTCCATTACTTCTCTTATTTGTTTAATCAACTGTCTCTTTTAAATTTAATTTGTTATCCAAATA
ATTTAGTAATAAAGTTTCGTCACCAGCAATTTTTGTTTTGGTCAACAGAAATTAAATAAGCG
AATAAACTTTCTCTTCTTTCTTTTGTAAAGAAATAAAACTATACAAATACAATCTGGTTAAAC
CAAATCCAACCAACATTAAACAAATAAGCAAACCAAATTTTTGATACTCTAAATCCATATCA
AGCCATACTAAAATGTATTTTGGTCCCCTAATTACCCTGATAGCATATAATTGTATACGTGTGA
TGATTTGTTGATTAAGACTGGATTTTATTGAAGTATTAATATTTATCTATTTTTAGTTGGACAA
GGCATCTGTCATCAAAGATTCTATCGACTATATGCAAGAACTTATTGATCAAGAGAAGACTCT
AGAAGCAGAGATCAGAGAGCTAGAATCACGGTCAACATTGCTAGAAAATCCGGTAAGAGA
TTACGATTGCAATTTTGCGGAAACTCATCTGCAAGATTTCTCAGACAATAATGACATGAGAT
CAAAAAAGTTTAAGCAGATGGATTACAGTACTAGAGTACAACACTACCCCATTGAAGTTCT
CGAAGTACGTACTATGCATTATTTGGCAGTACATTCTAAATTACCTTTCTTTTGAGGTAAAGT
TAATTATTCCAAATTGTTTTTATATAAACATGAAAAAACAGATGAAAGTGACATGGATGGGA
GAGAAGACGGTAGTGGTATGCATAACATGTAGCAAGAAAAGAGAGACAATGGTGCAGCTTT
GTAAAGTGTTGGAGTCTTTGAATCTCAACATTCTCACTACTAACTTCTCTTCCTTCACCTCTC
GTCTCTCCACCACCCTCTTCCTCCAGGTCACTCTCTCCCTCTCTCCCTCTCTCATATCTTTATT
TGGCAATGTGATTACTAGTACAAACTACAAAATTCTAAATGCATCTAGAGAATACTGTACGT
GTCTTGTTTTGGTTTGACGCTCATTTTGCACTCATGGAGCGAAATTATAAAATGTTAATGGAA
AGAGATGATAATATAGTTTAGAAGATTCTTGCTAATAAAGAATGTTAGATCAATTGTAATCAG
CTATAAAATTAGTTTTATGCACTTGTCGGACTATAAAACACACCAACCAAATATATTTGTCCA
TTTTTAATTTGTCGCAACTATTCAGACTTTTATATACCAAATTCAAATCTTACTATTTTCTTTTC
TTTTTGGTTATTTTGGGAGAAAAATGGTCGTCTGACGGCACGTAGACAAATCCTTTAGTGGA
CAAGAATTAATAATATGAATAAAAAGCATATGACTAAGTGCTTTAGTAAAGTGGATTGTTCTT
TTGTTTTGGTAAAGAAAATATGTTATCTTTACGTAATAGTACAAAAATGTAATTCAAACTTAC

GAATTGTTTGGAAAATCACTATCAAATTCAAAAGGATAACCTTTAGACGGACCCATTTAATT
ACTACTTTTCTCCCACCTGTCTCCATTAAGTGGACCTTAATTCACATTACACCTTAAGTATATA
TAGTCAGTAATATCTTAATCAATCAATTAAAGCTCCAATTATTGAGGTTGCCTCGAGCCCATC
ACATTTTTTTCGAATTTCGCCTTGACTTTTTCTGATAGGGTTAGATTCTCTCAAAAGTTAAAT
GTCATATTCCATAAAAAATTAGGATTAACTTAATCAAAAAATTAGTGTGTGAAATCTCAAGTG
CCTCGTGTCCCAGAAAGTGCCGTCCAAGCATAGTAGCACTAGAAAAACGTGGCTAATTTAT
AACCTCACGCGGTTCAACGTTTGTCTCTTTCACACTTTTAGCACGTGACTTTTCTAGCACTA
CACCTTATATCAATTCTTAACTCAAATTAACAATAGGCTACAAATCAACTGTAGCCAATTAGC
AAGTTATTTGTGTTAACTAGTTGTATATGGTTCATAAGGTCTTCATTATGCTAATCATTTTGGT
AAAAATTAGTTACTCCAAATGATATCCATAGCAGTTCATGGTAGAAATTAAGGTTTGGTCCTT
TTTAATATAAGCAGATTTGTTAAGAGTTATTATTTTGTGTTTTTACTCTAAAAAATACAAATTC
ATTTCATTATCTCTATCTATTTGATTTGTTTATCATCATCATATACTAGGACTTTGGTGTAAACA
ATCTAATTACAAAAATGCTAGTTTCGTGAACTAACGTTCTATTTTTTTTGAAAAAGGTAATTA
TATTATAAGCTGTAGGTTGGAAATTTTTGATGACAACAAATACGAGTATTTCTTGCCTCACAA
ATGAATATAATATTATGTTGGTTTTCTTTGACAAAAAAGAATTATGCACCTTTATAACATTTTC
TTGATAAAAGATAATATAATCAGACGCTAAAATTACAGTGAGGTATAGACCAGTGGAGTCAT
TGAGAGTTGGTGAAATCATACTCCAATCATTTATCAAATAAAAAGTCGATCATTTCATTTCAA
ACCTGTCCTTATCCGTCCTTTTAAAGATTATACAAAAACATTAAACAATGATTTATCCACCAA
TCTCATTTTCCACGGGAAATAATTATAGTAGTAATGCGTGCAGGTGTATATACACAAATTTATC
AGTTCTAATTTTCTTTTTACGTGCTGCCAAAAAAAAAGAAGTTTTTTTTGTCTGATTCGTGTA
AAACCTAAACACCCCTAATTTTAATAATCTATACTGACGTTGGACTTCCTAGAATCCTAATTT
ACTTCAAAATTCTGTAAAGATATCGTATGATTGAAATAATATAGGACTGTTTTAGTTTATATTG
TGATAGATGTATTAATTATAGTAAATCGTTGAATTTGCAGGCGGATGAAGAAGAAAGCAG

>AtbHLH028
TTAAAATTCATGAAAAGAAAACCACAGAAAAATGTAATATTTTCATGCACTCGTGTGAAAC
AAAATAAAGAGTACACTCTATACTCTATAGTTTCTCGTTTAGTTTAGATATATAAATACAAAG
ACTTGAGACGACTTCTAATACTCCGGCAACGATGATTAATACCGACGATAACTTATTGATGAT
CGAAGCTCTCTTGACCTCCGATCCGTCGCCACCATTACTTCCGGCGAATCTCAGCCTCGAGA
CTACTCTCCCGAAGCGTTTACATGCTGTGTTGAATGGAACCCACGAGCCCTGGAGTTACGC
CATTTTCTGGAAACCGTCGTACGATGACTTTTCCGGTGAAGCAGTTCTCAAATGGGGCGAC
GGAGTTTACACTGGTGGCAACGAGGAAAAGACACGAGGGAGGTTGAGGAGGAAGAAGAC
GATTCTGTCGTCTCCGGAGGAGAAAGAGCGTCGGAGTAATGTTATTCGGGAGCTTAACTTG
ATGATCTCCGGCGAAGCGTTTCCGGTGGTTGAAGACGACGTCAGTGATGACGATGACGTGG
AAGTGACGGATATGGAGTGGTTCTTCTTGGTTTCCATGACGTGGAGTTTTGGTAACGGATCC
GGGTTAGCGGGTAAGGCGTTTGCTAGCTATAACCCGGTTTTGGTTACCGGTTCGGATCTGAT
TTACGGGTCGGGTTGTGATCGGGCTAAACAAGGAGGGGATGTAGGGTTACAGACCATCTTG
TGTATTCCTTCACATAACGGAGTGTTGGAGCTTGCATCCACGGAGGAGATCCGACCAAATTC
GGATCTTTTTAATAGGATCCGGTTTCTTTTTGGAGGATCCAAATATTTTTCTGGAGCCCCGAA
TTCGAACTCGGAGCTTTTCCCATTTCAGTTAGAGAGCAGTTGTTCAAGTACTGTAACCGGTA
ACCCGAATCCTAGTCCGGTTTATCTACAGAACCGGTATAATTTGAACTTCTCGACGTCATCTT
CCACATTGGCGAGAGCTCCATGCGGCGATGTACTGAGTTTTGGCGAGAATGTTAAACAGAG
TTTTGAAAATCGGAACCCTAATACTTATTCTGATCAGATTCAAAACGTCGTCCCTCACGCGA
CGGTGATGCTGGAGAAGAAGAAGGGAAAAAAGCGCGGGAGAAAACCGGCGCATGGTAGA

GACAAGCCTTTGAACCACGTGGAAGCAGAGAGGATGAGACGTGAGAAGCTAAACCACAG
ATTCTACGCATTACGAGCGGTTGTACCAAACGTATCGAAAATGGACAAAACGTCGTTGCTCG
AAGACGCGGTTTGTTACATAAACGAGCTGAAATCAAAAGCTGAAAACGTTGAATTGGAGA
AACATGCGATTGAGATTCAGTTCAATGAACTCAAGGAGATTGCAGGACAACGAAACGCAAT
TCCTAGCGTTTGTAAATACGAGGAAAAGGCATCAGAGATGATGAAGATCGAAGTGAAGATT
ATGGAAAGTGATGATGCAATGGTTAGAGTTGAATCAAGGAAGGATCATCATCCAGGAGCGA
GATTGATGAATGCTTTGATGGATTTGGAGTTAGAAGTGAATCATGCGAGCATTTCTGTGATG
AACGATCTTATGATACAACAAGCGAACGTGAAGATGGGGTTGAGAATCTACAAGCAGGAAG
AGCTTAGGGATTTGTTGATGTCGAAAATTAGCTGAGAGTTTCTGCGGTGTGTTTTGAGAAAA
TTAATCATGGGGTCAAAGTGTAATTAAGTTTTGTTGATTTCTCATAAGTTAATGGCCGTTTTA
TTAGATTTTGGATAATATACAGTTCTAATCTTGTGACCC

>AtbHLH029
CATTTTGTCTCTCTTTCGTTGTCAACAACTTTTCTTCATTGACAAAAACACACAAATGGAAG
GAAGAGTCAACGCTCTGTCAAACATAAACGATCTCGAACTTCACAATTTCTTGGTCGATCC
AAACTTCGATCAGTTCATAAACCTCATAAGAGGAGATCATCAAACCATTGACGAAAACCCA
GTTCTTGATTTCGATCTTGGTCCATTACAAAACAGCCCCTGTTTCATAGACGAGAACCAGTT
CATCCCAACACCTGTCGATGACCTCTTCGACGAATTGCCTGACTTAGACTCCAACGTTGCTG
AATCATTCCGTAGCTTCGACGGTGATAGTGTTAGAGCCGGTGGTGAAGAAGATGAAGAAGA
TTACAACGACGGTGATGATTCTTCAGCCACTACTACGAATAATGATGGGACCCGTAAGACGA
AGACTGATCGGTCTAGGACTTTGATCTCTGAGAGAAGAAGGAGAGGGCGTATGAAGGATAA
GCTTTATGCATTGAGATCTCTTGTTCCCAATATTACTAAGGTAAGTCACAACATTTTCAAGAT
TTGGTAATGACTAATGAAACTGTAAATATTGATGAAGTATGTGTGTGTTGGCATTTAGATGGA
TAAAGCATCCATTGTTGGAGATGCAGTGTTGTATGTTCAAGAACTTCAGTCACAAGCGAAG
AAACTCAAATCCGATATCGCGGGTCTTGAAGCTTCTTTAAACTCTACTGGAGGGTACCAAGA
ACATGCTCCTGATGCTCAAAAGACTCAACCTTTTCGCGGTATCAATCCTCCTGCTTCCAAAA
AAATCATTCAGGTAATAACCTAAAAAAACCAATCAATCTTCTTGAAACATCAAAGTGTTTAA
AGTTTGTATGTTTTGTTGTGTTAGATGGATGTTATACAAGTGGAGGAGAAAGGGTTTATGT
GAGATTGGTGTGTAACAAAGGAGAAGGTGTTGCTCCATCTCTTTACAAGTCTTTGGAGTCT
CTTACAAGTTTCCAAGTGCAGAACTCTAACCTAAGCTCTCCTTCTCCGGACACATACCTCTT
AACATATACCTTAGATGTAAATCAACTTGTGCCCTTTTACTTTGTTTTGTTTCCGAGTATACCG
TTTACTTACCGAGTTTTCTTGTGTGGTGGAAACAGGGGACATGCTTCGAACAGAGCTTAAA
CTTGCCTAACCTGAAGCTGTGGATCACTGGATCACTTTTAAATCAAGGTTTTGAATTCATCA
AGTCATTTACTTGATTCTATAACGCTTGCTCTAACGTGAGTCAAATCCGGTTCTGCACTATAT
TGATTGTGTACCTTTCTTACATGTTTCATAACTTCCAGGGCTCTAATTTCTATTCTAGTGATGA
TGTAACCGAGATTGTTGATTCTCTATTGAATAAACACCATGTTATATAGTAATTTAGCGACAA
ATTGTATGGTTAAATGAAGTAATATTTATGTTTTGTTTATAGTCTCAAGTCTTAAGTTTCTTTT
GCTTTGATATATCACGTTTTGGTTCATTGGCTGTATTATTGCTTTGTTGTTGCAAGACCATTCA
CATCTTGATTCTATGTATTGTTCTCTGTTTGATTGGTAAAAGAATAAAGAATTTTAACCCCGA
G

>AtbHLH030
ATATGTCTGCGTCTACAACATTTGAAAGCAAACTTTTGTCTCTTTTTAACTCTCTTAACTTTC
GTTTCTTCTCCTACCTTCTTTTACCAACCTTTCCTTTCTCTTACACACATATATATATACATATAT

AGAGAGAGAGAAGAGGACAAAGAGTTGAAAGATGAAGACTCTCATGTCTTCATAGAAACA
AGTGATATGTGCGCTAAGAAAGAAGAAGAAGAAGAAGAAGAAGACAGTTCTGAAGC
CATGAACAACATACAAAATTACCAAAATGACCTCTTCTTTCACCAACTCATCTCTCATCATCA
CCATCATCATCATGATCCTTCTCAATCTGAAACTTTGGGAGCATCCGGTAACGTTGGATCTGG
TTTCACTATCTTCTCTCAAGATTCCGTCTCTCCAATATGGTCTCTACCTCCACCTACCTCGATC
CAACCACCATTTGATCAGTTTCCTCCTCCTTCTTCTTCTCCAGCATCTTTCTACGGAAGTTTC
TTCAACAGAAGTCGAGCTCATCATCAGGGATTACAGTTTGGGTACGAGGGTTTTGGTGGAG
CCACGTCAGCAGCACATCATCATCATGAACAACTTCGGATCTTGTCGGAAGCTTTAGGTCCG
GTAGTACAAGCCGGGTCCGGTCCTTTTGGGTTACAAGCTGAGTTAGGGAAGATGACAGCAC
AAGAGATCATGGACGCTAAAGCTTTGGCTGCTTCAAAGAGTCATAGTGAAGCTGAGAGAA
GAAGAAGAGAGAGAATCAATAATCATCTCGCTAAGCTCCGTAGCATATTACCCAACACCACC
AAAGTAAGTCCAAAATTTTTATAACATAAATTTTCATACATAAGTAAAAACATTCATCAATCA
CACACCGTTTAAGTTTTTTGTAAATAAAAAATAATACTAAAACAATGTAGAGTTTAAGTGGA
AAAACATAGTAGCTGCGACATGAAAGTTTATGTCACATTTTGTCTCTTTTTATTTTTTTACATT
TAGGTTTTATAAAAGGAGAAGTAAGTTATTCAAAGGTTTTCTGTTTTGATCAATGATCTTGTG
GGTATTTGTTAAAGTCCCGAGAGAGAATCAGTCAGAGACTGTAGTATAAATAAAAAAGCAA
GGAATATGGAACGTCTATGTCGTCGTTTGGTTAATTGATGTAATAAAGAGAAACAACCGGCG
CTGCTTAAAAAGCATAAAAAGTCTTATTGCCGGACCAAATCATAACTTCTACGACCACATAA
TTTTCTCCTCGAAACTATACAAATATGTTTTTTCTTCGTTTTTTTTAATGCAGACGGATAAAGC
GTCGTTACTAGCTGAAGTGATCCAACATGTGAAAGAGTTGAAGAGAGAGACTTCAGTGATC
TCAGAGACAAATCTTGTCCCAACGGAAAGCGATGAGTTAACGGTAGCTTTCACGGAGGAG
GAAGAAACCGGAGATGGCAGATTTGTAATTAAAGCGTCGCTTTGCTGTGAAGACAGGTCGG
ATCTCTTGCCTGACATGATTAAAACATTGAAAGCTATGCGTCTCAAAACGCTCAAGGCGGA
GATAACCACCGTTGGGGGACGAGTCAAGAACGTTTTGTTTGTTACCGGAGAAGAGAGCTCC
GGTGAGGAAGTGGAGGAAGAGTACTGTATAGGGACGATTGAGGAAGCTTTGAAAGCGGTG
ATGGAGAAGAGCAATGTAGAGGAATCATCTTCTTCTGGAAATGCTAAGAGACAGAGAATGA
GTAGTCACAACACTATCACTATCGTCGAACAACAACAACAATATAATCAGAGGTAATCAATT
TTTTACTTAAATCGCTTTTTTTTTCTTACTTTCGGTGTATCTACTACGTGTGTTGTTTGCTGGT
TATGGAAATGAATGTTGTACGTCACGTTATACTATAGATATATGTGTGTTTGTGTGTATGTATA
ACGGAAGTATTTGTATCCGTTGTGGTCTTGGACTTTTGGTTTGGTTCTAAGATACTTATTTTTA
AAAACTTGTATCGTTGAGTTGGTTTTCTAGATATGCTTAATGGGAGTATGTGACGAAAAAAA
AGTATGATTTCTGTAAGGCAATGCATGGGTTTGGAAAATTCGTTTTCTCTTGTCATGTGGGA
GGATTTGAATTTAATTTTGGCTTATTTTTGCAATTGTGTGAATGAGACCAAAAAGAGACAAT
GAAGAAGAACAGGAAACGTACCAAAGATGATGTGTATCATTAATTGATTTAGTCGGGTTCTG
TCCGGGGGAAAAACAAAAAGAGACAAATTTGGGATTCTGAGCTTCGTACGTTGATGCTTCT
CTGACTCTTTTGCCTTGTGTATTTATGTGTTCTGTAGAATCTTTTTCGTGTAACGATTTACATA
TATTCAAATATTTTGCTTTATATAAATTTGCATCATGGACTGGTATGAGGTAAACATCGGGACC
AAGACTAAGATATAAAG

>AtbHLH031
TTTAAATATGTTGGGAATATTATTAATTTATTAACAAGAACACAGTAACGCAATCACAGACTC
CACAGTCCAAGGAGTTTTGCATACTCACCAAGCCACAATCATTTCTCTCTTCTCTATCTCTCT
GGTTTTGAATCGGCGACGACTGAGTCAACTCGGTGTTGTTACTGGTTTCGTCGTATGTGTTG
TAACTGATTAAGTTGATGGATCCGAGTGGGATGATGAACGAAGGAGGACCGTTTAATCTAGC

GGAGATCTGGCAGTTTCCGTTGAACGGAGTTTCAACCGCCGGAGATTCTTCTAGAAGAAGC
TTCGTTGGACCGAATCAGTTCGGTGATGCTGATCTAACCACAGCTGCTAACGGTGATCCAGC
GCGTATGAGTCACGCGTTGTCTCAGGCGGTTATTGAAGGTATCTCCGGCGCTTGGAAACGG
AGGGAAGATGAGTCTAAGTCGGCGAAGATCGTCTCCACCATTGGCGCTGTACGTATTTTCTC
TCTTTCTTTTTTTTGTCCGGTTTGATTCGGTTTTTACCGGTTTAATGGTTCGACTTCTAATTAT
TTGGTTAGGCCGCTCATAATACTATTTTTGTGTGTGTGATTAGAGTGAAGGTGAGAACAAAA
GACAGAAGATAGATGAAGTGTGTGATGGGAAAGCAGAAGCAGAATCGCTAGGAACAGAGA
CGGAACAAAAGAAGCAACAGATGGAACCAACGAAAGATTATATTCATGTTCGAGCTAGAA
GAGGTCAAGCTACTGATAGTCACAGTTTAGCTGAAAGAGTAATGATTTCACATGATCATATA
AAAAGCTTCTTTTATTTGGTTGAGTGAGTAAAAGACAATTTGCTTTGTGTTTTTGGTAGGCG
AGAAGAGAGAAAATAAGTGAGCGGATGAAAATCTTGCAAGATCTTGTTCCGGGATGTAACA
AGGTATTGTAAAACTTTTCAATGGGAGATTCACATTTTTTTTAGAGTGGTGACATGATTGAA
ACTTGTGTATGTTTTGTAGGTTATTGGAAAAGCACTTGTTCTAGATGAGATAATTAACTATATA
CAATCATTGCAACGTCAAGTTGAGGTGTGAAACGTCTCTCTCTTCTGTTGTTTTGTTACTATA
CATTTACATGTGTGTTCTGATGTGATGGTTTGGGATTTTCAGTTCTTATCGATGAAGCTTGAA
GCAGTCAACTCAAGAATGAACCCTGGTATCGAGGTTTTTCCACCCAAAGAGGTGATGATTC
TCATGATCATCAACTCAATCTTCTCCATTTTTTTCACAAAACAATACATGTTTCTATCGAGGTA
TTCTCGGGGTAGGAGTCTCGATGTTTATGCGGTTCGGTCATTTAAGCATTGCAATAAACGGA
GTGACCTCTGTTTTTGCTCCTGCTCCCCAAAAACAGAACTTAAGACAACTATATTTTCACAA
AACATGACATGTTTCTGTCGATATTCTCGAGTAGGAGTCGCTATTAGTTCATCTAAGCATTGC
AATGAACCGGTTACGCTCTGTTTTTACTCCTACTGCCTAAGGAAAATTTATCATTTTCTGTTG
TGGAACTTAAAATACAAAATTCAAAAATCTGTTTTGTTTTCTTGATTTGGTTGGCTAATCTCT
GTTATATTTGGTGACACTCAGTTTGGTCAGCAAGCGTTTGAGAATCCGGAGATACAGTTCGG
GTCGCAGTCTACGAGGGAATACAGTAGAGGAGCATCACCAGAGTGGTTGCACATGCAGATA
GGATCAGGTGGTTTCGAAAGAACGTCTTGATAAAGAAACAACACTTGGTCATCATCTTACT
ACAAATAAGATCTAAAAGCTCTAATATCCCGAGAAGAAAAAGCATACATACATATAAATCTC
ATTCTCATTATAGGTTTAAATATATGGATGTGAATATACACATCATATTGTTGTTCTGAAGAAA
GCAAAATAGAAAAGAAGATATAGTTAGTTTCGACATCAAGAAAAAACAAGCTGGGAAGGG
TTTAAAGTAGAAGAAACCTCTTGTAAAAGAAAAAACGTTTGTTGTTATAAAATTGTATCTCA
TTCTTACATATTTGCTTAAGTTCTTGCTGAACA

>AtbHLH032
ATGTGTATGCATTATCAAATGGGATGTTTGTGACCTTTTGTCTCTTCTTCTCTAAAATTCTCTT
TCTCCCAAAAACTAAAAAACCAAAAAAACCAAAAAATGTACGCAATGAAAGAAGAAGACT
GTCTTCAAACATTTCACAACTTACAAGACTATCAAGACCAGTTTCATCTTCATCATCATCCAC
AAATTCTCCCCTGGTCGTCGACATCTTTACCTTCTTTTGACCCACTCCATTTCCCATCTAACC
CGACCCGTTATTCTGACCCGGTTCACTACTTCAACAGAAGAGCTTCTTCTTCCTCTTCTTCTT
TTGACTATAACGACGGTTTTGTCTCTCCTCCTCCTTCCATGGATCATCCTCAGAACCATCTAA
GGATTTTATCCGAAGCTCTTGGACCCATCATGCGTCGTGGCTCGTCCTTTGGGTTCGATGGT
GAGATCATGGGAAAATTGAGTGCACAAGAAGTCATGGATGCTAAGGCTTTAGCTGCTTCAA
AGAGTCATAGTGAAGCTGAGAGAAGAAGACGAGAGAGAATCAACACTCATCTTGCTAAGC
TGCGTAGTATATTACCAAACACAACCAAAGTAAGTCGTTTATAAATAAACACTCTTCTCCTTT
CATTATGGTTTGATATTTCAGAAACTAATTCACTATTTTGAAAACCCTATGGAAAAATAGTTTT
AATTAATTAATGATCCTTCAAAATTTCTCTTTATTTAGTTATTATTGGTTAGAGTAGTAGTAATT

GTTGTTGTTTTTAATAAAGAGAGTAGTAGTAATTGTTCTTTTTCTGATAAATATAGTACTAAT
TATTCAGTTTCAAAAATGATAAGTACTATATTTTTCAATATTATCATTTTTATCTATCTCTGTTC
AACTATTATCGTTTAGTAGTTGTAAATTTTCAAATTTAAAATAAATGTATCAAATCGATATTTT
TGGTAAGTAATTATAGTCCACAGATGGGGTATTTTTGTACTAGTTATTATTATTGAAGAAGAA
GAATAAGAAGATTAAATACTTATGATTCGTTTTGGTTTTGTATAGACGGACAAAGCTTCTTTG
CTAGCGGAAGTGATCCAACACATGAAGGAGCTAAAACGACAAACATCACAGATCACCGAC
ACGTATCAAGTCCCAACAGAGTGCGATGATCTGACCGTAGATTCGTCTTACAACGACGAGG
AAGGAAACTTGGTGATAAGAGCATCCTTTTGCTGCCAAGACAGGACTGACCTCATGCATGA
CGTCATCAATGCCTTAAAGTCTCTTCGTCTTCGAACTCTCAAAGCTGAGATCGCAACCGTAG
GTGGTAGAGTCAAGAACATCTTGTTCTTGAGCCGAGAATACGATGATGAAGAAGATCATGA
TTCATATCGTAGAAACTTCGATGGTGATGACGTGGAGGATTATGATGAAGAGAGGATGATGA
ATAATCGTGTGAGTTCGATAGAAGAAGCGTTAAAGGCGGTTATAGAGAAGTGTGTTCATAAT
AATGATGAAAGTAACGATAACAATAACTTGGAGAAATCATCTTCAGGGGGTATTAAGAGGC
AAAGGACTAGTAAGATGGTGAATCGATGTTATAATTAGTTAATTAAGTCAAGTCTTTATTAAC
TAGGGTTAGTTAATTAGACTTGCAAAATGGGATTTGATTATGGGTTGGTGATATTAGTATTATT
TTCGGTGTTTTTAGTAGTTGGGATTGGGTTTCTTCTTCTATGTTTTTTTAATCTATGAAGAACC
CTTTGTTTAGGGTTTTATAGTGATGAGTTTGGTTTATATATGGTAGTTAAGACATTTTATCGTC
TTAATTAATATAGGAAATGTATAGAGCTTGGGTTTGTATTAATTTATCAAGAAGTTTTATTTATC
ATATTCATGAACTCTGCAACTATTTAATCTTTTGTTTATAAGCAACAACACTACGTAGTAATAG
TATAGAGTATAGTTTTATACTTGTCAATTTATTTTCTATTGTGAC

>AtbHLH033
TTTAACTAAATCATCTTCCTAAGCTCTATCACTGAAATAATTCTGCAGACAAAAAAAAATGA
GAGAGAGGTGGCAAAGATGAAGTGGAGGTGAGAGACGGAAAGCAAAAACAATAGTCACG
AGAATCAACACCAACTCTTAAACCAAGATAGACCAAGAAACCAAACAGAGTAAATAAATG
GTGGCCTTGACAACATTGAAATCCCCAAAAATCTCAACTTTTTAGGGTTTTCGAGCTCTGAA
ATCTCTCTATCTTTCTTCTTCAGATTTCTCACTTTTTTCTTCTTCATAAGATCCTCGTGATTCCA
CTTTATAACGTACGCCTCCTTTCAAGAAAAATCTCTCTCTATATTATCTACGTTTCTCTCGCCT
TGTCTCCTTTTGAGGTTCCATTTCTACTTCTCTCCAAAATTCGAAACTTTTCCCACTCTCTCT
CTCTCTCTACTAGTTTGACATGAACAGCGACGGTGTTTGGCTTGACGGCTCCGGTGAATCTC
CGGAAGTTAATAACGGTGAAGCTGCGTCTTGGGTCAGAAACCCAGATGAAGACTGGTTCAA
TAACCCACCACCACCACAACACACTAATCAAAACGACTTCAGATTCAATGGTGGCTTTCCTT
TAAACCCCTCAGAGAATCTGCTTCTTCTTCTTCAGCAATCGATTGATTCTTCTTCTTCTT
CTCCGTTATTACATCCTTTCACACTCGACGCTGCTTCACAGCAACAACAACAACAACAACA
ACAACAGGAACAGTCTTTCTTAGCTACGAAAGCTTGTATAGTTTCTCTTCTCAACGTCCCAA
CCATCAATAACAACACTTTCGATGACTTCGGCTTTGACTCTGGTTTCTTAGGACAACAATTC
CATGGAAATCATCAATCTCCGAACTCGATGAATTTCACTGGCTTAAACCACTCAGTACCGGA
TTTTCTTCCAGCTCCGGAAAACAGCTCAGGATCATGTGGATTGAGTCCTCTGTTCTCAAACA
GAGCAAAGGTTTTAAAACCGTTACAGGTAATGGCTTCATCTGGCTCGCAGCCAACTCTGTTT
CAGAAACGAGCTGCAATGCGTCAGAGCTCGAGTAGCAAAATGTGCAATTCTGAGAGTTCTT
CTGAAATGAGGAAATCGAGCTACGAGAGAGAGATTGACGATACTAGTACCGGAATCATCGA
TATCTCTGGATTGAATTACGAATCTGATGACCATAATACTAATAACAACAAAGGTAAGAAGA
AAGGAATGCCTGCAAAGAATCTTATGGCTGAGAGAAGAAGAAGGAAGAAGCTTAATGATA
GGCTTTACATGCTTAGATCAGTTGTTCCCAAGATCAGCAAAGTAACCAAAAAGATTCTTCCT

TTTTTCTCTTTGTTCTCCTTAATAACTTCTTGTGTCACTAATTTTGGGTTGCAAATGTAGATGG
ATAGAGCATCAATACTTGGAGATGCTATTGATTACCTCAAAGAGCTTTTACAAAGAATCAAC
GATCTTCACACCGAACTTGAATCTACTCCACCGAGTTCTTCAAGCTTGCATCCGTTAACACC
GACTCCACAAACGCTGTCTTACCGTGTTAAGGAAGAGTTGTGTCCATCTTCCTCCTTGCCAA
GTCCTAAAGGCCAACAACCAAGAGTAAGAATAAGCTCAATTGCTTTCTAGTGGACAAAGCT
GTATTCTTTAATTTTCTTAGTAACGCTTCTTTGTTTTGATATAGGTTGAGGTTAGATTAAGAGA
AGGAAAGGCAGTGAACATACACATGTTCTGTGGACGTAGACCAGGTCTTTTACTTTCCACC
ATGAGAGCTTTGGATAACTTAGGATTGGATGTTCAACAAGCGGTGATTAGCTGTTTCAATGG
TTTTGCTTTGGATGTTTTCCGCGCTGAGGTAATATAGTTGTTAAGATGCTATGCTGATCACTG
ATTAGTAAAACTAGCAGTCAAAGAATAGTTATTTGAGAGAATGATTAGTATACAAGTAACAA
CATGTTTTGTGTTTCTAATCAGCAATGTCAAGAAGACCATGACGTGTTACCTGAACAAATCA
AAGCAGTGCTTTTAGATACAGCAGGTTACGCTGGTTTGGTTTGATTGCGAAGCAGAAGGAA
GCATCTGTGTGGAACAGAGCTAGATCTAAGTCCTTGTTCTGTTTTAGTCAGCAATTTTCTTAC
TTAGTTTCATCAATTTCAATCATGTATGGAAGATGATAACTGTTTTATTTTAGCTATGAGAACA
TGTGTTTGGGGAACAATAGTTGCAACCTTTGAGCAAAATGCCATTGTTTCTCAATAATTCTA
CTTTGCTTTTCATTTATATGTTGTTTGCAAGAAGAGAGCAATGTTGATTTTCA

>AtbHLH034
GAATAAAATTTGGTTTAAAATTCCAAAATTCTCTCAAATCTTTAATATATATTTTCCAATAACA
AAAGATATATATCTCCTCAAATTTTCTAGGGAATACAGTATTTGTGTGTTTGAGACAGATTTTT
GCAGATTCTCTCGTTCTGCATTCTCTGTTCTTCTTCTGATGTATCCATCAATCGAAGACGATG
ATGATCTTCTCGCTGCTCTTTGTTTTGATCAAAGGTTTGATTTTTATTGTTGAATTGTGTTGTA
TTGTGCCTTTTGTTATTTGGGTTTCTTAACAATTCCAAATTTCAATCCAATTTTAGCAAAAGT
TTCGATTTTGAATATGAAAAGTTTTTCTAGCGGTCGTTATTGTTGTTGATGGAGATTGTTGTT
ACCTTGATTCTGTAGCAATGGAGTAGAAGATCCTTATGGATATATGCAAACAAATGAAGATA
ACATATTTCAGGATTTTGGGTCTTGTGGTGTGAATCTGATGCAGCCACAACAAGAACAATTT
GATTCTTTTAATGGAAATCTTGAGCAAGTTTGTAGTAGCTTTAGAGGAGGAAACAATGGAGT
TGTTTATAGTAGTAGCATTGGATCAGCACAATTGGATTTGGCTGCATCGTTTAGTGGAGTTTT
GCAGCAAGAGACACATCAAGTCTGTGGCTTTAGAGGACAAAACGACGATTCTGCAGTGCC
TCATTTGCAGCAGCAACAAGGACAGGTGTTTAGTGGTGTAGTGGAAATCAATTCTTCGTCAT
CTGTTGGAGCTGTTAAGGAAGAGTTTGAGGAAGAATGTTCGGGGAAGAGGTAGTCTTTTAA
GAATTTGAATACCTTGCTCTTTACTTTTCTTTGTTCTGATGATTTGAAAGATGTTGTGTTTTCT
GTGGTGTGTTTAGATTGAATTAAGATATTTGCAGCTAATGATTTCTCTTGGTCTCCACTAGTT
TTGGGTCTTGAATTGAGTCTTTTAGTTATGTAGCTTGGATTGGCCATGGTTATCTTGTTGTTTG
GTTATCTACAAGACAACTATAGCATCCATTTCGTTGTTTGATGTGATTATGTATGAACAGGAG
ACGAACTGGATCATGTAGCAAGCCAGGAACCAAAGCCTGTCGCGAGAAACTAAGAAGGGA
AAAGCTAAATGACAAGTATGTTCAGAGCTTTCTCTACATATGTTTTAGTTATTGCTTGCACTG
TGTTGTTCTGCAGTCGTCTTATATGAAAAAATATTGGGTATTTTTTCAGGTTCATGGACTTGA
GCTCTGTTTTAGAGCCTGGCAGGACTCCAAAGACGGATAAATCAGCTATACTCGACGATGC
AATCCGGGTTGTGAATCAGCTTAGAGGTGAAGCTCATGAGCTTCAAGAAACCAACCAAAA
GCTTCTAGAAGAGATCAAGAGTCTAAAGGTTGGTTTCTCACTTTGTCATAAAGCTCAAGGT
CATAAAACTTTGTCATAAAGTTATGAGAACCTAATTTAAGTAGTAGTCTGTGTATCTTCTTCT
TGAAAATGTTTCTAGCTCATAATCATTATGTTTGGTACTTATCTGATTTCTCATCTAACTGTCT
GTAATAGGCGGATAAAAACGAGCTACGAGAGGAAAAGCTGGTGTTGAAGGCGGAGAAGGA

GAAGATGGAGCAACAGTTAAAATCTATGGTGGTTCCATCACCAGGTTTCATGCCCTCCCAGC
ATCCAGCAGCTTTCCATTCCCATAAGATGGCGGTGGCTTACCCTTACGGCTACTATCCTCCAA
ACATGCCAATGTGGTCACCCTTACCTCCTGCTGACCGTGATACGTCTCGTGATCTCAAAAAT
CTTCCTCCTGTTGCTTAATCTTCTTCATGTTGATTCATCATTGATCATTGTTATCAATCCCTTAG
GAATTTTTGTTCCTTCAGATGATTCTTTAATCAGTAGTTTTGCAGTAACCATTTGGAGATTAG
AGAATTTTATGATTTGAATAAAATGTCAGTTTCCTGGTGGAAATAGATTAATTCGAAAATGTA
TGACACCAGATGTAGAAGAAATAAAAAAATGAAGGAGTATTTTGTAAATTACTCAAAAGTT
AGTGGTCCTAAGTAGGTA

>AtbHLH035
ATTTTCTTGCAGTAAGATACAACGGCGGTGTCGTGTGTGTATAACTGGAAAATTACGTTGTT
GTCTCCCGAGACTTTGGTTTGTCTCATTCCCCATTCGCGTTCTTATATAAAGCTCACAGTTCG
AGAGAGACAAGAGACCAACGAAACGAACTAGAGACAGTTTGATTCGAAAATCTTGTCGGA
AAATGGAGGATATCGTCGACCAAGAATTAAGCAATTACTGGGAACCTAGCTCCTTCCTCCAA
AACGAAGACTTCGAATACGACAGGTTCTTTCTTTCGTCCTTTTTTTTTCCTTACTTTGCTTTC
CATCTTGAGATTTTGATATTTGTTTCTTTAAAAAAAAAGAAGCTGGCCTTTGGAAGAAGCCA
TTTCTGGGTCGTATGATTCGAGTTCGCCGGATGGAGCTGCTTCGTCGCCGGCTTCTAAGAAT
ATTGTGTCGGAGAGAAACAGAAGACAGAAACTTAACCAGAGACTCTTCGCTCTTCGATCA
GTTGTTCCCAATATCACTAAGGTTTCTCTTTTTCCCCTTGTTTTTTGTCTTCTAATTCCAGGAT
TCAAACTTATTATAGACTCTGTTTTTGTTTTCTGATAGATGGATAAAGCCTCAATAATCAAAG
ATGCTATTAGTTACATAGAAGGATTACAATATGAAGAAAAGAAGCTCGAAGCTGAGATCAG
AGAACTTGAATCTACACCAAAGAGTAGCCTTAGTTTCAGCAAAGATTTTGATCGTGATTTAC
TTGTTCCTGTCACATCCAAGAAGATGAAGCAGCTTGATTCTGGTTCTTCCACTTCTCTCATC
GAAGTTCTCGAAGTAAGTTTCAGTTCCTTTTTTCATTTAGAAATCATAAGATTTAGGAGAAG
AATTTTTCATTGAAAATTGTGTGTATTTGGGTGTGATTGAAGTTGAAGGTAACATTCATGGG
AGAGAGGACAATGGTGGTGAGTGTAACATGTAATAAGAGGACAGATACAATGGTGAAACTG
TGTGAAGTCTTTGAGTCATTGAATCTCAAAATCCTCACTTCCAATCTCACCTCTTTCTCTGGC
ATGATCTTCCACACTGTCTTTATTGAGGTCAGCATCTTTCTCTCTCTATCTCTCTTGTCTCTCT
AATTTGTAATTAGTTAATTACTAATTAGAAGCAATGATTGCAATTAGTCAAGAGTGGATAGTA
AGCTTTCCAAGAAAAAGTAAAAGGGAACCATTCAAATAGTAGATATATGATTGCTTTAGTAA
ACTTTTCTAAAAGTCTCCATCAAATTAATACTAATTATGTAATAACATGATAGTAATTTCTCTT
ATTTCTCAAATCTTTCTTAAATTTTACCAAAGTGTATTTTTTTTTTTTTGTAGTTTATCTTAGC
TTTGCAGTTTATTTGCTAATATGGGTGGCGTTGGAATTTATTTAACTAGATTGTGGAATATAAA
GGGATTTTCTGTGGCTTAAGTCTTTTGCTCGTGTCAAATACTCAAACTTATTTAAGAATAAA
TGTCTCATTCGTGACGCTGGCAATGAGCGTTGTATTATTACTTTATTTGTTTACCTGACAATAT
AAATATAGTTTTACAGATTAATTATAAGATTTCTTGATTTGATACTGATTTAGACGACATGGAC
CTTTCTGGTTGATTTAAAGCAAGATTTTATTGAGTAAACTAATGATTAAGTTAGCATCAATATT
CCACTTAGTCTATCTTCTTAGTTAATACAATTTTTTTTTTTGTTGGCCACTTAAAATGTGTTGT
TTATTTTATAATTTTGGTCACTATGTACATTGCATGATATTGAAAACTAAGTAAAAAGTATATT
CGTTAGTTCCAATATCATGCAGTTTTTGCCAATTTGTGTTAATTAAAAAGATTATGAATTTTTA
TTTCTTTTTGTCAATTTATGTATACTTATTACAAAGTTTTTGTAGTTGAAAATATTTCTTATGGT
TAAATTAAATGTTTAACGTATGGGAAGCTTCCAGTTGCGACCAAACATTTATTGGGTTGTGT
GGTTTTTAGTTTTTATGTCTATTTTTGGTCCCACAATTATTGTAATTTGGTCCATTTGGTTTATT
AAAAAGAAAATAATATTATCTCTGTGGTAAGTAGAATCTGATTGCTTTGATATTAAAATATTG

TTCAACTGCTCGAATTAAATACAATAGGATAAGAAATTATAGAAATGATAGAATATAGCTAAT
TCTGCCAAATACATCATATATTGTCTGTCTGGAGATAATAAAAGATAGCTGATTCTATCAAATA
CCTCACATAGAAAAACAAACATAAAGAGTCAATAATTATTGGATCTTTTAGAGATAAGATTAT
GATTGCATTTGCTAATTTTTTTTTTTTTTGGCAGCAGATTGCAATTGCTAATTAAATAATAGTA
CATGTACTTGATTCCTTTATCTTTAATGGGGGGAGTATGGGACCAGTCTCTTCTTTGGCTCAT
GGCTATTATGTATTTCCATTTGTAGTTGTACTGTCTCGTTTAAAGCATTGATAGAAATTCCTTT
TAGTAAAAAATTAAGAAAATATAATGAAAGTAACTTTACTTAAACTAAATTAATTTTGGATTA
TTTATCTAACAGGCGGATGAAGAAGAACAAGAGGTGTTGCGGTTAAAAATAGAAACAGGA
ATAGGAGCTTATAATGAAACTCAAAGCCCTACTTTGAGCATCGACTCTCTTTACTAATAATAC
TTTTTTTCTTCCTTTTTTGGTTCATTTTGGCTTCTCTCTTTACAATAATGTATGTCTCTCTTT

>AtbHLH036
ATTAATTTGCTCAATCTTCAAGACTGCAATAATTTTTCAACTTTACAACAAAGCCCTGAGCT
GTTCTTCTAAAGACTAAGATCACAAAATCCGCATAAGATCAGATCTTGCTGTCTCATCTTCAT
CATCAGAAGTAGAGAATCTTTTAAAGGAATATTTACTTTTATTAATTTAAGACCACCAGATGG
ATGATTGTCGGGACAAGAGAAGAAGACGGTGTACGAAGCTGACATGTGGTACTGATAATAA
CGACATGGAGAAGATGATGCACAGAGAAACTGAGAGGCAAAGGAGACAAGAAATGGCTT
CTCTTTATGCCTCTCTTCGTTCTCTTCTCCCTCTTCACTTCATCAAGGTCTCACACTCCCCTA
AAAACCCTAATCCTGATTTCGGTTCTTCATGACTTCTTAGATCTACTTAATCTTTGTCCTTTTA
TTTAGTCTTCCATGTAAGAAAATGAAAAAGGGATATAGTAACAAGTGTTTTGACTTTGTGAT
GTTACTTAACTATAGTGTTTTTCTTAAAAGGTTCCTAAATAGATCTATTGCTTTCTTGGTTTGT
TTTGTTTCTTGGCGGATTCTTTTGCTCTCTCTAGGGTAAGCGTTCGACGTCAGATCAAGTCA
ACGAGGCGGTGAACTACATAAAGTATCTACAGAGGAAGATCAAGGAGCTAAGTGTGAGGA
GAGATGATCTCATGGTACTGTCTAGAGGAAGTTTATTGGGTAGTAGTAATGGTGATTTTAAG
GAAGATGTGGAGATGATAAGTGGGAAAAATCATGTGGTGGTTCGTCAGTGTTTGGTTGGTG
TGGAAATCATGTTGAGTAGCCGCTGCTGCGGTGGCCAACCGCGGTTTTCGAGTGTTCTTCA
AGTGCTTAGTGAATATGGTCTCTGCCTTCTTAACTCCATCTCCTCTATTGTTGATGATAGGCTA
GTTTACACCATACAGGCCGAGGTAAACCTATCCTACCCTCATAATCTTAGTTACTTATTTCAG
CTGCGATATAGACATCAATTGCTCCAAAATATTTTTATTTTTTTCTAAAAATACTTTCTATTTCG
AACTTTTCATTAAATTCTTTAACAAAATGCAAATCAAAGTTCTCCATAAACCTTTCGTTTTGT
TTGGTTTTCATGTTTGTAGTTTATAAAAATGGTTGGTAAAAACAAGAATTTGTGTGGTTGCGT
TGTAAGATACACATACACACAATTTCATAACATCTCAATTTAGATTCAAAAAAAAAGAAAAG
AATTGAGGTCTTTGCTAATATATGTATATACTATCGACATTTGAATCCATCAGATTGTGGACGT
TAGTAGGAACCTTATATATCTTTCTATCTTAAGCATCAAATATTTGAAAAGAAGAAATCATAAT
TGCAACTGTCCATATATACAAGAAAATGTTCTCACATGTGGTAATTAAAATTTATATCCCTCAT
GCATTTAAATATATATATCACCTCATGGTGTATGTATTGAATTTTGAAAACTATTATATTTCTGG
AGGTTAGAGGGTGCATGTGCATTATCAAGGTATTTCAATAATTCTGTCCCAGGTTAGAGGGT
GGCATGGAATATCCAATTATAGAACAAAATGATCAATTAACTGTGCCTGTTGTTTGATTTTGT
CTTCGAATTTTCTTCAAAGCAAAAATCTTACTCGTATGAAGAGATGTATACTTCGTATATATAC
TACTATACATAAGAGTTTGGTACATTTGAGTATATATATTGAAAATTTCTTTTGTTTTGCAGGT
CAACGATATGGCTTTGATGATTGACTTAGCAGAACTTGAAAAGAGATTAATCAGAATGAAGT
AAATTCTTGTTGAGTAGGTTGCTCTTAACTTGTCAAGTAAACGACATATATAGTTAAGTACGT
ACTTTATTCGTACCTGTGATTGTTGTGTCGTGTTCACATCTATCTGTATGTATATTATGTTTATT
CCATCTGATTGTCATTAGCTCATTTAGTTGTTTATAGATCGGAAAATTATGTTTCCCATAAGCT

GAGTTAGTACTTCATGGTTATATGTTTTTTTAATATTTTATGGTTATATGCTATGATTGGGATT
TTATTTGATCAATTTCAAAAAGAAATTAAAGTTTTTAGTGGTGTCTAGGCAGTGTTTGAAGC
TGGTAGCATT

>AtbHLH037
AAGAATGGGTCACAAAGTGCTATATAAATCCATCACTTCCTTTTATATTGCTGAATCTCTGAT
TGGTAGTAACAGTTTACATTTCGCACATAACTCTCCCTCTGTCTTCCACCATCATCTCTTCTG
TGATTCAGACACACAAAGCCCTAAATTTCAAGCTTTGTCAGTCCATCTTCTTCTTCCTCCATA
CCTTATCTCACCAGCTTCTCCATATCTCTCAAAGAAAAAACAAACCCTATAAATTCCACAAA
AAAGGAGGATGGATAACTCCGACATTCTAATGAACATGATGATGCAGCAGATGGAGAAGCT
TCCTGAACACTTCTCTAACTCAAACCCTAACCCTAATCCCCATAACATTATGATGCTTTCTGA
ATCCAACACCCACCCGTTCTTCTTCAACCCCACTCATTCTCATCTCCCATTTGACCAAACCAT
GCCTCACCACCAACCCGGTTTAAATTTCCGGTACGCCCCCTCCCCGTCATCATCTCTCCCGG
AGAAGAGAGGAGGCTGCAGCGACAACGCCAACATGGCGGCGATGAGAGAGATGATCTTTC
GAATAGCCGTGATGCAGCCTATACATATTGATCCGGAATCCGTAAAGCCACCAAAGAGAAA
GAACGTGAGGATCTCTAAGGATCCACAGAGCGTGGCAGCTCGGCATCGAAGGGAGAGGAT
AAGCGAGCGGATTCGGATTCTTCAGCGGCTTGTTCCCGGTGGGACTAAGATGGATACGGCG
TCGATGCTCGATGAGGCTATCCATTACGTTAAGTTTCTCAAGAAGCAAGTGCAGTCGCTGGA
GGAACATGCGGTGGTTAACGGCGGAGGAATGACGGCGGTGGCCGGAGGAGCACTTGCGGG
TACTGTTGGTGGAGGATATGGAGGAAAAGGGTGTGGCATTATGCGGTCTGATCATCACCAGA
TGCTTGGAAATGCACAGATTCTTAGATGATGATGATGTTGATTTTTAAATATATATCATATGTTT
ATTAATATGACGGGAAAAAATATTATCGAGGGAGTTGAATTTAGTATCATGAAACTATGAGA
GCATTTTTTTTAAATGTTTTTATCTTTCCGGGTTTCGATAATGTTTGGGATGGTTAATTAACAA
TTTAAAAGTCAGACAACTTGGTTGTAAAGACTAAAGAATAAGCATAGTTTATCAATTTATCAT
TACTAAATGAAATAGTCTCTTGTCACTGATCAATGCAACATGTTTTAGCTTTCAGACATCTAG
TTAAGGTTTTGTGAGTTGAATAATAGTATTGTTTTCTGTCTTGTTTCCAATGTAAGCGTCAAA
AGTTTATAATTTATGGCATTTCGTACCTGTGGTACGTTCTTGATGCATAGGTGTATGATTTTGT
GAGCCAACTTTATAGATAACAGGAAGTTTTGGAGACAAATCGATAACACGAAC

>AtbHLH038
TATTATATAGTTTGTTGAACAATATTTAACAACAGAGATTTGTTTCTTTAGTCTTTCATCCGCA
TATATAATCAGGAGACTATAATATTCCTACCAAAACAACTTCAAGAAAAAAACAGAATCAAT
AACCTTGTCCTTGATTAAGCAAAAAATGTGTGCATTAGTCCCTTCATTTTTCACAAACTTCG
GTTGGCCGTCAACGAATCAATACGAAAGCTATTACGGTGCCGGAGATAACCTAAATAACGG
CACATTTCTTGAATTGACGGTACCACAGACTTATGAAGTGACTCATCATCAGAATAGCTTGG
GAGTATCTGTTTCGTCAGAAGGAAATGAGATAGACAACAATCCGGTTGTGGTCAAGAAGCT
TAATCACAATGCTAGTGAACGTGACCGACGCAAGAAGATCAACACTTTGTTCTCATCTCTCC
GTTCATGTCTTCCAGCTTCTGATCAATCGGTAAGATCGCTAGTAGCTACTCTGATATGTCATC
TACATCTGTTTCTAATAAGCTCGTCTATACAGCTATACAATTCTAAAGAAGAACACCGAGTTA
ACCCTTATTTCTTTTTCTTTACTTTCTTGCAGAAGAAGCTAAGTATTCCTGAAACGGTTTCAA
AGAGCTTAAAGTACATACCAGAGCTGCAACAGCAAGTGAAGAGGCTAATACAAAAGAAGG
AAGAAATTTTGGTACGAGTATCGGGTCAAAGAGACTTTGAGCTTTACGATAAGCAGCAACC
AAAGGCGGTCGCGAGTTATCTCTCAACGGTTTCTGCCACTAGGCTTGGTGACAACGAAGTG
ATGGTCCAAGTCTCATCGTCCAAGATTCATAACTTTTCGATATCAAATGTGTTGGGTGGGATA

GAAGAAGATGGGTTTGTTCTTGTGGATGTTTCATCATCAAGATCTCAAGGAGAGAGGCTCTT
CTACACTTTGCATCTTCAAGTGGAGAATATGGATGATTACAAGATTAATTGCGAAGAATTAA
GTGAAAGGATGTTGTACTTGTACGAGAAATGTGAAAACTCGTTTAACTAGGTGACTAATTCA
TATAATGGTGTGTTTATCCACTAGTTCTCATTTCTTTTTAGCTGTGTCCTTTTCTCATATGAATC
TAACACTGATCTGGACCAAATCATTTGTGTACTGAGTCAATATTGATTGAACAAAACAATATA
AACAGAGAAAAAAAAAACATTCACTGCTA

>AtbHLH039
ATATAGTTTGCGAATTTTTAAAGTTTTGGTTGTATAATAGTATAGTTTGTTAATTTATACTTAAC
CATAGAGATTGATATATTGAATTGTATATATCCGCATTATATATACGTTGGACAATAATATTCTA
CCTAAGTTCAGAACAATCCTAACCAAAGCAGCTTCCAAGCATCTAGAGAAAACAGAATCTA
CAATACTTGTCCTTAGTAAGCAAAAATGTGTGCATTAGTACCTCCATTGTTTCCAAACTTTGG
GTGGCCATCAACGGGAGAGTACGACAGCTACTACCTCGCCGGAGATATCCTCAACAACGGC
GGGTTTCTTGATTTTCCGGTACCGGAGGAGACTTATGGAGCTGTTACAGCGGTGACTCAACA
TCAGAATAGCTTTGGTGTTTCTGTTTCGTCGGAGGGAAATGAAATAGACAACAATCCGGTG
GTCGTCAAGAAGCTTAATCACAATGCTAGTGAGCGTGACCGTCGCAGGAAAATTAACTCTT
TGTTCTCATCTCTCCGTTCATGTCTTCCTGCCTCTGGCCAATCGGTAAGAAGCTACTCCGACT
CATTGATTAGACCCGTTATAGTTTATGCATCTATACAATTTAGAAGAAGAATACTGCGTTAAA
CCTTTTTTCTTTCCTTTCTTGCAGAAGAAGCTAAGCATTCCTGCGACGGTTTCTCGAAGCTT
GAAGTACATACCAGAGCTGCAAGAGCAAGTGAAGAAGCTAATAAAAAAGAAGGAAGAGCT
CTTGGTGCAAATTTCAGGTCAAAGAAACACTGAATGTTACGTTAAGCAGCCACCAAAGGCC
GTCGCGAATTATATCTCGACCGTTTCTGCGACTAGGCTTGGTGACAACGAAGTGATGGTCCA
AATCTCATCGTCCAAGATTCATAACTTTTCGATATCTAATGTTTTAAGTGGGTTAGAAGAAGA
TAGGTTTGTTCTTGTGGACATGTCATCTTCAAGGTCTCAAGGAGAAAGGCTTTTCTACACTT
TGCATTTACAAGTGGAGAAGATTGAAAATTACAAGCTGAATTGCGAAGAGTTAAGTCAGAG
GATGTTGTACTTGTATGAGGAATGTGGAAACTCATATATATGAGAATTTGGTCTTGTTTCTTTA
TAGTTATGTTATGTCGTCCTTTTTCTCTTCGAAATCTAACATTCTTGACAAAGCGTTGTAGTC
GAGTTGTTTCGTTTTTTTGGTCGAAGTCAATGATCGATTAGTTATGCTATTAAGTATAAACTG
ATTATTATGAAGTTCATGTTACAGCTATAAACTTTAATTTTCGCCTAGACTTTGAATGTTACTA
ATAAAAAATTT

>AtbHLH040
ATGGAAAATGGTATGTATAAAAAGAAAGGAGTGTGCGACTCTTGTGTCTCGTCCAAAAGCA
GATCCAACCACAGCCCCAAAAGAAGCATGATGGAGCCTCAGCCTCACCATCTCCTCATGGA
TTGGAACAAAGCTAATGATCTTCTCACACAAGAACACGCAGCTTTTCTCAATGATCCTCACC
ATCTCATGTTAGATCCACCTCCCGAAACCCTAATTCACTTGGACGAAGACGAAGAGTACGAT
GAAGACATGGATGCGATGAAGGAGATGCAGTACATGATCGCCGTCATGCAGCCCGTAGACA
TCGACCCTGCCACGGTCCCTAAGCCGAACCGCCGTAACGTAAGGATAAGCGACGATCCTCA
GACGGTGGTTGCTCGTCGGCGTCGGGAAAGGATCAGCGAGAAGATCCGAATTCTCAAGAG
GATCGTGCCTGGTGGTGCGAAGATGGACACAGCTTCCATGCTCGACGAAGCCATACGTTAC
ACCAAGTTCTTGAAACGGCAGGTGAGGATTCTTCAGCCTCACTCTCAGATTGGAGCTCCTA
TGGCTAACCCCTCTTACCTTTGTTATTACCACAACTCCCAACCCTGATGAACTACACAGAAG
CTCGCTAGCTAGACATTTGGTGTCATCCTCTCAACCTTTTTCATGTTGATATATTATATATAGAT
GCATAAAGATTCGATCCAAGATTGTATGGGTGTTTTAATATTATTATTCTAAGATATATGATGTA

CAATTGTGTACCAAGTTTCTTTATCTTGATATCATATGCATAAATAATTGGTGAATAAAAAGA
AGATATTGATTGTAAACAAAAAAAAGAAGATATTGATTGTTAATTAGGGTTTGATCATTCTGT
ATGAAAGCTTTGGCCTGCAAATTAATTTTCGATATATATATATATATATGGAGAATATATATCAA
ATACTTTTTTAATTTGACTATAATTTGTATCAATTATCTGAATCTGATGAGTGTAGGTTATATAT
GGATTAGCAAAAAAGAAAACAACCATTATTACGCACCTACATTAAAAATCATCCACCAAAG
AAGAAACCATCCTCAAGAGGGTTCCCATATTGGAGAATAATGAAAAATTTAAGAAGAAAAG
GGTCGAGATTTCGACGGACAAGCATTCTGATTATTACAAACATATTTTAGAGAAATTTAGAA
AGAAAATTAAACTAAAAATTAAAAAGAAGCAAACTGAGATAACTCTCTGTCTCAATGGAAC
GCCGGCCAGAAGTGATCATCGTGATTTTGGTTACCATCTTGATAAGGTCAATCGAGGAGTGT
ATCCGTTTCAAACACCACACGTCCACTATATTTCGCCAAGTTAAATTGAAAGGAAGATGAAC
AATCAAGAGATGTGGTAGTTATTTTGAATTAAGCATATCCAAAAAGACCCTAGGCGAAGCTG
AGAGATCGCAAGTTCGAGTGACACTTGGAACGAAACTAATATTACATGATGTGGTTTCGGG
CCTAAAGGAATTACGGGCTTCGGCCCAGAACCTCCTGATATTC

>AtbHLH041
TACTTTTCTCCACTTTAATATTTTCCTTTATTTTTGAAATAAGAAAACAAAAATATGGAACG
AATAATTTTCCCTCCCTCCATCTCTCCCTCTCTCTCTGGCTTAATACAGCAATGAAGCTTTAAT
CATCTCCTCTTATTCTTCATCTTCAATTCTCAAAATCTTCTGTTGTGTTTGGAAAAAAGCCAC
ACGCGTGATCTTCTTACATGGATGCATTCTTCTTAACGGATGATCCCAATACCCGGAACCAGT
TAATCCGGTCGCTAGCTCAATCTTTTGGATGTGTTTACGTTTGTCTTTGGTCTTACTATTTTCC
TCGACCTTCTAAGTAATAATCATATCTTCCTCGTTCTCGACCTTTACATCTATACACATTACAT
GCACCACATATATGTAACATGTTCAATCTCTCTAATGTAAATGTTTTTTTTTTATTAATCTCATG
ATGCATTTGATTTTGAGCTGCAGCTATTTAATATCAATGGATGGATATTACAATGAAGCTTCTG
AAGAACCTTCTTCGTCTTCTTCTTCTGGAAGTTTAGCTAGGAGCTTGTTCCATGAGTATCGC
CAATCCGTTATCCCGCTCCAAAATGGGTAAACTTGTTCATCAATTTGAGTCGTATATGCAATT
TATATTTACCAATATTTATGTTTTAGTAACAAGAAAATATATAATCAAAAATGTTATAATTTTAG
GCATGTGCCAAGCATGGCGTTCATGAACAATCTTCCATACGTAGAAATTCGACCACAAGAG
AGTCAAAGACTTGCTTTTAACGACACACAACGTCTCTTCTATCAGGTCATATATTGTTAGTTA
GAAGCATTACTACAACACCATTTTTATTTCAAATTATTTTTTCTTACTATGATATTCACTAACA
ATCAATTCTTCATCTATTTTCATTACTGGTCGCAGGAAGCCAGGATTCAGGTACATTATTTTCA
TCTTCATAATATTTCAACAAAATGTCACTAATATATGTAAAATTTAATGTTTTAAATGTGTTATG
GATCAGACGGTGATATTCATGGGTTGCCGGAGCGGCGAGATCGAACTCGGAATGACGTATG
ATACTACAAATGTATGAATCTTTTTTTTGTCAATTTTATAAAACCATATGCATTTATTAATTACT
TGAAGATTTTAAAATATATGTCTTTGTCTTTCTCTAAAATCTTTATATAATGGTCTCTAATGTGT
CATTGACTCAAGAACAGATGAAAATTGAAGCAAGTCTTCGAGAATGGTTCCCTGAAGATTT
CAATAGAAAGTCTTCTCCGGCAAACTCCGATTATCTCCGGCCACCTCATTATCCCTCTTCATC
GTCTTCTTCTCTTAGTCCCAACAACATCTCCGAATATTCCTCTCTTTTGTTCCCACTCATCCCT
AAACCTTCAACGACGACTGAGGCCGTTAACGTTCCGGTACTTCCACCGCTAGCTCCGATCA
ATATGATCCATCCACAGCATCAAGAGCCTTTATTCCGTAACCGTCAACGTGAGGAAGAAGCA
ATGACGCAAGCAATCTTAGCGGTTTTAACGGGGCCATCAAGTCCTCCGTCAACTTCTTCCTC
GCCGCAGCGTAAAGGAAGAGCCACCGCTTTTAAGAGATATTACTCCATGATTAGTGACCGC
GGTAGAGCCCCGCTTCCGAGTGTTCGGAAGCAAAGTATGATGACAAGAGCGATGTCCTTCT
ACAATAGGCTTAACATTAACCAGAGAGAGCGTTTTACTAGGGAAAACGCTACTACACACGG
CGAGGGAAGCGGTGGAAGTGGAGGGGGTGGACGTTATACTAGCGGGCCAAGCGCAACGC

AACTGCAACATATGATATCGGAGAGGAAACGGCGAGAGAAGCTTAATGAGAGCTTTCAAGC
ATTGAGATCTCTCCTTCCTCCCGGAACTAAGGTATGTGAGATTTTTTTTTTCATGTATATACTA
TGATTTCTGTTAATTAATGTTTTCAAAATTTGATAAGTTTCTTCTTCTAATGTATATTTGAGCTA
ATGTAATGACTAAATTTTTTGAACAGAAAGATAAAGCATCGGTCCTCTCCATTGCAAGAGAG
CAACTATCTTCTTTGCAAGGTGAGATTTCGAAACTACTAGAGAGAAATCGGGAGGTAGAGG
CAAAGCTAGCAGGAGAAAGAGAGATTGAAAATGATTTACGACCCGAAGAGAGGTTTAACG
TTCGTATAAGACATATACCTGAATCAACATCTAGAGAGAGGACTTTGGATCTACGAGTTGTT
CTAAGGGGAGACATCATTAGGGTTGATGATTTGATGATAAGACTTCTCGAATTCTTGAAGCA
AATCAACAATGTGAGCTTAGTGTCAATCGAAGCTCGAACTCTAGCTAGAGCAGAGGGGGAT
ACTTCAATTGTTCTTGTGATCAGCTTAAGGCTCAAGATTGAGGTAAAGTATCATCTTGATTAT
ATTAATTTGGTTTCTGTTAAGGAAACATATTATTTGACATTTATTTGTGATTTATTGATTAGGA
AAAGTTGGCATATACAGTATGTGATGATATAATATACAGTAAAATATACCAAAAAAGGAAATT
TGCTATGATTGCTACATTTTAAATTTAACAATTTAGTGGGGTTTTATTGAATGCATCGCATTGA
TTGGTTAGAGTTTAGTAGTGAATTTATATATTCACATATATACATATACACTTTTTAAAGGTCG
AGAATCTCTCTTCCACATAAATTAATTTGATAGAGTATTATACATAATTATTTGATAAGATAAGT
AGATAACCACTAGTTTGGTCTTGACTCTTGACTATAAAAAGTTTATCGAATAAAACTTACGG
AATTATTAACTGATTTTTTGTAGGGTGAATGGGACGAATCAGCCTTCCAAGAAGCAGTCAGA
AGGGTTGTTGCTGACTTGGCTCACTGACAACAAGTTTGACTATTTCGTAACTATATACTATAT
AGTAATTATTAAATTTCTATTCCTCTTTTACGCTTACAAATTCATTTTTAATGATCGAAAGATT
AAATTATTTATGGACAGTCTATTTTTTACTATGACGTGTGGCGGCTCCTATCGTTGTCGATGGT
TCTTATTACTGTGGAATTTTTCAGCCGTTCGTCATATTTCTTGAATCAATATTGACCGTTTGTT
TTTATTCAGTTTTTAGCTATTACCGTAAATTT

>AtbHLH042
CAACATGTTGCAAAAGCACTAAGATGATATACGTATACATGCATATTGCAAAAATCAGTGGT
CCCATACCATTTTAAGTCATCATGAGCGTATGAGAGTAAATTCTTCTCACATATTAATAACAA
CCCTTCAAAGTTATAAGATTTTTAGAGAGAGAGCTACCACGTTTTCGTATCTCCGGGAACGA
TGGATGAATCAAGTATTATTCCGGCAGAGAAAGTGGCCGGAGCTGAGAAAAAAGAGCTTC
AAGGGCTGCTTAAGACGGCGGTTCAATCTGTGGACTGGACTTATAGTGTCTTCTGGCAATTT
TGTCCTCAACAACGGTTAGTTTCTTATCTCTATAGTTCATTCATCTCTCACAATACAACCATAA
TATCTATATCCGTATATATTATGTAAACTATATGTTTAAATGAATTCGGCTAATTGTTGGCTTGT
CGTAGTGTTACAATTCGATCTTGAAATAATTTTTTTTTTTTTTGAAGGTTTTACAAAATTTATT
AAGATTTTTGAACTACTTATATGAAAGTATCATTGTTTAGGGTCTTGGTGTGGGGGAATGGAT
ACTACAACGGTGCAATAAAGACGAGGAAGACAACTCAACCAGCGGAGGTGACGGCAGAA
GAGGCGGCGTTAGAGAGGAGCCAACAGCTCAGGGAGCTTTATGAGACACTTTTAGCCGGA
GAGTCAACGTCAGAAGCAAGAGCATGCACCGCATTGTCACCGGAGGATTTGACGGAGACA
GAATGGTTTTATCTAATGTGCGTCTCTTTCTCTTTTCCTCCTCCATCTGGGTAAACAACTCTC
TCCCTATACTCAAGTTTCTAAATTTGTTCTTTCTTTTTTTTCCTTCTCTAAGTGAAGACAAAA
ATAGTATTGTGTGTTAAATGCGAATATCGAAAATATTGTGTTGACATTAAAGAGAAACTGAG
GAAGTTGAGTTACTAGAATAAGAAAGAAATGAGAGTTAATGAAACGTACACACACTACATT
TTCATGAACATTATTGCGACCGTTGAGATTCTCATTGTTTGGTGATTGATTATCTAAAGTAGA
AGCATGAATAGATATAACATAAATGCATAACAAAATGGGTTAGTTATGGGTATACTTCATGCT
TTTCTCTATGTGGTAAAAAAAAATCAAATATAAATTTGGGAGGTAGTAATTTGTAGGATGCCA
GGAAAAGCGTATGCAAGGAGGAAGCACGTATGGCTAAGTGGTGCAAATGAAGTTGACAGT

AAAACTTTTTCTAGAGCTATTCTCGCTAAGGTAAATTCTTTTATTCATTCACCAACTATACTAT
GCATCTATTTTTACCTATTTATGTATACACTATACTATGTATCTATCTACTTGTAAACCTATTTAT
CTATACTTTTATACATTTTCACTGATTAACTTGAATAATTCTTCTTGTCGTTTTATGGGCCTTTT
ACGTCGGTTCCTCAATGACTTAGAGTGCTAAAATTCAGGTAAAATTTGTCTTTATTAGTTTGC
TCTTTTTAGTAAGATTTGAGAATTGTATTCTGATGAAAATGTATTTTAAAAAAGCAGACAGTG
GTTTGCATTCCAATGCTTGATGGTGTTGTGGAACTAGGCACAACGAAAAAGGTAACGTTGT
ATATACTCTCTCTTTTATTTACTTGTTAATGAAATATATAAACAAAATAACAAAGTGAATATAG
CCTATCTTTCTTTAGGTTTAGTAATGTTAAATTAAGGAGATGCACTACAAAAAAAAAGGTTGTT
TTACATCATTTATTATAAATATTTGCATTAGTTAAAAGTGATGTAAAAGAATTTTGTATCACTT
AAATAAGTGACTTAAATATTGTTGATGCAAATAATTTGCATCAGTTAATTAGTAACTGATGTA
AATAATCAAAATAATTACATCGATTATTATAATTGATATCAATATTGTATCAGTTACTAAAAATG
ATGTAATGTTTACATCAATTTATTTAAGTGATGTTAGTATTTGTTTCGGTTTAAATAACTGATTT
AATTAATATATATATAATTATTAATACAATGTTATTAATATTTCAAATAATTATTTTATTTATTTCT
TTTTTTTTTTTAAATAACAGATCATTGTGGATAATTACTCGTTGGAATATCTACTAAAATTTTAT
CAAGATTTAATAAAATATTTTACATGAACAATAAATCTTTATATAAGATAAGCAAAAAAAATAA
TCATAATTTGGTTTTGGTTTCTTCGTGTTTAAACACATCTTTCTTCTGTTCCATGCTTCTTCCT
TCCATTAACTGTTGGTTCTTCTTGAAAAATACCATTTCTTCTTCTATGCCATCTCTCTTCCATT
TACAGAATCAAAATCATACTTTGGTTATAACTTATATCTCTTGAATCCTCATCACACTTGAGTT
TCTGCTGTACAAAATAAAAAAGAGAGTAATTGTTTGAAAATTTACAGAATTGTTTTGGTCGG
AATAAGAATAACCATTTGTAAGTATTTTTAAAAGAAAGTGATAGAGTACCTTTACTTTGTGTT
TCTCCAAACTGTGTAGGAAAACAAACAGTCCTAATGATCAATGGCAGATTTCTGAAAATAA
AAGAAAAGAAAAAAACTGAGCAATGGTTAAGAAAAAGAGAAAGATAGAATGATAGAAAA
ATAAGAAAGTACTTGTCGTTGAAGATGAAGAATTGAATGAGAGTTATGAGATTTCAAAAACT
AATTTGAAGATATTCTAAAAACGTTTTTGGATACAAAACTGTAAAAAAAGATCGTGAGCGTA
AAAGCAGGATTTATAGTGAAGGAAACAAATATATTTAGTTAGCTGATTTTGAGGGAAAATAT
AGGAATTAATAATAAATTATTAACCCAATATGATTATTTATTTATGGTAATAACAATTTCTCGTC
ATCTACAATAAATTCAAGTTAGTTTTTTGTTTAAATTAAAATACAGAAAAACTTATTAGCATC
ACTTCTTATTGATGTATAATTTTATATCATTTTTATAAACTGATGTTAATAAAATTAAGTGAATA
CATCGGTTATTTAATTGGTGTAAATACAGATTTTTGTATCAATTTGTTAAAATGATGCAAACTA
ATACAAATTTCTATCAATTTGATTAAAGTGACGCTAAACATGTTAATTTATACATCACTTATTAT
AATTGATGTTTAATTATTTATTTTGTGTCATTTGCTTATAAGTAATTTAAACTTGTACCATTTAT
ACTTGTGATACTATTTAATTGATGTAATCTATCCTTTTTTTTGTAGTGATGGATCAATATTTATT
TGTTGTATTTGTGTGTGTAGGTAAGAGAAGATGTAGAGTTTGTTGAGCTCACAAAGAGTTTC
TTCTATGACCACTGCAAGACGAACCCAAAGCCGGCTCTTTCTGAACACTCCACCTACGAAG
TGCATGAAGAAGCCGAAGACGAAGAAGAAGTAGAAGAAGAGATGACAATGTCAGAGGAA
ATGAGGCTTGGCTCTCCTGATGATGAAGATGTTTCCAATCAAAATCTACACTCTGATCTTCAT
ATTGAATCAACCCATACGTTAGGTATATATATCTACCCTAAGCTACTTATATTTTGCGGATTTAT
TTCGCTATATCTAAGAAATTATCATTTGTATATATATGTAGACACACATATGGACATGATGAATC
TAATGGAGGAAGGTGGAAACTATTCTCAGACAGTAACAACACTTCTCATGTCACACCCCCAC
AAGTCTTCTTTCAGATTCAGTTTCCACATCTTCTTACATCCAATCATCGTTTGCCACGTGGAG
GGTTGAGAATGGCAAAGAGCATCAGCAAGTGAAAACGGCGCCGTCGTCACAATGGGTGCT
CAAACAAATGATCTTCAGAGTTCCTTTCCTCCATGACAACACTAAAGATAAGAGGCTACCG
CGGGAAGATCTGAGCCACGTAGTAGCAGAGCGACGCAGGAGGGAGAAGCTGAACGAGAA
ATTCATAACGTTGAGATCAATGGTTCCATTTGTGACCAAGATGGATAAAGTCTCAATCCTTG

GAGACACCATTGCGTACGTAAATCATCTTCGAAAGAGGGTCCATGAGCTTGAGAATACTCAT
CATGAGCAACAGCATAAGCGGACGCGTACTTGTAAGAGAAAAACATCGGAGGAGGTGGAG
GTTTCCATCATAGAGAATGATGTTTTGTTAGAGATGAGATGTGAGTACCGAGATGGTTTGTT
GCTTGACATTCTTCAGGTTCTTCATGAGCTTGGTATAGAGACTACGGCAGTTCATACCTCGG
TGAACGACCATGATTTCGAGGCGGAGATAAGGGCGAAAGTAAGAGGGAAGAAAGCAAGCA
TCGCTGAGGTCAAAAGAGCCATCCACCAAGTCATAATACATGATACTAATCTATAGACCCTA
ACTTTATTGATGCCAACTCTAGAGAAGGATAATTAAGCGTATTTTTGTTTTAGCCTCACATGT
ATTAAGACATCAGTTACATATATAGCCGGATGCAACATATAAATGAAAATGTACTAGATGATAT
TGTTCATTTGTCCAATGTAGTACTTGTGTATGATGCAATTGCAACATATAAATGCAAATGTACT
AGATGACGATGTTGTTCGTTGTCCAATTTAGTACTTTTGTATGAATGCGATATTTGGATGCAG
AT

>AtbHLH043
CAAGCATTAAAATTTTTACGAACCAAACAAACAAAAATTATGAATAATTATAATATGAACCCA
TCTCTCTTCCAAAATTACACTTGGAACAACATCATCAACAGCAGCAACAACAACAACAAGA
ATGATGATCATCATCATCAACATAATAATGATCCAATCGGTATGGCCATGGACCAGTACACAC
AGCTCCATATCTTCAATCCTTTCTCTTCTTCTCATTTCCCTCCTCTCTCTTCTTCCCTCACAAC
CACCACTCTTCTCTCCGGAGATCAAGAAGACGACGAAGACGAAGAAGAACCTCTAGAGGA
ACTCGGTGCTATGAAGGAAATGATGTACAAGATCGCAGCCATGCAATCGGTTGACATCGAC
CCAGCAACCGTCAAGAAACCCAAACGCCGTAACGTGAGGATCTCCGACGACCCTCAGAGT
GTGGCGGCTAGACATCGCCGTGAGAGAATCAGTGAGAGGATCAGAATTCTTCAGAGACTCG
TGCCAGGTGGCACTAAAATGGATACGGCTTCAATGCTCGATGAAGCTATACGCTATGTCAAG
TTCTTGAAACGGCAGATCCGGCTACTCAATAATAATACCGGATATACTCCTCCGCCGCCGCA
AGATCAAGCTTCTCAGGCGGTGACGACGTCATGGGTTTCACCGCCACCACCGCCAAGTTTC
GGCCGTGGGGGAAGAGGAGTAGGAGAATTAATCTAGACAAGATGACATTTCCATTAGTAGT
AACTAAATTATGCTATAATGTGTGAGTAATGGTGCAATTATGGAAAGTGTTTATATATAATAAA
AATGGATACGAAATAAGGGTTTGTTAGGATATATAATATATTGTTGTTGACCGTGAAAAGGTT
GTGGAATAATCATAACGGTTAGCTTGAGTTGTGTACCACCATATATTTAATATTATGTTATCAT
TTCGTATATAATAATAAGTTCTTGATTTGGAGTTTGGTTGTATACAAGTTTTGGCCGGGCATTT
TTCGGGCATAAG

>AtbHLH044
AAAGAAGTTGTCAGAAAAAAAAAAAAAAAAAAAGTGTCAAGAAGATCATATCTCAATTCATT
ATATTATGGCAAATTTCGAGAATCTTTCTTCTGATTTTCAGACAATAGCCATGGATATATATTC
TTCCATAACTCAAGCTGCAGATCTAAACAACAACAACAGTAACCTTCATTTTCAAACATTTC
ATCCTTCCTCTACTTCTCTCGAATCGCTCTTCCTTCATCATCATCAACAACAATTACTTCACTT
TCCCGGAAACTCTCCAGACAGTAGTAACAATTTCTCTTCAACTTCAAGTTTCCTCCATAGTG
ATCACAACATCGTCGATGAGACCAAGAAGAGAAAAGCTTTGTTACCTACTTTGTCTTCATCA
GAGACTAGCGGCGTCTCCGATAATACGAATGTTATTGCCACTGAAACAGTAATTGATCTCGA
AACCCTAGCTAATTATCTTATCATTTTCATAACTTATCATTAAAAAGTTCGTTTAAAATAATTA
CTTTTATTGTCTAAAAAATATCTAGGGTTCTTTGAGAAGAGGTAAGAGGTTGAAGAAGAAG
AAGGAAGAAGAAGACGAGAAAGAGAGAGAAGTTGTTCATGTGAGAGCCAGAAGAGGCCA
AGCCACTGATAGCCACAGCTTAGCAGAACGGGTAAAATTGCTTAAATTGTCTATATAAATTC
ATTTTTTTTATAAACGTGGTGTATTCATTTAAATTTATATACATGTCTACACTCTATATCAAAAT

TTCAGGTTCGGCGAGGGAAAATAAACGAGAGATTAAGATGCTTGCAAGATATGGTGCCCGG
ATGTTATAAGGTAATAATGCAAAGATAAATATAATAGTTTAACATTTTCAACTTTTCATTACTT
CTTTTTTAATATATTTGTAATCAAAATAGAAACTAATGGCCATTAAACAATAAATAGCATATGA
AAAGGTCATCATCGGCATTAATTGTCACTAATTATACTTGAATCAAATTTAAGCATTTTATGAT
AAAAGTGATACTTTTAGTTAAACAAGAACATATGTATCCAAAAGTATTTTTAACATGCATGAG
TGTGATTTTAGCTTTTTTCTTTGATACCGCTCATAATATTTTCCGAAAATTGTTCTTCGGATTT
TTTTTTGTTTTTTTTGTTTACAAAGAACTCAGAAAAAAAATTGTAGAAAAAGTGATGTGATT
TTCTATTGATTTAGTGGTGCTTACTTCTTATAATGAAAGTGTATGTTTGGTGGGTGCAGGCTAT
GGGAATGGCTACGATGCTTGACGAGATAATTAATTATGTCCAGTCTCTACAGAATCAAGTCG
AGGTAAATAATTTAAGTTGTAATCATAAACTACATATATCAATAAGACTTTGTCTTCTTGTATT
TTGGAGAAAGAAAAAAAAAAACTAGAAAATGGAGGTTTCATGAGATGGGAGGTTCGGTAAA
TACCTACTTTAGTAGTACTTTCCGAAACACGGCAAATCTTAGATATTCGAAAATTACAAAAA
AAAAACGAAAATCTTACCACACACTTTAAAAAACTTCCAAAACACAAGCTTTAATTTTATAA
ATTACTCAATTTTGTTTCATGTTTTTTGTGGATTTCAGTTCCTCTCGATGAAACTCACTGCAG
CAAGTTCGTTTTATGACTTTAACTCAGAGACAGATGCAGTTGATTCCATGCAGGTACTAGAA
CATTCTAATAATGTTTTTCAATTCTTAAGATAAGCATATATATAATTAATTAGTGGTAGTCTGGT
AGAGATGTTGTAATGGGTCCGGTATGTTTTGATCGAGTCGGTTCTACAAAGATTTATTCAAA
AATACAAGTGTTTGTAGTTTAGATTCAATCTTATTAGACTATAATATATGATCACGCGGACTAT
ATGACCCGCAATTAAAATATGTTCGACAGGTCAAATCCAGCCTTTGCATAATTGATCTTGTAT
GGTTTATTGGTTTAAGTAGTCTCTTTTTTTTTGGCATTATATTTGCATGGTTTAAGTCTAACTA
GTTGTTGTTATGACAATTACTTTATTTTAACGTGATAAAGTATTTCTATTAGTATTGAATAATAT
GAATGGAATACAATTGGCAGAGAGCAAAGGCACGTGAGACAGTGGAGATGGGGAGACAA
ACAAGAGATGGGAGTCCTGTCTTCCATTTATCAACATGGTCCCTTTGACTTTTGTTTTCTCTT
TTCTCTTTTCCTTCTTTTTTATTGTTTTCGAAATTATTCTTCTATTTATTTGGATGCGATATGTCA
TATATACGACCCAACTTGGGATAATATTTGTAATCTGTATACTTGTAATTTCGACGATATAAATT
TATTGGTGACTTATAATTGTCATGTAAATATCTCAGTTGTCCTCTCCCTTTTCGCTTTGCTATTT
CGTTTGCTTGGCACCTTTTTCCTTTTCCTCTTATGTAATTGTCCTCTTAAGTTATGAAATGATC
AGAAA

>AtbHLH045
TGTCGAACAAAGTGAAAACATAAGATCATCTTCTTCGTTGATAGATCAATATAGGAACTCCA
GAAGAGAATCTTGATCAATTAAGTATCATGTCTCACATCGCTGTTGAAAGGAATCGAAGAAG
GCAAATGAACGAGCATCTTAAATCCCTTCGTTCTTTGACTCCTTGTTTCTACATCAAAAGGG
TAAATTTCATATTTATCTGTTCGATCCCTTCATAGTCAATTAGCTAGTTTGGATGATATAGAAA
AAAGAACTCAATTCACAATCCTAATTATTTTCTCGAAGAGATTGGTATTTTTTTAATGATCGT
TGCTTTCTTTTGTTGAAGTTATTTCGTAGAGGTTGATTCAAGATTTATTCCAATTTATTTATAC
AGACAATTGAACCTTCTCAAATTGCTTATTTAATAGGATCCCAGATAAAATTGTGCAGGAAC
CAAAAATCTCTTAGATACTTATAATTGGATCATGCATGATAATCTTTCTAATATTGGATTTCCC
AACTTACTATTTTTGGAAGAAAACAAAATGTTGTGGCATACATATATAGTTAATCCTATATTCT
CACTGTCAGACTTCCTTGTTTGTATATGTTATGCATAGACTGTATATTTCTCTTTGGTAACTTT
CTTTAAACAAACTACTCGGATGCACCAATCTGTTTAATAATCATGTGGTCTTGTTTTCTATGA
TTGATCGGTATTAGGGAGATCAAGCTTCGATCATCGGAGGAGTGATAGAGTTCATCAAAGAG
TTGCAGCAATTGGTTCAAGTTCTTGAGTCCAAGAAACGTCGAAAGACCCTAAACCGACCAT
CTTTCCCTTATGATCACCAGACAATCGAGCCATCCAGTTTAGGAGCCGCCACTACCCGAGTA

CCGTTTAGTCGAATCGAAAATGTGATGACCACAAGTACTTTCAAGGAAGTAGGAGCATGCT
GTAACTCCCCTCATGCTAACGTAGAAGCAAAGATTTCAGGTTCTAATGTTGTATTGAGAGTT
GTCTCTAGGCGAATCGTGGGGCAGCTCGTAAAGATCATCTCTGTCTTAGAGAAGCTATCTTT
TCAAGTTCTTCACCTCAATATTAGTAGCATGGAGGAGACTGTCTTATACTTTTTCGTTGTTAA
GGTACATATCTTCATCTACATACAAATTAAATACATTGTTAGTCAATATATTGAACAACCGATA
AAACTCATTATATCTATCATGAAATTAGATGGAATTTGGTGTTATAGAACTATTACAAAGTAA
AATGCTCCTAATGAATGATCAAACACCAATTATTGCTAAACCGATGTCTTGCATCGATTTAGT
TACTTAACCAAATTATGACTATTTAACTTGATTTGTACTTCTTTAATTTACACCAAAAGATATA
TCAATATTATTAGTTAAGCTCAATGTATATATATATGGATCAGATAATATTTGTATGAGTCTTTT
GTTTTGAATTCGACGGAATTAAATTTACATGTGCAGATAGGATTGGAGTGTCACTTAAGCTT
GGAGGAGCTAACTCTTGAAGTTCAGAAAAGCTTTGTGTCTGATGAAGTGATCGTCTCTACC
AATTAAAAACAAAATTCTACATGTACTAGAGCGTGTATCGTTTTTTGGGATTAATAATCATATA
ATCGTTACATGAGCCTTGATACTTTGCTAGAAATAAGCTCCTCTAAACAAAACCTTCTTTTTA
AAAAAACACACTTATGTTTTACTTAGTTTGTTGTTGTATCCGAAGTTGATCAACGTTGTAATT
TCCCACAATAAATCATGACATTTTATATGCTCTAATTAACTAGTATAATATAGTTCTGA

>AtbHLH046
ACATGAGATTCTTAATTAATCATACATATCTTACCATTTTAATTAGTTAACTGATTTCCTTTTGA
TTGCATTTACTTCGACGCATGATAGTAGTCGGCGTAATTTCAACAGTACCTTGACACGCCAA
AAGCTTCACGTAATTTGCTGTCGAAGAAGGTACGCTTCTTAGCTCAAATCTAATCCATTCGT
GTTCTTTAGAGATTTGACTTGGATTCTAGTTTTTGTATCTCCATGTTCATTGGTTGACTTGAAT
TATGCGAAATTGAGTTGGTTTGAGCTATGAATTCCGCTGAATTATGGTTATTCTCTTGAATTTC
TACCCAATTCTGATGACAGATCTATCGATTCCACGAATTTGGTGACTTCTGCCGATTCTCCCG
TTTGAATTCTGTGGAATTCGCTGAGAAAAGTTGCGGCGGAGGCTGATTTAGGTTAAATCGA
GCAGAATTTGACCAAATTCGAGGATTTTTATCGGTAAAAAGATACAGAGAAAGCATAATTCA
GCAACTTCAGAGCTCAATTCCGCTTCTTCAACCATCGAAAACCACAATATCTCTTGAATCAG
CTGCTACAAGTCGCAGATCTGAGCTGCTAGCCAATTTTTGCGATAATTCCCGCGCACAGATT
AGTAATAGAATAATTTAAAAAGAATTCAAAAACCGTCGAAGCTCTGTCGTTCATGGAGCTTC
CTCAACCTCGTCCCTTCAAAACCCAAGGTAAACACCATTATCAGATATTATTGTTACCAATTC
CTTTATTTTATTTTAAAACAGAGTGTTTAGTTTCTACAAGATTCATAATTTTAAATTTATGTATT
TTTTTGAATTTTGATATATATATTAAGAAGAATTTCGCACAGGGAGAAAACCAACACATGATT
TTTTATCGCTCTGCAGTCATTCAACCGTCCACCCAGATCCAAAGCCAACACCACCACCTTCT
TCTCAAGGTATTTTTTTTTACCATTTTACCCTTTTTTCTTCTTTAAGATTCTCTTAAAAGGGGT
CACGTTTTTTTCGCGCGTGCATCTGATCTGACTTTAAGACGAATCATGGATCAAAATGTTGCT
ATTTTGTTGGGTCCTAATGGGCCCATCTGTGTTTAAAGCCCATGGAATTTTGGGACCGTTTTT
TTCTTTCTTTTTCGTCTACACATGTCGTTAAACCTTGCCTTCGATTAAATAAATATCTTTCAAC
CCTCATGATGTTGATCCCTAGAATTGCATCACATAAATAACAAAATGTTGTTAATTACTATAA
GCGTTTAAATGCATACAACTTGATGTATAAGAAAATGTGTATTATCTAGGGATTTACTTCTCTA
ACATATAGTTTATGTTTTTAATTTTGATGGTACGTGAAGCAGGTAGTCACTTGAAAACCCATG
ATTTTCTTCAACCGTTAGAATGTGTTGGTGCTAAAGAAGACGTAAGTAGGATTAACTCTACC
ACTACAGCGTCCGAGAAGCCACCGCCCCCTGCTCCACCTCCACCGTTGCAGCACGTGCTTC
CCGGTGGTATAGGGACTTATACGATTAGCCCCATTCCTTACTTCCATCATCATCATCAGAGAA
TTCCTAAGCCGGAGTTGTCACCACCAATGATGTTCAATGCTAATGAGAGAAACGTTTTGGAT
GAGAATTCTAACTCTAATTGCAGCTCGTACGCCGCTGCATCAAGCGGGTTCACTCTGTGGGA

TGAATCTGCTTCTGGGAAGAAGGGACAGACAAGGAAGGAGAATAGTGTTGGGGAGAGAGT
AAACATGAGAGGTAACAAGAAAACTTGAATCGTGATGTTTATTTAGACTAGTTTTAAAGTAG
TATTTAGAAATCAATGGAATGATGAAATATAAGTTTTTTTGAATGTGTAGCTGATGTTGCAGC
AACTGTGGGACAATGGCCAGTGGCGGAGAGACGGTCCCAGTCTTTGACAAACAACCATAT
GAGCGGTTTCAGTTCTCTCTCTTCCTCTCAGTTAGTCTCATATCTTCTCTTTTTTATATAACTC
GGTCTATGGACTTTAAAGAGTGATCAACTAAAGTTTTACTTTGGTTGCAGAGGGTCTGTGCT
TAAGAGCCAGAGCTTCATGGACATGATAAGGTCAGCAAAAGGAAGTTCACAGGAAGATGA
TTTAGATGATGAAGAAGATTTTATCATGAAGAAAGAAAGCTCCTCGACTAGCCAGAGCCATA
GAGGTTTGTATTGTCCGAGATTCCACCTATATGATATCTAAAGTCTAAACAGTAACAGCTTAG
GATCTGATTGATTGCTTCTTTACAGTGGATTTGAGGGTAAAAGCAGATGTGAGAGGCTCTCC
TAACGATCAAAAGCTGAACACACCTAGGTCTAAACATTCTGCTACAGAGCAACGGAGAAG
GAGCAAGATCAATGATAGGTAAGTCCTGAATATGTAGCAAAAGCCACCAGAATTGGTTATAT
AGCTTAGCAGAGTGGAGATACTAATCAAACATTGTTAATGGATCCCTCTGCAGATTTCAGAT
GTTGAGACAACTAATACCTAACAGCGACCAAAAGCGGGATAAGGCCTCCTTCTTACTAGAG
GTATAACTATACTCTTTTGGCCGAAAATTTTGTGACAGTAAGATTCAATCACATCTGCTCCTA
ATCTCCAATTTTTTTTTTTGACAACTTCATACAGGTTATCGAGTATATTCAATTCTTACAAGAG
AAAGCAGACAAGTATGTGACCTCTTACCAAGGATGGAATCACGAACCTGCGAAGCTATTGA
ATTGGGTAAATATAATCTCACATTTTCCGACCATACTTCATTTTTATTCCCCGGGTTGGAAACT
GAAAACAAACAAACCATGTAATGTTAACCCGTGTGATCCATTTACATTATAGCAGAGTAACA
ACAACCAACAGCTAGTACCTGAAGGAGTTGCTTTTGCTCCAAAATTGGAAGAAGAGAAAA
ACAACATTCCGGTTTCGGTCCTTGCAACTGCACAAGGTGTTGTTATTGATCATCCAACAACC
GCGACGACCTCTCCATTTCCATTGTCGATTCAAAGCAACAGTTTTTTCTCTCCTGTGATTGC
GGGTAATCCCGTACCCCAGTTCCACGCAAGAGTCGCATCATCAGAGGCTGTAGAGCCAAGT
CCGAGTTCCCGGAGTCAAAAAGAAGAAGAAGATGAGGAAGTTCTTGAGGGTAACATCAGA
ATTTCAAGTGTTTACTCACAAGGGTAAAACTAAAAAACTCTCTTTTGAATTTTGATCTTGCA
ACAGTTAAGTAAAAGTAAAAAATTATTAACCTTTTATACATTTACTTTTGTGTGTGGAGCAGA
TTAGTGAAAACACTGAGAGAAGCATTGGAGAATTCAGGAGTGGACTTAACGAAAGCAAGC
ATCTCCGTTGAAATCGAGCTCGCTAAACAATCCTCGTCTTCTTCCTTCAAGGTATCTTTTTCT
TTACTTACGTTTGATTCAATTCAAACACATTTTCATGAGTTGGATCTTGATAGTTTATAGTTTT
GTTCACATCTCGATAGGATCATGAAGTTCGTGAACCGGTTTCTCGAACCAGAAACGACAAT
GTCAAGCAAACTCGGAAACCAAAACGGCTCAAGACGGGACAGTAGAAGTTATAAAAACAA
AACCCAAACACACAATCCGGTAAAAGTTAAACACCAAAATTGATTCCTCTGTTTTTTTCTTT
AAATCATTTTATCTCAAAAAGGGATTATAATTTATTATTATAGTTATATCTTTGATTTTTTTCTTT
TGTACATCCGAAATCCTGTTATATATATCTCGTGGGTCCATAATCGTATGAGACCTAAGAAGA
CTAGGGCGACCCGGTTATGCTAACCGCATTTTAGAACATACTCTTTTGAGGAATATATCGTTA
AGGAAGGCTTTCAAAGTTTTCTGGAAGCGTCCAGTGTTAAGTGAAAACATTTTTCACTCTC
TTATATACTTGATCATCTCACACTTTTTATATTTGTTTCTTCCTCTTACTTTGTTCTATGGTTTTC
AATGTAATACTAGATATCTTTTATGTATTTGACTAAACCAAAGCGCATGAACGATTTTGAAAA
AGAAAAAGAGAAACACACTTAATTATAATGCCAATCAAAAGCCTGTATGACCCAACTCTCT
AGGACAAAGTCTGGAGCACGGCAAAAAGATTAAATCAAGTGTACTTTAGCAACAAGAGCT
ATCTTGTACTATTATTTATGCAATGTTGTTAGCAAAATAATCAAATGTTATGTGGGG

>AtbHLH047
GAGAAAGGCACATGTCGAACTCACGTGTCCACTTAAACGCACCACCTTCTTCTGTGCTCTC

CACGCGCCTCCACACGCCCTCCTCAACTTCGGACCTTCTTCCTCCAAAAAAAACAATTTCA
CTCTTCATTTTCTCAGATTCTCTTATTATTTCTTCTACTCGTTTCGAATCCTAATCGTCTACAGT
TTCCGACGAGATTCTCACCGGAGAAAGAAAGGTAGTTCTTTTCTCTGAATCTGTTGTGTTGA
GAACTTAGCCGTAATTTTTGAGAATTATTCTTCACTATTTGATTTGACAAGAATATGGGTAAT
AAAGACAGAATCTAAAACACTTGCTTCAGTTTTTGAGGAATCAAAAATCACACGAAAGTTT
TTTTTTTTTTTCTGCTCCTATGTTTCTTGGTGCGTTTGTGTTTATTTGACATTAGGAGTGATTG
ATTGATTATAAAGATGAATGATTTGTTAGATTCATTAAGATCACTGTTCTTTTTGATTAATCAT
CTTATGTCTAAATTTTAGGTGACTTGACAGAGAATCTTGTTCTGTAATAAAAGCAAAGATGG
TATCGAAAACTCCTTCTACATCGTCTGATGAAGCAAATGCTACTGCAGATGAAAGGTGATTC
TTAAGGAGACAATTCTTGGAAATAGATTATGGAACCTTTTATTTTTATTTATGGGGATTGATTA
TGTTTGGTGTATAAAACAGATGTAGAAAAGGTAAAGTACCGAAAAGGATCAACAAGGCCGT
TCGTGAGAGGCTTAAGCGTGAGCATTTGAATGAGCTTTTCATTGAATTAGCCGATACTCTTG
GTAAATTTCTCATCTTTCCGTAGATTTGAATGAACAATTGCTCGAGTGTGTTGTGATATTTTG
AATGTGATTGTGATATCTAACTGTGAACATGTTTCTTTGTTGCAGAACTGAATCAACAGAAC
AGTGGGAAAGCTTCTATACTATGCGAAGCTACTCGATTCTTGAAGGACGTGTTTGGTCAAAT
TGAGTCTCTTAGAAAGGAGCATGCTTCTCTCCTATCTGAATCTAGCTATGTAAGTCAATTTCT
ATATGCACATTGATTGTGTTTATGTATTCTGTGACGTGAATCAACAACTAAGACGAATGTTGC
TTCAAGAAGTTACTTCATCTTTGTATTTTTCTAAATGGTCTTGTTGATGGAAACTGGTGTTGT
GTGTAGGTAACCACAGAGAAGAATGAGCTCAAGGAAGAAACATCAGTGCTTGAGACTGAG
ATTTCGAAACTACAAAACGAGATTGAAGCTAGAGCGAATCAGTCGAAACCTGACTTGAACA
CCTCTCCTGCACCCGAGTACCATCATCATCATTATCAACAACAACATCCTGAACGTGTATCTC
AGTTCCCAGGACTTCCCATTTTCCAAGGCCCCGGCTTTCAACAATCTGCTACAACTCTTCAT
CCTCCTGCAACAGTTCTTGTCCTTCCAATACAACCTGATCCCCAGACACAAGATATCTCAGA
AATGACTCAAGCGCAGCAGCCTTTGATGTTTAATAGCTCAAATGTGAGTAAGCCATGTCCAA
GGTATGCTAGCGCGGCTGACTCGTGGTCTTCTCGGCTACTTGGAGAGCGGCTGAAAGCCAG
TGAATGAGGTCTTGAACGGCTCCACATGGAGTAGCAACGCAAGTTATAAGACATGGCAACT
TATGAAGGACTCTGGTTTTGTATTTTTTAATACATCGTTCTGATGTCCTTGTGGGTTTGGCCTT
GGCTATTTCTTGTTTTTGATGGTTGTTCATAGGATCAGGAAGTTATGGCCTTGATGGGTCGTT
TGAGATAAATAAGTAAATTAGCTCTAACCATTGTCATTTTCTTTTGTCTTAAGATTCTAAAAA
CACAGAAACAGAACAAAAAAAATCAAATTTCTTTTTAAGAAAAAAGCTT

>AtbHLH048
AAAATTTGAAAATTTACTCACCAGAGTCTATAAAAATTAAAAAGGAAAGGAAAAAAAAAG
AATGAATCATAGTGAAATGACCTTATTTATGGCTCCAACTATAGTGATTAGTAACCACTAAAC
CAGAAAAAAACAAGTGACCCATTACACAGGGATTAAAAATACTACCGAATAAAGCCCAATA
GGCCCAATTCAAAAGCGTCTCTCTATCATCGTCTCTCCAAACCCCATAAATTGGATATTTCCT
TTCTCTAAAAATTTGGCTTTATTAACAGACGCACTCCAAAGCTAACGTTGCTTTCTTCCCACT
CCAAATGGCTCTTGTGCTCTCAGAACACACACAGATATAATAAACTCTTAACGAAGAAGAA
GACTTTTTCTTTTCTTGAGCAAGCTCTGAGAAACTCACAGAGAGGGACAGAGAGAGAAATA
TTTCATTCGCTACTCTCTTTAATACCGCCATTGATGCAACCTCCTTCGCGTTAACTGTCAAAA
TACTCCCCAACTGACAAAAATTAAATTCCTTTTTTTCTCTCTCGATTTTAAAAGCTCTAAAAG
GAACGAGTTGATCAAGTCGGTCGGTTCTGGTTATCACTGAATTTCATTCAACTCGGTTTATTC
CGGTTTAAGTGTGATTTTCCCGGCTAATGGATCTGACCCAAGGTTTCAGAGCTAGATCCGGC
GTTGTTGGGCCGGTGGCCGGACTAGAATCTCTCAATTTCAGCGACGAATTTCGGCACTTAGT

GACGACGATGCCTCCGGAGACTACTGGCGGCTCCTTCACGGCTTTACTCGAGATGCCTGTG
ACTCAGGCCATGGAGCTTCTCCATTTCCCAGATTCATCGTCTTCTCAGGCCAGAACCGTTAC
CAGTGGTGATATTTCTCCTACCACTCTTCACCCTTTCGGGGCTTTGACTTTTCCTTCTAACTC
GCTTCTCTTGGACCGCGCCGCTCGTTTCTCGGTGATTGCTACAGAGCAAAACGGTAATTTTT
CCGGCGAGACTGCTAACTCTTTACCTTCTAATCCCGGCGCGAATCTGGACAGAGTCAAAGC
CGAGCCTGCTGAGACCGATTCAATGGTGGAGAATCAGAACCAGAGCTACTCTTCTGGAAAA
AGGAAAGAACGCGAGAAGAAGGTGATACCAATTGTTCACACAATCAAACTTGTTAATTAAA
CAAGAGAGATTAGTCCCTAATTTCTTTATTCCTTAATTTCTAGGTCAAAAGCTCGACGAAGA
AGAACAAGAGCTCCGTGGAGTCAGACAAGTTGCCATATGTTCATGTCAGAGCTCGTCGTGG
TCAAGCTACTGATAACCATAGCTTAGCAGAGAGAGTAATTCACAATCTCACAGACATGGTTT
GTATTAATTAATTAGTGGCAATATGCTAATTAATCATTAACTCTTGATTATAGGCAAGAAGGGA
GAAGATAAACGCACGAATGAAGCTGCTACAGGAACTGGTCCCAGGCTGTGATAAGGTTTGT
CTTTAGATAATTGACCTTTTAATCAAAAAGCTAATCAATAATTAGATCTTCATTTGTGCTTAAA
GACAGAATCTTTCTTTATTAAGACCAACCCCCAAAAAGTGATTTTTTTTCTATAATTTGATCA
TGTGGTTTGATAAGGGTTTAGTGCTAAATTCCTCTTATATGAAAAAGGTTTTGTGGTGTGAC
AAAGAACAAAACCACTAGTTTGGTAATTTCACTGCCTTGAAGATATGTAAACTTTATTTTCG
TGTGGGGAAGAATATCTCACGGGTCACAACCATTTAGGTAAACTAGTAGTAGTAATACGGAA
GTGATGTGACATTGCTCAATAGATTCTTGTTTCTTCTTCCCAAAGTGTATTCATTTGAGCTTG
AAGCTTACTCCATAAATGCCGATAATGGATTATATGATGTGGGAATGCTAGCTGGACTGTTCC
CTTAAGGTTTTGGGTAGAGGGGACAGATTTTGGTGGATAGATCAAGGTCAGACTGTGTTTT
GGGGTCCATCTTTTGATAATAAGTGGCAAAAGTTGCAGGCTTTCTATGGAGGGTGAGGAGT
GTAGTATCGTCTCTAATAAGTTTGGTATCTATCAAGTAGAAAGAGGGTGAGGAGTGTAGTAT
CGTCACTAATATTGTTATATGCTTTGAGTCCCACAATACCTAATTGGTTGGATACATGATGTGA
CACTCCTGCAGACAGCTAACTAATCCATAGATATCTTGGAATTTAGCTGCAATTATTGGTGGA
AGGAGATGATGATAAGTGACAACTCTTTAAGAGTTTGTCAAATTTGTGGGGTTTTGGGACTT
GATTGTTTGTTTCATGAAATCCTGAGAAATCCTGAATCCATGTTTGTAAACTACAGATGTTGC
TCTGATTCGCTTTTCTGGCATATTATCTTGAAAAGAGTAGAGTTGTTGATTTTATAGAAGCCG
TTATACATATGCAACTTAGGATTAAGGCTCATTTTCACACTGCTTCGTTAACCACATATATTTC
TAAAAACTATGCCTTGTTCTTCTTTCTTTCTGACTGACCAGAAAGCTTTGGCCTGCAGATTC
AGGGCACTGCATTGGTTCTGGATGAAATCATTAACCATGTGCAAACTTTACAGCGTCAAGTG
GAGGTAGGACTTCTTGCACTATCTTTTGTTGTTGTTTACCTGAAGGTCCTGAACTGAAACAC
TAAGCTACACAATGTATGATAAAATTGGTGCCACAGTGTCACATAACCGTTTTTCTTCTTTTC
AGATGCTATCTATGAGACTTGCTGCAGTAAACCCCAGAATCGACTTCAATCTAGACAGCATA
CTGGCTTCAGAGGTAAGTATCAACTAATCATATAGGAGAATTAAGGTTGCATGTGATCTTCTT
TTACAATAAAAACTCTTAAATTCAATGTTCACTGGGAAAAACTTCCCTTTGACCATGTTACT
CGTTTAAGTAACCATAGGGATTATGTTTCTTTGAGTAACCTTTTTAAGTTGTATCACAAAAAG
AGAAACTTTTGGATATTTATTGATCCAACATTAATCGACCCTATTCTAGACTAGGCTTAAAGA
AGAAGAACAACGTGGGGGAAATAAATACTTAGACCTTACAAAAATGCAGTACAAACAGTG
AGATCAAGGACTCTAAAAAATCTTTTGAAAGGACTCATTAGTTCTCTCAATTATATCATACAG
AGAGATAAAAGTGGTCAGAAACACGGACCACCTTTGTTCTATGGTCTATGATGCTGTCTTTT
TTTTTTATCCTTGCACAATTATTTTTCTGTGGGTCTCAGTCTTAAGTTATTGTTGCTTCAAGTT
TCACCTATCTAATCTAATAATCAACTTTTGTTTCTCTGCAAATAACTTTCTTATTAAATGACCT
TCTTCTTTTACAGCTTGTACACCATTTAAGAACCAGACCCCACTCATTAAAAACCTTGTTCTT
CTAGGATGAGTTACAGTAAAATAGAGCTGGCTTGTTGACTAGATTGAGAATCTAGGATGTTG

GATCAATCATCTCTAAGAGAAGCTGATATGTTTTACAAATTGTGTTAATAATGATGAGGTCCA
ATTATCTTTTCTGCAGAACGGCTCTCTTATGGATGGTAGCTTCAATGCAGAGTCTTATCATCA
GCTGCAACAATGGCCTTTCGACGGCTACCACCAGCCGGAATGGGGAAGAGAAGAAGATCA
TCATCAAGCCAATTTCTCAATGGGTTCAGCTACTTTACACCCAAATCAGGTGAAAATGGAGC
TCTAAGTTGGAAACAGTTTGACATGCTTATGTGTATATATAAGAGTTATATATATCTACTAAAG
TTTCGATTGTTGAATTAGAGGTAGAAAATGGTTATGATTGTTTCAGGTCTTTGTTCATGGCCT
TTCTATATTTACTAGATTCCTCAATGTAGAATTCAGAATGTCTAAATTGCGTTTCCCTTGAGA
GAAGTATATGAAGATTTGATATGTTACTGAGC

>AtbHLH049
CTTTAGCTCACAAAAAAAAAATGTCTCTGCAAATTATAATCCTTGAATACTTTGGCTATGGCG
GTGTTTATGTTTAATGATCAAAAAGCTCTGTTTTTATGCAAGCGTGGGTATAAAATCCAGTT
CCTTTTCCGATTTTTTGTTCTTCCTTCTTCTTCACTTCCTTTTTTTCTTCTCCATTTCTCAAAA
AGGCCACTCCTTTGCTTATCTTTCTCTCTCTCTGCAGCTGAAGAACAGAGAAAAAGGGTTTT
TTATTGTCTGACCCAAAGGACCCAGTTTCAATTCAGCTTTTGTTGTTAGAAATTGAGGTAAA
AGATTCAAAATTTGTTCAGAAAAGTGGCACAGATTCTGAAAAATTAATATCTTTCTTCTCAC
TCTTTTAGCTCGATTCTAAGCTTAAATCTGGTTTAGGGTTTTTGGAGTTTGTACTGATAAAGC
ATGATCCTTTTTGTCTTCCGTTGACTGATTCTGTTCCTTTTCTCAATATATTGATTTGGGCTTC
TCTTGTTCTTCATCGAATCATTGGTCTTCAAGGTGAATGTGTTTGTGTAGTGGCAAGTCTTGT
TGGTTTGTAGTGGTTTTGATCTTGGAGATTTTCTGATTAGTGAGTGGTGTTCATCAAGTATTA
GAAGAAGATGGATTTAAGTGCGAAAGATGAGTTTTCAGCAGAGAAGAGGAATCCTGATAAC
TATGATTCTGTTAATAATCCGTCTGGAGATTGGCGAGTTGATTCATATCCTTCAGAGAATCTG
ATTTCAGCTGGTCCTGCGTCTTGTTCTCCTTCTCAGATGATGGATTCATTTGGGCAAACTCTT
TGGTATGATCCCACGAGTGTTCAGGCTGTTGGTTATGCCGGTTTTAATGGTGGTAATGCTTCG
TCTTCTTCGTTTAGGGGTAGTATTGATAGATCTCTTGAAATGGGTTGGAATCTGCCTAATTTG
TTGCCTCCTAAAGGCAATGGTCTTTTCTTACCGAATGCGAGTAGTTTCCTTCCTCCGAGTATG
GCTCAGTTCCCGGCTGATTCAGGTTTTATAGAGCGTGCAGCGAGGTTTTCGCTCTTTAGCGG
TGGGAATTTTAGTGATATGGTGAATCAACCACTTGGGAATTCTGAGGCTATTGGTTTGTTTCT
TCAAGGTGGTGGAACAATGCAAGGGCAGTGTCAAAGTAATGAACTTAATGTTGGTGAACCT
CACAATGATGTATCTGTAGCAGTGAAAGAATCAACTGTTAGATCTAGTGAACAAGCTAAACC
GAATGTTCCTGGATCGGGCAATGTATCCGAGGATACTCAGTCTAGTGGTGGTAATGGTCAGA
AAGGCAGAGAAACCTCTTCCAACACAAAGAAGAGGAAAAGAAATGGGCAGGTGAAGATT
TGAAGTTACTCGTTTACTTTTGTTCTCTCCGCCTTATTATGTTTCTTTTTGACATTCGTAATCT
TTTCTGTTTGTAGAAGAACTCTGAAGCAGCTCAATCACACAGATCCCAGCAGTCTGAGGAA
GAACCAGACAACAATGGTGATGAAAAGCGCAATGATGAGCAAAGTCCAAATTCACCTGGA
AAGAAGTCAAACAGTGGGAAACAACAGGGCAAACAAAGTTCTGATCCTCCAAAAGATGGA
TATATTCATGTACGGGCACGAAGAGGCCAGGCCACAAATAGCCATAGTCTTGCAGAAAGAG
TACAATTTCTAAAACTATCTTGGAAGTTTAAGTCGAATAGAAGCTATCTCTTCTTGTATTTTTC
ATCTAATTACTTTGCGATGATAGGTTAGGAGAGAAAAAATTAGTGAAAGGATGAAGTTTCTT
CAAGATCTAGTACCGGGTTGCAACAAGGTAAGGTCACTTTAAGTGTTGATAATGATGGATAT
TAATATGTAAGAAGAGAGACAAAGCTAATTCAGAAAAATGAATGTGTGTTTGCAGGTGACT
GGGAAGGCAGTTATGCTTGACGAAATCATAAACTACGTACAATCACTACAACGCCAAGTTG
AGGTATGCCGTTTCCTAATTCTCAACGAGGTGCTCTTTGTGTTTGCTTCTTGTAATCAATTCT
TTTGCTCATTTCTCTTTTGCTTAAACAGTTTTTATCGATGAAACTTGCAACTGTGAACCCACA

AATGGACTTTAACCTTGAAGGTCTTCTTGCAAAAGATGTAAGATATTTTAACTAATTCTCCTA
ATCATTTGATTCGTCATAAGATTCTTATAAACTGAATTCTATGCGCAGGCACTTCAACTACGA
GCTGGTTCTTCATCTACAACACCATTCCCACCAAATATGTCAATGGCTTATCCTCCTCTACCT
CATGGATTCATGCAACAAACTCTTTCCAGCATTGGAAGGACCATTACCTCTCCATTGTCTCCT
ATGAATGGTGGATTCAAGCGACAGGTATGTATTAGCGGTGTGGAAATTGAACAATTCCGAGC
TCAGAATCTGATTTTGTTGGTGATTATTTGTGTAAAATAGGAAACAAATGGATGGGAGGGTG
ATTTGCAAAATGTGATCCACATTAACTATGGAGCTGGTGATGTCACACCTGACCCTCAAGCA
GCAGCTACAGGTGAAAACTGAAACCTCTTTTACTTTCTTTTTTCACCAAGCAATAAGAATTT
TGTTTGGTAAAACTTCTCTGTAATTTTTTTTATAACAGCATCTCTTCCAGCTGCAAATATGAA
GGTTGAGCCATGATATTTCGTCCTGTTAGATAGAATATTAAATTATTGTAATCTTTTTTATTTAT
TTATTTAAAGTTGCTATATGTACTATATTGTAATGATGTTACTATTAACGAATACGATACTTAGA
CTAAACTACTACTAAGCGTTTGAGATTAGTGGAAGAAGAGTATAAACTGTGAACACCAACA
ATGCAAAATAGTTTTGTATAAGTCAATAACATTTTAGTTTGGTCTGCTATCTCAATCTTTGTAT
TGTGGATTCGTTGTTACGACGGAAAATGCCTGATGCGGCTATATGAAAATATAACTCGTTATA
AAATTTTGCCAAATAAATGTTATGATG

>AtbHLH050
ATATATAAACATTGCCACTCGCTCGAAAACAGCCACTTTCTATTTTCACCAATTTTCAAAAAA
AAAATAAAAATTGAAACTCAGAAATGGCGAATCTCTCTTCTGATTTTCAGACATTTACAATG
GATGATCCCATAAGACAACTAGCAGAACTGAGCAACACGCTTCATCATTTCCAAACATTTCC
TCCTCCTTTCTCTTCTTCTCTCGATTCTCTTTTCTTTCATAATCAATTCCCTGATCATTTTCCCG
GAAAATCTCTCGAGAATAATTTTCATCAAGGGATATTCTTCCCTTCTAATATCCAAAACAACG
AAGAGTCTTCTTCACAATTCGATACCAAGAAGAGAAAATCATTAATGGAAGCTGTTTCTACG
TCGGAGAACAGTGTCTCTGATCAAACTCTCTCTACCTCTTCTGCTCAAGTTTCCATAAATGG
AAATATTTCGACAAAAAATGTATTTTATTAACCTAGAAACCCTAATTTCACACACATATATTAG
AGTACTCTCAGTTTCTATGAATTAATCTAACTTTGTTTTTCTCTTTTTTCTTATTGATATCACAG
AATTCTTCAAGGAGAGGGAAGAGGTCGAAGAATAGAGAAGAAGAGAAAGAGAGAGAAGT
TGTTCATGTTAGAGCTAGAAGAGGCCAAGCCACTGATAGCCACAGCATAGCAGAACGGGTA
AAATTGATTTTCTCTATACAAATTATAGTGTACATAAATAATATAAATGAAAACTATGAGTAAA
AAAATTAAAATTAACTTTCAGGTTCGACGAGGGAAAATAAACGAGAGATTGAAATGCTTGC
AAGATATAGTCCCCGGATGTTATAAGGTGATTATATTTTGCTTAGACTTTAAGAAAATATATTA
TTAGTTCTTTTATTGAATATATATTTGGTGATGCAGACAATGGGAATGGCTACTATGCTTGATG
AGATAATTAATTACGTCCAGTCCTTACAAAATCAAGTCGAGGTATAACAATAACAATTAATTT
CTCTTAAGTTATAGGTCATCTAACCAATTCCATTCGTTTCAATAATTATACTAATTTACTTTCTT
TTTTATGTGAACTCAGTTTTTATCTATGAAGCTTACAGCAGCAAGTTCGTATTATGACTTTAA
CTCGGAGACTGATGCTGTGGAATCCATGCAGGTACAGAATCTAGAATTATAACTTTTAATTA
AAATTTTCTCGTAAGACTGGTGATGTTTGCTATGATTTATTTATTTCTTAATTTAACTATATGGT
TTGATGTGATTATAATTAGATGATTTAAATATCGATTTTTAATTGGAATATTAAAATGCGCAGA
AGGCAAAGGCACGTGAGGCAGTGGAGATGGGTCAAGGGAGGGATGGGAGTTCTGTCTTCC
ATTCATCATCGTGGACCCTTTGACTTTTGTTTTTTGCTCTCCTTTTTTTGTCTTTCTAATTTTCT
TCCTTTTTTTGGATTTAAAAAGATACTTTTAAAGATTAATTTCAAGAAATCTCGATGCCATAT
GATATGTATACTTGTGTAACTTCGACATTACAAATATATATACTGCCTCGAGATTGCATTCAAA
GTGGGGATACATAACGATCAAAATTTTACCGAGCAGACAAA

>AtbHLH051
AAAAAAAAAAAAAAAAAACTTATAACTTCTTTTTAAAATACAAAAAATAAAACTTATTAAG
CAATTCTTTATTAGGTTTCTCGTGGCGATGGAGAATTCTTACGACTCAAGCAAGTGGTCTGA
TTCTACAACTCCATATATGGTCTCGTGGTCACTACAATCTGAGTCCTCTGATTCTGATTGGAA
CCGGTTTAATCTTGGCTTCTCTTCTTCCTCCTTCGGTGGTAATTTCCCGGCTGATGATTGTGT
CGGTGGGATCGAAAAAGCTGAGTCACTTTCAAGAAGCCACCGTCTAGCGGAGAAAAGACG
CCGTGACCGGATAAATTCTCACCTCACTGCTCTCCGGAAACTTGTCCCCAATTCCGACAAG
GTATGTATGACTCTCACAACTGAAATACTCTCGGTTCAGTCATGAAACTTCAATTTGAGCAA
ACCGGTTTATATCTGATTTCGGTTAAAGAATTTTTAATTCGAGTTGACCGGTTTATTTTCTAGC
TGGGTTATGTAAACCGATTTTGACTTGACTCTACTGCAGTTAGACAAAGCAGCTCTATTAGC
AACAGTGATTGAACAAGTGAAAGAACTCAAACAAAAAGCAGCAGAATCACCAATCTTCCA
AGATCTTCCAACAGAAGCAGATGAAGTAACTGTACAGCCTGAAACTATCTCTGACTTTGAA
TCAAACACAAACACAATCATCTTCAAAGCTTCTTTTTGCTGCGAAGATCAACCCGAAGCAA
TCTCAGAGATTATTAGAGTTCTCACAAAGCTTCAACTCGAAACAATCCAAGCAGAGATAATC
TCTGTTGGGGGAAGAATGAGAATCAATTTCATCTTAAAAGATAGCAACTGCAATGAGACTA
CAAACATAGCTGCTTCAGCAAAAGCTTTGAAGCAATCTCTCTGCTCTGCGTTAAATCGAATC
ACATCTTCTTCTACGACTACTTCTTCAGTTTGCAGAATCAGAAGTAAAAGACAGAGATGGTT
CCTCTCTTCTCATTATTCTCACAATGAATAAATTTCACAATATACATATACATAGTATAACACA
AACCTCATTATTACTAAAGGTTTTTTCCGCATTTTGGTTTAGATTTGTTCCTACTAAACCAATC
ACGATTTGTATATTTCAAGATTTGTATAACCTAAATCTAAATCATTCCATAAGACCTAAGCCA
AGAATAACATTATTCGTAATAAACTGAGTAGATAGATTAAGCTTTAACATTAATTGAGAATCC
ATAACAATCCAACAACAAGGAATAACTTAACTGAAACAAGTTCAGACAAAAGTACTTAGGA
TTTAAGCGAAAAGACAGAAACAGGGAAGGTCTTGAAGATGAGTCTACTTAGATCGCTGTCT
CATC

>AtbHLH052
ATGATTATCCCTGAAACTGACAGTTTCTTCTTCCAAGAGCAACCGCAACATCAGCCATTGTA
TCCCGACGAAGCTCTTTCACCGTCTCTCTTCGGGTTTGATCACTATGATCATTTCTACGAGTC
GTTTCTTCCATCTCAAGAAATCTTCCTTCCTAGCCCTAAGACCCGAGTCTTCAACGAGTCAC
AGGAATTAGATTCCTTCCACACGCCAAAACACCAGAAACTCATTGACTCCAGCTTCCATTTC
AACAGTCACGACCCTTTTTCCCCTAGCCCTGAATCTAATTACCTCTTGGATTCTTACATCACA
GAAGCTTCCAACATTTCCAAGTTTCAAGCTCCTGATTTCTCATCGACGTTCAAGGTTGGGTG
GACTGAACAAGGCGACACCAAGAAGCGAGAGCTATCTGCTCAGAGCATCGCAGCACGAAA
GAGGAGAAGAAGAATCACAGAGAAGACTCAAGAGCTGGGAAAACTAATCCCTGGAAGCC
AGAAACACAACACTGCCGAAATGTTCAATGCCGCAGCTAAATATGTCAAGTTCTTGCAGGC
TCAAATTGAGATTCTCCAACTGAAGCAGACCAAAATGCAGGTATAAGATAAACCCTAAATCC
AAATGGTTCCTTGCAGTATTTTCCTTCCTTTTTTTATACGATACTTGTTTAAATTTTGCAGACT
CTGGATAGTTCAAAGGTGGGAAGAGAAATGCAGTTTTTGCTTGGTTCTCAAGAAATCCAGG
AGAAGCTATCTACAGAAGAAGTGTGTGTGGTTCCAAGGGAAATGGTTCAAGTCCTAAAAGC
CGAAGAATGCATCTTGACAAACCCTAAGATTTCTCGAGACATTAACAAATTGTTGTCAACAA
ATCTGATGAATTAGATTTGTCTATGTTCTGTCTGTTTCTAATTTGTTCCTTGGTAGTTTCAATAT
TAGTTAGAGTTTAACCATTAGTTAATTGTCTTCTTAACTAATGTAAATTCGTTGGTTTTTTAAT
CCAAAAGTGCTATGTACTCTGTT

>AtbHLH053
GCTCCTCAATGTCCATGGATTGCTTAAGCTACTTCTTTAACTACGATCCTCCTGTCCAGCTCC
AGGATTGCTTTATTCCCGAGATGGATATGATTATCCCTGAAACCGATAGTTTCTTCTTCCAATC
TCAACCGCAACTGGAGTTTCATCAGCCATTGTTTCAAGAAGAAGCTCCTTCACAGACCCAC
TTTGACCCTTTCTGCGACCAGTTTCTTTCTCCGCAAGAAATCTTTCTCCCTAACCCTAAAAA
CGAAATCTTCAACGAAACACACGACCTCGATTTCTTTCTCCCCACGCCAAAACGCCAGAGA
CTTGTTAACTCCAGCTACAATTGTAACACTCAAAACCATTTCCAGAGCCGTAACCCGAATTT
CTTCGACCCTTTCGGCGACACTGATTTCGTTCCAGAATCTTGTACCTTCCAGGAGTTTCGAG
TTCCGGATTTCTCTTTAGCTTTCAAGGTAGGCCGGGGAGATCAAGATGACTCAAAGAAACC
GACGCTTTCATCTCAGAGCATCGCGGCTAGAGGGAGGAGAAGAAGAATTGCAGAGAAGAC
TCACGAGCTCGGAAAACTCATCCCCGGTGGCAATAAACTTAACACCGCCGAGATGTTCCAA
GCCGCCGCTAAGTATGTCAAGTTTTTGCAGAGTCAAGTTGGGATTCTCCAACTGATGCAGA
CCACAAAGAAGGTAATAACCAACCCCAAATAAGAACTTTATCATCCAATTGAAACTCTAATC
GTGTTTTCTCACAAGCTTCTTAATTTGTTTACGCAGGGTAGCTCTAATGTGCAAATGGAAAC
TCAGTATTTGCTTGAATCGCAAGCAATCCAGGAGAAGTTATCAACAGAGGAAGTGTGTTTG
GTACCGTGTGAAATGGTTCAAGATCTAACAACTGAAGAAACCATTTGCAGAACCCCGAATA
TTTCTCGAGAAATCAACAAGTTACTGTCTAAACATCTGGCTAACTAGTTTTAGTTTCAAGCC
TGAAGTTCTCTATGCCTAAATTTGTGTCTGTTATCGTTGTTTTGTCTTCTTAGTTAGTGTTTTG
TCTTGTTGATTTAGGGGCTAATTATCCTGGTTAATCTCCTCTTAACTGGGAACTAATACTTCAT
CTGTATTAGTCTATGTATTAGCTTTTGTCTGACATTGTTCTTGTTAGTTAATGTATGTCCGCAT
GTTGAAGCTTTCTTACATTTAAAAGAGATCTTTGTGTAAAATCAAGCTTTTATCCATAAGAAT
TTTGGGGCTTTATTA

>AtbHLH054
CACAAGTTGACTGATCGTGTTCATCATCATGCATGGCTTCGTTTCACTTATTTAATTAACGAC
TTGTTCACGACACAAATGATTGTACCAGTTTAAGAATCTCTTGATCTCTCTTCATCATCACCA
AATCTTCCTTGAGAAATATTCTCTCCATCATCAAGTGTCTCCAACCTATATATATATATATATAG
TTGCACAAGTTCAAGAATTATCATAACAACAAACTAACAAACAACAACAACAACAACAAG
AAAGGCAAGAGTTGATCAGTTAGAGCGATGGACGTTTTTGTTGATGGTGAATTGGAGTCTC
TCTTGGGGATGTTCAACTTTGATCAATGTTCATCATCTAAAGAGGAGAGACCGCGAGACGA
GTTGCTTGGCCTCTCTAGCCTTTACAATGGTCATCTTCATCAACATCAACACCATAACAATGT
CTTATCTTCTGATCATCATGCTTTCTTGCTCCCTGATATGTTCCCATTTGGTGCAATGCCGGGA
GGAAATCTTCCGGCCATGCTTGATTCTTGGGATCAAAGTCATCACCTCCAAGAAACGTCTTC
TCTTAAGAGGAAACTACTTGACGTGGAGAATCTATGCAAAACTAACTCTAACTGTGACGTC
ACAAGACAAGTAAGTGGAAGATCCAATATATAATGTTTACTTTCATTCACTACTACTACGCAA
TAAACTAAATAAAATATCATTAATTTGTGGGATATAGGAGCTTGCGAAATCCAAGAAAAAAC
AGAGGGTAAGCTCGGAAAGCAATACAGTTGACGAGAGCAACACTAATTGGGTAGATGGTC
AGAGTTTAAGCAACAGTTCAGATGATGAGAAAGCTTCGGTCACAAGTGTTAAAGGCAAAA
CTAGAGCCACCAAAGGGACAGCCACTGATCCTCAAAGCCTTTATGCTCGGGTTAGAAAACA
TACTTTATATTACTTCGGTCTCAAAACCAAATGACAATGAGTAAAGTCGTTCTTAAGTGTGTT
ATCAATTGTTGCAGAAACGAAGAGAGAAGATTAACGAAAGGCTCAAGACACTACAAAACC
TTGTGCCAAACGGGACAAAAGTCGATATAAGCACGATGCTTGAAGAAGCGGTCCATTACGT
GAAGTTCTTGCAGCTTCAGATTAAGGTATGTAGATTGTTAAAAGGATTTTTGTTTTTAGTCA
GAATCTTATCATAACTCATGTTCAAGTTAACTAACCAAAATGCATATTTTATAAATGTAGTTGT

TGAGCTCGGATGATCTATGGATGTACGCACCATTGGCTTACAACGGCCTGGACATGGGGTTC
CATCACAACCTTTTGTCTCGGCTTATGTGAAGGACTTACAAGTTACAATGTCAATGTAAAGA
AACCCATTTAGAACATATATATCCCTATTTGAGCCAAACTAAAGAGATCTACGCTAAGATCCT
ACCATTTTCTTCTTGTTGTTCATTCTGATATTTTTATTATTCTTAACTTCTTTTGTCTCTTTCGT
CATTATGGTTCTAAGAACTTTTGGTACTCTTTTTCTTTTCTTTTTTGTAATTATATTTTTAAATT
ATGAAAAAGTCAACTCAGACTAATTAAATAACAATCGTCTTTCCTTTTGCAATTTGAA

>AtbHLH055
TTCATATAGTACCCAAAAATTCTCTTATTACCAAAACACTCCCATAACCACCAACAAGAAAA
TAAAAAGGGCATGAATTTTCCAGATTCTTCATTGTTCACACCAAATTTTGCTTATGAAAATGA
TTTAGATTTCTCTAGTTTGATCACTCCTTCAACGCGTGTATCATTCCAAGAACCTAAACCATG
TAATCCAGTCATTCATAGTGCAGGGATTGAGAACGATGGAAGACAAAACTGTGAGACGACC
ATGACATTAAGCGAAATCATGAAAGGAGACGATGAGCCGAAGAACAAGAGAGCTAAACAT
AAAGAGCTTGAGAGACAAAGAAGGCAAGAAAACACATCTCTATTCAAGATTCTAAGATATT
TATTGCCATCTCAATACATAAAGGTAAGTAACATTTTCAAATTTTTCTTATACAAATTCTGTCT
CATCTCATGAAGTCAAAAAATTTCTATAGATTATATAGAAGATCCCCTTAGTCTAATCACGGT
AATTAATTAACAATTGTGTAAATAGGGTAAACGTTCCTCGGCAGATCACGTTCTAGAAGCAG
TGAATTACATCAAAGACTTACAGAAGAAGATCAAAGAGGTCAGCGAAAAAAGAGATAGAA
TCAAGAGATCTATTACTCATCCATCATCAAGAGGAGAATTTTCAATAAGATCATTAGCATCAT
CAACTTGTTCTTGTGTTGGAGACACGAACATTGCTGTTGTGGTTAGGCCTTGTTTGATCGGA
CTAGAGATCGTAGTAAGTTGTTGTAACAGACACGAATCTTGTCTATCAAGTGTTCTTCAACT
CCTGGCTCAAGAGCAATGCTTTAATATTGTTAGTTGCATCTCAACTAGACTACACCAAGGAT
TCATACACACCATTGCTTCTGAGGTAATTATCTATAATCTATCTTCGATCTGATTTATGAAGAA
AGAGGGAGTTAATCTTTATATTTTATGTGATGAGAGATAAATATTAATATTTTGGGAAATTTCT
TAAACTTGTGTAGGTCGAGGAGGGAATAGAGGTTTATTTCTCAGAGCTTCAAGAAAAGATA
ATCAAAATTGGAACCTCAAGAGTCACTACTCGCTAGTCAGAGTTTATTAAACCTAAAAATTT
TATGAATTTACGTCCTAATTAAGATATATATCCTCAACCATTCCCACCACCACAAGCATGTTGT
GTTTTTAGTTTTTAGAAGTTTATATTGTTCAGGATGTGGATGCTGTTTGAATTGTTGACTAATA
CAAAAAAAATTATGAATTGTTTTTTTCCTATAACAAGTTGTATGAATAATTTGATTAATGCAA
AATTTTGTG

>AtbHLH056
TGATATAGTGGAGCTCTTATGGAAGAGTGGCCAAGTCGTTAGAACCAGTCAAACACAGAGA
CCCTCCTCCAATACACCACCATCTCTTCCTCCACCACCCATTCTTCGTGGTAGCGGAAGCGG
CAACGGAGAAGAAAATGCCCCGCTTCCACTTCCACAGCCTTCACCTCCCCTCCATCATCAG
AATCTTTTCATTCTGGAAGACGAAATGTCTTCTTGGCTTCACCATTCTCACCCCGGCGTTAC
GTCCACCCCGGCTTCTTCTGTCTCCCTGCCACCACCACCCAATGCTCCGTACAGCTCCGGTT
TTTTTTTTCCTCTTTTAATTCTCTTACTTTATGCAATTTGATGAGTCTGATGCTAGGGTTTGGA
TTTGGTTTGAAATTAGGCGTGAAGATGATATAGTGGAGCTTTTATGGCAAAGCGGCCAAGTA
GTTGGAACCAACCAAACACATAGACAATCCTACGATCCTCCTCCCATTCTCCGCGGCAGCG
GAAGTGGCAGAGGAGAAGAAAATGCTCCCCTTTCACAACCTCCGCCTCACCTGCATCAGC
AAAATCTCTTCATTCAAGAAGGCGAAATGTATTCGTGGCTACACCATTCTTACCGCCAAAAC
TATTTCTGCTCAGAACTTCTCAACTCCACTCCGGCTACTCACCCGCAAAGTTCCATCTCTCT
GGCACCACGTCAGACTATCGCCACGAGAAGGGCGGAAAACTTTATGAACTTCTCGTGGCTA

AGAGGGAACATATTTACCGGCGGTAGAGTTGATGAAGCTGGACCGTCGTTTTCGGTGGTAA
GAGAATCGATGCAGGTAGGCTCGAACACGACCCCCCCTTCTTCTTCTGCCACTGAATCATGT
GTAATACCAGCTACAGAGGGCACCGCGAGTCGAGTGTCGGGAACTTTGGCAGCTCATGATC
TTGGTCGGAAGGGAAAGGCGGTGGCGGTTGAGGCGGCCGGAACACCATCTTCAGGAGTGT
GCAAGGCCGAAACAGAGCCGGTTCAGATACAACCAGCAACGGAGTCGAAGCTAAAAGCG
AGAGAAGAAACCCATGTGAGTTAGTACTCCCATAGGTTAACCTAAGCACCCATGTCGGCGG
GTGTTACTCACTTTACCGGTGCATTTTATCAGTCAAGGCTTATTTTTTTTCTTTGTTTCGTGG
TGTACAGGGAACTGAAGAAGCTCGTGGTTCAACGTCTAGAAAGAGATCACGAACTGCAGA
AATGCATAACCTCGCCGAAAGGGTTTTATATTTCCATTGGAGTTTCAACAATGGACTCTCTG
TTTCATTTCCTTTAAAACGTACTTTTGTTTGTAAAACTTGTTTTTGTTTAACAGAGAAGGAGA
GAAAAGATCAACGAGAAGATGAAGACTCTGCAACAACTCATTCCTCGCTGCAACAAGGTC
TCCCAAAATTTCTTATATTCCTCGTTGTTTTATTGAACTGAAGGTTGAATCTGATTCTGTTTCT
ACTCTGATCAGTCTACTAAAGTTTCAACGCTGGATGATGCTATCGAGTACGTCAAATCGTTA
CAGAGCCAAATACAAGTATGCTCTTCAAAACAGAATGTGTTTAAACCAATGGTTCAACATG
GAAAGAGTTCATATGTATCTAGTTTTGTTGAGGTTACTAGAGTTATTTTTGGTCTCTATCAAC
CAGCGTCTCCGAAGAGTTTGAATGTTTGATGTCCGCAGATTCATCTTCTTGGAAGGGTTATA
AAGTTTCTTGGGAGATAAATGCAAATCTTATAAGTCTCTACTTCGAAACACTGGTACCTCCT
TTAGATGGGATGGCCTAATAGCTAATTCCTCTTATTATGCACTGTTCTTGAGGTCTTACTCTTC
TGTTTTGCTTCCCAGTTTACAGGAGGATTATGTATAATATATTAATATCTGAATCGATATTGTTA
TGTCAGATGATGTCGACGGGACAGGGTATGATGTCGCCAATGATGAATGCCGGGAATACGC
AACAGTTCATGCCCCATATGGCCATGGATATGAACCGACCTCCTCCATTCATACCTTTCCCCG
GCACATCTTTTCCTATGCCGGCTCAAATGGCAGGTGTAGGTCCATCATATCCAGCACCGCGC
TACCCTTTTCCCAACATTCAGACCTTTGACCCATCCAGAGTCCGTTTACCAAGCCCGCAGCC
TAACCCGGTGTCGAACCAGCCTCAGTTTCCGGCTTACATGAATCCCTATAGCCAGTTTGCTG
GTCCCCACCAGTTGCAACAACCTCCTCCTCCTCCATTTCAGGTAACTCTTTACCATGGTATCC
ATTGTGTTGTTGCAGGTAATCCTTATGTATGATGATGCTCACAAGATCCTTTTTTAAATATTCT
CTTAGGGTCAAACAACATCACAACTGAGTTCCGGGCAGGCAAGTAGTAGCAAGGAACCTG
AGGATCAGGAGAACCAACCAACAGCTTAGTAAGAATCAGGGAATGAAGGGTTTCCTTGGG
ATGTAAGTTA

>AtbHLH057
CCCTTGGTTTTTTATACATATATGCACCGAACCTCTTCTTCTTCTCTTCCTCGTCTTCTCTCC
TTTTATATATGTGGAAAACTGCATTTATTAAGAACAGTTTAGAAAGTGTCAACCCCTAAAGG
AATGTTTTTAGTTTAGAGGAAAGAGAGAGAAGAAGAAGCAGCAGCAGAAGTTGTTAATTT
GAAGACTATTTGAGGAAAGACACCTATATCTAAATACTCAAAGTTACAAAAATATTACTTCA
GAAAACAGTTCCATTAGAGAGACTCATAAAGCTTCTCATGTAAGCCTCTCTTGTAATTTATTC
TCATATACAGAATACTGTTTTGTGCATTTACTCTTCTTCTTCCTCCTCCTTTCATATGATTAACA
TTCAATGCAAAATTCTTGTAAATTCATTTTACAATAGCTTATCCAAATTCTGCTACTTTTATTAT
TGTTTCCTCTCGCTGCGACATTCTTTATTTCTCATCATTTAATGTTCTCTCCTCGTACATCCTC
TTTGTTCTGTCTTAAGAATTCACCTACCAAACCATTTATTTCTCATACCCACTTTAAGATTTAG
TAGATTTTTGTTTTCTTGTCAAAATGGTATCCTAAAACTAATCTCTCTTGTTTTTTTCTTTGTT
GAAGCTAATTATGAGTGGATTGATGAGTTTTGGTGAATTAGAAGACCAATTTGGTCAGATTT
CAGACACTACTATGGAAGAGAAGATACCATTTCTGCAAATGCTTCAATGCATAGAACACCCT
TTTACAACAACAGAACCAAATCAGTTTCTCCAATCACTTCTCCAGATCCAAACCCTAGAATC

AAAGAGCTGTCTCACCCTTGAAACAAACATCAAAAGAGATCCGGGTCAAACAGATGACCC
GGAAAAGGATCCAAGAACAGAAAACGGAGCAGTAACGGTCAAAGAAAAAAGAAAACGG
AAACGTACAAGAGCTCCAAAGAACAAAGACGAAGTTGAAAACCAAAGGATGACTCACATT
GCCGTCGAACGTAATCGAAGACGACAAATGAACGAACACTTAAACTCTCTCCGATCTCTCA
TGCCTCCTTCGTTTCTTCAACGGGTAACAAAATGTTTTAAGGGTTCATTAAACAAGAACGTA
ATTTTTTTTTCTTTTCAAGAACGTAATTTAGCTTTAACAAGAAAACGTTTTTGTTTCTCTAGG
GTGACCAAGCTTCGATTGTAGGAGGGGCAATAGATTTCATCAAGGAACTAGAGCAACTCTT
GCAATCTCTAGAAGCTGAGAAACGAAAGGATGGAACTGATGAAACTCCTAAAACGGCGTC
GTGTTCTTCATCTTCGTCTCTTGCATGCACTAACTCTTCTATTTCTAGCGTGTCTACGACGTC
GGAAAATGGATTTACGGCGAGATTCGGCGGTGGAGATACGACAGAAGTGGAGGCTACGGT
GATACAGAACCATGTGAGCTTAAAAGTTCGGTGTAAGAGAGGAAAACGACAGATCTTAAA
AGCTATTGTCTCGATTGAAGAACTAAAGCTTGCGATTCTACATCTCACTATCTCTTCTTCCTT
TGACTTTGTCATCTACTCTTTCAATCTCAAGGTAATTTTCTTAATCATCTCTTAAACTATCAGT
TTCAATTTCCACTAGTTTAAGCATTATTATAAATAGTCTTATGTAACTAGCAATTTTTGACTTT
TTGAGGAAAATTAAGGAATCTTCATTTTGAAATGTTTGGTACTTAAGAAAATTAAAATGCAT
GAGAAACCATAATAGAAAATATCTACAATTATGTGTGTGTGTGTGTATTTTTTTCCTTCACTCT
AAGCTTAGAAAATTTTAGTTTAATTTTTTAGTAAGTTGCTAATTTTTGTTTAGTTTTGGAGAG
ATGAGAAATGAATGGTGTTGTGCATGCAGATGGAAGATGGTTGTAAATTAGGATCAGCAGAT
GAGATAGCGACAGCCGTTCATCAGATCTTCGAGCAAATCAACGGTGAAGTCATGTGGTCAA
ATCTTAGTCGAACTTAGTTGACTTTTGACTCCTAGTAACGTGTGTAAACTTTAGGTTACAAA
GAAAAGGGACGTGATATAAATAAGAAAAACCAAAGAGGTGAAATTTTGGGAGTTTTAATTA
TTATCTTATACTTTTTGGATTTTAGATTAGTAGCAAACTCGCAGTGTTCTACGATGACATTATT
ATTGGTCACATGAAGGTTTAGGTTATTATTGCCTGTAATTTTTGAATATAAACTAACCTATGAG
TTCAGTTTGACCCCGAAAACGAAAAAAGTTAACCATGAGTAATATTTACTTCCATTTATAAAT
GTTTC

>AtbHLH058
AATGGATTACTTTTCTTCCACACATATATAACTTAACTCTCTCTTTTTCTCTTTTGCTTTAACC
CCCTCAAAGAAAAGATAAGAGCCTTTTAAAGTTTTTTTTTACCTTCTCTTCATGGACTTGTC
TGTACTTGATAGGCTTAAGTGGCTGCAACAGCAACAAATGGTTTCACCTGAGTTTCTTCAGA
TACTTGGCTCAGATGGGAGAGAAGAGCTCAAAAGAGTTGAGAGTTACTTGGGAAACAACA
ATGATGAGCTGCAGAGTTTCAGACATTTTCCCGAATTCGGACCGGATTATGATACTACTGAT
GGCTGCATTTCTAGGACAAGTAGCTTCCATATGGAGCCAGTGAAGAATAATGGACACAGCA
GAGCCATTACCTTGCAGAACAAGAGAAAACCAGAGGTTTGTAGCTCAAGAAAGTATTGAGT
ATTAGAGTTTAGATTTTGTTTGAAACATTGTTTCTGATCGTTTTAACTATTGGTTTTAGGGTAA
GACAGAAAAGAGAGAGAAGAAGAAGATCAAAGCAGAGGATGAAACAGAGCCAAGCATGA
AAGGGAAATCAAACATGAGTAACACAGAGACATCTTCAGAAATTCAGAAACCAGATTACAT
TCATGTTAGGGCTAGACGAGGTGAAGCCACCGACAGACATAGCTTAGCAGAGAGGGTAATT
AACTAAAGTGTTGTTGTTAAATAGGTTTCAGAGAAATTACTTTCATTAGAGATTTGAGTGGT
TTTATATTTGTGGGATTTTGGCAGGCAAGAAGAGAAAAGATAAGCAAGAAGATGAAATGTC
TACAAGATATTGTTCCTGGATGCAACAAAGTTACTGGAAAAGCTGGTATGCTTGATGAGATC
ATCAACTATGTCCAATCTCTGCAACAACAAGTCGAGTTCTTGTCGATGAAACTCTCTGTCAT
AAATCCAGAACTTGAGTGTCATATCGATGATTTATCCGCAAAACAGGTGAATAATAGTCAGT
TTTCATCCCAAGAACTAACCAAGAGTTTAATTACCGCATTTATTGACTGGTTAAATATTGGAA

TCTAGTTTCAGGCTTACTTCACAGGTCCTCCAGAAGGTGACTCGAAGCAGTCAATCATGGC
GGATTTTCGGTCTTTTCCATTACATCAGCAAGGATCTTTAGATTACTCAGTCATAAACTCAGA
CCACACCACATCTCTCGGCGCTGTAATAATCTTTATCTCAGACAAATCTTTCATTAGTCCCAA
TTTTTTGTTACTTGTCCGTGAAGTAACTTATTTCTTATTCTGTTTTGCAGAAAGATCATACATC
ATCAAGCTGGGAAACTCACTCACAGTGTCTTTACAACAGCTTGAGAACCGATTCTGTTTCC
AATTTCTTCAGCCTCAAGTAAAAAAATTAGGGATAGCCTCATTAAAAAAATCGCGGTTTTTT
GTTGTTGTCTTATCCATTTATCTATCTTATCTGAAATTTGAACCAAAAGAGACAGAGGAAACC
AATCCAAAGATCTTTCTCAATCTATTATCTTCATACAAATATAGTGATTTACATATATTCCAGG
GGATATGTATATGTGTAGAAGAAAGAGAAAAAACTCTTGTGGTCATAGCAATTCCTTTTTTT
GTACATTGTAGAATCAAACTCTTGTGGTCGTAACAATTATTTCCTTCACAAATTACAACTACA
CTTGTATTAATGGAGATGCCTTTTGGCCTGGTATCAAC

>AtbHLH059
AAAGGATATTTTTTTTATAAACAGAGCATAAAGTTTTCACTTTTCTTCTGCTCCTTTCTCGTCT
CTGTCTTCTTCGTCCTCATTCGTTTAAAGCATCAAAATTTCATCAACCCAAAATAGATTAAA
AAAATCTGTAGCTTTCGCATGTAAATCTCTCTTTGAAGGTTCCTAACTCGTTAATCGTAACTC
ACAGTGACTCGTTCGAGTCAAAGTCTCTGTCTTTAGCTCAAACCATGGCTAGTAACAACCC
TCACGACAACCTTTCTGACCAAACTCCTTCTGATGATTTCTTCGAGCAAATCCTCGGCCTTC
CTAACTTCTCAGCCTCTTCTGCCGCCGGTTTATCTGGAGTTGACGGAGGATTAGGTGGTGGA
GCACCGCCTATGATGCTGCAGTTGGGTTCCGGAGAAGAAGGAAGTCACATGGGTGGCTTAG
GAGGAAGTGGACCAACTGGGTTTCACAATCAGATGTTTCCTTTGGGGTTAAGTCTTGATCA
AGGGAAAGGACCTGGGTTTCTTAGACCTGAAGGAGGACATGGAAGTGGGAAAAGATTCTC
AGATGATGTTGTTGATAATCGATGTTCTTCTATGAAACCTGTAAGTTCTGTTCTTGCTTTGGT
GTTTGATTATTAGAAATGGGAAGAAATCGAATTTGAGGGTTTTGAAGAGAACATTTTTGGTA
ACTTTTGTTTTAGTTTTTGATTTGTGGTAAGTAACTTTAACAAAGTTGATGATTTGCGTTATG
TGCTTTATTTTGGCTCAGACTTGTTGAATCTAATCGATTTTGGTTTGTTTAGTAAACTAAGGTT
AGCTCTTGTTACTCTATGAGCAACTTCTCACTAGTTCATGTAGCTGCGTGGCTTATCTTGCCT
TCTAAGTTGGTCAAATGAGTAATCTTAAGGGTCTAACTTAAATTATTCTCTTACAACTAATGG
AGTCAAATCCTTGTGCAAATGGTTTATTTTCGTTTGGTCTTATTTCTTCTGTGTGTTGTCTGG
AAGGTTTTCCACGGGCAGCCTATGCAACAGCCACCTCCATCGGCCCCACATCAGCCTACTTC
AATCCGTCCCAGGGTTCGAGCTAGGCGTGGTCAGGCTACTGATCCACATAGCATCGCTGAG
CGGGTATATTTCAGATTCTCTTTTGTATTTCCCTGTGATATACTATGTTGGTGGCGAGTTGGTT
GTAGTTGAAAATATGTTTCGGATATAAAAAAATGTTAACATTGTGTAATGATCTCTTGATTTTA
TGGAGGGATTACCTAAAAGTTTGTTTTACTTTACCACAGCTACGTAGAGAAAGAATAGCAG
AACGGATCAGGGCGCTGCAGGAACTTGTACCTACTGTGAACAAGGTCTGTACCTTTCACCC
TAACCCTCACCTGTATATAATTTCTCTAGAAAGTTTGACACTTTCTGTTTCTCCCGTCTTCGT
GTGCAGACCGATAGAGCTGCTATGATCGATGAGATTGTCGATTATGTAAAGTTTCTCAGGCT
CCAAGTCAAGGTATATTCTTCTCTCTTCTGCAGTTTTAAACTCGAACTCTCTTTCTTGTGAAT
TATCTTATTACTCCCTATGCAATATGTTTAATCTGTTTGTTCATAAGATTAGAGATCTAAAGATC
ATGTCTTTCAAATTCTCTGGATGGATTTTTGGTGTTAATTCTGGTCCCACCATTACCTACCCTT
CAACCAAATTTCAATCTGATTAGATCAGTTATTTAATGTGGGAAAGAATAAACAAGTCCAAA
ATGCTTTTGTTTTCTTTGGCATTTAGTCTCTTTTCACTTTTTGTTTTGTCATAGGGAGCCTAAT
CACAGTTTATGATTATAATACCATATTCAACAATAATTACAATCATCATGTTCACATTATAATAT
AGTAATCTGGTAGATCATCAAATGCTTCTTATATCAAAATGCAGTCGGTTACTTAAAGCTCTC

GCAAGCGAAAAAAGGAAGTCTCTTTACTTTATAAGATAATGGAGACACTAAAGAAACAGAA
TGCAATGATGGATTTCTGATAAGATAATACATCTTTTGTTTTAAAAATAGAAAATGTGTTTCTT
GTGAAATCTTCCATGCTCATCTAAAAGTAAAAGCTTTTTTTATATATATAATTTTCATGGGGTG
AAGCTTATCACTTTCAATGATATCTGTTTCTCTTTGTCAACTTTGGTAGGTTTTGAGCATGAG
CCGACTTGGTGGAGCCGGTGCGGTTGCTCCACTTGTTACTGATATGCCTCTTTCATCATCAG
TTGAGGTAAAAACTTTTTAAGAACAAAAAGTAAAAACTTTAGTAATGGGGTTTTGATTCAG
ATTTTGCTTAAAATGTCAATAGGATGAAACGGGTGAGGGTGGAAGGACTCCGCAACCAGCG
TGGGAGAAATGGTCTAACGATGGGACTGAACGTCAAGTGGCTAAACTGATGGAAGAGAAC
GTTGGAGCCGCGATGCAGCTTCTTCAATCAAAGGCTCTTTGTATGATGCCAATCTCATTGGC
AATGGCAATTTACCATTCTCAACCTCCGGATACATCTTCAGTGGTCAAGCCTGAGAACAATC
CTCCACAGTAGGATTTCTGCAATAAAGAGTTTGTACAGCTAATCCAACTGTCCAACATGGGT
TTTTCTTCTGCTCTAATGACTCTGGTTTCTTCTCTCCTCTCTCACCCACTTGAAAGGTAAAAA
AGTGAAAAAGGCTTTGTAGATGGAATCAATGTAGGATTTGCAGTAGAGGGAAAAAAAATGT
CAAAAAGCTCAATTGATCAAGTATTATTGTAATCATTGTACCTTTATTTTAGGTGGACTTTGAT
GAAAGCAACTTTTTGTTTTCAAGACTTTAGTGGGAGGTTGAGGAAGGAGCTTGAAGGGTG
TTATTTATTAGTAGTAGTAGTAGTGGGAAGTTGTGGGACCTTGTTGAGTTGTGTTCAAATTGA
AGAAAAAACAAGTATTTGTAATTTGTCACCCCTTGTATTATTATTTATTTTGTATGACTTTGGA
GTAGTATAATTAATTATCAAATAATATTATTAGTTTGATCTAGTAACCATCCTTT

>AtbHLH060
AGAAGACGAAGAAGAAGAGAGAATAACTATTCCTTCTTCGCCAAGTTCTCTGAGCTATAGA
AGAAGGAGGGATTTCTTCAGACCGCCATTGTTGCCCACCTTCTCTCCTTTCTATTTTTCGCC
GCATTTTACTCGCTTACTGACAAAAGTCTTCTACTCAAGTCTCTCTCTCGCTCTCTTTGGAG
GCTCAATTAGGGTTTTGTTGTCGTGAGATTTGATTACACAAATTGCTGAATTTGGTTTCGATT
ATTGGTGTTATTGTTTTCGAAGATTTCCAGTGAGTTTCCGTTTATGGATCTGACTGGAGGATT
TGGAGCTAGATCCGGCGGTGTTGGACCGTGCCGGGAACCAATAGGCCTTGAATCGCTACAT
CTCGGTGACGAATTTCGGCAACTAGTGACGACTTTACCTCCCGAGAACCCCGGCGGTTCGT
TCACGGCTTTGCTTGAGCTTCCACCTACACAAGCAGTGGAGCTTCTCCATTTCACTGATTCT
TCGTCTTCTCAACAAGCGGCAGTGACAGGGATCGGTGGAGAGATTCCTCCGCCGCTTCACT
CTTTCGGTGGGACATTGGCTTTTCCTTCTAACTCAGTTCTCATGGAGCGAGCAGCTCGTTTC
TCGGTGATTGCCACTGAGCAACAAAACGGAAATATCTCCGGGGAGACTCCGACGAGCTCTG
TACCTTCCAATTCAAGTGCTAATCTCGACAGAGTCAAGACGGAGCCTGCTGAGACCGATTC
ATCTCAGCGGTTGATTTCTGATTCAGCGATTGAGAATCAAATCCCTTGCCCTAACCAGAACA
ATCGAAATGGGAAGAGGAAAGATTTCGAAAAGAAGGTTAGAGTGAGAGACAAATGTTTTT
ACTTTTTTACATTTGATCCTTCGTTTCATCAGATTGATTTTTTATAGGGTAAAAGCTCGACGA
AGAAGAACAAAAGCTCTGAAGAGAACGAGAAGCTGCCATATGTTCACGTTAGAGCTCGTC
GTGGTCAAGCAACCGATAGCCATAGCTTAGCAGAACGAGTAACTTGCATCTGCCTTCACTAA
TTTTTCTTTTTTTTGGTATTAATCAGTTGATTAATTCGCTAATTGATCACTAATTTTTGGATTAT
AGGCAAGAAGAGAGAAGATAAATGCACGAATGAAGCTGTTACAGGAACTGGTCCCAGGCT
GTGATAAGGTTTGTCTCTTAGATATAGACGTGTAATCAATTTGTTAATCAATCATTAGGTCTTC
ATTTGTGATTAACGACATTATATTTCTCTAATTGATTCACTCCAAAAGTAAGAATTTATTATTA
GATGATCTAGTCTTTTTGTTAAGGGTCTTAATCCTTCACACCTACTTTTTTTTTTCTTCCTAAA
AAGTGGATTGCTTAATTGCTTTTTTATTAAGAAAAGTGGTTCCTGGTGTGCCAAAGTAGCCA
GTTGGGTAATTGTGTAGTAATGCCTTTAGAGATCTCAACTTTATTTTTTTATTTCGTGTGGGG

AGAAATATCTCACGGGTACACACTTAGCTTTAAGGTTAACTCAGTCACACGGAAGTGATAG
GACCTTGCTTTATCGAATTCTTTTTCCATAAGTGTGTTCGTTGAGCTTGAAGATTAGTCTATA
AATGACGAGAATGACCTTAGGAAGTGTGCTAGCTGTAATATGCCATTAAGGTTTTGGGTTTA
GGGGACAGATTTTGGTGGAAAGATCAAGATCAAAGTGTGTTTTGGGGTCCATCTTTTGATGA
TAAGTGGGAAAAAAGTTGCAATCTTTCTATGGAAGGTGAGGAAGGGGTAGTATCGTCTCTA
ACAAGTTCGGTATCTATCAAGGAAAAATAGAAAATGACTCTTAGAAGGACGATTAAAGCAA
ATGATCCTTTACAAAAATTGATATGCTTTGAGTCCCACATACCTAATTAGTTGGAAACATGAT
GTGACACTCCCCCAGACAGCTAATTAATCATCCCTGATGTTAGAAGTTAGCAGCAATTATTG
GTGTGATAGAGATGATGATATGGAACATCAGTGTTTGTTTAAAATTTGGATTCTATAGGTGAG
CTGTGAGGACTTGATTGATTGTTCCTTTAGCCCCCCTAGGGTATACTGATTACTTGTGATTGT
AGTAGATACCATTTATTATAATAGCTTTAGATTAGCACGTCATTCTCTTGCTGCTTTTTTCACT
GTGTATTTTGGAATCTTGGTATTTTCTCGTTGAAATATTGGATCATCTTAACAATAGTTAGCTG
TCTAGAAAGAATTTTTGGTATGTGAAAGCAGATCTACCTAATATGAATCCGAGCTTATCAGA
AAGTCTTTGACCTGCAGATTCAAGGTACCGCGCTGGTGCTGGATGAAATCATTAACCATGTC
CAGTCATTACAACGTCAAGTGGAGGTGAGACTTTCCGTGGTCCAATTTTTGTTCTTTATACCT
ATATTTTACAATAGCCCTGGGCACAATAAAAGGGTCAACTCAAAACAATTTGAAAATGAATA
GAATTATGTGCCGCAATCTCATGAAACTGTGACTTGTGTTTCCACAGATGCTATCAATGAGA
CTTGCTGCGGTAAACCCCAGAATCGACTTCAATCTCGACACCATATTGGCTTCAGAAGTAAG
TATCAAAATCTCAAACAATAATTTGGTCATTCTGATTAGTCTTCTCTAGGAAAAATTTAACCA
TTTCATGTGTTTCTCATTTAAGTTAACCATACCTCTTTGAGTAGCTTCTAGTTTCTATCACAAA
AGGAGAAACTTTTGGGGTATTTATTGATACAAACATTAATTTAGACCATTTTAGTGTAAGGCA
TAGGAAGATCACAACGTCTACTCTCATGAAGGAAACTCAGGGAAAAATAAATACACAACAC
ACACACACACCCAATGCAAATAACAGTAGTGGGTCAAAACTCGGACCACCTTTGTTCTGTA
AAACCTTTCTGTGTCTTCTGTGACTTATGTCGGATGTGTTACTTGATGAACCTTTTAAAAAA
AAAGTTAATTCATCCCTCAATAATTTCTGTGGGTCTCTTCCAAAATCTTGTTATTTCAAAGTG
ACCTATCTAATTTCATGATCACCTGTGGATTCTCATCAAAAGTTTTGGACCAAACTAAATAAA
TGCTCCCTCTCTCTTTCTTCTTACAGCTTGTACCACCATTAAATAGCCAGGGACCCTCTCATC
AAACCTTGTTCTTTTAGGATAATAATAACACAGCAACAACAGTTGACTTCTTTGACTAGATC
AAGATCTCTATGAATGATCCCTTTGCTGATATCTTTTCCGGTTTTGGCTCTGTGTTTTTTTCTT
TGACAGAACGGTTCTTTAATGGATGGGAGCTTCAATGCCGCACCAATGCAGCTTGCTTGGC
CTCAGCAAGCCATTGAGACCGAACAGTCCTTTCATCACCGGCAACTGCAACAACCACCAAC
ACAACAATGGCCTTTTGACGGCTTGAACCAGCCGGTATGGGGAAGAGAAGAGGATCAAGC
TCATGGCAATGATAACAGCAATTTGATGGCAGTTTCTGAAAATGTAATGGTGGCTTCTGCTA
ATTTGCACCCAAATCAGGTCAAAATGGAGCTGTAAGTTGGGAAAACGGTAGAGATCATGAA
TGTGTATATACATCGTATAAGCTCGTTTCTCTCTATATAAATATAATCATAAATATAGATATCTGT
TAAGAAGGTATCAGTCATTTGATTCAGAGAGACAACACTGGTATGATTGTTTCTTATTCTTGT
ACCAGATTTCGACAATGTAGAATTTAGTAGGATATGATCATTTTGATCTCGTTATATATATCTTA
CTAATAATAAATAGAGATAAAAAAA

>AtbHLH061
GTAATTCTAATCAGTTAAAAAGTGAATTCTTCATTACAATTCACACTTATGTAATGCATTTACA
TGCATTTGTATCTTCTTTGACCCCAAGCTCACAAACTCTGTTCACTTCCACTTTCACAAATAA
CAGAGTTTCAGAAAATAGAGAGAAAGAGAGATGGAAACGGAATTGACGCAATTGAGAAAA
CAAGAAAGCAACAATCTCAACGGCGTTAATGGAGGATTCATGGCGATTGACCAATTCGTCC

CAAATGATTGGAACTTCGATTACCTCTGTTTCAACAATCTTCTCCAAGAAGACGACAACATT
GATCATCCTTCTTCTTCTTCTTTGATGAATCTAATTTCTCAGCCACCTCCATTGCTTCATCAAC
CACCACAACCGTCGTCACCTCTTTATGATTCTCCGCCGCTCTCCTCCGCTTTCGACTATCCTT
TTCTCGAGGATATCATACACTCCTCTTATTCTCCTCCTCCATTGATCCTTCCGGCTTCGCAAG
AGAACACCAACAATTACTCACCATTGATGGAGGAAAGCAAGAGCTTCATAAGCATCGGAGA
GACTAACAAGAAAAGGAGTAACAAGAAGCTTGAAGGTCAGCCTTCGAAGAATCTTATGGC
GGAGAGACGGCGGAGGAAACGGTTAAATGACCGACTCTCCCTGCTCCGATCCATTGTCCCT
AAAATCACCAAGGTCAAATTCCATAAACCTCATCCTTGTCACATGTTATATGGTTTTTATTTTT
AAGTCAAGAAGAAATATTCTTCCTTTTTTGTTAAGTTCTTAACGTTACATGTATGTTTTCTTCT
TGTTGAAATCTTTTTTTTGGTACACTGTTCACTGATAAAAGGTTATTGCTTTTTGTGAATCTG
CATAGAGTTTGTTGTATGAGAAAATGTATATAATTTTTGATGATTATTATATAATAAAATCAGAT
GGATAGGACATCGATACTAGGAGATGCAATAGATTACATGAAGGAGCTTTTGGACAAGATTA
ACAAGTTGCAAGAAGACGAACAAGAACTTGGAAGCAACTCTCATCTAAGCACCCTCATTAC
AAACGAATCTATGGTCAGAAACTCCCTAAAGGTAAAAGCTAAAAACAAAACATAATGTTATT
ATGGTATTTGTGCTTCAGTACTCATAAGTCTTGTGTTTTTACAAATTCATCCCATTACAAATCA
TTTGTTACAAACACCCTCATCATATACTTGAATCAATTTGATTCCCATTAGATATCACTCAAAA
AATTATAATCACTTGAAGGTGCAGACAATTTTTCTGGCTATAACGAAAACGAATCTTTTGCA
GTTTGAAGTAGATCAAAGGGAAGTTAATACGCATATTGATATATGTTGTCCTACGAAACCGG
GTTTGGTTGTATCAACGGTTAGTACATTGGAGACTCTAGGATTAGAGATTGAACAATGTGTC
ATTAGCTGCTTCAGTGACTTCTCATTGCAGGCTTCTTGTTTTGAGGTACAAATATTATTACAC
TTTGATTTAGCCTTACTTGGTGCATCGACAAATTAAATGTATATTCTATAATTATTTTCTGTTAT
ATTCTGTTTTTTTCGTATATATGTAGGTTGGTGAGCAGAGATACATGGTTACTTCGGAAGCTA
CTAAACAAGCACTAATCAGAAACGCAGGTTATGGAGGAAGATGTCTGTAGGGGAGCTTCAA
AATTGCAATGTTTTTTAATGATGATAATATTTTCCGGTTCTTCTTATTCTCTTGTGATGAGTATG
ATATTTCATAAGGAGATAGATAAATGGGAAAATCAAAGGTGTAAAAAGAACTCATTGATGTT
TACCAATTAGTCTCCTCGACTGACCCCTCGGTTTCTAATCCGACTCATTAATAAAACATGCAA
A

>AtbHLH062
GAAGCTTGTTTCTCCAATTATCTCATTTGGTAAAGACGTTTCATTAGCGATATCGTGTTAACG
TCGAAAGTCCTCCTCTCGTTACGGTCCGGTCCGACACAGAAATTTTTATTTAATATTTACTTT
ATTTTTTGTCCATTTATTAGAAAGAAAGTCATTGATTGGTGCAATTTATTAATATAAATTTATTA
AACAACTATATAAACTCCTCATTACTGCCCATTAACGAGCTTCCCTTTTCCTCTGTTCTTCAC
TTTAAGAAAATTACAAACTTTTATCTTCTATTGGACGAATCTTTGATCTGGTTTTAATTAATCA
CACGCTTACTTTAGGATTTTTTTACCTGAATTCGAATATATACTTTTGATTTTCATCTTCCGTA
GTCCGTACCGTAGAATTGAGGAGCTTCATGGAGAACGAGCTGTTTATGAATGCAGGAGTTT
CGCATCCGCCGGTGATGACGTCACCGTCTTCTTCGTCGGCGATGCTTAAGTGGGTTTCAATG
GAGACTCAGCCAGTGGATCCAAGTCTCAGTCGCAATCTGTTTTGGGAAAAGTCAACGGAA
CAGAGCATTTTTGATTCGGCTCTGAGCTCACTCGTCTCTTCTCCTACGCCGTCAAATTCAAA
CTTTTCGGTTGGCGGAGTAGGTGGAGAAAATGTCATTATGAGAGAGCTCATCGGGAAACTG
GGTAATATCGGCGATATCTATGGAATTACGGCGAGCAATGGAAACTCGTGTTACGCAACTCC
GATGAGCTCTCCACCGCCTGGAAGTATGATGGAGACGAAAACGACGACGCCGATGGCAGA
ACTGTCCGGCGACCCGGGATTCGCTGAGAGAGCGGCGAGGTTCTCGTGTTTTGGTAGCCGG
AGTTTTAACAGCAGAACAAACTCGCCGTTTCCGATTAATAACGAACCACCCATCACAACCA

ATGAGAAAATGCCACGTGTATCAAGCAGCCCAGTTTTTAAGCCTCTTGCGTCTCATGTTCCC
GCCGGCGAATCGTCCGGTGAACTTTCCCGGAAAAGAAAAACTAAATCCAAGCAGAATTCTC
CTTCTGCAGTTTCATCATCAAAGGTTCATAATGAAGCTTCACATTCGAATAAACCCATTTTTT
GTTTGGTTTAAACAAATACTAATTTTAGTTTGATTATCAGGAGATTGAAGAGAAGGAAGATT
CTGATCCAAAGAGATGCAAAAAATCAGAAGAAAATGGAGACAAAACTAAATCAATTGATCC
TTACAAAGATTACATTCATGTTAGAGCTCGACGAGGCCAAGCCACCGATAGTCACAGTCTCG
CCGAACGAGTCAGTCAATTTCCTATTTTCTTACCAAATTTATTGATTTTAATCTTAATGTTTAA
TTACGTCCTGATTAGTTGAATTAATCATGCTTTGTATTCAATGTGTTGTTGTGATTTCGATATT
AAAAAGGTTCGAAGAGAGAAAATAAGCGAAAGAATGAAGCTGCTTCAAGATCTTGTCCCT
GGATGCAACAAGGTGAGAGTTTGATTAAACAACTAATTAACTCGTTTTTAGTTGTACTAATAT
AGTTGTGATTAGAGCAATAACAAATTTACTTTTTAAAGGTTACTGGAAAAGCACTGATGTTG
GATGAAATTATAAACTATGTCCAATCATTGCAACGACAAGTTGAGGTAATTACTCATTAGATT
ATTGGTCTACTTTAAACTCTTTCCATTATTTCATAATCACTTTTTAAAACAACTACACGACTTC
GAATAATTTTTGTTTCTTAATTTTAATTTCAAATATGGTGCAGTTCTTGTCGATGAAGTTATCG
TCAGTGAACACCAGGCTGGACTTTAACATGGACGCTCTCTTGTCTAAGGATGTAAGTCACC
GTCACAACATTTATAGTAATTTTAATTGACAATTATTGTAATAATAATTACTTTTTTGTCTTTGT
GAACCAATCGTGTGTTTGTTTAAACTAATTTCTTTTGTTTTCTGACAGATATTTCCTTCAAGC
AACAATCTAATGCATCATCAACAAGTACTTCAATTAGATTCTTCAGCAGAAACGTTATTGGG
TGATCATCACAACAAAAATTTGCAATTGAACCCTGATATTTCTTCTAATAACGTAATAAACCC
TTTGGAAACTTCTGAAACTAGAAGCTTCATTTCTCACTTACCAACACTTGCTCATTTCACTG
ACTCTATTTCTCAGGTTTCCTTTTCTAAAACATTCTTCTATCACTTTTCTTTTCTTTTTCTCTTC
AATTTGAAGCGGTCTTAACCCTTTTTGGTTTTGTTTTTAGTATTCAACATTTTCCGAAGATGA
TTTACATAGCATTATTCATATGGGTTTTGCGCAAAACCGTCTCCAAGAATTGAACCAAGGTA
AGAATATTTAAATCATATTTTTTCTTTCCATTTCATTATATAGGCAGGACCGTTTAGATATGATA
TGTATAAATTGTACTATTATATATGCAGGTTCATCAAACCAAGTACCATCGCACATGAAAGCT
GAACTTTGATCAACCACTATATGCATTTTTACAAAAATATTGGCGTTTTAAAGGTCTCGAGGA
TAGAAAGTAAGAAAAGGATTGATGGGTGTACATAACAAGAAATTTGAATTGGACCACACCA
TATGCGAATTCATTTCTATAATCATCTTCATATACATAACTTCTTCTTTTTATCCTTCCGATTCAT
TTGTGGTGCTCAATTGTATTGTCATGTTGTTATCCGCTATTCTTCTTCTTATCAGATTCTTTAA
ACTTTTCTTGTTTGGATTTAATATCATTGTATGTTTTAACCCCACTATAAGTACTATAGCTATAA
TTCTTCCGAACAACAAGACTGTTTTTATCAAAC

>AtbHLH063
CTCTGTTTCTTTCTCCACTCTCTCTCTCTCTCGTTGCAAGCAACTTGTTTTCTGAATCCAAAG
AAGACGATAACGATGAATGGAGCTATAGGAGGTGACCTTTTGCTCAATTTTCCTGACATGTC
GGTCCTAGAGCGCCAAAGGGCTCACCTCAAGTACCTCAATCCCACCTTTGATTCTCCTCTCG
CCGGCTTCTTTGCCGATTCTTCAATGATTACCGGCGGCGAGATGGACAGCTATCTTTCGACT
GCCGGTTTGAATCTTCCGATGATGTACGGTGAGACGACGGTGGAAGGTGATTCAAGACTCT
CAATTTCGCCGGAAACGACGCTTGGGACTGGAAATTTCAAGAAACGGAAGTTTGATACAG
AGACTAAGGTTCGTGTGTAAAGAAAACTTAAGTTTAAGTTCTATAAATGAAAAAGAAAACA
GATCTTACTTTGAGATTTGGCTTATCTGTTTCTTCTGTGTGTGCTTTTAGGATTGTAATGAGA
AGAAGAAGAAGATGACGATGAACAGAGATGACCTAGTAGAAGAAGGAGAAGAAGAGAAG
TCGAAAATAACAGAGCAAAACAATGGGAGCACAAAAAGCATCAAGAAGATGAAACACAA
AGCCAAGAAAGAAGAGAACAATTTCTCTAATGATTCATCTAAAGTGACGAAGGAATTGGAG

AAAACGGATTATATTCATGTTCGTGCACGACGAGGCCAAGCCACTGATAGTCACAGCATAGC
AGAACGAGTAAGAGAGCCTCATTAACTAAAATTCATTAACTTTGTTTTTTGTGTAATCATTGA
GACTAATTATTAAAGTATTAAATTTGTGAAGGTTAGAAGAGAAAAGATCAGTGAGAGAATG
AAGTTTCTACAAGATTTGGTTCCTGGATGCGACAAGATCACAGGCAAAGCAGGGATGCTTG
ATGAAATCATTAACTATGTTCAGTCTCTTCAGAGACAAATCGAGGTCTGAAAATTATTTCCAT
TTATAGAAAATATGTTTTAAAGACTAATTTTGCATTACCTTCCTAATTTGTTATCAAAATATTG
TTTAAAACAGTTCTTATCGATGAAACTAGCAATTGTGAATCCAAGGCCGGATTTTGATATGGA
TGACATTTTTGCCAAAGAGGTTAGTCATTGATTTGTTTATGCTTATATGAAAAACAAAACTTT
AAAGGCTATTTATAGATGAGCTAAAATACGCCATGTGGATTCAAAACCAGGTTGCCTCAACT
CCAATGACTGTGGTGCCATCTCCTGAAATGGTTCTTTCCGGTTATTCTCATGAGATGGTTCAC
TCTGGTTATTCTAGTGAGATGGTTAACTCCGGTTACCTTCATGTCAATCCAATGCAGCAAGTG
AATACCAGTTCTGATCCATTGTCATGCTTCAACGTAAGTCATATTTTTAATACTCTTTCATTAT
GATCTATCCTTTAGTTTAACTTTGGATATGATAATTTTTTACTAAATATGTTTTGATTGTTACAG
AATGGCGAAGCTCCTTCGATGTGGGACTCTCATGTGCAGAATCTCTATGGCAATTTAGGAGT
TTGATTAAGAAACCAATCCACAACATAACAATGTTCCCTAATTCAAATTATTCATCGGAAGA
GAGAGCAAACCAGTAGCTGTTATTTACTAATTCCACTAGATTATTGACTTGAGATCCCCCGC
CCATTAGTTGATGTATCAAAGAGTGTAGACATTATTGTGTATAATTTGCAAACCAGTCCAGTA
ATATATTTCTTATCGGTGGACTTGTGTCTTTAATTCAAGGTGTGT

>AtbHLH064
GTAGGGGACCTTATGTGTGTGGGTCAATGTTGTTTTCTATTTAAAGCTAAGAAGAATGATTTC
AATGGATTAGTTTCTCCCAACTCTTTCATCTTTTTTAAGCAACCACCCAAAAACAATATTACA
TAACCTAAATCTCTACTTCTTCATACATTTATAGCAAATCCTCTTTTGCTTTAAACCCACCAAA
TAACTCAGAGCTTTTTTGCATTTTTTCCCATTCTCTATTTTGTTTTGTACTTTTGGTCTCACTTT
AAAAGATCATAAGTTGAAAGATTTCTGCAGAGAACAATATGTTGGAAGGTCTTGTCTCTCA
AGAAAGCTTGTCCTTAAACTCTATGGACATGTCTGTACTTGAAAGGCTTAAATGGGTACAAC
AGCAACAACAGCAACTGCAACAAGTTGTGTCCCATAGCAGTAATAATTCACCTGAACTTCT
TCAGATACTTCAGTTCCATGGAAGCAACAATGATGAGTTGTTGGAGAGTAGTTTCAGCCAAT
TTCAAATGCTTGGATCTGGTTTTGGACCAAACTATAACATGGGTTTTGGTCCTCCACATGAAT
CCATTTCAAGAACAAGTAGCTGCCATATGGAACCTGTGGATACAATGGAGGTTTTGTTGAAG
ACCGGTGAAGAAACCAGAGCCGTTGCCTTGAAGAACAAGAGAAAACCAGAGGTTTGTAA
GATCAAAATTTTGGATAAAAACTTTGTTTTGAGACATGGTTTTTGATATTTTTTATTAATAATG
TCTTAGGTTAAGACAAGGGAAGAGCAAAAGACAGAGAAGAAGATCAAAGTAGAGGCTGA
GACAGAGTCAAGCATGAAAGGAAAATCAAACATGGGAAACACTGAAGCATCTTCAGACAC
TTCAAAGGAGACATCGAAAGGAGCTTCAGAGAATCAGAAATTAGATTATATCCACGTGAGA
GCTCGTCGAGGCCAAGCCACTGACAGACACAGCTTAGCAGAAAGGGTAATAAAAATAAAT
AAGTTTCTTGTGATACAAGAATCATGATATATCTAGGGTTTCTGAATGGATTTGGATTTGCAG
GCGAGAAGAGAAAAGATCAGCAAGAAAATGAAATATCTGCAAGATATTGTGCCTGGATGCA
ATAAGGTCACAGGAAAAGCTGGTATGCTTGATGAGATCATCAATTATGTTCAATGTCTCCAA
AGACAAGTCGAGGTAAGCAATGAAACCATGATTCTCTGTAACTTAACTTCACTATTAACCAA
AGAGAATAAGTCTAATTCTTAAAGAGCCATACTTTGCAGTTCCTGTCGATGAAACTTGCTGT
CTTGAACCCGGAACTAGAGCTTGCCGTGGAAGATGTATCCGTAAAACAGGTGAAAACACAT
TGTCTAGACCCTCAAAGTCATATTGATATTGCTCAACAAACTTAAGGGTTATGAAATTTTCAG
TTTCAGGCTTACTTTACAAATGTAGTTGCTTCAAAGCAATCAATAATGGTTGATGTGCCATTG

TTTCCGTTAGACCAGCAAGGATCTCTAGATTTGTCTGCGATAAACCCGAACCAAACGACATC
TATCGAAGCTGTAAGTACAGCTTTATATGAAACTGAAAAACTTGTCAGAGTTTTTGTTCCTT
GTCAGTGAAGTAACCTGTCTCTGTATCTCTCTATTTCAGCCATCTGGAAGCTGGGAAACTCA
ATCACAGAGTCTCTACAACACATCTAGCCTCGGTTTTCATTACTAAGCAAGATTCATTGAAA
CAACATGGTTGACATCAATCAATCATCAAAATCAGAAGCAAATTCTATTACATTTGCTCATCA
AAGTAGTAATTTCGAAATTTGGTTAATGCATTATCCTTTGATCCTTGTTTTCTGATATTTAAAC
CAGAAGAACTGGAGATAGCAATCCAATGATCTTGTCACCATCAATCTTTGTGTATAGATTATA
TAGAGAGATAGATAAATATGTTTTAGAGGAGTTATAGATATATATCATATTTGTAGAAGAAAG
AGAGAAAACTCTTGTGGTAATTACAATTCTTGTTTGTTTTTTTGGATATATAAATATCTAAGA
AAATTATAAATCGTTTTCCTGTTTTTTTTTTCTGCAACGAATATCTAAGAAAACTCTTGTGGT
AATTACAATAAAATATCTAAGATTCTATTGTCAACGAAATGTACCCATCAATATGTTCCCATTC
GAAGGGTATGGGTATAGAGTTATTAGGTCGCCATGATTTTTATAAATCGGTGGAAGAATACT
TTTGATGATCAAAGTAAACGGTATCTAGGCAGCCAATTAATCAATGTCGATTTTATCTTCACA
TAAAATTTTATTCATTGAATACAAATTATTTTC

>AtbHLH065
GCATCGTAGAAAATAGAGAACTTCAATTTGTTTTTTGTTTACCATTATTAGAGATACTATTCG
CTGAAAGAGAAGCATAAGAGGGGTGTTATGATTTGACAAAAACACCCTTAATCATTGGTCA
AGACTCAAAGAGTGTTGCAGATAAGGTACAGATCAGAAACCCACCTCTAGAAATCGAGCAA
TAGATCTCTCTCAACTTCACAGACAAAATCCAACCGAGTTGGTGGGTCTCACTAACTCTTTA
ACTAGTTGTCTGACAATTCGCCACGTGTCGGTCATCCTGATCAGTTTCAGTACTATTGCCCCC
ACCGAATCATTATCTCCCCCTCGCTCACTCGCTTACTTACCGTAATAATCTCTCTGCTTCCCTC
CTCTAGCTCTTGTCCCTTTCTGTCTGAACCTGGCAAGAAGAACCTTAGCCTCTCTTTCTTCTT
TCTCTCTCTCTCTCTCTGTGTTACTGTTCTGTTTCAACTTTACTCCCTCAGTTTCAGAACAAT
TCCCTATCTAGAAGAGAGATAAAACCGAGAAGGTTTTGGAGATAGAATCTTTTGTTCTTCTT
TTGTCCCTCCTTGCTCGATTTTTGTTACGTGTGAAGCAATAAAAAAAAACTGATATAGCTAA
ATCTTCCATCCATTCAGAGGCTTCTAAATCTGGTAATTACTTTTATAGCTTCCCCTTAGTAAGA
GTTTACTATATATAGAATTAGTTGCTTTATTAAATCATTTCCTCCTAGATTGTTGGAGAGGGTT
GTTTGGTTTAATTATTTTCTAATCATTAAACTGTTGGGATTTCGAATAATCTTTTCTAAGTTGA
TTGCTTTCATCATCCTTTTTCCTCAAATCTCCTGTTAATCATTATACTAATTATGAATCAAATCA
TTTCTTATTCACTTACAAAAAAGTAATATATTCTTTATTTAAGTGTCTTTACAGATCTGACATG
GAACAAGTGTTTGCTGATTGGAATTTTGAAGATAATTTTCACATGTCCACTAATAAAAGATC
AATCAGGTATATATCAATTTTCCAATTCAAGTATTTCATAATTATATACCAATTTCACATCTTAA
TCATAGCTATTTAAGTATTGTTTGTTTAAAATATGTTTGTAAACATTCATATGTTGGGACAGAC
CAGAAGATGAATTAGTGGAGCTATTGTGGAGAGATGGTCAAGTGGTTTTACAAAGCCAAGC
TCGTAGAGAACCGTCAGTCCAAGTCCAAACCCACAAACAAGAAACCCTAAGAAAACCCAA
CAATATTTTTCTTGACAACCAAGAAACAGTACAAAAGCCTAACTACGCTGCTCTAGATGATC
AAGAAACCGTCTCCTGGATACAATACCCTCCGGATGACGTCATCGACCCTTTCGAATCCGAG
TTCTCCTCTCATTTCTTCTCTTCGATCGATCACCTCGGAGGTCCTGAGAAGCCACGAACGAT
CGAAGAGACAGTTAAGCATGAGGCTCAAGCCATGGCTCCTCCTAAGTTTAGATCCTCGGTTA
TAACAGTCGGACCGAGTCATTGCGGCAGCAACCAGTCAACAAATATTCATCAGGCCACTAC
ACTTCCGGTTTCTATGAGTGATAGAAGCAAGAACGTCGAAGAAAGACTTGACACTTCGTCA
GGTGGCTCCTCCGGTTGCAGCTATGGAAGGAACAACAAAGAAACCGTTAGTGGAACAAGT
GTAACCATTGACCGTAAAAGAAAACATGTTATGGATGCTGATCAAGAATCTGTGTCTCAATC

AGATATAGGTTTGACCTCAACCGATGATCAAACCATGGGTAACAAATCGAGCCAACGGTCA
GGATCTACTCGAAGAAGCCGTGCAGCTGAAGTTCATAATCTCTCAGAAAGGGTATACATATA
CTATTTCTGACTAGTTGATCAACCATTCAGTAATAATAATTCTGATGTGATCTTTGTCTATTAA
TTACAGAGGAGGAGAGATCGGATCAATGAAAGAATGAAAGCTCTTCAAGAACTCATACCTC
ACTGCAGCAGAGTAAGTTTTTCATCACAAAAAAACTGAGTTAATTAAGCTGTAATTGATTG
GTGTGTTTAAATATGGTTTTGTCTTCTTCTTTTTTTGTGTAGACAGATAAAGCTTCGATATTGG
ATGAAGCAATTGATTACTTAAAATCACTTCAAATGCAACTCCAAGTGATGTGGATGGGAAGT
GGAATGGCGGCGGCGGCAGCAGCAGCAGCAAGTCCGATGATGTTTCCCGGGGTACAATCAT
CTCCATACATTAATCAGATGGCTATGCAAAGTCAGATGCAATTGTCTCAATTCCCGGTTATGA
ACCGGTCCGCTCCGCAGAACCATCCCGGTTTAGTATGTCAAAACCCGGTACAGTTGCAGCT
CCAAGCACAGAACCAAATCTTATCGGAGCAGCTCGCTAGGTACATGGGCGGGATTCCCCAG
ATGCCGCCGGCGGGAAATCAGGTCAGTTGCAGACGGCATCGTTTTGAAGAAAGACAAATAT
ATTTATTTAATATCTGACGTGTCATGTTTTAAATTAGATGCAGACCGTGCAACAACAACCAGC
GGACATGTTGGGATTTGGATCTCCGGCGGGACCGCAAAGTCAACTGTCGGCACCGGCGACC
ACCGACAGTCTTCATATGGGTAAAATAGGCTGACTTGGCATATAGTTTTCCTCCGAAATTATT
CTTCTTACAGTTGGTGATTGTTATTTATTTTTGGTCGCCTAAGCAAGCATAAAAGCTAAGTCA
AATGTATTATAGAGATCTAATAAGTTAGTCTCATACTTATAACTTATTTTTAAACAGTTGAATTA
TAGTATCAATCAAGTGTTGGGAACCTAAAGATCATACATGTGTCAATACTTTTATATTTGTTCT
CAAGGTTCATCAGAAAAACAAAATAAAAAGGATAGACTAGGCCTGCATTTGACATTATCATG

>AtbHLH066
AAACAAAAGATGATAATCATCGATTCATCATCACGATATAATATGGGTTGCTAACCACAATAG
AAAAACAAAAGTTCTTTTAAGGTTTTAATAATATATCACCAAAAATGATAATTAAAACCTAA
AATTAAAATAATATATCTACAAGACTACAACAATTCTGCTTTATAATAAAGCTATAAACCTCA
ACATTTTTCAACCTCTTCCCAAAAAAAAAGTGTAGAAGCAGAAGAAACCCATCATCATCATG
ATGAACTCTTCTCTTCTAACTCCTTCTTCTTCATCTTCTTCCCATATCCAAACTCCATCAACAA
CTTTCGACCACGAAGACTTCCTCGATCAAATCTTTTCCTCGGCCCCGTGGCCCTCCGTCGTC
GATGATGCTCATCCTCTTCCCTCCGATGGCTTCCACGGCCACGATGTCGACTCAAGGAATCA
GCCGATCATGATGATGCCTTTGAATGATGGCTCCTCCGTCCACGCTCTTTATAATGGCTTCTC
CGTCGCCGGATCTCTTCCTAACTTTCAAATCCCTCAGGTATAATCTCTTTCTCTTTTCTAAGTT
TTTCTCTTCTTGAAAACGAGACTTATGGTGAAATATAGGGATCGGGAGGAGGATTGATGAAC
CAACAAGGACAAACGCAAACGCAAACGCAACCTCAGGCGAGCGCGTCTACAGCTACTGGT
GGTACGGTGGCGGCTCCGCCGCAGAGTAGGACTAAAATCCGAGCTAGGAGAGGTCAAGCA
ACTGATCCTCATAGTATCGCCGAAAGGGTTAGTTGTTTTTGTTTTTTATTTTACTTTGTTCGAT
GCATTAATTTGTATCAGTTACCGAGAAAGGTGTCGAAATTGAATTTTTTTTAGCTTTTTGAAG
AAAAGATTTGAGTATTTTCTGGAAAAAATGTTTTTATGGGACAGTTACGAAGAGAGAGAAT
TGCGGAAAGAATGAAAGCTCTTCAAGAACTCGTTCCTAACGGCAATAAGGTAAATTTTTATT
CAATTCATTTTCAGTTTAGTTTCCAAAAATTAAGATTAGTTAAAATATTAAATGGATCATTATG
AAAATAAACTTCACTTTCCATCATTTTATTCATCATCCACTCTTGATTCGTGCGATCCATAATT
CCATCTAATTTACAATTTAAACTCCATCGTCTATTTATAGTTCTCATCTTCTAGACCTAAAATT
GAAAAAATACAAAATAAATAATGAAAATAAGTGTCGAAATGATCAGGAACTTTCTTGGAGC
TAGTTGTTCAAGAGATAGTTTTAGGCTGTCAACTAGAACTTTCAGGAGTTAACAAGTAAATT
TATATTTTTTGTTTTACTCATATAATTGGTGGTTGTGTAGTCTATCTATATATAGGAGTAGCTAG
TAGTATATATCAATCAAATGATTAAACACTGATTAGCTAATTAATTAATTAGGCTACAATAATA

AAGTTAACTAATGCAGTAATAAATGAGTTAGGATCTCAGTTCTGACAGTTTTGTTTTTGTATT
TTATTTGTATAATAATGGACAGACAGACAAGGCATCGATGCTCGATGAGATCATAGATTATGT
CAAGTTTTTACAACTCCAAGTCAAGGTGAATCTCTCCCTCTCTCTCATATATTTACAGTTATTA
TTTTATTAAGTTTGTTTTTCATACGTACAAGGGGTAATTCTTTTACAGAGTGCAATTTGTTTTA
TTTTATTCTCTTCAATATAGATGGAAGATTCGTTTTTTTGCCTTTTAGTTTGACATATTAAACA
AAAAGAAAAGCTGACATATTAGTACAATCTTTACATACCAATATATCAAATTTATGGGCCTCT
GCAACTAAAATTCCTTATGCTCCCTCCTCAAATTTTATATATACATTCTGGATTATTATGTCATA
TAAATACATACATGATAAATAATGTTTTTAAGTTATAAATTAGTGGACCAGTGTACTTAATCAT
TCTGTTTTGAATTTAATTATGCGTTTTAGGTACTGAGCATGAGCAGATTGGGAGGTGCTGCTT
CCGTTTCTTCTCAAATCTCCGAGGTAACTAATATATTACTATCTCTACGTACTCATTTTTCATA
AATTTCAATAAACATCAAAGCCAATGGACAAAATTAAGAAATTAGACAATCACTTTTCTCTT
ATAATGTTAATTAGATTTTACAAAATATTATTTTGGTGTGTAAAAATAGATAAATCGTGCAAGA
CGTTGAAAACAAAATGAATGTAAAAAGTTTCCAGAAACAGAAAACAAATAAGACAAATTTT
ATAATTCTAAAATAGAGAAATATAAAAGAGAGCACGCGTGCCGACTAATCACTTAGGGTTTC
GCTTTCTTTGTCTTTTTGTTTTTCAAAATGTTTTGTTTTATTTTGCTTAGGCTGGTGGATCCCA
CGGGAACGCATCCTCCGCCATGGTCGGCGGTAGCCAGACGGCCGGAAACTCCAACGACAG
CGTTACAATGACGGAACATCAAGTGGCCAAACTAATGGAAGAAGACATGGGCTCGGCCATG
CAATATCTTCAAGGGAAAGGTCTTTGTCTCATGCCAATCTCTTTAGCCACCGCCATCTCAAC
CGCCACGTGTCACTCCCGTAACCCTTTGATCCCTGGAGCTGTTGCCGACGTCGGAGGTCCT
TCCCCTCCCAATCTTTCTGGCATGACCATACAGTCGACGAGTACAAAAATGGGTAGCGGTAA
TGGGAAATTAAACGGTAACGGCGTGACCGAGAGGTCGTCTTCTATCGCCGTTAAAGAGGCC
GTATCCGTTTCGAAAGCGTGATAACGGCCGTTTACTTTTGGGGTTAGGCAGAGAGATACCAA
AAACAAAGAGAAAGTGGGGTGAAGTGGGAGATAAAGTCAAAGTCTACGAAGAATGAGGT
TGAGGTTGTTTGATGTGTTCTCTGAACGAAGCCATATTTTTCGTAGAGTTTTGGTTCTTTTAC
CTACGCACTACAAAACCATTCAAGGGACATTTCTTTTTCTTTTTTAAATATATGGTTGCAATAT
GTTTTATTTTTATTAAATAAAGTTGGTATCGTTGATCTGAATATTAGGCTAGAAAAAGAAAAA
AATAGTATATTGGTTTTTCACTTAGAGATGGAGTTATAATCTCGTCTCTCAAATCTCAAATATT
GAATCCAAGACTTTAGATGTTTATGGATCGCACAAGTCTAAATATTAGGATATGAGTTTGAAG
AATATACAAATTCTGT

>AtbHLH067
CTGCTCTGCTCAGCCTCACCAACTCTTACAGTACCCAAATACATCTATCTTATTACCTCTCTG
TTTTTTACAGCTCCTCTGTTTCCACTCTACTCAATAGCTTTACTAAAGACTCACTGTTCTATAT
AAAGCTAACAGTTACAAGTCCATAAAAGCTCAGAATCAGAAGAGAGTACATGAGTTTCCTC
TTCTGCCTCTTTGGTGATTCTTAGCATTTATTATTACTCACTCATCTTCTTTTCATTACATTACA
TACCAAACAAGAGCTCTCAAATGGAAAGGTTTCAAGGACACATCAACCCCTGTGTATGTAT
CTTTTGCTTGATTTAGAAAACTTTTAACTCTCTTTCTTCAATCTTTCTCTCTTCCCTTGAATCA
TTTTTTTCCATACTCAGAGACATGGACTTGCATTTACTCATTCCTAATTAGTCCAGATACTGT
GCATTTAATGCTTTTGTTGTTTGTTTTAGAGAAAACAGTAACAATTCCTAATTAGTCCAGATA
CTGTGCATTTAATGCTTTTGTTGTTTGTTTTAGAGAAAACAGTAACAATTCTACAATCATTTT
TGCTTCCCTTTCTTTGAAAACATTCCATTTTCCTTTCTATTTTTTTGCTTATCTCTGTACTAAG
AACCAAACTAACCCACAGTAGAACTAAATTACACTTCTCTTTCAATCATTTGCGTCTCACCG
GCTAATCTCATTCCTCTGTCATTTTTACAGTTCTTCGATCGAAAACCGGATGTGAGAAGCCTC
GAGGTTCAAGGATTTGCAGAGGCTCAAAGCTTTGCTTTCAAAGAAAAAGAGGAAGAAAGC

TTACAAGATACAGTTCCATTTCTACAGATGCTGCAAAGTGAAGACCCCTCATCGTTTTTTC
AATCAAAGAGCCAAACTTTCTGACGCTACTGTCTCTTCAAACCCTCAAGGAGCCTTGGGAA
CTCGAAAGATATCTTTCACTTGAGGATTCACAATTTCATTCACCGGTCCAATCTGAGACCAA
CCGCTTCATGGAAGGAGCCAATCAAGCTGTGTCAAGCCAAGAAATTCCCTTTAGCCAAGCA
AACATGACACTCCCTTCTTCTACCTCATCACCACTCAGTGCACATTCAAGACGAAAGCGCA
AAATCAACCACTTGCTGCCTCAAGAAATGACTAGAGAAAAGAGAAAGAGGAGGAAAACA
AAACCAAGTAAAAACAATGAAGAGATTGAGAATCAAAGAATAAACCACATTGCTGTTGAA
CGAAACAGAAGACGTCAAATGAACGAACATATCAACTCTCTCCGGGCCCTTCTCCCACCTT
CCTACATCCAACGAGTAAATAACCAAAAGCATACATGAAAGTTTCAACCTTTAATCATCAAA
ACCTTATCAAATGTTGTCCTGCAGGGAGACCAAGCTTCCATAGTAGGAGGAGCAATAAACT
ACGTGAAGGTCCTCGAGCAAATCATACAATCTCTCGAATCGCAAAAGAGAACGCAACAAC
AAAGTAACAGTGAGGTAGTAGAAAACGCACTTAATCATCTCTCAGGCATTTCGTCGAACGA
CCTGTGGACAACTCTTGAAGATCAAACTTGTATCCCCAAAATCGAAGCTACAGTGATACAA
AACCATGTCAGCCTTAAAGTTCAATGTGAGAAGAAACAAGGACAACTTCTCAAAGGAATCA
TATCACTTGAAAAGCTTAAACTCACTGTTCTTCATCTCAATATCACTACTTCGTCTCATTCCT
CTGTTTCTTATTCCTTCAACCTCAAGGTAAACACTACTTTCCATTTTTTACTTCAATCAACAA
AAACCCAACATTGAAAATTGAATCTTTAATTAAAAAATCTTGTTTTTTTTTTCAATTGTGTCG
GAGCAGATGGAAGATGAGTGCGACTTAGAGTCAGCCGACGAGATTACGGCGGCTGTTCATC
GGATTTTCGATATTCCGACAATTTGATTAAACACATATAATTCCAAAAAATATTAACAGCTGAC
AAAATGGTATCTTTTTTTTTTTTCCTACTTGTACCAATTTTTTAAGAATTAAGAGAATAAAAA
AATTTCGTTTTTCGTTCTTTCATTTTCCTACTGACAGAATTAAATTCTGT

>AtbHLH068
CACATGTTATCAAATGAGATCAAAATTTAAGTTAGTTTTGAGTTTTCTCTCTCTTTCTCTTCTC
TAAAACTACTTAAGCTAAAGAATCAAACACTTCTCAACGTTTCTCCAAAGCCTCTGATTAGT
AAACACATAAAGAGATCTATAGAGAGAGAGAGAGTAGAGATAAAAGGTGATGAATAGAGG
TGTGTTGGAGAGTTCGCCGGTTCAGCAGCTGATGGCAGCCGGAAACCCTAATTGGTGGAAC
GTAAGCGGCGGCATGAGGCCACCACCACCGTTGATGGGTCATCAGCAGGCGCCGTTGCCAC
CACATATGACTCCTAATAACAATTATCTCCGACCACGGATGATGCCCACTCCCTTCCCGCACT
TCTTGCCATCTCCGGCGACTTCTTCTTCTTCCTCTTCTTCTTCACCGTCTTTGCCTAATAACCC
TAATCTCTCTTCTTGGCTTGAAAGCAATGATCTCCCTCCTGAGTCTTGGAGCCTTAGCCAAC
TTCTTTTGTAACTATCCCTCTATCTCTCTCCCTCTATAAATATTCCAAAATGAAATATTCTCTAT
ACGCACATGACATGATCTTGTGTTTACTTACTAGTTCACTCTTTGTTTTCTCGTGTGTTGCTA
CTTAGTCCTAATTACCTTTTTGTTATACGTATATACGTATATACATGTATGTGTTTGTATTTTAAG
TATCAAACAAACAATCTAATATTTTGGATATTTTCTTTGTGAACTTAGGGGTGGATTGATGAT
GGGAGAGGAAGAGAGATTGGAGATGATGAACCATCATAATCATCATGATGAACAACAACAC
CATGGTTTTCAAGGGAAGATAAGATTGGAGAATTGGGAAGAACAAGTGTTAAGCCACCAAC
AAGCTTCCATGGTGGCTGTTGACATCAAACAAGAGGGTAACATTAACAACAACAATGGCTA
TGTCATATCTTCCCCGAACTCACCTCCTAACAAATCTTGTGTTACAACAACTACCACAACAA
GCCTCAATAGTAACGACGATAACATCAACAACAATAATAATATGTTGGATTTCTCTAGCAATC
ACAATGGTCTTCACTTGTCAGAAGGGAGACACACTCCTCCGGATCGATCCTCTGAGGTAAC
ACTTTTTCCACATCTCAGAGATTTTGATAATATACGGAAAAAGCTCGGAAATAATATCTAGTT
TTACACATAAATTAGTAAACACATCAACTACTATGAAAAAGTGAAATCTAAAACGTCATTTA
AAAAGATCAGGACAACATATTTTATTTTAAGGTTTGGATCTAAATTCAACACGCAACCAGGA

TGAGTTTTGCTCTGAATTTTTTTTGATTAAATGACTAATTGATCTTTGTTTTGTTTGTTATCTAT
ATATGTGATGCAGTGTAACAGCTTAGAGATTGGTGGGTCTACTAATAAGAAGCCAAGGCTCC
AACCTTCTCCTTCGTCACAATCAACCCTCAAGGTAACTAATTGATTATATCAACGAAACTTTG
GATAACTTATATAACGTTTTCTGTATATAAATTCCCTCCCTGACATCATACTAACAAAACAAA
AACACAAACACAAAGGAGAACAGAAGAAGCAAACAAAACAGAATGGGCACAGAATAAGC
TGTGTGGTTTTGTGTTTTGTAACTCTTTTGTTCTCTCCTGTTTTGTCTTCTTCTTCATTCTTCA
ATATTCCTTTCCTCTCTTTATTTCATAAAAATCAAACTTAAATTAATAAATTTCAACATTCTCC
TTAGAATTAGACGACACTATATGTAATAAATTTTATTTTATAGAATATGGACATTTTGAGAGAT
TTCAAAGTTCTGTCAAAAAAAAAAAAGAGATTTAAAAGTTTTGTTTTTTTCTGTAACTTGTTT
TGAAACGTAGAAATGGAAGAGTCGCATTAGACATTGGATTCAATAATATCATGATAATTATAT
ATACTAGTGAAAGTAAAATAATTGTTTATCACTTGTACATGCTAAGTAAGCACGTTTTCTCTA
TGCATGGTAATTAGTAAAAATAATATGACCACACATGCACATATATATAGAAAGCTTTTTAATT
GATGCTATTAATTAATCTATGGTGGTAATTAAAGGTGAGAAAGGAGAAACTAGGAGGCCGAA
TCGCAGCGCTTCATCAGCTAGTATCTCCATTTGGAAAGGTAAAGATCGATTCCATTAACAAA
TCAAATTTATTAATGTCGTCTAATATTCCTCTATCAAATCAACTTTTAACATTAATTGTGGAGA
TTTTTTTAGTTTCATTCTTGTTAATCTAAAATCTAAATCATTCTTCATCCCTTTTTACCCATCTT
TTGCAGACTGACACAGCCTCTGTCCTGTCGGAAGCTATTGGATACATTAGATTCCTTCAGAG
TCAAATTGAGGTGTGACTCGTCTTTTCCAAACTCCAAATATTCTCATTCAGTAAAATAAAAAT
AATGCTTATGATTACTCCGTTTACCAAAAAAAAAAAAGAAGAAAAAAAAGGTTGTGATTACTG
AGAAATAAATTACAGAATAAAGCATAATGATATTATATAAGAAAGAATAAAGATAGTAGTATA
AAGTTTTGTTCTCTCCATGTTAAGTAATATTTGATTGATGTGATTATATATAAAGTATTTATGAC
TTTTTGCAACTGTTGCACTGTTGCAGGCTCTGAGTCATCCATACTTTGGTACGACTGCCTCG
GGGAATATGAGGCACCAACAACATGTAAGTAGCAAATTTTCGTATATATACTGATATACACAA
ACAAATCATCATGCTATCGTTTTATACCCTTAACTTCTTCTTTTTCGATAACCTACACGAAACT
TTTTATACTTATATATTATCTCTTAAGTCTTAACTATGGTTTATATGAATAGTTGCAAGGAGATA
GGAGTTGCATATTTCCTGAGGACCCTGGTCAGGTACGTCCATTTTTTCTTGGTCACTATACTT
TTAATATATAATTAGAGTCACTATACTTTTTATTGGATCAATTGAATCCAAATCTAAAAAATGT
GGACAGCTTTAGTTTCCAAAATTTATCCTTTTTTTTCTTTTGTTTTTTTTCTTTTCTACTGTTTT
CTTTAAAGTAAAGTTGGATTAATATTAAAAGATGCAGCGGCCAGCTTTTTTTTCACCCTCCCT
TACATTATTATAAACCAAAAAAGTTCGTTAAAACCAACAAAGCCACTAAATTTATTTAAATAT
CAACTTTTTCTCCTTACTTGTTTTCTTCATCTAAGTGAAATTCAAATTCCTTTGGCGGGGTTT
TATATATGCTTTTCACTAGGGGAAATTCAATGATTTTTTTTCTTATCACCTCTTTATATAGGTGT
TAGAGAACATGTGTTTACTTTTTTTGTTAATCAATGCTATATATCATGATTGTTTTTTTTTTTTT
TTGATATGGTTTTAGCTCGTGAACGATCAGTGCATGAAGAGAAGAGGAGCTTCGTCGTCATC
TACGGACAATCAAAATGCAAGTGAAGAGCCTAAGAAAGATCTGAGAAGTCGAGGTTTATGT
CTTGTCCCAATCTCATGCACACTCCAAGTAGGCAGCGACAACGGCGCGGACTATTGGGCTC
CAGCACTTGGCTCCGCCGGTTTCCATTGAGCCAATGAAATTACGCCACGTGGAATTCCAGC
GTTAGAGAAAAATGATATAACATCACGAGCAGTCAGTCTACTGAGAAAACAACAAATTGAG
TTTCCAAGGCTGATTGCTCATGATGCTATACAAAGTTCTTCGTTTAAAACTTTGCTAGAGAAT
TTGCGTCTGGCGTCTGATGAATATGCGTTTGGCGTTTCATCATTTGATGCGTTATCAGATGATA
TGACCAAGTGATCAATTTATGTTCTTGCTTTAGGAAACTGTTTGTTTTCTTTTACCGGTTAAT
TGTAATTTGTGGTTAAGTACTTATTTTAATTACTTCCACGTGGTTTGTATCGTTATAATTTTGCT
ATTACTTATAATATGTCTATTGTGTGGGATATATACGACATGCAGACCATTAAAATAAAAAAGT
GTGCCGGAAAATATCGATATAAGTTA

>AtbHLH069

GGTAAATTTGGTAAACGTATTTAACATGTCAAAGTCAAACCCTTCAAATCTCAAATATTACAA
TCTCCATCCCTCTTTTAAACACCATAAAGCCTTAAACCTCAAAAGACATTTCAACAACATTC
ATTTTCATTCTCACTCCCCACCAAAAAAAAAAAAAACAGAAGCCATGAACTCCTCGTCTCT
TCTAACTCCTTCATCATCTCCTTCTCCACATCTTCAATCTCCTGCAACATTCGACCACGATGA
TTTCCTCCACCACATCTTCTCCTCCACTCCTTGGCCCTCATCCGTTCTCGACGACACTCCTCC
ACCAACTTCCGATTGTGCCCCCGTCACTGGATTCCACCACCACGACGCCGATTCAAGAAAC
CAGATCACTATGATTCCTTTGTCACATAACCATCCTAATGACGCTCTCTTCAATGGCTTCTCC
ACCGGATCTCTCCCTTTCCACCTCCCTCAAGTATAACTGATAATCCACTTTTTCTCTCTCATTT
TGCAAATTACTACAAAAGAGAAAACATTCATTACATGTTCTTTTGTTAAGTCTTAATTTAATG
TTTTCTGATATATATTTTAGGGATCGGGAGGTCAAACGCAAACGCAGTCGCAGGCGACGGCG
TCAGCCACCACCGGTGGTGCAACGGCGCAACCTCAGACAAAGCCTAAAGTCCGAGCTAGG
AGAGGTCAAGCCACTGATCCTCACAGTATCGCCGAACGGGTTCGATTTTTTTATTTAACTTT
TCCTCATTATTCGCTTTTCAGTTACCGAGAAAAACGTATAAAGTTATTTAGATGATTAAGGTG
ATTATTGAAATATTTTGAAATTATGGAACAGTTACGGAGAGAGAGGATAGCGGAAAGAATGA
AATCTCTTCAAGAACTTGTCCCTAATGGTAACAAGGTAAATTTTAATTATCTTCATTAGTCTA
AATTTCGATCATCAAAGTTGATATAATTAAAAGAGTAGATTCAAATAATCCAATTTGTATTTTA
CATTTCTTTAATAGTATATATCTCAAAATGAATAATGTGTTAAGTATATGAGTTACACTGATGTA
TTTTAGTCTTGGACCACAAATTGCTACTAGTTTAGTAGGTTATCTAGAGATTTGGACTTTTAT
GCAGTCAAATGATTAAACACTGATTAGCTAATTAATTCTAAAATTTAATTGATAATAATACTTA
ATAAACAGATGCAGAATTGCTAACAAGGTTTTTTTATTGTTAAAAATAATAATTAACAGACA
GACAAAGCATCAATGCTCGATGAGATTATCGATTATGTCAAGTTCTTACAGCTCCAAGTCAA
GGTGAATCTCTCTCTCTCTCTCTCTCTCGAATTATTTTATAAGATATGAGAATTTTAAATTTGTTT
TTGTTATAAGAGTGGAAGCTTCTTTACCTTTGAGTTGACATTAGAATAAGAAATCGAATATGG
GCCTCTGCAACTAAAATTCCATATGCTCCCTTTCCCTTTTATCTTGTAAATTATTTATCTTTTGC
GTTTGTTAAAACATAGTGTTAGTGTATATCTACCTCTTCTTTTGCTTAATTATAATATTTGGCA
AATTTATGTATGCAGGTACTAAGCATGAGTAGACTGGGCGGTGCTGCTTCTGCTTCTTCTCA
AATCTCTGAGGTAATAATTTCAATTCCTTCTCTCTTAAAAGGGATGATAGGATGACGTGATCA
CTATATAGAATGAGATTTTATTTTTTAAACTAATTAAAAAGAGATGTAAATTATTGAAAAGGT
TTTAGTTAGGGAATAATACTAATTAAAAAGTAGTTTTGTTTTCGACTTTCTAAAAACAAATGA
AAAAGTTTTCCAAAAAATAGTAAAAAAAACAAAACAAGGACAGGGCACGCGTGCCTACTT
TCTCTCCAACAAATACCAAAACCTCCCATTTCAACATCATCATAGCTTTAGGGTTTTGCTTTT
CTTGTTTTCTTTTTAATTTTATTGAACATTTTCAGGATGCCGGTGGATCCCACGAAAACACCT
CCTCCTCCGGCGAGGCGAAGATGACGGAGCACCAAGTTGCAAAGCTAATGGAAGAGGACA
TGGGATCAGCCATGCAATATCTACAAGGCAAAGGTCTTTGCCTCATGCCCATCTCGTTAGCC
ACCACCATCTCCACCGCCACGTGTCCTTCTCGTAGCCCCTTCGTTAAAGATACCGGCGTTCC
TTTGTCTCCTAACCTATCCACTACAATAGTTGCTAACGGTAATGGCTCATCGTTGGTCACCGT
TAAAGACGCTCCCTCCGTTTCCAAGCCGTGATAACGGCCATTTGTCCATTTCATTTTCCCTTT
TTTGGGTGGGAAAGAGAGAAAAAAGTTTAGAAGACAAAGACAAGTGGGATAGGTGGTTTT
GGTCAAAGTTTAGAAAGAATAAGGTCGTGTTTTCGGATACGACACCGTATTTGCGTACACTT
TGGTTTTCTGTCTTTACCTACTACAAACCACCCATAAGCACACTCATGTTATCATGTTTTTTTT
TTTTTGGTTTATAAAGTTATATCCTTAAATTTTCTATCACAGAACCGGTCTGGTCTAACAAATG
TCGTCAATGATGATCATTTAGGCCAAAGAGTTTGTTTAAAACTCGTTATGCTTAAGTTTCTAA

ATCTACACAACACAGGAATGTAACAC

>AtbHLH070
ATGTTTGTCCTGAGAGTAAGTAACCAGAGCTTTAAGTTACATCAGCAAGTACAATGTAAAGA
TGAAATCTTCGTAAGTCATTCTTAAAAAAACTATGTAAAAGTTGACTCTTTTTTTTACTTGGT
AGGCGGTTCTGGTTCTCTTATTACACAGTAGTATGAACGAGATCTCTAGATTGCAGACAACT
TAATTCGTGTTTTCTGGCAAGTTTTTGTGTTGGATCTTGTTTTGGCTGTGTTAGACTTACCAC
GTGAGATAAACAGAGGCCACAATTGAGAGAACATGGATAAGGTTTCTTCTTGTGAGCTTATT
AAGATCGAATCTGTGTTCTTGTTTGTCAACAAAGAGGTTAAAATTTCGGATTTGATCGATAA
CAAAATGTACTTCTTTGGAATCCTGTAAATATTACAAAGAAGTAAAGGTTAAAAAGGGAATT
TTACCGTTTATTCGATAGCTCGTAAACACAAAATCTGATGCAAAAACAAAGATATTTACAGG
CAAACACTGGTTAGTACCCTTAAAGCCAAGGCATCGACAATTACATGTTTAGCAGATAAGA
AGCCTCAATTCACGGGTTAACGAAATAAGAGACTTCAAAAGATTCAATTTTTATCTATTGGG
TTGTAACAAAAATCAAGCTTTTCATGAAATGTAAGAACACAAAACCAATGGAAACTCTCTT
CTCTTCTACTTCTTCTTCTTCATCATCATCTTCCGTGTGTTAACAGAAGTCCCCACAATTGTC
TGTCTTCGCTGCGAGACAAAACTGCCACAGCCAATAATGTTTCTCTGAGGGACCTTGCTTCT
GTCAGAGACTCGCTCTCTCTCTCCTCTTCTTGCTCTGCTCAGCTCTCTCACCAACTCATCTTC
AGTCCTCAAACAAACATCTGTTCTCATCTTTGTTTTCTTTCCTTTCTTTCTCATATCTCATTTT
CAATTTTCCCAATTTCTCTTCAACATCTTCATAGCAATTTAAGACCACTATTCCATTATAAAGC
TAACTGCTTTAGAAACTCCTCACATTTATTTCTTCCCCATCATTGTTTTAGAGAGGGAGAAAG
AAAAAGAGCTCAGCTTTCTGATGGAGAGGAGTATTCAAGGACAAAACAAGCTCTGTGTAAT
GTATCTTATCTCAATTTGGAACGTTACTAGTTTTTATCTTTCTTGTAATCTTTTCTTTCAACTAT
TGATTCAAAGATGTGGACTTGCATTTACTCCTTCCAAAAGAGTCCACAATGGTTGCATTCAA
TGCTATATGTGTTTGTTATACCAAAAACACCAACAAATCTGCTCTCAACTGTTTCCCTCTCTT
TGAAACATTCCATTTTTCATTCTAATTTTGTTTCGCTAGACTGACCCACTAACCAAAACCCAC
ACTAGTCTACCCAAATTTAATATCTCTCTTCTCAATCTCACTTTCTCTTCTTACTCACTTGGTA
GGTAACTCCTTCCCCTGTTTTTTCTTTTGTTGCAGTGTTTGGACCAAAAAGTGAATGTGAGA
AGAAGCCTACAAGTTCAAGAAACTGTAGAGGATCATCAAAGCTTTGCCCTTGAAGAGGAA
GAACAACAACTCTCAACTCCGAGCTTGCTGCAAGACACAACAATACCATTTCTACAAATGC
TGCAACAAAGTGAAGACCCTTCACCGTTTTTGTCATTCAAAGACCCAAGCTTTCTAGCACT
ACTATCTCTCCAGACACTTGAAAAGCCTTGGGAACTCGAAAACTACCTCCCACATGAAGTT
CCAGAGTTTCATTCACCGATCCATTCTGAAACCAACCACTACTATCATAATCCATCTTTGGAA
GGAGTCAATGAAGCCATCTCAAACCAAGAACTTCCATTCAACCCACTAGAGAATGCGCGTT
CAAGACGCAAGCGGAAAAACAACAACTTGGCATCATTGATGACAAGAGAAAAGCGAAAG
AGAAGAAGAACTAAACCAACAAAGAACATAGAAGAGATAGAGAGTCAAAGAATGACACA
CATTGCGGTTGAACGAAACCGCAGACGCCAAATGAACGTTCATCTGAACTCACTCCGCTCC
ATCATTCCATCTTCATACATCCAGAGGGTATAATAAACAAAACCCACAAATCAAAGTTCCAA
CCTTTATCAAATGTCGAATTTTCTTAACTAATATCTCTCGTAGGGAGACCAAGCGTCAATAGT
AGGAGGAGCAATAGACTTCGTAAAGATCCTAGAGCAACAGTTGCAATCCCTTGAAGCACAA
AAGAGAAGTCAACAGAGTGATGATAACAAAGAGCAAATTCCAGAAGATAACAGTCTCAGG
AACATTTCGTCGAACAAGTTGCGTGCGAGTAATAAAGAAGAACAAAGTAGCAAACTCAAA
ATCGAAGCCACAGTGATAGAGAGTCACGTCAACCTAAAAATTCAATGTACGAGGAAACAA
GGACAACTTCTCAGATCAATCATATTGCTGGAGAAACTTCGATTCACTGTTCTTCATCTCAA
CATCACATCTCCGACCAATACATCTGTCTCTTATTCCTTCAACCTCAAGGTACTTACTTATATA

ACAAAAAACCCATTATTCAAAATCATCGACAATGGTGAAAAAATTCAATCTTTTTTGTTTGT
TGCTCAGATGGAAGATGAATGTAATTTGGGATCAGCGGATGAGATAACGGCGGCGATTCGTC
AGATTTTCGACAGCTGATTGACTAATCCAAGTAAAAAGTAAAATAAAAAAAGAAACGTTTA
CTTTGGTAACTTCGTTTTCATGATTAAATTCTTTATTTGGTCGTATGTGATTGGAGTCTTCTCG
GCATGGAACTTGACTTTGGTTTTAGGGTACTAGTCTCTACAGAAGCTGTGGTCCTTCTTTGG
ATAGATTTATAATGAAAGTTTGACCCCTCTCTCTTTTTTTATTGTCTTTTTGTTTTGCTTTTGCC
TCTCTCTCGGGGTTTTGACTTTTGTATCATAAAACTAGAATATAAAAGTTTACAAATTATATTT

>AtbHLH071
ATTTTTTTCGAAAACAATAAATTAAAGATTTGGAATGTTGCTATATAAATCAATGATACTTAGT
AATTCAATAAGGAATAAACTTCGTGAAGAGAAATGACTCTAGAAGCTTTATCATCAAACGGT
CTTTTAAACTTTTTGCTCTCTGAAACTCTTTCACCAACTCCATTCAAGTCTCTCGTCGATCTC
GAGCCATTGCCGGAAAATGATGTCATCATATCGAAGAACACAATTTCGGAGATATCTAATCA
AGAACCGCCACCACAGCGACAACCACCAGCTACGAATCGAGGGAAGAAGCGGCGGAGGA
GGAAGCCTAGGGTTTGCAAAAACGAGGAAGAAGCTGAGAATCAACGAATGACTCACATTG
CCGTCGAAAGAAATCGAAGAAGACAAATGAATCAACATCTCTCTGTCTTGCGATCTCTCAT
GCCTCAACCTTTTGCTCACAAGGTTTAACTTTTATGGATATATATTCTTTTTAATGTTTGTAAA
GTTTCAAGTAGGGTTTCAATTTTTATGGATAATTTTTTTAAATGGTTTACTATTTCGATTTGGG
TTTCAATTAATATTTACAATTCACTGAAAAAGATTAAATTTTCATCTTGAGTTTTATTTTTTATG
AAAGATTCATTCTAACGGTTTAAATCTATTTGATTTTCAATATTTATGGCCAATTCTAAAAAAT
TGATTTTTTATTAAATATATATTTGGGTTTTTATTTGTTTACTTATATGTTGTTTTGGTGGATTTA
TTTGTTTCCCCATGAAAGGGTGATCAAGCTTCAATAGTTGGTGGAGCCATAGATTTCATCAA
AGAACTTGAACACAAATTACTATCTCTTGAAGCTCAAAAACATCATAATGCTAAATTAAACC
AGTCGGTTACTTCTTCAACAAGTCAAGACTCAAATGGTGAACAAGAGAATCCTCATCAACC
ATCTTCACTATCTCTATCGCAGTTCTTTCTTCATTCATACGATCCGAGCCAAGAGAATAGGAA
CGGCTCAACAAGCTCGGTGAAAACCCCTATGGAAGATCTTGAGGTGACTCTAATCGAAACT
CATGCTAACATCAGAATCTTGTCGAGAAGAAGAGGTTTCCGGTGGAGCACGTTGGCCACCA
CCAAACCGCCGCAGCTTTCGAAGCTGGTGGCTTCTCTACAATCGCTGTCCCTCTCCATTCTT
CACCTTAGTGTCACAACATTGGACAATTATGCTATTTACTCCATCAGCGCTAAGGTAATAAAA
TTAAAACCCTATCATCTGAACCGGACTAAACCGGATTAGTTTCAATGCAATTTGTGCGGTATT
TAGATCAAACAGATTACAAGATAGGACATTTATGCTATTTACTCTATCAGTAACTGATGAAAA
AAAAAAAAAAACTCTATCAGTAACTAGGGTGATACAAACCGGAGCTAAACCGGATTTGTTC
AAATGCGACTTGAGGGGTATTTTGGGAATATATGTTAGGAATTTAAAGCTAGACGGTCGATTA
TGTGCGGTCCAAGGGACAGAGCATATAAAAGGAATGTCTATGAGCTCTTAACTAGATTTGGT
CCAACATTTATTACCATACTTGGTGGAGTCATTTTTCTTATTTTTCACCCATTTCTTGAAAACA
GGTTCAATTATTGCCACGTGGGTTATAAAAATTGTAACAATTTGAAGCTTTTTTCAAATTCAC
ATTATGCTATTTTTGTAAAATCATTTTCTTATTGGATGTTGAAAGGTGGAAGAGAGTTGCCAG
CTAAGTTCAGTAGATGACATTGCAGGAGCAGTTCACCACATGCTAAGTATCATTGAAGAGG
AGCCTTTTTGTTGCTCATCAATGTCAGAATTACCATTTGACTTCTCTTTGAATCACTCAAATG
TCACTCATTCTCTCTGAGAAATCTCTTTTTTTGTTGTTGTTATTCCTTCTTTTAATTTTATCACAT
AGCACATCTTTAGTTTTTTTTTTTCAGAATGAAGAAACACATAAGCTTTCTTTTAAAATCCTA
GGTTCTTTTTCTGACTTTGTAAAGGCTAAATGTGAATTTATAATGGCACATAACATATTAGTT
GTTTTACTCTTAATTTTATGCATTTTCTG

>AtbHLH072
CACAAACAATCCTTTGCCACACATGTGCTTCGTCCACATAAAAACCATTTGACGTTTTGCAG
GGACCATTTTTGAGAAAGAGGAGACAAGAAGAGAGAGAGAGAGAGGCCTCTTTATTATAA
CTTTCAATATGGATCAAAGGCAGATACACCGCATGAGTTGTCGCTGTGATTGTCAAGAGTTT
GAACACACAAAGAAGAAAGAAGAACTCAACATTTCAAGCAAGAAGAAAGAGAGAAGAG
AGAAGGTCCAATAATAGAGAGAACAAAAAAAAAGAGAGCTTAATTGTCAGTTTATTCTCTG
CAAACGTGCGGCCTAAGTAACACATGTATCTCTCTCTGTTTCTGATAAAGAACTCTTCTCTG
TTTACACCAAAAAGAGACAAAACCTTACGCTTCTGTCCTTTTTTGTTTATCTCTCTCTCTCTC
TTAAGTCTTAACACAAAACCAATTTCTTTTTTTTCGTGACTCAGGTCGAATTATGGAGTTAA
AGAGCTCACATGGGAAAATGGGCAACTAACCGTTCATGGTCTAGGCGACGAAGTAGAACC
AACCACCTCGAATAACCCTATTTGGACTCAAAGTCTCAACGGTTGTGAGACTTTGGAGTCT
GTGGTTCATCAAGCGGCTCTACAGCAGCCAAGCAAGTTTCAGCTGCAGAGTCCGAATGGTC
CAAACCACAATTATGAGAGCAAGGATGGATCTTGTTCAAGAAAACGCGGTTATCCTCAAGA
AATGGACCGATGGTTCGCTGTTCAAGAGGAGAGCCATAGAGTTGGCCACAGCGTCACTGCA
AGTGCGAGTGGTACCAATATGTCTTGGGCGTCTTTTGAATCCGGTCGGAGCTTGAAGACAG
CTAGAACCGGAGACAGAGACTATTTCCGCTCTGGATCGGTAACAACTAACAAAATTACTCTT
CCTTTTTTTCCTGAAAGATGACTGGAGAAATAACCAAAAGAGGACTTTAATAGGAAACTCA
AGATACTGAAGGAGATGAACAAGAGACAAGAGGAGAAGCAGGTAGATCTAATGGACGACG
GGGACGAGCAGCAGCGATTCACAACGAGTCCGAAAGGGTAAAAACTAATCCATCTTTTCTC
TTTCCGTGATTACCAAACTACTGACTCTGAGTTTTGATGCAGAGACGGCGTGATAGGATAAA
CCAGAGGATGAGAACACTTCAGAAGCTGCTTCCTACTGCAAGTAAGGTAAATGTCTATCCT
AGAAGAACCCACTTTTAAGTTTAAGAACTTTGTTTATATTGATATCGGTCAGTTTAAACGCGT
TATTCGGATATTGCAGGCGGATAAAGTCTCAATCTTGGATGATGTTATCGAACACTTGAAAC
AGCTACAAGCACAAGTACAGTTCATGAGCCTAAGAGCCAACTTGCCACAACAAATGATGAT
TCCGCAACTACCTCCACCACAGTCAGTTCTCAGCATCCAACACCAACAACAACAACAACA
ACAGCAGCAGCAGCAGCAACAACAGCAGCAACAGTTTCAGATGTCGTTGCTTGCAACAAT
GGCAAGAATGGGAATGGGAGGTGGTGGAAATGGTTATGGAGGTTTAGTTCCTCCTCCTCCT
CCTCCACCAATGATGGTCCCTCCTATGGGTAACAGAGACTGCACCAACGGTTCTTCAGCCA
CATTATCTGATCCATACAGCGCCTTTTTCGCACAGGTAAGACCGCTTTTAACTGTTTTCGAGA
AAAGTGTTCAAAGATATACAAATCTTGAGCTCACTTCCATTGGCCATGCTGATGCAGACAAT
GAATATGGATCTCTACAATAAAATGGCAGCAGCTATCTATAGACAACAGTCTGATCAAACAA
CAAAGGTAAATATCGGCATGCCTTCAAGTTCTTCGAATCATGAGAAAAGAGATTAGTCTAGC
GACCTAGTATTATTGATCCATATATATAGTTCTTGAAAGATTGTTGTATCATGATTGTAAAAAC
TGTTTTGAGTATGGAAAAAGACTTGCAGATAAAACTTGAAAGCTTCACAACTGAATTCAGT
AACTGTCAAAGTACATTACTAAACCCTTTGCTATTTCGAGGACAGTAACAAGAAAATTATCT
CTTGGTCGCTCTTGAAGTTTAAAATCTTCAATGTTAATAGATCATATGCACAAATAAACTTTT
GTCTTTTTTTCTTTCTTTCATTGTTCGGAGACCAAAGGTCTAACCTTAGAAGCAGATGTTGT
GGCCTTGCTCTGAGGTTTGTGAGATGTCTTATTAGTGTCCTGAAGAGAGATAACAGTTTCAA
AA

>AtbHLH073
TCCTACCTCCCCCGCCTATAAGCTTTACTACGAAAAAGCCACAGTGATAATTTTTACACACA
GAGTAGAGCAGAGAGAGAGAGAGAGAGAGAGAGATGGGTGATTCTGACGTCGGTGATCGTCT
TCCCCCTCCATCTTCTTCCGACGAACTCTCGAGCTTTCTCCGACAGATTCTTTCCCGTACTCC

TACAGCTCAACCTTCTTCACCACCGAAGAGTACTAATGTTTCCTCCGCTGAGACCTTCTTCC
CTTCCGTTTCCGGCGGAGCTGTTTCTTCCGTCGGTTATGGAGTCTCTGAAACTGGCCAAGAC
AAATATGCTTTCGAACACAAGGTATAAACTTAACTATTCTTAGCTGCAGAGATGCTTCACTT
GGCTTTCCTTGTAAAAGAAAACAAAAACCAAAATTAGTCTCTTTTCTTTTTGGAATGGCTAA
ACACTAAAGAGAAGTGGAGCTAAACAGAGAAATTCGTTGAAGAGAAACATTGATGCTCAA
TTCCACAACTTGTCTGAAAAGGTTTTCTCTTTTATCTTCCTTTTAAGATTCTTAATTTAGAAA
GAAGAAGAACCTTGAGATTGTAGTTGATTAGAATCTGAGTGTTAGCAGAAGAGGAGGAGC
AAGATCAACGAGAAAATGAAAGCTTTGCAGAAACTCATTCCCAATTCCAACAAGGTTAATC
AATCTTTGTTCGAATCAGAGATAGTGAGAAACATTGTTCTGATTGATCCGTTATCTTTTGTTT
GTTTATAGACTGATAAAGCCTCAATGCTTGATGAAGCTATAGAATATCTGAAGCAGCTTCAA
CTTCAAGTCCAGGTTTTTTTCCTACTTACTATGATTATATACGTTCAAAGTCTGATTTGTAAAT
TACATCACTCAGATCATTAACTTGATTTACTGCATGATGCAGACTTTAGCCGTTATGAATGGT
TTAGGCTTAAACCCTATGCGATTACCACAGGTTCCACCTCCAACTCATACAAGGATCAATGA
GACCTTAGAGCAAGACCTGAACCTAGAGACTCTTCTCGCTGCTCCTCACTCGCTGGAACCA
GCTAAAACAAGTCAAGGAATGTGCTTTTCCACAGCCACTCTGCTTTGAAGATAACATTCAG
ACAATGATGATGATCGGAATTCCTCTAGTACCTGCCAGACAGGAGTGAACAATGTTTTGAGT
TTTAGCATTGGCCAGATTTCTATGTTCAGTTATAGTTATGCTAATAAGCTTTAGGAGTGAACA
AAATCTGAGTAGTTTGATTATAATGATGTCTGAAGCAGATTATATATAAAAGACTAATTTACTT
ACATATGAGATGATTATTACAACT

>AtbHLH074
TCTCTTAAATGGTAATTATCAGCTTTATTTGTTCTCTTTCTCTAGGTCTTTTATTGCCCATTAAA
CTAAACACAATTATAATTACTGTATCCTTTGATTCTATAAAGTTGGGAACTTGATGCTTGCTTT
GTTGTTTGTTCAAGTAACCCAACTTGGTGAATCTCTCGTTTTTTTTCCTCATTTTTATTTGATT
CCTTTTTAAAAATTCTACATTCACCCCAATTCAAGCAAAAACAGCATTTGATACTCTTTGAAT
TGAGTGTACTATCTCTTCCCTCATTTATCTGATTTAGGTTTAACAGTGATTGTAAACTCCCCA
CCCAAGAATTTGATTCTTCTTCTTTTGTACTCTCTCTTTCCAAAGCAACCACAATCTTGGAGT
ATCTCTAACAGGGCTTGTCTCCATTACCTTAATTCCCAAAAATTCTTGCTTCTCTGATCTCAC
ACACACATTCATATGTGAAAGGTCTAATCTTTTTTTTTTTGTTTCTTGAAAAACTGAAGAGAT
TGGTTTATGAGATAGTGACTTTGCTCTCTCACACTCTTAAAAGCGAATCTACTTTTTTTTTTC
TCTCTTTTCCTTTTTATTTTTCTCTTGTGAGCTGTAACTTAGCTAAATCCATTGCTTGTTACCA
CTTTATGCTCTGAAAGTTCTCAGCTTTCTTTGTCTATGATAAGTCTATTTGTTCTCTTTAATTAT
TTTTCTTCGCTTGACTTGAGTACGAATAAGCATTTATCCTAGGCTTCTCTCTCTCTCTCTCTTT
CCTAAAAGAACCTGTGACTTGCTTCTGCTACATTAGATTTGATGCTCTGTTGATTTAGTTTTG
TTTTCTCTTTCCTCTTTCTCCACTGTATAAAATCCTGCTAAACTAGTTTACTTCAGTTGTTCCT
TGTCAAAGTAGCAAAACCAGGTTCTGAAATTGGAAAACTTTTTTTGTCCTGAGGGTTCCTG
GTTGTTGTTGAAGCAGAGGAATGGGTGGTGAGAGTAATGAAGGAGGAGAGATGGGCTTTA
AGCATGGAGATGATGAGAGTGGTGGGATCTCTAGAGTTGGAATTACATCAATGCCCTTGTAT
GCAAAGGCAGATCCTTTCTTCTCTTCTGCAGATTGGGATCCAGTTGTCAATGCTGCTGCTGC
TGGCTTCTCCAGCTCTCATTACCATCCTTCCATGGCGATGGATAATCCAGGGATGAGTTGCTT
CTCTCATTACCAACCCGGTTCTGTTTCCGGTTTTGCAGCAGACATGCCTGCTAGTCTTCTTCC
GTTTGGTGATTGTGGTGGTGGTCAAATTGGTCATTTTCTTGGTTCAGACAAGAAAGGGGAA
AGATTGATCAGAGCTGGAGAATCATCTCATGAGGATCATCATCAGGTTTCAGATGATGCTGT
TCTTGGTGCTTCCCCAGTTGGGAAAAGAAGGCTACCTGAAGCCGAATCACAATGGAACAA

GGTATAAATAAACTTCTTGAACTGGATTTGGTAGAATTAGGAAAGACTAAAGCTTTTGATGG
TTTAGGGCTGAAATAATTGAGTGTTGTTAAAATTGGCAGAAAGCTGTGGAGGAATTTCAAG
AAGACCCTCAAAGGGGAAATGATCAGAGCCAGAAGAAGCATAAAAATGATCAGAGTAAAG
AGACGGTGAACAAGGAGAGCTCACAAAGTGAAGAAGCACCGAAAGAAAACTACATTCATA
TGAGGGCAAGAAGAGGTCAAGCCACTAATAGTCACAGTCTTGCAGAGCGGGTAAGGACTC
TTTTACCTGTATATAGATTATGAGAATCTGCTCTTTGTTTTCTGCCAATTACTGGTGCTGAGAA
GTTTGCTATTTCTTTTGTTGTTTTGAAGGTTAGAAGAGAAAAGATCAGTGAAAGGATGAGAT
TGCTTCAAGAACTTGTTCCCGGATGCAACAAGGTATTGGACTTAGAGGTTACTTTTAGCAGT
ATGGTTTCCAACGAAATTTTGCTCAAGTTTTCATTGTTGGTAATTGTTCAACTAGATCACCGG
AAAAGCGGTTATGCTCGATGAAATAATCAACTATGTTCAGTCATTGCAACAGCAAGTTGAGG
TAAGATTATCCCAGTAATGCGAGATTCTTGAAACTCTCAAGTTAGTTTGGTGTGAAAGAGGT
TTTCTTTCTTGAAAAACTGCTTTTATTTTCTTCAGTTTTTGTCTATGAAACTTGCGACGGTGA
ATCCAGAGATCAATATTGATATAGACAGGATTCTCGCCAAAGATGTAAGAGCTCAAAATCCC
CCATAACTTCACATTGAAATAATAGTTTGCAAGTTTTGAATAAGAGTACTAAACTTGCCATTT
TCTCCTTTTAGCTTCTGCAGTCAAGAGACAGAAACACTCCTACACTCGGGCTGAATCCTTTT
GCCGGTTTTCAAGGGAACATACCAAACCTTTCTGCCACCACAAATCCACAATACAACCCAC
TACCTCAGGTAAGCAACCGCTTTGAAGAAATTAAACAACATATTATGTTCAATGTTTTGGTAT
TGCTGTAAATAGTCTTTCTTGTCTCATCTTGCAGACAACACTAGAGAGTGAACTACAAAACC
TTTACCAGATGGGATTCGTCTCAAATCCATCGACTATGTCCAGTTTCTCACCTAATGGTAACT
AAAAGCTACACAAGCTTGCTTCTCTCATATATCTATCTGTCGTTTCCTTTGAATTGTCTTTAA
AACATCTTCTTCTTATCTTAGGTCGATTGAAACCTGAGCTCTAGTACTTCAAGGACACGGAA
AATTTGCAGGGAGTTTGTATAGAAGATGGCTTTGGTTCTAAGTATCATATGTGTTCGTTCTTC
CATATTATTTGTTCTAAGCACAAGGATTTGGATTCTCAGTTGTTGTTGTTTTTGCTTTTCCTCA
ACGAAGAATACATTTATTATATATGGAAATCAGATTTATCTAAA

>AtbHLH075
ATAACTGTCTTAGAGAAAGAAAAAAAACAAAACTAGCTCACAAAAAGGAAATCATATTTTG
ATTTAATTTGTAGTGTCTCTAATGGCACGGTTTGAGCCATATAACTATAATAATGGTCATGATC
CTTTCTTTGCACACATTAACCAAAATCCAGAGCTAATAAATCTGGACTTACCAGCTTCTACC
CCTTCCAGTTTCATGCTTTTCTCCAATGGAGCTTTAGTTGATGCCAATCACAATAATTCTCAC
TTCTTCCCAAATTTATTGCACGGTGAAAATTTTATAATTTTATTGTTTGTGGGAAAAACTGTTA
TATCTATGTTTATGTGTGTTCTGATGAAGCTAGAAATCGCAGGTAATACGAGAAGAAAAGGA
AATAAAGAAGAGAGTGGGTCGAAGAGAAGAAGAAAGAGGTCGGAAGAGGAAGAAGCCAT
GAATGGAGATGAGACTCAGAAGCCAAAAGATGTTGTTCATGTCCGAGCTAAGAGAGGTCA
AGCTACTGATAGCCATAGTTTGGCTGAAAGGGTAATTCTACAAATACATATATACTCAAAAGT
ACAAAAAACTTCACTTTTCTTAGTTGTGTTGGATTCTTTGTGGAATGCATAAAGGTACGAAG
AGAGAAGATCAATGAAAGGCTGAAATGCTTACAAGACCTTGTTCCAGGATGCTACAAGGTA
CTTGAATTAACGCAAACCCTAATATTTCTTTTTGTTTGTTTGTTGTCTACAACTCCCTAATTTT
TGTTTTGTAATTAAAATAAAATCAACAAAACTCTAAAAACAAGTAACTCTAGATAAATTAGT
TACCTCTAAATCCAAGTAACTCTGGTGCTATTTTTTGGTTTAAAAACACTTTTTAAAGATAAT
TTTTCAAGCTCACAATTCTTTAACTATTAATATTTTTTTTCTATGTATCATTGAATTAAAGTGTA
AAAAATTAGGAATCATGTGATAGAATATTTTAAAACTATAGAAATAATTAGTTTTTGTTTTTGT
TTTTTCGGAACGCCAAAATCTAAAAGGGTGTGGCAGCGTCTCTTACGTAAATGAACAGATT
GTTTTTTTGTATAGCGATTTTTTTTTTCCACAGTAATAAATCAAAATGGGTTTATTCTCTATAAT

GATAATTCAAAACGTGATGCAGGCAATGGGAATGGCAGTGATGCTTGATGTCATCATAGATT
ATGTACGATCACTCCAGAATCAAATCGAGGTTAGTTAGGCCATTGATTTCACTCTTCAAAGT
TCAAACATCCCATTAACTTTTATAATATCCCACTAATACTTAAATGTTTTCCTGCTTTGGCCTC
TTGACTACAGTTTTTGTCCATGAAACTCTCAGCGGCAAGTGCATGTTACGACCTTAATTCTT
TGGATATTGAGCCAACGGATATATTTCAGGTAACTATCACATAATATTACATGATTAAAATTAT
AATAAGATAAAACATTTTCATGGATTTGTATTAACAAAAAAATGATTACAGGGAGGGAATAT
TCATAGTGCAGCAGAGATGGAAAGGATTTTAAGAGAAAGCGTTGGAACACAGCCTCCTAAT
TTCAGTTCAACATTACCCTTTTGATCATAAGAAAATTATGAATTTTCAGAGAAATTATTCTCTT
TTTCTATAATTAAACTCCATAAATAAGGACTTACCATGATCAGTATATATAGGTTTATCTATCTT
TTGTGTGTGACGTCAGTATACTTTTATCATAAATGTGTAACCTTATGATTATGAAGCTTATCCA
TATAGTATGTACCATGAAAATGAGTAAAGCTATATGTTACAAAGAAACTCTATTTGAAGTAAC
ATAAGATTTCGATATT

>AtbHLH076
TAAACTCGTTTTTAACTCTCCATCTCTAAAAAGACCATCAACGCTCTTCTCTTTATTGCAGCA
GAAACAGAACCCAGTTCAATAAAGCTTTTTGGTAAGAAAATTTGAAAGGAAAAGATATTTG
AAAACTCGTAGAGAGAAAGTGGCACAGAATCTGAAATTCTGACACTTTTTACCACGAATAA
TGATTCTCTTTTGAAAAACATGACTCAATGTTTTCTTTATCTTTTTCTCTGAATTAAGGTTTGT
TTGTACTTAAAGTATGCTCCTTTATCTACCGTTGACTGATTCTACTCTTTTATTCTCAACTTTT
CTCTGATTTGCTCGTGTGTTTACTTCAATTCCTCAGTTCTTGTGTAGAAAGTTGAGTTTCATG
TGATCAAAATTGGGGCTTTACTACTTTTAAGAGAATGAGTGACAAAGACGAGTTTGCCGCA
AAGAAGAAGGATTTGGTCAATACGCCAGTGGATTTGTATCCTCCGGAGAATCCAATGTTGG
GTCCTTCTCCGATGATGGATTCATTCAGAGAAACTCTTTGGCATGATGGTGGTTTCAATGTCC
ACACAGATGCAGACACTTCCTTTAGAGGTAATAATAATATTGATATACCTCTTGAAATGGGTT
GGAATATGGCTCAGTTCCCTGCAGATTCAGGATTCATTGAGCGTGCTGCAAAGTTTTCTTTT
TTTGGATGTGGTGAAATGATGATGAACCAACAACAATCATCTCTTGGAGTTCCAGATTCAAC
TGGCTTGTTTCTTCAAGATACACAGATTCCTAGTGGATCCAAACTAGATAATGGTCCTCTTAC
TGATGCATCTAAGTTAGTGAAAGAGAGATCGATTAATAATGTATCAGAGGATTCTCAATCTAG
TGGAGGTAATGGTCATGATGATGCTAAGTGTGGGCAAACATCTTCCAAGGGGTTTAGTAGTA
AGAAGAGGAAAAGAATTGGGAAGGTATAGAGATTTGTAGCACTCCTTTTGTATTTTGTGTTT
TTTGGTCTTTCGTTGATAATATTTCTGTTGGTAGGATTGTGAAGAAGAAGAAGATAAAAAGC
AAAAGGATGAGCAAAGTCCAACTTCAAATGCGAACAAGACAAACAGTGAGAAGCAACCTT
CTGATTCTTTAAAGGATGGGTATATTCACATGAGGGCACGAAGAGGCCAGGCTACTAATAGT
CACAGTCTTGCTGAAAGAGTAGGAACAATATTTTTTTGAAGTTGAATAGAAAGCAAATTTCT
TTTGTTGTTGTATTTGAATTTGGTTAAACTTGTTATCGTAGGTAAGAAGAGAAAAAATCAGT
GAAAGGATGAAGTTCTTGCAAGATCTTGTGCCAGGTTGCGACAAGGTAAGTCCTTTTCTTT
TTAGATGTTGATGTCTACATGTAGAAGAATGGAAGCTGAAGCTGTTTTCTTTTCATATAAATG
GGACATTGCATTTATTGTGTAGGTGACTGGTAAGGCAGTTATGCTCGATGAAATCATTAACTA
TGTGCAATCACTTCAATGCCAAATCGAGGTTTCCCTTTGTTTGCTTTGGGAATTTCCTATTTT
CTTAGTGTCTGTTCTTGAGACTTCATTTAATTAGCAACCTCACTTTGTGTTAGTTACTTCTTC
CATGCATTTCAGTTTTTATCGATGAAACTTTCGGCTGTGAATCCTGTGCTCGATTTTAACCTC
GAAAGCCTCCTTGCAAAAGATGTAACATATGTTGAAACCCTTCTCTTTCTTATCATTTGACG
AGTAACTTCATTGATATGATGTTGATGTCTTCTATATATTCATGCAGGCTCTTCAATCATCTGC
ACCGACATTTCCCCACAACATGTCGATGCTTTATCCTCCTGTATCGTATCTCTCTCAAACAGG

ATTCATGCAACCGAACATTTCCTCAATGTCGCTATTGAGTGGAGGATTAAAACGGCAGGTAC
ATATATTTGGTTGCGCATTGTACTCCAACAATAAACATATCTTTGCAGAATCATTTCTTTCTCT
TGCATTCATCTAATATTGTTAGTGAGTTTTGAACAGGAAACACATGGATATGAAAGTGATCA
CCACAATCTTGTCCACATGAACCATGAAACCGGCACTGCACCTGATCATGAAGATACAACA
GGTGAAAGTAAAAGCCCTATAAGTTTCATACTTTTGTAACCAGATACTTGAAACTCGCTAAA
GCTTTTCATGTTTATCTGATCTTACAGCTGACATGAAGGTGGAGCCGTAGATGTTTCTTCTTC
ACTTTGTACTCAGCTTTAGCTGCTGATATAGTGCGCCGCGGTCGAGAATTTTATTAACTTCGA
ACAGAAGAGTGAAAACGAAACAGAGGAGGAAGCAGATTTTGTCTTTGAAATATTGACAGC
TCCCGGAGAAAGAGTCGAGAGTAAGAATCAACAATGTTGTGTAATGAATAGCTACTGTCTG
CTTCTTTTGTAGCTATTGTCTGCTTCTTTTTTGTACATATAAATCCATGCAAGGGAAGAGGAA
TGTTAATTCAACACATGTGCACAACATGATCATCACATATGTACTTCAACTTGATCAATCGGA
ATTAGCTCTACTTGGCA

>AtbHLH077
AGTCTCTTAACCAAGAATAATCAAACATGATAATTAAAAACAATTTTATCCTTTTGTTTGTTT
TCTCTCCCACAACTTTCCTGTCTCTGCCATGCACATGCTCTCTCTCTCTCTCTCTTTCTTCTCT
CTTATTCTCACTCCAATGATTACAGAGGCCAGCCTAAGCAGCCTACCCACTTTCACCATCTAA
TGTTCCCACATTAACATAAAACACACGTAAGATTCTTCTCCTTTTGAACTGTAAAATTCACTC
TTTTTTAATTTCTTAATCTTTCTTATCATTACTTTCTCTTTTTTATTTTGTCATTTTCTCTTAAAC
CTTCTTCTTCTTCTTCCTTTGATCAGTAAAAAAAGTCACCGCCTTCTTCTTCTCTTTCCTCTT
TCCCCTAATTGATCAATCATGCTTCTTCATTTCTAGTTCTCTCTTCCTGACTCATTTCCCTCATT
AGATCTTCTTCTCCACTAAACCCATAATTGAAAGAAGCTCTTTTTTTGCTTACATTGAAGTTA
AAAAATCATCACTTGTTTTGTTTTTGAAGAAACACACATAAAGTTTGTCTTTTTACTTCTCAA
ATTTGGTTGTTTTCACTCAATTTCAATCTTGCTTTCTGATTCAGATTCTGGGTTTTCTTCAGAA
TTCCCAGAGAAGTTGTTGGAATTGAATTTTTCACTTTTTTGTTCATAAAAGTTCTAGTCTTTT
TAGATGAACATGGACAAGGAAACAGAGCAAACCCTAAATTACTTACCTTTGGGTCAGAGCG
ATCCCTTTGGCAATGGCAATGAAGGAACAATTGGAGATTTCCTTGGAAGATACTGCAACAA
CCCTCAGGAGATTTCACCGTTAACTCTACAATCCTTCTCTCTGAATTCTCAGATCTCCGAGAA
TTTCCCAATCTCTGGTGGAATCAGATTCCCTCCATATCCAGGTCAATTTGGATCCGATCGTGA
ATTTGGGTCACAACCAACAACGCAGGAGAGTAACAAGAGCTCTTTGTTGGATCCAGATTCA
GTTTCGGATCGAGTTCACACCACGAAATCTAACTCTAGAAAGAGGAAGTCGATTCCTAGTG
GTAATGGCAAGGAGTCTCCAGCTTCATCGTCTCTTACAGCTTCCAATTCAAAGGTTTTGATT
CATTATCTCTCTTGCTCTATTTCAAAAATGTATACTTTAGGAAACTGTAGCCATATGTGTAAGA
AAGTTTGTGTTTTGTGGAGACTTTTTATCTATAGACCTTTTAGTGTGTGTACAAGGTTTTTA
GTTGTGTTTGGTTTTGGATATATGATTAGGTTTCAGGAGAGAATGGTGGATCTAAAGGTGGG
AAGAGAAGCAAGCAAGATGTAGCTGGGAGTAGTAAAAACGGAGTAGAGAAGTGCGATAGT
AAAGGCGACAATAAGGACGATGCTAAGCCTCCTGAGGCGCCTAAAGATTATATTCATGTCAG
AGCTAGAAGGGGTCAAGCAACTGATAGCCATAGTCTTGCTGAAAGAGTACTACACACCTTC
TAAAACTGATCTGTTTTTTTGTTGATTAGGGGCTGTTTTGATGTTTATGATGTTGATGTTTTTT
TTGGCTGTGTTTTGGTTTCAGGCAAGAAGAGAAAAGATTAGTGAGAGAATGACGTTGCTTC
AGGATCTGGTTCCTGGTTGCAACCGGATTACAGGGAAAGCAGTCATGCTTGATGAAATTATA
AATTATGTGCAGTCCTTGCAGAGACAAGTTGAGGTATGAATTGTGTATTTCATAAATGTTCTT
GAAAGTTGGAGTTTGTATATCTTGAATCTAAAACTCGTGGTTTGTTTCTTTAGTTCTTGTCCA
TGAAGCTGGCTACCGTAAATCCAAGGATGGAGTTTAATGCTAATGCTTCTTTATCCACAGAG

GTAAGATTCTGCTTTGTATCTATAATGGAATGGTCAGATTATGGAATGTTTTGTATCTATAATG
TGTGTATTTGTATGTGTAGATGATTCAACCGGGGGAGTCGTTAACGCAGTCTCTTTACGCAAT
GGCTTGCTCAGAGCAAAGACTTCCATCAGCATACTATTCACTTGGCAAGAACATGCCAAGA
TTCTCAGACACACAATTCCCCTCAAACGATGGATTTGTTCACACTGAGGTAGTTTTAGTAGT
AGCTAACGTCAGATTCTAGAAAAACTCGTTTGTGCGTATCGTAAACTCGATCCTTTTATGTTT
TTGATTCATTATAGACACCAGGATTCTGGGAAAATAATGACCTGCAAAGCATTGTTCAGATG
GGTTTTGGAGATATCCTCCAGCAACAGAGCAACAACAACAACAACAACTGTAAGAAAAATT
AGAAGCAAATTCGTCTATCGAAATTATAGAACTATCTCTTATGTTGTTAAAGTCTCGTATCTTT
ATGTTGCAGGTTCTGAGCCAACGCTTCAGATGAAGCTTGAACCATAGTCCATAGCTATGATT
ATTATCTCTGTTGTACATACAGAAAACCAAGTAGTGATGAGTAACGGAGTGAATCATATTGG
AATTTTAATGTTTACAATCGTTGCTTCTTTGCTTGTGTCTCTGTTGATTTCTTCTCAACCTTAA
ACAAAAAAAAAACAAGAAAACTTCAGATTAAGTATGTAAAAATATGACAAGAACTTTTCTC
TGTACTGTTTTACAATCGTTATGTATTATGATTGCTCTAAGCTTGTAAGAAACAGGAATAAAT
TTGTTCACTTCTTTATATTCTTTTCACATTTTCTTACGAAACAAGATTGAGAGTACGAG

>AtbHLH078
GCAAATATCTCTCAACGTCAAAGGCCCGGCAAACAATTCCTAATCTCCGCCCGTTACGGTGC
AACACACAAAATGATAATTGAATTACTATTTTTTTATAACTATATTACGTTAAATGATTTATTAA
TATAAAAAAACGTAATTTAATGTTCCATTAAACAACTATATAAACCAACTTAGCGACCACTTA
AGCATTTCCCTTACTATTTACGTTCTCTTCTTTCTCCTCCGACACACTCCAAAACTCATAAAT
CTATCTGATGCGAATTTGACTAACTCTTTCGTAATTCAGGATCTTACTACAAATCTTTAACTC
ACCAATCTTCTTCTTATTCAAAGATTAGAGAGAAAATGGACAACGAGCTGTTTATGAACACA
GAGTTTCCACCACCGCCGGAGATGGCGACGCATTTCGAACACCAACAGTCTTCTTCATCGG
CCATGATGCTTAATTGGGCTTTAATGGATCCAAATCCGCATCAAGATTCTTCCTTTTTATGGG
AAAAGTCAACGGAACAACAACAACAACAAAGCATCTTTGACTCTGCTTTAAGCTCATTAGT
CTCATCACCGACGCCGTCAAATTCCAACTTCTCCGGCGGTGGCGGTGACGGTTTTCTCATCA
GAGAACTCATCGGAAAGCTTGGAAACATCGGTAATAATAATAACAACTCCGGTGAGATCTA
CGGAACTCCGATGTCTCGCTCCGCCTCATGTTACGCAACTCCGATGAGCTCTCCACCGCCAC
CGACGAATTCGAATTCTCAGATGATGATGAACAGAACGACGCCGTTGACGGAATTCTCAGC
AGATCCGGGTTTTGCGGAGAGAGCAGCTAGATTCTCTTGTTTTGGTAGTCGGAGCTTTAACG
GAAGAACCAATACAAATCTTCCGATTAACAACGGTAATAACATGGTCAACAACTCCGGGAA
GCTGACACGTGTCTCCAGCACACCAGCTCTTAAGGCTCTTGTTTCACCGGAAGTCACACCC
GGCGGCGAATTTTCCCGGAAGAGAAAATCTGTGCCTAAAGGAAAATCCAAAGAAAACCCC
ATTTCTACAGCTTCTCCATCTCCTAGTTTCTCAAAGGTACACAAAATTTTCCCGGAAAGTTAT
AATTTTTTCGTCAACTTTTCAAAATTTGGAAGACCCGCTTTTTTTCTAGCGTCACTGTTGGA
AGAACCCAGTTCTTTGAAAAGTCAGCTTTTATATAAAGTGATTAACTTTTTGTATTTTTAATTT
CTTAGACGGCGGAAAAGAATGGTGGAAAAGGAGGAAGTAAAAGTTCAGAAGAAAAAGGA
GGAAAAAGGAGAAGAGAAGAAGAAGATGATGAAGAAGAAGAAGGAGAAGGTGAAGGGA
ACAAAAGCAATAACACAAAACCACCTGAGCCTCCTAAAGATTACATTCATGTTCGAGCTCG
ACGAGGCCAAGCAACCGATAGTCACAGCCTCGCCGAACGAGTAATTATTTCTCTTTACTCTC
TACTTCTCCTAAAATCTTATATAGTAAAGTTGACCTCAATTATCCGGAGTTTGTTAAACTCCG
GATAAAATTGGCAAAATATTCTTTAGATTCGGTTAAATTTTAAACCAAAATTTCGAGAGAGT
CGGGCTAATGTATGAGTTGGAACACTAGTTGCGAGGCAAAATAAATGCTAAGAAATTTATAG
GTTTTGGCACAAAGAAATGTATAGGTTTTGGCCCAAAACTTATCGGATATGAAGATAAAACA

AAATATAATCACATTTAGTGAATTCTTGATGACATTGCAAGTTCAATGATCTAGTTTAAATTTT
GATCAGTACTGTTTTTTTTAGTAATAAGGGGATAAATTAAACCTTTATTAGTTGTGTTAATTGA
ATTTGGGTTTGATTTTGTTCTTTAATGTTGGTTTAGGTTCGGAGGGAGAAAATTGGTGAAAG
GATGAAGCTTCTTCAAGATCTTGTGCCTGGATGCAATAAGGTGAGACTTTTATTTTTATACTT
TTAAGTACATGATTATCACTGATTATTTTTCGTTATGAACTAAATTGTTGATTAGTATAATTAGA
AAATTAATGTCTTTGTTTTTCTTTGTTTATACGATGAAAGGTTACTGGAAAAGCACTGATGCT
TGATGAAATTATAAACTACGTACAATCATTGCAAAGACAAGTTGAGGTATAATTATTATTTGG
TTTCATCTAAAATCTAATTAATTGTTTCATTCATTAATCACCGAATTTAAATTATTTGCAATGAT
TTTTTTAATCTATTTTTCTAATATTTTGTGTTTAATAATCATGGGCAGTTCTTGTCAATGAAGTT
ATCATCAGTGAACGACACCAGGCTGGATTTTAACGTGGACGCTCTTGTGTCAAAGGATGTT
GTAAGTCACCTTAAACATTAACATCAATTTAACAACCATTAATAATGATAATTTGTAATTATTC
ATTTCTCAAATCCTTGTTTTTTTTTTGCTTTTTGTTTTTGTCGTCACTTTTATATATATATATATAT
ATATATCTTAAATACTTTTTTCAATCCACCCATTAGCATTAATCAGGCAGCTTTTTATTTTCTTT
TATAAAGTACCTTTTGTACCTTTTCTATTGAATAATATGCAATCTTTTTTTTTTAAATGTGTTTT
GTGTAATTTGATTTTTTTTTTCAGATGATTCCATCAAGTAACAACCGATTGCATGAAGAAGGA
CTCCAATCAAAGTCTTCAAGTCATCATCATCAACAACAACTTAATATTTATAACAATAATTCA
CAATTACTTCCCAATATTTCTTCCAATAACATGATGCTCCAGTCTCCTATGAACTCTTTGGAA
ACCTCTACCTTAGCCAGAAGCTTCACTCACTTACCAACACTTACCCAATTTACTGACTCAAT
TTCTCAGGTTTAAATTACTCTTTTAATTTTCTTCTTCAATCTCAAGTTTTATATAACAAGTTTTT
TTTTTTCTTTTGTGTTTTCAGTATCAAATGTTTAGCGAAGAAGATTTACAAAGCATAGTAGGA
ATGGGAGTGGCAGAAAACCCCAACAATGAATCTCAACACATGAAAATTGAGCTTTGATCAA
CCATTTTTTTCAAGGTCTCAAGGAAGAAAACAAGAGAAAAAGGATTGATGGGTGTACATAA
ACCATACCAATATTTGTGATTTATTCATATACTCATCTCCATATATATATGTATACACACTTTTAT
GGAAATAACCTATATTTTTTTACTTTTTATACTTTATATTATCATCTTCCAATTCATTATTAGCAT
TATTAGGGTTCAATTGTATTGTTATCCACTTTGGATTCCTTTTGCTTTTCTGATGTAATATCAAT
GTAAGTTTCTTACCATCCACTACAATCCAAGATAACTAATCTACTCTAAATTAG

>AtbHLH079
CCATTGTAATATTTTTTTTATAACAGAAACGGCAAAATATTTGATGTAAGCTGTGTAATTAATG
AGATTTTCTGAAAGTATTGGTGATAAATCATCTTCCATTGCATACTAGTGGCGCATTATCACT
ACCCACATCTGTCTTACCTTCTCCGATCTCTCTCTCTATTTGTCCACCTCACACACAACCCTA
TAAACAAGACAAGACCTCGCAAAGCTCAAACCTTTTCCTTCTTTTTTTCTATCAAGTTCCTC
ACTTCTCGGATCCGGGTCCTAAATCTGGGTCAACCCTTTTTACGATCATCGGAAATGGACCC
TCCACTAGTGAACGATTCCTCCTTCTCTGCAGCTAATCCTTCTTCTTACACTCTCTCCGAGAT
TTGGCCTTTCCCTGTAAACGACGCCGTTCGCTCTGGTCTCCGTTTAGCTGTTAACTCCGGTC
GAGTCTTTACTCGCTCTGAACATTCCGGCAATAAAGACGTCTCCGCGGCTGAGGAATCTAC
AGTCACCGATCTAACTGCTGGCTGGGGAAGTAGAAAGACTAGAGATTTGAACTCTGAGGAT
GATTCTTCAAAGATGGTTTCTTCCAGCAGCAGTGGTAATGAATTGGTGAGATTTTGCACTTC
AATTTTTGAAAAGTCCTGATTTTTATTTGTTGGGTCTTTTGAATGTTTGAATCTAGATCCTTTA
GCTTGGTGGTGATCTAAAAATCAATGAGTCAAAGTTTAAGTCTTTGGTTTAGGGATCAAGGA
TTAGTTTATAGATTAGATGACTTATCTCCATTGACTTTTTGACTTAAGTTGGATTCAGCTTGAA
GACCATTAGTTTGATAGATGTTGGAATGGTCAATGCTTACTTAGTGATCTTAGATTGAGAGTT
TGATTTAGGATCTTAAAGCTTTCCATCTGTTTACTTTTTTAATAGAAAGAATCAGGGGATAAG
AAAAGAAAACTGTGTGGATCTGAAAGTGGAAATGGAGATGGTTCGATGAGACCTGAAGGC

GAAACAAGTTCGGGTGGTGGTGGAAGCAAAGCAACGGAACAGAAGAACAAACCTGAGCC
ACCAAAGGATTATATTCATGTGAGAGCAAGAAGAGGACAAGCTACTGACCGGCATAGTTTA
GCAGAGCGAGTAATTCTTCTGCTAAAATCACCATAAATTTTATCCTCAAAAACTGTTCCTTTT
ATTAGAATCACAATGTTGTCTTTGACAGGCTAGAAGAGAAAAGATCAGTGAGAAGATGACA
GCTCTTCAAGATATAATTCCAGGATGTAATAAGGTACATGTTCTTTTGGTCCCTGCTTGTGAA
TGATGTTATTATAGCCTAAAAAACATTCAACAAGATATTTCTGTTGATCAGATAATCGGAAAA
GCCCTTGTGCTCGATGAGATTATCAATTATATTCAGTCATTGCAGCGGCAAGTTGAGGTAAA
AGTCGTTAACACCTCTCTGTTATATGTTACAATTATACATGCCTAAGCATGGAGTTTGGTCTG
CAGTTTCTATCAATGAAGCTAGAAGTTGTTAACTCGGGTGCAAGTACTGGTCCGACAATAGG
AGTTTTTCCTTCCGGTGATGTAAGTCCCAAATCTAAAAGAACATAGAAAAATCTTATCTTTCT
TAATCAAAGCTACTAAATCTAAAAGCTCTTGGTGTGTTGTATCAGCTCGGGACTTTACCGAT
TGACGTTCATCGAACAATATACGAGCAACAAGAAGCTAATGAAACCCGAGTATCTCAACCG
GAGTGGCTCCATATGCAGGTTGATGGGAACTTTAACCGAACCACATAAAAAAGAGTCTTTC
TCTGATTGATGGATGTGTTTTGTAGGCAGTAAAAATGAATGCAACCCTTTATTTTTTATCCTAT
GCGTGTGTATCATTTTGCTGTTATATCATAAGATAGAATATGAGAGTGACCAGAGAGATTAAC
ACTTTATAAAACATTGTTGTATTGTCCTATTATATAACTTGTAACAATTGATTCAGTAACATAT
GTTGGTCTTAAATCTTGTCCTTTGTATAATCTTTATGTTATGATGAATCAATGATACGTTTTTTT
TGTTGTC

>AtbHLH080
AAAAAACAGCTTCAGCCAGAAAAAAGGTCACATTTTTTCATTTTTGGAGGTGGTCTCTCCT
TTTAGCCTCCGAACGTATACCTCACTCGCTACAGTTGCGCGTAGACGAATAATAAATAGAAA
CCATATCCACTGAAAAATAAAGAACAAACAGTGACGTAAAAGCTAGAATTCAATGTAATGC
ACCGCACGAAACCTCTCACTTATCCACCGTTAGATCTTAGTCAAAAGTCAACAAAACACAA
ATCCAACGATTCTGTTTTTGTATCCGTGTAAATTAATCACACGGTAGTTTTTGATGAAAAGAC
AACAATCGGAGAACAATCTGGTCTGCTGCTAAAATTTAATAAATTGTTTTGTCTAATTGTCTC
CACCCATAAAAAAGCGCGAATTCAATTCACCGACTAAAGACATTCTCCGGTGGAGACCCCG
ATGCAATCCACTCATATAAGCGGCGGAAGTAGCGGTGGTGGTGGTGGAGGAGGAGGAGAG
GTGAGTCGAAGTGGATTATCTCGGATCCGTTCAGCTCCAGCTACTTGGATTGAAACCCTACT
CGAAGAAGATGAAGAAGAAGGTTTAAAACCTAACCTTTGTTTAACAGAGCTGCTTACTGGT
AATAATAACTCTGGAGGAGTGATAACGAGTCGTGACGACTCGTTCGAGTTCCTGAGTTCTG
TTGAGCAAGGATTGTATAATCATCATCAAGGTGGTGGCTTTCACCGTCAGAATAGTTCTCCG
GCTGATTTTCTTAGTGGGTCTGGTTCTGGGACTGATGGGTATTTCTCTAATTTTGGTATTCCG
GCGAATTATGACTATTTGTCGACCAACGTTGATATTTCTCCGACTAAACGGTCTAGAGATATG
GAAACACAGTTTTCTTCTCAGCTGGTAAGACTCTGTTTCCATTAAATTCAGATAAAAATGCG
AACTTTAATGGAAAGATAAACTTTTTTTTGTTGCTTACTTTAATGTCAAGTTGTGTGAATTTG
TGGTCAAAATGGTGAGGTTAGGTTCTATTTTGGGGATTGATACGGTTTTAGTTTAATTGGGTG
TTTGAATTCTTCAGTTGTGGAGACAATAAAGATGTAAAAGAGTTGTCTTGTCTAATTTGACA
ACTTGCATTTGTTTGAAACAAATTCCCCTGCAAGTTGCAGTTGTTTTTTCTTTGTTTCCCTGT
GGCTGTGAAAGTGAATGTTGCATAAAGTTTGTATTAGTAGATGGGCAAACAAAAAGAGCCT
TCATTGGTTAGATATGTATCGATCTTGTAACTTTAGAAGTTTCTGAATCATGACTTTTGTGAAA
TGAAACATGTTTTCATGTGTGCATATGGTTTGTGAGTTAACTAATAACTCTTCCCTTTTGGTT
TGTGAGTAGAAAGAAGAGCAAATGAGTGGTGGGATATCAGGAATGATGGATATGAACATGG
ACAAGATTTTTGAGGATTCAGTTCCTTGTAGGGTTCGTGCTAAACGTGGTTGTGCTACTCAT

CCTCGTAGCATTGCTGAACGGGTAATTACTTAAAAGTTTGTGCTAGATGCTTTGCAGTACTAA
TTCTGTTTCTGCCTCATTCTTGTTTTGTCATTAAGCTTTTCTCTGTTCATTTTGTTAGGTGAGA
AGAACGCGAATAAGTGATCGGATTAGGAGGCTGCAAGAGCTTGTTCCTAACATGGATAAGG
TAAATTCTTTTTACAAGGCAGATTGGTTTTAAGTTTTCTTTGAAGTTTTTAAAAAGAATTACC
AAATGGGTCCAAGATTAGATATGCTTAAAGCTTAAGGTTAGAGCATTATTGTGAAGGCATTAT
TGGTTCTTAAGGTAGGAGCTAGTGCTTAGCCGTAAAGTAAAGGAATTTGGATGTTAACTGTT
GATGATTGTGAAGGCATTATTGGAACTTCAAAACTACTTACTCCTTAATCCATAAACTATGGT
TGTGCAGTCTTTTAGAAGATTCAATGCATGATAGTCCGGGAGAGTTATAACGCGAAGATTCG
TTTTTTTTTGTATTTTCTTTGTTGCAGCAAACCAACACTGCAGACATGTTGGAAGAAGCTGT
GGAGTATGTGAAGGCTCTTCAAAGCCAGATCCAGGTTTATCTACAGATACTAATTGATTAGA
AACACAAATTTATGTTTAATAAAATATGTTAGGATTTTGGATTTTTTTGATCATTTTATTTGGTT
TTGATGATGGATTCAGGAATTGACAGAGCAGCAGAAGAGATGCAAATGCAAACCTAAAGA
AGAACAATAATGTATCCTTTAGGATTTGATATATCTGTATTTTATTTTTGTACTATCTAAAAATG
GTGATGATCTGTTCGAAAATTCGAAACATGATCTTATATATTGAACTAGAAAAAATAGATATA
TATGAATTTTAGCTGTAAAATTTTTGTACAATAAGGAGAAAAAGATTTAGAAGAGTCAATAA
AAAGATGATGTTTACAAGTCGAGAATATATATTTTGTTAGTTTTGTTTATCAATTTATGCTCAA
GGTTTCAG

>AtbHLH081
GGGTTCTTTATTATTACTAGCCGCAATTTTTTTATTGAAACAAATAAATAAAAAAATAACCCA
AGAACAACAACAATCTGGTCTGCTGATGTATTAATTAAAGAAAAAAATAAAACAGAGCAAG
AAACAAAAAAAAAAATGTAAAGAAAGAGAAAAAAAGCTTTCGTAGTGTCTATTGAAACCA
GAGAAAAGCCAAAGGGGATGCAACCAACATCCGTCGGTAGTAGCGGCGGTGGTGACGACG
GAGGAGGCAGAGGAGGAGGAGGAGGGCTAAGTAGAAGTGGACTATCTCGGATCCGTTCAG
CTCCAGCGACTTGGCTTGAAGCTTTACTTGAGGAAGATGAAGAAGAGTCTTTGAAACCTAA
TCTTGGTCTCACCGATTTGCTTACCGGGAACTCGAACGATTTACCGACAAGTCGCGGCTCGT
TCGAGTTCCCGATTCCTGTTGAGCAAGGGTTGTATCAACAAGGTGGGTTTCACCGACAGAA
TAGTACTCCGGCGGATTTTCTTAGTGGTTCTGATGGATTTATCCAAAGCTTTGGGATTCAGGC
GAATTACGATTACTTATCGGGGAATATCGATGTTTCTCCGGGAAGTAAGCGGTCTAGAGAAA
TGGAAGCACTCTTCTCTTCTCCTGAGTTTACTTCTCAAATGGTAAACCAAAATTAGTTCTTA
AATTAGGGTTTTCTTATACTTGCCAAAAATAGGATGAACTTAACTGACTTATTACTTGTTTTA
GTTAATTTGCTGACCAAGTTCTCGAGATTGAAAAGACCAGTCTTGTCTTAAAATTGACAACT
TGTGTTTTTCTGAACCAAACTAAGCAAGTTGCAGTTTCTATTTTAACTCAAAAAAAAAAAA
ATGGCAGTTTTTGTTGTTGGCTCTGTGGCTTTAATGTGTCAATGTGAACATTAAAGAATGCTC
CTATGGGTAAAGTGTTTAGAAGACCATAAGTCCACATGATGATACATACTTCGGTGGTTAATG
TGTACAAATGTATTGATTCTCCACACCTTTTGGTACATTGGTCTACTTAGTATTAAGTAACTAC
ACTTGATCAAAATTTTGGGAACATATGTGTTACCTATGTGTTGATTAAGGTATTAGTGCTGAT
TTGCGCTTTTTGGAATTTTTTTCTGTAAGTAGAAAGGAGAGCAAAGCAGCGGTCAAGTTCC
TACCGGAGTATCAAGCATGTCGGATATGAACATGGAGAACCTTATGGAGGACTCTGTTGCTT
TTAGGGTTCGGGCTAAACGTGGTTGCGCAACTCATCCCCGCAGCATTGCCGAGAGGGTAAA
CAACATTCAATCTTTATTTTCAATGTTTTGGAAGTGTAGTGATCAAGGTTCTCTTAACTTTATA
CATTGAAGTTTAGTGATACAACTTATATCTTTACTTTCAATTTACGGATGTTTAATGATGCACT
GTATGCTTCATTGAACAGAGGATTGTTCTGTTCTGGTCAGTGTATATTTATCTTTACCTTTCAT
TGTGTAGGTACGAAGGACGCGGATTAGTGATCGGATAAGGAAGCTACAAGAGCTTGTACCT

AACATGGACAAGGTAATATAATTTTCAGCTTAGGTATAATCTGATGACCTCGGTTACACAATT
ATATTGACTAGAAATAGGTCAAAGATTATCTATGCTTAAATCAAGTGTAGAAGTTAGAGGTC
AAATTAAATATGACTGTTAATGGTTGTGATGCAACTTCAGAGACACGGTTCTCAGCTCTGTA
ACTAAGTAATTCACAAACTGTGGAATGGATCTTGATATGGACTTGAATTGTTGTCACCTCGA
TCAAATAAGTATGAACTTGGTTTCCTCTCTATAGATTATGAACAAACTGAGGAAAACTTTTG
GTTTTGGTATATATGGAGATTTGGAATCTAGGCTTTGAGTATATCGGTTAACTTTTTTGTCTTT
GTTGCAGCAAACCAACACTGCAGACATGTTAGAAGAAGCAGTAGAATACGTGAAAGTTCTT
CAAAGGCAGATCCAGGTAAATTATCTAAGTTAATATATATACACACAAAACCTTTTAGAGGA
ACCCAATAGATGAATTAAAAATGATTAGTCAACAGTATATAGTAAAAAGAGTTATGAGTTGG
TTTATTTTGGTGTGGATAATATAGGAGTTAACAGAAGAACAGAAGAGGTGCACATGCATACC
TAAGGAAGAACAATAAGGTTTGCTCCTGATTTGTTTTATATTTGCTTAACGGCAATGATCTGA
TCGAAAAATTCGAAAGATGATCTTAGCTTGAATTTAGATGGATGTCATGTTGAAAAGTATATT
ATTTGATAAATGGATGTAGGTGTAATATAAAATTTTTGTACAATAATGAAGAAAGTTAAAAAG
AATTAATGAAAACATATATTCTTTATGATATTAGTGAAATGACTTTGATTTCATGTTGTTTGATT
ATAGTAAAGTATTTTCAAGATGTGT

>AtbHLH082
TTATGGGAGTAATGAAAGAACAAATGAAACGGCCAGTAAATTAAAAAAATAGAAATCAAAT
TTTTTTGAAAAGAATAAAAACAGGGGAGGGAACAGAGGAAGAAACCAGAAGAAGAGAAG
AAGAGATGGAAAATGGAAATGGAGAAGGAAAAGGAGAATTCATAAACCAAAACAATGACT
TCTTCCTTGATTCCATGTCAATGCTCTCCTCTCTCCCTCCTTGTTGGGATCCATCTCTTCCTCC
TCCTCCTCCTCCGCCACAATCTCTTTTCCACGCTCTAGCCGTCGACGCCCCCTTCCCCGACC
AGTTCCATCATCCTCAGGTACCAAAACTATTATCTTACATCATAACTCACCAAAACTAAGAAT
GTTTCTGATCCTTTTCTGTGTTGTTGTTGTTGTTTGTCAATGCAGGAGTCAGGAGGTCCAAC
AATGGGTAGCCAAGAAGGGTTACAGCCACAAGGTACAGTGTCGACCACGAGCGCACCTGT
TGTTCGTCAAAAGCCAAGAGTTCGAGCCAGGAGAGGCCAAGCCACCGATCCTCACAGTAT
CGCTGAGAGGGTATCTCATTGTTCACTTCCTCCATATCCTTCTTCTCTTATCTTGTGTTTCTGA
TTGTATGGTTTTGTACAGCTTAGAAGGGAACGCATCGCAGAGCGTATGAAGTCTCTCCAAG
AACTGGTTCCCAACACCAACAAGGTATTCACTACTTTCCTAATTTATGAATCTTGTTTGGATT
TGCATAATAACCGAATTGGTTGATGGTGAGATGCAGACGGATAAGGCATCAATGTTGGACGA
GATCATCGAGTATGTTAGGTTCCTTCAGCTTCAAGTCAAAGTACTAAGCATGAGCAGATTGG
GAGGTGCAGGATCAGTTGGTCCACGCCTCAATGGTCTCTCCGCAGAGGTCAGTTTCTTAGT
CCCTTATGGCTTTCAACATATGTATAGTGGCATCACCAATCATCATCAATCGTCATGGTTTTTG
GTAATGTAGGCAGGAGGACGGCTCAATGCCCTCACTGCACCGTGCAATGGCTTAAATGGGA
ATGGAAACGCTACAGGATCGTCCAATGAGAGCCTAAGGTCAACAGAACAAAGAGTGGCGA
AACTGATGGAGGAAGACATGGGATCAGCAATGCAATACCTTCAAGGGAAGGGGCTTTGCTT
AATGCCAATATCTCTCGCAACCGCGATATCATCATCAACTACTCACTCTCGTGGATCCCTCTT
TAACCCCATCTCCAGTGCTGTAGCAGCAGAGGATTCAAATGTAACAGCAACTGCAGTTGCT
GCACCTGAAGCCTCGTCTACTATGGATGATGTATCTGCTTCCAAGGCCTGAACCATAGAATA
CTCATGTGTTTCGTTTTCTCAGTTTAAGTCAAACGCTAATCTCCCTATCTTTTTACTATTTTCT
CTGCTAAGCAACAATAATATAAAACAGTTGCAACAAAGAACAAAACCGACAAGAGATGAA
CATTCGTATACGTGTGTTGGGTCATGGGTGGTGGGACCGGAAATTTAGAGACATGCGTCCCA
CTACCTTCCCAAATCACTTTGTGTCAACGTGTTACGTTACTAGTGCGTGGTGCCTTAACATAT
TTGTTTGCACAATCGTGGTTCGTGGAAACAGTATAATATTCATG

>AtbHLH083
AAACACATTGTCTCTCTTTGCTTACCAATAGACAACCTTAAAGCCACTTTTCGTCAACAAGA
CACACAAAAACCCTAAACAAAAAATCACTTATCAAACTTATTAGTGTCTAATGGCACTCGTT
AATGACCATCCCAACGAGACCAATTACTTGTCAAAACAAAATTCCTCCTCTTCCGAAGATCT
CTCCTCGCCGGGACTGGATCAGCCAGATGCAGCTTATGCCGGTGGAGGAGGAGGAGGAGG
CTCGGCTTCGAGCAGTAGCACGATGAATTCAGATCATCAACAACATCAGGGGTTTGTATTTT
ACCCATCCGGTGAAGATCATCACAACTCTTTGATGGATTTCAACGGATCATCATTTCTTAACT
TTGATCATCACGAGAGCTTTCCTCCTCCAGCCATAAGCTGTGGTGGTAGTAGCGGTGGGGG
CGGCTTCTCCTTCTTGGAGGGCAACAACATGAGCTACGGCTTCACAAACTGGAATCATCAA
CATCATATGGATATTATTAGCCCTAGATCCACCGAAACTCCCCAAGGCCAGAAAGACTGGTT
ATATTCTGATTCAACTGTTGTAACCACTGGTTCTAGAAACGAGTCTCTTTCGCCTAAATCCGC
TGGAAACAAACGTTCTCACACGGTAATTACAAACAACAAAGTCTTGGTGAATATTTTTCATA
TAAGATGTCAATGAAAGATCGTTGATATATACTACTATCAATTTGAATTATGGATATATAGGGA
GAGAGCACTCAACCGTCGAAGAAACTGAGTAGCGGTGTGACCGGAAAGACCAAGCCTAA
GCCAACAACTTCACCTAAAGATCCACAAAGCCTAGCAGCCAAGGTTTGCTTTCTTGACATT
AATTTACATTCATTAAAATAAAACTCAATCTCTCAACACATATTTACATATGTTTTATTTTGTTC
AGAATCGAAGAGAAAGGATAAGTGAACGTCTCAAGATATTGCAAGAACTTGTTCCCAATGG
CACCAAGGTACACATCCATATAACCATTTATGACAGATTTATTATATTACTTATTCTCATGATAA
TAATATTTGTTATGTTTCACAGGTTGATTTGGTGACAATGCTTGAAAAGGCTATTAGTTATGT
CAAGTTCCTTCAAGTACAAGTTAAGGTATATTAGTTATGACAAACATCCCTCAAATTAATTTT
CAGGATTGATTTAATTACCATATTTATCGAATTATTACGTAACACGTATTGATATAATCTATTGT
TTGTTAGGTATTAGCGACCGATGAGTTTTGGCCGGCTCAAGGAGGAAAAGCTCCTGACATT
TCTCAAGTTAAAGACGCCATTGATGCCATTCTCTCCTCATCACAACGAGACAGGAATTCGAA
TCTGATCACCAATTAATGAAGGGTTTTATCATTAATAAATCAGTTTATCAAACATTAATTACGT
ACGTTGTAATAATATCGGGGGAAACAATGATTCTCTCGATTATAAATCCCACGTAAATTTTGA
AATATGATCCAAAGAGAAGACAATCGAGATTATGTAACGTTAGATTATGTGAAAATCGAGAA
GTTCTTAATGCATCTATAACGAAGAGAAGACAAGAGACATTGAATTCACCGATGATCAGTCT
GATATGGGTCCACACGGGGATGGAATCTTTAACTAGTAAATAAGTGTTAAAAACATAAATAT
GTTTTTCAATTTCTTTTTCTGTTTTAATTGTATTTTGTGTGTTTTAGTCTACGGAATACAGATC
TTTTATTTAAACTCCAAGAAATGAGTAATAAAATTCTTCACTTTTGTTTTTTCCTT

>AtbHLH084
ATCATTCCTTCAATACTCTCACATTACTTATCGGATTTTAATTAAGATTAATTAGTCTATTGTTA
AAGTCGCTTAGTGATCAAAAATGGAAGCCATGGGAGAATGGAGCACCGGCCTAGGCGGAAT
ATATACAGAGGAAGCTGACTTTATGAATCAGCTCCTTGCCTCCTATGAGCAACCTTGTGGCG
GTTCATCTTCAGAGACAACCGCCACACTCACGGCCTACCACCACCAGGGTTCTCAATGGAA
TGGTGGCTTTTGCTTCTCTCAGGAGAGCAGTAGTTATAGTGGTTACTGCGCGGCGATGCCAC
GGCAAGAAGAAGATAACAATGGGATGGAGGACGCGACAATCAACACGAACTTGTACCTTG
TTGGTGAAGAGACAAGTGAATGTGATGCGACGGAATACTCCGGTAAAAGCCTCTTGCCTTT
GGAGACTGTCGCAGAAAACCACGACCATAGTATGCTACAGCCTGAGAACTCCTTGACCACG
ACCACTGATGAGAAAATGTTCAACCAATGTGAGAGTTCAAAGAAGAGGACGCGTGCCACA
ACAACTGATGTAAGTTGTACAAACGTTTTTTTTTTTTTTCAGCCGAAACCATAATTCTAAATA
TTTTAATGTTCTTGAAACCAAATCAGAAGAACAAGAGAGCCAACAAGGCACGAAGGAGCC

AGAAATGCGTAGAGATGAGTGGCGAAAATGAAAATAGCGGCGAAGAAGAATATACGGAGA
AGGCTGCGGGGAAGAGAAAGACCAAACCACTTAAGCCGCAAAAGACTTGTTGTTCGGATG
ACGAATCAAACGGTGGAGACACTTTCTTGTCCAAAGAAGATGGCGAGGACTCTAAGGCTC
TCAACCTCAACGGCAAGACTAGGGCCAGCCGCGGCGCGGCCACAGATCCTCAAAGCCTTT
ACGCAAGGGTAATTTACACATAAACTTTAAAAATTAAGGCTTCGATGTTATTCAAACATGTA
CACAAATGTGATGATGAGTCTTATGTATATGCATTGTACAAACATAACAAAATTTATTATGATA
GCTGAAACAATTAAACAAAGTCCATTGTATGATGGTGCAGAAAAGAAGAGAGAGGATAAA
CGAGAGGCTAAGGATTTTGCAACATCTCGTCCCTAATGGAACAAAGGTACATATGTCTTTCA
TTCATGCAAACCTTTTTTTTTCCATGCAAACTTATTTATTACTACGTAACCACATCACTTTTTA
CTCATTTCGTTTCATGATTACTAAGTAAATTAAATGTGTTAAATATGTATGTATCAGGTTGATAT
TAGCACGATGTTGGAAGAAGCAGTACAATACGTCAAATTTCTACAGCTCCAAATTAAGGTAC
GTTAGGTTCTCAATGCTTATAATATAAATTAAACCATAGAAAATATTGTATCAATTAAAGATAG
AGAATTAAATATTGGTTATAATGAATAATGTTATGTTATACAGTTATTGAGCTCTGATGATCTAT
GGATGTATGCGCCTATTGCTTACAACGGAATGGACATTGGCCTTGACCTAAAACTCAATGCA
CTGACCAGATGATTCAAGAGACACCTTCGGATTTTAATTCTATTTATTTATATATATATATATAA
TTTAATACTAGAGATATGAAGAGTCACATTTTGACGTTTTATACTCTAAATATTATTTTATATTT
GTATGTCGAGTTATGTCCAACAATTTTTTTGATTCTTGAGATATATATTGGTGTACGATCATCG
TTTTGATAATGGATTCGTTTATTTTATCTCTTTTTTTTTGTTTGAATTATTTGATTCGTTTACGTC
ATACAC

>AtbHLH085
TATACATTCCAACTCCTCTTCCTCATTCCCTCACTTCTCACAAGACTTCCAACTAACAATCTT
CTTAATTAAGATTAATCTCTTAATTAAGACGCTTAGTTAACAAAACATATAAAAATGGAAGCC
ATGGGAGAATGGAGCAACAACCTCGGAGGAATGTACACTTATGCAACCGAGGAAGCCGATT
TCATGAACCAGCTTCTCGCCTCTTATGATCATCCTGGCACCGGCTCATCCTCCGGCGCAGCA
GCCAGTGGTGACCACCAAGGCTTGTATTGGAACCTTGGTTCTCATCACAACCACCTTAGCCT
CGTGTCTGAAGCCGGTAGCTTCTGTTTCTCTCAAGAGAGCAGCAGCTACAGCGCTGGGAAC
AGCGGATATTACACCGTTGTTCCACCCACGGTTGAAGAGAACCAAAATGAGACAATGGACT
TTGGGATGGAAGATGTGACCATCAATACAAACTCATACCTTGTTGGTGAGGAGACAAGTGA
GTGTGACGTTGAGAAATACTCTTCTGGAAAGACTCTTATGCCTTTGGAAACCGTAGTGGAG
AACCACGATGACGAGGAAAGCTTGTTGCAATCTGAGATCTCTGTGACTACTACAAAATCTC
TCACCGGCTCCAAAAAGAGATCCCGTGCCACATCTACTGATGTAAGTCTTTAATTTTCGAAC
TGGTTGGCAATTAGCTAGAGAGATCACATTTGGTTTAGTGACTAATATAGAAACGGTTTGCC
ATGGACTCAGAAAAACAAGAGAGCAAGAGTGAATAAGAGGGCCCAGAAGAACGTAGAGA
TGAGTGGGGATAACAATGAAGGAGAAGAGGAAGAAGGAGAGACGAAGTTGAAGAAAAGA
AAGAATGGGGCAATGATGAGTAGACAGAACTCAAGCACCACTTTCTGTACGGAGGAAGAA
TCAAACTGCGCTGATCAAGACGGTGGAGGAGAAGACTCATCCTCTAAGGAAGATGATCCCT
CAAAGGCCCTCAACCTCAATGGTAAAACAAGAGCCAGTCGTGGTGCAGCCACCGATCCTC
AAAGCCTCTATGCAAGGGTAAATTGCTTTGTTTGTTTCCCACAAAAAAGATAAAATATCAAA
TTTTTATGGAAGATTATGTTTCAAAAAATTAATTTGGTTTTTTATTATTTTTTTGTCGTGCAGA
AAAGAAGAGAAAGGATTAACGAGAGACTAAGGATTTTACAAAATCTCGTCCCCAATGGAA
CAAAGGTAAAAAAAAAAACTCATGCAAACGTTTAAACATGCAGAAGATATGGCTCTTTTAC
ATGCAAATTCATTAATTATACAAGAAATTGTTAATAAAGATTCTAAACATTTACAATTGCTTAA
ATACGTAACAGGTCGATATTAGTACAATGCTTGAGGAAGCAGTTCATTACGTCAAATTTTTG

CAGCTCCAAATTAAGGTAAGCAAGGGTTTTAAATACAAATCGTTTTTTTTTTGTACGTTCAAT
TGTTATATGAATATGATCTAACTAATATTTTAAAATATAATTACGCAGTTATTGAGCTCTGATGA
TCTATGGATGTATGCGCCGATTGCTTTCAATGGGATGGACATTGGTCTCAGCTCACCGAGAT
GAAGCAGAAAACTTGTGATTTTAGTTGGATATTTTCTCTTATATATAACCAAAAATGTAATATT
CTTTTTCTTTTTTAATATTCTCCGCCTTCGGACGAGTTCGATTATAAATTCTCGTACGATGTTC
AAATATGAGAGAAATGGAGAGTCACGTTCGATTTTTTTATACTTTCATTTTCTTTTATATCAC
ATATTTACATTTC

>AtbHLH086
GCTTACCAATCTACAATCTTAAAACCATTTTTCGTTGTTTCGATTTCTCAAATCTCCTTAACCT
CTCAAAGCAAAACCAAAGACATAAAAAAGAAACCCCATACATCTCACTAGAACACCCTTTA
GTACCAATGTCACTCATTAACGAACATTGCAATGAGCGTAATTACATCTCAACCCCAAATTCT
TCAGAAGATCTCTCTTCACCACAGAATTGCGGATTAGACGAAGGAGCTTCAGCTTCAAGCA
GTAGCACCATAAATTCTGATCATCAAAATAATCAAGGGTTTGTGTTTTACCCTTCCGGGGAA
ACCATTGAAGATCATAATTCTTTGATGGATTTCAATGCTTCATCATTCTTCACCTTTGATAATC
ACCGAAGCCTTATCTCTCCCGTGACCAACGGTGGTGCCTTCCCGGTCGTGGACGGGAACAT
GAGTTACAGCTATGATGGCTGGAGTCATCATCAAGTGGATAGTATTAGCCCTAGAGTCATCA
AAACTCCAAATAGCTTTGAAACAACGAGCAGTTTTGGATTGACTTCAAACTCCATGAGTAA
ACCGGCCACAAACCATGGAAATGGAGACTGGTTATACTCTGGTTCAACTATTGTAAACATCG
GTTCAAGGCACGAGTCCACGTCCCCTAAACTGGCTGGCAATAAACGGCCTTTCACGGTAAC
TATAAATTTAAGATTTGTGATTTGTCTTTTTCATATATCATTTAGATCAATCAAAGATGTTTAAA
GTTTACCATCTAATGATCGATAGGGAGAGAACACACAACTTTCAAAGAAGCCGAGTAGCGG
TACGAATGGAAAGATCAAGCCTAAGGCAACAACTTCACCTAAAGATCCACAAAGCCTAGCA
GCCAAGGTTTGCGTTTTTTCTATTCATCAACATTTATTGTCAGGGTTCTCTTTTTGGATTTAAG
TCAAGGAAATTAAGAAATATATATGTGCTTTTGTCAATAAATTAACCAATACGAAAATATGTT
TCTTGTATAGAACCGAAGAGAAAGGATAAGCGAACGCCTCAAGGTATTGCAAGAACTTGTA
CCGAATGGTACCAAGGTATGATAAATGTATAAGAAATTTAATTTTATCGTAACCTTTTTTTTCA
AACCTAGAGCGTAATAAATATTTTTCTGTACTAATTTTTTTTTTTAAAATCTTTATGGTGGTTT
TTATGTAGGTGGATTTGGTAACTATGCTTGAGAAAGCAATTGGCTATGTAAAGTTTCTTCAAG
TACAAGTTAAGGTAATTAATCTTAACAAACATTCCGTCAACTATATATCATACTATGTCATATAT
AAGTAGGGTCTTTCAAGTATATTGATATAAACAAAAAAACTATGTCTTTTTATAGGTACTTGC
AGCCGATGAGTTTTGGCCGGCACAAGGAGGGAAAGCTCCGGACATTTCTCAAGTTAAAGA
AGCTATTGACGCAATCCTCTCATCATCACAACGAGATAGTAACTCAACTAGAGAAACAAGTA
TAGCAGAATAATTCCGACCAAAACAAAAAGTATAGCAGAATAATTAGTTACTAAATGTATCG
GCATTAATTATAATACCAAACAAGTCATCACTTTTGAATTCGGTTTAAAAAATAGTTAATTGG
TGACGTTACAGATCGAGAAGTTATTGATTCCTTTAAGGCTTTAATTATGTAAGAGCCAACAC
ATTGAAGTCACCGACGATATGTCAGACGTCGGTCCACACGAGCATGGAATCTTATAGTAGAT
TAGATATGTAATAGTTATATATGTGGAGGTATGTTTCTATTTTTGTATGTTTTGTAATTCTCCAA
GTATGCG

>AtbHLH087
ATGAATAGTAGGAGGGGTGTATAGGGCTTTCAAGTCTTTTCTTACAGAAACTATCTATAACTC
TAGCAGAGAGCTCATTATAGAACAGAACAGAAACAGAGCTTTCTAAGTCTACAGCACAGAA
ATAAACCTCATTTACAGACTGTTTTACTTTCTTTCCTTTTTGAGCATTTCTTCTTTCTGTTTCT

TTCCTCTGTTTTCTCTTTCTTTCCCTTTTTGGGTGTCATCTCTTTAAGACAGAAGAAACGTTT
CTCAGAGTTTCCTGTGAACTCATCTGTTTGTTCTCTTCTTCCGGTTTCACTTTTTCATGTCCT
GCCGTTATTACAACGAGGTTGGTTTAAAGATCAGCTTTTATCATTTTTCCCTGCATGTAACAA
GAAATATTTAGGTTTCATGTCTGTTTACAAATATGCTCTGTTTCTAAGTATGCCTCTCACATTT
GTCTTTGTTTTGGGTGTTTTTAGGATTGTGTTTGATCCGATGGAAGGATTGGAATCTGTGTAC
GCTCAAGCTATGTATGGAATGACACGAGAGAGCAAAATCATGGAGCATCAAGGATCAGATT
TGATTTGGGGAGGAAATGAGCTAATGGCTCGAGAACTCTGTTCTTCTTCTTCTTATCACCAC
CAACTCATTAATCCGAATCTTAGCAGCTGTTTCATGTCTGATCTTGGAGTCTTAGGTGAGATT
CAACAGCAGCAACATGTTGGCAACAGAGCTAGCTCGATAGATCCATCATCACTCGATTGTTT
GTTATCTGCGACGTCGAATAGCAACAACACCTCGACGGAGGACGATGAAGGAATATCTGTG
CTTTTCTCAGATTGTCAGACTCTTTGGAGCTTTGGTGGAGTCTCATCTGCAGAGTCTGAGAA
CAGAGAGATCACTACTGAGACGACAACAACGATAAAGCCTAAGCCTTTGAAGAGAAACAG
AGGAGGAGATGGAGGAACTACTGAGACTACAACAACAACAACAAAACCTAAGTCTTTGAA
GAGAAACAGAGGAGACGAGACAGGAAGTCACTTTAGTCTTGTTCATCCTCAAGATGATTCG
GAGAAAGGAGGTTTCAAGCTTATATACGATGAGAATCAATCGAAATCAAAGAAACCAAGAA
CAGAGAAAGAACGAGGCGGTTCTTCGAACATTAGTTTCCAACATTCAACTTGTTTGTCTGA
CAATGTCGAGCCCGATGCTGAGGCGATTGCACAAATGAAGGAGATGATATACAGAGCGGCT
GCATTTAGACCGGTGAATTTCGGGTTAGAGATTGTGGAGAAGCCTAAGAGGAAGAACGTCA
AGATATCGACGGATCCTCAAACGGTTGCAGCGAGACAGAGAAGGGAGAGGATAAGTGAGA
AGATTAGGGTTTTACAAACATTGGTTCCAGGTGGGACGAAGATGGATACTGCATCAATGCTT
GATGAAGCTGCTAATTATCTCAAGTTCCTTAGAGCACAAGTAAAAGCTTTAGAAAACTTGA
GACCCAAGCTTGACCAAACCAATCTCTCTTTCTCTTCTGCTCCTACATCGTTTCCATTATTCC
ACCCATCTTTTCTTCCATTGCAAAATCCTAATCAAATCCATCATCCAGAGTGTTGACAGATTA
TAAACTTTTGAGTTTCATCATCATCAACAGAATCATGGCGTCTTGATTGTTTTAGCAGTTCTC
AAGAAAGGCAACTTCTGTGACAAGGGTGGTGTCGGGCAGTGTTGTTTACACTTTCCAGTCT
TTGTTTTGCATTTCTTTTTATATAAAGTTTGTATTTTATATAGAATCTGTGGAATTCGAGGGTT
GAAATATTGTGAAAAACAGAGCCGCAAGAGGTTAATTACAGTCTCTGCAATATTTTCAACCT
TTTATTACTTTATTAGAGTAAAGATAGCGTAAGATAATTGCAATTATGAAGCGATGATTATCAA
CAAAAGTGTTTAGAAAGTGCCTCC

>AtbHLH088
CTTCTTGTTGTATACTATTGCTTGAGTTCTGATTGGGCACAGTAGTACCATTGCCATTTCTCTC
ACACATACCGTCTCTTTCTCTCATCATCAATCATCAATCATCCAAAAGAAAAAACCCTAAAA
TTTCACTTGTAAGCTTTTCACCAGTTTCTCTCCATACCCATTTTATCAGCTTCTCCATATCTTT
CTCTATGGATTCTGACATAATGAACATGATGATGCATCAGATGGAGAAGCTTCCTGAGTTTTG
TAACCCTAATTCCTCTTTCTTCTCTCCCGACCACAACAACACTTACCCTTTTCTCTTTAACTC
CACTCATTACCAGTCCGATCACTCAATGACCAACGAACCAGGTTTCCGCTACGGTTCCGGTT
TACTCACTAACCCTTCTTCTATCTCTCCCAACACAGCTTACTCTTCCGTTTTTCTTGACAAAA
GAAACAACAGTAACAACAACAATAATGGCACGAACATGGCAGCTATGCGAGAGATGATCTT
CCGTATCGCCGTGATGCAACCGATCCATATCGATCCCGAGGCGGTTAAGCCACCGAAGAGG
AGGAACGTCAGGATCTCTAAAGATCCTCAAAGCGTGGCGGCTAGGCATAGAAGGGAGAGA
ATAAGCGAGAGGATTCGGATTTTGCAACGGCTTGTTCCTGGTGGGACGAAGATGGATACAG
CTTCGATGCTCGATGAAGCAATTCATTATGTGAAGTTTTTAAAGAAACAGGTGCAGTCTCTG
GAGGAGCAGGCGGTGGTTACTGGCGGAGGGGGAGGAGGAGGAGGAAGGGTTTTGATCGG

TGGAGGTGGAATGACGGCGGCGAGTGGTGGTGGTGGCGGCGGGGGAGTGGTTATGAAAGG
GTGTGGAACAGTGGGGACTCATCAGATGGTGGGCAATGCACAGATTCTTAGATGATGATGA
TTTTTAATTTTATTATTATTATATTAATGTTGGAGAAAAAGAGAAAAATGATTCTGGAGAGGG
AAGCCAAGTAATTTATGTGAGAGTCTTTAATTTAACTTTATTTCTTGTTTAGATAATGTGTAA
TGATGGTTTTTAAAGCCAAAGACTCTCCATGGTTGTTGGAGCGAGTTTGTAAATTAGGAACT
ATTATCATTGTATCCTTGTTAATCAAATAATTAAAGCAGTATTATGCTTTCAGCTATCAGG

>AtbHLH089
CAAACGTATAGGCATTTCAAAAAGAGAGACAGAGAGAGAAAAAGAAAGAGTTGTTAATGT
TAAGTCTCTTCTTGGCGTGAATTCGGAAAAAGTTCTTCGGAGAATCATCATCAGCCAAGTCG
GTAACAATATACCCTTCTCTTACTCTTTTATTCCTAATAACCTGATCATTAGATATACATTACAA
GAATTCATCTAAGCTTAGACGATTTTTAAGTTGGTTAGAACAGATTTGTTCAAGATTAGGTTG
CAGTTTTCAATATATAATCTAGCAAATCAAAACTGTGTCAATGTTTTCTGATTTTGAAACCAT
CTTTCTTAGCCCTTTGGACCGGTTTTCATGGGAGGAGGAGGCATGTTTGAAGAAATTGGGT
GTTTTGATCCAAATGCTCCGGCAGAAATGACTGCTGAGAGCAGTTTCTCTCCGTCCGAGCC
ACCACCGACGATCACGGTCATCGGTAGCAACAGTAACAGCAACTGCAGTCTAGAAGATCTC
TCTGCATTCCACCTCAGCCCACAAGACTCTTCTCTACCTGCCTCAGCCTCAGCTTATGCTCAT
CAGCTTCACATTAATGCTACTCCTAATTGTGATCATCAATTCCAGAGTTCAATGCACCAAACC
CTCCAAGATCCATCGTATGCGCAACAGTCGAATCATTGGGACAATGGTTACCAAGATTTTGT
GAATTTGGGTCCTAATCATACCACTCCTGACCTACTCAGTCTCTTGCAATTGCCAAGATCCTC
ACTTCCACCTTTCGCAAACCCTAGCATTCAAGACATTATCATGACAACATCTTCCTCTGTCGC
CGCCTATGACCCGCTCTTCCATCTGAATTTTCCTCTGCAGCCTCCTAATGGGAGTTTCATGGG
AGTGGATCAAGATCAAACTGAGACTAACCAAGGAGTGAACTTGATGTATGATGAAGAAAAC
AACAACCTCGACGATGGTCTTAACCGTAAAGGCAGAGGATCGAAAAAGCGTAAAATTTTCC
CTACAGAACGCGAGAGAAGAGTTCACTTCAAAGACCGGTTTGGTGACTTAAAGAATCTAAT
TCCAAATCCCACCAAGGTTTACACTTACTTAAATACTAACTTCTTCTTCAAGTAAGAAATCTT
CAGAAACTAATTAAACCCTTTTTTCTTATATCTCTAGAATGATAGAGCATCAATCGTCGGAGA
AGCGATAGATTACATCAAAGAGTTGCTAAGGACGATCGATGAGTTCAAGCTGCTAGTGGAA
AAGAAGAGAGTGAAACAGAGGAACAGAGAAGGAGATGATGTTGTTGATGAGAACTTCAA
GGCTCAAAGCGAAGTAGTAGAACAATGTTTGATCAATAAGAAGAACAATGCCTTGAGATGC
TCATGGCTCAAGAGGAAATCGAAATTCACTGATGTCGATGTACGTATCATAGACGATGAAGT
TACCATCAAGATCGTGCAGAAGAAGAAAATCAATTGTTTGTTGTTCGTATCCAAAGTCGTTG
ACCAACTTGAGCTTGATCTTCACCATGTAGCTGGTGCACAAATCGGAGAGCACCACAGTTT
CTTGTTCAACGCCAAGGTTTGTCATAAAATCTCAATTTAATCCATAGTCAGACACTATTAGAC
TAACTAACGAATTGTGATATAACAGATTAGTGAAGGATCTAGTGTTTATGCAAGTGCCATAGC
AGATAGAGTAATGGAAGTGTTGAAGAAACAATACATGGAAGCTCTTTCGGCGAATAATGGT
TATCACTGTTACTCTAGTGATTAAAGCGTTTTCATCTAGCTGGCTTATTACTTTGAATATGGAT
AATTAATAATCACATTAGTTATATTTTCGTATTATTGTTCTTAGCTTGTAGCTATGCCTCTGTTT
TTACTTTTTCCTTGTTTTGGTTTAATCACATATTCCAATCATATCCCTCG

>AtbHLH090
CTCTGGGTTTTAATTCAAAAAGTGAAGTTGGTTACCATTGTCTTGTACGATCGTATATTCCCG
ATATGATTTGTTTTGACAGAGCAACTGCTTTGTATATATATGCCAAATATCTCTCTCTCTCTCC
CTCTCTCCTTGCATCAATTTATAAGTAAGACCAAAAGAGATCACCAAAACTCAGGCTTCTTG

TTTGGCTTTTCTTATTGTTGATTGAGAGAGTGAGAGTAAGAGAGAGAGAGAGAGAGAG
AGTGAGAGAGAGAGAGAGAGAGAGTGAGATGATGATGATGAGAGGTGGTGAGAGAGTGA
AGGAGTTTCTTCGACCCTTTGTCGATTCCAGAACTTGGGACTTATGTGTTATCTGGAAACTC
GGTGATGATCCTTCTAGGTTCAACACACTCTCCGGTTTTTCTTTGTTATTTGTTCGTTGTTCAT
GTGAATCATCATCAGCTGTCTACTATTTTGTTTCAGTAGCAAAGCTTCCGTTTTTATGTATACT
GATGGGTTTTTCGTGTTTGTCTTTTATCTTTGCTTTCTGAATTAGGACTTTTTTTTTTGTTTTT
GCTCTGTTATTGGCTTCCTGAAACTATTTTGTTTCAGTAGCAAAGTTTCCATTTTTTTGATTTT
GATTAGTCCTTAGTAACTTCTGGCTCTGTTCTATCAAATTTCTCTGGTTTCTGTTTTTTTTTTT
TTTTGCTCATCTGGTTTTTGATTTTAATGGTTTATGGTTTGTTTTGCTTTACCATCTACCTCCTG
TTCTTGGTTTTGTCTTGTCTAGTTTTACTAAAAGTTTGTGGGTTTTATGATCTTCATCTCGTAA
TTGGTATGAATTCCATTTAAACTACAGGTTTATTGAATGGGTGGGATGCTGCTGCAGTGGTTG
TTATATTGATAAGAATATAAAGCTTGAAAACTCAGAAGAAGGAGGAACTGGGAGAAAAAAG
AAGGCTTCTTTCTGTAGAGATGACCACAACAAGCATCGTATAAGAACCTTAGCTTGTGAAG
CGCTTTCTCGTTTTCCTCTCTTCATGCCTCTCTATCCCGGGTATGTTTCCATCTATTCAACCCC
CTTTCAAGTTAATTTTTTCTTCAAGAATAATATGAAGCTTCTTTCTCAATTGCAGGATTCATG
GAGAAGTAGTGATGTCAAAATCTCCAAAGTGGTTGGTTAATTCAGGATCTAAAATGGTAAA
AAAGACTCTTTTCGTCAGCCTTTTTTACTCGTTTGATCATAAGAACAATCTGATCTCCATGTG
TTTTGGCAGGAAATGTTCAGCACTCGTGTTCTTGTTCCTGTGAGTGATGGTCTCGTTGAGCT
GTTCGCTTTCGATATGGTAAAAACATGTTAGCATTTCGCATAGAAAGTCTGTCATTGGTTTTG
GTGGTTACTTCATAAAATGTTGGTCTGTATCAGAGACCGTTTGATGAAAGCATGGTGCATTT
GATCATGTCGCGTTGTACCACCTTCTTTGAACCATTCCCTGAACAAAGGCTGCAGTTCAGGA
TCATTCCCCGAGCAGAGGAATCTATGAGTAGCGGTGTGAATCTCAGCGTTGAGGGCGGCGG
ATCATCAAGTGTTTCCAATCCCTCCAGTGAAACTCAGAATCTTTTCGGCAATTACCCGAATG
CTAGTTGTGTGGAAATTCTCAGGGAGGAACAAACACCGTGTTTGATAATGAACAAGGAAAA
GGATGTTGTGGTGCAAAATGCCAACGATTCCAAAGCTAATAAGAAGCTGCTTCCTACGGAA
AACTTCAAATCAAAGAACCTTCATTCAGAGAGGAAAAGAAGAGAGAGAATTAATCAGGCC
ATGTATGGTCTAAGAGCCGTAGTCCCTAAAATCACAAAGGTTTCTTCTGAAACTCAATGCAG
TTTCTGGATCACTCTTCTTGTGGTCAATGTAGCACTTTCCATAGGATTATTATATTTATCTTTCT
TTGTGCTATATGCAGTTGAACAAAATTGGAATTTTCAGTGATGCTGTTGATTACATCAATGAA
CTGCTAGTGGAGAAGCAGAAACTTGAAGATGAGCTTAAAGGAATCAATGAGATGGAATGC
AAAGAAATCGCTGCAGAGGAACAATCTGCAATAGCTGATCCAGAAGCTGAAAGAGTTTCCT
CTAAAAGCAACAAGAGAGTGAAGAAAAACGAGGTATTATTCGAATATATAGACCTTCAAGA
TAATCTATATCTGAGTTGTAAAATCCCACAAACTCTGGTTTCTTTTCAGGTGAAAATTGAAGT
CCACGAGACTGGTGAGCGAGATTTCTTGATTCGGGTTGTGCAGGAACATAAACAAGATGGA
TTCAAGAGGTTGATAGAAGCTGTAGATTTATGTGAACTTGAGATCATTGACGTCAATTTCAC
CAGACTCGATCTCACAGTCATGACCGTTCTCAATGTCAAGGTAGTAACGTAATCACATCTCA
TTGCCCCTGCACGTAATCTCAGATTCTTGAATTCTGATAAACAGTTTTGGATTTATTCATTCTT
AGGCGAACAAAGACGGGATTGCATGTGGAATATTGAGAGATTTACTGCTCAAGATGATGAT
AACTTCAATATGAAAACTCAGTAAAACAGAGTACATAACCTCAAAAGGTCAAATGTTTTGTT
TGCTTCAGTCACAAGTTACTGCTAGAGATATGATTTCTCACAAGCTTTTAGGTGACAGTTTA
CATGTTTGAAACTATACATTCTTCTGGTTTGTCATTGGGTTTTCATTAAGATCTTTCACAAATA
TAATTCTAAAGGGTCGTCGTCCCTAAGAACTAAGAAAAACAAAACACAACAACTTGAGTTT
AATTTATTGGTTTCAATG

>AtbHLH091
ACGTCCAAAAAACCCTCTCCTATGGGAACAAGAAAGACCCAAAAGACACAAACACACACC
CAAACTCCATCAAACAGAAATAAAGCAAACCAAAAAAGCTTCTCTCTTTTTTCCTACATTTG
ATTTAAGCTCTAAATGTTGAAATGATCTTTGATTTGACATTGTGAAGCTGAAATATAGCTGGT
GGTAACCTTCAAAGACGACAAAGAAGGAAAAAGATCATCATCTTCTAACTTTTGGTAATTA
GATAATTTCATTCTCTAAACAAAGTCTGATTTTTGAATCAATACTAAAAAAGTGATAGAAGA
GAAGCTATCAAAACACAAAAACAGAGGATTTTTCTTTTCCAATCTAAAAGAAAGGTGCTTA
AGGGAATTAGTTTGAGATTAGGTTTTAACTGAGTTTATACAAACTTTCTTTATTTAATGAAAT
TAACTTAGATTGGCTTTTGTCTTAAAGATTAATTATGGTTGTGAAAATTAGTTTTGGTTGCAG
AAAAAAATCAGTTGAAAATGTATGAGGAAAGTTCATGTTTTGATCCTAATTCGATGGTGGAC
AACAACGGCGGCTTCTGTGCGGCGGAAACAACTTTTACGGTAAGCCACCAGTTTCAACCAC
CGCTTGGAAGCACAACAAACAGCTTTGATGACGATCTTAAGCTCCCAACAATGGATGAGTT
CTCTGTTTTCCCTTCTGTTATCTCTCTCCCAAACTCAGAAACTCAGAACCAAAACATCAGCA
ACAACAACCATTTGATCAACCAAATGATTCAAGAATCGAATTGGGGTGTTTCTGAAGACAA
CTCTAATTTCTTCATGAACACTTCACATCCAAACACAACAACAACTCCAATCCCTGATCTTC
TCAGCCTCTTGCATTTGCCTAGATGCTCTATGTCATTACCAAGCTCTGATATAATGGCGGGTT
CTTGCTTCACGTATGACCCGCTCTTTCACTTAAACCTTCCTCCACAACCTCCATTGATCCCTT
CTAATGACTACTCAGGGTACTTGCTTGGTATTGATACCAATACTACTACTCAAAGAGATGAGT
CTAATGTTGGAGATGAAAATAACAATGCTCAGTTTGATAGCGGAATCATCGAGTTTAGCAAA
GAGATTAGGCGTAAAGGAAGAGGGAAGCGAAAGAACAAACCTTTTACTACAGAACGTGAG
CGAAGATGTCACTTGAATGAGCGGTACGAGGCCTTGAAATTGCTCATTCCTAGCCCGAGTA
AGGTTAGTTATTTACTTATGACAACAATCTTTCTTGGTTTAGCTTCATTCATTATCTCCAATGG
CACTTTGCTTAATTCATATTATATGTTATTTAGGGAGATAGAGCATCAATTCTTCAAGATGGAA
TCGATTACATCAACGAGTTACGCAGAAGAGTAAGTGAGCTTAAGTATTTGGTAGAGAGGAA
GAGATGTGGTGGGAGACACAAGAACAATGAAGTAGACGACAACAATAACAACAAAAACTT
GGATGATCATGGTAATGAAGATGATGATGATGATGATGAAAACATGGAGAAGAAGCCGGAG
AGCGATGTAATAGACCAATGTTCAAGCAACAACTCGCTGAGATGTTCATGGCTGCAGAGGA
AATCAAAAGTAACAGAAGTTGATGTTAGGATTGTTGATGATGAAGTAACAATCAAAGTTGTT
CAGAAGAAGAAGATCAATTGTTTGTTACTTGTCTCCAAAGTACTTGATCAGCTTCAGCTTGA
TCTTCACCATGTTGCCGGAGGACAGATTGGTGAGCATTACAGTTTCTTGTTCAACACCAAG
GTTAGTGATCAATGAATATATATATATATATAACAAATCTTGGTTTATGGATTTTGATATATACTG
GATTTAGTTTTGGTAATAATGATTGAATAAATTGCAGATATATGAAGGATCAACAATATATGCA
AGTGCAATAGCAAACAGAGTGATTGAAGTTGTGGATAAACACTACATGGCTTCTCTTCCCA
ACAGTAACTATTAATTGTTAGGCTAACTTCATCTATTTTCTAACGTTTTTAATCCATGTAATTT
TTACTCATTTGCAAAACTAAATAAAAACTCATTTTGGATGATTTGGATGTTTAGTTTCTAACA
AGATATGTATGATTTGGATCCCTC

>AtbHLH092
ACACAAATCAAGATTGTCAAACACACTAAGAGAGAAAGATGGATAACTTTTTTCTAGGTTT
GAGTTGTCAAGAAGAAAACAACTTCTGGGATCTAATCGTGGCCGACATCTCGGGTGATAGA
AGCGTCAGTGTACCTATCAGAAGCGCCTTTAGGTCATATATGAAGGACACGGAACTGAGGA
TGATGTCGCCGAAGATATCTTCTTCGAAGGTGAACGTGAAGAAGAGAATGGTCAATCTTCT
GAGAAAGAATTGGGAGGAGAAGAAAAATACGGTGGCTCCAGAGAAGGAAAGAAGCCGGC
GACATATGTTGAAAGAAAGGACGAGAAGAGAGAAACAAAAACAGAGTTACTTAGCTCTCC

ATTCTCTACTACCATTTGCCACTAAGGTACCTTGATTATTTGTGTAATATTAAACTTGAAACG
AACTTTATTTTTCTTAATGACTTGGAAAGAAAATCATAGTAGTTCTAACAAAAATCCGAATTT
TTACAGTTCAAAATGGCACACAAAATTAGATATTCGGTAGAAATGGATTAGATTCATGGATA
CTAAATTTTCAGAATTTCGACTACTATGTCTGTTCACTGATCTTTGATGTTTGTATGCTTAATT
ACATGCATTGATCTGGTTTTAGAAGCTTATCTTAAATATCTATAAATTTATATTGAGTAAGGTAT
AATATAAAGTTTTGAGTATGATAAATTTCCAACTACAATCATTTGTATAAAATGTTTGTAAGTT
TTTGCACTGGAATCTTTTCTTTTATCACTATTTTCTTTTACTTAAGGATTTTGATATAGAAACA
AGTTTAAAGAACATCATAGATTGATATCGAAACAAGTTTCAAGACCATCATTTCTCCTTATAT
TCTCATTTTATTTCAGAATGACAAAAATTCGATTGTCGAAAAAGCCGTGGATGAGATTGCGA
AATTACAAAGATTAAAGAAAGAACTAGTGAGAAGAATTAAAGTGATTGAGGAAAAATCAG
CAAAGGATGGTCATGATGAAATGAGTGAAACAAAGGTTAGGGTTAATCTAAAAGAACCTTT
GTCTGGACTAGATTCAATGCTTGAAGCGCTTCATTATCTTAAATCAATGGGAACAAAACTGA
AAACAGTCCATGCCAATTTCTCTCCTCAAGAGTTTTCTGCGACCATGACCATCGAGACTCAG
GTACATATTTAATTCGATTCGAATTGGTTGAATCCGGTTTTGGTTTCTCTTTGGTTATGACAAC
TTGACAAGTACATAAAGGTAATAATAGGTAGGTTGTTTTGTGTTAGTAGCAAAAAAAAAGTA
GATGGTTGTATGTTTTTTTAATTAAGTAGTTAGATCTGTATTAGTGTGATGATATTAAATTTAA
AGTTAACTTTTTGGCAGATTAGAGGAGAGGAGGTTGAAAAGAGAGTGGAAAGAAGACTCC
AGGAAACTGAATGGAAACTTCTCTTTCTCCCAGAAGCTTCCTTTTACAAAGACTACTAATTC
TGTTGACTTTCTGTCATTAATTTTCTTTTGTATTTAAATTTGTATATAGTTAAATACTTAAATTA
ACTGGTCATTGCTTTATTGACACACATAAAGAAAAATAATGGGGCACACTACTTTTGAGTTT
TGAGTTTTGAGGTTTGAAAAATAAAACTAAGAAAATAATATTGATAGAAT

>AtbHLH093
CGGGAATCTTTTATTTAATTAATAGTATACTCTCTCACACAGACAGACATCTCTGGATTTATAT
ATAAATACAGTTCATCTTCTTTAATCCACAAACTCACACACACACACACACACTCTGTTTTC
TTCGTCCATTCTATTACCAGAAAACAGAGTTTGATAGAGAGAAAAGAATGGAACTGTCGAC
TCAAATGAATGTGTTCGAAGAGCTTCTTGTTCCGACAAAGCAAGAAACAACCGACAACAA
CATCAACAATCTGAGCTTTAATGGCGGATTTGATCATCATCATCATCAATTCTTCCCAAATGG
ATATAATATTGATTACCTCTGTTTCAACAATGAAGAAGAAGACGAAAATACCCTTTTGTATCC
TTCTTCTTTCATGGATCTAATCTCTCAACCTCCTCCATTGCTTCTTCACCAACCGCCACCGTT
ACAACCACTGTCGCCGCCGTTATCCTCCTCCGCGACCGCCGGAGCAACATTTGACTACCCTT
TTCTTGAGGCTTTGCAAGAGATAATTGACTCTTCTTCCTCATCGCCTCCATTGATCCTTCAAA
ATGGTCAAGAAGAGAACTTTAATAATCCGATGTCGTATCCCTCTCCATTGATGGAGTCTGATC
AGAGCAAGAGCTTCAGTGTTGGTTACTGTGGAGGAGAGACGAACAAGAAGAAGAGCAAA
AAGCTTGAAGGCCAACCTTCTAAGAATCTCATGGCGGAGAGACGACGGAGAAAACGACTT
AACGATCGTCTTTCTATGCTCCGATCCATCGTCCCAAAAATCAGTAAGGTAAATTACAAAAT
GCTAATCTTTTTTTTCTTTCAAACCATTAATAATAATTGTAGTCAAAATCAGCAAAAGATTTG
ATCATTTTTTTAATGAGTAGGTTAATGACTGTATTATTATATGTGTTCATAAAGAAAATGACCT
TTTTAATCTTCTTGATTTACCTCAAAATCTAACCCTAGCTAAGGTTTTTGTGTTCTTTGAAGAT
TTATTTAACATTTAATTCAAAAAATTGGTCTCAGGAAAATCAACAAAAGATTCGTCCTTTTAA
ATGAGTGAATTAGTTATAAAATTGGTTTCAAATTCAGCCTTTTGTATTTTAACATTATGTGTTT
GTTTCTTCTTTTTTGAAACCTTAGTTTTCTTCTTCAAGATTGTTTAATTATTGTGTTCTTACTTT
TAAGGGAATCTACAGAACCGGCTGAAAAGTGATAAATCATTTAAAAGTGTTTTAAAATAATG
ATTATTGGTATGTTCAAAAAAATCAGATGGACAGGACATCGATATTAGGAGATGCCATAGATT

ACATGAAAGAGCTTTTAGACAAAATCAACAAATTACAAGATGAGGAACAAGAACTTGGAA
ATAGCAACAATTCACATCACTCTAAGCTCTTCGGTGATCTCAAGGATCTTAATGCGAACGAA
CCTCTGGTCAGAAACTCACCAAAGGTACAAAGATATTTTACATTGCTCTTGTTTTTTTCCTTC
TACATATTAGCCCTTGTATTTATGAATTGTTATAAACAAACCCCCTGTCTTTTTAACAAATCTT
TTTGTAATTCTGTTTATGTTTTGCATAGTTTGAAATAGATCGTAGAGACGAGGATACTCGAGT
TGATATATGCTGCTCGCCAAAACCGGGATTGCTACTATCTACTGTGAATACATTAGAGACTCT
AGGCTTGGAGATTGAACAATGTGTTATAAGCTGCTTTAGTGATTTCTCTTTGCAGGCTTCTTG
TTCTGAGGTATCGATTTAATATAAAGTATTTATGCATGGAATGTTAATTATATAGTATTCGTAAT
GATAATATTGATGAGTTTTGTTGTATATATGTTTATAGGGAGCTGAGCAGAGAGATTTCATAA
CATCAGAAGATATAAAACAAGCATTATTCAGAAACGCAGGTTATGGTGGAAGCTGCTTGTAA
TGAGTCATGATTGAAACTAATAAACCAAAACTATTGTTTATTTCTAATAATACTAGCGCTTATA
AACTTTAGATGTTGTTTTAATTCCCTTGTGAGTTTGGTTTTTTTTTCCTAGGGGAAAACATTA
TGAGTTCAGTTGTATAAACAAAAGAAGGAAATTTAAAAAGAGATGTTTCTCCTAAATTATAT
CAAAGAACAAATTGAGATATAAAATAAAAGGTTATATTATGTTGCTTTGAGGGAAATAAACC
ACTTCGATAATTGAAATTAGGGTTCGTATATGACAGTGACCGATGTTCGAAGGATGCATCATC
A

>AtbHLH094
GACAAAAAGGAGAGCGACAAACACTTCCTGTCTAAATTTTGTTTTCTCTCCTTCTCTCTCCC
TCTCTCGGTTTCTCAATCTTTCTTATTTTTTCCATAATTCTGAATTTTTCTTTTCAACAATCTCC
GGCTAAAAAATGCCCTTAGAGGCTGTCGTATACCCGCAAGATCCATTCGGATATCTCTCCAA
TTGCAAAGATTTTATGTTCCACGACTTATACTCTCAAGAAGAGTTCGTAGCTCAAGATACGA
AGAACAACATTGATAAGTTAGGGCATGAACAGAGCTTTGTGGAACAAGGTAAGGAGGACG
ATCATCAATGGCGAGACTATCATCAGTATCCTTTGTTGATCCCTTCGTTGGGAGAAGAGCTT
GGTCTTACCGCCATTGATGTGGAGAGTCATCCTCCTCCACAGCACCGGAGGAAGAGGAGGA
GAACGAGAAACTGCAAGAACAAGGAAGAGATCGAGAACCAGAGAATGACTCACATCGCC
GTCGAGAGAAATCGCCGGAAACAGATGAACGAGTATCTGGCTGTGCTCCGTTCTCTAATGC
CGTCGTCGTATGCTCAAAGAGTAACTTACTAATTCAATGTTTATATATGCTATATATCATTTGG
TCAGGGTCATTCTTAGGTATATGTGTCTTTGTTTTATTACTTGCTTGGCTGTAGTACTCATTTC
TCCATAATATATTAACACAAAAGAATAGAATGAAAATGACTGAGAATATATATTGATGTTTTTA
TACAATATATTAACAAGAAAATCCACCGTAAATATGGAAGGGTGACGCGTTTATATACATACT
AATAATCTTTGCTGTTACATATTTTAATGTATTTTTTAAAATAAAATATTATGTGTCACCAAAAA
TTAGAACGAGTATGATTGAGAATATTGGGATATGTTTTAGAACAATTTCGTAATAAACTTAAG
CAATTAGCAATGATACGTGTGTGTGTATATTTATACATTCAAAGATTTTTTCAATCTTTATGAT
AACTATATGATCTGACATGAGCGGGTTTTGGTGGTAAAGGGAGATCAAGCGTCGATAGTAGG
AGGAGCTATAAACTACGTGAAGGAGTTAGAGCATATTTTACAATCTATGGAGCCGAAGAGA
ACTAGGACTCATGATCCCAAAGGAGACAAGACTAGCACTAGCTCGTTAGTGGGTCCATTCA
CAGATTTTTTCAGCTTCCCACAATATTCTACAAAGTCATCATCAGATGTACCGGAAAGCTCAT
CTTCACCGGCGGAGATAGAGGTTACGGTGGCAGAAAGCCATGCGAACATCAAGATAATGAC
GAAGAAGAAACCGAGGCAGCTTCTTAAGCTCATAACTTCTTTACAAAGCCTAAGGCTCACT
CTTCTTCATCTCAATGTCACCACTCTCCACAACTCCATTCTCTACTCCATCAGCGTCAGGGTA
CGTACATTTTCAACTTCTCATCATTACTTTGTTTGCTTTTGGGGTAATCATGAGATTAAAGTG
AGTAATGACTGTGTGTGTGGTGCATATGGTTTCACTTTGTTGATTTATTTTTTTGTCTCTTATT
GTTGGGGGGAAATTTTGGATTAGTACTATTGTGGTTATGCCTGGGCTTTAAAAAGGTTGTGA

AGAAAAATTAAAATCATTCTCTTTGTTTTTTCTTACCTCCTTTGGGTACTTAGGGCTGGGGC
CTCAATAGTTGTGGGTCAAACTCATAATCATCATTTTGATTATTATTTTTGTCAATAATAAATT
GCGTATCTTAATCGTGCCTGTAAGAATCAGGGGACCAACCTAAAGAAACGAAACACGAATA
TGTGTCTATCTATATCCACGAATTGTATACGTGGTGTTGTGTATTTATATATGTTTATGTATATGT
TTATGTACTGTGGATATATGTTCGGTGGTCACATAATTGTAGATGAGATATGTACGGACAACG
AAGTGAGTCCACCATTTGTTTCAGGAGATATTTAGCAAATAGAAAGATATTTTGTTATAGTTA
TCTACATCCTTCTAATTTTGTTTGTTG

>AtbHLH095
ATGACTAATGCTCAAGAGTTGGGGCAAGAGGGTTTATGTGGGGCATATCCAATTCTGATGA
TTCCGGAGGTGGTTGCAAAAGAATCGAGAAAGAACCGCTTCCATCACATCCGTCTCATCCG
TCGCCAGAGATCCAGACTACGACGGTTAAAAAGGGAAAAAAGAGAACGAAAAGAAATGAT
AAAAACCATGAAGAAGAATCACCTGATCATGAAATACATATATGGACCGAAAGAGAAAGGA
GAAAGAAGATGAGGGACATGTTTTCTAAATTACATGCTTTGCTTCCCCAACTTCCTCCTAAG
GTATGATTCAATAATAAGAGTGTATGCTTTAATTGAAGGATTTTTTCTTATTTAGCATGAAATT
CATATATTAATTACCATCGTTTTTAACCGGTAATTTATATGCGAAAATTGTGTGTTTATAACAC
GATTATCTGTATGAATTACCAATCATATAAAATTACAGATATGCATATACATATAACTAAAAAA
AATCCAATTTCATCCTGAAAATAACATTTGCATTCAGAGAATGGTCTAGGCTTATCCAAATGC
AAAAGAATGACTTATTTTTGGTGAAACGACAGGTTATAAACTCATCAATTTTATTTAGATCTG
TAGATTTCTTCTTCTATTTTTTATACCGTCATTCTGTTTATTACCAATCAGAACAATTTTATAAC
ATAGTTTTTATATATGTTAAGTTAGCATAAGATTCTCTTATGTTTCATTCTTTTGCCGATTGACA
TGTTTTAAGATATCATGCTTACGCTTGTTCCTTTTCAGGTTTTGTTTCACATCTTTGCGCTTTT
AATTAATATAACTATGTCACTTAAATTTGGTGTGATTTGGCAAGGTCGCCATGCTTTCGATTT
CTCTTTACTTGCTTGTCTTTCTCACATTTCTTCTTCCTCTTTTCATAATTTTTTCTTGTGGGTGT
TATCAGTTATGTATATATTATAGTTAACTTAGTAAATTTTTATGAAATGTATATTTGATAATTTTC
TTATAGGATTATAGGATATAGATCTAATATAGATCTTAGCTAATACATCTAATTTATAAAAAAAA
CAGGAGTCCTATTTGAATCCACCGATTACACATGGTAAAAAGGCTCCGGTTTTAAAGCACTA
CATATAATAAATAATATATGAACTGATATGTACATTTATGTGTCGGATTTCTTACGTTATATAGA
ATCATATATTCTAATATTTTATGTATGATATGATCGACAGGCAGACAAATCAACAATTGTAGAT
GAAGCAGTGAGTTCAATCAAATCCCTTGAACAAACTTTGCAAAAACTTGAAATGCAAAAG
CTTGAGAAGCTTCAGTATTCTTCTGCCTCAACAAACACAACTCCTACTACAACTTTTGCTTA
TGCTCCATCATCATCCTCTTCTCCAACAGCTCTCCTCACACCAATATCAAACCATCCCATAGA
CGCCACCGCAACAGATTCTTACCCTCGCGCAGCTTTCTTGGCCGATCAGGTATCTTCTTCCA
GTGCCGCTGCTGCGAACCTGCCTTACCCTTGTAATGATCCGATCGTGAATTTCGATACTTGGT
CCTCACGTAACGTGGTGCTGACTATCTGTGGGAATGAAGCTTTCTTCAATTTGTGTGTCCCT
AAACACAAACCAGGGGTTTTCACTTCTGTTTGTTACTTGTTTGAGAAGTACAACATGGAGG
TTTTGTTTGCTAATGTCTCCTCTAATGTTTTCTGGAGCACCTACGTGATCCAAGCACAGGTAT
CTTATACATATAACCATTTTTATCATATGATATGTGGTGACATCTATTATTTACCATGGTTCTTTT
TTTTTATGTGATGAAACAGGTAAATCCGAGTTGTGAGAATCAGTTACTGGGAAATGGTCTTG
GAGTTGTAGACGTTTTCAAGCAAGTTTCTCAAGAACTGGTGTTATATTTTTCATCTCTATAAA
TCATAACTGGCTGCTTCGGTTTGAGTTTTAAGTATTTTCAGGGTTTGTTTTCTTTGCGAAGTC
CCAAGGCTTATGCAGAGAGATTAGCTTGGACTTTTCACTTTAAACCCCATCATCAGAAGGAA
AGAAAGAAAAAAAAAGGAATAAGAGAAGATATATAGCTTCTTCGGACATTGTTTTTTTCGC
AATTTTAATTTTATCTTCTTTATCAATGTATTGGCAAATGTTATCAAATGAGATATTTAAGTTC

>AtbHLH096
CTCACATGGTATACGGCAAAAAAATATTAAATTCATGGCAACGTATAAAGTTGATATTTCAAC
ATGTTATAATTTCTCTCTCTTCGAACATGAGTGTAACAAGACGAACCCCAAAGCCTGCAAAA
ACACACAGAATCTTTTCTTTGGTTTTGTGGACCGGTCTACAATCTCACGTGCAAAACCAAA
CATACACGATCTCTCTCTCTCTATATCTCTACTTTTTCTGAACTCAGTTGGTTTCGATTTTCTT
CGGACCTTCTATTGTTAAATCAGCTTTATAACCTCACAAGCTTCTCCTCTATTCCATAGATCA
AACCACAACTTTGTTTGACCTTTTTTTGAGTACTTCTCTTGTCTTTTCTTCTTCAAGTTTACTT
AGTTCTTTGAGAACAAATTTCTGTTGATTTTTTTTCTTTCTTGTAGAGGGATAATTACAAGTT
TAATTTCCCTAGAGTGTCTTTACCTTTTGCCTTGTCTCCCTCTCGAGACAAAAAGAGAAAGA
GAAACACTTTCTGTCCTCTTTTTTTTTCCTCTCTTTTTCTCTCTCGCTGGAAACCTTTTCATAT
TCAAGTGTAATTTTAATATTTCAAGCAATTTTCTAGCTAATTATGGCCTTGGAGGCTGTAGTTT
ACCCGCAAGATCCGTTCTCCTACATCTCTTGCAAAGATTTTCCGTTTTACGACTTATACTTTC
AAGAAGAAGAAGATCAAGATCCACAAGATACTAAGAACAACATTAAGCTAGGGCAAGGAC
AAGGACATGGTTTTGCGAGTAATAATTACAACGGTAGAACCGGAGATTATAGTGATGATTAT
AATTACAACGAAGAGGATCTCCAATGGCCACGAGACCTTCCTTATGGATCTGCCGTCGACA
CCGAGAGCCAGCCTCCACCGTCGGATGTGGCGGCGGGAGGAGGGAGGAGGAAAAGGAGG
AGGACGAGGAGTAGCAAGAACAAAGAAGAGATCGAGAACCAGAGGATGACTCACATCGC
CGTTGAAAGAAATCGCCGGAAACAGATGAACGAGTACCTCGCCGTTCTCCGGTCTCTCATG
CCACCTTATTATGCTCAAAGGGTTAGTCTCTACATACAACAGTTATATGTATATGTGAGTGTG
AGCGCGTGAAATATTCAGTCCTTTAGCTGTATTTCTTGACGTGTCAAAAAAAAAAAAAAAA
AGCAAAGCCTATGTTCTAGGCTTTCAGCTAATTAGGACATGCATAGGCTATACATAGATATGT
CATATGCATGTATGTTTATATGTTTTGAAATATAAATTTGAGAATGATGATAGATGAATGAAAG
CCGTTATATATATTGACAAAAAATAAAAACTAAACGAAAGTCGTTATTTCTTTTTTTCATAATT
TTATTAGATCATGTAGTCTCTAAATGAAAATTTTGTTAGTGGAATTGTGGGAGCATAAATAAA
GTCTTGAACATCATTGCACCCAAAAAGGACTATACATATTATAGATTTATAATGAGATGGTGA
CATAATATATTTTTTAAAAAATTAAGGTACGAAATATAGACATGTTTGATTGTTTTTGCAAAAC
CTTTTGCAATTTCTATGTGCTAGACTAACATAAGGTTGGTGGTGGTTAAAGGGAGATCAAGC
ATCGATAGTAGGCGGAGCCATTAACTACTTAAAGGAGCTTGAGCATCATTTACAATCAATGG
AACCTCCGGTTAAGACCGCCACAGAAGACACCGGAGCCGGCCACGACCAGACCAAAACA
ACCTCGGCTAGTTCGTCGGGACCATTCTCGGATTTCTTTGCATTTCCTCAGTACTCGAACCG
GCCTACCTCTGCGGCGGCGGCTGAGGGGATGGCGGAGATAGAGGTAACGATGGTAGAGAG
CCATGCGAGTCTGAAAATACTTGCGAAAAAGAGACCGAGACAGCTTCTTAAACTGGTCTCA
TCGATACAGAGCCTAAGGCTCACTCTTCTCCATCTAAACGTCACAACTCGAGACGACTCCG
TCCTCTATTCCATCAGCGTCAAGGTACATTTTTTTCTCTCTTTTTCACCATTTTTTGTTTTTTTT
TTCCTAACCAGATTAGAGTGATCATGTTATATAATACCGTTGTTTAGGGTTAGTGGGTTGAAT
TTGCAAATTCAAATTAAATTGGTATCTAATAATTAATGGGTGACTAATCATTACTCCCATTGTT
GGTTTTGGTTCAATGATTATTCATTGATTGTTTTTCCAATCACAAATGTTTTGTGCATTTAATG
TGTCTCTTATTAGTACATGTTGTGAATTATTTGTCTGGGCCAAAAAAGGTTTTCCAAGAACA
AAAATAAAAATCGTTCCTTTTTTCTCGTTCTTTTTTACTTGGTTAGGGCTGGCCTCAATTATT
GGGGGGTCAAAATCATCATTTCTTATTTATTAAAAAAGACAAAATTTTGAGTACTATCATAAT
ATTGATTCTTGTAAGAATCAGGGACCAAAAAGATGACATATGCCTACACATGTGTGAATGTG
TGTATATATATATGTGTACTGGGCATGTTTGGTGGTCACAACACTGTGGATGCACTGTGCACG
GCAGAACGAAATGACGCCACTTGTTTCAGGACACATTCATAAGCTTCTTCCTCGAAATATAA

ACATTTTCTTTAATCATCTTTTTACCTTTTCAATGACTATATATCAATCTTTTTCATGTAACTTA
AAAGAAAATATTAAAATAATGGGTTTATTCAGGTTGAAGAAGGGAGCCAATTGAATACAGTA
GAAGATATTGCAGCAGCTGTGAATCAAATCCTAAGGAGGATCGAAGAAGAGTCATCCTTTA
GCTAGTATTGTTGAATTTTAGTTATGGCACATTTAGAGGAGCTCTAATTTTTCATGTGTGATCT
TTTGCTCTCTCTTTTTCATATATTAAATAATATAAATTTCCTTTTTCTTTTTGTTAACGTTAATTC
TAATGCATTATATATTGGAGTTTTTGTGTGATTGACCTTTTAGACCTGATCACAAAAGAGGAA

>AtbHLH097
ATTTATGACTTCTCAATACAAAAAGCTCCCCTCACTTTTTTAAGTTTTGTCTTCTCTAATCCGT
CTTCTTCTACTATCTTGCATGTCTTGCGTCTTTTATATACATCTCTCGTAAACCCTAGCAAATC
ATACAAGGTCAGTCCCTTTTATTTTGTTAACTCCTTACTAAACAAAGCTCTAATATATTTGGT
GTTAATCATTTTTTTTAATTGTCAAAATTCAATTTGAAAACTTGTTTTGTACATATAAAATACA
TGTATATGTATATGGTGAAACATGTAATCTGCCTAGGTTTGGTTTTAACTTGTTCTACGTGTGT
TTTTGTTTCATGTATATGTTTTTAAAACTGACTGTGAAGCTCATGGATTGTTGTCATCAGGTC
AAGAAGCTTGACCTTCATTAGACTTAAGCAGTTTATAATCAACTACCACGAATAGCAATGGA
TAAAGATTACTCGGTACGTACGTTTTTTTTTCCAGTATTTCAAGATCCTTAATCTCTTTTCTTA
TACAAATTGATAGATATCTATGTATAAGATTCAAATCTTCCTGCTTATGATATTAACTTCATTCT
CCAATTCTGGTTTTTATTAGGCACCAAACTTCTTAGGTGAATCCTCAGGCGGTAACGATGAT
AACAGCTCTGGTATGATAGACTATATGTTCAATAGAAACCTTCAACAACAACAAAAGCAATC
GATGCCACAACAGCAGCAACATCAACTCTCTCCTTCCGGATTTGGAGCAACACCTTTTGATA
AAATGAACTTCTCTGATGTGATGCAGTTTGCGGACTTCGGTTCGAAACTTGCGTTGAACCA
GACCAGAAACCAAGACGATCAAGAAACCGGGATTGACCCCGTTTATTTCTTGAAGTTCCCT
GTCTTGAACGACAAAATAGAGGACCATAACCAAACCCAACATCTCATGCCTTCTCATCAGA
CGTCTCAAGAAGGAGGTGAGTGTGGAGGAAACATAGGCAATGTGTTTCTTGAAGAAAAAG
AAGATCAAGACGATGACAACGACAACAACTCCGTGCAACTACGTTTTATTGGAGGAGAAG
AAGAAGATAGGGAGAACAAGAATGTTACGAAAAAGGAGGTGAAGAGCAAGAGGAAGAGA
GCTAGAACGAGCAAGACCAGCGAAGAAGTGGAAAGCCAACGGATGACTCATATCGCGGTC
GAAAGAAACCGTAGGAAGCAAATGAATGAGCATCTTCGTGTCCTTAGATCTCTCATGCCTG
GCTCCTACGTTCAAAGGGTATGATATTCATAAATTATATCTTAAAGAAAGAATTTTCGTGAAT
GTTTTTTTATATGTATATGTACAACTAATTATACTGTCTACACCAAATATTAATTGTATAAAATA
GGGAGACCAAGCGTCAATCATAGGAGGAGCAATAGAGTTTGTGAGAGAGCTCGAGCAACT
CCTACAATGTCTTGAATCACAGAAGCGTCGAAGAATCTTAGGAGAAACCGGTAGGGACATG
ACAACGACAACGACTTCTTCTTCTTCTCCCATAACTACGGTAGCGAACCAAGCACAACCGC
TCATTATTACGGGAAATGTAACCGAGCTAGAGGGCGGAGGAGGGCTTCGGGAGGAGACTG
CGGAGAACAAGTCGTGCTTGGCTGACGTGGAGGTGAAGCTGCTAGGGTTTGACGCCATGA
TCAAGATACTTTCAAGAAGAAGGCCGGGACAGCTGATTAAGACTATAGCTGCTTTGGAGGA
TCTTCATCTCTCTATTCTTCACACTAACATCACTACCATGGAACAAACCGTCCTCTACTCCTT
TAATGTCAAGGCAAGTTTATATGTTAAAAAGTAATTCATCATTCATATTCAGCTTTCATATGCA
AAATCTAGACTCATCAAATAATACATGTAATTATCTAATCTGGATTAGCTGTCATAAAACACAT
TTATAATTTTATTTAGGGTTTTCAATATAATCATAGATTATGGATTTGTATTGTTAAAGTATATGT
AATAACTATCAATGGCTAACATGAGGTAGATGTAAAATATGTGCAGATAACAAGTGAAACGA
GGTTTACGGCAGAAGACATAGCAAGTTCCATCCAACAGATATTTAGTTTCATTCATGCAAAT
ACCAACATGTAAGGATATATATTTTTCTGCTATTTAAATATTCTTTCGATTAAAGATACTTTTGA
TAAGTTTCTGTTGGTCCCTTAAAACAAAAGTTTATGTTGGTTCATTGTTATCCATTTCACATG

CCTTTTGTGTTTTGTTTGTTTTTACCTTTTAGATGTCATGATTATATCTTTTTGTGTGCAATGTG
GTCTATCTAATCATCAAGACATCATTACTATGTGTGTGTGTGACTCTGTGAGTGTGTGAGTAT
ATGAGTCTGGTAAAAAGTCTTAGAGTAAAAGATATTGGTGGTTCGATGTTATACATTTTACTA
TTTTAGATGTCTAGCTTTTGTCTTTTGTTTGTTCTTTTACCTTTTAGATGTTATGATTATTTGCT
TTTAATGTTAGATATATATGGTGTATCTAATCTATCATGGTTATTTCTTTGTGTGTTACATATGG
TGTGTATATATATATATATATATATATTGTAACATATTTATCACAATATTTGAAGTGTTAAAGCTTGG
AAACAATTTTATTTCAGATCTGGAAGCTCTAACCTGGGAAATATTGTGTTTACTTGAAAATCA
TCACACGGCGACAACTTTGTACACTGGTGAAGATTACAGTACGTAATAATCTCTACATATTG
GGTTTTATTCTCCAAGCATTTGGAAGAGTGTTTAAGTTAAAGGGAGTGCTTACTTTATTTTTT
TGGGGCTTTTTTCATGCAATTTAAATTTTAGTGATGATTGTGTCGCTTGTAATGTTAGAACTC
GTTGTTGTGATTTCTGCTGCTTTGATTTGTAGGTTTTGAACAAGCGGTTTAGAATGCTAAAC
CACTTATTTACTTGAAATAACTTTTTTCACAAAATAAAAAAGAAAAGAAAAAAACAATGCAT
GAAAACTTTTCTTGAGGGGAGGGAGAGGAACTATCATTTTCTTGAGATTTGTCTATGAGAAA
TGGGCCAATATATATACAACAAGAAAA

>AtbHLH098
ACACGAAAAACCTAGATCCTCCCCCAAATTTCATCCAACGGCACAAACCGCATAATAAATA
ACCCTATCAAAATATAATAATAAAAATTGAAATTTGATAGCGATAGTTTAGTCCTCGATGAAT
GCGAGTCCCTCATATTTACTCCTTTTGGCATTCACTCTCTTTATTTCTCTTTCTCAACTTGATA
TGTCTTTTGGCGTCTCTTTCTTTCCCTAAATCTTTCTTTTATTTTGCTGTTTAAAAAAAAAATC
CAACCATAAGACAAAACAACGAACGAGGAAGAGAGAGAGAGAAGGATATATCTCTAATCA
CGATGCAGGAGATAATACCGGATTTTCTTGAAGAGTGTGAATTTGTCGACACTTCACTAGCC
GGAGATGATCTATTTGCCATCTTAGAGAGTCTTGAAGGTGCCGGAGAGATATCTCCGACAGC
TGCATCTACACCTAAAGATGGAACCACAAGTTCCAAGGAGTTAGTTAAGGATCAAGATTATG
AAAACTCATCTCCTAAGAGGAAAAAGCAAAGACTAGAAACCAGGAAAGAAGAGGACGAA
GAAGAAGAAGACGGAGACGGAGAAGCAGAAGAAGATAATAAGCAAGATGGGCAACAAAA
GATGTCTCATGTAACCGTGGAACGTAACCGGAGAAAGCAAATGAACGAGCACTTAACCGTT
TTGCGTTCTCTTATGCCTTGTTTCTACGTCAAACGGGTATATTCAATACATCCATAGATTTTAC
TAGTCAGTATGTGAGTTTCTGGTAGTGCCCGACTAATTAATACTATTCGTTCAACTAATCTTTT
TGGTATACCTAACTATCATATTCATATATCATTTCTTAGTAACATCAAAAGTATCATGCATATCT
ATGTTTTTTTAGGAATAATGACAATTATAATGACATTTTGGTCCCACTAATCATCTCTTTTTCT
TTTCTTTCTTTCATGTCACAATGAGATATATAGAATGTTCATATTGACTTATATTTTCTTTATTT
CCTCTTAAACCAACCTATTAAAAAAAGGGGGACCAAGCATCGATCATAGGAGGAGTTGTGG
AGTACATAAGCGAGTTACAACAAGTTCTCCAATCTTTGGAAGCCAAGAAACAACGTAAAAC
CTACGCCGAAGTCCTAAGCCCGAGAGTTGTCCCGAGCCCTCGTCCTTCACCGCCTGTTCTA
AGCCCAAGAAAACCGCCTCTTAGCCCGCGCATCAACCACCACCAGATTCACCACCACCTAC
TTCTCCCTCCCATAAGTCCTCGAACACCTCAGCCAACAAGCCCATACCGGGCCATTCCACCG
CAACTACCACTCATCCCACAGCCTCCGCTTCGCTCTTACAGCTCATTGGCCAGTTGCAGCAG
CTTAGGAGATCCACCTCCATACTCTCCTGCTTCATCTTCTTCATCTCCTTCAGTTAGTAGTAA
CCATGAGAGTAGTGTGATCAATGAGCTTGTTGCTAACTCAAAATCGGCTTTGGCTGATGTGG
AAGTGAAGTTTTCAGGAGCTAACGTGCTGCTCAAAACGGTGTCGCATAAGATCCCGGGACA
AGTTATGAAGATAATTGCTGCTCTTGAAGATTTGGCTCTTGAGATTCTTCAGGTTAATATTAA
CACCGTCGACGAAACCATGCTTAATTCTTTCACCATCAAGGTACGTACGAATAATCATCCTAA
TTAATTTTCACTGACTTGTTTTTTTTGTCTCTATTCATAAGTGTAATTCATACATTTATTTTCTT

ACATAGGATATATGGTGGGGGAAGAAATGGGTAGGGTAGTGTAATAAGTAATAATTCATTGA
GATTCACATAAACTCATACACACTTTCGATATATATGAAATTTAATGTATGATTCTAATCTACAT
TATATATATATAGATAGATATATATATCATTATATAAAACGTAGGGTATGTGTATGTCAGATTCCT
CACAAACATACAAAACTAAACTGTTCGTTGTTTTATACTACTTTTACTAAAACTCTTCTCTAA
TTTATACATTTGACTTTCTTAAAAAAAACGGTGTGAAAGAACACGTGTTTAAATATTATATAA
ATTATACGTGAACATTGAAGAGCCCCCAAAATCTTCAATTTTAAATTCGAAAGGAATTATTCT
ATATATTTGGAAATTATTCAAATAGATCAAGCTGCTGAAGTTTATAAGTGTCAAGACTTAGTT
TCTATTTTTGATAAGTACTTAAATTTGTTCAATGAAGAATTTCTACAATGGATGTTTTAGTATT
GATGATTTGATTATCGAAAAAAACAAGATCAGAGATCAAGTAGGGATACTATTAACTATGTC
TCGCAAGTATAGATATTTGGTAATTAAGTTCCTTAGAATAGGTGATTATGTTGAGACGAGAAC
AGATCTTTTTTTTTTTTAATAATAGTTAAATTTGTTTCTTTTTCGTTGGGAGTTTAGTGCCTCG
AGAAATATAGAGCTACTCAGAATGAAATTAATTGAAAACACTTATTTTTGTATAAATTATATTT
TTTTTCATATCATCTTTTTTTTTTTTGTTAAAGAACAATTATCAATGTATACTTCTCTATCTTTTT
TTCTTTTGATAGATTGGAATTGAGTGCCAACTAAGTGCAGAAGAACTGGCTCAACAAATTC
AGCAAACATTCTGCTAGTAAAGAAGGATTTAATATAGCTTCGTATAAACCTTAACGAGAGAG
CAGTACGTACTCACTTTCTCTCCTTAGTATCCCTTTAATTATCTTTTCAGTTTTCTGCAAAGAT
ATGGAGTTTAAAAAAATAAAATTGTTATCTAAAGTTTTAATCAAATATTGATTAATTATAACTA
ATATAGGTATAAGTGAGTTTTAAAGATTATCAGCTTCATAACAGCCATCGTCATGTTTACTTTC
TTTTAAATTTTAGAATTTAGACGTACTCCTACCATGTAATTTTATTTCTGTCATTACATCAAGC
ATTGTAGCTGTAATTGCATATGAATGAACAATAGTGTATGAGTGATCTCATGAATAATATTCTT
CTTGCAACACAAAATGTTAAGG

>AtbHLH099
AAAAGATAACGTAGCCATTAACTTTTATGCATTAACTCCTTCATTTCTTTTTGTGCTCGTTTG
GTTGAGAGAGAGAGAGGCTATATATGATGTTCCAACAAGATTACCCTCATGGCTTTTCACTC
GTGGAAACATCCTTAAGTTACGAAATGTTGGATTACTTTCAAAACATCGTCGTTTCGAACTC
TGAAGACGTGGCGTCACAGCAAAATTCCATTTCGTCCTCTTCTTATTCATCAGCGACACTCT
CCTGCTCCATAACAGAGCAAAAATCTCACTTAACTGAAAAGTTATCTCCTCTACGAGAAAGA
TATGGTTGCGGTGACTTTCTGTCGCGGAAGAGGAGAAGGAGAAGTGAAAAAACGATTGTA
GATAAAGAGAATCAAAGGATGAATCACATTGCCGTCGAGCGTAACCGGAGAAAACAGATG
AATCATTTTCTGTCTATCCTCAAGTCTATGATGCCTCTCTCTTATTCTCAACCTGTGAGTTTCT
TTCTATCTTTAATTTAGCTTATAGGATATAAGCAGGCTAAGCAATGTTGATAATTTGATATGTT
CGGTTAAAATTTAGTTTAGACAAAGAGTGTTAAGGTTCCTTATTAAAAGTAAGCCGTTAATT
AAATAATAGTAATTTAGTATATTTGTTTTTGAATCAATTACTAAATAAAATTGCGAATTGATAA
ATTTTTGGGTTTTTCCTATAATAGAATGACCAAGCATCAATCATAGAAGGGACCATTAGCTAT
CTGAAGAAGCTAGAACAACGTCTCCAATCTCTCGAAGCCCAATTAAAAGCTACTAAACTCA
ATCAATCACCAAATATATTTTCCGACTTCTTCATGTTCCCTCAATACTCCACCGCCACTGCCA
CTGCCACCGCCACTGCCTCCTCATCCTCCTCGAGCCACCACCATCACAAGCGACTAGAGGT
GGTTGCTGACGTGGAGGTTACAATGGTAGAAAGACATGCCAACATTAAAGTGTTAACGAAG
ACACAGCCAAGATTGCTCTTCAAGATTATCAATGAGTTTAACTCTTTAGGTTTAAGTACTCTT
CATCTCAACCTCACAACTTCCAAAGACATGTCTCTCTTCACTTTTAGCGTCAAGGTAATCTAT
TCTCTTCCTCTCTTGTTTTAGGGGAAAAAGATAATATATATGATTATAGAAGTTGTTAGTTAAC
ATAAATTTATATGAAATAACTAAAATAAGGCTTCTTGTTTTAAAAGTCAACTATATATGCTCAA
AATGATTGGTCTCTGTATTAGGTAGAGGCAGATTGTCAATTGACGCCTTCTGGTAATGAGGT

CGCAAATACGGTGCATGAAGTCGTTAGAAGAGTTCACAAGGAACGTTGAATTTTGTTTACAT
ACTAGCTAACTTTGAAATTCTATTTTATTGTATAAACAATCTCTTATGTGTGTAATTTACATATA
TACAATTAATTAACATTAAAGTTGTTTAGTTTTTTTTTTTTTTTTGTTGGTTTACTGAGTTAAT
AACATTGTGTTGACGTCTATTAAATTGAGGATATAAAATACAAGCATGATGAATTGA

>AtbHLH100
TGTGTGTATCAGTGTATATATAAACAAAAATGAGTGTCCACATAAATAATCCAACTAACACAT
CAGCATAATCCCAACCGAAACAGCTTCTAGAGAGAGACAAAAAAGAACAGAGTAACTATCT
AAAACTCAAAATGTGTGCACTTGTCCCTCCATTATATCCCAATTTCGGCTGGCCTTGCGGAG
ATCATAGCTTCTATGAAACCGACGACGTATCCAACACGTTTCTTGATTTTCCGTTGCCGGACT
TGACGGTGACTCATGAGAATGTGTCGTCTGAGAATAACAGAACATTACTAGACAATCCCGT
GGTGATGAAGAAGCTTAATCACAACGCGAGTGAACGTGAGCGTCGCAAGAAGATCAACAC
AATGTTCTCATCTCTTCGTTCTTGTCTTCCTCCCACCAATCAAACGGTAACTGTTAATTCTATA
TACTTTTCTATTTCTAAAATTATGAAAAAATTTGAAAGTTTAGTTTAATATTCAACAGAAACA
AAAAAATATCAAATGTTTTCTTGCAAATGCTAAAAGTTGTTATTCATTGCTTTTGCAATTTTG
GTGTTGATACAAACAGTGATATGTTTTGTTTTTTCTAACGCAAAGAAGGAAAAAAAAATCG
GTCCCAAGTAACATAAACTGAAAGATCTGTCCTATTTTTTATGTATTTTCTTGCAGAAGAAGT
TAAGTGTTTCGGCAACAGTTTCACAAGCATTGAAGTACATACCAGAGCTGCAAGAGCAAGT
TAAAAAGCTCATGAAGAAGAAAGAAGAGCTCTCGTTTCAAATTTCGGGTCAAAGAGATCTC
GTTTACACCGACCAAAACAGTAAGTCAGAGGAAGGGGTTACAAGCTATGCGTCGACAGTTT
CTTCGACTAGGCTCAGTGAGACTGAAGTGATGGTCCAAATTTCATCGTTACAGACTGAAAA
ATGTTCGTTTGGGAATGTCTTGAGTGGTGTAGAAGAAGATGGGTTGGTTCTTGTGGGTGCTT
CATCTTCAAGGTCTCATGGAGAGCGACTCTTTTACTCTATGCATCTTCAGATAAAAAATGGC
CAGGTGAATTCCGAAGAATTAGGTGATAGATTGTTGTACTTGTACGAGAAATGTGGACACTC
GTTTACATGATTTAATAATTAACGATGCTATATATATTTTTTCTGTTTTGTCTCTCGTTAAAATT
CGAGGTTCTTTTTAGAAATAGGTTTTTTTTTTGTATATTAATTTTAATATATTATATTATTTTGGC
GAAATGAAAGTTTCACGGGCAAACGTTTTGGTACCGATTTGGAAGGAGAACAACGCGAAC
ATGGGTTGTGTGCAACAGTGTAAAGGTTTATATATCTGTGTATATTTTATGAGGATTTGTCATC
TCACATCTGTGGTTACACACCGATGTCATATGTGGACACTAATTATCTG

>AtbHLH101
CTCAAATTCGGTCTTATCATCTTTTTTCCTACTTCATCCCATCAAAGTCTCTCTAGCTATATATT
CTTCTCACACATAACATAAGTGGACAAAAAAAATGTGTACCTTAACGCCAATGTTTCCAAGT
AAGCAGCAAGAATGGTACTCTGCTTCAACAATGGAGTATCCATGGCTGCAGTCTCAAGTTC
ATTCCTTTTCACCTACTCTCCATTTTCCTTCCTTCCTTCATCCTTTAGATGATTCCAAGAGCCA
TAACATCAATCTTCATCATATGAGTCTTAGTCACAGCAATAATACTAACAGTAACAATAACAA
TTATCAAGAAGAAGATCGAGGAGCGGTGGTTTTGGAGAAGAAACTGAATCACAACGCAAG
CGAACGAGACCGCCGTAGAAAACTTAACGCCTTGTACTCTTCACTTCGTGCTCTCTTGCCTC
TTTCTGATCAAAAGGTTTGTGCTTTTAAACCTAATCTAAAATATCGGATCAAGATTAGGTTTT
GTAATCTTGAAATGGATATATGAATACTAAAGAAACCCTTTTGATCTGCAGAGGAAGCTGAG
CATTCCTATGACGGTAGCGAGAGTAGTGAAATACATACCAGAGCAGAAGCAAGAACTTCAA
CGTTTGTCTCGGAGAAAAGAAGAGCTCTTGAAGAGGATCTCGAGAAAAACTCACCAAGAG
CAGCTGAGAAACAAAGCAATGATGGACTCAATAGATTCTTCTTCCTCTCAACGGATCGCAG
CAAATTGGCTCACTGACACAGAGATTGCTGTCCAGATTGCTACGTCGAAATGGACATCTGTT

TCAGACATGTTGCTTAGGTTAGAAGAAAACGGGCTTAATGTCATAAGCGTCTCTTCTTCCGT
TTCTTCCACCGCAAGGATCTTCTACACTCTACATCTTCAGGTACTTGTCTAAAGATATCAGAA
GGTCATGTTCTTCTTACATGGAACTCATTAAGAGAAACTAAAATCTATTGTTTATTCTTCTTT
TTGTGCAGATGAGAGGAGATTGCAAAGTGAGACTGGAGGAACTCATCAATGGTATGCTCTT
GGGATTACGCCAATCATAATGAGCGATATGAAGTGTTTAAAAGAGTGAATGTTGTTAGTTTAT
GTTAGACTTTTATCAAATCTGTAACATTTTTTTCTTCTTCTGATTAATGATTATTCAACTGGAA
AACTTATTCAATAAAGAGAAATTGCAATTTTTTATGAGATA

>AtbHLH102
CGAAACTGACGGACAAAAGCTCAAGTAAACTCACGTAGTCTCTCCCACAGCTAACTAGAA
CTCAGCCGAGTCACGAGGCACCCAAAAAACAAAAAACAAATAAGCAACAAAAAAGAGCT
CGTCGACGGCAAAAGTTGAAAATAAAACAATAAACAGTCGAAATTCTCCGGCGAAGATGA
GAACCGGAAAAGGAAATCAAGAGGAAGAAGATTACGGAGAAGAAGACTTTAACTCTAAAC
GTGAAGGTCCTTCTTCCAACACTACTGTTCATAGCAACAGAGGTAAATTGTTCGATTTGGGA
GACTTTTGAATTTTTCTTAGTTGTTGTTTCGTCAATTTGTGTTTTGGTTGGAGCTATTGTTGAT
ATGTATCGAGATGGATTTGAATAGTTTGGGATTAGATTTTTCTAAGTTTTGTCTCTGTGTGATT
CGGTGTTTGATTTTGAAAGCAGATTCAAAGGAAAATGATAAAGCTAGTGCGATACGTTCTAA
ACATTCGGTCACTGAGCAACGTAGAAGAAGCAAAATCAATGAAAGGTATGTTGATATAAAG
TGAGGATTTCAAACTTTTTGGATTGTTGTTTTTGTGAGAACTTGAGATTAGGAGTAAACGCG
ACATTCTTTGTTTTGATATCTGAATCAAAATTCAATTACTTTGCTAACAAAATCATAAGAATA
CTTAGGACTTGTGTTTGGGAATTTTGCTCTGCTTCAGTTTTGTGGTGTTTGATATAGGACTTA
GTTACATTATAATTATTGTTCTTGTTCTGAGTGCTGAATTCATAAATCTCTTCTCAGATTTCAG
ATATTGAGAGAACTAATTCCCAACAGTGAACAAAAACGTGACACTGCTTCATTCCTTTTAGA
GGTATGTCATGGTATTTGATGATGCTTTGTGCGAATATCCTGTCTTTTAGATGTTGTTAGCTTA
ATACCGCTATTCCATTCTAGGTGATAGATTATGTTCAGTATTTACAAGAGAAGGTGCAAAAGT
ATGAAGGATCGTATCCAGGTTGGAGTCAGGAACCAACAAAGTTGACGCCATGGGTAAAGTT
TTGAGCTTCTTCTTATGTCGGTTTCTCAATGAGTTTCTGTGATTGTAGATTCTGAGTATGTTTT
TGCACCCGCAAGGCTTTCTTTGTGTGACTTTTAGCTGAAGGTTATGTTTTCTTGATTGGAAC
AGAGGAATAATCATTGGCGAGTTCAAAGTTTGGGGAACCACCCAGTAGCTATAAATAATGGA
TCTGGTCCAGGGATACCTTTTCCTGGAAAGTTTGAAGACAACACTGTGACTTCGACACCTG
CAATTATTGCAGAACCGCAAATCCCGATTGAATCTGACAAAGCAAGAGCTATAACCGGCATA
TCGATTGAAAGTCAACCTGAGTTAGACGACAAAGGATTACCACCACTACAACCAATTCTTC
CAATGGTACAAGGCGAACAAGCTAATGAATGTCCTGCAACTAGTGATGGGCTAGGCCAAAG
TAATGACCTGGTTATTGAAGGTGGAACCATTAGCATCTCTAGTGCCTACTCGCATGAGTGAG
TCTGATCTTCTAAGTCTTCCTCGTTTCTGTTACTGTGCTGCCATTGATGTGTTCGCTTCTACCC
TTTTTTTTTAACTGCATTGTGTACTAATCTTGTGAGACATTAATTCTGAAATGGCCAGGTTATT
GAGCTCATTAACACAAGCACTCCAGAATGCAGGCATTGATCTGTCGCAAGCAAAGCTTTCT
GTGCAGATCGATCTTGGGAAGCGAGCCAATCAAGGTCTAACTCATGAAGAGCCATCAAGTA
AGGTAAATAGTTGGTTCGAAATGCTTCATACTTAGTTTTGTAACAATAGCCATATATCATGAT
GTTGATACATTTTTCTATACAGAATCCATTATCATATGATACGCAAGGAAGGGATTCAAGCGT
TGAGGAAGAATCTGAACATTCTCACAAGCGGATGAAAACACTGTGACATTTCTCTCTCAGG
CTCCACGGCGCTGGAATATATAAATGTATTGACAGATTCCTTGTATCGTGTACATTGTTCGGC
TTCTAGGGGTTCCTATAGTACTCGTTAAGATAAACTTTGGTTAGTAGATTCTGCGTTTTTTTG
TGGTTCTGAGTTTGTATCATCTTCTGCATCATTCATGTAACCTATCAAATATGATCTCTTGTGG

TTTCAAGTTATTGCATCATTTTTTTCCGATTTGATCTTCCTTTTTTGTTGAGAGAGCGAGATC
AAAGGCTAGCAATCTCTTAACCTTAAAGTGAAAGACTATTATAAAATCATTTGATGCTAAAC
CAAAGATGATGTGATGCTTTTATCTATTGTTGTGATGACCCATAACTTTGAGAAGAAAAGCAT
AATCAAAGGCAGTCTCTTCGCATTGAGCATAGCGACCACCTTCACCAAAATGCCCTCCGTTC
ATATTTGTTTTCAGAATCACAGCTCGGGAGCAGTCGTGGCACGTACTATCCCTTATCTTAGCC
ACCCATTTTGCACCTTCCCATACTCCAACCCTGACCCAATAACACACGCAAACTATGATCCA
AAGATTCTTACTTAAAACAAAAAAGAGATATGGGTTTAAGGGTTCTTTTGTGAAGCTAGCTT
GCCTTGAGTCATGAAACGAAGTAG

>AtbHLH103
AATACAATCCCCATCATCAACATCATTACTGTCTCATGCAAACTCTGACACTTACGAAGAAAT
AAGTCTACACAAACTAAACCATTGTCTACGTAACCAACAAAACATGACAGAAGAATTCGAC
ACTACGGGAGTTTGTACCGGAACATGGTGGAGCTCCTCCAACGGCATGTTCTCAGGCTGTT
CTCTGCCACGTTCCGCGGAGATTGTTGTCGATTTTGGTGAGATTGAATGGCAAAACATTGAT
ACCCTTGATGCCAAAACTTACAACGAGAACTACCTTAGTACTTCCACATTCTTAGGCAATGC
AAATCTTGATACCACATCACAAATCTACGTTTCGAGTCCATCTAACATGTAAGTCCATTTATC
ATCTTACGTGACGTATAACGTAAAATCATGATCTTTGTTCCTGAATCCTGACATTCTTTTTCTT
TTCTCTTTTTTACCAGCCATGAAGAAGAACGATACAATCAGATAAATAGTTTTCTAGAAGGA
TTGTTTGATTCTAGTGAGCAATTATTGGTTCCTAACTGTCCCAAACCAGAACTATTCGAAAG
CTTTCACTTCTTTGATGACGTTTTCCCGAACGAATCAAGAATGATATCAGTATTTGATCACCA
AAAACCCAAGGAAGACATGCAAGCTTGCAAGAGTCTGACTACATGCAAGGTGGGCAAATC
TATTTAATTAGTTTCTTTTAATAGAGATATTTTACAATATTTCTGTAGATTTTGACATTGTGACC
AATTATTTTTAGAGAGCAAGTGAGAAAAGTGGAGAACTCGAAGATATTGAGTCATCACAGC
CATTAAAAAGGCCAAGACTTGAGACACCATCTCATTTTCCGAGCTTCAAAGTTAGTAACAA
ATGATCTTACTACAGTTTTCAAGGATTGTCATTTATCACTAAAAAAAGTTAGTTATGCTAAGA
AACACATACCCATGCACAAACTATTGCTGATATTTTTCCAAAAACTATTTTGATTGATAAGGT
TCGTAAAGAGAAACTCGGAGATCGAATTACCGCGCTTCAACAGTTAGTTTCACCCTTTGGC
AAGGTTTGTGTTCATCATGAATTCATGATCTGTTATATTGATATCTCTACTATATATTATCCATA
CATAATTACGAAATTTTTTTAGCTACTTCTGTTTTCAAATTTATTTATTATTATTTTGTGATGAT
TTTTACAGACAGACACTGCTTCTGTCCTCCATGATGCTATAGATTACATCAAGTTTCTACAAG
AACAAATCACTGTAAGTTGATAATCTCTCAAATATGAAATACTACTACTAATTAATTGTTGGT
TTTAGTTAACTGTTATATTTATTCTTTTTATAATCATTTAAATAGGAAAAGGTCTCTACTAGTCC
ACACCTGAACTCTATAGGATCTGGTGAACAGAAGCAGGTACTTAACTTAGTTAACGGTTAA
ACAACTCTATAATCTAAAATCAATTAAGTAGTTTGATTTTTGTTCTTTTGCTTATGATCATTAAT
AAGATTCTTTCAATTTCTAGTGGTCAGACAAATCAAGCAATAATACTCATAATCAAAATTGTA
GTCCAAGACAAGATCTTCGAAGCCGCGGTCTTTGTCTGATGCCGATCTCAAGTACTTTCTCC
ACTCCTCCGCAGCATTTGGACACATCTAGTTTGTGGAATTAGACTCTTATACTGAATCAAAA
ATGTAACATTAGTACATTACACATGAAAACTCATAGATTAGTTTTGTTCTAAGAACTGTAAAG
TCTGGTGTTAATTTGTGTATAAAATGTGATGTCTCGTTCCATTACTTCCTACAAATTTAGAGC
CTACTTCGTGGAATGGCCGAAAGATTAGATAATTTGTGTTTCTTTTTTTG

>AtbHLH104
TGAGATTGTAATCCGCCATATACTAACTCGATAACCCTGACTTTTTAGAAATCTAAAAGTATA
ACCCTATAATAAAATAGATTATGTCCAACTCGCGGATTCTAAACCTGAATTGACATGGCGGA

ACCATTCATAAAACCATACCAATTCTTGCGTTTTGGAAAGTTGGTTTCCTTTTCATTTCTTTG
AATTTGATCCTAGATTGTGTAGTTCTCAAGCTCATGCTCTTGCTCGTCAAAAACGTATCAAC
GATTTGCTTTTCCTTTAGACGTTCTTGCATCGGATTGGTTTACTCAACTCCACTCGATGAAAA
AGTTACTATCTTAAAAAGAACAAATGCAAATTCAATAAAATTCCCCAATATCTACGCATGAA
AATATGATTCTGTCTTTTTTTTGTTTGTCTATCTTTCAATACTTTAAAAAGTGTATTTCTAGAG
GCGATTTGTCATTTCCCGAATAGGAATTTGAGTAAAAAAAAACACTCTGTTTTTATTTCCAC
TAATATCAAATCGGCGAATATAGTTCCCACCATTCTTATCTCCTTGTCGTCACTCGGAATTTTT
TAAATAATAGGATCGCTTTAATAATATAATCTCTATAATTTATAGTTCCTTTTCCAATATTGGCG
TCTCCGTTGAAGCTTTCCACAAATCTCAAATTTGTTTGGAGACTCTCAAATGTATCCTTCTCT
CGACGATGATTTCGTCTCTGATTTGTTTTGCTTCGATCAAAGGTTCGTCCTTTCGCACTCTTC
TCTTCCTTCACCTTCCTCTGTTTTCTCTATCTCTTTCGATTCAGTTGTTTTATCGAATTTGATCC
CTTTTTTCAATTGCAAAATCATATCTTTAATGTTCAATTCGATTTCTGTGAAAAGTGTTGTTGT
TTTTTTCGCATTTCAGGGGGAAAGGTTGTGTCTTTTGATGATAATTTGACGAACCCTAATGTA
TCTGAAAGCTGTTAAATTGAATAGGAGAAGAAGTAGCTTTATGGATGTTGTTGTTTATATTTA
CTCCTATTGATGGATGTGAATCTTTTTGGTCATGATGACTCTTGTAGCAATGGAGCAGAACTT
GATGATTACACACAGTTTGGTGTAAATTTGCAGACTGATCAAGAGGATACCTTTCCAGATTT
TGTGTCATATGGTGTGAATTTGCAGCAGGAGCCAGATGAAGTCTTTAGTATTGGAGCTTCTC
AATTGGATTTGTCCTCGTATAATGGAGTTTTGTCGCTAGAGCCAGAACAGGTGGGGCAACA
AGATTGTGAAGTTGTGCAGGAAGAAGAAGTAGAGATCAATTCTGGTTCATCTGGTGGAGCT
GTTAAGGAAGAACAGGAACATTTAGATGACGATTGCTCCAGAAAGCGGTGAGTTTTGAAA
AAATATAAACTTTTTGTGATTTGGTGGTAGTTGGTTGTGTTTTTAATTGAATCTGTGATGGTT
GTTGATAGGGCAAGGACTGGATCGTGTAGCAGAGGAGGAGGAACTAAAGCGTGTCGTGAA
AGGTTGAGGAGGGAGAAGCTAAATGAGAGGTATGTTTAGAGCTTAGTCTATGTTTTTGTTTT
GTGAAGTGAGTTGTCTCAAGGGGAAAAAAATTGTTGTTGTTCTGTTAGGTTTATGGATTTGA
GCTCGGTTTTGGAGCCTGGGAGGACTCCTAAGACTGATAAACCGGCTATACTCGATGATGCA
ATCCGTATATTGAATCAACTTAGAGATGAAGCTCTTAAGCTTGAAGAAACTAACCAGAAGCT
TTTAGAGGAGATCAAGAGTCTCAAGGTTGGTTGTTCATTCACTTTCACCTAAACCAAGAATC
TTGTTCAATGGGATGTGAACAGATTTCTAAGTTTGTATGTTTCTTTATAGGCGGAGAAGAAC
GAGCTGAGGGAGGAAAAGCTGGTGTTGAAGGCGGATAAAGAGAAGACAGAACAACAGTT
AAAGTCTATGACGGCTCCATCTTCAGGGTTCATACCTCATATTCCAGCTGCATTTAACCACAA
CAAAATGGCTGTTTATCCAAGTTACGGTTACATGCCAATGTGGCATTATATGCCTCAATCCGT
TCGTGACACATCTCGTGATCAAGAACTCAGGCCTCCTGCTGCTTAAACTCTCAATTGTTTTT
TTTTGGTACCCATAGGGAACTCATAGAGATTTCTGAATCACTTGGTTTAGGATTCTTCCGTTT
GGCAAGTGTAAAAGCTTTACTCTTGATCTTGGGTTGTATTTTTCTTATAAAATAAATATAACT
CCAGAAACTTGTGATTCTTCTC

>AtbHLH105
CGTAAAATGAATTAAAAATAAACTAACAAATAAAATAAAAACCCTTGGTATGATGGGCCTTG
GAAAGCCCAAAAAAAAAGGAAGAGTGCGCGTGAAAACCACTCTAATTTCTCTGGATTCCA
AGATTCCCCAATTCGTCTCCTCCAACAGTTTTCTTCTTCTCTTCTTCTTTGGGTGTTCCTTCC
ACCAACGGCAGAAATCGATTTGGCTTAAATCTCCCCCTCCTTTCGATCTCTCTGATCGCCGC
CGGGAACATTCAATTTCCCGGGAGTTCAACAAAAAAAAAACTCTCCGTTTTTATTTTTCCCC
CTTTTTCACCGGTGGAAGTTTCCGGAGATGGTGTCACCCGAAAACGCTAATTGGATTTGTG
ACTTGATCGATGCTGATTACGGAAGTTTCACAATCCAAGGTCCTGGTTTCTCTTGGCCTGTT

CAGCAACCTATTGGTGTTTCTTCTAACTCCAGGTTTTTCTTCTTACTTCCCCTTTCTCTCTTAA
GTGCTTCAAATTAGGGTTCCGATCTGGTTTTTAAGAAATTCAAATTGATTTGAGTGAAATCA
GTGTGATTGCTTGAATAATTTTATGTGGGTTTTTTCTGGTATGCGAAGGTGTATCGTTAAATC
AAGGGAAGGGCTAATTTTGTTAAAGTAACGAACTTTGTGATTTTAAGCTTGTGTGATTGTTT
GAGTAAGTTATAGAGAAGAAACTGAGTTCTTATTCATTTGAATTGGTTTCTATGCTTAAATTT
GGGTTCTTTTAGCTTGTGTAGATATCTTTAGAGTGGGTGAAATGATATGTTAAGTTAGGAAGT
TTGGCCTCAGGAGAGGATTTAGGTTTGTTTATCTAAAGTCAACTGTGTGTGCCTGACATGTT
TTTAGTGTAACTTCTGACATGTTACTTGATTCGGGAATTGATTGATCATATATCACTTCTTGTT
AGGTGGGAATTAGATTCCTTTTTCATGGATAGTGCTTTCACATATGAGTGGATGGTTTGACCT
TAGCATGTCAATGTTAACTAATTAATCTGCTTCCTTGGAAGTGAGTAGTTGTAAGTTGCTACA
TGATTGAGTATAGATGTAATACTTAGTTGGTTCGATGTATTTCTTGTTGGTTCAGTGCTGGAG
TTGATGGCTCGGCTGGAAACTCAGAAGCTAGCAAAGAACCTGGATCCAAAAAGAGGTAAG
CTTTTTATGTTAAACTCTTTGTGACACACCTTTAATTCAAAAGACTTACTGAACCAGGAAAC
TATAGCAACTTTCAGAATCTATAACTTTGAGAAATCGCTATGGAATTGTTTATGGTTCTTTTGT
TGTGGGGATCTGTATTGGCTCGCTTGACATATTTTGGGCTTGACAAGGTATCAGAAAAAATA
ATCTTGTTCATTCCATGTCTCAGGGGGAGATGTGAATCATCCTCTGCCACTAGCTCGAAAGC
ATGTAGAGAGAAGCAGCGACGGGACAGGTTGAATGACAAGTAAGTTTTAACAATTTTGTAT
ATCCTTCCTTTACTCTTTAGTAGGTACTAAAATGATCGCCTTTTAAATAAATAACAGGTTTATG
GAATTGGGTGCAATTTTGGAGCCTGGAAATCCTCCCAAAACAGACAAGGCTGCTATCTTGG
TTGATGCTGTCCGCATGGTGACACAGCTACGGGGCGAGGCCCAGAAGCTGAAGGACTCCA
ATTCAAGTCTTCAGGACAAAATCAAAGAGTTAAAGGTGAAATTTGAATCCTCCTTTATCCCC
ATAAAATCATCGAATGCTTACTAGTTTCCGGTTTATGTCAATCAGACTGAGAAAAACGAGCT
GCGAGATGAGAAACAGAGGCTGAAGACAGAGAAAGAAAAGCTGGAGCAGCAGCTGAAA
GCCATGAATGCTCCTCAACCAAGTTTTTTCCCAGCCCCACCTATGATGCCTACTGCTTTTGCT
TCAGCGCAAGGCCAAGCTCCTGGAAACAAGATGGTGCCAATCATCAGTTACCCAGGAGTTG
CCATGTGGCAGTTCATGCCTCCTGCTTCAGTCGATACTTCTCAGGATCATGTCCTTCGTCCTC
CTGTTGCTTAATCAAGAAAAATCATCAACCGGTTTGCTTCTTGCTTCCGCTTAAAAGAAAAG
TCTCCATTTGTTTTGCTCTCCTCTCTTTCTCGGCTTTCTTAGTCTTATCCTTTTGCTTTGTCGT
GTTATCATCGTAACTGTTATCTGTTGAACAATGATATGACATTGTAAACTCCAATTGCTTCGC
GCAATGTTATCTATTCACATGTAAATTTAAGTAGAGTTTGGCAGATCGTCTCTCACTTTATGT
GTTCTTACATTAATACATAGAATGTGGTTACTTCCTCGCC

>AtbHLH106
ACTTTTTCCCCACCAATATATATATAAATACAAAAATCAAGTATTTAACATCTAAAATAAAAA
AAATAAAAAAAATAATCTACACTCTCTACCCTCTCATTTCACGTATATATAATCCATCTTTTAG
GACAGAACCCACCGAAAGCTCCACAAACCCCATTAATTAACAAAAAACCAAAAGAAGGCG
GCGCAAACAAAAGATAATGCAACCAGAGACCTCAGATCAGATGTTGTACTCGTTTCTTGCC
GGAAACGAAGTCGGCGGTGGAGGGTACTGCGTCTCCGGCGACTACATGACGACTATGCAG
AGCTTATGTGGGTCTTCGTCGTCGACGTCATCGTATTACCCACTGGCGATCTCCGGCATCGG
AGAAACGATGGCTCAAGACAGAGCTTTAGCTGCTTTGAGGAACCACAAAGAAGCTGAGAG
AAGAAGGAGAGAGAGGATCAATTCTCATCTCAACAAGCTTCGTAACGTACTCTCTTGTAATT
CTAAGGTAATATAAACTTTTGATACTTTTTCATTTGGCGTATGTATCTCTCCTCGTACATATGAT
GTTTATACATCTGTACGTATATATTTCTTTGAAATTTTAAGAAAAGATTTTTGAAATGGGTATA
TAATTAGGGTTTTGCATCTTCTCTAACCCAGAGTTTACATCTTTAAAATTTTCTCATGTTTCAT

TCGATTTGGTGTTAGCTCATGAATACTGTACTTTTTCATGATAACATTTTATAATCATTTGGTTT
CAATTGAGTCTTTTTTGTCTTACTATTCTTACTTATTAACAAGCACATATTTATCAAAGATGAT
CCCTAATTTTCTACATTTATAATTCTAAAGATTGATATATATTTTGATTTGAATATTCAAAACTG
GCGAGTAGTGATCAGATGAGAAAAACGAAAAGCTTCATGGCCTTTTTGTGAAGCTCGTTGG
GATAGTATATCACTTTTTGGGAAAGTTCTCAAGCTTTTTCAGTTGATTTGATTTGATACAAAC
ATCAAATTTCAACTTCTTGATCCAAACTTTTCATTGTAATTTCATGTTTCTATTTTCAGACCGA
TAAAGCCACACTGCTCGCCAAAGTAGTTCAACGAGTCAGAGAACTTAAACAGCAAACCCT
AGAGACCTCCGACTCCGACCAAACATTATTACCATCAGAGACCGACGAAATTAGTGTTCTAC
ACTTTGGAGACTATTCAAACGACGGTCATATAATCTTCAAAGCCTCTCTATGTTGTGAAGATA
GATCAGATCTCTTGCCGGACCTTATGGAGATTCTCAAGTCTCTTAACATGAAGACTCTCCGA
GCTGAGATGGTAACCATTGGTGGTCGGACAAGAAGTGTTCTTGTCGTAGCTGCTGACAAAG
AGATGCACGGCGTCGAGTCTGTGCATTTTTTGCAAAATGCTCTCAAGTCGCTGCTTGAGCG
GTCAAGCAAGTCGTTGATGGAACGTAGTTCTGGTGGTGGAGGAGGAGAACGGTCAAAGCG
GCGTCGTGCGCTGGATCACATCATAATGGTGTGAAATGATGAGAATTGAGCACACTAAAAA
GTCTATAATTGATTAATATATATAGGGTATGATCATAATTAACTTGGTTATAATTACCAAAACCT
TTTTTATTCCTCTTTAATAATATATATGTTATATGGTTTTAGTTACTAAGTCTTGGGAGTGTAAG
CAAATGTTGTAAGTAGGTTTGGTGTGTTCTTTTTCTTTTCTTTTTTTTTTCTTTTTCAAGAAA
AAGAAAAAGATCTATATTATATATAAAGAATATTTGTCATGTTATTTTCCTCTATTTATATATATC
TTTTTGGTTGTTGGTGTGATGACTAGGGTTTTGAGATTGTGGGAATATTTGTCGACGTAGAA
GAATCTAAGTAACAGATGTGATTGGTGAAGTCCAAAAGAAACTAGGGTACTTCGATTTACAT
ATACACACTATACATGTGAATATATATGTATATATAGTGAAGAGAAGGTCTATGTTTGTATGCT
GTGAGTTTCAAAGCGACCCTTATGTCACCTTTGTCCCCAAATGAATCCAAACTCTCCTATCT
CTTTCTTTGTTCATGTCTTTCTAAATGTGTACATTTGACCACATTTTTCAGACCAAAGAAT

>AtbHLH107
ATCTCCTCTCACTCTAAGCTTATTTTCGTATAAATTATAGTATAGTCATATTCTTTTAGGACAG
AACCCACCGAAAGAAAGCTCCAAACCCAACAAAAAGGGAGGCGGCGGAGAAGCAAACA
ACAGCAACAAAAAAATGCAGCCAGAGGTTTCAGATCAAATATTTTATGCCTTCCTCACCGG
AGGATTATGTGCCTCGTCTACTTCCACCACCGTGACGTCGTCGTCTGACCCTTTTGCCACGG
TTTATGAAGACAAAGCTCTTGCTTCTCTGAGGAACCATAAAGAGGCTGAGCGAAAGAGAA
GAGCAAGAATCAATTCCCATCTCAACAAGCTCCGCAAGTTACTCTCTTGTAACTCCAAGGTA
CACAAATCTTTTACTTTACTATCTCACTACTCTACTCTAAACTTATAATGAAACTTAGATAGAG
ACTTGAACCGGAAAATTATAAAAAGCATAGAAGATAAAAATGGAAGATTAAGCATGATGAA
ACTATTAATTCTTTTGGGTTATTTAAGATATGAAATTAACTTCTAAAGTTACATATTCTTCAGT
ACTCGTCTAAACATGCATATAATTTAATTAGGAAAAGATTTATGAAATGAGGATTAAAGTTTT
GATGTTATCTCACCAGAATTTTGTGTTTTTAATTAAATCTTTATATCCAAATGACAATTTTTTT
GTCTTAATCAGATTAATCTTGCTTGTGTGATATACGTTACTACAGTCTAGTGTCTAGCTAAGAT
TTTATTTATTGGTGAGAAAGGTGGAATATTGGAAGTATATCACTTATAGAAAATGAACCATTT
TAAAACTAACATCAGTCAAATGTAATGTTTTCGAATAATAAAAAAGCTGCAAGTACTGGGAA
TATCTAGAAAATTGAGGAGATATAAAAAGCTTACAAGCCCCTTTTTTTCTCTGAAGCTGATA
ATCACTTTGTCGGGAAAGTTATCGAGCTTTTTTTTTTCTCATTTGAAAACAATTGAAACATG
AAGACAAAATTTCTCGAATCAAGACAAATTCTAATGATCTGACCTTTTCTCTTTCCTTGTTTC
CTTTATTTGTTTTAATTTTCTTTACACAGACAGACAAATCCACACTACTAGCAAAAGTGGTTC
AACGAGTCAAAGAACTAAAACAACAAACCCTAGAAATCACCGACGAAACAATACCGTCGG

AGACTGACGAAATCAGTGTACTCAACATTGAGGACTGTTCCAGAGGCGACGATCGACGGAT
AATCTTTAAGGTATCGTTTTGCTGCGAGGACCGGCCAGAGCTCTTGAAAGATCTCATGGAG
ACACTCAAATCTCTTCAGATGGAAACTCTCTTTGCCGACATGACAACAGTCGGTGGTCGAA
CAAGAAACGTTCTCGTTGTGGCCGCTGACAAAGAGCATCACGGCGTCCAGTCGGTGAATTT
TCTACAGAACGCACTCAAGTCTTTACTCGAACGGTCAAGCAAGTCGGTGATGGTGGGACAT
GGTGGTGGTGGTGGGGAAGAAAGGTTAAAACGACGTCGTGCGCTGGATCACATCATAATGG
TCTGATCTCGATATGACTTGAGCAATTATTTAGGGTTTAACTTTATTATATAATTAATTAACGTG
AGTATAATTACCACAAATCTTTTGTTTTTTTTCTCAAAAAAAAAGAAGACTTTATATGGGTTT
AGTTATAAGTTTGAGTAATGGGTGTAAACATGTGTTGTAACTTGTAAGTAGGAATTTGTGTG
TTCTTTTCCGATTCTTTTAGAAGAAAATACAAAAAAAGAAAAGGAAACTATAATTTTGTACA
AGTTAAATCAAATTTTAGGGGTACGTAAAGTATAGGGTGATGACGAAGATTAT

>AtbHLH108
ATGAACAAGGACGAAGTATTTCTTCGGCAATGGTTTGAGATACTATACTCGCTCACTAATCC
AGAGGCGAATAGTGACTTGCGTAGGATCAACAACGAAAAAGGTGTAGAGAAAGTAGGGCA
AAAGAGGTCAGCTGAGTCGAGGAGAGAAGGAAAGAAGAAGCGAGTTAAGACACAGTGCG
TGATTAAATCTTCAGACAAATCTGATCATGATACCTTGTTGAAGAAGGTAACCAATAATAGTT
GTATTGTTATGATACGTCTTCCCCAAGAAACTCTTGATTTACTCTTTTCTCTTCTTTGGATACC
TTAGAAGAAACGGCGGGAACGTATAAGAAGGCAACTAGAAACACTTAAGGAAATTACACC
CAACTGCCCACAGGTTCACAACTGCTTCCTTTGTTAACTGATTTTTTTTTCTTGGGAAATGTT
AGTTTTGTTAATGTTTTTTTTTTCCGTATAATCTTACAGTCTGATATCAACGCCATCCTTGATT
GCGTTATCGAGTACACGAACAACTTGCGCCTTGCAGTTCTTTTCGGAGGTAATTTTTTTTTAT
GGCCATACTCTGATCTGTTCGTTAAAGATTATTTATCTTTCTGGTTTTTAAATTTCTCTTCATTT
CCAGCATTACAAGGGTTCGCAGGGAATATGTGACGACTGGAGACTGTTTACTGAAGCTGGT
GCCGTTTTGTATTATATAGATACTTGA

>AtbHLH109
ATCCTTAACCCTCCACTTTTCACTCTTTAACAAACTCAAAATCCTCAATTTAGTCAATCATCA
ATGGAGAGAAACAACCGCAACGAGGGGACTCACGAAGAAGAGCAATGTTCGCTTTCTGAT
ATTATTTATTCGTTTTGTTCAGAAAATCATAGTGAATTAAACCCTTTGCAAGAGATATTCGGT
GTTACCAAAAACAATGATCATGAGAAACACGACGAGGAGCCAGACGAGGAAAGTTACCGT
ATGGCTAAACGGCAACGTTCCATGGAATATCGTATGATGATGGAGAAGGTGAAGATGACTTA
ATCTCCTTCTTCTCTTACGTGATTCCCTAGCTAATTTATATTTTTGCTTTCTAAACTTTGTAGA
AAAGACGAAAGGAGATCAAAGATAAAGTAGATATTTTGCAGGGGTTGATGCCTAATCATTG
CACAAAGGTTAACTATTACTTAATTTACTCCATATTTTTTATATATTAGAGTCTAATACAATATC
AAGTTTCGAATATCGAGTTTCTTGTGTGTTTATCTACAGCCGGACCTTGCATCGAAGCTTGA
AAACATCATTGAATACATTAAAAGTTTGAAATATCAAGTAGATGTAAGCATTAAACTATATCT
AATTTAATTTAGTTACTTGTATCCATTAGAGTATATATGTTCTTATGTGTTATGTGGATGTGGTC
CAGGTTATGTCAATGGCATACACAACAACTCCGGTTTACACACCACCATTTTATGCAGCAGC
TCAAGCACCATGTATGTCTCCTTGGGGCTATTATACGCCAGGAGTTCCAATGATGCCTCAGC
AAAACATGACCTATATCCCGCAATATCCTCAGGTTTGATCAAATTAAAGTTTAATCTGATTCC
ACAGATTCTGATTTCTGCCATCAATCTTTTATCACAGGTATATGGAACAGTGCCGCCAAATCA
AACTCAACCTTAAAACAAGCTCTAGCTATGTGGCTTTTTAAGACCCTCCGGCTTTAGACTCG
TTGCTGTTTATATAGACTGTCTTTGTAAACTAAGATATGTACATAGATACATAAATACATACGT

AGTTTTATGTATAACTTTTAGTTTGATGCCACAATATGTAAAAACACCTGTTTTTTATGTTCTG
TTTTGGTTGAGCATTGTTCCTCTGTTATTTATGTTCTGTTTTGCTCAACCTTTTTCTTTGTTGA
ATGTAAGTTAAATAAACAACAAAGATGTTATGTTACGTGTAATAGTACGTTTGTACCAAACAT
TACTACAACCC

>AtbHLH110
GAGAGAGAGAGAGAGAGAGAGAGAGAGAGAGAGAGATGCGTCAAGCTTCAAAAAAGCTAA
GGTAATGTTATGTGATGTGCTTTTGAAAGGAATAGAGAGCTATGAGAGAGCACATATATACA
ACAACAATTTTTATTAATTACACTTCATCTTAATCTCTTGGGATTTGTAAGAGAAACTCTTCA
AAAATCATCATTCTTTTTCATCTTTCTTTACACACAAACACACATACACACACATATATAATAC
AGAGATCAGGTATAATAAATACTTATATATATAGCATCATCAACTCTCATACATATATGGACTCT
GCTAATCTCCATCAGCTTCAAGATCAATTACAGCTTGTGGGGTCTTCTTCATCTTCTTCTTCC
TTAGACAATAACTCTGACCCTTCTTGCTATGGAGCTTCATCTGCCCATCAATGGAGCCCAGG
AGGTATTTCTTTGTAAGTTTCCTCACTCAATCTCTCTCCTCTGATGTATCATATATTTACATGG
CCATGTATCTATTATATACATCCATTTAACACGTGTATGCTACTAGGTTTCCGCAGCTATATGTG
TGTGTTTAGAATATTCTTGGTATCAAGATGATATGAAGAATTTGGAATCTTTCTTCTTCTTTTT
TTCCTTCTTTTTTGGTTTTTGAAAGATAAGTTAGTGAAGAAGACAACAAGATAGAATTTAAG
TGGCTGATAAATTATTAAATGTCTATTACATTATCCTTCATATATATAGACATAGTACTCTGATT
TTGCATTGATTCGAAATAAAGAAATTTTTTGTGTGGTTTTCTGGCAGGAATAGTGTGAGCTT
GAGTCATAATTATAACAATGAGATGTTAAACACAAGAGCTCACAACAACAACAACAACAAC
AACACAAGTGAATGTATGAGTCTCTCCAGCATCCACAATCACTCCTTGATCCAACAACAAG
ACTTTCCTTTACAATGGCCTCATGACCAATCTTCATATCAACATCATGAAGGACTTCTCAAGA
TCAAAGAAGAGCTTTCCTCATCAACTATCTCAGACCATCAAGAAGGCATATCCAAGTTCACA
GACATGTTAAATAGTCCAGTGATAACAAACTATTTGAAGATCAATGAACATAAGGACTACAC
TGAGAAGCTTCTTCTCAAGAGTATGTCTTCTGGATTCCCGATCAATGGAGACTATGGTAGCA
GCCTTCCCTCTTCTTCTTCTTCCTCTTCACCTTCGTCTCAGTCGCATAGAGGCAACTTCAGTC
AGATTTACCCAAGCGTAAACATATCGAGTTTGAGCGAATCTCGGAAGATGAGCATGGACGA
CATGAGTAACATCTCAAGACCATTTGATATAAACATGCAGGTTTTTGATGGAAGATTGTTTG
AAGGAAATGTATTAGTTCCTCCTTTTAACGCTCAAGAGATTAGTAGTCTTGGGATGAGCAGA
GGAAGCCTTCCTTCTTTTGGCCTCCCTTTTCATCATCATCTGCAGCAAACACTTCCCCACCTT
TCTTCTTCCCCTACTCATCAAGTAAGTTTATTTTGAATTATTACTTTCTATTTCTTTTCTCTTTT
CGCTTTTTAAAAAAAAACTTTTCTATATATATATATAAATGATGGCTTAGAAGGATTGGTCTGA
CGGTGAATCTCGTGAAGTTAGTGCATCTGAAAACATTACGTTTAGGGCTCTATTTCTATTATG
TAACATAGATTCACTATATATATATATATATATATATATATACCCTCATTTCTTAATTCTAAAATGT
AGTTTAATATTACTGTTTCATAAAATACTACGGCCTAGTTACCTTTCAGCTTTAATCATATAATT
TCATTTCACTCATGAAAGAGTGTCTAAAAGATTAGTTTTCTGTTTTATTTATAAAAATGCTTA
GTTCTCTGTGTACCCCTTCAACTTTATTTATGCATCAATGGAATGCATTGCAATATTATTCGCA
TAACTTTTTGTAAACGTAAAATCATATTCATAAGGTGGATAAAATATCATGTTCATTAAAAGT
TTGTGGCATAAATCAGAAAAAAAAAAAAGAAAAAAAAAAAGAACTTGAGAAATAGCAAAAA
ACAAAAAAAAATATATACTGAAATATTATAATCACGAAGTATAGATAGATTAATCAGATTAGTGT
AAAGTGCGAGACCTTTGTGCAATATTGAAGCTTTGCAACAAAGTTATGAATAGAGAGATAAT
ATTTAAATATATATGTAAAAACTTTTAACCTCTTTTGATTCATCTTTTACTTTGTTGTTGTGATT
CTTGGTTGGAGGGCCAAAGCTTGAGCTAAAAAACCTTTTAACATAATTCATAAGCAGCACT
CTTTAAGCTGAAACATCAACCTTTGTTCTATCTTTTCTTTTCCTTTCTCTTTATTCCTTACTTT

GTTTTATTGTCTTTTTATGAATCTCTTTTCTTAACATTTTTTTCTCTCATTGTCGGGGGTATTAA
TTATTACGTTACGTTCTATTATTATCCTTTACATCGAATAGCAATAATGAACGTTATTTGATACT
TGCATACATATATATGTGTGTGCATTACTTATGGGGTTTTGGTTTGGGTTGAAGATGGAAATG
TTCAGCAATGAACCTCAAACAAGTGAAGGGAAGAGGCATAACTTCTTGATGGCAACAAAA
GCAGGAGAAAATGCTTCCAAGAAACCGCGCGTGGAATCACGCTCCTCTTGCCCACCCTTCA
AGGTTATTACTTTTGTTTTAAAAAAAAATACATGCAATTAATTATAAATGTTTTTTTTGGAGAAA
TTAAATAAAGCCTGAATAAATTTTAGTAGTTAAGAAGAAAAAAAAAAATGAAAGAAATTTTTC
TAATTGGAGGATATTAATTTAAAAAAATATATATAGTCAAATGTTAAGATAATCCATTGAATGA
TCATTGTAGTGTTTATACGTTGTGAAATTGAAAAACAGGTGAGGAAAGAAAAGTTAGGAGA
CAGAATAGCAGCTCTGCAGCAGTTGGTTTCACCCTTTGGGAAGGTAATTAATTAGCAACAAT
TAGTTTTACCCAATTTATTCCTTACTTCAATATTTCATTTCCTTTTTGCCCCATTTTAGAATATG
AGCCTAATCATTAATAAAATATATAGCTGTATGAAAATTGATTTTGTTACAATAAGATATTTTT
GTTTCTACCATTTAAAGATAGTAAGGATTTTAAGAAAAAAATTCACAAGTAGCGGATTGTTA
TTTTTTGCTTGTTACTCGATTGTTGATGATTAATTACATGATGATTAAAATGTTAAACTGCAGA
CAGATACAGCATCTGTGTTAATGGAAGCAATTGGATACATCAAATTCCTACAGAGCCAGATC
GAGGTACGTAACTTCAATGCATGCATGTCACTCATTCATATCCATACATCCTATACTTATAGTT
ATCAGTACATGACATTTGCATGAGAAAAGGAAACATATTCAAGGAATGAATTTGGAGTGGTA
GAATAACGGTATTTACATGGTAATACCGTGCTAAGTTCCAGACATTCTTCTTGATCATCGTTC
CGTTTTGTCAAATATTCTCAACTTTTTATATCAATTATTACTAAATATACTATCTTATATATTAAG
GACTTTGACATGTACTGAATTATCAAACTTTAAAACACTATTGTGTATGGGCTAACTTATTAC
TTATGAACTTATATTTAATTAGTTCCATAGGGTTTAGCTTGCCTTTAAATTAAAGCAAGAGATT
AGTTAGAATTGATCTTTATTTTATCATATCCTCTTAGGCGCACTACGTAGTAATTAAGCACCTT
TCTCCTGTTTCTTTTATGGATACATGTTTAGATAAGCTCATGCTCAATATTGCTCATGGTCGTC
AATCTTAAGGATCTAAATTATATGTACAAATTAAAGAGTAAAGTTTTTATAACTCAAATGTCA
ACTTTATTGTTTGATGAGCAGACTTTAAGCGTCCCCTACATGAGAGCATCTAGGAACCGACC
CGGAAAAGCCTCCCAGCTGGTATACTTTTGAGAACCTATATAATTCATTACTAGTTTTACATA
AAAGTAAATCATTCCGGATATATTTAGTCCACAAATTTACTTATATAGTAAAATGTTGATTAGG
TCTCACAATCACAAGAAGGGGATGAGGAAGAGACGAGAGATCTTAGAAGCCGTGGGCTAT
GTCTAGTGCCGTTATCATGCATGACTTATGTTACCGGAGATGGTGGGGATGGAGGAGGCGGT
GTTGGTACTGGTTTTTGGCCAACGCCACCTGGTTTTGGTGGCGGAACTTAGCCGTGGACTT
AACAAACCGTAGGACTATGATGAGTACATTTATCGGACTTGGAGGTAGAGAATAAGAAGAA
ATGTTAAAGGTGGAGTATTAGTTCTTTAATCTCTTTTGGTTTTGGTTTATTAATTGAAATTTTC
GGTTTTGATAGTGGAGCAAAGTTGGTCGTCCTGATTAGAAAGAAGTGTTACAGGATAGACC
AGCTTTGATCCATTTAAGATTAGTAGTGAGACTTGACGATATGTTTCTACTTACATGATGGGC
TGTGGGGGCTACATAAAATATCAAATAGCTTTGGATTATTTTGTTAAATCTCTTTTGTAGATAA
TGTGTCAATATCTTTAATAAATGTATTCCTTAAAAATAAAAAACATATGAAAATATTTCGCCAT
GTAGTAAGAGGACT

>AtbHLH111
CCACCACCGATCGTATCATATATACACTTTATATTCTCTAATGCACATATCCTAGACTCTTAAC
CAACAAAATAAAAGAAAGAAGCAAAAAGAAAAAGCTCGAAGATAGGAAAATGTTAAGGG
AAGAATGTACTCCAAGTTCATCATGGTGGGAAGATGTTCAACATCATCATAATGATCATGCA
AACTCGATTAGTTCGACGTCTTTCTACCATAAGAGTAGTAATAATAACTCACACGCCAACGC
TAGCTGTGAAGAAGACAATCTCTCCGTCTCTACCGTACGCGCTTCGAATCGTTTGGACCTAA

CCGCTGAATCCTCCAATCATCACTCTCTCTCGGCTTCGAATCAGCCTGCCTCCTCCTCCGATG
AGTTGCTTCGCGACCATGTAGTATCTTCTCATAATCATCTATGGAGTCTTGCCTTCTTGTAAG
CATCTATCAAAACACTTAAATTTTATTTATCTTTTCGAACCATTAACAAATCTTCTTTAGTATC
GTGTTCTTACTTACAATTCACATAACGGTTATTGTGGATTCAAAGTTTAGTTTAAGGTTTTAG
GTTCTACGCTCCACTCCATAAAGAAATCATCTAGTTCTCACTATTAATACTTTCGTTTGTTTAA
AAAGAATGAATGCTCCAGGCCTAAAAATAAAACAAATAAAAACATTAGAAACAAAAGAGA
GGTCTAAAAGTTGGTTTACAAAAAGTATATTAGTAGCTCTTTTGAATTATTGTTCCCCAGTAA
ATGATTTAACGTTTGATTGGTTAGGGTATGCCTATAATTTTGCACTTATGATTATTCATCTTTAT
TATTGGCCACTCGAAGATGTATTTTTATTCTTATTTTATAATTAAAAGAAGTTTAATAGTTTAT
AAAATACTATTTGTTTTTGTTTTTGCGTCTACTGGTTCCAATATCATGTCTTACAATTTTAAAA
ACAAAGTATAAACGACAACGTAATTACTTGAACAATATACGAAAACGTTATCATACCAAAAA
AATATATAAACGAAAGGTCAAATGCAAACCAAATTAAAGTACCACGTTGTAACTTTTTACAA
TTTCCCAAAATTATTTAGTGGAAGTTTTAATTTGTAGAGTTTCCTAAGTAAAATTAATAATCT
AGCATAACACTAAATTCATTAAAACCAAAAAAAAAATCGAGCCCATATTTAACTTAAGTTTT
GTAATACTAAATACCATATTCTTACACACACATTGACATTAATTTTTAAAAAATGGACAGGCC
TGGTAGAAGCTTAGGAGATCAAATGATGGATCATCATCATCACATAGCATCAAGAAATTCAT
CAACAACATCAGAATTACCATCATTCGAGCCAGCGTGCCATAACGGTAATGGTAACGGTTGG
ATCTATGACCCAAATCAAGTTAGGTACGATCAAAGTAGTGACCAACGGCTGTCAAAGTTGA
CGGATCTTGTAGGCAAGCACTGGTCAATTGCACCACCGAATAATCCCGACATGAACCATAAC
CTTCATCATCACTTCGATCATGATCATTCTCAAAACGACGACATTTCTATGTACAGACAAGCC
TTGGAGGTGAAAAATGAGGAAGATCTTTGTTACAATAATGGCTCAAGTGGTGGTGGTTCCT
TGTTCCATGATCCTATAGAAAGTTCTAGAAGTTTCCTTGATATAAGGTTAAGTAGGCCATTAA
CGGATATTAATCCGTCATTTAAGCCATGCTTTAAGGCCTTAAACGTATCCGAGTTTAACAAGA
AAGAACATCAAACGGCATCTCTGGTACGTATACGCTATCAAATATGTGATATCATTATCATAG
CTTGCTAACGACGTCGTTTCAATTTGCTTATTATTTGATAATACGTTGTACTTTAGAATGGATT
TAACGTTGATATTTCTGTTGCAATCTTTAACTTTGCTTAAGGATGCTACCATGCATTAAAATTT
CTTCCCAGCATTTTGTGGCAAATATATATTCTATTTTGAAAAATATACTAAAATAACCACAAAT
AATTTTCAGTTTCGAAGATGCCATAAGTTTGAGAAAATTCTCAAAAGTACCACCACTTAAGC
TTTTAGGAATTATTTCTAAATGTAAAGTTTGTGGTAGTTGAAATTTCCTTTTTTTTTCTTAGTA
AAGTGTTTTTATTTTTCCTTTTGCTACTATTTGCATGGATTGTTTCTTAGCGTAAGAGGTTTCT
AGTTCGCCTTTAGAATTGTCATCCTGCATATACAATTCAAATCCACAAAACAATGTAATATTA
CAAATGAAAGTCTATGTACCGACGTATAAACCCGAATTTGGAAGCTATTTTTGCAGGCAGCA
GTGAGACTGGGAACAACAAACGCTGGAAAAAAGAAGAGATGTGAAGAAATTTCCGATGA
GGTTTCAAAGAAGGCCAAGTGCAGTGAGGGCTCTACACTTTCGCCAGAGAAGGTAATATAA
TCTACATAATCTCATTATGTTTTGTGTGTACCTAAGTAAGTCTAAATTCATGCAAAAAATATAA
TGTGTTTCTTAGGATAACTTAAAAAACAAGTGATGAATCTACTTTAAAAAAAAAAAAAAAA
CTATACGTCGACAAACACTGAATGTGAAAATAATCTTTAAAAAAAAATGTATGATTCGTAATG
GGACATATAGATAACAATTGCCCTGATTTTGTATGGTAAACATATGTAAATAATTTGTCACTAT
TATTTTCATATGATTTGTTCATGTTATGATATTAATTGTTTGAATCATACCGGATAAATAAAATC
AAAATTCTAACATTATTTTCGAAAAATAAATGGCAGGAACTACCCAAAGCCAAACTTCGAG
ACAAGATCACGACTCTACAGCAAATTGTGTCTCCCTTTGGAAAGGTACTACAAAAAAAGAC
CGTACTCCTTTTCTTTATTTGTTGTCATATTATTTAAAAATATTTTATTTTTGTAACAGCTTTT
AGTTTTTTTTTTTAATTTTATAAATCATATATAGTTCTTCTCTCTTTCAACGTTTCTTTTCAAAA
AATACAACGAAGAAAATGAAACATTTCGTTTGAAAACATTTCAGACTGATACTGCTTCTGTG

CTTCAAGAGGCCATCACTTACATAAATTTTTATCAAGAGCAAGTTAAGGTAAATTATATACAT
ACACGGTAAAATTTACGAAAATGAAGGTAAATTGTTGCATGTCTATGCAAGCATGACTATCT
TAGTTGACTGTTGGTGATTTTAACGAACATGCAGCTGCTAAGCACTCCTTATATGAAGAATT
CATCAATGAAGGTAACGTTTATAAAATATTTTCTTCATGACGTTTTAGATAATGGAATCTCTTC
AGCTAATTAATCATAATGTCATATGTATGGTTTTAGGATCCATGGGGGGGATGGGACAGAGA
AGATCACAACAAAAGGGGACCGAAGCATCTTGATCTAAGGAGTAGAGGGCTTTGTTTGGTT
CCTATTTCATATACCCCAATCGCATACCGCGATAACAGTGCAACTGACTACTGGAATCCCACG
TATAGAGGTTCTTTGTATCGTTAGTGACTAGTATAGCAAAAAGTGTGTAGAGAGAAAGATTT
GATTAAATTATGGTAGTGGTAAACCATTCCAAACATCGATCATTTTATAGGGAGCAAGCAGTA
AAACGGGAGAAGAGTATGTGTACATAAAGTATATGCATATTAATCATTGGGGTCACACTTCTT
TGATTTGCAATTGTTCTTGCGTAACCTAAAACATTGTGTTATGGTCATATATAGAATAATACAT
TAATATTGGAGTTTGAATTAGATATACTACAATGTAATAAAAAGTAGTTAATTAATGGCATATT
CCTTGTTGATTTCAA

>AtbHLH112
GTCAACTAAATTCCGGTAATTTCCACACACTTGTCCGTACTTGCCTATATAAACATACACACC
CTCATAAACATATATCAAAATTTTACACTTATGCTCTTCATATCACATACAACAAACTCATATAT
ATAAAAACTTTAACCTTCGTATAGCAACGATCTAGTAGAAAGCTTATACTAAAACTTTCTGAT
TCTCTTAATATGGCGGAGGAGTTTAAAGCTACGGCATCGATTTGTGGTGGCGGAGGTGGCGC
GTGGTGGAACTCGCCAAGGAGCGTTATGTCTCCTTCGGATCATTTCTTGTCACCATGCTTCG
GAGCGGCTATCACTTCCAATGATTTTAGCTCGCAAGAGAATCATCTCAAATCTAGGATGACT
TGTACGGACAACAACAACATCGTTTTCGGGCAGCGAGAGGCGGATTCTGACAGTGGAGGA
AGTACCGTGACGATGGACTCGACGTTGCAGATGATGGGTCTAGGGTTTTCCTCAAATTGTTC
TTCAGATTGGAACCAAACCATTTTGTAAACTTCTTAAAGAAAAACCCTAACTTAATTTTTTTT
GTTCACTTTCTTGATGAACAAGATTGAAGATTTTTAATAATAATCATGAGATCTCTTTTGTTCT
TGTTTTGTATTTTTCAGACAAGAAGACTTAAACTCAAGCTTCATAAGGAGCTCACAAGATCA
AGATCATGGCCAAGGATTCTTATCCACAACTACTTCACCTTATATACTTAATCCAGCTTGTTC
TTCATCTCCATCAACTTCTTCGTCCTCAAGCTTGATACGAACCTTTTACGATCCCGAACCAA
GTCCATACAACTTTGTCTCCACCACAAGTGGTTCAATCAACGATCCTCAACTTTCTTGGGCT
AATAAGACAAATCCTCATCATCAAGTAGCTTATGGACTAATTAACAGCTTCTCTAACAACGC
AAACTCTCGACCCTTTTGGAATTCTTCATCAACCACTAACTTAAACAACACGACGCCGAGC
AATTTTGTAACCACTCCGCAGATAATATCTACTCGTTTGGAGGACAAAACCAAGGTAAATTA
TCTCATATATGTCAAAATAATTTTGCTACTATATAAGTTTTGTGGCTATATTATTGAATTTATTTA
TTTATTTATTTTTGTTGAGAAGAATCTGAAGACAAGAGCTCAGAGTGAGTCTTTGAAGAGA
GCAAAGGATAATGAATCTGCGGCGAAGAAACCGAGAGTCACCACGCCTTCACCGTTGCCA
ACTTTCAAGGTACATGATCATATTTCAAGATTTGTTTAAGACCTTTTATTTTAATTCAATTTCT
TGGTGGATAAAATGGATTGTATATGTAACAGGTGAGAAAAGAGAATCTAAGGGACCAGATA
ACTTCATTACAACAACTAGTTTCACCTTTCGGAAAGGTGAATGATTTATGTGTTTACTTTATT
TTATATGGCTTAATATATACAACCAAAAGCATTTATTTTCCCTTGGTTATGTATTGAGCTTCTTA
GTTTGTTTATTTTGTATATCTTTGGGTTAAACTTCAGACAGATACTGCATCTGTTCTCCAAGA
AGCTATAGAGTACATCAAGTTCCTTCACGACCAAGTCACTGTACGTTTCTTGTGATTTCTCTC
CTAACTCACTGAATTCATAAAACCCTAGAAATGAGCAAATTTTTAAGCATATTAATGTTTTTT
AAATACAGGTTCTAAGCACTCCATACATGAAACAAGGTGCCTCGAACCAACAACAACAGCA
GGTTCAATACACGATCTAGTGTTTTAGTTTTGTCCAAGTTGGAGTCTTCGTGCTATAATCATG

GTTAAATAAGTACTAATTTCAGTATGGATTCATGTAGATATCTGGTAAATCGAAGAGTCAAGA
TGAAAATGAGAATCACGAACTAAGAGGACATGGTCTATGTCTCGTCCCAATATCAAGCACG
TTTCCGGTGGCTAACGAAACAACAGCCGATTTTTGGACACCAACGTTTGGGGGCAACAATT
TCAGGTAGAGAAGGTGAATTACGTATATCATACGGTTCTTAATTAGCATTAAGATATTATTTAA
TAATGCTTAGCATAAAAATAAAAGAAAATAAAATTCACAAGTCATGATCAAAGATTGAATCA
TTACTTGGAGAAAAAGTAGAGATGGGAGAAGAAGAAGGTGACTCTTCAAAGGTGGGCAGA
GGCAACAATAGTGTTGGAGGGGACCAACTACGAAAGCAAGAATGATTTAGGGCTTAGAAA
ATTTGGGATAATAATCTATCATAAACTAATAGATAGATATATTTTTAAAAACATTAACTTACATA
CTCTCATGTTATATATGTAATAATTAACCTAAATTTCACTTTTTAATTATATATAGGTGATTAAA
GAGAGAGGGTTGTGGTTGTCAAGACGGATGGGAACATATGCTAAATTCAGCTTGGAGACTC
TAAAGCATAACAATAATTTTTAATGTATTTTCTTCTTCTTTCAATATTTTAAGGGGGGTTTATT
GCTGAATTTAGCATATATGGGTTGTTTTTATGTTGAATAACCTTTTGTTCCTGTCATTGTTCTT
GAATATATATATATACATATTTTTGTCGTTGGTAACATCTTTGTTAATCACTTATTAAAGTTAGA
GAACATAATAATTGGTGGTTTGTCATATGAAGTTCTTTGTTGTTTTTATAGAGTTGTTTTTTTC
ATGTTTAATCATAAATTTTAATTTTAACAATCACAAAACCAACACTTATAACCTAATTGTTTGT
GATAG

>AtbHLH113
ATAAAAGTCTAAAAAAGGTAAAGAGAAAAACATTTCACCAAAAAAAAATCCTCTGGTTAATG
TTTTCTCACTTTCTCTTTGTTTTCTTTCTAACCTCTTTCCACGAAAAGTCACGATGGGAGATA
CCGCAGAGGATCAGGACGACCGAGCTATGATGGAAGCTGAAGGAGTAACGAGCTTCTCCG
AACTTCTTATGTTCTCTGACGGCGTTCTCAGCTCCTCTTCCGACCACCAACCTGAGGGTAAT
GTCGGCGATGGTGGAGAAGACAGTTTAGGTTTTGTATTCTCCGGCAAAACTGGCTCAAGAA
TGCTCTGTTTTAGTGGAGGCTATCAAAACGACGACGAGTCACTCTTCCTAGAACCTTCTGTT
CCAACCTCCGGAGTCTCTGATCTTGACCCTTCATGCATTAAGATTGATTGCAGGAACTCAAA
CGACGCGTGTACGGTCGATAAATCTACTAAATCATCAACCGTAAGTCTCTCATTACTGATTCT
TTATTTGCATAGTTGCATGTAATTAAGGATTACAAATTTAAAGATGTGTTTTTTATTGTGTGTG
TCTTGTGAAGAAGAAGAGAACCGGAACGGGTAATGGGCAAGAGTCGGATCAAAATCGAAA
ACCGGGCAAGAAAGGGAAACGAAATCAAGAGAAATCATCAGTTGGAATTGCAAAGGTGTG
ACATTTAATAGTATGTATATACACATTTCTTTATGATCCATATTGTTATAGATTAATATTCTCTTG
ACCATAATGTTATAGATTTACTGTTTATCTTTTTTCGTTTTGGTTGTTAAAATATTAAAATTAGG
TCAGGAAAGAAAGGTTAGGGGAAAGAATTGCAGCGTTGCAGCAGTTGGTTTCTCCATACG
GCAAGGTGATTTTTATATCTAACTCAAATTCTTTTATAATGATGCAATTTATGATTTTGTTAAG
TCAAGCAAGAAAGACCTCTAATCATAACATAACACCAAAGTAATGAAAAATAATTTGTTTTG
AATATTAATATAGTATTTCATATTGAGTTTTTCCAAAAGGAAGCTTAAGAAGTCGCGATTTGG
GGTAGTGTAGCATAAATTGGCATCCAATGTGAACTTCAAAAAGACATGAAAGACAATGAAA
CTTGATCAATAAATTAAAGTATCTTTGTTTTTCTTTATTCTAGTCTGGTTTATGTTTAACCATCA
TCATTGTACATTAGTTAAACAATGTGTACCTTTTTAGAGGGGTAAAATTACCAAACAAATATC
TATAAATGACCCTATTTGCAATAGTAAAATATCTATAAATGACACTATTTGCAATAGTAAAATT
TGACTTTTCTGACTGATACAAAAATAAAATTAAATGACAGACAGATGCAGCTTCTGTGTTGC
ATGAAGCGATGGGTTACATCAAATTCTTACAAGATCAGATTCAAGTCCTTTGCTCTCCTTATC
TAATCAACCATTCTCTCGTAAGTAGAAGTCTCTCTCTTTCTCTCTTAATTATTTTTTATTAATG
AAATGATACTTCTGACATGTCGGCGACGTTTATTAGGACGGTGGAGTTGTCACCGGAGATGT
AATGGCGGCGATGAAAGCGAAAGACTTGAGAAGCAGAGGATTATGTCTAGTACCTGTCTCA

TCCACCGTACACGTGGAAAATTCCAACGGTGCTGATTTCTGGTCACCTGCTACTATGGGTCA
CACTACGTCACCGTCGTTACCTCAAGGATTTTAATTAAACGGTCGTAGGCTGATATATATTAG
TAGTATTTTAGTAGCCTTTTTTCTTGGGTTTTGTTTTTGGCTGAAGAACTATTACTAGTAAATG
TTACACCCAGACATTTTTTTGGGATTATCTGGGTGGTATAAAAAGGTTGGAATCTTTTGTGTT
TGTAAAATTAGATTGTCTAAGTTTTCTTAAAGTTTCTTTGATGTCACTGTCACAGTTGCGACG
AGTAGTGATGGTACTTACTGAAAGGCTAAGCTTATTCACGCTCTTAAAGTGTTG

>AtbHLH114
TACACTATGTAATAAACATCATGTCTCATGCAACTTCGACATTAAATTCCAAAGTAAACTACA
CAATTAAGAACATGACGGAAGAGTTTGAGATCGCAGGAATCTCCACCGGAGCTTGGTGGA
GCTCGCCAACAAACACCGCCGCAGTATTCTCTGGCTACTCGCTGCCATGTTCCACTGAGATT
AGCCCTGATGTCACAAATTTCGGATGGCAAAACTTCGATAACAAGATTAACGATCATAATGA
TGGTTGTATGAACATGTAAGTCCACATGCATACATACATTAACATACAAAGTAATAATATTGA
ACTTTCTTAAATTTATATTCTTTCTTTTATATTTCTTGTAGGCATAATAGTTTCTTCGAAGGACT
ATTAATCGATCCTAACGATCAATTACTACCGGATCCATGGTCCAAAAGTACAATTCCTAACGC
TAAATCTGAGCTCCTTGAAAACTTCCCATTCTTAGATAACATGTTCTTGGTCGATTCAGAAG
CTGAATCTTTGTTGGATCATGAAATCAGAAACCATAAGTCGTCTAAGGAGCAAATTACACAA
GACTACAAGAATCTTACTTCAAAGGCACGTAACAAACATAAAAACCTAACTATTTTTGTTGT
GACTAATACTTAGAGGTAATTAACATTACAAAAGATGTGATGATTCTTTCAGAGGAGTGAAG
AATTAGAAGAGAACAGCGATGAGTATTCACCGCGATTACTAAAAAGACCGAGACTCGAGAC
ACTATCTCCATTGCCAAGCTTCAAAGTTCGTAAAGAAAAACTCGGAGATAGAATCACTGCG
CTTCAGCAACTGGTTTCTCCATTTGGCAAGGTAAATTAAAACAACATTACTCTAAGTATTTTA
CGCATGTTTAATCTTTGATTTCTTCTTATTTAAGCTCAAAAGATTTGTCTTCTAAACTCTTTGC
AGACAGATACAGCTTCTGTTCTCAACGAAGCTGTAGAGTACATAAAGTTTCTTCAAGAACA
AGTCACTGTAAGTTCAGATTCTTGATTTTTCATTTGCAGTCAAAATCAAATCAATTAATTAAC
AATTCGTTTGATTCTTTAATTTAAAAATTAGGTATTGAGTAATCCAGAACAGAACACCATAGG
ATCTGTTCAACAACAGCAGGTACTTAAAAATTAGTTATATGTTATAACAACTCTCTTTTCTTG
TCCTTTTGCTTGTGACATCAAGAATCTTTTCAAAACTAATCATCACCAATTAATGATTCGTAT
AATTAATAGTGTTCAAACAAGAAATCTATCAATACTCAAGGAGAAGTAGAAGAAGACGAAT
GTAGTCCAAGAAGATATGTAGATCTTTCGAGCCGTGGACTATGTCTAATGCCGATCTCAGCT
TCATATCCCGTGGCTGCAGCTGCGGCTTCGGCTGCAGAGATGAACGTACATCTGGTTTCTGG
CATCTTCCATTCTTTGTAAACTTATAAACTAAATACGTAGATACATAAACAAACAAAGTAATC
TTCCGCAAACAGGGGATCTTGTAAAGTTTTATGTCCCTTTTTAATTTATACAGAAATGTAAAT
GTCCTGTAATACTTCAAAATCATATATTGTTTGTTTGCGTACGTTTACTATTTTTCTCTAAATG
AACCTACTATTTGAAGCAAAA

>AtbHLH115
AAGAGAATCCATGCCTAGCACTATACCATATCTCTTTTTTCTTCCGTTTTTCCACACATTGTCT
CTACCTTTCCCTTTTTCAGATTTGACCCTTTGTTTTCAAAAGAAACGATTTTTAGTAGCCGCC
GGGGAATCAGGAGAATGGTGTCTCCGGAGAATACGAACTGGCTTAGTGATTACCCTTTGATT
GAAGGTGCTTTCTCTGATCAGAACCCCACTTTCCCTTGGCAGATAGATGGCTCAGCTACTGT
CAGGTTAGTTTTTTGTTAATCAGATTCAAGGGTTTCTGGGTTCTGTGTTTTGTTTCTTTCACC
ATCTATTAGTTTTGTTTCTTGGCTTTGGACTTTTGGTGTTTTAGGATTGGTAGTTATATCTGGA
GAAGTTGTTATGGATGTGTGATCACTCAGTTGGTTTTTAGATGCTCTTGTTGTGATAGAGATA

TTGGACTTCTGCATAAATCAATCCTCTGTTTTTTTTTTTGTCTCCGTTTTGCTTACGTTCCTCT
GTTTCCTAAAGAAGTAGACGAATCTAGATCTCTACCAATTGCGAAAGGAAGGTTCTGTAATT
AGTATCTGGAGAAGTTCTTTATGGAGTTTGACCTTGTTGATCACTCAGATTGCTATTAAATGG
TCTTCTTGTGATAGAAATATGAAAGAACTGAGAACCTAAAGTATAATGCTTTTTCTCTCTGTT
TTGCATCCATTCCTCTGTTTCCTTAAGCAGTAGACGAATCTTGCTTCTATTCTGGAATTCTAC
AAATTGGCTGTAATCTTTGTAATTGATCCTTCCTCTTGTGTTGATGTGATCTGTTCACAGTGT
TGAAGTGGATGGCTTCCTTTGTGATGCAGATGTGATCAAAGAACCAAGTTCAAGGAAGAGG
TATCAAACGCTATTTGGTCAAACCTGCGTTAACTAATGACTTTCTTGTTCTTGCTATGCAATA
TTGGAACCTGTTGTAAAGTTTCCTTCTTACTGCTGCAGGATCAAAACTGAATCTTGCACTG
GTTCTAACTCGAAAGCTTGTAGGGAGAAACAAAGACGTGATAGACTAAATGACAAGTATTT
TCTTGCTTTCTCGTCATTCTCTTCTATCATTTACCTTTTGAGTTCTTAATCTTTTGGTATGGTGC
TTTGTCAATAGGTTTACGGAGTTGAGTTCCGTATTGGAACCTGGGAGAACTCCAAAAACAG
ACAAGGTTGCTATTATCAATGATGCAATTCGCATGGTGAATCAAGCAAGAGATGAAGCGCA
GAAACTAAAGGACTTGAACTCAAGCCTCCAGGAGAAAATCAAGGAGTTGAAGGTAAAAAC
TGAAAACTCCTGCAAGATCTTTAGCTTGTCCATTGATCTTATGTATATGCCAATATGTAGGAT
GAGAAGAACGAGCTGCGTGATGAGAAACAGAAGCTTAAGGTCGAGAAGGAGAGAATCGA
TCAGCAACTGAAAGCTATTAAGACACAGCCTCAGCCTCAACCTTGTTTCTTACCAAATCCGC
AAACACTCTCTCAAGCTCAAGCTCCTGGAAGCAAGCTTGTCCCTTTCACAACTTATCCCGG
CTTTGCAATGTGGCAATTCATGCCTCCTGCTGCTGTTGATACCTCACAGGACCATGTCCTTC
GTCCTCCAGTTGCTTAAAGCTGCTGCTTCTCTCTACTACTATTAACGGTTCTGTAAGATTACT
TCTTACGCGCTTTTTTCTGATGTAATGATTCTCACATTCTGTGATTGGTGACATAGTCCACTG
CAACTTAAAATGTAAAATTGAAATAAGCTTGCACTAAAAATCAAATCTCATTGTGCAA

>AtbHLH116

TATATAAAGACAGAGACAGTGACAACAGAGACAGAGAGATAAGCTTAAGCCAAAATTAAG
CAGAAAAATGGAGAAATAGTGACGGTGAGAGAGAGAGAGATACGGTAAACGAAGCAAAG
CAAAGAGAGTCACGAGAAATCTGGGGTATGTGTTCAATGATAAAGCAATTTCATGGTGGCC
GAAATTGAATCCATCAAAAAAAAAGTTTCAATTTTTGAAAGCTCTGAGAAATGAATCTATCA
TTCTCTCTCTCTATCTCTATCTTCCTTTTCAGATTTCGCTTCTTCAATTCATGAAATCCTCGTG
ATTCTACTTTAATGCTTCTCTTTTTTTACTTTTCCAAGTCTCTGAATATTCAAAGTATATATCTT
TTGTTTTCAAACTTTTGCAGAATTGTCTTCAAGCTTCCAAATTTCAGTTAAAGGTCTCAACT
TTGCAGAATTTTCCTCTAAAGGTTCAGACTTTGGGGTAAAGGTGTCAACTTTGGCGATGGGT
CTTGACGGAAACAATGGTGGAGGGGTTTGGTTAAACGGTGGTGGTGGAGAAAGGGAAGA
GAACGAGGAAGGTTCATGGGGAAGGAATCAAGAAGATGGTTCTTCTCAGTTTAAGCCTATG
CTTGAAGGTGATTGGTTTAGTAGTAACCAACCACATCCACAAGATCTTCAGATGTTACAGAA
TCAGCCAGATTTCAGATACTTTGGTGGTTTTCCTTTTAACCCTAATGATAATCTTCTTCTTCAA
CACTCTATTGATTCTTCTTCTTCTTGTTCTCCTTCTCAAGCTTTTAGTCTTGACCCTTCTCAGC
AAAATCAGTTCTTGTCAACTAACAACAACAAGGGTTGTCTTCTCAATGTTCCTTCTTCTGCA
AACCCTTTTGATAATGCTTTTGAGTTTGGCTCTGAATCTGGTTTTCTTAACCAAATCCATGCT
CCTATTTCGATGGGGTTTGGTTCTTTGACACAATTGGGGAACAGGGATTTGAGTTCTGTTCC
TGATTTCTTGTCTGCTCGGTCACTTCTTGCGCCGGAAAGCAACAACAACAACACAATGTTG
TGTGGTGGTTTCACAGCTCCGTTGGAGTTGGAAGGTTTTGGTAGTCCTGCTAATGGTGGTTT
TGTTGGGAACAGAGCGAAAGTTCTGAAGCCTTTAGAGGTGTTAGCATCGTCTGGTGCACAG
CCTACTCTGTTCCAGAAACGTGCAGCTATGCGTCAGAGCTCTGGAAGCAAAATGGGAAATT

CGGAGAGTTCGGGAATGAGGAGGTTTAGTGATGATGGAGATATGGATGAGACTGGGATTGA
GGTTTCTGGGTTGAACTATGAGTCTGATGAGATAAATGAGAGCGGTAAAGCGGCTGAGAGT
GTTCAGATTGGAGGAGGAGGAAAGGGTAAGAAGAAAGGTATGCCTGCTAAGAATCTGATG
GCTGAGAGGAGAAGGAGGAAGAAGCTTAATGATAGGCTTTATATGCTTAGATCAGTTGTCC
CCAAGATCAGCAAAGTAAACACTTACTTTGTCTCTTTTATCTCCTTAAGAGCTTGTTTACTTG
TTGCTGTTATAGAGAATTGTTGTGTTGTAGCTTTGTAGGGCCTTTGTTGTTGTCAAACTTTGC
ATGTAGTTGCTTTACTCTTTTGAGGAAGAGGCTCGTGATAGTGTTTTATGGATTTTCGATGAA
TTTGCAGATGGATAGAGCATCAATACTTGGAGATGCAATTGATTATCTGAAGGAACTTCTAC
AAAGGATCAATGATCTTCACAATGAACTTGAGTCAACTCCTCCTGGATCTTTGCCTCCAACT
TCATCAAGCTTCCATCCGTTGACACCTACACCGCAAACTCTTTCTTGTCGTGTCAAGGAAGA
GTTGTGTCCCTCTTCTTTACCAAGTCCTAAAGGCCAGCAAGCTAGAGTAAGGACTATATTCT
GTATAACTTTTGCTTAGACTGGAAAGAAGAAAACAAAGATTCATGTTTGAGAGATTACTCTG
CTTCTTTTTCACAGGTTGAGGTTAGATTAAGGGAAGGAAGAGCAGTGAACATTCATATGTTC
TGTGGTCGTAGACCGGGTCTGTTGCTCGCTACCATGAAAGCTTTGGATAATCTTGGATTGGA
TGTTCAGCAAGCTGTGATCAGCTGTTTTAATGGGTTTGCCTTGGATGTTTTCCGCGCTGAGG
TGATCTTCTACTCTCAGTTGAAAGGTTAAGGATTTGTAGAACAGTTTTAGTAGTAACATGTTT
TCTTTTGTCTATCAGCAATGCCAAGAAGGACAAGAGATACTGCCTGATCAAATCAAAGCAG
TGCTTTTCGATACAGCAGGGTATGCTGGTATGATCTGATCTGATCCTGACTTCGAGTCCATTA
AGCATCTGTTGAAGCAGAGCTAGAAGAACTAAGTCCCTTTAAATCTGCAATTTTCTTCTCAA
CTTTTTTTCTTATGTCATAACTTCAATCTAAGCATGTAATGCAATTGCAAATGAGAGTTGTTTT
TAAATTAAGCTTTTGAGAACTTGAGGTTGTTGTTGTTGGATACATAACTTCAACCTTTTATTA
GCAATGTTAACTTCCATTTATGTTTCATCTTAAAGCTATGCTCAAGAATT

>AtbHLH117
ATGGAGACACCAGCTTACGATTTCGACTCACTCACTGACTTACCACCGCTTCCTCCTTCCGA
TTTCACTCCTTCCAACGCCTTCACCTTCCCCGACCACAACCTCGACTTTTCCTTCCTCGACT
CCACTCTGTCTCTCCTCAATCGGCACCATCTCTCTGAGTCCACTCGCTTGGAACAGATTTTTT
ACGACTCCACTCACACTCAGCTCTTTCACAACGACGACACCACCACCACCACCACACCGTT
TCTCCACCTTCCTGATCTCAAATCCATCGACGCCGTTGAAGAACCGACGACGATGAAGCTG
TTTCCATCTCTCTCTCCTCCTCTTCCCGCCGCCAAACGTCAGAAGCTTAACTCAACTTCCTC
TTCAACAACCTCTGGATCTCCAACTGCGTCAAACGACGGTGGTATTATTACCAAACGACGG
AAGATCTCAGACAAAATCAGATCCCTGGAGAAACTCATGCCGTGGGAGAGAAAGATGAAC
TTAGCGATGACTCTAGAAGAATCTCACAAATACATCAAGTTTCTTCAATCTCAGATTGCTTC
ACTCCGTTGGATGCCTCTCGAATCTGTCTACAACACCGCCGGTGAAGTAGGAGAAACAGAT
TTGCTCAAATCTCTGACACGTCAACAGATTCTTCAGGTTCTTGCGAATTCTCCAGGCTCACG
AAACGTACTCTCCTCTCGTGGTGTTTGTGTCTTCTCTTATGAACAGCTTCTCTCTCTCAAGAC
AATGTCGAGAAACCTCTGAATCCAACGACGTCGTTTGAAGAAACACCAAAAGCTCTCTGTT
TTTGTTTTGTTTAACGTTTCTTCTACATAGC

>AtbHLH118
CTTTTTTCCGACTCCCATATCTTTTTTTTTTCCGACTCCCATATTATTCAACTTTACAACAAAT
CCCTGACTAGAGCAAATCCCAGAGCACAAGATCAGATCCTTCTGTCTCATCATCATCATCATT
ATTATAATCATAATTACAAGAAGAATCGTTGTTCAAAATAATTAACGTATAACGACTTCTAGA
TGAATAATTTTCAGGAGAAGAAAAGAAGGCGTAGTAAAACGCCGAGAGTTTGTAATAACGA

AGAAAACATGGAGAAGTTAGTACATAAAGAAATTGAGAAACGAAGGAGACAAGAAATGGC
TTCTCTCTACGCGTCTCTCCGCTCTCTTCTCCCTTTAGAGTTCATCCAGGTCAAAACCCTAAT
TACTCTTGCTTTTAATTTCTTCCTCTTCTTCTTCTTTTTTTTTTGTTAAGGAAAAAGTCCACAA
GTATGCTTTGTCGCTTAGTTTGTTAAAAAGATTCACTAGAACTCTAACACTCTATCATCACAA
TATCTCAAGTTGACCTCTACACCTTAATTACGTTTGATTTGGTTTTGTAGGGAAAACGTTCAA
CGTCAGATCAGGTGAAAGGGGCAGTGAATTACATAGACTATTTGCAAAGGAATATCAAAGA
CATAAATTCCAAGAGAGATGACCTCGTGTTATTGTCTGGACGAAGCTTTAGGAGTAGTAATG
AACAAGAATGGAATGAGATTTCGAATCATGTCGTGATTCGTCCGTGTTTGGTCGGCATAGAG
ATAGTGTTGAGCATTCTCCAAACGCCATTCTCAAGCGTTCTGCAAGTGCTTCGTGAACACG
GTCTCTATGTTCTTGGCTACATCTGTTCCAGTGTAAATGATAGACTCATTCATACTCTACAGG
CTGAGGTACAATATCTCGAGCTTACTAACTTTACAGCAATCGAAGCATTACTAACTTTACTAA
GAATATAGAGAAATACTTAATCATAACATATATTTCTAACTAACTTTACTAAGAATATAGAGAA
ATACTTAATCATAACATATATTTCTAACTTTGTTAATGTTTTCGATCTTGTCGGCTTACAAACG
CGTTTATGTCCTAATTTGGAATGGAATATAGAATATAGCATACATCTTCTTCATAGCTTTTTGG
AATGGCTTATTGTATCAGAGTAATGAATATATATGTTTGAGTTAATATGTGAAGTTGATTTGCC
TTATGTTG

>AtbHLH119
TGTTTCTGTTCTCTTCTCTTCTCAAAATTTTTTGTTTCTTTTTGTCTCAAGCTATGAACTTCCA
TACTTTGAATCTTCTACTTCTCTTTCTTCCTCTTACTTGATCTTCCTATAGCATACTTCCATCAT
GTAAGTTCCTTTTTGTATTTTTATTTCTCTTACTTGCTAAAGTTTTATCTTCACATTTTTTGATG
ATTAGGGTTTTGGTTTGAGATTAGGGGTGAAGATGATATAGTGGAGCTCTTATGGAACGGCC
AAGTCGTTCGAACCAGCCAACCACAAAGACCTTCCTCGGGTAAACCTTCTCCTACTCCACC
CATTCTGCGTGGTAGCGGAAGCGGCAGCGGAGAAGAAAATGCTCCCCTTCCACTTCCACTT
CTACAACCTCCACGTCCCCTGCATCATCAGAATCTCTTCATTCGGGAAGAAGAAATGTCTTC
TTGGCTTCACTATTCTTACACTGGTGTCACGTCCACACCGGCTACTCATCCGCAAAGTTCTG
TTTCCCTGCCACCACCACCACCCATTGCTCCGTACATTTCCGTTCTTTTTCCTTTCATTCTCTT
ACTCTTTGCAATTTGACGAGTCTGATATTTTAGGGTTTGGATTTTGGTTTGAAATTAGGAGTG
AAGATGATGTGGTGGAACTCTTATGGAAAAGCGGCCAAGTCGTTCAAAGCATCCAGACACA
GAGACCCATTCCTCCTCCCATTTTCCGCGGCAGCGGAAGTGGGGGAGGTGAAGAAACTGTT
CTGCCTCTGCCTCCCCTGCATCCGTCTCATCAAAATATCTTCATCCAGGAAGACGAAATGGC
CTCTTGGCTTTACCATCCCCTCCGTCAAGATTATTTTTCCTCTGGCGTCGCCTCTACTTCAGC
TACTCGCCCGCAAAGTTCCGCCTCTCTAGCACCAACACCACCACCACCCAGTGTTCCCTAC
GGTCAGATTCCCGTTGAGAGAAGGACAGAGAATTTTATGAACTTCTTGAGGCTAAGAGGGA
ACATATTTTCCGGCGGTAGAGTTGAAGCTGGTCCGGTGGTTATAGAATCGACGCAGATAGGC
TCAAGCGCGACCCCGTCGTCTTCAGCCGCTGAATCCTGTGTAATACCAGCGACTCACGGCA
CCGAGAGTAGAGCGGCGGCCATTACCGGAGTATCCCGTACTTTTGCAGTTCCTGGTCTTGGT
CGGAGGGGAAAGGAGGTGGCAACCGAGACGGCCGGAACATCATATTCAGGGGTAAACAAG
GCCGAAACAGAGCGGGTTCAAATACAACCGGAGAGGGAGACGAAAATTACCGAAGATAAG
AAGAGAGAAGAAACCATTGCAGAAATCCAAGTGAGTCACAGCTACCATAGGTTCACCTGTT
GACACGTGCAGTGTAGCAGTCAAGGCTTAATTTTATCTTGTTTCTTGGCGTACAGGGAACTG
AAGAAGCTCATGGTTCAACGTCTAGGAAGAGGTCACGAGCTGCAGATATGCATAACCTCTC
CGAAAGGGTTCTTATATTTTACTTTCTAGGTTTTAATAATGGACTCTTTGTTTCGTCTCTCCAT
TAAAACGTAGTTTGTATAATTGTTTAACAGAGACGGAGGGAAAGGATCAACGAGAGGATGA

AAACTCTGCAAGAACTCCTTCCTCGCTGCAGAAAGGTCTCTCTCAGTTATTTATAACCTGTG
GCTGTTAAACATAAAAAAAGTTAGATTCTGATGACATCTGTTTCTATTCTGATCAGACTGATA
AAGTTTCAATGCTTGAAGATGTTATCGAGTACGTGAAATCGCTACAGCTGCAAATACAGGCA
TGTACTCAAAAACTGAATGTGTGTTAAAGCAAAACACAACCGGAAATTAAGTGCTGTGGAA
TAGTTGAACATGGTTATTTGAGTGCTATGGAATGGTTATACTCTTTGTTTATCAACCAGCGTC
TCTGCATTTTAAACAACCAATTTTTTAGATTTGATTTCTGCAGATTCATCTTGGAAGGGTTTT
AAAGTGTCATGGAAGATAAATGCAAATCTTATAAGTCATGTTCTTGAGGTCTTACTCTTCTGT
ATTGCTTCACACGTTTACAGGATTATGTTGAAGAATCTACAGCTTATAACTGAGATTTTAATAT
ATGAATCAATGTTGTTATGCCAGATGATGTCGATGGGACATGGTATGATGCCACCGATGATGC
ATGAGGGGAATACGCAACAGTTCATGCCCCACATGGCCATGGGGATGAAGGGTATGAACCG
GCCTCCTCCTTTCGTACCGTTCCCCGGCAAAACTTTTCCTAGGCCGGGTCATATGGCAGGTG
TAGGTCCATCATACCCAGCATTGCGCTACCCTTTTCCAGACACACAAGCCTCTGACCTATCC
AGAGTCCATGTACCAAGTCTGCACTCTAACCCGGTGCCAAACCAGCCTCGGTTTCCGGCTT
ACATAAATCCCTATAGCCAGTTTGTTGGTCTCCACCAGATGCAACAACCTCCTCTTCCATTGC
AGGTAATTCTTTCTCAGTATCTATTGCCGTTATTCTGCCTCTCTTCCTACGAATGGGTGATGGT
CATCAATAACCTTTTTTCTAAATGTTCTTGTAGGGTCAACCAACATCACAGCCTAGTTTCAGC
CACGCAAGTACTAGCAAGTAACTTGGGGATCAGGACAACCAACCAACAAGTTAGTAGGAA
TCAGGAACAGCCAGAGGCTTTTCTTGCCGGATACATTAATGGTCAAGATTCTGACAGATGGA
TCTCCATTGTGTTATTGCAAGTTGATGTTGAAGAGTTAAAAAAAAAGGATCTGTTCTCTAAG
AAAAGCACAATTTTGAAGGTGCTAACATGCAAATGTTTTTCAGAAAATGAAAATCTATTTCT
TCGGAG

>AtbHLH120
AAACATTACAGCAAACCAAACTATTTGACTAAAGATGAAAACAACACCTCTCCCGAGGCTG
CATTATTTAGTAAGTCTTCTTTGTTTTTTTCTTTCTTCCAAGATTAAAGAAGACAGACCCAAT
TATGTAAGGGCAGTCTCTCCTATAAACCTAACTAGTTCATTGGAAAAAACAAGAGAGAAAA
AAAAACGATTACTGCTGAGATCTACCATTTCTAAACCTCAACCCATGAATCCTTCTAACAAT
CCTAAAAAGACAAGGCACCAGAGTCATATGCCTCAAGAAAGAGATGAAACGAAGAAGGAG
AAGAAGCTTCTCCACCGCAACATCGAACGACAAAGAAGACAAGAAATGGCGATCCTTTTC
GCCTCTCTTCGTAGTCAGCTACCTCTAAAATACATCAAGGTACATATATTTACTCCAATAGTC
TCATTGAAGTCCCAATCTTCCTATCAAGTTCATTGATGGGAGATTTTTGTTCTCTAGGGTTTT
AGTTTTCTCAGGCTCTTTCTTCGCAGGGAAAGAGAGCCATGTCAGATCATGTAAACGGAGC
AGTAAGTTTCATCAAAGACACGCAAACAAGGATAAAAGATCTCTCCGCAAGAAGAGACGA
GCTAAAGCGAGAGATTGGTGACCCTACTAGTCTAACTGGATCCGGATCCGGATCTGGATCAA
GTAGATCGGAACCAGCTAGTGTCATGGTGCAACCGTGTGTGAGCGGCTTCGAGGTGGTTGT
GAGCAGCTTAGCATCAGGTCTCGAGGCTTGGCCACTCTCAAGAGTTCTTGAAGTTCTTCAC
GGGCAAGGACTTGAAGTCATCAGCTCTTTAACAGCACGAGTCAACGAGAGGCTCATGTACA
CTATACAAGTCGAGGTACGGTTACATATTTGGAGTTATTGTAGAGTTTTTAGTGTAAGCTTA
TCTATTGTGTAAATATGCTTATTCTTATGTATCCATATATATGATATATCCACAGGTAAATAGCTT
TGATTGCTTCGACTTAGCTTGGTTGCAGCAGAAGCTAATTGAGCAGTTAGTACTTTCCACGA
CTAGGCACTGAAAAAACTAACATTTGTCATAAGAATATGTTCATTGAATGATGCTTTTGTCCC
AATCTCTTTGTTTTAGTGTCCGTAAGGGTGTGTTCTTTTGGTCCTTCACTACTTGGAGTGATT
TTTTTTTTTTTTTTTGTAAAAATGATCTGGTTGATTAGATTGAATTAGCAAATTCTAAACTTTC
ATGCATGCTTCTTTATATAGGAGAATCTTTTGATCACCAACAAAACAAAAATATAGTGATGCG

CCTGATTTCTTTATACATTTTTGAATATATATATTCATCAATCGATGATAAAGACTAGAGTCTCA
TCGGTGTCTTTTTATACTTCATATGCATGAACCATAGTTGATAGATATTTTGAACATAGCATAC
ATACACTTCTCTGAGACTTACCAATGAAGATATTTTATGAGAGGAGCATGGTAATGCAGGTT
AAATAATGGAGCAGCTGCCATTCAACATTAGTAGATATCTTGTGAGTTTTACTGACTAAATTC
GTACGACATTGACCTATAGCTAGGAACAAACACTGATCAAAAGAAACTTTCATTTGATTAAC
CCGCATATATATTAGTAGATAATATGGCATGCACATAATTTGATTTGACTCACGGTTTTCAAGT
TTTCTAATCATGCCATATTTTGTTCATAAATGAAATTACATGACATTACTGAAATATATGGAAA
GGAATACTGAAAACGTTTTTTTGGGTTAAGAAGAAGAAGAAAGTACATTCAAATATTCATCT
ATATTTTATAGTGACGTATATATAACTCTAGGTTTCTTTTGCCTCTTCGGCCTCAACCAGAAGC
ACTGAAGCACTGAAGCACATAAACAGTATCGCATAACTTCGGTCATATATATTTTGCCGTTGA
ATAATTTTTCTTAGATTTATGTAAAATTATGGCTTAAATACATCTTAATTATATAAAAATTAGCG
AGGATGGCAGTCGAATGTTAAAGCAATAAAGATACGATTGAGTGGTTGAGATCTCCCTAATA
TACTTTTGATTTAATTATTGTT

>AtbHLH121
AAATTCTACACAATTGTTTTTTTTTAAAAATACAGAATTGTAGATAGAATGAATATTGTTTTTT
TTAATTAAAATAATTGAAAAAATGGGGATAAGAGAAAATGGAATAATGCTTGTGAGCAGAG
AGAGAGAGCGAGCGAGGAGGCTAGAGAATCGAGAATCGATCTTCGCCGAACCACCTTGTC
TTCTCTTAGCTCATCGAATCTCTCCGTCGCCGTCGATTCTTCCCGCCGGTGAATCTCTGCCTT
ATTGTTTTCTTCAATTTGATCGTCCTGAATTCATCGTCCTATTTAGGGTTTCGATCACAATCTG
GTACTTCTCTTAATCAAAGGAATTTTTATTTTGATTTTGAGGGTTTTATTGGTTTGAACTTAGC
TCTCTAGCTCGTGAAAGTGTTGTTAGTTCGTCGTGGAAATCAATTCCTTCCCTGTGTTCTTCT
TCAACAACGTTTAGTAATTTTTTCGTAGATGTTTAAATCTCTAACTTGTTGATAGAGATAGAT
GTTATTATTGCCAAATTAGGTGTACAAAAGAATGTGGAATTTTCTGTTTGAGATTAGCTTCTC
TAGTAACCTTCTTTGCAATTCATGATGCCAATTGCTAAGTATGTAACTATGTCTGGTATGTGA
GCTGGGATCATATGATACCTGACTATCGACTCAAAGAGAATTATGTTAAGTCAATCAAGGCTT
AGTTCTGAGGCTACAGTGTGTGTGGTCGATGTGCTGTTATGGTGGAATTGGATACTAAAATA
TGTCTAAGAGTGGGACTGCAATATGACCTTCTTAGTTTATTCGATGTTTTGGTTGCAGAAGA
GGAGGTCATGGACGTTTCTGCTAGAAAGTCACAAAAAGCTGGGCGCGAAAAGTTGAGGAG
GGAAAAACTGAATGAGCATTTTGTTGAACTGGGAAATGTACTCGGTATAGACTCAATGGTTA
TGTGGATTTTTCTAAACAGCAGATGATCTCACTTTGATCCTCACATAGCTCTTCTGTTTGCAG
ATCCAGAGAGACCCAAGAATGACAAAGCCACGATTCTGACTGATACTGTTCAGTTGTTGAA
AGAGCTCACATCTGAAGTCAACAAACTGAAATCTGAGTACACCGCATTGACAGATGAGTCC
CGCGAGGTTCGTACCTTATCCCTTACGCTCCAGGCTTTCCTAACAGAAAAAATTAGCAGTC
TACATGACGTAAAGATATCGATAGGGATCCCAATATAGATAACTCATTGTCTTTTTGCTTTTAC
GATATGGTGACTACACCTATTTCGTAAGATGCAAACTCAGACGGGCTATATAATGTAACTGCT
TATAGGACTTGTTGTTATCATCAATAGCAACACTTACAAATTTACTGGTACTTGTATAACAAC
GGTTCTTTCTGTAGTTGACACAGGAGAAAAACGACCTGAGAGAAGAAAAGACATCGCTGA
AATCAGATATAGAGAATCTCAATCTTCAATACCAGCAGAGATTAAGGTCAATGTCTCCATGG
GGAGCTGCGATGGATCACACAGTCATGATGGCTCCACCACCCTCCTTTCCATACCCTATGCC
TATTGCTATGCCTCCCGGGTCAATCCCAATGCATCCATCAATGCCATCTTACACATACTTTGG
GAACCAGAACCCTAGCATGATCCCAGCTCCATGTCCTACATACATGCCCTACATGCCTCCTAA
TACAGTCGTTGAGCAACAATCCGTGCACATTCCACAGAACCCCGGTAACCGTTCTCGGGAA
CCTAGAGCAAAGGTTTCAAGAGAGAGCAGATCTGAGAAAGCAGAGGACTCCAACGAAGTT

GCAACACAACTCGAATTAAAAACCCCTGGATCTACTTCTGATAAGGTAGAGAGACTTAAAA
CTAGAAGCTAGAGATCTCCAATTTTTTTCGGATTTATTATCTTCACAGTTATTCGCGAAATTGT
ATTTTGAAGGATACATTGCAAAGGCCAGAGAAGACAAAGAGATGTAAGAGAAACAACAAC
AACAACTCAATAGAAGAAAGCTCTCATTCTAGCAAGTGTTCATCTTCTCCGAGCGTACGAG
ACCACAGTTCTTCCAGTAGCGTAGCTGGTGGCCAAAAACCTGATGATGCAAAATGATTCGA
AAGAATCTGATGTTGATCATCTCAAGTATCCAAGTATCGTTTCGATGAGTACTGTATATAGTG
CGAGTACAAAATGCACTTAGCTGTTTAAAGCAGTGTTTTGATGCACCGTGGCATTCGTTTTC
CTCGGATAGTCATTTCTCAGATGATTTTCATCCTTAATAGGTCTGCTTTAGTTCTAAAACTCG
GATGATTTGTAATTTCCAGTGTCCAAATCTACTAATTTTATTAATCCTATAAATTAAACAAACT
TATATCTTGATTTTTTTTTAAAAATCATAGATTATTTATACTATTGTACAAAAAGAATTACATTT
AAATCGGTAAATATAGTTTCAC

>AtbHLH122
AAATCCCAATCTCTACCTGCCCTCTGTTGTTTCTTCCTCTCCTCTTCTCTCTTGCACATAAGTT
TCTCTCACGTTCTCTTTTTTAATTTTAATTTCTCGCCGGAAACAATCTCATCTCCCGGCGAAC
GAAACTTCCGGTGTGGTACTGCAAACGGAGAAAAAAATAACCAAAGAAGAGAGAAACTC
AAAAGCTACTAAGATGGAATCAGAATTCCAGCAACATCACTTCCTTCTCCACGATCATCAAC
ACCAGAGACCAAGAAACTCAGGATTGATTCGTTACCAATCAGCACCAAGTTCGTACTTTTC
GAGTTTCGGTGAATCAATCGAAGAGTTTTTAGATCGACCCACAAGTCCTGAAACTGAGCGA
ATCTTATCTGGCTTTTTACAAACCACCGACACAAGCGACAACGTTGATAGTTTCCTTCACCA
TACTTTTAACAGTGATGGAACTGAGAAGAAACCTCCGGAAGTTAAAACAGAGGACGAAGA
TGCTGAAATTCCGGTGACTGCGACGGCGACGGCGATGGAGGTTGTTGTTTCCGGTGATGGT
GAAATCTCAGTGAATCCTGAAGTATCGATTGGGTATGTGGCTTCGGTTTCGAGGAATAAGAG
ACCAAGAGAGAAAGATGATCGGACTCCGGTGAATAATCTAGCTCGTCATAATAGTTCACCG
GCCGGATTATTTTCATCCATTGATGTTGAAACAGGTTTGACTCTTTTCTTTCTAGTTTTAATTA
GAAATTATTTCCTAATCTGGAAACTTTTTCTGGTAACTTTTATGAGAATATACTTAGGATTTTG
ATAGGACTATAATGATTTTAAAGCAACTTGCTTGTTTTGGTCTCTGTAGATTGGATTCTATTGA
TTTGCTATTTATTACAGATATTATCTGCAACTAGTACTTTGTTAATCACTAAATTTGGTGATGA
ATGAAAAGTGAAGTCTTTTTGAATCTTTTGTTCTGTTTTTTCTTGGAAATTTTGCAGCTTATG
CAGCTGTAATGAAAAGTATGGGAGGTTTTGGAGGAAGTAATGTGATGAGTACAAGCAATAC
TGAAGCTTCGTCTCTTACTCCTAGAAGCAAGTTACTTCCTCCTACTTCTAGAGCGATGAGTC
CGATCTCTGAGGTTGATGTTAAACCCGGTTTCTCGTCTAGATTGCCTCCTCGGACGCTTTCC
GGTGGGTTTAATCGTTCTTTTGGGAATGAAGGGTCTGCTTCTTCCAAGCTTACAGCTCTTGC
TAGGACCCAATCTGGAGGTCTAGATCAATACAAAACCAAGGTATGAACTTATAATGAGAGA
CGTTTTGCCATTGAAACCAACTTCTTGCGGTTGAAAATTCATTCTGCTATTGTTCTTGTTTGA
TTGATTGTACTAAAGAAATTCAATTTAACAGGATGAGGATTCAGCAAGTAGACGTCCTCCTT
TGGCACATCACATGAGTTTGCCCAAGTCTTTATCAGATATTGAACAGTTACTGTCAGATTCTA
TCCCATGTAAGATCAGAGCCAAGCGGGGTTGTGCAACTCATCCTCGAAGCATAGCCGAGAG
GGTAAATCTTTCCCCTTACTATTATGCTCTCAGTGGCATTGATTCCATGATGATATAATATCAG
TTTCTCCAACGATTATATATGTTTCCAAAATGTTGTGGAACAGGTGAGAAGAACCAAGATCA
GTGAAAGAATGAGGAAGCTGCAAGACCTTGTTCCAAACATGGACACGGTTAGATTATGCGA
TCTTCCTCTCTCATATTCGTACTTTCATGTTCTTTGCTCAATGTATTTATCATCTTCAATTGATA
TGCAGCAAACAAACACAGCAGACATGTTGGATCTTGCGGTTCAATACATCAAGGACCTGCA
AGAACAAGTGAAGGTAATGATCTCACTAGTGTTGCATACTAATCACTCTTGAATCCCTTTGT

TCACTCTGTAAGAAACTGAATACTTTTATTTTTGGTCAGGCGCTCGAAGAGAGTCGGGCAA
GATGTAGATGCTCTAGTGCGTGAAACTAGAAGTGGGAGTATGCGCGAGTGCTAGCCAGGGA
GGGAGTTGTGCATAGAAGTATCGGTTCGGCCTTTGGAGAAAAGTCGAAGATAGCAAAGTAG
AGAAGAGATCATGAACAAAGCTAAATTTGGTGGTGGTGGTGAAAAGGGTTTTTGTAAAGTT
GGAACCTTTTTTTGGTAGGGAAGAAAGTAGCAAGGTTGTGTAATGGTCCGAACTCCAATGC
TATTGTATGTTCTTACATCCAAAAAAAAGAAAAGCAGAAGAGAAGTGATGTACAATGAGAA
GCTTATTTTATTGTAACTCAAGAATAATTCAGGTAATTAGTTTATTTTCTTTTGTTAGAAACTT
AGAATCATC

>AtbHLH123
ACGTGGGCAAGAGAGTCAGCGAGAGCCACCTGAAAAAATGTCAACCAAACATTAATCGGA
TGTCTTCACTCTTCATAAATAGTTCACGAAAACCCTACCAGGAAAAAATATCCAATTTAAATT
GATAAATATTTATAACTATAAGTATCTCTCTCGTGTGGATCCTCATCATTTCATGTAACCACAA
TCTGAGAAGACACAACAAATTAAGATCCAACATCAATACACACACACATATATATATATATAT
ATATATAGTGAGAAAAAAAGAAGCTATGGGAGATCATCATGATTTCATCAACTCAGGAAGCT
GGTGGAAAGTATCTTCTTCTTCTTCACCATCTTCTTCTTCTTCCATGAGAGCAAGCTCTATTG
AATCTGGTGGTTCTGCTGTTTTCCATGATAAGCTTCATCATCATTCTTTAGCTACTGATCACCA
TCTTCAGATGATTGGTTTAGGACTTTCTTCACAATCACCTGTTGATCAATGGAACCAATCTCT
CTTGTAAGTCTCAATTTTGTTTCTTTTTTTTTATTTGGTTTCAGTCATAAGATTTATGTGTTTTG
TCTTTGTTTTCTTTTTCTTTGCGGGGAACAAAAGACGAGGAGATAGTAAAGCGGAGACAAG
CTTTGGTGTGATGCTTCAAGAGAATCTCAATTTAGATGCCACTTCAAACGCAAACGCTAACA
CAACGTCATCCACATCTTCTTACCAGCTGCAAGAATCTGATTCCTCTCACCATCATCAAGCTT
TGTGGCGAGATCCACAAAGCGATTTCAAACCGCAGATTTTAACGAGTGGTGGTAATCGCGG
GTTTTTCTTAGATCATCAGTTCAGTCCTCATGGTAGCTCAAGTACCGACAGTAGCACAGTAA
CATGTCAAGGTTTCGCTGTTGACAACTCGTCAAACGCCATGTACGCAGCAACAACAACAAC
TCCTAATTCATCTTCCGGTATGTTTCATCATCAGCAAGCAGGTGGTTTTGGCTCTTCTGATCA
ACAACCGTCGAGGAATCATCAACAGTCATCTCTTGGCTATTCTCAGTTTGGATCATCCACCG
GAAACTATGATCAAATGGCGTCAGCGTTACCATCGACTTGGTTTTTAAGATCTTCTCCGCCG
CCAAAACCACACAGTCCTTTGAGATTCTCTAATAACGCAACGTTTTGGAACCCCGCGGCTG
CGGGAAACGCCGGTGCTCCTCCTCCTCATGACGCGTCTTCTAACTTTTTCCCGGCGTTACAA
CCGCCGCAGATTCATCCGCAGAGTTTTGACGAACAACCAAAGGTATATATTTATAGCTACAC
GAACTACTTTATTTAGATTTATGGATATGATCTATATAATTTATATGTATTGGTTGGTCGTAGAA
TATATCGGAGATTAGAGATTCAAGTAGCAACGAAGTAAAGAGAGGAGGAAACGATCATCAG
CCGGCGGCGAAAAGAGCTAAGAGCGAAGCAGCGTCTCCGTCACCAGCTTTCAAGGTATTTT
GCTTTGTTGATATTATTTTTTAAATAAGGGGAAAATAAACAATAAAGCTTCTTTTTGGTATGA
AAATAAAAGCATCTTTAACGACATTGAAATAAAACCAGCTTTCCATGGTTTTGCCAAAACAA
TCCGAATCTTTCTATTAAACAAAACCGAAACCCTAATTTGAACAATAAAATTAGGTTTATCAT
TTACCACCAAAGTATATGCATGATAGGATATTATGAAACATTCTTTTATTGCGAAGTTGTTTTA
TTTTATATCAAGAAAACAAACATTCTTCCCACAATTCTTGCATAATTGCTTATACTAGATCTCT
TTAGTTTTTTTACTTCATAGTATTTTTTTTATAACAAGGATTTGTTTTGATAAAATTTTGCTGAA
TTAGAGGAAAGAGAAAATGGGGGACAGAATCGCTGCGCTCCAACAATTGGTTTCACCTTTC
GGAAAGGTACTATCTCTCTCTCTCTCATTGTCTGCAAATTTTTGCTTTACCTTTCAGAAAGAT
GATGAGTGTGTATTAGTATATTTGTATACTGTCATTATTACTTATCATGTTATTGTTATTATATCT
TTATGGATTTATTTTATATGTAAGGTCTATTTCAATAGATATTTATGTTGGAGATGAATGAATGA

TATACCATTTCTTAGATTTTGTTTTACTGAATCTCATGTGCTTTTGTGTCAGACTGATGCAGCC
TCAGTGCTCTCTGAAGCCATTGAATACATTAAGTTCTTACACCAACAAGTTTCTGTAAGTTA
GCCCACTCCTTCGTATTTTTAGTACACAAACATTTTCTGCTACAGTTTAGTTAGTTATTGTATT
TCTTCTACTGATGTACAACAAACCTGCAAATGATTATAACTCAGGCTCTGAGCAACCCATAC
ATGAAAAGTGGAGCTTCTTTACAACATCAACAGGTAATATTTTAATTATATAATTTTATGTATA
CATTATTAAACCATTATCATACTGTTATTATTATTATACCATTGAATAAACGTATGGCTCTCGTT
CGCATTCTTTATTACATGTATTTAATATGGTTGCTTCATCTTTAAGATTGGACAATAAAGCATT
ATTCCAAGAAACAATGTAGCGAGACGGTTTTTGCAACGAAAAAAGAAATCATTTGCAACTT
ATATTTTTTCTTTGTTTTCTAATGATGATGCAGAGTGATCATTCCACAGAGCTAGAAGTATCA
GAAGAACCAGATCTTAGAAGTCGAGGTTTATGCTTAGTGCCAGTTTCAAGCACATTTCCAGT
GACACACGATACTACAGTAGATTTCTGGACTCCTACATTTGGTGGGACTTTTAGATAGATATA
TAGACGGAAAATCGTATATTAGATGTCGGGTAACGTACGTATCTTTTTTAACTTATAATATATA
CGTTGATTGCTGATAAGAATCACCGCATTTTTAAACGAAGAGGAGATAAGTTAATGGACAAG
TGAAACGTGTTGTCCATTTATACTTCTTATTCATAGAACTAAAAAATAATGTGTAATTGAAGA
ACCATGCATAGATATATAAGAGCTATTGAGATTTGACATGTATTTTTTCACACGACCAAATTAA
TTCCAAACATATATCTATAGGTTTTTTTGGGGGGTTATAATAGTTATGTATGAAAGATAGATGT
TTTTTTTTTTTTTTTTTTTTT

>AtbHLH124
ATAAATGAAATAAGCGAATATACATATATACAACCGACTATATATATATATATATGCTGCTCTTC
ATTAACCCCAAGAAAGAAAACCAAAGTGTGAAGTCCGAATCTCTCTGATTCTACAATTCAC
AAAAACCGGAAAAAAAAAAAGACAAGTAAAGAAAGCTTTGTTCAGTTTACTTCAATGGAA
GCAAAACCCTTAGCATCATCATCATCTGAACCAAACATGATTTCTCCATCATCAAACATTAAA
CCAAAGTAAGTTTATTATGGTTTCATCTACACTTTTTGCGACTTCTATAAACATATACACATGC
GTGGTTTTGAAATAATGGGCGCTTATGGTTCTGTCGGTGCTTGAAACAATTTTGTCTTCTCCT
GACTATTTTTTTTTTGTTAATAGGTTATTTATACGTGACTCTTTTCTTGCAGATTAAAAGATGA
AGATTATATGGAGCTGGTGTGTGAAAATGGGCAGATTCTTGCAAAGATTCGAAGACCAAAG
AACAACGGTTCTTTTCAAAAGCAACGTAGGCAATCTCTCCTGGATTTGTATGAGACCGAGT
ACAGCGAGGGTTTCAAGAAAAACATCAAGATTCTTGGAGACACACAAGTTGTTCCGGTGA
GTCAGTCTAAGCCACAACAAGATAAAGAAACCAATGAACAAATGAACAACAATAAGAAGA
AGCTAAAGTCCTCCAAAATCGAATTTGAGAGAAATGTTTCGAAAAGCAACAAATGTGTTGA
ATCATCAACATTAATTGATGTTTCTGCTAAAGGTCCAAAGAATGTTGAAGTTACTACAGCTC
CTCCTGATGAGCAATCTGCAGCTGTTGGTAGATCCACGGAATTGTATTTTGCTTCTTCATCGA
AGTTTTCTCGAGGAACTTCGAGAGATCTAAGTTGTTGTTCTTTAAAGAGGAAGTATGGAGAT
ATTGAAGAAGAAGAATCAACCTATTTAAGTAATGTAAGAGAAATTAATTAGCTGCTTGTCTT
AGTTCTTTCACATGGTAGATCATTAATTGTCTTTTTCTTATTGCTTATGCAGAATTCAGATGAT
GAATCAGATGATGCGAAGACACAAGTTCATGCGAGAACAAGAAAGCCGGTGACTAAAAGA
AAACGAAGCACAGAAGTCCATAAGTTATATGAAAGAGTGAGTTTATATGTATGAAAATTTCA
CCTTAGTTAGTAGAAAAAAAATAATGTATGAAAATTAATGCTAATTAAGGATTTGTTAACAGA
AACGAAGAGATGAATTCAACAAGAAAATGCGTGCTTTGCAGGACCTACTACCAAATTGTTA
CAAGGTTTGAGATTAACTTAAATCACATTACATTGTTTACTTAGTCTTAGAGAGTTAATTAAT
TTTCTCTTTGTGGAGATTTCTGAATATCTGTAATTGTTGATGATAGGATGATAAGGCTTCATTG
TTGGATGAGGCTATCAAATATATGCGGACCCTTCAACTTCAAGTTCAGGTATTCAAAATAATA
CACCAATTCAATAAAATTTCATTGAGAGATAATAAAATGATTCAGGTGTTAATTATTTAAACA

CAGACTTGTGTTTATATTTACATATAAATTTGTTTTTTAACATTAAAATATACTTTGTAAAGTGT
TTTTGTTATCAAATATTATTGCGTTTGGCTTTTACCTTTACGAATGATTCTTGATAAAGTCACT
AGAAACTAGTTTCATAAGAGAAACCAAAACAAATTATAAACGTAATTAAACACTAATCAATA
AACAATCAAAAGTTTATTCCTTATTTATTTTACTACAAATTTTTTTACATATATATAAGATTCTT
AATTAACATGCTCTAATAATTATTTACAGATGATGAGTATGGGAAATGGATTAATAAGACCAC
CTACGATGTTGCCAATGGGTCATTACTCTCCCATGGGTCTAGGAATGCATATGGGTGCAGCA
GCAACACCAACATCAATACCGCAATTCCTGCCTATGAATGTTCAAGCAACCGGTTTTCCGGG
GATGAACAATGCACCACCACAAATGCTAAGCTTTCTTAATCACCCAAGTGGACTAATTCCAA
ACACTCCTATCTTTTCTCCATTGGAAAATTGCTCTCAGCCATTCGTGGTGCCTTCGTGTGTTT
CTCAGACTCAGGCTACTTCTTTTACTCAATTCCCAAAGTCTGCGTCCGCCTCAAACTTAGAA
GATGCAATGCAATATAGAGGAAGCAACGGTTTTAGTTATTATCGCTCGCCAAACTAATGATTT
GTAGAAAGTTGATGTTTTCTCCAACTAACTAACTTTAAGCAAAAAAAAATGATCGTCTACTC
TGTGTTGTTAGTCTATGGGCTTTTGGGCCTTGATTCTTGGAACGATTTGAACTTAATTCCAAC
TATTTTCAAAGTGGATGTACAAAGTAAAATATAACAAATATAAGTTATCATAGAC

>AtbHLH125
TGGAGATTTTAGTTTCTTCGAGATCGATCACTAACCTATCATCACATCTGTCCAATTAAAGAG
GAGAAGAAACAAATCTTGGAGGCAAGTATAAAGAGCCTCACAAGCCAGAAAGTCACAACA
CTACAAGAAAAATCAAACCAAATTAAAAGTACTCATAGATATCAAATTCCAATGGATTGTGT
TCCTTCATTGTTTATGCCTGATTCAACCTACGAAGATGGATTACTATTTTCTGATTCTTTTCTT
CTTTCTCCGTTTATATCATACCAAAACAATGATGTTTTCCATTCGATCACAAACAAAATTGGT
GGAAGCAATAAGAAACGAAGTTTGTGTGATATAACATATGGTGCGAATGAAGCCAACAAAA
ATGATGATGATCGAGAGAGCAAGAAGATGAAACATAGAGACATTGAAAGGCAAAGAAGAC
AAGAAGTTTCATCTCTTTTCAAAAGACTAAGAACTCTCTTGCCATTTCAATATATCCAGGTAA
GTTATCTAGTGGGAGATATTTCATTATGATTAGCTTGAGAAATATTTTATATTTTTATCTCTATAT
ATAGTCCTTAATTACATACTAATTTACAAAACCATTTCATTTGTTAAAGTGCAAGCGAGCAGT
TTTCTTTCCATTGAAAAGGAAAGTATATAGAGAACCTTAGTTTTGAAAGCTTTTACTAACTAT
TCGATCATTCATGCTTTCGTATATACTTTTAAAACAATTTTTTTACATAATTAAATCTTTCGTAT
ATATATTGTGGTACCACTTTATACATAGAATTAGAAACTCTTACACATTCGTATTAATATCTAA
AATATATAAATTGAAATTTTGACAAATAGTTTTTTTTTTCAACGTAGTGTAATACTCGAATTTT
GGATACCACATCAAGGAAACATCTTCTTAAACTGATGAGTTCATTCTTTTTTCTTAACACTAG
TTAATATATCCAAGTACATTCTTGAATCAAATAATTGCTAGTTAGTTGTTCTAAGCATGACCAT
ATCATTTAACATACAGGGTAAACGCTCGACATCAGATCACATCGTGCAGGCAGTGAACTACA
TCAAAGACTTACAAATCAAAATCAAAGAACTCAACGAAAAGAGAAATCGGGTAAAAAAAG
TCATATCGGCCACAACTACTACTCATTCAGCTATAGAGGAATGCACCAGTAGTTTATCATCAT
CAGCAGCATCAACACTATCATCAAGCTGCTCATGTGTAGGAGACAAACACATTACTGTTGTG
GTCACACCTTGTTTAGTTGGTGTTGAGATCATCATAAGTTGTTGTCTCGGACGAAACAAGTC
TTGTCTCTCGAGTGTTCTTCAAATGTTAGCTCAAGAACAAAGGTTCAGTGTAGTTAGTTGCC
TCTCAGCTAGACGGCAACAGAGATTCATGCACACCATTGTTTCGCAGGTAAAATTGTTACTA
AAAATATAAAGAGAAAAGGTTACAAGAATTTACAAACTTTTGGGTCTATCATAGTTGCTAAT
ATCAGTTTTTAGTTCACATACCATTTTATTTGCTAATTTCTATACAAATTATAATCTGTTTGTAA
ACTACTTTTGTGGTTATCAAAAATACAATGAATATACTTACCTGAAATTAGAATTAGATAAAT
AAATTACTAATTTTCTAAAAAAAAGTTTTAGTGTTTGATCTTTTCTTGCCTTAATTTTCTAAAA
ACAAAATTGACACTAATCATCATTTCATTTCAAATTTTCAGGTGGAGGATGGCAAACAGATC

AATATTTTGGAGCTTAAGGATAAAATAATGACTATGTAGCCCCATGTACAAGAAGAAAATTA
GTTACTATGACAAATGAAAATATTACGTGGTTTGAAATTGGAATTTTACTGAATTTCAATGTA
TGCATTATTTGAGTTGTTAGATACTAAGATCATGCTATTTTAGTGTATCGGCTTACTTCTGAGT
GCTTAGATTAATGTTACAAACTAATGCTTGTTTTTCATTTTTTTTTTTGTAAAATAAAGTTTGT
TTTTCTTGGCATTCTAATTATGATATACTTCATTTTAATTACTATTATTAAATAGAAAATATATGA
TCGTTACAAG

>AtbHLH126
TTAAGGCACCATCATTCAACAAGTAAACAATAAAGGAAATTAAATTATCACAAGACAGAAC
CACAAAATCCTTTGGAGATCTGACACTGGTAAAGCTCAAAGTGTCAAACTATGGATCCTTAT
AAGAATCTTAATCCAAAAGGTTACCAGAGACAGAGACCGTTTAGCTCAGCCGGCGAGAGT
GGCGGCAGCGGCGGCTCCGGTACGGCCCATGAAACAGATGACAATAAGAAGAAGAAGAAG
CTTCTCCACCGCGACATCGAACGCCAAAGAAGACAAGAAATGGCTACACTCTTTGCTACTC
TTCGTACTCACTTACCTCTTAAATACATCAAGGTTCATATTCTTAATTACTCACTCTTTTCTCT
CAAATTTATTTACTTTTTCGTTATATCAATTGTTCGAAGAAACCAAGATTTGGTGAGATTTGG
CTGCTAGGGTTGTGAATATGTCGTTTTTTGTATTTAATTTAGTTTTGTATTTAATCTTTATTTAT
AGGGAAAAAGAGCTGTGTCGGATCATGTAAATGGAGCGGTAAATTTCATTAAGGACACGGA
AGCACGGATTAAAGAACTTAGTGCAAGAAGAGACGAGTTAAGTAGAGAAACCGGCCAAGG
ATATAAATCGAATCCGGATCCAGGAAAAACTGGATCCGATGTAGGCAAATCGGAGCCGGCG
ACTGTGATGGTGCAACCACACGTGAGCGGTTTAGAAGTGGTAGTGAGCAGCAACTCCTCA
GGCCCCGAAGCTTTGCCACTATCAAAAGTGCTCGAGACAATTCAGGAGAAAGGGCTTGAA
GTCATGAGCTCCTTCACTACAAGAGTCAATGATAGGCTCATGCACACTATTCAAGTAGAGGT
ATGGTTGGTTACAACATGTCAACATGCCATGTCAATGGAAGTTAATCTAGGGTTTCATTACAT
TTAACTATATTCAATACAGGTTAATAGTTTCGGATGCATAGACTTATTATGGTTGCAGCAGAA
GCTAGTTGAGGATTTGATACTTTCGACGGGGTACTAATCATGCAGAAACATACCATTCGTAG
TCCTAAAGAACTTCCTCATCGTATGATCTCTTTTACCCGAGCTCTTCATTCGTTATATGAGCTT
AGCTAAATAGAAACTCATTTCCAAATTATTGTCCTTTTTAGATGTGGTATTGAAGCCTTGCAT
GTTAGTGGATATAAAACAGTATTTTTTGTAGATATCATATGTTTGTTGCTCCAATTATTGTTTTC
TTGTTCATGAACTTATATATACTTAGAAGTTAAGAACTAATGTGAAAGTCATATATAATTATAA
ACACTAGAATTTCACATGGTTCTAGTTCATATATCAATGGAAGACTAATATTAGCAATGTTCG
ATATGGTGTATTTTTGTTCATGTGTTTTACAACATTATAACTATGGTTGATGTTCTAATGAACT
AATCTCACCGTATACTTGATGCAGAAAAAATCAAAGTCTTCTTGAAATTTCGTTATGAGAGC
AAGGTGCATAGGTGTTAAAGTTCATTCATGGATTGAGGTGTAGCTTTGGTTTAAACAAGTTC
AAGTGACTTCATGGGGTAGATCATAACTTCACCATGATTCGCCGCCGTAAAGACCAAGGGT
CTGATGTTTTCAGTAGAGATTTTTTTTTTCTCACGGGCAAGCCATTTTTTTGTTTCCCTTTCAT
TATAACATTTGTATCACTCCATTGTTAGGTTTTCTTGGGTTTTCTCGTATGGAAAATAAGTTT
TTCTGTAATACATATCCATGAATCATTTAGACCTGTATTAGAAGGAAAATCCGGAAGGGCATA
GTAAAATAAAGGCCAATACAATAGCATAATATATATATATCGCCTAGTTCTAAC

>AtbHLH127
ATGATGATTATATCATCACAGATTCTTCTTCTTTTTGTTCTATTTCTTCCTCCTTCTTGATCATAT
TAGAAATTCCCCTCCATCATGTAAGTTTCTTTTATCTTCTTACTTGAAAGTTTTTTTCTCACTC
TGTAATTCCATGAATCTTTTATTATTAGGGTTTTAAACTTTTTTTTGAAACTAGGGGTGAAGAT
GATATAGTGGAGCTCTTATGTAAGATTGGCCAGACACAGATACCCTCCTCCGATCCTCTTCCC

ATTCTCCGTGGCAGCGGAAGTGGCGGACGAGAAGAAAATACTCCGCTTCCGCCTCCTCTGC
CTCATCAGAATCTCTTCATCCAGGAAGACGAAATGTCTTCTTGGCCCCATCATCCACTCCGT
CAAGATTATTTGTGCTCTGAACTTTATGCTTCCACTCCGGCTCCTCATCCGCAGAGTTCCGTC
TCTCTGGCACCACCACCACCAAAACCACCATCCAGTGCTCCGTACGGTCAGATTATCGCTCC
AAGAAGTGCTCCGAGAATCCAGGTGAGTTACCGCTCCGAGAAGTGCGCACTCATGTCAGC
GCGTGTTAGTTACTTGACGCGTGCAGTTTAACCCTTTTCTTGTTTCTTGGCGTACAGGGAAC
TGAAGAAGCTCGAGGTTCAACGTCTAGGAAGAGGTCACGAGCTGCAGAAATGCATAACCT
CGCCGAAAGGGTTGTTATATTTCCATTGCATTTTCAAGATTCTTTGATTTCAACAATGGACTC
TCTGTTTCATCCTCCATTGAAACGTACCTTGTGTTTTAACAGAGACGGAGAGAAAAGATCA
ACGAGAGAATGAAGACTCTGCAACAACTCATTCCTCGCTGCAACAAGGTCTCTCACAGTTT
CACATAACTTGTTGTCATTAAACTTGATTCTCATTCTGGTTCTGGTTCTACTCTGATCAGTCTA
CTAAAGTTTCAATGCTGGAAGATGTTATCGAGTACGTGAAATCTCTAGAGATGCAAATAAAT
GTATGCCCCTGAAGATTCCTCTTTTTAGATTTGATGTCTGAAGATTCATCTTCGTTTAGATGG
TTGGCCTAATAAGATGATTCCTCTTATATGTCCTTGAGGTCTTGCTCTTCTGTTTCTTGCAAGT
CATGAATTTGTTTTACAGCAATTATGTAGATGAATAATCTTCAGATCATGAATGAGGTTTTTTA
ATATCTGAATCGATGATGTCAATGGGAAGTGGTATGATGCCACCGATGATGTATACATCGAAT
ATGCAGCAGTTCATGCCCCACATGGCCATGGGTATGAATCAGCCTCCTGCATACATACCTTTC
CCTAGCCAGGCTCATATGGCGGGTGTAGGTCCATCATATCCACCACCGCGATACCCTTTTCCA
AACATTCAGACCTTTGACCCATCCAGAGTTTGGTTACAAAGTCCACAGCCTAACCCGGTGT
CGAACCAACCTCAGATGAATCCTTATGGTCAGTTTGTTGGTCACCATCAGATGCAACAATCT
CTTCCTCCTCCATTGCAGGTAATTCTTTCCCAATATCCATTGTGCTTATTCCTCTGTTCCAACA
AATGACGTTGCTCACAAGACCCATCCTCTTTTTAAAAATGTTCTTATAGAGTCAACAAATAA
CATCACAGCTGAGTTTAGGCCAGGCAGGTAGTAGC

>AtbHLH128
TTATATACACTAGAGAAAAATAATTGCATTCCACTGAAAACCTCAACATCTTGGGAAATTTA
AACTTAATCAAAGACATCGCAAATGTTGCTTATCTTTTTATCAAGCCCAGCCCACATATTCGC
GGCCCCAAAGCCCAAAGGTTAAGCTGACTCGTTACAAAATAAATAAGTATTAATAAAGGTTT
TTGATTGGCTTATTTCGGTTTAAATTTAATACCATATCTTTCGGAAGATCACGTGAGACACAT
GTGACATCCAACGAATAGCCAATCGCTGAAAAGTGAAGACTTTTTGTTACCCCATAAACAA
AAGCATCATCTCTCTCTCATCTTTTATTTTTCTTTTAGAAACCAAACTTTTCAGATTTCTCTCT
CTCCATTTGGCGATAGAAGAAGTCATGTACCAATCATCATCCTCCACGTCATCATCATCGCAG
AGATCATCGCTTCCCGGCGGCGGAGGACTGATCCGTTACGGCTCAGCTCCGGGATCGTTTCT
AAACTCTGTGGTTGACGAAGTCATCGGAGGAGGCTCATCAAACGCTCGTGACTTCACCGGC
TATCAACCGTCGTCGGATAACTTCATCGGTAACTTTTTCACCGGAGCTGCTGACTCATCCTC
GCTGAGATCCGATTCGACGACTTGTGGAGTCAACAACTCATCCGACGGACAGAAACAGCTA
GGCAATAACAATAATAATAATAGTAATAAAGATATCTTCCTCGACAGATCCTACGGTGGATTC
AACGAGATCTCGCAACAACACAAGAGCAACGACATCGGAGGAGGAAACAGCTCAGGATCT
TACTCTCTCGCTAGACAACGTAGCTCTCCCGCCGATTTCTTCACCTACCTCGCCTCAGATAA
AAACAGTTAGTCCTTTTTTTACTCAAGAATAGTAACTGTGATGAGATATATATATTTAGACAG
ACAAGTTTACTCATTTTCAGACCATTTTTCCTTAGAATTTGCCAAAAATCGTAATGTAGAATA
TGAGCTATATAGTTTTCAGTCATTCAATCATTCATTGCTCAACTAAAGATCCGTTTTTTGGTG
AAATGAAAATCTAACCACGAGGGTATTTTCGGTATTTCTCACTGTGAAGTATCTAGTTTCTTA
CAAGTTAATTAACGACTATGTATATAAAATAAGTTGACATTGTGAGCTTTGATATTTAGGGTC

GTTGCTAAATTAGCTTGGTTGGTACTTGTGCAAGACGTGATCCTCGTATTTTCGGAATGACG
AAAATACCATTTGATCCCTAACCACGTGCTTGAAAATACAGCAAAAATGTGGCTCGGTTACC
CTTAATTTGGGATATTATTTAAATCTTCGAGGATTCCTATATCTCAAGTTGCATTTATTTATTA
GAGGATGGATTTTTTCGTTGATGACCAATAGTTTCTCTGCTGAGATGGCGAAATATCATTGGT
TGTGATGAAGATTATTCAAATAGAAAGCGATGTAGATCTAGTTGGTGGATCTGATCTGTCCA
CTTTTATATTATTTTTTCCTTGGTCCTGATGATTCGTGCTTTGCTTCGTTGTTATCTAATCATAA
TATCTTCCACCAAGCCTGGATGTGACTTCCCACACCTAACCGGTCACCGGCCTGAACCGGA
ATTCAACTATCCATATTATCCAAAAATCCCTCTTTATTTACTTACATTATATGGTTTCTAATATTT
TGGATGTGAATGTGAATTATTTTTAAAAATAATTTTCACGTAATGTCATTATAATATAGTTTACT
TTGTAAATCAGATTTCTCGTTGAACCAACCAACCAGTGATTATAGTCCGCAAGGAGGGTCTA
ATGGGGGACGAGGACATTCCAGATTGAAGTCTCAGCTAAGCTTCACGAATCACGACTCTCT
GGCTCGGATCAACGAGGTCAATGAGACCCCAGTCCACGACGGTTCAGGCCATTCGTTTTCT
GCGGCTAGCTTTGGTGCAGCCACTACTGATTCTTGGGATGACGGTTCCGGTTCGATAGGGTT
TACCGTGACTAGGCCCAGTAAACGATCCAAGGACATGGACTCTGGTCTCTTTTCGCAGGTA
CAATTTATTTGTTTCCATGTGGGATTCCTTTTTTTTTTTCTTCATGTGTTATTACTATAAACATG
CTACTTTAGTCAAATTATATGAAATTTCAAAATTTCTAGAAAAGGACAAAAAAATGTAAAAT
ATTTGTTTTAGGATAGACATAAAAAAAAAAAATTAACTAGTAAAGGTAAATGCAGCAAAACTC
TTTAGTGGCCATACATTTTTGTTGGTGGCCAATTCTTACCGAATATTTTTATCACTGGTTTTGC
AGTATAGTCTTCCTTCAGACACTTCAATGAACTACATGGATAACTTCATGCAGCTTCCAGAA
GATTCTGTACCCTGCAAAATCCGGGCCAAACGCGGCTGCGCCACCCATCCTAGAAGCATCG
CTGAGCGGGTCAGTTATAAACACTTTTTAATTTATTTAGCGATGGATAATTAATTCACTAAATT
TAGCAACAAATTTGATCGTCCCTAATCATGTAGACCCTATTCTTTTTGCCATCCTCCTATCTTA
ACCTATACAATATGTTTATATTATAGGAGAGGAGAACGAGAATAAGTGGGAAGCTAAAGAAG
CTACAAGATCTTGTCCCCAACATGGATAAGGTTACTTTATATCAAATATATATAGTATATATGGT
AACATCATTTTGAAAAAGTGTTCATTACCTCACACACTTGTTGCTGATTTTCTTAGCAAACA
AGCTATTCAGACATGCTGGATTTAGCTGTACAACACATCAAAGGCCTTCAGCATCAACTTCA
GGTTCCCTTATCTTACTTTTTCTGTTTAGGTCAGCTGAATCCATTTCCAGAGTTCTCTCTAGTT
CATGATCCATATTGATACATAAACACATTTTCATATACAAACGCAGAATTTGAAAAAAGATCA
AGAGAATTGCACGTGTGGGTGCAGTGAGAAACCAAGCTAGCTCAAACCCAAAGGTAGGGT
CATCCTAATTTTATTTGCATTGTTCCGATTTTATCATATGAGAGAATTAGAGAAGATGTAAATA
AAATAGATTATATTAAATGTCTAATCCTCTTATAATTAAGCTCCTCATTCGTTATCATTATAAGA
TAGAACAGTGACAATTGTCCATTATGTTTTTGTTTCAAGTTTATGTTTTGAGTGTCGCCTATTT
TGTAAGGATTATGCTATGAATTCTGAAACTAATTCGAGTTATGATCATAAGCAAAGCAAGCTA
GCATCCATTATATATATGACTTTCTAATTTG

>AtbHLH129
TATAGTATTAATTAATAACTACTCAAAGTCTCTTTCTCTCAAACCCTCTCCTCAAGATTTTTTC
CTAAGCATCAGATTAGCTTTTGTAGGGTTTTTCTTTTCCGGTTTCATGTACCCTCCTAATTCCT
CTAAGTCCACCGCTCATGACGGCGGCGGCGATGCCGACACCAATCAATATGACTCAGCCGC
TGGAGCTACCCGTGATTTCTCCTCTCTTGGCCCTCAAACCCACCATCATCCGCCACCGCAGC
GGCAGCAGCAGCATCAGCAGAATCCCAACCTCGTCGGCCATTATTTACCGGGCGAGCCATC
TTCCATCGGATTCGATTCCAACGCTTCTTCTTCGTCTTCTTTGTTCCGACACAGAAGCTCTCC
GGCTGGATTCTACGACCAACATCTTCCCACTGATCCCAACGGTAACTTTTTCGTTTCTTTATT
TTTCCAACTTTTTTTTCACCCCACCAAAGAGTTCTCCTCCTAATTAATTGGATTTAATTAGTG

CTGAATCTAGTCGACATAATTTACATATTATACAATTATACACATATCAAGAATTTTTTTTTAA
GTGTTCAACTTTTTTTGCACATTAAAAAATTTGTTACAACATTTAATAAATATATGTTTCCTAT
AAGTTAGACAAATTTGGTATAGAAAAAAAAACAAAATTAAAATTCCATATTAGTATTCGAAA
ACATTTTAAAAAATGAATATGTTATTTTACATCGATAAAGTCGGCAAGTAAATTGGTGTAGCA
CGTAAGTTTTGACTTACTGTAATCTAAACCATGTAGTCTTGGTTTTGTTTCGATGACTTAAGT
AATTTGTCTATAGGTTGGAATTTGTTTGGTTTCCCTCCATACGACTTTTGTTTTGTTTTAATTA
AAGACCAATAAGTATCAACCACGTAATTGAAAACCTAATACACATGTAGTTCTCAAAATATAT
CTATTTGCTTTAGTCAATACTTAATCTATCTCTAGATCTTATACACACCTTTCTCTTTCAGCTTA
TGTCTGAAGAGATTAACCATGGTTGATCTTAGTCATATGTATACATGTGTACACACGTATATAT
ACATGCATGAATAATCAAATACGTATTTCCAATAGTGGCAAGTTGTTTATTTTAATAATGCAAT
ATAAAACACACACTTGCCAAAAGTATATGTTAAAAAACGAATCGTTACAAAAAGAAATAAA
AGTATAGTCCACTTGGAAATAATATATCCATCCCACTTGCTCTTTTTCTTCTGACAAAGCAGC
CATGATGGCCTACCCCTGCTGACATGGCTTGATCATGGTCACTATTTTGATTTTTGGATACGC
CTACTTGTCATTATATATAATAAGCTTTATTACTATAGTGTTAGTTGCAAGTAATAATTTATATTA
TTTTTGTGCAATTTGATCTTCGGTTCATTGCATATGTCCGGTTCACATTCCCTGTAATCCATGT
GCGAGCCCCAAGAAATTTACCCCCTCTTGCATTATTATATACATACTACTTTGTATAGTATAT
GATAGTAATTGGAATTCGAATGGTGAATATTTCTAAGATTCTTTTTCAGGAATCCTTATTCTCT
ACCAAATTGGCGATAATTAAAATATATATAAGTAGAGAGACTAGAGACAAAGGAAGGGGTC
CTCGAGTTTCGAAGACGACCCATTCTCTTCTTATTTTGTACTAAAGATTTTTAAAATCAAACT
GGTTATTAAAATACTGAGATAACAAGTTATAAACCTACATTACATTCTTTCTTTCTCTTTCAAT
CTAAATTTGTTGATGGAAGAAGGAACAGGTTTTTCTCTAGGACGGCCAAACGGAGGCTACG
GCGGAGGAGGAGAGCAAGGGCCGTCGAGGTTGAAGTCGGAGCTGAGATTCTCTAGTGGGA
GTAGTAGCCATCAAGAACATAATTCTCTACCGCGAATCTCGGAGGTTGAAGCGGCTGCAGC
GGCTAGAAACGGTGTCGCATCAAGTAGTATGAGTTTTGGAAATAATCGTACTAACAATTGGG
ACAACTCGTCTTCTCATATCAGTTTCACCATTGATCAACCCGGAAAACGGTCCAAGAACTCC
GACTTTTTCACCTTAGAAACTCAGGTATTTTCTATAACTTTAATTTGAGGTTATTTATGTATGT
GTATCAATACTTTTAGATTATTTTTTTATACAACAAAGTTAATAAATCTCGGATTCTTCTAGGT
AAAGGGAGAAAAGAGTGAATATCCCCAATGTTAGTTGTTTTGTGTATTATTAGTTTCTATAGG
CTTGGAGAAGGTTTATGATTGATCTATAATAGGTTGACAACTTGATACTAAGAAAGTATATTA
TCATATCGTATGAAATTTTATATCAGTTTAATTAACTTCCATAAAGTTAGCAATAGAATTCCAT
GTTGAAATATTGCCAACAGCAATGTCTATGTCACAGGTTCATGTGTATAAACTGCTTGGAGA
AGGTTTATAACTTTTCACAAGTCCTTGGCAATTAAATGATTTGTGTGTTTAATTATATGTATGG
TCCACAAGTTGTATGCTAACAAGTGTTATTATATAGTGCGCGTCATATACAATATGAATTCTAC
AAGCCCAATTTAAATTCCATATCCACAACAGAATTCCAAGTTGAAATAAATGCCAATAAAGT
GCAAATAGCCAAATTTCTGTGTGTAGAAGCCAGAGTTGAGTGGTTGTGTAATAGATATAAAT
AGCTTAGAGAAGGGTTATGATTAATTTATGTATATTTTTCACAAGCCCTTTGCTACCAAATGA
TTTTTGTGTAAACATTCTCACTGATGTTGGTTTGGTTATGATATTCATTGAGTGGTTATACATT
GATTTTCATCTCAATATATATGGTTTTGACTTTTAGTATAGCATGCCGCAAACAACTCTGGAA
ATGGCGACAATGGAGAACTTGATGAACATCCCAGAGGACTCGGTGCCTTGTAGGGCTAGAG
CCAAGCGCGGCTTCGCGACTCACCCACGCAGCATTGCTGAAAGGGTTAGTTTATTCTATTAT
TATGGTGATTACTATTGCATAAGTCTAGATACAAACTTAAAAGGTGAATTCATTTGAAACCAA
AGAAAACAAGAGTAAAAATTAGGTTTTATATATGATTTGGTTTGCGTGTTTGTGGTATATAGG
AGAGAAGAACGAGGATAAGCGGGAAGCTGAAGAAGCTACAAGAACTTGTGCCTAATATGG
ACAAGGTAAGTGATATTATTTCTGTTTTTAATGTAATGTTGGTGTGGTTTTACATATCTATAAT

AAGTTTGAAAAATTTGGCAGCAAACGAGCTACGCAGATATGTTGGATTTGGCTGTTGAGCAT
ATCAAAGGTCTTCAGCACCAAGTAGAGGTGCGTCCTTAGATACAAAATGAATCTATATATGC
AAAAGTGACATTTTCTCAAATTGAAGCAAAGGGTGATCATATTCCTTCTATGTTGGGAAAAT
GTTACCTTAAAGCCAAATTTTAGTATTTTAGAAAATAGTTATATTAATATAATGGTTAGTTATTG
TCCAATATGATCATAACCATGGATCAACCATTTACTCGGACGTGTTTTTTTCTTCCAGAAAA
TTTCATGACATATCAAAATGTCATGCACGTGAAATTAAAATATTACATTCTCATGATCTCTTAA
CAAAAGGGTTTTTGCGTTGGTCTCATGAAAATGTTTTCGTTATGTTCATTTATATCATTTTCAC
CCAGTTGAAATAAAACTGATCATACATGCATCTTAAATTTTGTTAAAAACTTTCGTAATAGAA
TACGTTAAGTCGTTAACAGTAGACTCTTCTAAGAATCTAAGTTAAGAATCATGCATAATAGAT
GGTTATACATTATATACTATATATTTATCATACACTTGGTCTACGTCATGTTTGATCGGTTAGAT
TCTGATGTCCCGTTTATTCACATGTCTAGGTCTCAATGTCTTATTCAAGCTAAGTATGCATATG
GAGAAATTAATATGATTTTTTAAAATTATCTCTTATATATGTGCAGTCACTGGAAAAGGGAAT
GGAGAGATGTACTTGTGGGGCATGCAAGAAGCGATGATGAATGATATTGTTCCAAGTATGAT
GCAATAAAGTATAGAAGAAAAGGAGATCACTTACGAACCAAATTCTAAGACAGAAAGAAA
CATAGCAAAAAGGAGTTATTTGGAGGTCTAAAGAAAGTGATGATGGATTAATGAAGGCTG
GTTTTGTTACTCTCTGAAAGTCTTGACCTTTATTATAACTTAAAATTCGATCAATCGATTTTTT
TTTTCCTGTTTTCCTCAAAGTTGGTTAATTTTAGCTTAGAGCAAAACCCATTATTTGAGATTG
AAAGAGATGCGCGTTTGTGTGTGTGAATATAATCCTACGTTTTGAATTTTGATCTGTGAATAA
AAATCTCAGAAAAACATCAGGAGGATCATAGTTGTTTTCAACAAATGTATATAAAATAAAAC
CTTAATTGAACCAAATTCATTATTTCAA

>AtbHLH130
CTTTTGGATCCGCCGCTATCTTCAGCAATTAAAGTTGGACGCAGAAACTCAGAAGCAAAAA
GACGATAAATATTCATCATCACCTTCATCTCTCTCTTCCTCTCATCAGACTTCACCGTTCTTCT
TTCTCTCTTCTTCATCTTCAGACGAAACCTCCTTTACTTCCCCTTCAAGGTTTTATATTTCCCA
AATCCTACACCTGCTCACATCTCAAGCTCAAGTTTTGTTTTCCCGGGAAAAGTGACACAAA
CAAGAACTTAACAAGGGAGAAGAAAAAGCTTATATTCATATCAACGGTTGACGTTGGAAAG
CTATTAAGATTTGGTTTTCTACAAATTTGTTCTTCCTGAAACGTCACGAGACAGAGCTTACA
AGAAGAGAAAACAGAGGAAATTTCGTTGCATTTTTTTTACATATTGATTCGATTAATGGATTC
AAATAATCATCTCTACGACCCGAATCCCACCGGGTCGGGTCTTCTTCGTTTTAGATCAGCTC
CGAGCTCTGTTCTCGCCGCTTTTGTTGACGACGACAAGATTGGTTTCGACTCCGATAGGTTG
CTTTCAAGATTCGTGACCTCTAATGGCGTTAACGGAGATCTGGGTTCACCTAAATTCGAGGA
TAAGTCTCCGGTTTCGTTAACGAACACCTCTGTTTCATACGCCGCCACTCTGCCGCCACCGC
CGCAGCTTGAGCCGTCGAGTTTTCTGGGTTTGCCGCCGCATTACCCGAGGCAGAGTAAAGG
GATAATGAACTCGGTTGGTTTGGATCAGTTTCTCGGTATCAATAATCATCACACCAAACCAG
TTGAATCTAATCTTCTCCGTCAAAGCAGCTCTCCAGCCGGAATGTTTACTAATCTCTCTGACC
AAAACGGTACCGTTTTGCTCTCTGCTACAAAGTTTTTCCCCCTTTTTTTAGTCTGAAATATTT
TTTTCATTTATAACAGAAGTGTTTTTGGTACTTCTTGATTCAACTTTTTTGAATGTTTTAACAC
TTGCTTCAAGATTTTTGAATGTTTTACGTTCCTTGAGAATAATTTTAAGACAAGATCTGATTT
GATTTTGTCAACCATAGAGTTAAAGCACTTTGGACTCAACAAATCTAATAAGTGTTTACTATA
TACTTACAAATTTGCAGGTTATGGTTCAATGAGGAATTTGATGAATTACGAAGAAGATGAAG
AGAGTCCATCTAATTCCAATGGATTAAGACGCCATTGCAGTCTCTCTTCAAGGCCACCTTCT
TCACTTGGAATGCTTTCTCAAATACCTGAAATCGCACCCGAAACTAATTTTCCATATAGCCAT
TGGAATGATCCATCCAGCTTTATTGATAACTTATCCTCACTTAAAAGAGAAGCCGAGGACGA

TGGAAAATTGTTTCTCGGAGCTCAGGTAACTCGGTTATCTAACCGGTTTGTTTTGGTTATTGA
TCAGTAGCTGAAAGTGGAGTTGATTTCTTGGAGTGGTTTCTAATGATGTTTTTGATGGGTTT
CTACAGAACGGAGAGTCCGGGAATCGTATGCAGTTACTGTCGCATCATTTGAGCCTACCAA
AGTCATCATCGACAGCCTCGGACATGGTTTCAGTGGATAAGTATCTTCAGCTACAAGATTCT
GTTCCTTGTAAAATCAGAGCCAAACGTGGTTGCGCTACACATCCTCGAAGCATCGCTGAAC
GGGTAACTTCACCTAGTTAAGGCCATCTTTACATATATCAATGGAAGTCCATTTGAAGCAGA
ATCTAGTTTGTTTTCAGAAAGCCTTTCCTAAGAAATCTTGTCAATTGTGTCAGGTAAGAAGA
ACGCGGATAAGCGAGCGAATGAGGAAGTTACAAGAGCTTGTTCCTAACATGGACAAGGTAC
AGTTTTCAGGAACTTCCTTGTAATGACTTTTGATCATAAACAAATTGTTAAATGATGTTGTTT
TCTTTATGTTTTAACAGCAAACCAACACTTCGGATATGTTGGATTTAGCTGTGGATTACATCA
AAGATTTACAAAGACAGTATAAGGTAAAGTTCCTGATCATTGTGGAAAAAAAACAAAGGGG
CCTGTTTAGTCTTCTGTTGAATTGA

>AtbHLH131
TATGATTATCTCCTCTTTGGTTATTAGTTATGACAAAATATTCTCGTGCTTATTGCTCATTAATC
ACCCATTTATTATTACCGACCTTTTGGTATCTTCTCCATATCTTTCTTCTTCTTACAATGTTTTT
TAGTCTTTATATATATATATATACACAACAAATTTAACATTCCCTCCAATGTCCTACTGTCTCCT
TCTCTGTGTGTTACCATGGTTTTACTTCACCATGTAAGTCTCTCTCATTATCAAAATTCATCTT
CTCTGTTTTCTTCCTCCTCTGAATCAATCCTTTGTTTATTTCTTGTGTTGTGTGTGATGCAGTT
AGAGCAAGGGATGCGTCCGATTTCACGATGTTACAATCCAACCGCGTATTCGACAACAATG
GGAAGAAGTTTCTTCGCAGGTGCAGCCACAAGCAGCAAGCTATTCTCCAGAGGTTTCTCAG
TCACAAAGCCAAAATCTAAAACCGAATCTAAAGAAGTTGCTGCAAAGAAACATAGTGACG
CAGAGAGAAGGAGACGGCTTCGGATTAATTCCCAGTTTGCAACTCTCCGCACCATTCTTCC
AAACTTAGTCAAAGTAAGTTTAGCTCTGCATTCAATTACACAAAATGTTTCACCAGAGAAGT
AACACTTTTTGTATTATGTTCAATGAAACTAAACAGCAAGATAAAGCATCTGTGCTTGGAGA
GACTGTCAGGTACTTCAATGAATTGAAAAAGATGGTTCAAGACATACCAACCACACCATCT
TTAGAAGACAACTTGAGATTGGACCACTGTAATAACAACAGAGACTTGGCAAGAGTCGTGT
TCAGTTGTAGCGACAGAGAAGGGCTAATGTCGGAGGTTGCAGAGTCAATGAAAGCAGTGA
AAGCAAAGGCGGTGAGAGCTGAGATCATGACAGTAGGTGGAAGAACCAAGTGTGCCTTGT
TTGTTCAAGGTGTCAATGGGAATGAAGGATTGGTGAAGCTCAAGAAATCGTTGAAACTTGT
AGTGAATGGTAAATCATCATCAGAGGCGAAAAACAACAACAATGGAGGATCGTTGTTAATT
CAGCAGCAATGAGTATTTTGTTTATATACTTGTACATCTCTGTTTCTCCTAGTCCATTAGAGAA
GGTAGATGTAAAGGTATAAAAGCCCATGTGTTATTGAAATTGGGTGGATACTTACAAGAGTC
TATATGAATAAAAATGATGCAATTCTTTCTTTGGAGATGGTGTGGATGTTATAACAAAATATG
AATCATGTGAAATTTTTTGTCCCATCTTTGTT

>AtbHLH132
GAAGAAATAACTTTTGGAACATTCAACAAGACAACAAAATATGACTTCCCCATCATCCACCT
TCAGACCAAATTAAGTTCTTCAATCTTGTTTCCCTGTTTCACACACATATATATATATATATATA
TATATATATATATATGTGTGTGTTTGTGTGCAGACGATGATGTTCTTACCAACCGATTATTGTTG
CAGGTTAAGCGATCAAGAGTATATGGAGCTTGTGTTTGAGAATGGCCAGATTCTTGCAAAG
GGCCAAAGATCCAACGTTTCTCTGCATAATCAACGTACCAAATCGATCATGGATTTGTATGA
GGCAGAGTATAACGAGGATTTCATGAAGAGTATCATCCATGGTGGTGGTGGTGCCATCACAA
ATCTCGGGGACACGCAGGTTGTTCCACAAAGTCATGTTGCTGCTGCCCATGAAACAAACAT

GTTGGAAAGCAATAAACATGTTGACGATTCTGAGACTTTGAAAGCTTCTTCATCAAAGAGG
ATGATGGTTGATTATCATAACCGAAAGAAGATCAAGTTTATACCTCCTGATGAGCAATCCGTG
GTTGCTGATAGGTCGTTCAAATTGGGCTTTGACACTTCCTCCGTAGGTTTCACTGAAGACAG
TGAAGGATCGATGTATCTAAGCAGTGTAAGAGGGATTGATTCACAATTTTTTTGGTTTGGTCT
ATCTTGTTAGGTTCTTGTCTGAAGACAGGTTTTGTCTCAACGCAGAGTCTAGATGACGAGTC
AGATGATGCGAGGCCACAAGTTCCTGCAAGAACAAGAAAAGCTTTGGTCAAAAGAAAACG
AAATGCAGAAGCGTATAATTCACCTGAGAGAGTAAGTAATCTCTTTAAGATACAAAGCATAG
TGAACACTGAATCTGATACAACGGTATAATGGTATTGTGCAACACAGAATCAAAGAAATGAT
ATCAACAAGAAAATGCGTACTTTGCAAAATCTACTACCTAACTCTCACAAGGTTTATCATAA
GAACCAACTTTTTCATTTATTTCGAAAGAGATCAAAAGCTTTTTAAAGAGTCTATACTTACAT
TTCCCTAAATACTCAATATTGTCCATAGGACGACAACGAATCGATGTTGGATGAAGCAATCA
ATTATATGACAAACCTTCAACTTCAAGTTCAGGTATAATAAACAAATACTTCAAACACTTTTT
CACATCAAAAACAGATTCAATCTAAAGATTCTTTGTCCTAACCTGTATAAATGTTGGATGGTA
GATGATGACGATGGGTAACAGATTTGTTACACCATCAATGATGATGCCTTTGGGGCCGAACT
ACTCTCAGATGGGTCTAGCAATGGGTGTGGGAATGCAAATGGGCGAACAACAGTTTCTGCC
TGCACATGTTCTAGGAGCTGGCTTGCCTGGGATTAATGATTCAGCAGATATGCTAAGGTTTCT
TAACCATCCTGGACTAATGCCAATGCAAAACTCTGCACCTTTCATTCCAACGGAAAATTGTT
CCCCACAATCTGTCCCTCCATCGTGCGCTGCTTTCCCTAACCAAATACCAAATCCCAACTCT
TTGTCAAATTTAGATGGTGCAACCTTACACAAGAAATCAAGGAAAACTAACAGATGAAGGA
ATTCTCCCTGGTAAAAATCTGGAAACCCGTGAGCAGATGAAGTAAACAGTGATGATTGTTA
CCTGCGGATTCACGGTCTTCTTCTTCATGAGTTAGTCGTGAGAACACTAGAGCCATCTCAAG
GTGAACCATACAGATCCACAAAAGCACTACAACGACATCAGAACAACTACCATGGAAGGAT
CTCCTCAAGGTTCTCTTTTTTAGTGCCGGCTTTTCTAGTTCATGTAACATATAGATATAGGAA
AGGAGCTATAATAATAACAATAATAATCACGAGTACTAGTTTCCTTCGAATGAATAAGACTGC
ATGTTAGAAACATTAATAAATTATCGAACACTATCGGAGTGATAAAAATCATAACTCGACGTT
CCTCTCTGTTTATGAAACAAGCTGCTGACTTTATACAATGGAGTGAAGCTATTGTTTCTAGAA
TCCAAACAAGAGGCATAGCATACAAATACTCAACTCACTTTACCCGGTTCTATTCTATTTATA
TGATACTGAGTGTATTTCTTCACTAAGAACAACAGGAAGAGAAGAGA

>AtbHLH133
GGAAAAAATAAAAAATAAAAACAGAGAAAAAAAAATCGAGAGAGGGAGAGAGAATGGTGT
TAAATACATAAAAAACCCATCATTGACATGTATCGATTGCTCAATTTGTTTGTCTCTCTCTTCT
TCTTTCCTTTTCTCCAAAAACTAGAGCCTAAACTAAAGCATCGAGAAACAACAAATCTTAA
AATCTTTTCATATTCTCGGTTAGTAGAAACTAGGAAAAGAGATGAATAGAGGAGTGTTGGAG
AGTTCGCCGGTTCAACATCTCACGGCGGCTGGAAACCCTAATTGGTGGAATAATGTCAGCC
GCGGCTTGAGGCCACCAACGCCGTTGATGAGTCACGAGCCGCCGTCTACCACCGCTTTTAT
TCCAAGTCTCTTGCCAAACTTCTTCTCTTCTCCGACTTCCTCTTCATCTTCTTCACCTTCTTT
CCCTCCTCCTAATAGTAACCCTAACTTCTCTTCTTGGCTTGAAATGAGTGATCTGCCTCTTGA
TCAGCCATGGAGCCTTAGTCAACTCCTCTTGTAACTCCCTTTCTCTCTCTCTCTCTCTCTCTC
TCTCTCTCTCTCTCTCTCTCTCTCTCTCTCTCTATCTATCGTTCGGATGTTTTCTATGTATAAAC
GGTTTTGAGTTCTTGGATTTGTTTTGTAATCTAATTCGATCTTATAAAGTTTCTTTCTCTTTTTT
TTTTCTTTCTCATGTATACATAATCTACTTATATTATTTCATATAAACAAACATGATCTAATATGG
ATTGTCTTTGAATGAAGGGGTGGATTGATGATGGGAGAGGAAGAGAAAATGGAGATGATGA
ACCATCATCATCATCAAAATCAACACCAAAGTTACCAAGCGAAGAGGATACAAAATTGGGA

AGAACAAGTTTTGAGGCACCAAGCTTCCATGAAACAAGAGAGTAGTAATAATAATAGTTAT
GGAATAATGTCCTCACCAAACTCACCTCCAAACAAATCTTGTGCTACAATTATCAACACCAA
TGAAGACAACAACAATAACATTCACAGTGGTCTTAACTTGTCCGAGGTGGGTCAAAATAGT
GAATTATTCAGATCAAGATATAATCTTTACCAAATATTAATTAACATCTCCATATTTTTTCTTTA
TCAATGAATATATCTGAATTTTGTTTCTGTCTATGTGTGTTCATGAAGTGTAATAGCTCAGAGA
TGATTGGGTCTTCTTTTGCTAATAAGAAACCAAAACTTCAAGTTCCTTCATCACAATCAACT
CTCAAGGTAACTAACTAAATCCATATGTCTTTGGTTCTATCTCTTGCTGGCATTTTAAATAAC
AAAACAAATGCACAAACATAAAGGAGAAGTAGAAAAAAACAAAAGAGAATGGGCACAGA
ATAAGCTGCATGTGTGTGTGATTGTAACATTTTTGTCTTTCTTATGTGTCTTCTTCTTCTTGAT
CCTATTATTCTTTTTTCTCTTCCTTTCCTATGATTCAACGGTAGTTACGCAATCCTTTTTTGTTA
GTTTTTATTTTAAAATGAGAAATAGATACTAAATCTGTTTTTTCAGTTTGATTCAGTTTTAGCT
CAGCTTTCCTTTTCTAACAAAAAAAATAAAAATTATCGAAGTTAATTAACTTTATTATGTTTTCT
CCTTCTTGCTCTGTTTATTTTGTTCTGTCTAAATTGATATTGTTATGTTTGTAACAAAACATAA
CTTTATTTTGATCAGTTATCAAAATGTGTCATTCTTTTTCCTTTTTTTTTGTTTGGATCTTGGAT
CAAGTTTTGGTTAATGTTTGATTTAATCTAAGTGCATATTCCTTCTACAATATCAAAATTCCTA
TGTTTATGTATAATTTGCACGCTCATACTAACAAAAACTAATCATATATTAAAGACTTGAATGA
TCTTTTTCCTTTTTCCCATTTTAAAATAGATAAAATCACTCATAGATCTAATTACATTCGTTAA
ATTACGAAATGATCACATGTAGAGTGTATTTATATACGATCTCTGTATTTATCAAAGGTGAGA
AAGGAGAAACTTGGAGGCAGAATAGCATCTCTTCACCAGCTAGTATCTCCCTTTGGCAAGG
TATAATTACTGATTAGTTCCCTTTACTAATCACTTGTTTAAGATTATCTAATCTATGAATCAAAA
TTGAATTATATTAGTCACTGTTTGGTTGATTGATCCCTTTTATTTCAGACTGACACAGCCTCTG
TCTTGTCGGAGGCTATTGGATACATTAGATTCCTCCACAGTCAAATTGAGGTGACTTGTCTTT
TCCAAACTACCATGCTTCTTATTTCATTTATTTCAAGACAAATATTTATTTGAATCTTCCTCAC
AACTACAACACAAATAATCATTTGTGAACCCAAGTATGGAATTATTTGGAGTTCTATCTTCCT
TTTTTATTTGGGATTTTTTTTAAATCATAATAAGAACTGAATAACATAAGTAAATTGTATTCTAT
TGAAACAATTAGCTGAGGTTTATGTATTTCTATAGCATTAATTAATTTAACGACTATATTTTGT
GAGATTAGTTTTGTGTGAAAAACTATTTAGTTTTGTTGTCACTCTAATTAATTTTCACAATTAC
AGGCTTTAAGTCTACCATACTTTGGTACTCCCTCGAGGAACAATATGATGCACCAACATGTA
AGTATTAAATTAATATACAGCTTTAATTAGTAACATGTATAATGTAATTATACACTACAAGATG
CACATACATGCATGGGTTTCTACTAACACATCATATATATATAATGGGGCATTAAAATGTATCT
TTTTTTTTTTGGTAAGTGCATTAAAATGTATCTGAATGCTAATCATTATTAATTTGTAATTACGT
GAATATAGGCACAAAGAAATATGAATGGTATATTTCCTGAGGACCCTGGTCAGGTACGCACA
TATATTTTTTTACTTTCTAGTTTTATATACTTTACTTTTTATTGGATCAACTAAACCCAAATCTC
AATTTAAAAAAAATAAATACTTGCCAGCTTTATTCCAAAACAATTACCCTTTTTCTCTACTAT
TGTTCTCTTGAAATTGAAATGACTACGATAAAGTATGGTCAATAAAATAAAAAGATGACATT
ACCAACTTTCCTTTTCAGTCCTCTATTAAACCTAGAAAAGCCATTTAAATGATTTATTATATTT
ATTTTCCTCAATCAGAGTAGCTTTGGTCACAAAAGTTTTTTTGGCAAATGATCGATCTAAATC
AACTTTTCCAACCAAGATATCACGATTCACCTGTCCCACAAATGGTTTTATATTTTGTGACTA
CTTGTGAGTTTGAAATTAACTCTGACTCAAAATTTTAGCAATATTTTTCAAAGCAAAATTTAT
CGCTTTTCAAATTCAGAAAATTAAATACAAAATACTCACACTCAAAGGTATTAAACCCGCGG
ATTGGTGCACAAAACAGTAAATATTCATTTTTTGGTTGCAAAACAGTAAATATTCATGAAAC
TGGCATAGGTTATTTTATCCATATATCATTTACATGTAATACAGGCCGACCCACCGCAAAAGA
ATTTGGGCTGTTGCGAAGTAGGCCAACCCGTTTTGTCACCAATACTCCCACTAGATCACACT
TTCAATATTATTTTAAATATCTATGCTGAAGAAATATTTTCTGATTTGGTTAATCAGCTTGTGA

ATGAGTATTGCATGAAAAGAGGAGTTTCATTGTCATCGACGGACAATCAAAAATCAAATCCT
AACGAAGAACCGATGAAAGATTTGAGAAGTAGAGGGCTTTGTCTTGTTCCCATCTCATGCA
CACTACAAGTTGGCAGCGACAATGGTGCCGACTATTGGGCTCCGGCGTTCGGAACCACTCT
CCAGTGAACTATTCACTTTCTGTTATTTTATTTTTGGTCAGCCAAAAAAAAATAGTAGATGTT
ATAACATCATGAGCAACCAATCAATTGAGAAAACATCAAAATGAGTTTCCAAATTTGATTGC
TCATGATGCTATACTAAAACCGATTGGTTTTGGCGTTTGGCCTTTGAGCATTTTCAGTTGACG
AGTATGAGATGGCTTGTTGAATCATGTTTTTCTTACTTTAATAGGAAACTTTATTTATTCTCTT
AATGTATATGTTGGTTCTATTTTAATTTTATTATTGTTATAATAGAATGGTTAAACAGTTAAAGA
TGTTTTCATACTAACTTCATCACTAGGTTTAATCGTTTAAGATGTTTTAAAGAAGC

>AtbHLH134
CCAAGACTCCAATTCTAAACCACTGTTCTCTCTTGTGCACTTGTCATCGGCATATCCAACAC
CCCTATTTAAGATTATTTGTATTACTCTCGCTCTCTCTCTCTCTCTATCTAGAAGTGGTCCT
AGCTCTCTATATAACACCATTTCTTCTCCTTCTTTCTTCTCCCTTTCTTTCGACAAGCACAAA
CAAAGCCATCAAGAGAAGAAAGCCTTTTCTTGGATTCACATATATATAAGAATATTTTTTCAA
ATCAAACATGTCTTCTAGCAGAAGGTCGAGACAAGCAAGCTCATCATCAAGAATTAGCGAT
GACCAGATCACTGATCTCATCTCAAAGCTCCGACAGTCCATTCCGGAGATTCGCCAGAACC
GTCGTTCCAACACGGTAAATCTTTTCAAAAGTTACTGTGTTTCTTCTATGTACCAAGGTTTTG
GCATAGTCACATAAGTTTTACTTTGATGATATTTATTTAGGGTTAGCTATTATGTTTAATAGAG
AATCACGCATATACAAGTATACAACATGTTAGAATAGTGCATTATAGTCATAGAAGTACGTGT
GGGAGACTGCATTTTGAAGTTTCTAAAATTACTTTTGACCATTAACCGGACTTTGAGGAGAG
GGGAACATATTTTCCCTAACTTCAGCATTAAGCTTCTAGTTAATTTTACTTTTATTATTGATTA
ACTACATTGAATGTGAGACGACTATATTGCTTCACGGACCATCAATTCAATACATTTATGTAC
AGCACCGACTAATTTTTATATTTGGTTTTCTTAGTTTCTATATAACAAGAAAAGTACGAACTC
TAGAAAAATAAGAAAAAGCTGGTTTCTATAAATGGAAGCATCGTTTTCTTATAGTACACACA
ACCAATCAGATTTGTTCTCAATTTCAAAACAATGTGTTGGTTAAGTTATGAGCATTTTTATTT
TTTTGATAAAGTATGGGCATTTTATCTTAAGCTTTAGGTTTAACTTTTAACCTATGCATAGTGT
AGTATGTCAAGCAACTAGCGTCAATATATTTTCTTACATTATTAACACGCATCATAACTTCAA
GCTAAGACTTGTTTCTGTTGTTGTTAAAGCAAGCTAAGACTTGTTTGAATATTTATATTGAAT
CCTTACTATAAAATGTACAATTGTGAAAATTGTTTGTAGGTATCAGCGTCGAAAGTGTTACA
AGAGACTTGCAACTACATAAGAAACTTGAACAAGGAAGCCGATGACCTCAGTGATCGATTG
ACTCAGCTTCTGGAATCCATTGATCCTAATAGCCCACAAGCCGCAGTTATTAGGAGCTTGATT
AATGGATAATTAAGATATAAATTGATTAGTTGTGCTTTATATATATAAGCTTAAAATCTCGTTG
GGAGGTTGATCCATCAGGGTGTTGCATAATTATATATCTATTTTATGTTTCTTATATATTATTTAC
AATCCTATCTAGTTAGGGTTCATATTTTGACCCTTTTTTGGTTTAACGTCATGCATGCAATTCC
ATTAAGCTTAAAAATTATAATAAATAAGATTTCGAGTAATTATTTAATTTAAATCCTTT

>AtbHLH135
ATACTATCAACTTTTCTCTATCTATCTCTCTCTCTTCTTTTTCCGGCATAACTTCTGTGTTACCC
TAAACTCCATAACCTGTTTCACCGATAAAGTGCCTTTGCTTCTATCTCTGTCACTCTTACTAC
TTGTTGAACAATATTCTACAAAAAAATGTCGGGAAGAAGATCACGTTCGAGGCAATCATCA
GGAACTTCAAGGATCTCAGAAGATCAAATCAATGATCTGATTATCAAGTTGCAACAGCTTCT
TCCTGAGCTCAGGGACAGTCGTCGTTCCGACAAGGTATGCTGAATCTAACTAAGTCAACTA
GTTATGTAATATATATGCGTATATATGGCATGAAAATAATGATACATGATACATATTGACAGGTT

TCAGCAGCGAGGGTGTTACAAGATACGTGCAACTACATACGGAATCTGCATAGAGAGGTTG
ATGATCTAAGTGAGAGGCTATCTGAGTTACTAGCAAACTCAGACACTGCACAAGCTGCTTTA
ATCAGAAGCTTACTTACCCAATAATTCCTATCTATCTTTTTCTTCTTCTTCTTTTTTTTGTTTAC
TATAATAATAATAATAGTTTGCGGGTTTTTTTTTCTATAGATGTTGATGACCTTATAAACGTTTA
ATGATACGAGTTCGTCACTTCTGCTTGGGTCATAAATATTCGCTTGTTTCCAATATAAAACCT
TTGCCCATTTTCGGTTATCTTGTTTGTCTCTCCTTTTTTTCTTTCTAATATGCATTTAATTTGGT
TAGGGTTTTGTTCACCAAAGACTTGGCTATGTTCATTTCTAAGCTTTTTATTGGTTTCGTGTT
TTTGAGTAATATAAAACATGTCATAACAAGAGCTTATATGAAGAACAACGAATTTGTAATTTA
AAACATGCCATTCAAAAAT

>AtbHLH136
GGCCTATTTAAATCTCTCTTATCACATTCTCAATTTCTTTATATTTGAAGTGTATTCAAAACCC
CAAAACACTTTTCTCATTCTCTTCTCTATTTTCTTCTTGCTCTCTAGTTTTTCTTTCTTCTTGG
TCGTTTCCTTTCAGCATAAAAACCTTATAAAATCATAAAAGCTTACACCTACTTGCCACATAG
ACATAGCCGATCTCATTATATCTCTATTTCTATTTCTCAATAGAACTTGTTTGAGCTAGTGTGA
GAGAAGTAAAGAAAGAGAGAAGAATCCACAACTTAGTTAGGGTCTTTTCTTGCCACATTGT
TGAACATGTCGAACAGAAGATCAAGGCAATCTTCAAGTGCTCCAAGGATCTCCGATAATCA
AATGATTGACCTCGTATCTAAGCTCCGTCAAATTTTGCCGGAGATTGGTCAACGACGTCGTT
CTGATAAGGTATCACGCATAATATATGCATATATTCATTTATAACGACCATTTAACATTATCCAT
CGTGTAAACCTATCCAAAAAAGGAAAGAACAAAAGTTTTGTAAAAAGAAGAGTGGACACT
AAAATCAAACAAGAAAGAAAAAAGAAGTCGGAACTTTTGACTCCGGACATTAAGGAACAT
ATATACCAATATTCACAACCTTTTTCTTCCATATTTACATAAGAGATTACATCAAAATCATCAG
CTTGCATGCATCTGCAAACGCATAAAAGCGCATGCATTGGTTCAAAAAACATATATTATAATG
ATTATGATTTTATTTCGTTTTTGTTGTTGAAGGCATCAGCCTCGAAAGTATTGCAAGAGACAT
GCAATTACATACGAAATTTGAACAGAGAAGTTGACAATCTGAGCGAGCGTTTGTCTCAGCT
TCTCGAATCTGTCGATGAAGATAGCCCTGAAGCCGCCGTTATTAGAAGCCTACTCATGTAAT
CTTTTTTGTTCTTTTGTTTGTTTTTGACAAGCCTATCCATGTAATCTTAAATGATCGCTCTATA
ATAATTATATTTTTAACATAATCGTCTTATTATGTAAAATTCAAAGAGATGGGCTTGATCTTTAA
TGACATACGAATTTCATAGGGTTAATTCCATAGTCAAAAGTCTCCGACATATATGTGTGTAGC
AGAACTGCACGAGCATTGCCTTAGCAGGTGCATTATTAAAATAAACCAGTGGGATGAAATTT
G

>AtbHLH137
TGGTAGTAATTTCTTCTCAAATAAAAAGGTATCTTCAATATACAAGTGGTCCCTAAAATACTC
TTTCTTCTGTTTCTCCTTGTTTCTTTGAATAGTGAATACAAAAGCAATTCCTTATATAAACCAC
TTCAAGTCCTTAATCTTCTCTCACAAAGAAAAAACACTATCTCTATCGACAAAGATAAGAGA
ACTCAGAAGCTTCTACTCTTAAAATGGCAACTTTCTCTTATTTCCAAAACTACCCTCATTCCC
TTCTTGATCCTCTTCTCTTCCCTACACCTCACTCCTCCATTAATCTCACTAGTTTCATCGATCA
AAACCATCTCTATCCACTACCAAACATTTCCACAGTTGAAGACATTTCCTTTCTTGAATATAA
TGTTGACAAAACTGAAAACTCTGGTTCAGAGAAACTGGCCAACACAACCAAAACAGCAAC
CACCGGTTCTTCTTCCTGCGACCAGCTCAGCCACGGACCATCAGCCATCACCAACACCGGA
AAAACCCGTGGGAGGAAGGCCAGAAACAGTAATAACTCCAAGGTATGTTAAACCAACTTGT
AATGTATCATCTATCATTTATGTGATGGGGTTGGGATAGTCAAAAACATAAATGTTGGTGTGG
TTTGGTGAATTAGGAAGGAGTAGAGGGAAGAAAAAGCAAAAAACAGAAGAGAGGAAGCA

AAGAAGAGCCTCCAACAGATTACATTCACGTTCGAGCTAGAAGAGGCCAAGCTACTGATAG
CCACAGCCTTGCTGAGAGGGTATTTTAGTCATTTCTCTTAACATTTTAATTTTTCTTCATTAAT
CATGTTTTCATAAAATACATTTTGATTAATATTTGATTAAACTTATATATATATATATGTAAAGGT
GAGAAGGGAAAAGATAAGTGAGCGGATGAGGACTCTGCAAAACCTTGTTCCGGGCTGTGA
TAAGGTCAGCTAGAGTAGAGACAATTTTATTTAGGAGAATTGATAATGGTTTAATTTTATAAT
GTTTCTAGAAAATGTTATTAATTGATTAAATGTGACTTGAAAACGAATAGGTAACAGGGAAG
GCCCTCATGTTGGATGAGATTATAAATTATGTCCAGACCTTGCAGACTCAAGTTGAGGTGCA
TTTTCTTAATTATTTTGTTTTTTCTTGTAACATTTTATGTTAAAACTAATGTTTCTAATTCATAA
CTTTTTTTTCTGTTTCATTAGTTTCTCTCAATGAAGCTAACTTCTATAAGTCCGGTGGTCTATG
ACTTTGGCTCCGACCTTGATGGCCTCATACTTCAATCTGAGGTATTTCATTTACACAATGTCT
CTTAATCCGGTTACCGATAAAACCAAGTCATGTATCCCGAAAAGCCGGTTATGGATCTTAAG
TAGATATTTTCTTGATGGACAGATGGGTTCTCCAGAAGTAGGAACGTCTTTTACCAATGCAA
TGCCAACAACTACTCCTATCTTTCCTTCACTATTGGATAATTCTGTAGTTCCCACACATGCAC
AAGTTCAGGTTTTTCATTTTTTTCTATAAGTGATTATTTCAGATAATAATTATAATAAGGAGAA
TGAAGATAGATAAGAATCAATTATTGGAAGATTAATAGATAGTGATGTGTTGCTTAATTTAGG
AAGAGGGAGAAGAAAGAGAGAACTTTGTAGACAGAAGTGGGTTCAACAACAACAACTTC
TGTTCTTTCCCTTGATAAAATTACATGTCGACACAAACACAAAAGCCTGCTTTCGTGAGTTT
CTTCTGGTCTTGTCTCTTCAACTACCTAATTATTATCATTACAATTATTTTTAACATTTCTCATA
TAGAAATTGTTAAAAATCTTGATGTGGGTTCGTAATTATTGCTATCGTTTATTGGATGTAGGTT
GATTTAATCTCCTCCCATTTGCACGGAGGGAATAAAATCTACGTAAACCAGGTTTTGTAAAA
GTGGATGTACTTATTTGTTCCGTTTATATAGTTTGAAGAAATAAAGTATTTTAGAGACTTAAA
AAGCTCATTTTCTCTATTTGTCCCGAGAGTTTCAACATAATCATTACATAAAATCAATGGATA
AATTCATACTAAAAGAGAATGGTATATGAGGCAGAGGCAAATTCGATGGAGGAAAGAAAGA
CATATAAACTAAGAAGACCTATCAGCTTAATCATTGTTTTTTTAATATAATTGAATTTTGATG
TATCCACTCATTAATTTTATAAGTTCTTGTATTTTCACCACTTCTGTCTTTCTGATTTCTTGCAC
CAAAGTTTCCTTTTTCCTCCAATCTTTTTAAATGTAAACTGTTATCCACACACTCATGTTATAT
TTCCTATATATATAAAAATGTTCGAGGGATAACTACTTATGCA

>AtbHLH138
ATGGAACGTTACACAAAAAAAAACGAGAGGTTCAAGGCAGAGGAAGGAAAAGGATCAAA
GAAGTCGAGAACTTTCCTTACAGAACGCGAAAGACGAGCTCTCTTCAATGACCGTTTCTTC
GACTTAAAGAACCTCATTCCAAATCCCACAAAGGTTAGATATATATTTTTATGTTTAATCTTTC
ATTCTTTTTCGTCTGTTTAATCTTTCATTCATTCTCTCCAATATCTCTTGATCAATACACATGCT
CTATTACTAGGGAGGTGAAGCATCCATTGTTCAAGATGGGATCGTTTACATCAACGAGTTAC
AAAGACTAGTTAGCGAGCTTAAGTATTTGGTTGAGAAGAAAAAATGCGGTGCAAGGCACA
ACAACATTGAAGTAGACAATAAAAACACGATTTATGGGACTAGCAAGATAGAGCATCCATTC
TCCAAAAACAAAAACACTTTTAACTGTTTGATTCGTACACTTAGATTTGTACACCACTTTTA
G

>AtbHLH139
AATGAGATTTTAAAACCCTGATTTTATAATCAGCCATCAAATATTTATATTCCTTCTCAAAGTT
ATTCTCCTCATGTCTCTAATATAAGCTTGAACGTTGCTATATATAAATGTAAAGGCGAACGCAT
AAGAAAAGAAAAATGGAGAATGAAGCTTTTGTAGATGGTGAATTGGAGTCTCTTTTGGGGA
TGTTCAACTTTGATCAATGTTCATCTAACGAATCGAGCTTTTGCAATGCTCCAAATGAGACT

GATGTTTTCTCTTCTGATGATTTCTTCCCATTTGGTACAATTCTGCAAAGTAACTATGCGGCC
GTTCTTGATGGTTCCAACCACCAAACGAACCGAAATGTCGACTCAAGACAAGTAAGTTTAA
GTAAAAAATGTTAAGTTTCCTATGTTTACATTTGTCGAATATTAACCCATAACGGTAATGCAA
AGCAGGATCTGTTGAAACCAAGGAAGAAGCAAAAGTTAAGCTCGGAAAGCAATTTGGTTA
CCGAGCCTAAGACTGCTTGGAGAGATGGTCAAAGCCTAAGCAGTTATAATAGTTCAGATGAT
GAAAAGGCTTTAGGTTTAGTGTCTAATACATCAAAAAGCCTAAAACGCAAAGCGAAAGCCA
ACAGAGGGATAGCTTCCGATCCTCAGAGCCTATACGCTAGGGTAATTCAAGATAACCTAAAC
AAATATTTTCATTGTTGATTTGAGTTTTTAGGTTTTGTGGAATAATAATGTTGATGAATGTTGC
AGAAACGAAGAGAAAGGATAAACGATAGGCTAAAGACATTGCAGAGCCTAGTTCCTAATG
GGACAAAGGTCGATATAAGCACAATGCTGGAAGATGCTGTCCATTACGTGAAGTTCCTGCA
GCTTCAAATCAAGGTAACGACTTTTTTTCTTGTTGGAGTATTGATCGTTTCTTGTACTGCAAG
AAACCATGTAAAACTCTTACCTAAACTCTGTTTTACCATTTTTCCTAACCAAACACTTTTGTC
GTTTTTGGAAATTTTGCAATGTAGCTCTTGAGTTCAGAAGATCTATGGATGTATGCACCTCTT
GCTCACAATGGTCTGAATATGGGACTACATCACAATCTTTTGTCTCGGCTTATTTAA

>AtbHLH140
CGTCGTTTTCATGATGCGAAGACGTCACGTATTTCAAATACAGGCGTGCCACACGACACGCA
TTACGAGGGCACTGCAATTGTATAATCGGAGAAACAGAGGAATTCGCCGATCAACGAGAGA
AGATGGAAGTGCAAATTGAAGAATCTGGTAAAAATCAAACCTCTCCGGAATCAATTGAAGC
AGACAAAGCGAAGCAGATAGTGGTGCTTCTGATTGGTCCACCAGGAAGCGGCAAATCGAC
GTTTTGTGACACCGCAATGCGTTCCTCTCACCGCCCTTGGTCTCGTATCTGCCAGGTCTCTC
TTTTTTATCGATTGTTTTATATTGCACATAAGCTGACTGATGAAATTCTTCAACGAGACTAAGT
ATTTACAAAACTTATGTAAGAAATATCTGTCTTGGTTGCTATCAATGTAAGAATTTTGTGTTG
GCTTAGATTTCATTTGTTTAACTTATTCAGGACATTGTTAACAATGGCAAAGCTGGAACAA
AGGCTCAGTGTTTAAAGATGGCTACAGATTCTCTTAGGGAAGGCAAGAGCGTCTTTATAGA
CAGATGCAATCTTGACAGGGAGCAACGTTCAGAGTTTATTAAGCTTGGTGGTCCTGAGTTC
GAAGTTCATGCAGTGGTTCTGGAGCTTCCTGCTCAGGTTTGTATATCTAGATCAGTTAAAAG
AACTGGTCATGAAGGAAATCTACAAGGTGGAAGAGCTGCAGCTGTTGTGAATAAGATGCTT
CAGAGTAAGGAACTTCCCAAAGTGAATGAAGGGTTTTCTCGGATTATGTTTTGTTACAGTGA
CGCTGATGTTGATAATGCTGTTAACATGTATAACAAGCTTGGTCCAATGGATACCCTTCCTTC
TGGCTGTTTTGGCGAAAAAAAATTAGATACTAAAAGCCAACCCGGTATAATGAAGTTCTTTA
AGAAAGTCAGTGCTCTGCCTGCTTCATCTTCTAATGAAGCTACTAACACAACTCGGAAAGC
TGATGAAATGACAGCAAATGTCCGAGTTTCGCCAGTCAAGCTTGGTTCTGCTGATATTGTAC
CTACACTGGCATTCCCATCAATTTCGACTGCGGATTTTCAGTTTGATCTTGAAAAGGCTTCT
GATATCATTGTGGAGAAAGCAGAGGAGTTCTTGTCTAAACTTGGGACTGCGCGTCTCGTCTT
GGTGGACTTGAGCCGCGGGTCAAAGATTCTATCTCTGGTCAAGGCTAAGGCTTCTCAGAAG
AACATTGATTCAGCGAAATTCTTCACATTTGTTGGGGACATAACTAAGCTTCGTTCTGAAGG
TGGTCTACACTGTAATGTCATAGCTAATGCTACTAACTGGTAAGCTTCCATTATTTTCTTGATC
TTCAGTATATACATCTTCACTGGATAGCTAATTTTGAAAGCTAAATGTGTAGGCGGCTTAAAC
CTGGAGGTGGAGGTGTGAATGCAGCAATATTCAAAGCTGCTGGTCCAGATCTTGAGACTGC
GACAAGAGTACGAGCAAACACTCTTCTTCCTGGAAAAGCCGTGGTAGTTCCTCTTCCTTCT
ACTTGTCCATTACACAATGCAGAAGGAATTACACATGTCATACATGTTCTAGGACCAAACAT
GAACCCAAACCGACCAGACAACCTTAACAACGACTACACCAAGGGGTGCAAAACTCTCCG
GGAAGCTTACACATCTCTATTTGAAGGTTTTCTATCAGTAGTACAGGATCAATCGAAGTTGC

CCAAACGAAGCAGTCAAACAGCTGTGTCAGATTCTGGTGAGGATATTAAGGAAGACTCTGA
GAGAAACAAAAAGTACAAAGGATCACAAGATAAGGCCGTAACTAATAATTTGGAATCAGAG
TCTCTGGAGGACACAAGAGGCAGTGGAAAGAAGATGAGTAAAGGATGGAACACATGGGCA
TTGGCTCTCCACAGCATCGCGATGCACCCAGAGAGACACGAGAATGTCGTGCTTGAATATTT
AGACAACATCGTTGTCATAAACGACCAGTACCCAAAGGTAAAAAAGTCTTGATGCTGATTTA
TAAATATAGTATCTTTCTTCATGTGACAAATGACAAATAGGATTTTTCAACTGAGTTTAGGCG
CGGAAACACGTGCTAGTACTGGCGAGACAGGAGAGTCTAGATGGGCTAGAAGATGTTCGC
AAAGAGAATCTTCAACTTCTGCAGGAAATGCACAATGTTGGTCTGAAATGGGTTGATAGAT
TCCAGAACGAGGATGCATCTCTTATCTTCCGCCTTGGATATCACTCGGTACTTAAGTCATCCT
TTACATTCAAAGCACTGCTGAATTTTCTGTTTCCCCTGAGTGTAGACTTCAGATTTGCACTTT
CTGTTCGGTCGTTTTAGAGGTTAAACCTAAGAGTTCATTAGCAAAATCATTGAAATTGATATG
TTTAACATACATTGAGTTTTTCTCTTTCAGGTTCCCTCGATGCGACAACTACACTTACACGTT
ATAAGCCAAGACTTTAATTCAGATAGTTTGAAGAACAAGAAACACTGGAACTCTTTCACGA
CCTCGTTCTTCCGTGACTCAGTTGATGTACTTGAAGAGGTCAACAGCCAAGGCAAGGCCAA
TGTGGCGAGCGAAGATCTGTTGAAAGGTGAGCTGCGTTGCAATCGGTGTAGAAGCGCACA
CCCAAATATTCCAAAACTTAAATCACATGTCAGAAGCTGCCATTCTCAATTTCCAGACCATTT
ACTCCAAAACAATCGCCTTGTGGCTAGAGCAGAGACGTAGTAGTTATAACGACCGGTTCTAT
TTCGGTTAGGATTTTTTTTTCTTTGTTATCTAATTGTTACGGGTAATATATATTGTGATTTTGGT
TCAGATTCAGTCTCTGTAAAAATTCATCTCTGTTCTTAGGTTTTCATCTTCTTCTGGTTATTAA
GTTTTTGATTTTGTTCTTGAGTTATAAACTTAAATGGCA

>AtbHLH141
GTTTTAAATGGGCTTTTGAGCCTTTCACATTATCTATGTAATTTTCGATTTTATCTTCCTTTTCT
TTTCACCTTCTCGACGAACACACCTACACAAAAATCAATCACAGTCTCTGTCTCACTCGCCG
GAGTTTCGGTCACTGCGCCATCTCCGGGGTCGAGTTCGTCGTACTTCATCGAACTATGAACT
CTCATGATATCGATGATCAGTTAGAAGCAGATGTATACAGTAACTTGCCTTCTCGAAATGATT
CTTCAACAGGGAGGAGAAATCGAAACTCGTGTAGATCGAAACACTCAGAGACTGAGCAAC
GAAGAAGAAGCAAAATCAATGAAAGGTAAATAATAAGTGTTCAAAGTTTGAATTTTTATCA
GATTTTCTAAAAAGGGTAACAAGAAAGTTTCGTAATTTGTGATTGTTGGTTGGTGGATACAG
ATTTCAGAGTTTAATGGATATTATACCTCAGAATCAGAATGATCAGAAACGAGATAAAGCTT
CTTTCTTGTTGGAGGTTAGTAAAATTTATAGTTTTGAACAGAACATGTGTTTCAGAGAATCA
GAATGATGTTTGCTTTTATATGTTGTCTCAGGTTATAGAGTATATTCACTTTCTTCAAGAAAAA
GTTCACATGTATGAAGATTCTCACCAAATGTGGTATCAATCCCCAACCAAATTGATTCCTTGG
GTATATTCCTTCTCCTATCTTTGGTTGGTTTTCTGTTATATTGAATCGTTTTTGTTACATTTTTTT
AGAGAAACATGGTTATTGTGTTAAGTGTTTCATCTTTCTTGGTATTTTTGACAACTGTTTCAT
GTAACTTTGATGCAGAGAAATAGTCATGGGTCTGTTGCGGAGGAAAATGACCATCCTCAAA
TCGTTAAAAGCTTTTCAAGTAATGATAAAGTTGCTGCTTCTTCGGGTTTTCTCTTGGATACGT
ACAATTCAGTAAATCCTGATATTGACTCAGCAGTTAGCACCAAGATTCCTGAACACAGTCCT
GTTTCAGCAGTTTCATCGTATCTACGTACCGAGCCTTCGCTGCAGTTTGTTCAGCATGACTTT
TGGCAACCAAAAACAAGTTGTGGAACAATTAACTGTTTTACAAATGAATTGTTAACATCTG
ACGAAAAAACATCAGCCAGCTTGTCCACTGTCTGCTCACAAAGGTAAGACATATGTAGAGC
AAAATAAAGCTTTTTTTGTTCAGTAATTAATTCATTTTTTTGGTAGAAAGTTTGGGTTGGTATA
TAGTTTCTCACGGATGTACACTCATTTCCTTCCTGTCTGGCTCTCCTTAAACAGTGGCTTACC
TTTTTTGGATACTAAGAGAGAATGATATAATACAAGGTTCCAATGATGCCTCAGCTATTTGCG

GAGACTTCTTGTTTGCCTTGGCTATGAAGTTCTGTAGGATAGAGTATGAGATAAATGCTGTA
AACTTGGTAATTGTTAGAGGAAAAGATTTTGCATAACCATAATCCAAAGTTTCTCTTTTGAAT
AGTGCTTCTCTCTCCTAAATAGGTAGTCATTCAAACTGAAGATCCTATCTGCTTCTGTATTTG
GAACAGACTCAGGGTTCTAAAAACGGATAACTGGAGGAAATAAAGATGGAGAGATTTGATT
GAAAACTCTTGTTTGGTTTGATTGTGAACAGGGTACTTAACACTCTAACTGAAGCACTTAAG
TCATCGGGTGTTAACATGTCAGAGACCATGATATCTGTGCAGTTAAGCCTTCGGAAAAGAG
AAGATCGCGAGTATTCTGTTGCTGCGTTTGCTTCTGAGGTATGCCTGTAACATTTTTACAGTG
GAAAAGAAAAAGAAGTCTACTACTCTGCAAGTAATTCTTATAAAAACATTTCTATGTATTTTA
ATTTGTATTTTGATGCTATGTATCTTTTTCATTAGGATAATGGTAATAGCATCGCTGATGAAGA
AGGGGACTCTCCTACTGAAACTAGAAGCTTCTGCAATGATATTGATCATTCCCAGAAGAGAA
TAAGACGATAAGATCTGGTTTTCTATTTCTATGTTATGACAACTGTTTGATAATTTTGTTACT
TTCGGTGAGTCAGGATCAGTAGGTGTAGATACTACTTTTATCGTGCTGCAACTATTTTAAATG
ATATAGTAAAAACTAAGTAGAATGATGTAACAAGTTTCGCAAATCTATAACAAGAGTTTGCT
CCATTTAATGGTACTTTAAGTATATGGTCTCTTAGAT

>AtbHLH142
TCTTTAGAAATATTTCGTAAAAAAAAATTCTCCGTTTAGACATTATTGTTCGTTGGTCACTTT
CATCTCTCTTATCCCTTTGCTTTTCCATTGATTCAGCTCAAAAAAAAACAAAAAAGTCTCAT
CGTTTTCTCTGGATCTGACCGAAAGATCATTCCTTTCTAAGGTAATCAAAATCCAATTTTTCT
CTCTTATAGTCTGTGTCACTGTTCTCTCTGTTTGTGTATCTGGAGACTGTGGTTAAAAACGGT
TTGAGTCGTTTGTGAAATGGTAAAGTTCTGTAATTTATGTCTGAATCTAGCTTAGAACATTGT
GATTTGTGTATATTCTGAGTTGTAATAGTAAAATGTAGCGTAATTTTGCTGGAATTGATGCTGT
GAATACACCCACTTGCTAGACGTTTATAAATCTATGTCCTTTACTGTTTTTGATCTCTGGATTT
AGCTCATTTTTGGTGCTGATGAGATTCTTCCTTCGACTTTTTGCACAGATACTAAAGTAGGTT
TGAGTGATCAGAATCAATTAGAGTGTGTATAAGAGGAAGAGAAAGAGAAAACAGCATGTGT
ATTGCCGTATACCGTAAAGTTTTGAGCTTGAATCTGTATTGCCGTGTGATACTGTAGATTGTT
TCACCTTTGAAGCAACACCTTCAAGAGTCAAATCCTTTTCTTTCTTATTCATCTTCCCTTCTT
TTTCGGTATATTATAAACACTTTTGAAAATGGGATCACAACATTTCTATGTGTACCATTGTGA
ACAAAGAATCAGCTAGACTAAAAGCTCACTCTCCTTCCACAAACAAAACTTTCCTCCTTTT
GGTTTTGGTATCTGTCTACTTTCTTCAATCAAGATTTCCTCAAAGTTATGGTGTGCCAATCAC
CTGGTAAGACAAGATTTCGAGGATTGAAGTACGAGACCGGAAACGCTAACGAATCAACAAT
TGTAGTGAGAGTTATTGAATGCTATCAACCGATGGATAATTGCCAGGTTTGTACCAGACAAT
AATGCTCTTGTTATCATACTTTTAGCTTATATAGTTCTAGTCTTCTAACACTGTGGTTTTGTGA
TTATTCTTTGTGTTTCAGGCTGAGTACTTCAGACTTCTACTCAAACCAGTCACGTAGTTGGTT
GGTGACATTTCGCTGCATTTTTCAATCTGTGATTGTTTTTCGTTCGTCTTTCTTTTACTATTTT
CTCGAAAAGGACACAAGAAGTATTGCATTCACTCAGTTGAGCAACTTAACAATCGGTATGC
TCTTTTCCCTCTAATTACGCTTGTGATATTACTGACTTTATCTTTCTTGAGCTGATTTTTGCCT
CTGTTTTGTCCTGCAGTGTTGTACTTTTTGAAGTTCCCTTGAGCTAAACTGCTAAGAGCATG
CCTCTGGATAAGAGGCAACGGGATTTGCCTCTGGGCTTAAGTCCTCAAGCTTGCTTCAAGG
ATATAGTAGGTCGGTCTGTCCTTCCTAGAATTCCTCTCCCTGAGCTTGGGAAACTATATGCAG
CTAAGCTTCAGGCTCGCTGTTTGCAGCCACCACCATTCCAGTCTTTGCTGTGCAGTCATGAT
AAGGAGTCTTATGGAAAAAGATTCTCACGGTCTGACATGCGGTCTTGGTGCGCTGCTGCTA
CTACTACTACTACTCCACTTGGAGCATTAGAGTCTTCTCAGAAAAGACTTTTGATATTCGATC
AGTCAGGAGACCAGACTCGTCTATTACAATGTCCATTTCCTCTACGGTTTCCATCTCATGCG

GCTGCAGAACCAGTGAAACTCTCTGAGTTACAAGGTATAGAGAAAGCTTTCAAAGAAGAT
GGTGAAGAGTTTCACAAGAGTGATGGAACAGAGTCAGAAATGCATGAAGACACTGAGGAG
ATCAATGCATTGCTATATTCAGATGATGATTATGATGATGATTGCGAGAGTGATGATGAAGTA
ATGAGCACTGGTCACTCTCCTTATCCAAATGAAGGAGTTTGCAACAAAAGGGAATTAGAAG
AAATCGATGGTCCTTGTAAAAGGCAGAAACTACTGGATAAGGTCAACAACATCAGCGACTT
ATCATCACTTGTGGGCACTGAGAGCTCCACACAACTCAATGGATCTTCCTTTCTTAAGGACA
AAAAGCTCCCTGAATCAAAAACCATATCGACCAAAGAGGACACTGGTTCTGGTCTGAGCA
ACGAGCAGTCGAAGAAAGACAAGATCCGCACAGCTCTGAAAATACTCGAGAGCGTAGTCC
CTGGTGCAAAAGGAAACGAAGCGCTCTTACTTCTGGACGAAGCAATTGATTACCTAAAGTT
GCTGAAACGAGACTTAATCTCCACAGAGGTTAAGAACCAAAGCTCCACCACTCACAAGTC
ACCAATCTTGTTGCTTAAAGAGACAACATGGGGAACAAGAAATCTGCAGACAGATAAGGC
GTGAAAGATTCTGACGAGTTAAAACGTGTGAAGTGGGTTTTTGGGTACGTATCCTCTGCAC
CAGCTTTTTAGCTGTATATAGTGGACTACTTTTAAGTATTATATATGCTACTGCAAATTTCAAA
AAGTTAAAGAAGAAAAGAGGTCACTTTCTTCTTCTCTCTGTGTGGAATGAAAAGCCAAAGT
CGTCATGGTCATTGATGGACAATGATTATCTTTTTTGGCTGAGTGTGGATGGTGAAATCAAA
AGGCAAATATTCAAAGTCTCATCTCAAGCTCGCGTGATCTATGAATCTCCCCTAAGGTAATA
AAATATCTCTGACCCACACACACTCTTTTGTCCATATGATGACTAGATGGGTCCATGATTTTT
ATGAAATATTGATGTCATGTTCATGTAATTAATTTGAGTGTCTGTAATAATGCAAACTATTCCT
TTGTCTTTTGCTTCAACCCACATGCTACTGGTATTGTTGTGTCTCTATTGTTGTATTGTTAAAT
GGTTTTGTAATAAACAAATGGCGTTTTTTGGTTTGTGATAAAAATCGAATCGTTTATG

>AtbHLH143
GCCCACTTTTAGTGAATTTGCTAAAAATATATTTTCGAAATAATATTCCGCTTTCTCGTTTCTT
ATCCGTTCAAGCTTTCGTGAATTCGTCTCATAAACCTCTCCACTCTCTCTGGATCTCCACGCA
TAGATCTTCCTCTGTTTTCCTCCATGTACGCTTCTTGGATTCACTTAACCATTGTTTGTCGCTG
TGCTCTCTCTGTTTTTTTATTCCTCGAAAAAGTTGAATTATCAGCGAATTTGATGCAAATGTG
TTTAAATCGTTACCAAAACGGATAAATTCTTCTATTTTCGTAGCTTGGCTTGCAAAATCCAAG
CTTTTAGTTCTTGATTTTGCTAAAATAAATTATACTTGTTCAATGCTGCAGAACATGTACTTAT
CTTCTTCTGCTGTTTATGGTTTCTCTATCCTTTCAATTGTTGTGAATGTTTGGTTTCTTTGGTT
TGTTATTACTGGAATTTGATGAGATGATTCTCTGTTTTGTAACAGAATTCTTCTCTCTTTCTCT
GGATCACAACAAAGCAGTGAATGAGAAAAACAGCATGAGTTGGTTCACAAGGTCTGTTGA
TGTGTACCGTAAAGTTGTGAGCTTGAATCTGTATTGCCGAGTTATTCTGTAAACTTTTTCTCT
GCCTTGAAAAACCCCATCTTCAATCTTCTTCTAAATCCTCAGCCTTTTCTAAACTCTTTAATA
ATGGGATCCCAACGTTTCTACGTTTTCCACTGTGACCAGTATAGGATCATCTAAACCCTTAAA
GCTTCTCTTTTTCTTCTTCAAGTCATCTGTCTTTTGGTGACTTACTCTGTTCTGTGGAGAAAC
ACTAGTTTTACATTCGAGAGTTTGGAAAAGTTATTATTATGGTAAGTCAATCAGCTGGTCAA
ACAAGATTTCGAACATTCAAGTACGAGAACAACGGAGACTCTAGCAGACCAACAATTGTAG
TGAGAGTTATTGCTTGCTTCCAACCGATGGATAATTGTCAGGTTTGTTGCAGCAAAAATGAT
ATTATACTCTTTTTTTTGTCACATTTCATCTCATAGTCCTGTGTTTCTTATCTAACACTTTTGTC
TCTGTTCCACTTGCAGGCTGAATACTTTAGACATATACTCAAACCAGTGACGTAGTTGGTTT
GTGAGCTTGTGTTTGTATTTTTGGAGCTGGCACCTTTTTTCTTTTTGTCTCTACTTTCTTATAC
GATTGTTTCAAGAAAGAATCAACAAGTGTTGCGTTCCACACATTTGAGCAACAGCTTCCAC
AATCGGTATGCTAACTGAATCTTTGACTCTTTATGGATTATTGCTTTCTTGAGTTGGGCTACTA
GTAGTTGTCTCATTCATAGCCATGTCCAATGATTAGTCCCTTTCCTTTTTCGTGCAGTATTGTA

TTCCTGTAAAGTTCCCTTGGCTTAAACTGCAAGAGCATGCCTCTTGATACCAAACAGCAGA
AATGGTTGCCATTAGGCTTAAATCCTCAAGCTTGTGTCCAGGACAAGGCGACTGAGTATTTC
CGTCCTGGAATTCCTTTTCCGGAACTCGGTAAAGTTTATGCAGCTGAGCATCAGTTTCGCTA
TTTGCAGCCACCGTTCCAAGCCTTATTGTCTAGATATGATCAGCAGTCTTGTGGAAAACAAG
TTTCATGTTTGAATGGGCGATCTAGCAACGGTGCTGCTCCAGAGGGGGCACTCAAGTCTTC
TCGGAAAAGATTTATAGTATTCGATCAGTCGGGAGAGCAGACTCGTTTGTTACAATGTGGAT
TTCCTCTGCGGTTTCCTTCTTCTATGGATGCAGAGCGAGGGAACATTCTCGGTGCCCTACAC
CCAGAGAAAGGGTTTAGTAAAGATCATGCCATTCAAGAAAAGATATTGCAACATGAAGATC
ATGAAAATGGCGAAGAAGACTCGGAAATGCACGAAGACACTGAGGAAATCAACGCGTTAC
TGTATTCTGATGATGACGATAATGATGATTGGGAAAGTGATGATGAAGTAATGAGCACTGGT
CACTCTCCATTCACAGTTGAACAACAAGCGTGCAACATAACAACAGAAGAGCTGGATGAA
ACTGAAAGCACTGTTGATGGTCCACTTCTTAAAAGACAGAAACTACTGGACCATTCGTACA
GAGACTCATCACCATCCCTTGTGGGCACCACTAAAGTCAAAGGCTTATCAGATGAAAACCT
TCCTGAATCAAACATTTCAAGCAAACAAGAAACGGGTTCTGGTTTGAGCGACGAGCAGTC
AAGAAAAGACAAGATTCACACCGCTCTGAGAATCCTGGAGAGTGTAGTTCCAGGGGCAAA
GGGAAAAGAAGCTCTTTTACTACTAGACGAAGCCATTGATTACCTCAAGTTGCTGAAGCAA
AGCTTAAACTCATCAAAGGGTTTGAATAACCATTGGTGAAAAACCTACAACCCCTTTTGTCC
TATTGATAAGGCATGTTTGGTTGGTTAAAGAGAAGACATGGGACAAAAGATAATCAATGAG
GTAAAGGACTGATGAAGAAGATTCTCTCAAATTCATTAACGTGGGTTTGAAACAATTAGAA
CACGCCTGGTGACCCTAGTGGGACCGTATCCACTGTTCATCTAGCTGGATCAATAGTGGTTT
ACTTTTGGATTTGGCATGCTCTCTCAAAAAAGAACCAAAAAAAAGTCACTTTCTTCTGTGC
TTGGAGTGGAAGAAAAGTGAAAGAAGGGAAAAAAGCAAAGTCTTGGTGCTCATTGATCGT
TAATAATTATCTTTACTCGTGAGTAATAATAATATCTCTGTGAAGGTCTGGTGGTGGTGGGTG
TGGATGAAGAAAAATCAAAAAGGCAAATATTGAAATTTTTGGAGTGGTTGTGACTTGTGGA
AATTGAAATCTTCCCACTTTCATTAGGTGAAAATCTATGTCCCCACTCTCTCTTGTCTCATTAT
TACTCTTCTGTTGCTATCAATCCCATAACTTGTCATCTTTTTTGTTTTTTCTAATCTCTCTGGCT
CTTAAGCTCTTCAAAGGCCTTGTAGTTTTGTACTATTGTAATGTTATTTGCTTCTGTTGTTTCT
CTCATGGGTCTAAACATGTTTGTGTATCATGTTCATGTAATAAACTCGAGTGTT

>AtbHLH144
AGATCTTCTTCTTCTTCTCCTCTGTAACAACAAAACAAAAAAATCACTCTTTCGAATTTGAT
CAGATTCTTCTAAAGCTTTAGTTTTTACGCATCACCGATTCATTTGATCCAAAGGGTTTTTGA
TTTCTTCTACGGTATGAATGTTTTCTGCTTCTTTCTTTCTTTATACCGCCGCAATTTCTGACTT
ATTAGGACTTATATGTTTTTTTCTTTGTCTTGTAATTAGTACTTTGACGGTATCTCATAAATAGCT
ATTTTATGGTTTATCAGATGATTGCTGAATTTGATTTTTCTATTGAATGTTCTGTCTTGTTTATG
TTTGTTTGTGTTATTATTAATAAACTATTCATGATCGCGTAAAGATTCTTTCTTTTAACAAGTC
CAATTCTTTTGTTGAGGTTTTGTTTCCTCTATGTGTTCTGTTTTGAAGCAGAGTTGTTAAGTT
ATTTTGATTTTGGTTTGTGATCATGAAATCGAAAAACATTTATTCTGGATCCGATGTTAATGTC
TTTAGTCATGATCCTATCTTTTTGTTTGTTGTACGCAGATGCCTAAAGTTGTGCTTTTTTATTA
TCTGAATCAGGAATTAGAAATTTCCCATTTTTTTGTTTCTCTGTTTTGTTTTGCCAGTAGTTAT
TTTAGGATTCTACCTCGAAGTTAGTGCCTTTCAACTTCCTATACTGAAAATATATTAGTATTTG
AATAGCGTTGGTCCTAATTTTTGTTTGTTTGTGCAGAGTTTGTGAGAAGAGGAACGGTATTT
AATACACTTACAAATTTGATATTGGAAGAACCAAAGATGCCCTGGACAGCATTCTTCATGTT
TTTCAACAGAACTTGCACTCGTCTGGTTGTCTTTTTCCTCGTTATTCTGTAATTTTGTTCCTTT

CAGGTGAGCCAGCCTCTCATTTCACATCTTGTGTTTTTGTCTTACATTGGTACCTTGTGGAAG
CAAATTTTGTTGTTGCCACCTTATATACAGCTTCTTGTGTTAAAACTATTGGCATCTCTTCCTT
GTTTTGCAGGTGTTGTTTCCGGAATTCGGAATAGTTCTTTGTTGTTGTGCTTATTATACTGGTT
AGTTTGTGAGAATGTGCATTGTTGGAAACAAGAAGGGAAACCGATCATTGAAGGAAATAG
GAACTTTTATGATGACTACATGTTTCATTGCTAATTACCAATCCGTTCAAGTTTGCCAGGTAC
ATGCCTCTCTTTGATTTAGTATTTCCTAACACTTCTATCGCTTAGGCAACACTGATTTATGTCA
TTAAAAGAACTCTATCCTCTTGATGACATGTGGATAGCTATTTGTTAGAGTATTAACCATAGT
TTTTCTGATATTCAACCTTTCTTATAAATCATTGCAGGCTGAATATTTCAGACAGTTGCTTAAG
CCTGTAACGTAGTCTAGCGTCTGGAGTTTTCGGAAATTGGGGTTTTGAGCGGCTATCTACTT
TTGATTTCGAAAAAATTTACTGTGAGTTGTTAAAGAGTCTGGTATTCTGAAGAGCTGTCTGT
GGGGGAGCTTCTGGTTTGGGGGGTAAGGGCTCTGGTGGAAACTCGTGATATCTTTAGTATGA
TGGCCATAACTTGATATAGCATTTGGTTCCGTGTGTCAACTTAGTGTAGTAGTAACTGAGCA
GCTGGAAGCTTACAAAGGCGACAAATCGGTAGTAGAAAATATTCAAGCAGCTTGCAGTTTG
AGGTGTACAACTTGTTTTTCTCTTTGCGTTTCCTTTTTTACGTTTGCACGGGTTTTCTGATGC
AGAACAATCAGTTTCCTCACTTCTCAGATGAAGTGGGTGACAGAAATATGCACAACCCGTA
TGCATCAGGGTCATCCTATGATGCTTTGTTCCCACCATGTGCAAAGTTGCCATACCATGGTGT
TGAACTTCAACCGTCTGCGGTCTGTCCAAAGAACTTTGTCATCTTCGATCAAACATATGACC
GCAGCCAAGTGATGTACCATCCTGAGCTGACTCATAAGCTCATGAATACCCCTTCGTTGAAC
AATTTAGCTTCGACGTTTCAGAACGAGTATGTTGGGGGAAGTTATGGTAACTATGGTAACTA
TGAGCAAGAAGTATCCTCTTCTTATCAAGAAGATCCAAATGAGATCGATGCTCTCTTGAGCG
CAGATGAAGATTATGAAGAGAATGATGATAATGAAGGTGAAGAGGATGGTGGTGATTCAGA
AGAAGTCAGCACTGCTCGTACTTCTTCCAGGGATTATGGAAACACCACAGCAGAATCTTGT
TGTTCCAGTTATGGTTACAACAACAACAACAACAACAACTCAAGGAAGCAGAGTTTATCGG
GCAGTGCTAGTAGTAGTAACAATGATGGGAAAGGACGTAAAAAGATGAAGAAGATGATGG
GAGTATTGAGGAGAATTGTCCCTGGAGGAGAACAGATGAATACAGCTTGCGTTCTTGATGA
AGCTGTTCAGTATCTCAAGTCACTTAAAATCGAAGCTCAGAAACTTGGCGTTGGACATTTCT
CAAACCAATCTTGAATGCTACCGATATTCGGTTCTTCCATCACTATTGTTTCTCGGGAGGTAA
ATCATCGGTCTAGCGCTATCTGGACCTTTCTCTTTATTTTCCTCCCTCTATACTCACTTCCTTT
TGTTGGCAGGATGGTATCTATTTTATGTTTGAATCTCTCTATGTATTTTGCTTTTTGTGTGTTAC
TGCTTCGATGAAGAAGCAAGGTTTGAACAAGTACTTGTGGATTTGATTTGAGATTTTAATTT
GTATGGTTTTACTTTCACTGTAATCATTTGACTATGAACTACAATTATCATTATGTATTTGCTTC
TACATAGTTTTAAACATTTGGTCTTCCTTCTACTGAACAATGTAAGGGCCATCATTTTGAAAA
GTCACGCTTATACC

>AtbHLH145
ATCACTGAAACTAATATCTCTCTGCTTCCTCCTTAGAACTTCTATTTGCTTCTCTTGCATTCCA
CTTTTGTGAGTTTCTATTATCTTCAGTTTTCGAGATAGAACTTATAAACTCTGTAATAATATGC
ATTGTGCATATTTTTCTAGGTTAAGTTATTTGTTTTAAAGTGTTCTGTTTCTTGCTAGCACTAA
ATCTATTGCTCTCGTTTATTTTGCAGATTCATTTGAAGTAGTGAGATTGACTCCAGAGATAAA
ACAAGAAAGAGCAACAAAGCAACAGCATGCGGTGGTGGTTGTGTCTTTCTGCTTATGTTTT
TCGTACAGTAGTAGTCTTCTGTCGTGTGATTCTGTAAACTGCGATCTTACTTCAGACATAGCT
TTTTTATGTAATGGGGTCGTCTTTTTTCTTCACATTCCCTTACTTTGTCCGCCAAATTATTTAA
GACTTCGTTTGGATACTCTGTTTTGGGAGAAGGAATCAGTTGGGGAGAAAGTAGTTATGGT
TTGTCAATCAGCGGGACAGACAAGATTTCGTACACTCAAACACGAACACGGTATCACAGGA

AACATTGTAGTTAGAGTCATTGCATGCTTTCAACCTCTCCAAGATTGCCAGGTTATTTTCTTG
TTTGATGAGTTTATGTTATTGCAATATATTTAGATTTTTAAACCACCAGAACTTTTTTTTATTGA
TTTCGTTTTAGCTTTGATGTTGTGTATTTTGCAGGCTGAATATTTCCGTCAATTGTTGAAGCC
CGTCACGTAGAGTAGCTCGATACTTATCGTTCTTGTTTCAAGCCTAGTTTCTTCTATCCATTTA
GTTGGTAAGTTCCAGTCTTTGCACACATAATCTTTTGACCTCCCATTCTTGATTATAAGTAGC
TGATTTGTTTGAGATGATGATGTCCTCTGTTTTGGATAGTTGATTTGTTTAGTGTTGGATGGG
ACAAGATCGTGGGTTTGGGTTTCCTACTCAGCGCTTGTGTTCCTTGTCTTCCCTAGCTCTGT
CTCATCTCGGGAAGCAAGACTTGAATTTAGTATCTAAAACCTGCGGCGACACCACCGACAT
GTTTTCTACTCGTGGATCTTATCAAGTTTCTACCCAAGTCTCTCAATCCTATTTTGATGGGTAT
TGTGGATGGGTTCATGGGTCATCCCACCTACAGCAGCAGTTCTTGCCTCCTCAAAACCAATG
TATGAAGCAAGTACCTCTTCAAGTAGACGGTGTTATATCTAAAGCTGAGGAACAATGTAGTC
AGAAGAGGTTCCTAGTGTTTGATCAGTCTGGAGATCAGACGACTTTGCTACTCGCTTCCGAT
ATACGGAAATCTTTTGAGACCCTAAAACAGCATGCCTGTCCTGATATGAAGGAAGAGCTCC
AAAGAAGCAACAAAGATTTGTTTGTTTGTCACGGAATGCAAGGAAATAGCGAACCGGATTT
GAAGGAGGATTCGGAAGAACTAAATGCATTGCTTTACTCTGAAGATGAGAGTGGTTATTGC
TCTGAAGAAGATGAGGTTACAAGCGCAGACCATTCCCCAAGCATTGTAGTATCAGGCCGTG
AGGACCAAAAAACATTTCTTGGAAGCTATGGACAACCATTAAATGCTAAAAAGAGAAAGAT
ACTCGAAACCTCGAATGAGAGTATGCGGGACGCTGAATCGAGCTGTGGTAGTTGTGACAAC
ACAAGGATTAGTTTTCTGAAACGTTCTAAGCTGTCTAGTAACAAGATAGGAGAAGAGAAGA
TATTTGAGACTGTAAGCCTCTTGCGCAGTGTAGTCCCCGGGGAGGAACTGGTGGACCCGAT
CTTAGTCATCGATAGAGCCATTGATTACCTTAAATCATTGAAGATGGAAGCGAAAAATCGAG
AAGCATGAGGTTCTTTGTTTGTAGGATACCTAAGACATATTAGCATTGGCAAGTGGACTTGG
TTTCTAAACAAAGTAGTAGACATATTGGACCGTGGTACTCTTGTCAAGCTACAAAGGGATCA
GGTTGTTGCTAGTTGGAAGATAAACCAAATAATGTTTCGGAGTTGCAAGAGAAGGAATTGA
TATTAAAATGCAAATTGAATATTTCATATTAGTTGATGATTAAGATGGATTGCGAAATTTTAAT
TTCAGTCGTTTAGCTGTTTGTTATATCATGTAGTTGCTTCTTCTTTTTCTATAAGATTTTGTCG
GTGTGGTTGTTATTGTTCATCTGTGAGTCGTGTG

>AtbHLH146
CAAATAGTCTTGTGTTTTGTACGTGAACGAAAAATAATAACACTCCCAAACATATTACCTCAT
ATATGTATACAAAGAAAATTAAGTTCTACAACTTTACTTTATACATCATCAACGCTTGTTATAT
ACAAACAAACGTAGGACACCGCTCTTCTCTTTCTTTGTCTCTCTCATATTCTAAGTTTGCTAT
ATATGTAACTTTCTCCCTTGTAGACTTTCATTTGCAGCAAAAATAGTTTCTCATATGGAGAGG
CAAATCATAAACAGGAAGAAACGAGTGTTTTCTCTTGAACCAAACAAGAACCCTAGTGCA
GTTTTCACGAGAAAATACACAAGCCACTTGGTTCCTGCACTCAAGAAGCTCAACATGAACA
AGAACTCTTCAAAACAAACCGTGAAGCATGAAGTAGATATGGCTTTGGCTTTGTCTGCTCA
AGAATTTGCATGGAGCCGTTTCTTGCTGCAGAAGCTATCGTCCTCATCGAATCCAACCACTA
CCACTAGTTCTTCTTCCGATGGAATTCGGATTCTTGAAAGACCCGATAAAGAAGGCGGAAA
CGAAGAAGGAGGGATAGAGGAGAGACTGAGGGAATTGAAGAAGCTTTTGCCAGGTGGGG
AAGAGATGAATGTGGAAGAAATGTTGAGTGAGATTGGTAACTACATTAAATGTCTTGAGTT
GCAGACGATTGCTCTCAAGTCCATTGTTCAAGATAGTACTTGACTTTTAGTTTAAGTGTATAA
TTTATAGTTTGTTTCATGTTTTTTTTGTTGTCTTTAAACGAAAATCATCTGTGTTTTTTTTTTTC
TCTTGATTTCTTTAAGAACAAGAGCATGTATTATAGTGTTTTTCTTTTTTCCTTTTCTTAATTAA
TCATGGTTACCGAAATACCTAAAGTACCACACATGGCCGTTTATACGTACATCTTATAAAATT

CGATCAAAATCTGATTGAAATTTAAATTATTGGTTGTAATGCCAAATAGACGAT

>AtbHLH147
AAGGATAACAATAATTAATTCTAACAGGATAGTGCACATCACATGGCAGAAGTAGGACAGTA
ATTTGTTTTTGGTTACATGTGTATAAAAATGCGATTCTTTGCTTCATTCCTCTTTGTTAAAATA
AAATAAAATAAAAATAAAAGGAAGAAGAAGAAGAAAGGATTCGTCTTCCTTCTTCCA
CACCCTTTCTGACACCTCATCTCCCCTCCCACCTTCTCCGTCCCGTTCCGTTTCTCCATCAGG
TAATTATTAATCTCCTCTTAATCATCCCTCTAATCTCTTTCCCGTTTCTTCATCTCCCGATTCTC
AATTCCATCTACACAGATCCTCGATTCCGATCCAACAGTTCGAATTCAGTTTCCGATTCTGAT
CTGATCGAACCAATACTCACTGAGATTTGATCGGCGATTATGGAGTCTATATCTCCGGTATCG
AATCAGCTTCTTCAGCCGACGACGACGAGCTCTAATTCCGATCGCTCTCGTCGTAAGCGTAA
GAAGAAATCATCGCCATCATCGGTTGAGAAATCTCCATCTCCGTCAATATCGTTGGAGAAAT
GGAGATCGGAGAAGCAGCAACAGATCTACTCAACGAAGCTAGTTCATGCTTTAAGAGAGCT
ACGAATCAGCCAACAACCATCTTCATCATCATCTTCTTCGATTCCACGTGGAGGTAGAGCCG
TACGTGAAGTCGCCGATAGAGCTTTAGCAGTTGCGGCGAGAGGTAAAACGTTATGGAGTCG
AGCGATACTTTCAAAAGCTGTAAAACTCAAATTCAGGAAACACAAACGCCAGAGAATCTCT
AATCCGACGACGACGACGTTAACCACCGGGAGTATCAGGTCAAAGAAACAGAGAGCGACG
GTTTTGAGGCTTAAGGCTAAAGGTTTGCCAGCTGTACAGAGGAAAGTGAAAGTACTGAGCC
GGTTAGTTCCCGGTTGCCGTAAACAATCATTACCGGTGGTTTTAGAAGAAACCACTGATTAT
ATAGCTGCGATGGAGATGCAGATTCGTACTATGACTGCGATTCTCTCCGCCGTTAGTTCTTCT
CCTCCGCCGCCAACGCCAGGTCACGAAGGGGGACAAACACACATGCTTGGTTAGTTGGCC
AAACTGTCCTTTTATTTTTTTCCAATTTTAAAAACCAAAAAAAAAACTGTTTTTTTTTATCAT
ATTATTATTCTTCTCACTGTATTGTTTGTTGTTTGAATATTTTACTATTCAACTTTATTTACCATT
TCGTCATGTATATCCTTTTTTCTTGAATCTCTCTATTACCAAAAAAATTAGTGCCAAGAAGAA
GAAACAGGACAGTGATTAATTTATGTATAATCGTAATAAATTTCAGTTTCTTGATAATCGTGG
AAGCACATTTGAGAGATTGTGATTTAGTTTGTTGTTTTTGCAATACTTGTTGATTAAGATAAC
AACTGTGGACATAATGACAAATTTGTTTTTAAAGTGTTTGGTGAATTAATGATATGGCAAAAT
GAAACAATGGAGAGTATAATTAGTTTTTGTTTTGTTATGTTTGAAGTTAGGTTGGTGATTGGA
ACTGAGTTCTCTAGATCTGTTATGACAACATTTATTTTC

>AtbHLH148
TTCGTATCTGGCTAAAACAACAAATACTAAAAAACAAATTGACAAAACGACGCCGCTTATT
AACTGCTACATCTAAAACGGGGCCGTTTGTTAACTGCTACGTCTCGTGATAGTTGATACCTC
TCTCCCTTTATATATCTTCCTCTTCTTCTCCCTTCCATTATTCTCCACAATTTCATAACTTTCTT
CCGCTCAACTTCAGATAAATTCGGATTCTGTAGCTCTTTCAATACGACTGCGGAGATCAGAG
CCAATTATTTGGTTATGGCGTCTCTGATCTCAGATATTGAACCGCCGACGAGTACTACTTCAG
ATCTCGTTCGGAGAAAGAAGAGATCCTCTGCTTCATCCGCCGCATCGTCTCGTTCAAGCGCA
TCTTCCGTCTCCGGTGAGATTCACGCGCGATGGCGATCGGAGAAGCAACAACGGATCTACT
CAGCCAAACTGTTCCAAGCGCTCCAACAAGTCCGCCTCAACTCTTCCGCCTCAACATCATC
ATCTCCAACGGCTCAGAAACGAGGAAAGGCCGTCCGTGAAGCCGCCGATCGAGCTCTTGC
CGTTTCCGCTCGGGGAAGAACACTCTGGAGCAGAGCGATCTTAGCTAATCGGATCAAACTG
AAATTTCGTAAACAGAGACGTCCTCGAGCTACGATGGCGATTCCGGCCATGACTACGGTGG
TTAGTAGCAGCAGCAACAGATCGAGAAAACGGAGAGTGTCGGTGTTGAGATTGAATAAGA
AGAGTATACCGGATGTTAACCGGAAAGTACGTGTTCTAGGCCGGTTAGTTCCCGGTTGCGGT

AAACAATCCGTACCGGTGATTCTAGAAGAAGCAACTGATTATATTCAGGCTCTGGAGATGCA
AGTGAGAGCCATGAACTCTTTAGTTCAGCTTCTCTCCTCCTACGGCTCAGCTCCTCCACCGA
TTTGATGAGGTTAAAATCGTCTTTTTAATTCTACCATCTCTCGATCTTTCACAGCTTATGTGTA
TATAGAAGATTCGGTTTGATTATAATCTGTAACTACTCTTCCCAACCGCTGATTCTTCTCTGCT
ACAAGTAAAAGTAAATTTTGAACCGAGTCTTCCCATTTTTACGATCCTCAAGTCTAAATTAA
GTATATGATTGATTAATAAAGTCTTTACCATTAGGGTTCAAATCGTAGTTGGATTATTTGATTT
AGATTGATGAATTCGTTGTTGATCAAAAAAAAAATAAGATTGATGAATTCGTTATTGGAGTT
AGTGGGAAAATGGGTTCCTCTTTTAACCTCATAGTATGTGATAACTAAACCAATATGGAACT
TTTGATGTACTTTTAGTTCATTCAATATGGCTTTGTACTATATCATCTTATGTACTATATGAAAT
TTCAACATGAAATAGATTTGTACTGCAATAAAGTGGAAAGAAAATAATTTCCCATTTAGAAT
TTTAAAATACATTATAGTAGATGTAGGAAGATAATGGGTTAACTTTAGTTTTGATTATGGTAGC
ATGTGATGATGCATTAGAGAATGAATGGAATGATACATTCAAATTCATTGAATTGAATGATGA
CTGATGAGATTGATCTGTCACTCAATCATCTACTCGCTTTATTGCCCCTCCT

>AtbHLH149
ATCGCTCAGCATTTTGACTATTAAAGGGCATAATTTTGGAGCTTCATGCCAAGGCAAAAAGA
TAAAGAACAATCACATGAATGACAAAAGATTAAAAACAAAAGATTTTTAACTAATGAAAAA
AAACACAAAAAACAAATACCGGTAAATAAGGGCAAAAGTGGAATTAAGTGTTATAGCTCCG
CTGGCGATTGGCAGAGATATAAAAACTGATCTCTCTCCATTCATCTTCTCTTGTTTCTAACTA
GTCACATTGAGAGAGAGAGAGAGAGAGAAAGAGAGACTCTCAGAATCTGAAGAAGAAGAAG
AGATTGTTGTTTTTGCCTTTTATCATCGGTTTCTTTGAATCTCTGGTTTTAAATCGGATTTAAT
GGTGGAGTCTCTGTTCCCGAGCATCGAAAACACAGGTGAATCGTCTCGAAGAAAGAAGCC
GAGGATATCAGAGACGGCGGAGGCGGAGATAGAGGCACGACGTGTCAACGAAGAAAGCTT
GAAGAGATGGAAAACGAATCGTGTGCAACAGATCTACGCTTGTAAGCTCGTCGAAGCTTTA
CGCCGAGTTCGTCAGAGATCTTCCACCACCAGCAACAACGAGACCGATAAACTCGTCTCCG
GCGCGGCGAGGGAGATACGTGATACGGCGGATCGAGTTCTAGCTGCGTCCGCTCGTGGTAC
GACTCGGTGGAGCAGAGCGATTTTAGCGAGTCGCGTCCGAGCGAAGCTGAAGAAACATAG
AAAGGCGAAAAAGTCAACGGGAAATTGTAAATCGAGAAAAGGTCTCACGGAGACGAATCG
GATTAAGTTACCGGCGGTTGAGAGAAAACTGAAGATTCTTGGCCGTTTGGTTCCTGGTTGC
CGGAAAGTCTCTGTACCGAATCTTTTAGATGAAGCGACCGATTACATCGCAGCGTTAGAGAT
GCAGGTTCGAGCCATGGAGGCTCTCGCCGAACTTTTAACCGCAGCCGCACCACGGACGAC
GTTGACCGGAACTTAACGGCGGCAGTTAGTTTGTCAGTTGTTAATTAGCTTTTCTTTTACCTT
TTTACCCCTTTATTTTGGCTTCAAGTGTTTTTTTTTTCTCGTCGACGCGATTTTAATTTATTAA
ATTCATGTATTTTATTTGTATACATCATCACTTCTTTTTTTTTGTATCCATTTTGATCTTTCTTTG
TTTTTTCGTTAGATAATCAAAGTTATCTAAACAATAACAACAACAAAATGCCAACTAATGTC
GATTCCAAAAATTGTTTAACGCTTTAAAAAAAAATTAGACCTACAAATTAAAGAAA

>AtbHLH150
CACCAGTTCAATCATAATTGGCCAATGTGATTTTAGACGTACATTATTATTAAAGAAAAATAA
ATAAAAAATAATGGTATTCAATTCGAAAAACCCAAGAAAATATAGAAAATAATTGATTTAAG
GGTGGCTTCTCCAATCTCTCCCAAATGTTTATATATAGAGAATTAGATATCCCCTCCAATTACT
CAAAATTATTATCAAAATAAGAAAGAGCTCAACATTGTTCTCTGACTCTATAAATAACTGTTC
TTGGAAATTTTCCAAGAGAACAAGAAACAAAAACAAAACGTTGTTTAAGTGTGGTTCTATA
GATTTCTTGTGAATGTCTTCTGAACAAGGAAATGGATCGAATCCTTCCACGTCACCGGAGGT

AGAAGGGACTAAAACAATCCCCTTTCGCCGGAGATTGCAAAGAGGACAAAGAGTTTTCGC
TCCAAAGCTGATGGAGGCTCTACGTCGATCAAGGGTAAGCTCTGAAGAAGCTCCGGTAAGG
CATCTTAGTCGTAGATGGAGAGCCACAACGGCGCAGAAGGTATATTCCCTAAAGCTCTACG
ACGCTCTTCAACGATCGCGGCGGAGCGCAACAGTCCGGGACACGGCCGACAAAGTCCTTG
CGACGACCGCTCGTGGTGCAACTCGGTGGAGCCGAGCCATTTTGGTTAGTCGATTCGGAAC
GAGTCTGAGGCGGCGTAGGAATACCAAACCGGCGTCGGCATTAGCTGCGGCGATCAGGGG
AAGTGGTGGAAGTGGGAGGAGGAGGAAGTTATCGGCGGTGGGAAATCGGGTCCGGGTTTT
GGGTGGGTTGGTGCCGGGTTGCCGGAGAACGGCGTTACCGGAGCTTTTGGACGAGACAGC
AGATTACATAGCGGCTTTGGAAATGCAAGTCCGAGCCATGACAGCTCTATCAAAGATTCTGT
CGGAGCTTCAGCCGTCTACTAATCTCGGCTCGGCTTTATAGCCGAGTGTATAAGCCGATGAA
TTTGTTTCAATTAATTCTTTTACGATTTTATCGTGGGAATGTCTTATTGTTTTTAATGATTTGTT
TGCCCTGGAAAAAAAAAATTGTCAAAATCATCACGAGTTGGACGAAAATACTATGTTTTTCT
AAAAACCTTATAATAGGATTCGAAATATTTTGGTGTCGTTTCACAAGTTTGAATTGTAAATAT
TTTCTAAGAAAATGAAATTTCTAAAAAAAAATTTGAATATAAT

>AtbHLH151
TCCTCTGATTTAAGCTAAACCCTAATTATCCATCAAAAACAACATTTAAAACACCCTTGTGG
ACACTTGTCAAATTCTCATCCAATTCTTTTGAGAGATAAAAGGAGAACCTTCTCAGAAGCTG
TAATCTCCATTTTAAGCTATATATAAAGGTCACTGACCTCTATGTATGTCTATATAAACTAAGG
GCGTCTCATACTCTCATATATATTTGCATCTAATCTTGTAAGCAAACGTTATCACTTGTCTACA
CAACATTCTTTCATTTACAATAATAATATGGGTGTAACATTAGAAGGACAAAGAAAGGAATC
AATTTGGGTTTTGATGAGAAGACAAAGGGCTCGAAGGGCACTTGTGAAGAAGATCATGATC
CGACCAAGGAAGAGTGTAGAAGCTTCTAGAAGACCTTGTCGCGCAATACACAGACGAGTG
AAGACGCTAAAAGAGCTTGTTCCCAACACCAAAACATCAGAAGGTTTAGATGGACTCTTTA
GACAAACGGCAGATTATATCTTGGCTTTGGAAATGAAAGTGAAAGTTATGCAGACAATGGT
TCAGGTTTTGACCGAAACTAACTGTGTTTAAAAGCCTTCATATATTTTTTGTATATCTTGTTGG
ATTTTACGTTCTTTTTAGTTTTTATTTGTTCGTGTTTATTTTTTATTATCTCGTGTGATTGTCTTG
TGTTGCTTATATAGAAAAGGAATTTGGTTTATCTTGCTGCTGTAGACTATGCAGAAAATTAAA
TATCAAAAATATATATGTATTATATGCTTATCTAAATAACAGATGACTGTTGGTTCGGCAAAAA
AAACATGATAATTGTTCCATGGGAATAGTTTTTGTGTGCTACATTAGCAAAGAACAAAGGGT
CATTTCTTAAAAAGGTTGTAAAG

>AtbHLH153
0CTAGACGAAAAAGACAGACATAAAAATAAGTCCCCTTCTATTTGTTTGGAGCATAAACAAA
AACAGACACAGTTTCTTCTCTCTCTCTCTCTGTCTCTTTCCTTTTCTCACGCCGAGATTTCTG
CGACTGCTCTAATCTTACGACCTTATAAGAAACGACGCTTATTTTCACTCTGCTTGAGCTCTT
CATCGTGTCACACAGCACGATCTTGGACGTCGTTTTAAGCGCAAAACAAAGGAAAAAGAC
TGACGTTTATACATACTAAATACTGGTTTGGAGTTTTGCCTTTTGGACTCAGAAAACTCAAA
AGAGAGAGAGAGACATTTCTGTATCTTATCGGGTTTTGTGTTGTCAGAAAGAAGCTCAAGG
ACAAAAAAAAAAAGCAATTATTTTTAGGGTTCAAACGAAGCAAAATTTGGAACTTTCAGAAG
TTGTGGGTGGTGGCTTCTTGAACAATAAAGCTTTTTCTTAGACTCTTCTTCCAATTTGTGACT
CTACCTATCTCTCTCTCCGTAAGCTCTTTCTCTCTCTCTTTATTCGTTCTTTCTTTATTTGCTCT
GTTTCTTTACTCCATTTTTGGCTTAATTACACCAAATTTAGTCTGCTAAAAGCTAATTTCAAG
CATAATTACTTATCTCTCCTCTTCTTGTTGCTAACCTTCAGTTGGATTAGGTTTTGATTTCGTT

TATACGACAATCCCCGTGACTGAAAAAGTTACCAACTTTGATAGATATCGTTACCAGATTCTA
CTCATAGTTTTGTTATTGTAATGCAAGAAGTTGAGTTCTTGTTTACATTAACTTCAATAATTCA
CTGAGAGTGACTTTTTGTTCTCCTCTGATCCGTGGAGGATTCTCTATATGTGTTTTGTTTTTT
GGGGGGGTTTTCTCCTGTTTTTAGGGACTCTATTGATTCATGCATATATGATGTTATTCTCTGT
TTATGAGTTTATGCAAATAATCTAAGCAAGGTATGATAACATATTTAATCTTTTTGTTTGAGCT
TATAATTGTGCTTAGAAATCTTAAAATAATGCTTAAGCTGATGTGAACTTGAGGCCTAGCCTC
TGACCACAAATCCTAGCCTACTTCGTTGACCATACCTTTGTCTTGGCTTAATATTCTAAATTT
CTCTTCCAAATCCTGTCTCCCAATGCATTGGTTGGTCCAAAACAAGATCATCATTTCATAGAT
CTTGTAATCTTTCTGGTCTGTAATTTGCTCTAGGATGGTAACCTTGTACCCTATGTAACATGTC
TTGTTAGTATAAATTTTGGGTCATGTTGTGTTGATGACTCTTTAGCGCATTTCTTATTGACAAA
AGGTATGGAATTCTCTAGAGACGCTGGAATGATGATGGAGAATAAGCGGAATGTCTGCTCTC
TCGGAGAAAGCAGTATCAAACGCCACAAGTCTGATCTCTCTTTCAGTTCCAAGGTTTTGCTT
ATCCTAGCTAGTTTTCATGGATTTCTCGGTCGAAATAGTCTTCGGTACAACAAATTGCATGGT
AATGATCCGTCCATCTTTGTTCTCTTAATGGCAAACAGGAGAGGAAGGACAAGGTTGGAGA
ACGTATTTCAGCTCTTCAACAAATAGTTTCCCCTTATGGAAAGGTAACTTTGTTTTTTTTGGC
AAGCAAATCAAAAGAATCTGTCTCTCAGCTCATACTTTTCTCTAATGTTCACAGACCGACAC
TGCATCAGTTCTTCTAGACGCGATGCATTACATAGAGTTTCTTCACGAACAAGTGAAGGTCT
GCAGTTCAATACCAAGCATGATTCATTCATCTTTATCTGAGTTCCCATGTTCTTTTGTACAGG
TGCTAAGTGCTCCGTATCTGCAAACGGTACCTGATGCTACGCAGGTGAGTATGATCTTGAAT
CTCAACTTCATTTATACTTAGTTCACTAGTTCTACTCTGCATCTGACAGATCTTCACTTGGAA
CTTGAATACCTTGCACGGACTGATTTTCCCGTCTTTTGTAACCAGGAGGAGCTGGAGCAGTA
CAGCCTGAGAAACAGAGGATTATGTCTTGTTCCAATGGAGAATACAGTTGGAGTTGCTCAA
AGCAACGGCGCTGATATATGGGCGCCCGTGAAGACTCCTCTATCACCAGCTTTCAGTGTCAC
ATCTCAATCACCCTTTAGATGACCAATTCGACTAATCACCTACTACGATCTTTGTGTTAAGCC
TAAAAAAGAATGACCAATTGTTATTTTTCTGATGATGCCTCTGTAACATATATAGACAGAGAG
CACATGATGTTGGTTTAGAACAGCTCATGGTTGGCAATGATTGTTATTATTATTCGACTGCTT
AATGCATCCCCTTACTTTGGACTTGTGTAATATGCCCAAACAGGAACCAAATGATTTTAATGC
AAAGTAATCTTTATCCCAAAAAATGCCTCAGATA

>AtbHLH154
CTCTTTTCTTTGCTTTCTTCTCTACATAAAACTAAAACTCCTCTCTTTCACGACCCTTTAAGT
AACGACGCCGTTTTCATCCAAACACAAACCTCTGGAATCGTGTCACCGTTGGAGCTCCGGC
TTCATTAATGGCTTTCTTCAAGACCTGAAGACAAAAGAAAGTAAAAATATATATACACTAGA
GAGACCAAGATCCTCGTTTATGGGTTTCATCGTGTCTTGTGCTTAGGGTGACATAAATACCCA
TTTAGAGAGCTTAGACCAAGAAGTTTTAGAGTTTGTGAAGTTACTTGTTGGTTAGTCTGCTG
TTTCTGGCAAATGCTGAGTCTCTTCTCTCTGTAAGTTCTTTTTTCTTCATTTACTCTGTTTTGT
TCCCCATTTTTGGTCTTTTTGACTTAACTTAGAGTATTTTAAGGCAAAATAAAATCATGAATC
TTTTGGTATTTTTTGTTCTCATCTTCTTGTGGGGTTTTTCTATTGAAGTTTGCTTAGCTTTGAA
TCTTGTCTAATGGAGCTAACTCGATCCAAAATAAAAACACGTATAATGATTAACTAGTTCTGC
TCATTAGATGAACTAAATTAATGAGTTAATTCCGTAGTTTCATGCATCCTACAATGTATTGTCT
ATGTATGGAAGCAAAGTATGATATCATATTTAAACATCTATGATTGTGCTACTAAACTTTAAAT
AATACTTTAAGCTGATGTGATCTTATATGGATAACCTCTGACCATTATCTACTTTCTTTGACCA
TTGCTCTTGTTTTCTAATCCAAAAGTCGTATCTTCCAATGCATTAGTTGGTCCAAACCTAGAT
TTGCATCCCTTAATTTGTAGTAGTTAACATCTAATTTTGATGTTAAAACCAAGACACCACTTT

GTTTGTCTGGTCTGAGAATATCTCAAGGCGTATAGTTTTGTCCTTATGACGCTTGCAAAAGC
AAGTTGTTGAGTTGTAATCTTATGTTTTTTTCTGCAACTTGCCTTGGATGAAGCCATGAGATT
ATCTTTATAAATGGTAGCAAGTAAACCGGTTTCTTATGTCGAAAAACCGATCATTTTTGTTTT
GTTTGATGACAAGGAATGGAATACTCTAGAGACTCGGCAGAGATGATGATGGAGACTAAGC
GCAATGTCTACTCGCTTGAAGATAACAAAATTAAACGACACAAGTCATCTGATCTCTCTTTC
TCTTCCAAGGTCTTGTGATACATCAAAATCAAAATTCATTGTGGCTATGAAGCTACCGGACA
TTTCTGTACCAGCATGCTAATGATCTTGAAACTTTGTTGTTCTGTGGTTTAAACAGGAGAGG
AAAGACAAATTAGCAGAACGTATTTCAGCTCTTCAACAACTAGTTTCCCCTTACGGAAAGG
TACTACTTGGTCCGAAATAATCTTTCTTACACGCTCTTTGTCTTCTCTAGCCATGTTCAAGTC
TCTATCTATGCAGACCGACACTGCATCAGTTCTTCTAGAGGGAATGCAATACATTCAGTTTCT
TCAAGAACAAGTCAAGGTCTGCTCAAGTTTTCTAATCAAATACTTTTCATTCATTCACCTCCT
CCAATCCAATGATGATTATGCTTGATTTTCTCTGTTCAGGTTCTAAGTGCTCCTTATCTGCAA
GCAACCCCTTCTACTACAGAGGTGAGCTGAATCTATGATCTGGATCTCGTTTACTTGGATCA
CAATGCAAGACTGATATTTTCCATCTCTGTAACTAGGAGGAAGTGGAAGAGTACAGCTTAA
GAAGTAAAGGGCTATGTCTTGTTCCATTGGAGTATACATCAGAAGTTGCTCAAACCAATGGT
GCTGATATTTGGGCTCCTGTGAAGACTCCAACATCTTCTCATGCTTTTAATCTCTCGTCTTCT
AATTCGCCCTTTCAATGAGCTGCTGCTAAATTCTCTGTCTCAAGACTAATGAAAATTAGCCA
AAGCTTTGGTCTGATCATGTATTGATGGAGAGACCTTGAAGTCGGTGATGGCTGTTTCTGTT
ATTTTTTACTGCTTAATCCCAACTCTGTGGTGGCTTCTTGTAACAATTGATTCTAATGCGAAG
TTTAACGGACCGGTCAATGACCGGAATTAGCTTCCTGTTTAATAAAGTTTGAATTGGTGAAA
AA

>AtbHLH155
TTCCTTTTTCCGGCGAATTTCTGGGCTGAGATTGTTAGTGTCTCTGAGTGATGCAGAGATCA
ACTGTTGTAGTTAATCAATATCTTCTGACATAAGCTAAACTCGATTCTTTCTTACAATCTCTCT
GTCTCTCTCTGCTTCCTTTTCTACTGTCTTCTTTCTTCTTCTCTAGCTTTTGAATTTTCAATATT
GATTTCTCCCATGGCGAGTAGCAGTTGCAGCAGATCTATCTCTGTTTCAAATTCTGATTCTCT
CGGTGTTTTTTGTTTGAGCTAGGGCTAAATTAGTTTCCTTCTTGGGTTTTTATTCGTAATCCGA
TTCAAAGTTTGTATCTTTGGGAGGGATGGGTAAGAGGCAAATATCACAAGATGAAGTAGGT
CCGCCCATAAAACCAAGAGCTGGTTTACGAAGGGAACAAGCTGGAAGAGGTTCTTACAGA
GGTAGTTAGTTACTTTGTATCTCATCGAAAACGAATCAAAAGAAGCTTCTTTTTTTTTTTATT
AGAGGTTGGGGAGGAGATAGTATAAAACAACTGTTTAGAGTTCTGATTTGTGGTTTTTGAGT
CCGGGGTTTTGAGGTTTACCCTTTAGAATGGGTTCTACTTCTCAAGAGATACTGAAGAGCTT
TTGCTTTAACACGGACTGGGACTATGCTGTGTTCTGGCAACTTAATCATCGAGGATCTCGAA
TGTGAGTCATCTAAACAATTCTCTTTACTGTCTTTGCGTGGTGGATCATTATAATTGTCTACG
AGTTAGAACTGTGGTAATTGATTTAGGTGTATTGACTTCAACTTGCTAGAGTGGAAATATGG
AACCTTATATCGAAATGCAGCAGCTTTAAGTTAACAATTGTAGTTTAAGTGTCTGAAGATTGT
TTCTGTACTTTTAAGTTTTGTGTGCAGTTCAGGATTCTATTTCTTTATATGTTTTAGTGTTTTA
GTTGACCAGATAGTTTCTCAATTTTTTGAATAATGAACACCTGTAGGGTACTTACCTTGGAG
GATGCTTACTATGACCATCATGGGACGAATATGCATGGAGCACATGACCCCCTTGGGTTAGC
TGTGGCAAAGATGTCTTATCATGTCTATTCTCTAGGGGAAGGGTAAGAATATATTGCTCTGAT
TCACTCTTATCATGCCTATAGTTTGATGTGCCCTTTTTCCTTCTTTTTTTATTTGACAGTAGCC
ACATAATGCAAAGCTTTTGTTGTGTCCTTGAGCTTGATGTTAGCTTTTACAATAGTTAACAAT
CTTGGGTCTATTGTTGAATTATGTTATCATATCTATATAAACAGGATTGTAGGACAAGTTGCAG

TTTCTGGAGAACACCAATGGGTATTTCCCGAAAATTATAACAACTGTAACTCAGCATTTGAG
GTTAGGGACTTTCTATCTGAAATTAAATTTGTTTTCTTCGTGTTTTTAAACCAAATGTCTTTTT
TTAATTACAGTTTCATAACGTTTGGGAAAGTCAAATTTCTGCTGGAATTAAGGTATTTGGATT
TTTCTTTAGTGCTCTTTCTGTCTGCTTTATTATACATACTTGTCAAGAACATCCTCAAAGTTTG
CCACTGGAGACTTTCCTTTTTAAAATATCTATTCTTCCTTCTCCGCAGACCATTCTTGTAGTA
GCTGTTGGTCCATGTGGAGTTGTTCAGCTAGGCTCTTTGTGTAAAGTAAGTAAGATCTTAAA
ATTGGATGCGGTGTTCGCAGTTACAAAATAATAGCCAGGAAGAACACCTGCACACTTATAAT
AGATGGCACATATATACATATCTGGTCTTATTTTGTATTTGTTTGGAGCAGGTTAATGAAGATG
TGAATTTTGTGAATCATATCCGACATTTATTTCTGGCACTTAGGGATCCATTGGCAGACCATG
CAGCAAATTTAAGGCAATGTAATATGAACAATTCGTTATGTCTGGTAAAGATCTTCTTCATTC
AAATATGGATTCATGTTTTTTCTAGATTTCTTGTTATTTGCAAAATGGGGAGAGTAAAGTGCT
TAGGGTTCTTCTAACAGTTACTCAAACCTCCACTGACTTCCTGAGTAGACCATGATGTGTTG
TATAATTTTGTTGCAGCCAAAAATGCCTTCTGAAGGTTTACATGCTGAGGCTTTCCCTGATTG
CTCTGGAGAAGTTGACAAAGCTATGGACGTGGAAGAGTCCAATATTCTAACTCAATACAAA
ACTAGAAGAAGTGATAGCATGCCTTACAATACTCCTTCGTCATGTCTTGTCATGGAGAAGGC
AGCCCAAGTTGTTGGTGGTCGTGAAGTTGTGCAAGGATCTACTTGTGGGAGTTATAGTGGT
GTTACGTTTGGCTTTCCAGTTGACTTGGTTGGTGCCAAACATGAGAATCAAGTAGGTACTAA
TATAATCAGGGATGCACCTCATGTGGGAATGACTAGTGGCTGCAAAGATTCAAGGGATTTAG
ATCCTAATTTACATCTGTATATGAAGAATCATGTGCTCAATGATACAAGCACATCTGCTTTAG
CAATTGAGGCTGAAAGATTGATAACAAGCCAATCATATCCACGCCTGGACTCAACTTTTCAG
GCTACCTCGAGAACAGATAAAGAAAGTTCTTACCATAATGAAGTGTTCCAACTATCTGAGA
ACCAAGGAAATAAATACATAAAAGAGACTGAGCGTATGCTGGGGAGGAACTGCGAGTCTA
GTCAATTTGATGCTTTGATCTCATCTGGGTATACTTTTGCTGGCAGCGAGCTACTAGAAGCGT
TAGGCTCTGCTTTCAAGCAAACGAACACTGGTCAGGAGGAGCTACTGAAGTCTGAACATG
GTTCAACAATGAGACCAACAGACGATATGAGTCATAGCCAGCTCACGTTTGACCCTGGCCC
TGAGAATCTTCTAGATGCTGTGGTTGCTAATGTGTGTCAAAGAGATGGTAATGCGAGGGATG
ATATGATGTCGAGCAGATCGGTACAATCATTGCTTACTAACATGGAACTGGCAGAACCCTCA
GGTCAGAAGAAGCATAATATTGTTAACCCAATTAATAGTGCTATGAATCAGCCACCAATGGC
AGAGGTGGATACCCAACAAAATTCATCAGATATTTGTGGAGCGTTTTCTTCAATTGGATTCT
CATCTACGTACCCCAGCTCCTCTAGTGATCAGTTTCAGACATCCCTGGACATACCCAAGAAG
AACAAAAAGAGGGCCAAACCTGGTGAAAGTTCTCGACCTCGCCCAAGAGACAGGCAACTT
ATTCAAGATCGAATCAAGGAACTTAGAGAACTTGTGCCTAATGGATCTAAGGTGATTTTTCT
CTTCCTAGCTACAAGATTACTTGCACCTTCCTTTGTAATGAATTTAACTTATAATATATTTTTGT
CATTCACTAATATTTTTGTCACATTTTCAGTGCAGTATCGATTCTTTGCTAGAACGAACGATC
AAACACATGCTCTTTTTGCAGAATGTTACTAAGCATGCTGAAAAGCTCAGTAAAAGTGCTAA
TGAAAAGGTAAAAATGTCAGCTTTCTTGATAACTGATAATCTCTGTTCTATCCTAGAAATTTT
TATTCCAGCCTCCATAGTTTGTAAATGAACACATTAAGCAGTTTGTTAGGTTTGATTGATTGA
TGTAGCAAATAGTGGATCAAATTTCTGAATCCTTGAATGAGAAGATAGGATTATTAGGCACA
TGGAGATATATAGAAAAACTCAACTGAAAATCAGAAGTAAATATCAAAATTAGCTTATACCT
TTGTAATCTCAGATGCAACAAAAGGAAACTGGTATGCAAGGTTCAAGCTGCGCAGTGGAGG
TTGGAGGTCATCTTCAAGTAAGCTCGATCATCGTGGAGAATCTGAACAAGCAGGGGATGGT
GCTTATCGAGGTATGTAATCTTACATTTTGTCTCTTTTCTTTGGTTTAGTTTAATCTTTGTTTGA
ACTCGAGTCCCAAGTTCTGCGAGTGTGTATTAAAAGTATTTTTGGGTATTGGACAGATGTTAT
GTGAAGAATGTGGCCATTTTCTTGAGATAGCAAACGTCATAAGGAGCTTAGACCTCGTCATC

CTAAGAGGTTTCACCGAGACTCAGGGCGAGAAAACATGGATATGCTTTGTAACTGAGGTAG
GAAGTCGAATTACCCAATTCATGAAAGAGATTCCAAAGCAAATCAAAGTATTTTCCTTGGTT
TTGATTGAACTTTGGATTAGTATGATATATATGACTGACTAACATCGAGATACTGATATATGAA
TGGCAGAGCCAAAACAGCAAAGTAATGCAGAGGATGGACATTTTGTGGTCTCTGGTGCAAA
TATTTCAACCAAAGGCCAATGAGAAGGGGTAACAGGCGTGGTTGCAGAGTTTGCGTTTACG
TAAGATAAAGAATAGGCTTGTGTGGGCTTGTACAGTGTTGTTGTGACACTCACTGCAATACT
TTCATTTGTTTGTTTTGATTTTTTGTATAAAAGATAGGACTTCAAAGTTGAGTGTTTAGTGTG
TTTATGATTTGCATTTGTTTGCTTTTGAGTTTTTGGACCCTTTGAAGTTTGTATATATACATGA
CTTGAAACTTTGATTTTCTT

>AtbHLH156
ACCAAGAAGCAGAGAAAAGAAACATTAAAGCAGAGAAAAAACAAAGACAAATAATCTCC
ATTTGCTTTCTTCTCCATCTTCTTCTAATTTTCTTCAAGTTTTATCAATGGCTGGTGTTACTGG
AATCTTAAAGCTTGAAGCTTGGAGCATCTTGTGAAGAAGATTAACTAGTTTCTTGTATTTTTC
TTTTCTGGGGTTTTGAGTTTTTTTTCTCTTCCTTATTACCGGAAGTAATGGCATGTCGGAGT
ATGATCGCTTTTAGTTTAGATCCTGAAAGAAAACAAAGTGGAGGACTTAGGACAAAACAAG
CTGGAAGAGGATCTTGCCGTGGAAGTTAGTCTTTGTTCAAATCCATTTTCCTCTTAAAGAAT
GTAATAATGGGTTTTTATTGATTGTTGTAGATTTGTTAAAAAGTTTTGTTTTTAGACCTAAAGT
TTCGATTTTTTTCTTCTGGGCAGTTTGAAATTGCACTGTTGCAAGTGAAAAAGTTTGTTCTT
GTTGGAGCTTAAAGAAGAGAGAAGAGAAAGAGAAAAGCTATGGGAGTTTTACTAAGAGAA
GCTTTAAGGTCTATGTGTGTTAATAATCAATGGTCTTATGCTGTTTTCTGGAAAATTGGCTGT
CAAAATTCTAGGTACCACTCTTATTCTCCTAATTCTAATAAAGTGTCAAACTTTATTCAGTCA
AATATATTCAGTGTTTGTTAGTTTAAAGTTTGAGAATTTTTTGATAAGGGTTTAAAACTCAGA
TCTCGAGGATGACTAGAATTATTTATCTCTGTTTAATTCTTGGAGTTTTGTGTTTGGTTTGATC
TTGTTTGTCTTAAAGAATCTTTCGATTTGTAGTTGTTTTGTCGACAATAACTATATCCTTATTA
TTTGTTTATGAATTGTCTAAAGAGTTCAAGTTCTTTAACTCTCTTGTCATTTCAATTTTTATTT
CTTGTCTTTGTTCGTTTTGTCTTTGTTTAGCCCAAAATCTTTTGTAATCGTCTTTTTGTGGTAT
TCTAACTTAAACGATTGTTGCATATTTTTGTCATGTGTATTCTAATTTTTTTTTTTTGATGTGTT
TTTTTTACTTGTTTTTGTGCAGCTTGTTGATTTGGGAGGAATGTTACAATGAAACCGAATCG
AGCTCGAATCCTAGAAGACTTTGTGGACTTGGTGTTGATACACAAGGGAATGAGAAAGTTC
AGTTGCTTACAAACCGAATGATGTTGAACAATCGAATCATTTTGGTAGGTGAAGGTCTAGTT
GGCCGAGCTGCATTTACTGGACATCATCAATGGATTCTAGCTAACAGTTTTAACCGCGATGT
TCATCCACCCGAGGTTATTAATGAGATGCTTCTCCAATTCTCTGCTGGTATTCAGGTTTGATA
CTTTCTTCATGTTTTGAACATCCTAAGTTAGGCTTCTTTAGTGTATATATATGTTCAAGCTCAT
AAGAACTGTTTGTGTGTGCTTATTTTGTTTGTTCTTCAGACTGTTGCAGTTTTTCCGGTTGTT
CCTCACGGTGTTGTTCAGCTCGGTTCCTCTCTGCCTGTAAGTTCAAGTTACATTGTTATGTGA
AGTGAGATTCTTGTTCTTGATTTAACTGTGAATTGTTTTTTGTGTGTTCAGATTATGGAGAAC
TTGGGGTTTGTGAATGACGTGAAGGGTCTTATCCTGCAGCTCGGATGTGTTCCGGGGGCTCT
CTTATCTGAAAACTACAGAACATATGAACCTGCTGCTGATTTTATTGGAGTGCCAGTGTCTC
GGATAATACCTTCTCAAGGACATAAGATCCTCCAATCTTCGGCTTTCGTAGCTGAAACTAGC
AAACAACATTTCAACTCCACTGGATCATCAGATCATCAAATGGTTGAAGAATCTCCCTGTAA
TCTTGTGGATGAACATGAAGGAGGATGGCAAAGCACAACAGGATTTCTAACAGCTGGAGA
AGTTGCAGTACCTTCAAATCCAGATGCGTGGCTGAATCAAAACTTCTCTTGTATGTCTAATG
TGGATGCAGCAGAGCAGCAACAAATCCCATGTGAAGACATCAGTTCGAAACGCTCGCTTGG

AAGCGACGACTTGTTTGATATGTTGGGTTTGGATGATAAGAACAAAGGTTGTGACAACAGT
TGGGGAGTTTCACAGATGAGAACAGAAGTACTCACTAGAGAGTTATCCGACTTTCGGATCA
TTCAAGAAATGGACCCTGAGTTCGGTTCTTCGGGGTATGAACTTTCAGGAACAGATCATCT
GTTAGATGCCGTGGTCTCGGGTGCTTGCTCCTCCACAAAGCAGATCTCAGACGAAACTTCC
GAGTCCTGCAAAACCACCTTGACTAAAGTTAGTAATTCTTCGGTTACCACGCCGTCTCACAG
TAGCCCTCAAGGTAGCCAGTTATTTGAGAAGAAACATGGGCAACCATTGGGGCCATCATCG
GTCTACGGGTCTCAGATAAGTTCTTGGGTTGAACAAGCGCACAGCTTGAAGCGTGAGGGCA
GTCCAAGGATGGTGAACAAGAATGAAACTGCAAAACCGGCTAATAACCGGAAAAGGCTTA
AACCAGGAGAGAACCCAAGACCAAGGCCTAAAGATCGCCAAATGATCCAAGATCGTGTCA
AAGAGCTGCGTGAAATAATACCAAACGGTGCAAAAGTAAGTGAAAAACTTATCTGTTCTTG
GCAAATTAAACAATCCCTAAGAAAGGTTATTACTAACAAAGAGTACATGTGGTTTTTATTTA
ACAGTGTAGCATTGATGCACTCCTTGAACGTACAATCAAGCACATGCTCTTCTTGCAAAATG
TCTCCAAGCATTCTGATAAGCTGAAGCAAACCGGGGAATCCAAGGTATAACATAAAACAAT
AATATTTTGGTGTCTCTCATGGATTCTTGATCTAACGTTTGTTGTATTGTTATAAGATTATGAA
AGAGGACGGTGGAGGAGCAACATGGGCTTTCGAAGTAGGGTCAAAGTCTATGGTGTGTCC
AATTGTCGTAGAAGATATAAACCCGCCTCGCATTTTCCAAGTCGAGGTAACATTTCTGCCTA
AGGTTCTTAATAAAAAACACTCAAGATCTCGGTTTTGATCGAACGTTTTCTGGTTTTGTAAC
AGATGTTATGCGAGCAACGAGGATTCTTTCTAGAAATCGCAGATTGGATCAGAAGCTTAGG
GTTAACGATCTTGAAGGGAGTCATCGAAACTCGAGTAGACAAGATTTGGGCTCGCTTCACT
GTTGAGGTATATTATATAAGTTACACAAGGTTGTTTAACTTGGAAAGATGAGATTGTGTTAGA
ATGTATCTTATTTAAACTGAAACCGGGTCGATATTGGATTGTAGGCGAGTCGCGACGTGACG
AGGATGGAAATATTCATGCAACTAGTGAATATTTTGGAGCAGACAATGAAATGTGGAGGAA
ACTCTAAGACTATTTTGGATGGTATCAAAGCTACAATGCCTTTACCGGTTACTGGTGGCTGTT
CAATGTAATGACACAAATTATTGACCGTACCGGATTGCTCCGGTTTAATAAACCGGGCTGGT
TAAAAGCTTGATTCATATAGAGGTTACGAGCGGACCGCTCTCAAGGATTTTAGGCTCGCTAT
GACTAACTAAAAAGTCAGTCTAAACCAGAGATGATGATTTTTAAGTGTGTTTTTTTTTCTCA
ACTTTATGTTTTGTACATTAGTTGCGAATTTTCATTTGTGTGGAATTTTTTAATTTTAATTCAG
TTTTGGAGTTGTAAAAAATTTAGTTAAATTGTTT

>AtbHLH157
AACAAAAAGCAAAACTCAAAGGAATTTTCTCTGTATTTTTTTCTATCTGCAGAAAATTAAAG
AAAAAAAGAGAAAACTCAAAAACCCACATTTTTCCTCTGTTTCCAGCTTTTCTTTATCTTTG
GAGTTTGGTTTCTGGAGAAGACAAAAGTTGTTGTCCTTTTTAAGAGTTCGAGATCCTGCAG
AAATTAAAAGGGCTCTTCCCGGCTTGGATTCCTAAATATGGGTTCAGAGTATAAGCATATATT
GAAGAGTCTCTGTTTAAGCCATGGATGGTCTTATGCTGTGTTTTGGCGTTATGATCCCATCAA
TTCCATGTAATTTCTCTCTTTTTTTCTTTCGAATGTTATATGCATGTTCCTGTCTTGGATAATATT
TGCATAAAACTAGTTTTGTTTTCCGTTGATTTAGGCAAAAGACTGAATTGCATATCCTCTGGT
TTTATATGTGGCTTCCCTACTTTGGTGTCCATAAATGTAATTTAGTTTTGTTGAATTCCATCAG
GATATTGAGATTTGAAGAAGCATACAATGATGAACAATCAGTCGCACTAGTTGATGATATGG
TTCTCCAGGCTCCTATCTTAGGCCAAGGGTATGCTTTTACACTTCACTCAAATCTTTTATATAT
CTGTCTCTGTGTTTGTGTTTATATATCTTCAATATACTTTTCTTTTGATGAAATTAGAATTGTGG
GAGAAGTAGCTTCATCCGGAAACCATCAATGGCTGTTCTCAGACACATTGTTTCAGGTTTGT
TCACTTCTTTTTCTTGGTGTTTGATTTTTCTCATCTGTGAGCTATAATGTATGTCGAAATATAG
TGACTGCATAGAACATTTCATGATGAAGAAGAAAAAAGATTTGATATTCGTGCGTTTGATTG

TTTTGCAGTGGGAACATGAGTTTCAGAATCAGTTTCTCTGTGGCTTTAAGGTAATTTATATTT
TGTCTTCTCTCCAAGTTTAAGGATTCATATATCCATTTTGAATCAGATTCTTATACGGCAGTTT
ACTTATACACAGACTATCGCAATCATTCCACTAGGATCCAGCGGCGTTGTTCAGCTAGGATCT
ACTCAAAAGGTTTGCATATATAATTATATAACAATGTTTTCTTGTTGTGGAAGTCATGGTCTAA
TTACTTACTATCAATCTTTAGATTCTTGAGAGCACAGAGATATTGGAACAAACAACAAGAGC
TCTCCAGGAGACGTGTTTAAAGCCACATGATTCCGGTGATCTCGATACTCTGTTTGAGTCAC
TGGGAGATTGTGAGATTTTCCCAGCTGAATCTTTTCAAGGATTTAGCTTTGATGACATCTTCG
CTGAGGACAATCCTCCTTCTTTGCTCAGCCCTGAGATGATCTCCTCTGAAGCAGCTTCTTCC
AACCAAGATCTGACCAATGGAGATGATTATGGGTTCGACATTCTCCAGTCTTACTCTCTGGA
CGATCTATACCAGCTGCTCGCTGATCCACCAGAACAGAACTGCTCTTCCATGGTGATCCAAG
GAGTTGACAAGGATCTTTTCGACATTTTGGGGATGAATTCTCAAACGCCTACAATGGCTTTG
CCACCTAAAGGACTCTTCTCAGAGCTCATAAGCAGCAGTCTTAGCAACAACACTTGTAGTA
GTTCTCTCACGAACGTGCAAGAGTACTCTGGTGTGAACCAGAGCAAAAGACGGAAGCTTG
ATACATCATCGGCGCATAGCTCAAGCTTGTTCCCTCAGGAGGAGACAGTAACAAGTCGGAG
TTTGTGGATTGATGATGATGAGAGATCAAGTATTGGAGGGAACTGGAAGAAGCCTCACGAA
GAAGGTGTGAAGAAGAAAAGAGCAAAGGCAGGAGAAAGCAGGAGGCCGAGGCCTAAGG
ACCGTCAGATGATACAAGACCGTATCAAGGAGTTGCGAGGGATGATTCCAAATGGAGCTAA
GGTAAATTGAATAATAATATAGTTTAGTTTAGGGCATCCAAATTATAGATTAACAAATGCATTC
TCTAATAAGTTTCGATATATATTTAAACAGTGTAGCATAGATACGTTGCTGGATCTGACCATAA
AACACATGGTGTTCATGCAAAGCCTCGCCAAGTATGCAGAGAGACTCAAACAACCATACGA
GTCTAAGGTAATGAATCAAATATATCCATTCAGTAAGTACACAAGTGTTTGAGTATTGATGAG
TGAATGATGTTTATCAGCTGGTGAAGGAGAAAGAGAGAACATGGGCGTTGGAAGTGGGGG
AAGAAGGAGTGGTATGTCCGATAATGGTGGAGGAATTGAACAGGGAAGGAGAAATGCAGA
TAGAGATGGTGTGTGAGGAAAGAGAGGAGTTCTTGGAGATAGGACAAGTGGTGAGAGGAT
TGGGATTGAAGATATTGAAAGGAGTGATGGAAACAAGAAAAGGACAAATATGGGCACACT
TCATCGTACAGGCAAAGCCACAAGTCACAAGGATTCAAGTCTTGTACTCACTTGTTCAGCT
CTTTCAACACCACACCAAACATGATGATTTATTATCATAGTATAATACCTACCACTTTGATTTT
CTTTCTTTTTGTTACAACAACAACACAAGAATCTGTTGATAAATAGTAGTAACATTACATCAA
AGA

>AtbHLH158
TGAACCTATAGTTTAAATTTAAACTTTATAGAGAATTTGAATAGTTGTGTGTCTTGTGACATA
AAAAAACATAAAGAAAAGTTAAGTACAAGGAAAAATAAACATTATTTATTCCTCTCTATCTC
TCTCTCTCTCTCCACCTTTCCATTTATATTAACCCTAATATTGTGTCATTCTCAAAAAAAAAAA
AGTTTTATAAAGGTTCTCAACTCAAGTTCTCTTCTCTAAAAAACCCAAACAATGGCCTCTGC
AGACAAACTCATAAACACAGATGTCCCTGAAAAGGACGTTTTTGCCTTCCACTTCCTCCAA
TCCCTCTCAAATCTCAGAAAACAAAACCCTTTTGATACTCCGGACCAAAAAAACTACCGCG
TGAGGAAGATCAAGAAGGCTGCGTACGTTTCCATGGCCAGAGCAGCCGGAGGGAGTAGCC
GGCTATGGAGCAGAGCCCTCTTGCGTAGAGCAGACAAAGATGACAACAAGATCGTAAGATT
TTCAAGGAGGAAGTGGAAGATATCATCAAAACGGAGGAGGAGTAACCAGAGAGCTCCGGT
GGTGGAGGAGGCGGCGGAGAGGCTGAGGAATCTTGTTCCGGGAGGCGGAGGAATGGAGA
CGTCAAAGCTGATGGAAGAGACGGCTCATTACATCAAGTGCCTTAGTATGCAGGTCAAGGT
CATGCAGTGTCTCGTTGATGGCTTATCTCCCAAATGATCATCAATTAATCATCAACATCATCAT
CAATGATCGATGTATATAGAGATACGACACATACATAGTCTATTGGATTGGGGTAATCATAAC

GAATATATATGTGTATATATATATATATATATATATATATATATTCTTATATATATACATACGTGTAAGAT
AGGATACATATATGAAATGTGTGGATATGTATTAGTCTCGAAGTAACGAAAGATTTTTTTCTT
TTTCTTTTCTTGAATTTTGATAAAAGGGGATTTATTATTTATCATATGCTATGGCCGGTTTAAA
CATTAGGGCTTGGTAACTTGCCCTTGTCATATGATAAAAGGGGATTTATTACTTATCATATGCT
AAGAGAATTTTCTTTCTTCTTTTCTTTTAAGCTATGCAATTTACTTGGTTCCTCTTGATTGTGT
TATTCGTCAATGATGTTTCTATGGATGGATTATATATATAAAGTTATAACATATTAGTTATAAGA
AAATTATAGCGGAA

>AtbHLH159
GCATGTGCTTCTCCTATCTCCTATTGCCCCCACCATTTTCCCTCTCCTTTTGAAACAATTAAAA
TGGACCCTTTCCTTCTCCTTTCTCTCTCCCTTACCTCTCTGTCTCTTTCCCTCTCTCTCGTCGA
ATTCACGAATTCGTGAGGTGACCGCAGATTTGTTCTTAGCAATCTTTGACGACACTAGAAGT
AAACAAATTCTTAAAAAGGTATGTGTTCATGTCTCTTCTGGTTATAATAAGGAACCCTAAAC
GGGGAAAAAATCCAGTTTGGTTATGATTTGATGTATATTAGTATATTACATACATGTATCCCTT
TGATGTATAAAGACATCATGGTTTATTACTTAGGATTAACTTACTTTTTCTTGAAAAATGACTC
ATAATTGAGTATATTTCTACTTGATTAGGATTTAGGATGCAGCCTACGAGCTCAATGAATGAA
GAATTCCTAAAGAAATGGCAAATGGGTCTTCAAATATTCCGTCCTTCGATAGACAACACGAG
CGTCCACGAGAGAAAAAAAGCAATAAAGCTCTCTGCAGACGTTGCAATGGCGTCTCTAAG
AAAAGGAACAACTTGTTGGAGCCGAGCCCTAATCGAGAAAACCGCCACCGAGGACAATTT
CCTCGTACGTCAGATGCTTTCCGGCATCAAAGCGGAAACGTTAATCAACAAGAAGTTGCCT
AAGAAGACTGTGTGTCATAGGAAGATCGTGAGGCGAAGCAAGAAGATCTTGAGGAGGAAA
TCGAAATCAGCGAGCGAAGAGGCTGCAGCAAAAGCTAAGAGACTCGTCAAGAGACGGAC
TCAAGGATTAAGAAACGTTGTACCTGGTGGTGAGTTGATGAGCAATGACGTCTTGTTGCTTC
AAGAAACGTTAGATTACATTGTGTCGCTTCAAACGCAAGTAAATGTTATGAGAAGTATTGTT
GATGCTGCCGAGGCCGAAATTGAGAGATAAATGATAAAAAAAAATCTTGTTTTATTTTTTTAT
CTGCGGAGATTAGTTGCGAAAGCATGATGTCGATCGGATCTTGTTGTTGGTCATTAGAACCG
TATGTTATTCGGACAATGCAATTGTCATACGAGGATGGAGAGATATTATAGTAAGCCATTTTG
TACATAAGGAAATTTATACAATGCTTATAGAAAGTTTCTGCTACGTGATGGTGGTTTTTATTG
GAACATTTCTTGGCAAATTTTTATTTTTTTTGAATTTAATTTAGTTAATGTGAGTGAATTTTAA
GATATTTTTAACGCTTCGATTGCTTTTCTTGGCATGTTTT

>AtbHLH160
CTTAAAGAAGAGAGAGAGGAGAGATTACGGGTTACATATAATCAGTTAATCACAAATATTAA
AGAAATGTGTGTACTGTGTACTACAGTTTAGCGCCATTTGTACTGCATAAAGACCCCCCACG
TACATACATACACCAAACCAGAGAGACTTACATTAGAAGAAAAAACTATGGCAACCTCTGC
ATTCTTACAAAGAAGTAGAACATCGGAGGAGATGAATCAGGTAAGAAAGCCTCTGTAAATC
CTGTCTCACAAGTTTTCCTCTTCTCTAAAACAAATAAAACTGTTTTTGCTCTTCTCTGGCTCG
CTCGCTCTAGGTCTTATCATTTACAAATTCTTATGTACTGCAGATATACGACCATTTCTCTTCT
AATAACTTGCTGTGACCTGTGATTCCCTGAACCTCTTTATTTTTTAATCTTCAAGCATTTTAAT
ATTCAATATTAATGTTTAATTCTGCAGCTTCATTGCATTGTGCCATTCTTAATTTGTTATATATT
ACTGCGTCAGGGGTTGACTTTAGGCTAAGGCTACCTTTTCCTATATATATCATTGTCATGATCA
TCTTGTTAACTTAATTAGGGTTTTAGACATTAATTGCAGTTCTAAGTTTAACTTAGGACCATC
GGTGGTAGGAAAGAGCACAAGCCTCTTTTTTGTTCCTCTTGCCGTCGTATATATATGTCTTCT
CAACCGAATCATCAGACTTCAATATCAAGTTTACTCCATGATCGCTTGCATATCCCTCCTGCA

GAAACAATCGTTGAGAAAGAGTCTGCAGAGAAGGACACTTGTCAATCACAAAGAAAAAG
GAAAGAACCAGTTTTGCATGAGGTTGATGGCTCAAGTAGTGGTGCAGCAAAGAAACAAGA
CCATAACGCTAAGGAGCGTTTAAGGAGGATGAGACTTCATGCCTCTTACCTCACTCTTGGCA
CTCTTCTTCCTGACCATTCTTCTTCTTCTTCAAAGGTTTTGTTTTCACTGTTATTATTACAAGT
TAGGTATGTTCTGCTTGTTGTTGAGCTATATATAACTTTTCTTGCTGATTGGCAGAAGAAATG
GTCTGCACCATCTATAATCGATAATGTAATCACTTACATTCCCAAGCTACAGAATGAAGTTGG
AGAGCTAACTCTAAGAAAGCAGAAGCTTGTGGAGTTAGAGAGACGAGGTCCTTCTATTCGA
GCAATCTCGGTTCTTGAATTGGGGGAATCAGGTTATGAGGCTGTTGTACAGATATGCTTGAA
GAAAGAAAACGAAGATGAGTTCTCCAACTTGCTTCATGTAATGGAGGTGCAAGGTTTGAGT
GTTTTAAGTGCTTCTACTTCTCAAGTTTGCCGAGAGCAAAGGGTGGTTTGCTACAACTTCCA
TGTCAAGGTACTGTGTCTCATCTTATCTTCTTCATTTCTCTTTAGTTTATGTTTTGATAGAATG
GAGACAATGTGCTAGTCTTCATGGATATGAACAATGATGTAACTTTTCTTCTCCAAACAATAC
ATGTTTAGGGTCCAAGTCATGGGAGGATTGTGTTTTAACTTTAGCTAAGCATTGAGATTATGT
TAAGATTATATAACGTTTAGAAAAGATATAGGACTAATGGTTTATCATTAAGTTCATAAACCAA
TGGTTTTTGTTCTTAAGATATATTGATTCTATTGGTGGGAGAGAGACTGTTTCGACCTCAATG
AACCTGGATTTGAGTGTAGCATTATGTATAAAGAGGGATGATTTACATATATTTGCATGTATTA
TCAATGGCTGATCCTAGATCTATGGACCAAAAACACTAAACTAAATTCAAAATTAGTTTTTA
GCAATTCGATAGTATGGCACTAAAAAGTCATTGGATAGTATTTACATATATACATTGGATGTTC
TTTTTTGAGAGAACTAACAATTGCAAAATGTATATGGGAAAGTGTAGATGGATGAGAAGCCA
TGTGAAGGTGATGACTACATCACAGTGTTAAAGAACAACATCATTTCTTCGCTTCGTGACAA
CACAAAGTGCAAATGA

>AtbHLH161
TTATTTTAAGCAGTTGCAGTAGACTCTTCCACTTCTGTATTCAATTCTTCCTTCTTTTTTCTCT
TATAATATAAATAACATACAAAGAAAATACTTGCACTCACAAATATTGATTAAAGTTAATTAG
TGAACCCTAAGATTTATAAATCTCTAGCTATACTCTACCATATCATATATATTACATAATGTCTA
GCAGAAAATCACGTTCAAGACAAACTGGAGCTTCCATGATCACGGATGAACAAATCAACGA
TCTTGTCCTCCAGCTTCATCGGCTTCTCCCCGAACTTGCTAACAACAGACGCTCTGGAAAG
GTACAACGTTCTAATCAATTTTTCATTATGATTCCTTTTAAAAAATTTAATTTCCTCTTTCAAA
CCTTAAATCTTTTCTAAACCTTTTTAAATCTTCCTTGGATTAGTAGTGGGTACTTAAAAGAAC
AAACCTCCGGTTTAAAAAAAATAAAAATAATAACTAATATATATCCTCCATCAAAGTTTAACA
GTAATTCTTTCATCTTTCTGTTGTTGCAAACAAACGAGAGTCTTTAGTTAGAATTATAAATAA
ATATTAAACTAATATTCCATATATTCCAATATTGCATGTATGTATTGATTCATTTGATTGTGATGC
ATTTTGTGACGAGTGCTAAATTTTAATTTCCAAACTAATCGACAATAAATAGCATGCTGCATG
CATGATAATTAAATTGTTAATTATATATAAGATCCTTCTTCTTATCAATATATTGGTTACTTTCAG
GTTTCAGCATCAAGGGTATTACAAGAGACATGCAGTTACATAAGGAACTTGAGCAAAGAAG
TGGATGATCTTAGTGAAAGATTGTCTCAACTTTTGGAATCAACTGATTCAGCTCAAGCTGCA
CTAATCCGAAGTTTGCTTATGCAGTAGAATTAGTGCAATAAACGAAGGATATTTTTTTCTTTT
CTTTTTTTTGAAAAAATGTCTTCTCAGTTTTAATTCCCCTTCTTATTGTATTTATAATGTCTGG
CAGCTTGTGTCCCCTGTTAATTATGCAATGTTTTATTTTTAATATATGTAGGGTTTTGTTTTTGT
CAGTTGTTGAACGAAGCAAATGGTTTGGGTTTACGTTCACATTATTTAAG

>AtbHLH162
ATATATTACAAACCCTTTTCCAGTAAATAGTTAGCACAAACTAATTATCAGATGTCACTTAAA

CCAACCTCCTTCTTTATATAAGCTTGGTTAACATTGAGTTTGTTATCATGGTCTATGTGAAAC
AATTCAGGAATAAAAAAAGAAATGGAGCCGAGCCATTCAAACACAGGTCAATCAAGATCT
GTAGATCGCAAAACGGTTGAGAAAAATAGAAGGATGCAAATGAAGTCTCTCTACTCAGAAC
TCATCTCTCTTCTTCCTCATCATTCTTCTACGGTTTCTTCCTCTTGGCCCTCTTATATGCTTATA
TAATCTAAGATTCCATGTATGGGTGATGATCTTAATATTCTAAACTCTTTTATTATTTCATGTTA
GGAGCCTTTAACACTACCTGATCAGCTAGATGAAGCTGCAAACTACATCAAGAAGCTACAA
GTGAACGTGGAGAAAAAGAGAGAAAGGAAAAGGAACCTCGTTGCGACTACAACTTTGGA
GAAACTGAATTCCGTCGGATCTTCATCGGTTTCGTCGAGCGTCGATGTCTCCGTGCCAAGAA
AGCTGCCAAAAATCGAGATTCAAGAAACTGGTTCCATTTTTCACATCTTTCTTGTGACAAGC
TTGGAACACAAGTTTATGTTTTGTGAGATCATTCGTGTTCTCACCGAGGAATTAGGAGCTGA
GATCACTCATGCTGGATACTCCATTGTTGATGATGCTGTCTTCCACACCCTTCACTGCAAGGT
ATAATTCGGAAAGTAAAACATTTTTTGCAATGGAATTTAAGAATCAAACTTTGCTACTACTAC
TATGTTAAAACTAGGTCTAATCCAGCTTGTATACACATTGGGAATGAGTTCAAAGTTAGGGA
ATGATATTTATACTTGTTGTTTTTATATTTAATAAAACTTGCACTCAAAAACGCTAAGAAGCT
GCATGTTCCCATCTGATTGTTAAGTTTCTACTAGCTGATTCCTATTAATATCCATATGTTAGTAA
ACTAAATTTAATTTATATTTTATATAGGTGGAAGAACACGATTATGGAGCTAGGAGTCAAATT
CCTGAAAGACTGGAGAAGATTGTGAACAGTGTTCACTAATATTGATTCCTTGTTCCTTTATTA
AAAAAAAAAGTTCTATGTTTTTGTTCCTTTTCTTGAATTATTTTTGGTGTTAGCTAGATTCCTT
TTCTTGAATTGTATCATGTGCTGTGAGAGTGATGTTTCATTTGTAAGCATAATATATACTTTTC
TCTACTTAAAGCTGTGAAATATATTTTGTTTTTTGGTATCAACAAAGCTGTGAAATATATGAA
ACTTA

>AtbHLH163
GGGTGAGTTTTACACATATAACAATCAAGAAGTTGTTCTCGTGGGAAAGTAAGTTTAGAAG
CTTACCAAAAATAAACAGTATCTTTTTGTACTAAGCGACTATGAAAAAAACTGAGAACAAAT
AATAATAAATATTTGAAAGCTAAAGACACCCTTATTAAAGAAACATAACCCACACCCTCCCT
CCATGCACTTGTCTCTTCTCCTTCTTATTCTTCTTCTTCTTCTTCTTGATCTTCTCTCTCTTCAT
AAATTTGCATTATAAAAACACATCCACTTTGCTCCTCCCTCTTCAACACCTTCTTCTCCACTC
TCATTCTCTCTTTCTGACACATTAACTACTTATCCTTCTTGCATTCTTCTCTCTCTCTACACCC
AAACAAACACACTTATAATATATCAAGAAAGAAGATGTCTAGCAGAAGATCATCACGTTCAA
GACAGTCAGGAAGCTCAAGAATCTCTGACGATCAGATTTCCGATCTTGTTTCTAAGCTCCAA
CACCTCATCCCTGAACTTCGCCGCCGCCGTTCTGACAAGGTATACACTTCAAAATAACAAAT
TTTAATCTATCTTTCTTGCATATACATACTTATTTTGTATACTTGTATAACTTAGCCTGCACATG
GACTTAGAAAGTTCAAATGAATAGGAACCATATAGTATATAATAACTAATTAATATGCATGGT
GAAATCCAACGGCGTGTAGACATGTGGCCTACAATTAACTATACGTAGATAAAGTAACATTA
ATTCACAGCCTCATTTATGCATATATCTTGTTTGTTCTTTGAATGTTTTTTTGCAATTAAATTTT
ACAAGTTGGTGGTGTGGGACTCAGTTTGTTCCCTTCCTACTTCCACGTACCTTAACTACATG
TCTCCCCATTACTCTTCTCTACCTATATACTCACCATAATTCTATATATAGCTGAGGAATATGAT
AAATAAATTGTGGCCGTTTATACCGTTGTGCACATTGAAGTGGCAATCATGAGAAAAAACTA
AAGTTCTGTTAAAAGTTTGAAGATATATATGAAATTGTTACAAAGAAAGAAAAAAGGCAGT
AGCACATGTTGTCACGTACTCTTTGTAAGGGATATAATGATCGTTTTTTGTACTTTAAAACTA
ATTGTAAAATGTTTAAACTATTGGACAGGTGTCAGCATCTAAGGTACTACAAGAGACTTGCA
ACTACATCAGGAACTTACACAGAGAGGTTGATGACCTCAGTGACCGTTTGTCGGAACTCTT
GGCTTCGACGGACGACAACAGCGCCGAAGCAGCCATCATTAGGAGCTTGCTTAATTATTAA

ATCCGCATTACTTAATCTGAGAGCTATTAATCATCCGTTTCCGGCCACCAAATTTATCTTATTA
TGGGTATCGTCTGTTTACTTCTACATCATATATTATGAGATATAGCTAGGGTTTCGGGTCATTG
TTAGGCCAACTCATATATTTATATTTAATATATGGTTATGTATGTATGTATGCATGTTAATTGTAT
CTGAGGGTCCAGACCTGGCGTATAGTAGCCTGTGTATCATGAGATCCTCTAATATTTATGATT
AATGACACGGTCCGTTTCCTTTTTTACTATACTGATCTACTGTTATAAGTTTCGTTTATCATAT
CTTTTTCTATAATTAACAAGCTGAGGAGATTATCGGTG

>AtbHLH164
GAAAAATATAATTTTATTTAAACAAATTTTGTTTGAGGTTTTATCAAAGATTCAAATCTCTTTC
TCTCCCATTACATCATTGTCTAGGTTTATTGATCTCTTCCCTCTCTCCCTTGTGGTCCTTGACC
TCTTTAAATCTCTCTTATCACTTTCTCATTTTCTTTATATCTCGAAGTGTCTCTATTACCCGAA
ACACTTTCTTACAATTTTCTCTTCTCTTCTCTTTTCGTTGCTCTTCTTTTTCTTTCTTTCACAC
CTCTTCAACACAAATATAAAACCTGTAGAATAAACACAAACCTTCTACATAACTTCTCTCAC
TTTTTTTTTTTTAAAACTCTCTTCTTAATAACAAACCCTTCTCTCTCAATCTCTTCTCTATTATC
TAATCTAGAAAAGAGAGAAAGCATACAACATAAAGGTTATTTTCTTGCGGCATTGTAGTGTT
ACACCTAATCACAAAGTAAAAACAAGAAAATGTCTAACAGAAGATCAAGACAAACTTCGA
ATGCTTCGAGGATCTCCGATGACCAGATGATCGACCTCGTTAGTAAGCTCCGTCAGTTTTTG
CCGGAGATTCACGAACGGCGTCGTTCTGATAAGGTATACGACATATATACATTCATTTCACCT
CATTCATGTATATACAAAAAAAAAAAAAAAAACTGATCTTTTCGTCTACATTTAAATCAAACA
AAAGAAACCAAAAAAGTCGGAACATTTGACTCCTGACAACATTTTTGAACTTCGAAAAAG
TCTTGTTTATGTGGTGTTCGTACATGTGTGTATATATATTAATGTTCTTGATGATGTTTGTTGAA
AATGTAGAATCCTAACATTATTATTTGTTCAAAATAGGTGTCAGCATCAAAGGTACTACAAGA
GACATGCAACTACATAAGAAAATTGCATAGAGAAGTTGACAATCTCAGTGATCGTTTGTCGC
AGCTTCTTGACTCTGTTGATGAAGATAGCCCTGAAGCTGCCGTGATTAGAAGCTTACTCATG
TAACCTTCCAATATTTTTTATTATAACTTCTTAATATAGTATTTATTAATTTATCTATATATGTAAT
CTTTATCGTCCTTTATATATCAAGCGACGTGCTTTTATCTTTTATGAACTTTGGAATTTTGGTA
CAGAAATTTACATTAATTTCTAGTCAGTAG

>AtbHLH165
CCACAATCCGAAACACGAGGATAATAAAATTAAAAATAACGCACAACTGGACCCAGCTTTG
ATGGCAATACCGCTCATTTAACCATTTCCCAATTTCTAAATGTAGAATTTATTTATATCGTTTC
AATTTTTACATCGTTTTTTATAGACAAACCAAACCAAAATTCGTTCATAACCGAACCTTTCAC
TAATGTAAGCAAACAATGTAACATAAAGCATAGCAAACCAAATCAAACCGATAGAGTATTGT
CTCTAAACTCGCAACAAACCAAAATAAAATCAGGTTGAACCGACCACATTTCAACATTTTT
GTCTACTAAATCCATCATCACTCATATATATATATATAACACACACAGTTACACATATCCACATT
CCTTACTTGTTCATTATTCTCTCAGACTCATCATTCATCTCTCTCTTTCTTTCAATGGAAGAAA
CTCTAGCCACTCCCGACGCCACGAGACGCTCTCTGTCACCGTCATGCTCAGCCACCGTGAA
ATCACGCGCCGCTGGTTTCGAGCGCAGAACCAAACGGAGATTGTCAGAGACTAACGCAAG
CGTACGTGAAGACCGAGAAGAAGCAGAAGAAGAAGAAGATGAGGTCAAGGAAAAGATTG
AGGCGTTGCAGAGGATTATCCCCGGAGGAGCGGCGCTTGGTGTGGACGCGCTCTTCGAAG
AGACAGCTGGTTACATTCTGTCTCTACAATGTCAGATCAAAACCATTAAAGTCCTTACTTCG
TTTCTTCAACGCATAGATCAAGAAGACATGAAGTTCGGAGGTTGAAGATTATCACACCAAG
ACTGCTCTTCTTTCTTCGATTCCTAAAAACAAAAAAAAACGTTTTTTTTTTCAAATCTTTTA
TTTCCTTTTTTATAACATGGGATTTTAGCTTTGATATATGAGGAAAGAGACTCAACATTTTTT

CTTCGAATTTTTTTTTTCTTTAGTAGGAGTATAATCTTTAATTTTAATGTTCTGATTTATTTATTA
AGCCGTTTCACGGTTATGAGAAGTGTTCATTAACGAAAATTAGCATTAATTAATGTAGTTATA
TAACTTATTTTAGTAGGACGCCATATAGTATAGATATTAAATTGGATCACGAGGTTTCACTTGC
ATATGAGATTTGACATTAATGATATTGAGAAAGTTAATGTTACGTGGCCCATTTTTAAATCTG
GATGAGCTCATTTCAC

>AtbHLH166
TATCTATCTGTCAGGACCTCAGGGAGATCCACTTGAGTAAAGTGACGTCTCCACTAAACCAC
ACAACATAGCGCGTGACAATCACGATCCAAAATCTCTCTTAATAAATAAACCAACAAAACA
AACACTCCAACACATAATTCGAAACACTCAAGAACAAAAAAAGAAAAGAAAATCACAACT
GGACCCAGCTTTGATGGCATACCGGCATTACCCAATTTCCAAAATTATAGCCAAACCCCAAA
CCTAAATCGCACAACCAAAACATAGTACTAAACTAAAGTCTTAACCAAACGCACTAAGATT
CAGCTCATAATCAAATCATTAAAGGCAAACAAGAGTAAACCAAAGTAAAACGAACCGGAAT
GACTTTAACCGGTCCTACTTCAATCTACTAATTCACATCACTCATATAAATACACACAGGTCT
CTTTACTCCTTTTCTTCCAACACATCTTCTCTCCCTCCGTCAATGGAGAAAACCCTAGCCACT
TCCCATACCAAACGCTCTTCTCCTCCGTCTCCATCCTCCGCCGTGAACACAAGCTCCACTGG
TTTCAACCGCAGAACCAGACAGAGATTGTCCGATGCAACGGCGAGTGTAAGCGAGACCGA
CGTAGAAGATGAAGATGAAGATGAAGAAGGAGTGGAGGAGAAGATCGAGGCGCTTCAGA
CAATAGTTCCCGGAGGAACGGAACTTGGTGTCGACGCGCTGTTCGAAGAGACAGCTAGTTA
CATTTTGGCTCTGCAATGTCAGATCAATGCCATTAAAGTTCTTACTACGTTTCTTGAGCGTTG
TGAGAAAAAAGATATGAAGTTTGGAGGTTGAAGATTGAACAAACAATTTAGAATTCTGAAA
ATCAGAAGAAAAAAAAAAACAATTTGGAATTCTCTCTGTTTTTTCATTTATTTTCACCTTATA
TTTTTTTTTTTTATATATTGCTGTTTAGTTCGGGTTCATAATTTAGTTCTTAAAAAAAAAACAA
TCTTATGTGTTTTTATTCCAAAATATTACGAGAAATGAAAATTTTATCTCCACAA

>AtbHLH167
TGGAGAGAAAGTGGGGTATGTTGAACCATGTGCGTCCATGTGCTCTGTCTTTCTCTTCTAGC
CCCACTCTTAATTTCACTTCTTTTCCTTTGATCCAATAAATGCCTCCACACTCTCATTTCTTCT
ATATTAATTCTCTTCTTCAATCTAATCTCTCTCCCTCTCTCTCACTTCTTTCTAGGGTTTAAGTT
TCAGACACTATCGAGCTATTTGGATATCTTAAAACAGAAATAAGCGTAAGGTAAAAGTATTTA
TTGATTTCATTAACGTCACTTTTCTATTCACATCTACCGTTTATAATTAAAAGTGTATCTAAGG
AAATGTTTTACGACGAAGCTATATATATAGCGTATGTGCAATTATATATATCCGTTAAAATGTG
GATGATTAAAACAGTGAGTATACAATCTAACGTTTATATTCAGATCATGATATTTCCCTGAAAT
ACATTTATGTTTATTTTATTAGGATATGATGAACACGTACAACATGGTGAAGCAAGAATTTAT
CAAGAAATGGATAAATACTCTCCACATGTTAGATTCTTCTATCGAACATCCTTTGAACGTAAC
GGAAAGGAAAAATGCGATTAGACTATCATCGGACTTAGCCATGGCAGCTGCTCGAAATGGC
TCTACCGTATGGAGCCGCGCTCTTATTTCTAGGAGCGGAAATAAGACAGCAAACAAACCCA
TGGCACGTCGAATACTAAAAAAAGCTCGAAATCGGATGAAGAACCGTTGTAACATTCTTAG
ACGAAATGGCAATTTCACGGCGAAAACCTGGGTGAGAAAACGTACGGACTTGCTTAAGAG
TCTTGTACCGGGAGGTGAGTTGATAGACGACAAAGATTATTTGATAAGAGAGACACTTGAC
TACATTGTCTATCTCCGAGCACAAGTGGACGTCATGCGAACCGTCGCAGCCGTCGATTTATT
CACCCGAAACTTAACCAACGATCGTAGGAACAAATAAATGTACATGCATCACTCTTGTGAAT
ATGAAGTAGCTAATGATTTAAATGTAAGTCCATGTACATATATAGGATAGCACTATCTTATCAT
CTTACAATTTTCGAATGAATTATCGATTTACTGCTGGTCATGTAAATTATGGGTTAATTTCATA

TTATTAATTTGAAGACCACTTGAGTTGTAGCATTTCATTATATATGTAGTATGTAGTGATGTAA
ATTTAGGTTACGTTGATATAAATGTTTATAATACAACTAATTACATTATTAGTTCATTACAATAG
GGCACAAACTCTTTTCTTTTTTCGGTCTAACAAA

>AtbHLH168
GTAATATATTTGCCAAATAAGCTTACGACACAAACACAATGACACTATGACAGTAAGATATCA
TTTCAAAATACGGATATACCCCCAAATTGGTGGCAATGACAAAGAAAAAAAGAGTTCTTCA
CAGTGGCACATTCGTAATACATATGAACTTTGGTGGTTGTTTCGTAATATAGATCGTACTTAA
AACCTCTAAACACCGTTCTCTTTATTTGCCATCTTCTTCATTATCATCATCTCCATCTCTCTCT
CTCTCTCTCTCATTTTCTTGAAAAAGATGAGAACCTTAAAGACTCAGACCACAAGGGGAAG
AAGAAGAGCAAATGTGTCGTCACGTACGAGAGTTTTACACACGTGCTGTGGAAATGGTAGT
AGCGACGGAGGGAAGACGGTGATGGAGAAGCTTCTTGCGTTAAAGAGCCTTCTTCCTCCA
CCGGTGAATGTCGGTGGTGGAGAGACGGAGGAGCTGTTTCAAGAGACGGCGGAGTATATC
GTGAAGCTTAGAACACAAGTCGTGGTGTTGAAGAAACTGATTGAGATTTACGATAACTCTT
CTGATCAGAAGAAAGATGTTGTTTTATAATGTTCATTTATATTTTCTTTAATTTAAATTATTTCA
GGTTTTTCTGTTTTTTTTTGTTATAAACTTATAATTATTATATTGTGATCCGTAATGGTTTTAGG
TTGTCCATGTCTTTAATGTATTGGTTTAAAGGAAAAAGTATTGATGATTGATAACTTTCTTTTC
TTTGTTCTAGTCACACGTAGTCTAGGTGTTCTGCAGTAGTATTAATAAGTTTATTTTTTTTTGG
TTAATCTTCTTGCATTCTTTTGTTTTTA

>AtbHLH169
CGACAATCAAAGGAATGAAACCACCACCAGTGGTACTATCGTAATAAACAATAACTTAAGG
GTTATTAACGAATAACCAATCGTACATAAACCCCGATCCTTTTTCTTTTCCTCTTCAAACAAA
GAATCTAAATCCTATGTTCTTGTTCTTCACGATGAGAATCTTGAAAACTCAAAGATCCAGAG
GAGGAAGAAGAACAAGCAAGAAATTTGGAAACAGAAGAACAAGTGGAGGAGAAAAGTTC
TCGGAGAAGCTTCAGGCGCTTAAGAGTCTTCTTCCTCCGCCGTCGAAGATGACGGAGCAGA
GTCGTCAAGATGCTTACGTGGAAGAAGATTCCAGTGTTGGAGAGACGGAACAGTTGTTTCA
AGAAACTGCGGATTACATTGTCAGACTTAGGGGACAAGTCGTGGTGTTGCAAAAGTTAATC
GAGATTTATGGATCATCTGATCAGAAGGAAGATAATTTTGTTTCATAATGTTCATATTAAATTA
TGATAATGTTTTTCATTAAGCTTTTTTCAATGTGTTACGTATTTTGAACCTAATATGGTTTTTA
GGTAGTCACGATGTATAATGTTTGATTTTGACGAACGAAAAGAATGAAATCGGT

>AtbHLH170
ATGGAGAGGCAAATTATAAACAAGAGAAAGCGAGTCTTTTCTCTCCAACCAAACAAGAAC
CCTAAGGCAGTTTTCGCAAGAAGATACGTGAGTCACTTGGTTCCAGCTCTTAAAAAGATCA
ACATGAACAAATCCTCTTCAAAAACCAACAAACAAAGTTTAGAACAAACCGTGAAACATG
AAGTAGACATGGCTTTCGCATTGTCTGCTCAAGAATTCGCGTGGAGCCGTTTCTTGCAACA
GAAGCTATTATCTTCCCCTTATGATGATCCAATTAGCACTAGTAGTTCTCCTTCCGAGATTCTA
GAAAGATCGAGCAAGAGACAAGGTGGAGAAAAACACCAAGACAGCGACGAAGAAGAAG
AAGGAGGAGAGATCAAGAAGAGATTGAAGGAATTGCAGAAGCTTTTGCCAGGTGGAGAA
GAGATGAACATGGAGGAGATTTTGAGTGAGATTGGAAGCTACATTGTATGTCTTGAATTGCA
GATGATTGTTTTAAAATCTATTGTACAAGATAATACTTCTTGAATATAATATAATTCATTGTTTC
TTCTCTTCAATATGATAAGGCGAAT

169 amino acid sequences

>AtbHLH001
MGYRDEETMATGQNRTTVPENLKKHLAVSVRNIQWSYGIFWSVSASQSGVLEWGDGYYNGD
IKTRKTIQASEIKADQLGLRRSEQLSELYESLSVAESSSSGVAAGSQVTRRASAAALSPEDLADT
EWYYLVCMSFVFNIGEGMPGRTFANGEPIWLCNAHTADSKVFSRSLLAKSAAVKTVVCFPFLG
GVVEIGTTEHITEDMNVIQCVKTSFLEAPDPYATILPARSDYHIDNVLDPQQILGDEIYAPMFSTE
PFPTASPSRTTNGFDQEHEQVADDHDSFMTERITGGASQVQSWQLMDDELSNCVHQSLNSSDC
VSQTFVEGAAGRVAYGARKSRVQRLGQIQEQQRNVKTLSFDPRNDDVHYQSVISTIFKTNHQLI
LGPQFRNCDKQSSFTRWKKSSSSSSGTATVTAPSQGMLKKIIFDVPRVHQKEKLMLDSPEARDE
TGNHAVLEKKRREKLNERFMTLRKIIPSINKIDKVSILDDTIEYLQELERRVQELESCRESTDTET
RGTMTMKRKKPCDAGERTSANCANNETGNGKKVSVNNVGEAEPADTGFTGLTDNLRIGSFGN
EVVIELRCAWREGVLLEIMDVISDLHLDSHSVQSSTGDGLLCLTVNCKHKGSKIATPGMIKEAL
QRVAWIC
>AtbHLH002
MATGENRTVPDNLKKQLAVSVRNIQWSYGIFWSVSASQPGVLEWGDGYYNGDIKTRKTIQAA
EVKIDQLGLERSEQLRELYESLSLAESSASGSSQVTRRASAAALSPEDLTDTEWYYLVCMSFVF
NIGEGIPGGALSNGEPIWLCNAETADSKVFTRSLLAKSASLQTVVCFPFLGGVLEIGTTEHIKED
MNVIQSVKTLFLEAPPYTTISTRSDYQEIFDPLSDDKYTPVFITEAFPTTSTSGFEQEPEDHDSFIN
DGGASQVQSWQFVGEEISNCIHQSLNSSDCVSQTFVGTTGRLACDPRKSRIQRLGQIQEQSNHV
NMDDDVHYQGVISTIFKTTHQLILGPQFQNFDKRSSFTRWKRSSSVKTLGEKSQKMIKKILFEV
PLMNKKEELLPDTPEETGNHALSEKKRREKLNERFMTLRSIIPSISKIDKVSILDDTIEYLQDLQK
RVQELESCRESADTETRITMMKRKKPDDEEERASANCMNSKRKGSDVNVGEDEPADIGYAGLT
DNLRISSLGNEVVIELRCAWREGILLEIMDVISDLNLDSHSVQSSTGDGLLCLTVNCKHKGTKIA
TTGMIQEALQRVAWIC
>AtbHLH003
MGQKFWENQEDRAMVESTIGSEACDFFISTASASNTALSKLVSPPSDSNLQQGLRHVVEGSDW
DYALFWLASNVNSSDGCVLIWGDGHCRVKKGASGEDYSQQDEIKRRVLRKLHLSFVGSDEDH
RLVKSGALTDLDMFYLASLYFSFRCDTNKYGPAGTYVSGKPLWAADLPSCLSYYRVRSFLARS
AGFQTVLSVPVNSGVVELGSLRHIPEDKSVIEMVKSVFGGSDFVQAKEAPKIFGRQLSLGGAKP
RSMSINFSPKTEDDTGFSLESYEVQAIGGSNQVYGYEQGKDETLYLTDEQKPRKRGRKPANGR
EEALNHVEAERQRREKLNQRFYALRAVVPNISKMDKASLLADAITYITDMQKKIRVYETEKQI
MKRRESNQITPAEVDYQQRHDDAVVRLSCPLETHPVSKVIQTLRENEVMPHDSNVAITEEGVV
HTFTLRPQGGCTAEQLKDKLLASLSQ

>AtbHLH004
MSPTNVQVTDYHLNQSKTDTTNLWSTDDDASVMEAFIGGGSDHSSLFPPLPPPPLPQVNEDNL
QQRLQALIEGANENWTYAVFWQSSHGFAGEDNNNNNTVLLGWGDGYYKGEEEKSRKKKSNP
ASAAEQEHRKRVIRELNSLISGGVGGGDEAGDEEVTDTEWFFLVSMTQSFVKGTGLPGQAFSN
SDTIWLSGSNALAGSSCERARQGQIYGLQTMVCVATENGVVELGSSEIIHQSSDLVDKVDTFFN
FNNGGGEFGSWAFNLNPDQGENDPGLWISEPNGVDSGLVAAPVMNNGGNDSTSNSDSQPISKL
CNGSSVENPNPKVLKSCEMVNFKNGIENGQEEDSSNKKRSPVSNNEEGMLSFTSVLPCDSNHS
DLEASVAKEAESNRVVVEPEKKPRKRGRKPANGREEPLNHVEAERQRREKLNQRFYSLRAVVP
NVSKMDKASLLGDAISYISELKSKLQKAESDKEELQKQIDVMNKEAGNAKSSVKDRKCLNQE
SSVLIEMEVDVKIIGWDAMIRIQCSKRNHPGAKFMEALKELDLEVNHASLSVVNDLMIQQATV
KMGNQFFTQDQLKVALTEKVGECP
>AtbHLH005
MNGTTSSINFLTSDDDASAAAMEAFIGTNHHSSLFPPPPQQPPQPQFNEDTLQQRLQALIESAGE
NWTYAIFWQISHDFDSSTGDNTVILGWGDGYYKGEEDKEKKKNNTNTAEQEHRKRVIRELNS
LISGGIGVSDESNDEEVTDTEWFFLVSMTQSFVNGVGLPGESFLNSRVIWLSGSGALTGSGCER
AGQGQIYGLKTMVCIATQNGVVELGSSEVISQSSDLMHKVNNLFNFNNGGGNNGVEASSWGF
NLNPDQGENDPALWISEPTNTGIESPARVNNGNNSNSNSKSDSHQISKLEKNDISSVENQNRQSS
CLVEKDLTFQGGLLKSNETLSFCGNESSKKRTSVSKGSNNDEGMLSFSTVVRSAANDSDHSDL
EASVVKEAIVVEPPEKKPRKRGRKPANGREEPLNHVEAERQRREKLNQRFYSLRAVVPNVSKM
DKASLLGDAISYINELKSKLQQAESDKEEIQKKLDGMSKEGNNGKGCGSRAKERKSSNQDSTA
SSIEMEIDVKIIGWDVMIRVQCGKKDHPGARFMEALKELDLEVNHASLSVVNDLMIQQATVK
MGSQFFNHDQLKVALMTKVGENY
>AtbHLH006
MTDYRLQPTMNLWTTDDNASMMEAFMSSSDISTLWPPASTTTTATTETTPTPAMEIPAQAGFN
QETLQQRLQALIEGTHEGWTYAIFWQPSYDFSGASVLGWGDGYYKGEEDKANPRRRSSSPPFS
TPADQEYRKKVLRELNSLISGGVAPSDDAVDEEVTDTEWFFLVSMTQSFACGAGLAGKAFATG
NAVWVSGSDQLSGSGCERAKQGGVFGMHTIACIPSANGVVEVGSTEPIRQSSDLINKVRILFNF
DGGAGDLSGLNWNLDPDQGENDPSMWINDPIGTPGSNEPGNGAPSSSSQLFSKSIQFENGSSST
ITENPNLDPTPSPVHSQTQNPKFNNTFSRELNFSTSSSTLVKPRSGEILNFGDEGKRSSGNPDPSS
YSGQTQFENKRKRSMVLNEDKVLSFGDKTAGESDHSDLEASVVKEVAVEKRPKKRGRKPANG
REEPLNHVEAERQRREKLNQRFYALRAVVPNVSKMDKASLLGDAIAYINELKSKVVKTESEKL
QIKNQLEEVKLELAGRKASASGGDMSSSCSSIKPVGMEIEVKIIGWDAMIRVESSKRNHPAARL
MSALMDLELEVNHASMSVVNDLMIQQATVKMGFRIYTQEQLRASLISKIG
>AtbHLH007
MANNNNIPHDSISDPSPTDDFFEQILGLSNFSGSSGSGLSGIGGVGPPPMMLQLGSGNEGNHNH
MGAIGGGGPVGFHNQMFPLGLSLDQGKGHGFLKPDETGKRFQDDVLDNRCSSMKPIFHGQPM
SQPAPPMPHQQSTIRPRVRARRGQATDPHSIAERLRRERIAERIRSLQELVPTVNKTDRAAMIDEI
VDYVKFLRLQVKVLSMSRLGGAGAVAPLVTEMPLSSSVEVRLERDTSLKKEQNFGNGFSCTDL
WCKNFLQDETQAVWEKWSNDGTERQVAKLMEENVGAAMQLLQSKALCIMPISLAMAIYHSQ
PPDTSSSIVKPEMNPPP
>AtbHLH008
MPLFELFRLTKAKLESAQDRNPSPPVDEVVELVWENGQISTQSQSSRSRNIPPPQANSSRAREIG
NGSKTTMVDEIPMSVPSLMTGLSQDDDFVPWLNHHPSLDGYCSDFLRDVSSPVTVNEQESDM
AVNQTAFPLFQRRKDGNESAPAASSSQYNGFQSHSLYGSDRARDLPSQQTNPDRFTQTQEPLIT

SNKPSLVNFSHFLRPATFAKTTNNNLHDTKEKSPQSPPNVFQTRVLGAKDSEDKVLNESVASAT
PKDNQKACLISEDSCRKDQESEKAVVCSSVGSGNSLDGPSESPSLSLKRKHSNIQDIDCHSEDVE
EESGDGRKEAGPSRTGLGSKRSRSAEVHNLSERRRRDRINEKMRALQELIPNCNKVDKASMLD
EAIEYLKSLQLQVQIMSMASGYYLPPAVMFPPGMGHYPAAAAMAMGMGMPYAMGLPDLSR
GGSSVNHGPQFQVSGMQQQPVAMGIPRVSGGGIFAGSSTIGNGSTRDLSGSKDQTTTNNNSNL
KPIKRKQGSSDQFCGSS
>AtbHLH009
MLLLVSLNHKLSLALFTSAIKYILRLIKLFFLFILKCLQRSDMEHQGWSFEENYSLSTNRRSIRPQ
DELVELLWRDGQVVLQSQTHREQTQTQKQDHHEEALRSSTFLEDQETVSWIQYPPDEDPFEPD
DFSSHFFSTMDPLQRPTSETVKPKSSPEPPQVMVKPKACPDPPPQVMPPPKFRLTNSSSGIRETE
MEQYSVTTVGPSHCGSNPSQNDLDVSMSHDRSKNIEEKLNPNASSSSGGSSGCSFGKDIKEMA
SGRCITTDRKRKRINHTDESVSLSDAIGNKSNQRSGSNRRSRAAEVHNLSERRRRDRINERMKA
LQELIPHCSKTDKASILDEAIDYLKSLQLQLQVMWMGSGMAAAAASAPMMFPGVQPQQFIRQI
QSPVQLPRFPVMDQSAIQNNPGLVCQNPVQNQIISDRFARYIGGFPHMQAATQMQPMEMLRFS
SPAGQQSQQPSSVPTKTTDGSRLDH
>AtbHLH010
MEEERESLYEEMGCFDPNTPAEVTVESSFSQAEPPPPPPQVLVAGSTSNSNCSVEVEELSEFHLSP
QDCPQASSTPLQFHINPPPPPPPPCDQLHNNLIHQMASHQQQHSNWDNGYQDFVNLGPNSATT
PDLLSLLHLPRCSLPPNHHPSSMLPTSFSDIMSSSSAAAVMYDPLFHLNFPMQPRDQNQLRNGS
CLLGVEDQIQMDANGGMNVLYFEGANNNNGGFENEILEFNNGVTRKGRGSRKSRTSPTERER
RVHFNDRFFDLKNLIPNPTKIDRASIVGEAIDYIKELLRTIEEFKMLVEKKRCGRFRSKKRARVG
EGGGGEDQEEEEDTVNYKPQSEVDQSCFNKNNNNSLRCSWLKRKSKVTEVDVRIIDDEVTIKL
VQKKKINCLLFTTKVLDQLQLDLHHVAGGQIGEHYSFLFNTKICEGSCVYASGIADTLMEVVE
KQYMEAVPSNGY
>AtbHLH011
MDQPMKPKTCSESDFADDSSASSSSSSGQNLRGAEMVVEVKKEAVCSQKAEREKLRRDKLKE
QFLELGNALDPNRPKSDKASVLTDTIQMLKDVMNQVDRLKAEYETLSQESRELIQEKSELREE
KATLKSDIEILNAQYQHRIKTMVPWVPHYSYHIPFVAITQGQSSFIPYSASVNPLTEQQASVQQH
SSSSADASMKQDSKIKPLDLDLMMNSNHSGQGNDQKDDVRLKLELKIHASSLAQQVSDLFNS
FANKLFHGLTRVYFHAGCFWKREESKLDNHCKLIE
>AtbHLH012
MSLTMADGVEAAAGRSKRQNSLLRKQLALAVRSVQWSYAIFWSSSLTQPGVLEWGEGCYNG
DMKKRKKSYESHYKYGLQKSKELRKLYLSMLEGDSGTTVSTTHDNLNDDDDNCHSTSMMLS
PDDLSDEEWYYLVSMSYVFSPSQCLPGRASATGETIWLCNAQYAENKLFSRSLLARSASIQTVV
CFPYLGGVIELGVTELISEDHNLLRNIKSCLMEISAHQDNDDEKKMEIKISEEKHQLPLGISDED
LHYKRTISTVLNYSADRSGKNDKNIRHRQPNIVTSEPGSSFLRWKQCEQQVSGFVQKKKSQNV
LRKILHDVPLMHTKRMFPSQNSGLNQDDPSDRRKENEKFSVLRTMVPTVNEVDKESILNNTIK
YLQELEARVEELESCMGSVNFVERQRKTTENLNDSVLIEETSGNYDDSTKIDDNSGETEQVTVF
RDKTHLRVKLKETEVVIEVRCSYRDYIVADIMETLSNLHMDAFSVRSHTLNKFLTLNLKAKFR
GAAVASVGMIKRELRRVIDFREPICDVPLSLHQVFRVFVCKVCQSLVGIFDNVVSSSSTKPRSILI
HNSWAICIFH
>AtbHLH013
MNIGRLVWNEDDKAIVASLLGKRALDYLLSNSVSNANLLMTLGSDENLQNKLSDLVERPNAS
NFSWNYAIFWQISRSKAGDLVLCWGDGYCREPKEGEKSEIVRILSMGREEETHQTMRKRVLQK

LHDLFGGSEEENCALGLDRVTDTEMFLLSSMYFSFPRGEGGPGKCFASAKPVWLSDVVNSGSD
YCVRSFLAKSAGIQTVVLVPTDLGVVELGSTSCLPESEDSILSIRSLFTSSLPPVRAVALPVTVAE
KIDDNRTKIFGKDLHNSGFLQHHQHHQQQQQPPQQQQHRQFREKLTVRKMDDRAPKRLDA
YPNNGNRFMFSNPGTNNNTLLSPTWVQPENYTRPINVKEVPSTDEFKFLPLQQSSQRLLPPAQ
MQIDFSAASSRASENNSDGEGGGEWADAVGADESGNNRPRKRGRRPANGRAEALNHVEAER
QRREKLNQRFYALRSVVPNISKMDKASLLGDAVSYINELHAKLKVMEAERERLGYSSNPPISLD
SDINVQTSGEDVTVRINCPLESHPASRIFHAFEESKVEVINSNLEVSQDTVLHTFVVKSEELTKE
KLISALSREQTNSVQSRTSSGR
>AtbHLH014
MYNLTFSPSLSSSLLSFTQQTPAAIVSSSPPDLVLQQKLRFVVETSPDRWAYVIFWQKMFDDQSD
RSYLVWVDGHFCGNKNNNSQENYTTNSIECELMMDGGDDLELFYAASFYGEDRSPRKEVSDE
SLVWLTGPDELRFSNYERAKEAGFHGVHTLVSIPINNGIIELGSSESIIQNRNFINRVKSIFGSGKT
TKHTNQTGSYPKPAVSDHSKSGNQQFGSERKRRRKLETTRVAAATKEKHHPAVLSHVEAEKQR
REKLNHRFYALRAIVPKVSRMDKASLLSDAVSYIESLKSKIDDLETEIKKMKMTETDKLDNSSS
NTSPSSVEYQVNQKPSKSNRGSDLEVQVKIVGEEAIIRVQTENVNHPTSALMSALMEMDCRVQ
HANASRLSQVMVQDVVVLVPEGLRSEDRLRTTLVRTLSL
>AtbHLH015
MHHFVPDFDTDDDYVNNHNSSLNHLPRKSITTMGEDDDLMELLWQNGQVVVQNQRLHTKKP
SSSPPKLLPSMDPQQQPSSDQNLFIQEDEMTSWLHYPLRDDDFCSDLLFSAAPTATATATVSQVT
AARPPVSSTNESRPPVRNFMNFSRLRGDFNNGRGGESGPLLSKAVVRESTQVSPSATPSAAASE
SGLTRRTDGTDSSAVAGGGAYNRKGKAVAMTAPAIEITGTSSSVVSKSEIEPEKTNVDDRKRKER
EATTTDETESRSEETKQARVSTTSTKRSRAAEVHNLSERKRRDRINERMKALQELIPRCNKSDK
ASMLDEAIEYMKSLQLQIQMMSMGCGMMPMMYPGMQQYMPHMAMGMGMNQPIPPPSFMP
FPNMLAAQRPLPTQTHMAGSGPQYPVHASDPSRVFVPNQQYDPTSGQPQYPAGYTDPYQQFR
GLHPTQPPQFQNQATSYPSSSRVSSSKESEDHGNHTTG
>AtbHLH016
MSQCVPNCHIDDTPAAATTTVRSTTAADIPILDYEVAELTWENGQLGLHGLGPPRVTASSTKYS
TGAGGTLESIVDQATRLPNPKPTDELVPWFHHRSSRAAMAMDALVPCSNLVHEQQSKPGGVGS
TRVGSCSDGRTMGGGKRARVAPEWSGGGSQRLTMDTYDVGFTSTSMGSHDNTIDDHDSVCHS
RPQMEDEEEKKAGGKSSVSTKRSRAAAIHNQSERKRRDKINQRMKTLQKLVPNSSKTDKASM
LDEVIEYLKQLQAQVSMMSRMNMPSMMLPMAMQQQQQLQMSLMSNPMGLGMGMGMPGL
GLLDLNSMNRAAASAPNIHANMMPNPFLPMNCPSWDASSNDSRFQSPLIPDPMSAFLACSTQP
TTMEAYSRMATLYQQMQQQLPPPSNPK
>AtbHLH017
MNMSDLGWDDEDKSVVSAVLGHLASDFLRANSNSNQNLFLVMGTDDTLNKKLSSLVDWPNS
ENFSWNYAIFWQQTMSRSGQQVLGWGDGCCREPNEEEESKVVRSYNFNNMGAEEETWQDM
RKRVLQKLHRLFGGSDEDNYALSLEKVTATEIFFLASMYFFFNHGEGGPGRCYSSGKHVWLSD
AVNSESDYCFRSFMAKSAGIRTIVMVPTDAGVLELGSVWSLPENIGLVKSVQALFMRRVTQPV
MVTSNTNMTGGIHKLFGQDLSGAHAYPKKLEVRRNLDERFTPQSWEGYNNNKGPTFGYTPQR
DDVKVLENVNMVVDNNNYKTQIEFAGSSVAASSNPSTNTQQEKSESCTEKRPVSLLAGAGIVS
VVDEKRPRKRGRKPANGREEPLNHVEAERQRREKLNQRFYALRSVVPNISKMDKASLLGDAIS
YIKELQEKVKIMEDERVGTDKSLSESNTITVEESPEVDIQAMNEEVVVRVISPLDSHPASRIIQA
MRNSNVSLMEAKLSLAEDTMFHTFVIKSNNGSDPLTKEKLIAAFYPETSSTQPPLPSSSSQVSGD
I

>AtbHLH018
MATAMNVFSTKWSSELDIEEYSIIHQFHMNSLVGDVPQSLSSLDDTTTCYNLDASCNKSLVEER
PSKILKTTHISPNLHPFSSSNPPPPKHQPSSRILSFEKTGLHVMNHNSPNLIFSPKDEEIGLPEHKK
AELIIRGTKRAQSLTRSQSNAQDHILAERKRREKLTQRFVALSALIPGLKKMDKASVLGDAIKHI
KYLQESVKEYEEQKKEKTMESVVLVKKSSLVLDENHQPSSSSSSDGNRNSSSSNLPEIEVRVSG
KDVLIKILCEKQKGNVIKIMGEIEKLGLSITNSNVLPFGPTFDISIIAQKNNNFDMKIEDVVKNLS
FGLSKLT
>AtbHLH019
MDEDFFLPDFSLVDIDFDFNIYEENNLSPDESLSNSRRADQSSKFDHQMHFECLREKPKAAVKP
MMKINNKQQLISFDFSSNVISSPAAEEIIMDKLVGRGTKRKTCSHGTRSPVLAKEHVLAERKRR
EKLSEKFIALSALLPGLKKADKVTILDDAISRMKQLQEQLRTLKEEKEATRQMESMILVKKSKV
FFDEEPNLSCSPSVHIEFDQALPEIEAKISQNDILIRILCEKSKGCMINILNTIENFQLRIENSIVLPF
GDSTLDITVLAQMDKDFSMSILKDLVRNLRLAMV
>AtbHLH020
MDDSSFMDLMIDTDEYLIDDWESDFPICGETNTNPGSESGSGTGFELLAERPTKQMKTNNNMN
STSSSPSSSSSSGSRTSQVISFGSPDTKTNPVETSLNFSNQVSMDQKVGSKRKDCVNNGGRREPH
LLKEHVLAERKRRQKLNERLIALSALLPGLKKTDKATVLEDAIKHLKQLQERVKKLEEERVVT
KKMDQSIILVKRSQVYLDDDSSSYSSTCSAASPLSSSSDEVSIFKQTMPMIEARVSDRDLLIRVH
CEKNKGCMIKILSSLEKFRLEVVNSFTLPFGNSTLVITILTKMDNKFSRPVEEVVKNIRVALAE
>AtbHLH021
MESNMQNLLEKLRPLVGARAWDYCVLWRLNEDQRFVKWMGCCCGGTELIAENGTEEFSYGG
CRDVMFHHPRTKSCEFLSHLPASIPLDSGIYAETLLTNQTGWLSESSEPSFMQETICTRVLIPIPGG
LVELFATRHVAEDQNVVDFVMGHCNMLMDDSVTINMMVADEVESKPYGMLSGDIQQKGSKE
EDMMNLPSSYDISADQIRLNFLPQMSDYETQHLKMKSDYHHQALGYLPENGNKEMMGMNPF
NTVEEDGIPVIGEPSLLVNEQQVVNDKDMNENGRVDSGSDCSDQIDDEDDPKYKKKSGKGSQ
AKNLMAERRRRKKLNDRLYALRSLVPRITKLDRASILGDAINYVKELQNEAKELQDELEENSE
TEDGSNRPQGGMSLNGTVVTGFHPGLSCNSNVPSVKQDVDLENSNDKGQEMEPQVDVAQLD
GREFFVKVICEYKPGGFTRLMEALDSLGLEVTNANTTRYLSLVSNVFKVEKNDNEMVQAEHV
RNSLLEITRNTSRGWQDDQMATGSMQNEKNEVDYQHYDDHQHHNGHHHPFDHQMNQSAHH
HHHHQHINHYHNQ
>AtbHLH022
MGGGSRFQEPVRMSRRKQVTKEKEEDENFKSPNLEAERRRREKLHCRLMALRSHVPIVTNMT
KASIVEDAITYIGELQNNVKNLLETFHEMEEAPPEIDEEQTDPMIKPEVETSDLNEEMKKLGIEE
NVQLCKIGERKFWLKIITEKRDGIFTKFMEVMRFLGFEIIDISLTTSNGAILISASVQTQELCDVE
QTKDFLLEVMRSNP
>AtbHLH023
MTWKPKMLILSHDLISPEKYIMGEDDIVELLGKSSQVVTSSQTQTPSCDPPLILRGSGSGDGEG
NGPLPQPPPPLYHQQSLFIQEDEMASWLHQPNRQDYLYSQLLYSGVASTHPQSLASLEPPPPPRA
QYILAADRPTGHILAERRAENFMNISRQRGNIFLGGVEAVPSNSTLLSSATESIPATHGTESRATV
TGGVSRTFAVPGLGPRGKAVAIETAGTQSWGLCKAETEPVQRQPATETDITDERKRKTREETNV
ENQGTEEARDSTSSKRSRAAIMHKLSERRRRQKINEMMKALQELLPRCTKTDRSSMLDDVIEY
VKSLQSQIQMFSMGHVMIPPMMYAGNIQQQYMPHMAMGMNRPPAFIPFPRQAHMAEGVGPV
DLFRENEETEQETMSLLLREDKRTKQKMFS
>AtbHLH024

MISQREEREEKKQRVMGDKKLISSSSSSSVYDTRINHHLHHPPSSSDEISQFLRHIFDRSSPLPSY
YSPATTTTTASLIGVHGSGDPHADNSRSLVSHHPPSDSVLMSKRVGDFSEVLIGGGSGSAAACF
GFSGGGNNNNVQGNSSGTRVSSSSVGASGNETDEYDCESEEGGEAVVDEAPSSKSGPSSRSSSK
RCRAAEVHNLSEKRRRSRINEKMKALQSLIPNSNKTDKASMLDEAIEYLKQLQLQVQMLTMR
NGINLHPLCLPGTTLHPLQLSQIRPPEATNDPLLNHTNQFASTSNAPEMINTVASSYALEPSIRSH
FGPFPLLTSPVEMSREGGLTHPRLNIGHSNANITGEQALFDGQPDLKDRIT
>AtbHLH025
MSILSTRWFSEQEIEENSIIQQFHMNSIVGEVQEAQYIFPHSFTTNNDPSYDDLIEMKPPKILETT
YISPSSHLPPNSKPHHIHRHSSSRILSFEDYGSNDMEHEYSPTYLNSIFSPKLEAQVQPHQKSDEF
NRKGTKRAQPFSRNQSNAQDHIIAERKRREKLTQRFVALSALVPGLKKMDKASVLGDALKHIK
YLQERVGELEEQKKERRLESMVLVKKSKLILDDNNQSFSSSCEDGFSDLDLPEIEVRFSDEDVLI
KILCEKQKGHLAKIMAEIEKLHILITNSSVLNFGPTLDITIIAKKESDFDMTLMDVVKSLRSALSN
FI
>AtbHLH026
MSNNQAFMELGWRNDVGSLAVKDQGMMSERARSDEDRLINGLKWGYGYFDHDQTDNYLQI
VPEIHKEVENAKEDLLVVVPDEHSETDDHHHIKDFSERSDHRFYLRNKHENPKKRRIQVLSSD
DESEEFTREVPSVTRKGSKRRRRDEKMSNKMRKLQQLVPNCHKTDKVSVLDKTIEYMKNLQL
QLQMMSTVGVNPYFLPATLGFGMHNHMLTAMASAHGLNPANHMMPSPLIPALNWPLPPFTNIS
FPHSSSQSLFLTTSSPASSPQSLHGLVPYFPSFLDFSSHAMRRL
>AtbHLH027
MEDLDHEYKNYWETTMFFQNQELEFDSWPMEEAFSGSGESSSPDGAATSPASSKNVVSERNR
RQKLNQRLFALRSVVPNISKLDKASVIKDSIDYMQELIDQEKTLEAEIRELESRSTLLENPVRDY
DCNFAETHLQDFSDNNDMRSKKFKQMDYSTRVQHYPIEVLEMKVTWMGEKTVVVCITCSKK
RETMVQLCKVLESLNLNILTTNFSSFTSRLSTTLFLQVTLSLSPSLISLFGNVITSTNYKILNASRE
YCTCLVLV
>AtbHLH028
MINTDDNLLMIEALLTSDPSPPLLPANLSLETTLPKRLHAVLNGTHEPWSYAIFWKPSYDDFSGE
AVLKWGDGVYTGGNEEKTRGRLRRKKTILSSPEEKERRSNVIRELNLMISGEAFPVVEDDVSD
DDDVEVTDMEWFFLVSMTWSFGNGSGLAGKAFASYNPVLVTGSDLIYGSGCDRAKQGGDVG
LQTILCIPSHNGVLELASTEEIRPNSDLFNRIRFLFGGSKYFSGAPNSNSELFPFQLESSCSSTVTG
NPNPSPVYLQNRYNLNFSTSSSTLARAPCGDVLSFGENVKQSFENRNPNTYSDQIQNVVPHATV
MLEKKKGKKRGRKPAHGRDKPLNHVEAERMRREKLNHRFYALRAVVPNVSKMDKTSLLEDA
VCYINELKSKAENVELEKHAIEIQFNELKEIAGQRNAIPSVCKYEEKASEMMKIEVKIMESDDA
MVRVESRKDHHPGARLMNALMDLELEVNHASISVMNDLMIQQANVKMGLRIYKQEELRDLL
MSKIS
>AtbHLH029
MEGRVNALSNINDLELHNFLVDPNFDQFINLIRGDHQTIDENPVLDFDLGPLQNSPCFIDENQFIP
TPVDDLFDELPDLDSNVAESFRSFDGDSVRAGGEEDEEDYNDGDDSSATTTNNDGTRKTKTDR
SRTLISERRRRGRMKDKLYALRSLVPNITKMDKASIVGDAVLYVQELQSQAKKLKSDIAGLEAS
LNSTGGYQEHAPDAQKTQPFRGINPPASKKIIQMDVIQVEEKGFYVRLVCNKGEGVAPSLYKSL
ESLTSFQVQNSNLSSPSPDTYLLTYTLDGTCFEQSLNLPNLKLWITGSLLNQGFEFIKSFT
>AtbHLH030
MCAKKEEEEEEEEDSSEAMNNIQNYQNDLFFHQLISHHHHHHDPSQSETLGASGNVGSGFTI
FSQDSVSPIWSLPPPTSIQPPFDQFPPPSSSPASFYGSFFNRSRAHHQGLQFGYEGFGGATSAAHH

HHEQLRILSEALGPVVQAGSGPFGLQAELGKMTAQEIMDAKALAASKSHSEAERRRRERINNH
LAKLRSILPNTTKTDKASLLAEVIQHVKELKRETSVISETNLVPTESDELTVAFTEEEETGDGRFV
IKASLCCEDRSDLLPDMIKTLKAMRLKTLKAEITTVGGRVKNVLFVTGEESSGEEVEEEYCIGTI
EEALKAVMEKSNVEESSSSGNAKRQRMSSHNTITIVEQQQQYNQR
>AtbHLH031
MDPSGMMNEGGPFNLAEIWQFPLNGVSTAGDSSRRSFVGPNQFGDADLTTAANGDPARMSHA
LSQAVIEGISGAWKRREDESKSAKIVSTIGASEGENKRQKIDEVCDGKAEAESLGTETEQKKQQ
MEPTKDYIHVRARRGQATDSHSLAERARREKISERMKILQDLVPGCNKVIGKALVLDEIINYIQS
LQRQVEFLSMKLEAVNSRMNPGIEVFPPKEVMILMIINSIFSIFFTKQYMFLSRYSRGRSLDVYA
VRSFKHCNKRSDLCFCSCSPKTELKTTIFSQNMTCFCRYSRVGVAISSSKHCNEPVTLCFYSYCL
RKIYHFLLWNLKYKIQKSVLFS
>AtbHLH032
MYAMKEEDCLQTFHNLQDYQDQFHLHHHPQILPWSSTSLPSFDPLHFPSNPTRYSDPVHYFNR
RASSSSSSFDYNDGFVSPPPSMDHPQNHLRILSEALGPIMRRGSSFGFDGEIMGKLSAQEVMDA
KALAASKSHSEAERRRRERINTHLAKLRSILPNTTKTDKASLLAEVIQHMKELKRQTSQITDTY
QVPTECDDLTVDSSYNDEEGNLVIRASFCCQDRTDLMHDVINALKSLRLRTLKAEIATVGGRVK
NILFLSREYDDEEDHDSYRRNFDGDDVEDYDEERMMNNRVSSIEEALKAVIEKCVHNNDESND
NNNLEKSSSGGIKRQRTSKMVNRCYN
>AtbHLH033
MNSDGVWLDGSGESPEVNNGEAASWVRNPDEDWFNNPPPPQHTNQNDFRFNGGFPLNPSEN
LLLLLQQSIDSSSSSSPLLHPFTLDAASQQQQQQQQQEQSFLATKACIVSLLNVPTINNNTFDD
FGFDSGFLGQQFHGNHQSPNSMNFTGLNHSVPDFLPAPENSSGSCGLSPLFSNRAKVLKPLQV
MASSGSQPTLFQKRAAMRQSSSSKMCNSESSSEMRKSSYEREIDDTSTGIIDISGLNYESDDHNT
NNNKGKKKGMPAKNLMAERRRRKKLNDRLYMLRSVVPKISKMDRASILGDAIDYLKELLQRI
NDLHTELESTPPSSSSLHPLTPTPQTLSYRVKEELCPSSSLPSPKGQQPRVEVRLREGKAVNIHMF
CGRRPGLLLSTMRALDNLGLDVQQAVISCFNGFALDVFRAEQCQEDHDVLPEQIKAVLLDTAG
YAGLV
>AtbHLH034
MYPSIEDDDDLLAALCFDQSNGVEDPYGYMQTNEDNIFQDFGSCGVNLMQPQQEQFDSFNGN
LEQVCSSFRGGNNGVVYSSSIGSAQLDLAASFSGVLQQETHQVCGFRGQNDDSAVPHLQQQQ
GQVFSGVVEINSSSSVGAVKEEFEEECSGKRRRTGSCSKPGTKACREKLRREKLNDKFMDLSSV
LEPGRTPKTDKSAILDDAIRVVNQLRGEAHELQETNQKLLEEIKSLKADKNELREEKLVLKAEK
EKMEQQLKSMVVPSPGFMPSQHPAAFHSHKMAVAYPYGYYPPNMPMWSPLPPADRDTSRDLK
NLPPVA
>AtbHLH035
MEDIVDQELSNYWEPSSFLQNEDFEYDRSWPLEEAISGSYDSSSPDGAASSPASKNIVSERNRR
QKLNQRLFALRSVVPNITKMDKASIIKDAISYIEGLQYEEKKLEAEIRELESTPKSSLSFSKDFDR
DLLVPVTSKKMKQLDSGSSTSLIEVLELKVTFMGERTMVVSVTCNKRTDTMVKLCEVFESLNL
KILTSNLTSFSGMIFHTVFIELRPNIYWVVWFLVFMSIFGPTIIVIWSIWFIKKKIILSLWRMKKNK
RCCG
>AtbHLH036
MDDCRDKRRRRCTKLTCGTDNNDMEKMMHRETERQRRQEMASLYASLRSLLPLHFIKGKRST
SDQVNEAVNYIKYLQRKIKELSVRRDDLMVLSRGSLLGSSNGDFKEDVEMISGKNHVVVRQCL
VGVEIMLSSRCCGGQPRFSSVLQVLSEYGLCLLNSISSIVDDRLVYTIQAEVNDMALMIDLAEL

EKRLIRMK
>AtbHLH037
MDNSDILMNMMMQQMEKLPEHFSNSNPNPNPHNIMMLSESNTHPFFFNPTHSHLPFDQTMPH
HQPGLNFRYAPSPSSSLPEKRGGCSDNANMAAMREMIFRIAVMQPIHIDPESVKPPKRKNVRIS
KDPQSVAARHRRERISERIRILQRLVPGGTKMDTASMLDEAIHYVKFLKKQVQSLEEHAVVNG
GGMTAVAGGALAGTVGGGYGGKGCGIMRSDHHQMLGNAQILR
>AtbHLH038
MCALVPSFFTNFGWPSTNQYESYYGAGDNLNNGTFLELTVPQTYEVTHHQNSLGVSVSSEGNE
IDNNPVVVKKLNHNASERDRRKKINTLFSSLRSCLPASDQSKKLSIPETVSKSLKYIPELQQQVK
RLIQKKEEILVRVSGQRDFELYDKQQPKAVASYLSTVSATRLGDNEVMVQVSSSKIHNFSISNVL
GGIEEDGFVLVDVSSSRSQGERLFYTLHLQVENMDDYKINCEELSERMLYLYEKCENSFN
>AtbHLH039
MCALVPPLFPNFGWPSTGEYDSYYLAGDILNNGGFLDFPVPEETYGAVTAVTQHQNSFGVSVS
SEGNEIDNNPVVVKKLNHNASERDRRRKINSLFSSLRSCLPASGQSKKLSIPATVSRSLKYIPELQ
EQVKKLIKKKEELLVQISGQRNTECYVKQPPKAVANYISTVSATRLGDNEVMVQISSSKIHNFSI
SNVLSGLEEDRFVLVDMSSSRSQGERLFYTLHLQVEKIENYKLNCEELSQRMLYLYEECGNSYI
>AtbHLH040
MENGMYKKKGVCDSCVSSKSRSNHSPKRSMMEPQPHHLLMDWNKANDLLTQEHAAFLNDP
HHLMLDPPPETLIHLDEDEEYDEDMDAMKEMQYMIAVMQPVDIDPATVPKPNRRNVRISDDP
QTVVARRRRERISEKIRILKRIVPGGAKMDTASMLDEAIRYTKFLKRQVRILQPHSQIGAPMANP
SYLCYYHNSQP
>AtbHLH041
MDAFFLTDDPNTRNQLIRSLAQSFGCVYVCLWSYYFPRPSNYLISMDGYYNEASEEPSSSSSSG
SLARSLFHEYRQSVIPLQNGHVPSMAFMNNLPYVEIRPQESQRLAFNDTQRLFYQEARIQTVIF
MGCRSGEIELGMTYDTTNMKIEASLREWFPEDFNRKSSPANSDYLRPPHYPSSSSSSLSPNNISE
YSSLLFPLIPKPSTTTEAVNVPVLPPLAPINMIHPQHQEPLFRNRQREEEAMTQAILAVLTGPSSPP
STSSSPQRKGRATAFKRYYSMISDRGRAPLPSVRKQSMMTRAMSFYNRLNINQRERFTRENATT
HGEGSGGSGGGGRYTSGPSATQLQHMISERKRREKLNESFQALRSLLPPGTKKDKASVLSIARE
QLSSLQGEISKLLERNREVEAKLAGEREIENDLRPEERFNVRIRHIPESTSRERTLDLRVVLRGDII
RVDDLMIRLLEFLKQINNVSLVSIEARTLARAEGDTSIVLVISLRLKIEGEWDESAFQEAVRRVVA
DLAH
>AtbHLH042
MDESSIIPAEKVAGAEKKELQGLLKTAVQSVDWTYSVFWQFCPQQRVLVWGNGYYNGAIKTR
KTTQPAEVTAEEAALERSQQLRELYETLLAGESTSEARACTALSPEDLTETEWFYLMCVSFSFPP
PSGMPGKAYARRKHVWLSGANEVDSKTFSRAILAKSAKIQTVVCIPMLDGVVELGTTKKVRE
DVEFVELTKSFFYDHCKTNPKPALSEHSTYEVHEEAEDEEEVEEEMTMSEEMRLGSPDDEDVS
NQNLHSDLHIESTHTLDTHMDMMNLMEEGGNYSQTVTTLLMSHPTSLLSDSVSTSSYIQSSFAT
WRVENGKEHQQVKTAPSSQWVLKQMIFRVPFLHDNTKDKRLPREDLSHVVAERRRREKLNEK
FITLRSMVPFVTKMDKVSILGDTIAYVNHLRKRVHELENTHHEQQHKRTRTCKRKTSEEVEVSI
IENDVLLEMRCEYRDGLLLDILQVLHELGIETTAVHTSVNDHDFEAEIRAKVRGKKASIAEVKR
AIHQVIIHDTNL
>AtbHLH043
MNNYNMNPSLFQNYTWNNIINSSNNNNKNDDHHHQHNNDPIGMAMDQYTQLHIFNPFSSSHF
PPLSSSLTTTTLLSGDQEDDEDEEEPLEELGAMKEMMYKIAAMQSVDIDPATVKKPKRRNVRIS

DDPQSVAARHRRERISERIRILQRLVPGGTKMDTASMLDEAIRYVKFLKRQIRLLNNNTGYTPPP
PQDQASQAVTTSWVSPPPPPSFGRGGRGVGELI
>AtbHLH044
MANFENLSSDFQTIAMDIYSSITQAADLNNNNSNLHFQTFHPSSTSLESLFLHHHQQQLLHFPG
NSPDSSNNFSSTSSFLHSDHNIVDETKKRKALLPTLSSSETSGVSDNTNVIATETGSLRRGKRLK
KKKEEEDEKEREVVHVRARRGQATDSHSLAERVRRGKINERLRCLQDMVPGCYKAMGMATM
LDEIINYVQSLQNQVEFLSMKLTAASSFYDFNSETDAVDSMQRAKARETVEMGRQTRDGSPVF
HLSTWSL
>AtbHLH045
MSHIAVERNRRRQMNEHLKSLRSLTPCFYIKRGDQASIIGGVIEFIKELQQLVQVLESKKRRKTL
NRPSFPYDHQTIEPSSLGAATTRVPFSRIENVMTTSTFKEVGACCNSPHANVEAKISGSNVVLRV
VSRRIVGQLVKIISVLEKLSFQVLHLNISSMEETVLYFFVVKIGLECHLSLEELTLEVQKSFVSDE
VIVSTN
>AtbHLH046
MELPQPRPFKTQEFRTGRKPTHDFLSLCSHSTVHPDPKPTPPPSSQGSHLKTHDFLQPLECVGA
KEDVSRINSTTTASEKPPPPAPPPPLQHVLPGGIGTYTISPIPYFHHHHQRIPKPELSPPMMFNANE
RNVLDENSNSNCSSYAAASSGFTLWDESASGKKGQTRKENSVGERVNMRADVAATVGQWPV
AERRSQSLTNNHMSGFSSLSSSQGSVLKSQSFMDMIRSAKGSSQEDDLDDEEDFIMKKESSSTS
QSHRVDLRVKADVRGSPNDQKLNTPRSKHSATEQRRRSKINDRFQMLRQLIPNSDQKRDKASF
LLEVIEYIQFLQEKADKYVTSYQGWNHEPAKLLNWQSNNNQQLVPEGVAFAPKLEEEKNNIPV
SVLATAQGVVIDHPTTATTSPFPLSIQSNSFFSPVIAGNPVPQFHARVASSEAVEPSPSSRSQKEEE
DEEVLEGNIRISSVYSQGLVKTLREALENSGVDLTKASISVEIELAKQSSSSSFKDHEVREPVSRT
RNDNVKQTRKPKRLKTGQ
>AtbHLH047
MVSKTPSTSSDEANATADERCRKGKVPKRINKAVRERLKREHLNELFIELADTLELNQQNSGK
ASILCEATRFLKDVFGQIESLRKEHASLLSESSYVTTEKNELKEETSVLETEISKLQNEIEARANQ
SKPDLNTSPAPEYHHHHYQQQHPERVSQFPGLPIFQGPGFQQSATTLHPPATVLVLPIQPDPQTQ
DISEMTQAQQPLMFNSSNVSKPCPRYASAADSWSSRLLGERLKASE
>AtbHLH048
MDLTQGFRARSGVVGPVAGLESLNFSDEFRHLVTTMPPETTGGSFTALLEMPVTQAMELLHFP
DSSSSQARTVTSGDISPTTLHPFGALTFPSNSLLLDRAARFSVIATEQNGNFSGETANSLPSNPGA
NLDRVKAEPAETDSMVENQNQSYSSGKRKEREKKVKSSTKKNKSSVESDKLPYVHVRARRGQ
ATDNHSLAERARREKINARMKLLQELVPGCDKIQGTALVLDEIINHVQTLQRQVEMLSMRLAA
VNPRIDFNLDSILASENGSLMDGSFNAESYHQLQQWPFDGYHQPEWGREEDHHQANFSMGSA
TLHPNQVKMEL
>AtbHLH049
MDLSAKDEFSAEKRNPDNYDSVNNPSGDWRVDSYPSENLISAGPASCSPSQMMDSFGQTLWY
DPTSVQAVGYAGFNGGNASSSSFRGSIDRSLEMGWNLPNLLPPKGNGLFLPNASSFLPPSMAQF
PADSGFIERAARFSLFSGGNFSDMVNQPLGNSEAIGLFLQGGGTMQGQCQSNELNVGEPHNDV
SVAVKESTVRSSEQAKPNVPGSGNVSEDTQSSGGNGQKGRETSSNTKKRKRNGQKNSEAAQS
HRSQQSEEEPDNNGDEKRNDEQSPNSPGKKSNSGKQQGKQSSDPPKDGYIHVRARRGQATNS
HSLAERVRREKISERMKFLQDLVPGCNKVTGKAVMLDEIINYVQSLQRQVEFLSMKLATVNPQ
MDFNLEGLLAKDALQLRAGSSSTTPFPPNMSMAYPPLPHGFMQQTLSSIGRTITSPLSPMNGGF
KRQETNGWEGDLQNVIHINYGAGDVTPDPQAAATASLPAANMKVEP

>AtbHLH050
MANLSSDFQTFTMDDPIRQLAELSNTLHHFQTFPPPFSSSLDSLFFHNQFPDHFPGKSLENNFHQ
GIFFPSNIQNNEESSSQFDTKKRKSLMEAVSTSENSVSDQTLSTSSAQVSINGNISTKNNSSRRGK
RSKNREEEKEREVVHVRARRGQATDSHSIAERVRRGKINERLKCLQDIVPGCYKTMGMATML
DEIINYVQSLQNQVEFLSMKLTAASSYYDFNSETDAVESMQKAKAREAVEMGQGRDGSSVFHS
SSWTL
>AtbHLH051
MENSYDSSKWSDSTTPYMVSWSLQSESSDSDWNRFNLGFSSSSFGGNFPADDCVGGIEKAESL
SRSHRLAEKRRRDRINSHLTALRKLVPNSDKLDKAALLATVIEQVKELKQKAAESPIFQDLPTE
ADEVTVQPETISDFESNTNTIIFKASFCCEDQPEAISEIIRVLTKLQLETIQAEIISVGGRMRINFILK
DSNCNETTNIAASAKALKQSLCSALNRITSSSTTTSSVCRIRSKRQRWFLSSHYSHNE
>AtbHLH052
MIIPETDSFFFQEQPQHQPLYPDEALSPSLFGFDHYDHFYESFLPSQEIFLPSPKTRVFNESQELDS
FHTPKHQKLIDSSFHFNSHDPFSPSPESNYLLDSYITEASNISKFQAPDFSSTFKVGWTEQGDTK
KRELSAQSIAARKRRRRITEKTQELGKLIPGSQKHNTAEMFNAAAKYVKFLQAQIEILQLKQTK
MQTLDSSKVGREMQFLLGSQEIQEKLSTEEVCVVPREMVQVLKAEECILTNPKISRDINKLLST
NLMN
>AtbHLH053
MSMDCLSYFFNYDPPVQLQDCFIPEMDMIIPETDSFFFQSQPQLEFHQPLFQEEAPSQTHFDPFC
DQFLSPQEIFLPNPKNEIFNETHDLDFFLPTPKRQRLVNSSYNCNTQNHFQSRNPNFFDPFGDTD
FVPESCTFQEFRVPDFSLAFKVGRGDQDDSKKPTLSSQSIAARGRRRRIAEKTHELGKLIPGGNK
LNTAEMFQAAAKYVKFLQSQVGILQLMQTTKKGSSNVQMETQYLLESQAIQEKLSTEEVCLVP
CEMVQDLTTEETICRTPNISREINKLLSKHLAN
>AtbHLH054
MDVFVDGELESLLGMFNFDQCSSSKEERPRDELLGLSSLYNGHLHQHQHHNNVLSSDHHAFL
LPDMFPFGAMPGGNLPAMLDSWDQSHHLQETSSLKRKLLDVENLCKTNSNCDVTRQELAKSK
KKQRVSSESNTVDESNTNWVDGQSLSNSSDDEKASVTSVKGKTRATKGTATDPQSLYARKRRE
KINERLKTLQNLVPNGTKVDISTMLEEAVHYVKFLQLQIKLLSSDDLWMYAPLAYNGLDMGFH
HNLLSRLM
>AtbHLH055
MNFPDSSLFTPNFAYENDLDFSSLITPSTRVSFQEPKPCNPVIHSAGIENDGRQNCETTMTLSEIM
KGDDEPKNKRAKHKELERQRRQENTSLFKILRYLLPSQYIKGKRSSADHVLEAVNYIKDLQKKI
KEVSEKRDRIKRSITHPSSRGEFSIRSLASSTCSCVGDTNIAVVVRPCLIGLEIVVSCCNRHESCLS
SVLQLLAQEQCFNIVSCISTRLHQGFIHTIASEVEEGIEVYFSELQEKIIKIGTSRVTTR
>AtbHLH056
MSSWLHHSHPGVTSTPASSVSLPPPPNAPREDDIVELLWQSGQVVGTNQTHRQSYDPPPILRGS
GSGRGEENAPLSQPPPHLHQQNLFIQEGEMYSWLHHSYRQNYFCSELLNSTPATHPQSSISLAP
RQTIATRRAENFMNFSWLRGNIFTGGRVDEAGPSFSVVRESMQVGSNTTPPSSSATESCVIPATE
GTASRVSGTLAAHDLGRKGKAVAVEAAGTPSSGVCKAETEPVQIQPATESKLKAREETHGTEEA
RGSTSRKRSRTAEMHNLAERRRREKINEKMKTLQQLIPRCNKSTKVSTLDDAIEYVKSLQSQIQ
GMMSPMMNAGNTQQFMPHMAMDMNRPPPFIPFPGTSFPMPAQMAGVGPSYPAPRYPFPNIQT
FDPSRVRLPSPQPNPVSNQPQFPAYMNPYSQFAGPHQLQQPPPPPFQGQTTSQLSSGQASSSKEP
EDQENQPTA
>AtbHLH057

MSGLMSFGELEDQFGQISDTTMEEKIPFLQMLQCIEHPFTTTEPNQFLQSLLQIQTLESKSCLTLE
TNIKRDPGQTDDPEKDPRTENGAVTVKEKRKRKRTRAPKNKDEVENQRMTHIAVERNRRRQM
NEHLNSLRSLMPPSFLQRGDQASIVGGAIDFIKELEQLLQSLEAEKRKDGTDETPKTASCSSSSS
LACTNSSISSVSTTSENGFTARFGGGDTTEVEATVIQNHVSLKVRCKRGKRQILKAIVSIEELKL
AILHLTISSSFDFVIYSFNLKMEDGCKLGSADEIATAVHQIFEQINGEVMWSNLSRT
>AtbHLH058
MDLSVLDRLKWLQQQQMVSPEFLQILGSDGREELKRVESYLGNNNDELQSFRHFPEFGPDYD
TTDGCISRTSSFHMEPVKNNGHSRAITLQNKRKPEGKTEKREKKKIKAEDETEPSMKGKSNMS
NTETSSEIQKPDYIHVRARRGEATDRHSLAERARREKISKKMKCLQDIVPGCNKVTGKAGMLD
EIINYVQSLQQQVEFLSMKLSVINPELECHIDDLSAKQFQAYFTGPPEGDSKQSIMADFRSFPLH
QQGSLDYSVINSDHTTSLGAKDHTSSSWETHSQCLYNSLRTDSVSNFFSLK
>AtbHLH059
MASNNPHDNLSDQTPSDDFFEQILGLPNFSASSAAGLSGVDGGLGGGAPPMMLQLGSGEEGSH
MGGLGGSGPTGFHNQMFPLGLSLDQGKGPGFLRPEGGHGSGKRFSDDVVDNRCSSMKPVFH
GQPMQQPPPSAPHQPTSIRPRVRARRGQATDPHSIAERLRRERIAERIRALQELVPTVNKTDRAA
MIDEIVDYVKFLRLQVKVLSMSRLGGAGAVAPLVTDMPLSSSVEDETGEGGRTPQPAWEKWSN
DGTERQVAKLMEENVGAAMQLLQSKALCMMPISLAMAIYHSQPPDTSSVVKPENNPPQ
>AtbHLH060
MDLTGGFGARSGGVGPCREPIGLESLHLGDEFRQLVTTLPPENPGGSFTALLELPPTQAVELLHF
TDSSSSQQAAVTGIGGEIPPPLHSFGGTLAFPSNSVLMERAARFSVIATEQQNGNISGETPTSSVP
SNSSANLDRVKTEPAETDSSQRLISDSAIENQIPCPNQNNRNGKRKDFEKKGKSSTKKNKSSEE
NEKLPYVHVRARRGQATDSHSLAERARREKINARMKLLQELVPGCDKGTDFGGKIKIKVCFG
VHLLMISGKKVAIFLWKVSCEDLIDCSFSPPRIQGTALVLDEIINHVQSLQRQVEMLSMRLAAVN
PRIDFNLDTILASENGSLMDGSFNAAPMQLAWPQQAIETEQSFHHRQLQQPPTQQWPFDGLNQ
PVWGREEDQAHGNDNSNLMAVSENVMVASANLHPNQVKMEL
>AtbHLH061
METELTQLRKQESNNLNGVNGGFMAIDQFVPNDWNFDYLCFNNLLQEDDNIDHPSSSSLMNLI
SQPPPLLHQPPQPSSPLYDSPPLSSAFDYPFLEDIIHSSYSPPPLILPASQENTNNYSPLMEESKSFIS
IGETNKKRSNKKLEGQPSKNLMAERRRRKRLNDRLSLLRSIVPKITKMDRTSILGDAIDYMKEL
LDKINKLQEDEQELGSNSHLSTLITNESMVRNSLKFEVDQREVNTHIDICCPTKPGLVVSTVSTL
ETLGLEIEQCVISCFSDFSLQASCFEVGEQRYMVTSEATKQALIRNAGYGGRCL
>AtbHLH062
MENELFMNAGVSHPPVMTSPSSSSAMLKWVSMETQPVDPSLSRNLFWEKSTEQSIFDSALSSL
VSSPTPSNSNFSVGGVGGENVIMRELIGKLGNIGDIYGITASNGNSCYATPMSSPPPGSMMETKT
TTPMAELSGDPGFAERAARFSCFGSRSFNSRTNSPFPINNEPPITTNEKMPRVSSSPVFKPLASHV
PAGESSGELSRKRKTKSKQNSPSAVSSSKEIEEKEDSDPKRCKKSEENGDKTKSIDPYKDYIHVR
ARRGQATDSHSLAERVRREKISERMKLLQDLVPGCNKVTGKALMLDEIINYVQSLQRQVEFLS
MKLSSVNTRLDFNMDALLSKDIFPSSNNLMHHQQVLQLDSSAETLLGDHHNKNLQLNPDISSN
NVINPLETSETRSFISHLPTLAHFTDSISQYSTFSEDDLHSIIHMGFAQNRLQELNQGSSNQVPSH
MKAEL
>AtbHLH063
MNGAIGGDLLLNFPDMSVLERQRAHLKYLNPTFDSPLAGFFADSSMITGGEMDSYLSTAGLNL
PMMYGETTVEGDSRLSISPETTLGTGNFKKRKFDTETKDCNEKKKKMTMNRDDLVEEGEEEK
SKITEQNNGSTKSIKKMKHKAKKEENNFSNDSSKVTKELEKTDYIHVRARRGQATDSHSIAER

VRREKISERMKFLQDLVPGCDKITGKAGMLDEIINYVQSLQRQIEFLSMKLAIVNPRPDFDMDD
IFAKEVASTPMTVVPSPEMVLSGYSHEMVHSGYSSEMVNSGYLHVNPMQQVNTSSDPLSCFN
NGEAPSMWDSHVQNLYGNLGV
>AtbHLH064
MLEGLVSQESLSLNSMDMSVLERLKWVQQQQQQLQQVVSHSSNNSPELLQILQFHGSNNDEL
LESSFSQFQMLGSGFGPNYNMGFGPPHESISRTSSCHMEPVDTMEVLLKTGEETRAVALKNKR
KPEVKTREEQKTEKKIKVEAETESSMKGKSNMGNTEASSDTSKETSKGASENQKLDYIHVRAR
RGQATDRHSLAERARREKISKKMKYLQDIVPGCNKVTGKAGMLDEIINYVQCLQRQVEFLSM
KLAVLNPELELAVEDVSVKQFQAYFTNVVASKQSIMVDVPLFPLDQQGSLDLSAINPNQTTSIE
APSGSWETQSQSLYNTSSLENSCGNYNKISKILLSTKCTHQYVPIRRVWV
>AtbHLH065
MEQVFADWNFEDNFHMSTNKRSIRPEDELVELLWRDGQVVLQSQARREPSVQVQTHKQETLR
KPNNIFLDNQETVQKPNYAALDDQETVSWIQYPPDDVIDPFESEFSSHFFSSIDHLGGPEKPRTIE
ETVKHEAQAMAPPKFRSSVITVGPSHCGSNQSTNIHQATTLPVSMSDRSKNVEERLDTSSGGSS
GCSYGRNNKETVSGTSVTIDRKRKHVMDADQESVSQSDIGLTSTDDQTMGNKSSQRSGSTRRS
RAAEVHNLSERRRRDRINERMKALQELIPHCSRTDKASILDEAIDYLKSLQMQLQVMWMGSG
MAAAAAAAASPMMFPGVQSSPYINQMAMQSQMQLSQFPVMNRSAPQNHPGLVCQNPVQLQ
LQAQNQILSEQLARYMGGIPQMPPAGNQMQTVQQQPADMLGFGSPAGPQSQLSAPATTDSLH
MGKIG
>AtbHLH066
MMNSSLLTPSSSSSSHIQTPSTTFDHEDFLDQIFSSAPWPSVVDDAHPLPSDGFHGHDVDSRNQP
IMMMPLNDGSSVHALYNGFSVAGSLPNFQIPQGSGGGLMNQQGQTQTQTQPQASASTATGGTV
AAPPQSRTKIRARRGQATDPHSIAERLRRERIAERMKALQELVPNGNKTDKASMLDEIIDYVKF
LQLQVKVLSMSRLGGAASVSSQISEAGGSHGNASSAMVGGSQTAGNSNDSVTMTEHQVAKL
MEEDMGSAMQYLQGKGLCLMPISLATAISTATCHSRNPLIPGAVADVGGPSPPNLSGMTIQSTST
KMGSGNGKLNGNGVTERSSSIAVKEAVSVSKA
>AtbHLH067
MERFQGHINPCFFDRKPDVRSLEVQGFAEAQSFAFKEKEEESLQDTVPFLQMLQSEDPSSFFSIK
EPNFLTLLSLQTLKEPWELERYLSLEDSQFHSPVQSETNRFMEGANQAVSSQEIPFSQANMTLPS
STSSPLSAHSRRKRKINHLLPQEMTREKRKRRKTKPSKNNEEIENQRINHIAVERNRRRQMNEH
INSLRALLPPSYIQRGDQASIVGGAINYVKVLEQIIQSLESQKRTQQQSNSEVVENALNHLSGISS
NDLWTTLEDQTCIPKIEATVIQNHVSLKVQCEKKQGQLLKGIISLEKLKLTVLHLNITTSSHSSVS
YSFNLKMEDECDLESADEITAAVHRIFDIPTI
>AtbHLH068
MNRGVLESSPVQQLMAAGNPNWWNVSGGMRPPPPLMGHQQAPLPPHMTPNNNYLRPRMMP
TPFPHFLPSPATSSSSSSSSPSLPNNPNLSSWLESNDLPPESWSLSQLLLGGLMMGEEERLEMMN
HHNHHDEQQHHGFQGKIRLENWEEQVLSHQQASMVAVDIKQEGNINNNNGYVISSPNSPPNK
SCVTTTTTTSLNSNDDNINNNNNMLDFSSNHNGLHLSEGRHTPPDRSSECNSLEIGGSTNKKPR
LQPSPSSQSTLKVRKEKLGGRIAALHQLVSPFGKTDTASVLSEAIGYIRFLQSQIEALSHPYFGTT
ASGNMRHQQHLQGDRSCIFPEDPGQLVNDQCMKRRGASSSSTDNQNASEEPKKDLRSRGLCL
VPISCTLQVGSDNGADYWAPALGSAGFH
>AtbHLH069
MSKSNPSNLKYYNLHPSFKHHKALNLKRHFNNIHFHSHSPPKKKKTEAMNSSSLLTPSSSPSPH
LQSPATFDHDDFLHHIFSSTPWPSSVLDDTPPPTSDCAPVTGFHHHDADSRNQITMIPLSHNHPN

DALFNGFSTGSLPFHLPQGSGGQTQTQSQATASATTGGATAQPQTKPKVRARRGQATDPHSIAE
RLRRERIAERMKSLQELVPNGNKTDKASMLDEIIDYVKFLQLQVKVLSMSRLGGAASASSQISE
DAGGSHENTSSSGEAKMTEHQVAKLMEEDMGSAMQYLQGKGLCLMPISLATTISTATCPSRSP
FVKDTGVPLSPNLSTTIVANGNGSSLVTVKDAPSVSKP
>AtbHLH070
MFVLRVSNQSFKLHQQVQCKDEIFCLDQKVNVRRSLQVQETVEDHQSFALEEEEQQLSTPSLL
QDTTIPFLQMLQQSEDPSPFLSFKDPSFLALLSLQTLEKPWELENYLPHEVPEFHSPIHSETNHY
YHNPSLEGVNEAISNQELPFNPLENARSRRKRKNNNLASLMTREKRKRRRTKPTKNIEEIESQR
MTHIAVERNRRRQMNVHLNSLRSIIPSSYIQRGDQASIVGGAIDFVKILEQQLQSLEAQKRSQQS
DDNKEQIPEDNSLRNISSNKLRASNKEEQSSKLKIEATVIESHVNLKIQCTRKQGQLLRSIILLEK
LRFTVLHLNITSPTNTSVSYSFNLKMEDECNLGSADEITAAIRQIFDS
>AtbHLH071
MTLEALSSNGLLNFLLSETLSPTPFKSLVDLEPLPENDVIISKNTISEISNQEPPPQRQPPATNRGK
KRRRRKPRVCKNEEEAENQRMTHIAVERNRRRQMNQHLSVLRSLMPQPFAHKGDQASIVGGA
IDFIKELEHKLLSLEAQKHHNAKLNQSVTSSTSQDSNGEQENPHQPSSLSLSQFFLHSYDPSQEN
RNGSTSSVKTPMEDLEVTLIETHANIRILSRRRGFRWSTLATTKPPQLSKLVASLQSLSLSILHLS
VTTLDNYAIYSISAKVEESCQLSSVDDIAGAVHHMLSIIEEEPFCCSSMSELPFDFSLNHSNVTHS
L
>AtbHLH072
MSNYGVKELTWENGQLTVHGLGDEVEPTTSNNPIWTQSLNGCETLESVVHQAALQQPSKFQL
QSPNGPNHNYESKDGSCSRKRGYPQEMDRWFAVQEESHRVGHSVTASASGTNMSWASFESGR
SLKTARTGDRDYFRSGSETQDTEGDEQETRGEAGRSNGRRGRAAAIHNESERRRRDRINQRMR
TLQKLLPTASKADKVSILDDVIEHLKQLQAQVQFMSLRANLPQQMMIPQLPPPQSVLSIQHQQ
QQQQQQQQQQQQQQQFQMSLLATMARMGMGGGGNGYGGLVPPPPPPPMMVPPMGNRDCT
NGSSATLSDPYSAFFAQTMNMDLYNKMAAAIYRQQSDQTTKVNIGMPSSSSNHEKRD
>AtbHLH073
MGDSDVGDRLPPPSSSDELSSFLRQILSRTPTAQPSSPPKSTNVSSAETFFPSVSGGAVSSVGYGV
SETGQDKYAFEHKRSGAKQRNSLKRNIDAQFHNLSEKKRRSKINEKMKALQKLIPNSNKTDKA
SMLDEAIEYLKQLQLQVQTLAVMNGLGLNPMRLPQVPPPTHTRINETLEQDLNLETLLAAPHS
LEPAKTSQGMCFSTATLL
>AtbHLH074
MGGESNEGGEMGFKHGDDESGGISRVGITSMPLYAKADPFFSSADWDPVVNAAAAGFSSSHY
HPSMAMDNPGMSCFSHYQPGSVSGFAADMPASLLPFGDCGGGQIGHFLGSDKKGERLIRAGES
SHEDHHQVSDDAVLGASPVGKRRLPEAESQWNKKAVEEFQEDPQRGNDQSQKKHKNDQSKE
TVNKESSQSEEAPKENYIHMRARRGQATNSHSLAERVRREKISERMRLLQELVPGCNKITGKAV
MLDEIINYVQSLQQQVEFLSMKLATVNPEINIDIDRILAKDLLQSRDRNTPTLGLNPFAGFQGNI
PNLSATTNPQYNPLPQTTLESELQNLYQMGFVSNPSTMSSFSPNGRLKPEL
>AtbHLH075
MARFEPYNYNNGHDPFFAHINQNPELINLDLPASTPSSFMLFSNGALVDANHNNSHFFPNLLHG
NTRRKGNKEESGSKRRRKRSEEEEAMNGDETQKPKDVVHVRAKRGQATDSHSLAERVRREKI
NERLKCLQDLVPGCYKAMGMAVMLDVIIDYVRSLQNQIEFLSMKLSAASACYDLNSLDIEPTD
IFQGGNIHSAAEMERILRESVGTQPPNFSSTLPF
>AtbHLH076
MSDKDEFAAKKKDLVNTPVDLYPPENPMLGPSPMMDSFRETLWHDGGFNVHTDADTSFRGN

NNIDIPLEMGWNMAQFPADSGFIERAAKFSFFGCGEMMMNQQQSSLGVPDSTGLFLQDTQIPS
GSKLDNGPLTDASKLVKERSINNVSEDSQSSGGNGHDDAKCGQTSSKGFSSKKRKRIGKDCEE
EEDKKQKDEQSPTSNANKTNSEKQPSDSLKDGYIHMRARRGQATNSHSLAERVRREKISERMK
FLQDLVPGCDKVTGKAVMLDEIINYVQSLQCQIEFLSMKLSAVNPVLDFNLESLLAKDALQSSA
PTFPHNMSMLYPPVSYLSQTGFMQPNISSMSLLSGGLKRQETHGYESDHHNLVHMNHETGTAP
DHEDTTADMKVEP
>AtbHLH077
MNMDKETEQTLNYLPLGQSDPFGNGNEGTIGDFLGRYCNNPQEISPLTLQSFSLNSQISENFPIS
GGIRFPPYPGQFGSDREFGSQPTTQESNKSSLLDPDSVSDRVHTTKSNSRKRKSIPSGNGKESPA
SSSLTASNSKVSGENGGSKGGKRSKQDVAGSSKNGVEKCDSKGDNKDDAKPPEAPKDYIHVR
ARRGQATDSHSLAERARREKISERMTLLQDLVPGCNRITGKAVMLDEIINYVQSLQRQVEFLSM
KLATVNPRMEFNANASLSTEMIQPGESLTQSLYAMACSEQRLPSAYYSLGKNMPRFSDTQFPSN
DGFVHTETPGFWENNDLQSIVQMGFGDILQQQSNNNNNNCSEPTLQMKLEP
>AtbHLH078
MDNELFMNTEFPPPPEMATHFEHQQSSSSAMMLNWALMDPNPHQDSSFLWEKSTEQQQQQSI
FDSALSSLVSSPTPSNSNFSGGGGDGFLIRELIGKLGNIGNNNNNSGEIYGTPMSRSASCYATPMS
SPPPPTNSNSQMMMNRTTPLTEFSADPGFAERAARFSCFGSRSFNGRTNTNLPINNGNNMVNNS
GKLTRVSSTPALKALVSPEVTPGGEFSRKRKSVPKGKSKENPISTASPSPSFSKTAEKNGGKGGS
KSSEEKGGKRRREEEDDEEEEGEGEGNKSNNTKPPEPPKDYIHVRARRGQATDSHSLAERVRR
EKIGERMKLLQDLVPGCNKVTGKALMLDEIINYVQSLQRQVEFLSMKLSSVNDTRLDFNVDAL
VSKDVMIPSSNNRLHEEGLQSKSSSHHHQQQLNIYNNNSQLLPNISSNNMMLQSPMNSLETSTL
ARSFTHLPTLTQFTDSISQYQMFSEEDLQSIVGMGVAENPNNESQHMKIEL
>AtbHLH079
MDPPLVNDSSFSAANPSSYTLSEIWPFPVNDAVRSGLRLAVNSGRVFTRSEHSGNKDVSAAEES
TVTDLTAGWGSRKTRDLNSEDDSSKMVSSSSSGNELKESGDKKRKLCGSESGNGDGSMRPEG
ETSSGGGGSKATEQKNKPEPPKDYIHVRARRGQATDRHSLAERARREKISEKMTALQDIIPGCN
KIIGKALVLDEIINYIQSLQRQVEFLSMKLEVVNSGASTGPTIGVFPSGDLGTLPIDVHRTIYEQQ
EANETRVSQPEWLHMQVDGNFNRTT
>AtbHLH080
MQSTHISGGSSGGGGGGGGEVSRSGLSRIRSAPATWIETLLEEDEEEGLKPNLCLTELLTGNNNS
GGVITSRDDSFEFLSSVEQGLYNHHQGGGFHRQNSSPADFLSGSGSGTDGYFSNFGIPANYDYL
STNVDISPTKRSRDMETQFSSQLKEEQMSGGISGMMDMNMDKIFEDSVPCRVRAKRGCATHPR
SIAERVRRTRISDRIRRLQELVPNMDKQTNTADMLEEAVEYVKALQSQIQELTEQQKRCKCKPK
EEQ
>AtbHLH081
MQPTSVGSSGGGDDGGGRGGGGGLSRSGLSRIRSAPATWLEALLEEDEEESLKPNLGLTDLLTG
NSNDLPTSRGSFEFPIPVEQGLYQQGGFHRQNSTPADFLSGSDGFIQSFGIQANYDYLSGNIDVS
PGSKRSREMEALFSSPEFTSQMKGEQSSGQVPTGVSSMSDMNMENLMEDSVAFRVRAKRGCA
THPRSIAERVRRTRISDRIRKLQELVPNMDKQTNTADMLEEAVEYVKVLQRQIQELTEEQKRCT
CIPKEEQ
>AtbHLH082
MENGNGEGKGEFINQNNDFFLDSMSMLSSLPPCWDPSLPPPPPPPQSLFHALAVDAPFPDQFHH
PQESGGPTMGSQEGLQPQGTVSTTSAPVVRQKPRVRARRGQATDPHSIAERLRRERIAERMKS
LQELVPNTNKTDKASMLDEIIEYVRFLQLQVKVLSMSRLGGAGSVGPRLNGLSAEAGGRLNAL

TAPCNGLNGNGNATGSSNESLRSTEQRVAKLMEEDMGSAMQYLQGKGLCLMPISLATAISSST
THSRGSLFNPISSAVAAEDSNVTATAVAAPEASSTMDDVSASKA
>AtbHLH083
MALVNDHPNETNYLSKQNSSSSEDLSSPGLDQPDAAYAGGGGGGGSASSSSTMNSDHQQHQG
FVFYPSGEDHHNSLMDFNGSSFLNFDHHESFPPPAISCGGSSGGGGFSFLEGNNMSYGFTNWN
HQHHMDIISPRSTETPQGQKDWLYSDSTVVTTGSRNESLSPKSAGNKRSHTGESTQPSKKLSSG
VTGKTKPKPTTSPKDPQSLAAKNRRERISERLKILQELVPNGTKVDLVTMLEKAISYVKFLQVQ
VKVLATDEFWPAQGGKAPDISQVKDAIDAILSSSQRDRNSNLITN
>AtbHLH084
MEAMGEWSTGLGGIYTEEADFMNQLLASYEQPCGGSSSETTATLTAYHHQGSQWNGGFCFSQ
ESSSYSGYCAAMPRQEEDNNGMEDATINTNLYLVGEETSECDATEYSGKSLLPLETVAENHDHS
MLQPENSLTTTTDEKMFNQCESSKKRTRATTTDKNKRANKARRSQKCVEMSGENENSGEEEY
TEKAAGKRKTKPLKPQKTCCSDDESNGGDTFLSKEDGEDSKALNLNGKTRASRGAATDPQSLY
ARLKQLNKVHCMMVQKRRERINERLRILQHLVPNGTKVDISTMLEEAVQYVKFLQLQIKLLSS
DDLWMYAPIAYNGMDIGLDLKLNALTR
>AtbHLH085
MEAMGEWSNNLGGMYTYATEEADFMNQLLASYDHPGTGSSSGAAASGDHQGLYWNLGSHH
NHLSLVSEAGSFCFSQESSSYSAGNSGYYTVVPPTVEENQNETMDFGMEDVTINTNSYLVGEET
SECDVEKYSSGKTLMPLETVVENHDDEESLLQSEISVTTTKSLTGSKKRSRATSTDKNKRARVN
KRAQKNVEMSGDNNEGEEEEGETKLKKRKNGAMMSRQNSSTTFCTEEESNCADQDGGGEDS
SSKEDDPSKALNLNGKTRASRGAATDPQSLYARKRRERINERLRILQNLVPNGTKVDISTMLEE
AVHYVKFLQLQIKLLSSDDLWMYAPIAFNGMDIGLSSPR
>AtbHLH086
MSLINEHCNERNYISTPNSSEDLSSPQNCGLDEGASASSSSTINSDHQNNQGFVFYPSGETIEDH
NSLMDFNASSFFTFDNHRSLISPVTNGGAFPVVDGNMSYSYDGWSHHQVDSISPRVIKTPNSFE
TTSSFGLTSNSMSKPATNHGNGDWLYSGSTIVNIGSRHESTSPKLAGNKRPFTGENTQLSKKPSS
GTNGKIKPKATTSPKDPQSLAAKNRRERISERLKVLQELVPNGTKVDLVTMLEKAIGYVKFLQ
VQVKVLAADEFWPAQGGKAPDISQVKEAIDAILSSSQRDSNSTRETSIAE
>AtbHLH087
MEGLESVYAQAMYGMTRESKIMEHQGSDLIWGGNELMARELCSSSSYHHQLINPNLSSCFMS
DLGVLGEIQQQQHVGNRASSIDPSSLDCLLSATSNSNNTSTEDDEGISVLFSDCQTLWSFGGVSS
AESENREITTETTTTIKPKPLKRNRGGDGGTTETTTTTTKPKSLKRNRGDETGSHFSLVHPQDDS
EKGGFKLIYDENQSKSKKPRTEKERGGSSNISFQHSTCLSDNVEPDAEAIAQMKEMIYRAAAFR
PVNFGLEIVEKPKRKNVKISTDPQTVAARQRRERISEKIRVLQTLVPGGTKMDTASMLDEAANY
LKFLRAQVKALENLRPKLDQTNLSFSSAPTSFPLFHPSFLPLQNPNQIHHPEC
>AtbHLH088
MDSDIMNMMMHQMEKLPEFCNPNSSFFSPDHNNTYPFLFNSTHYQSDHSMTNEPGFRYGSGL
LTNPSSISPNTAYSSVFLDKRNNSNNNNNGTNMAAMREMIFRIAVMQPIHIDPEAVKPPKRRNV
RISKDPQSVAARHRRERISERIRILQRLVPGGTKMDTASMLDEAIHYVKFLKKQVQSLEEQAVV
TGGGGGGGGGRVLIGGGGMTAASGGGGGGGGVVMKGCGTVGTHQMVGNAQILR
>AtbHLH089
MGGGGMFEEIGCFDPNAPAEMTAESSFSPSEPPPTITVIGSNSNSNCSLEDLSAFHLSPQDSSLPA
SASAYAHQLHINATPNCDHQFQSSMHQTLQDPSYAQQSNHWDNGYQDFVNLGPNHTTPDLLS
LLQLPRSSLPPFANPSIQDIIMTTSSSVAAYDPLFHLNFPLQPPNGSFMGVDQDQTETNQGVNLM

YDEENNNLDDGLNRKGRGSKKRKIFPTERERRVHFKDRFGDLKNLIPNPTKNDRASIVGEAID
YIKELLRTIDEFKLLVEKKRVKQRNREGDDVVDENFKAQSEVVEQCLINKKNNALRCSWLKRK
SKFTDVDVRIIDDEVTIKIVQKKKINCLLFVSKVVDQLELDLHHVAGAQIGEHHSFLFNAKISEG
SSVYASAIADRVMEVLKKQYMEALSANNGYHCYSSD
>AtbHLH090
MMMMRGGERVKEFLRPFVDSRTWDLCVIWKLGDDPSRFIEWVGCCCSGCYIDKNIKLENSEE
GGTGRKKKASFCRDDHNKHRIRTLACEALSRFPLFMPLYPGIHGEVVMSKSPKWLVNSGSKME
MFSTRVLVPVSDGLVELFAFDMRPFDESMVHLIMSRCTTFFEPFPEQRLQFRIIPRAEESMSSGV
NLSVEGGGSSSVSNPSSETQNLFGNYPNASCVEILREEQTPCLIMNKEKDVVVQNANDSKANK
KLLPTENFKSKNLHSERKRRERINQAMYGLRAVVPKITKLNKIGIFSDAVDYINELLVEKQKLE
DELKGINEMECKEIAAEEQSAIADPEAERVSSKSNKRVKKNEVKIEVHETGERDFLIRVVQEHK
QDGFKRLIEAVDLCELEIIDVNFTRLDLTVMTVLNVKANKDGIACGILRDLLLKMMITSI
>AtbHLH091
MYEESSCFDPNSMVDNNGGFCAAETTFTVSHQFQPPLGSTTNSFDDDLKLPTMDEFSVFPSVIS
LPNSETQNQNISNNNHLINQMIQESNWGVSEDNSNFFMNTSHPNTTTTPIPDLLSLLHLPRCSMS
LPSSDIMAGSCFTYDPLFHLNLPPQPPLIPSNDYSGYLLGIDTNTTTQRDESNVGDENNNAQFDS
GIIEFSKEIRRKGRGKRKNKPFTTERERRCHLNERYEALKLLIPSPSKGDRASILQDGIDYINELR
RRVSELKYLVERKRCGGRHKNNEVDDNNNNKNLDDHGNEDDDDDDENMEKKPESDVIDQCS
SNNSLRCSWLQRKSKVTEVDVRIVDDEVTIKVVQKKKINCLLLVSKVLDQLQLDLHHVAGGQI
GEHYSFLFNTKIYEGSTIYASAIANRVIEVVDKHYMASLPNSNY
>AtbHLH092
MDNFFLGLSCQEENNFWDLIVADISGDRSVSVPIRSAFRSYMKDTELRMMSPKISSSKVNVKKR
MVNLLRKNWEEKKNTVAPEKERSRRHMLKERTRREKQKQSYLALHSLLPFATKNDKNSIVEK
AVDEIAKLQRLKKELVRRIKVIEEKSAKDGHDEMSETKVRVNLKEPLSGLDSMLEALHYLKSM
GTKLKTVHANFSPQEFSATMTIETQIRGEEVEKRVERRLQETEWKLLFLPEASFYKDY
>AtbHLH093
MELSTQMNVFEELLVPTKQETTDNNINNLSFNGGFDHHHHQFFPNGYNIDYLCFNNEEEDENT
LLYPSSFMDLISQPPPLLLHQPPPLQPLSPPLSSSATAGATFDYPFLEALQEIIDSSSSSPPLILQNGQ
EENFNNPMSYPSPLMESDQSKSFSVGYCGGETNKKKSKKLEGQPSKNLMAERRRRKRLNDRL
SMLRSIVPKISKMDRTSILGDAIDYMKELLDKINKLQDEEQELGNSNNSHHSKLFGDLKDLNAN
EPLVRNSPKFEIDRRDEDTRVDICCSPKPGLLLSTVNTLETLGLEIEQCVISCFSDFSLQASCSEGA
EQRDFITSEDIKQALFRNAGYGGSCL
>AtbHLH094
MPLEAVVYPQDPFGYLSNCKDFMFHDLYSQEEFVAQDTKNNIDKLGHEQSFVEQGKEDDHQW
RDYHQYPLLIPSLGEELGLTAIDVESHPPPQHRRKRRRTRNCKNKEEIENQRMTHIAVERNRRK
QMNEYLAVLRSLMPSSYAQRGDQASIVGGAINYVKELEHILQSMEPKRTRTHDPKGDKTSTSSL
VGPFTDFFSFPQYSTKSSSDVPESSSSPAEIEVTVAESHANIKIMTKKKPRQLLKLITSLQSLRLTL
LHLNVTTLHNSILYSISVRVRTFSTSHHYFVCFWGNHEIKVSNDCVCGAYGFTLLIYFFVSYCW
GEILD
>AtbHLH095
MTNAQELGQEGFMWGISNSDDSGGGCKRIEKEPLPSHPSHPSPEIQTTTVKKGKKRTKRNDKN
HEEESPDHEIHIWTERERRKKMRDMFSKLHALLPQLPPKADKSTIVDEAVSSIKSLEQTLQKLE
MQKLEKLQYSSASTNTTPTTTFAYAPSSSSSPTALLTPISNHPIDATATDSYPRAAFLADQVSSSSA
AAANLPYPCNDPIVNFDTWSSRNVVLTICGNEAFFNLCVPKHKPGVFTSVCYLFEKYNMEVLF

ANVSSNVFWSTYVIQAQVNPSCENQLLGNGLGVVDVFKQVSQELVLYFSSL
>AtbHLH096
MALEAVVYPQDPFSYISCKDFPFYDLYFQEEEDQDPQDTKNNIKLGQGQGHGFASNNYNGRTG
DYSDDYNYNEEDLQWPRDLPYGSAVDTESQPPPSDVAAGGGRRKRRRTRSSKNKEEIENQRM
THIAVERNRRKQMNEYLAVLRSLMPPYYAQRGDQASIVGGAINYLKELEHHLQSMEPPVKTAT
EDTGAGHDQTKTTSASSSGPFSDFFAFPQYSNRPTSAAAAEGMAEIEVTMVESHASLKILAKKR
PRQLLKLVSSIQSLRLTLLHLNVTTRDDSVLYSISVKVEEGSQLNTVEDIAAAVNQILRRIEEESS
FS
>AtbHLH097
MDKDYSAPNFLGESSGGNDDNSSGMIDYMFNRNLQQQQKQSMPQQQQHQLSPSGFGATPFD
KMNFSDVMQFADFGSKLALNQTRNQDDQETGIDPVYFLKFPVLNDKIEDHNQTQHLMPSHQT
SQEGGECGGNIGNVFLEEKEDQDDDNDNNSVQLRFIGGEEEDRENKNVTKKEVKSKRKRART
SKTSEEVESQRMTHIAVERNRRKQMNEHLRVLRSLMPGSYVQRGDQASIIGGAIEFVRELEQLL
QCLESQKRRRILGETGRDMTTTTTSSSSPITTVANQAQPLIITGNVTELEGGGGLREETAENKSC
LADVEVKLLGFDAMIKILSRRRPGQLIKTIAALEDLHLSILHTNITTMEQTVLYSFNVKITSETRF
TAEDIASSIQQIFSFIHANTNISGSSNLGNIVFT
>AtbHLH098
MQEIIPDFLEECEFVDTSLAGDDLFAILESLEGAGEISPTAASTPKDGTTSSKELVKDQDYENSSP
KRKKQRLETRKEEDEEEEDGDGEAEEDNKQDGQQKMSHVTVERNRRKQMNEHLTVLRSLMP
CFYVKRGDQASIIGGVVEYISELQQVLQSLEAKKQRKTYAEVLSPRVVPSPRPSPPVLSPRKPPL
SPRINHHQIHHHLLLPPISPRTPQPTSPYRAIPPQLPLIPQPPLRSYSSLASCSSLGDPPPYSPASSSS
SPSVSSNHESSVINELVANSKSALADVEVKFSGANVLLKTVSHKIPGQVMKIIAALEDLALEILQ
VNINTVDETMLNSFTIKIGIECQLSAEELAQQIQQTFC
>AtbHLH099
MMFQQDYPHGFSLVETSLSYEMLDYFQNIVVSNSEDVASQQNSISSSSYSSATLSCSITEQKSHL
TEKLSPLRERYGCGDFLSRKRRRRSEKTIVDKENQRMNHIAVERNRRKQMNHFLSILKSMMPL
SYSQPNDQASIIEGTISYLKKLEQRLQSLEAQLKATKLNQSPNIFSDFFMFPQYSTATATATATASS
SSSSHHHHKRLEVVADVEVTMVERHANIKVLTKTQPRLLFKIINEFNSLGLSTLHLNLTTSKDM
SLFTFSVKVEADCQLTPSGNEVANTVHEVVRRVHKER
>AtbHLH100
MCALVPPLYPNFGWPCGDHSFYETDDVSNTFLDFPLPDLTVTHENVSSENNRTLLDNPVVMKK
LNHNASERERRKKINTMFSSLRSCLPPTNQTKKLSVSATVSQALKYIPELQEQVKKLMKKKEEL
SFQISGQRDLVYTDQNSKSEEGVTSYASTVSSTRLSETEVMVQISSLQTEKCSFGNVLSGVEED
GLVLVGASSSRSHGERLFYSMHLQIKNGQVNSEELGDRLLYLYEKCGHSFT
>AtbHLH101
MCTLTPMFPSKQQEWYSASTMEYPWLQSQVHSFSPTLHFPSFLHPLDDSKSHNINLHHMSLSH
SNNTNSNNNNYQEEDRGAVVLEKKLNHNASERDRRRKLNALYSSLRALLPLSDQKRKLSIPMT
VARVVKYIPEQKQELQRLSRRKEELLKRISRKTHQEQLRNKAMMDSIDSSSSQRIAANWLTDTE
IAVQIATSKWTSVSDMLLRLEENGLNVISVSSSVSSTARIFYTLHLQMRGDCKVRLEELINGMLL
GLRQS
>AtbHLH102
MRTGKGNQEEEDYGEEDFNSKREGPSSNTTVHSNRDSKENDKASAIRSKHSVTEQRRRSKINE
RFQILRELIPNSEQKRDTASFLLEVIDYVQYLQEKVQKYEGSYPGWSQEPTKLTPWRNNHWRV
QSLGNHPVAINNGSGPGIPFPGKFEDNTVTSTPAIIAEPQIPIESDKARAITGISIESQPELDDKGLP

PLQPILPMVQGEQANECPATSDGLGQSNDLVIEGGTISISSAYSHELLSSLTQALQNAGIDLSQAK
LSVQIDLGKRANQGLTHEEPSSKNPLSYDTQGRDSSVEEESEHSHKRMKTL
>AtbHLH103
MTEEFDTTGVCTGTWWSSSNGMFSGCSLPRSAEIVVDFGEIEWQNIDTLDAKTYNENYLSTST
FLGNANLDTTSQIYVSSPSNIHEEERYNQINSFLEGLFDSSEQLLVPNCPKPELFESFHFFDDVFP
NESRMISVFDHQKPKEDMQACKSLTTCKRASEKSGELEDIESSQPLKRPRLETPSHFPSFKVRKE
KLGDRITALQQLVSPFGKTDTASVLHDAIDYIKFLQEQITEKVSTSPHLNSIGSGEQKQWSDKSS
NNTHNQNCSPRQDLRSRGLCLMPISSTFSTPPQHLDTSSLWN
>AtbHLH104
MYPSLDDDFVSDLFCFDQSNGAELDDYTQFGVNLQTDQEDTFPDFVSYGVNLQQEPDEVFSIG
ASQLDLSSYNGVLSLEPEQVGQQDCEVVQEEEVEINSGSSGGAVKEEQEHLDDDCSRKRARTG
SCSRGGGTKACRERLRREKLNERFMDLSSVLEPGRTPKTDKPAILDDAIRILNQLRDEALKLEE
TNQKLLEEIKSLKAEKNELREEKLVLKADKEKTEQQLKSMTAPSSGFIPHIPAAFNHNKMAVYP
SYGYMPMWHYMPQSVRDTSRDQELRPPAA
>AtbHLH105
MVSPENANWICDLIDADYGSFTIQGPGFSWPVQQPIGVSSNSSAGVDGSAGNSEASKEPGSKK
RGRCESSSATSSKACREKQRRDRLNDKFMELGAILEPGNPPKTDKAAILVDAVRMVTQLRGEA
QKLKDSNSSLQDKIKELKTEKNELRDEKQRLKTEKEKLEQQLKAMNAPQPSFFPAPPMMPTAF
ASAQGQAPGNKMVPIISYPGVAMWQFMPPASVDTSQDHVLRPPVA
>AtbHLH106
MQPETSDQMLYSFLAGNEVGGGGYCVSGDYMTTMQSLCGSSSSTSSYYPLAISGIGETMAQD
RALAALRNHKEAERRRRERINSHLNKLRNVLSCNSKTDKATLLAKVVQRVRELKQQTLETSDS
DQTLLPSETDEISVLHFGDYSNDGHIIFKASLCCEDRSDLLPDLMEILKSLNMKTLRAEMVTIGG
RTRSVLVVAADKEMHGVESVHFLQNALKSLLERSSKSLMERSSGGGGGERSKRRRALDHIIMV
>AtbHLH107
MQPEVSDQIFYAFLTGGLCASSTSTTVTSSSDPFATVYEDKALASLRNHKEAERKRRARINSHL
NKLRKLLSCNSKTDKSTLLAKVVQRVKELKQQTLEITDETIPSETDEISVLNIEDCSRGDDRRIIF
KVSFCCEDRPELLKDLMETLKSLQMETLFADMTTVGGRTRNVLVVAADKEHHGVQSVNFLQN
ALKSLLERSSKSVMVGHGGGGGEERLKRRRALDHIIMV
>AtbHLH108
MNKDEVFLRQWFEILYSLTNPEANSDLRRINNEKGVEKVGQKRSAESRREGKKKRVKTQCVIK
SSDKSDHDTLLKKKRRERIRRQLETLKEITPNCPQSDINAILDCVIEYTNNLRLAHYKGSQGICD
DWRLFTEAGAVLYYIDT
>AtbHLH109
MERNNRNEGTHEEEQCSLSDIIYSFCSENHSELNPLQEIFGVTKNNDHEKHDEEPDEESYRMAK
RQRSMEYRMMMEKKRRKEIKDKVDILQGLMPNHCTKPDLASKLENIIEYIKSLKYQVDVMSM
AYTTTPVYTPPFYAAAQAPCMSPWGYYTPGVPMMPQQNMTYIPQYPQVYGTVPPNQTQP
>AtbHLH110
MDSANLHQLQDQLQLVGSSSSSSSLDNNSDPSCYGASSAHQWSPGGISLNSVSLSHNYNNEML
NTRAHNNNNNNNNTSECMSLSSIHNHSLIQQQDFPLQWPHDQSSYQHHEGLLKIKEELSSSTISD
HQEGISKFTDMLNSPVITNYLKINEHKDYTEKLLLKSMSSGFPINGDYGSSLPSSSSSSSPSSQSH
RGNFSQIYPSVNISSLSESRKMSMDDMSNISRPFDINMQVFDGRLFEGNVLVPPFNAQEISSLGM
SRGSLPSFGLPFHHHLQQTLPHLSSSPTHQMEMFSNEPQTSEGKRHNFLMATKAGENASKKPR
VESRSSCPPFKVRKEKLGDRIAALQQLVSPFGKTDTASVLMEAIGYIKFLQSQIETLSVPYMRAS

RNRPGKASQLVSQSQEGDEEETRDLRSRGLCLVPLSCMTYVTGDGGDGGGGVGTGFWPTPPG
FGGGT
>AtbHLH111
MLREECTPSSSWWEDVQHHHNDHANSISSTSFYHKSSNNNSHANASCEEDNLSVSTVRASNRL
DLTAESSNHHSLSASNQPASSSDELLRDHVVSSHNHLWSLAFLPGRSLGDQMMDHHHHIASRN
SSTTSELPSFEPACHNGNGNGWIYDPNQVRYDQSSDQRLSKLTDLVGKHWSIAPPNNPDMNHN
LHHHFDHDHSQNDDISMYRQALEVKNEEDLCYNNGSSGGGSLFHDPIESSRSFLDIRLSRPLTDI
NPSFKPCFKALNVSEFNKKEHQTASLLFLQAAVRLGTTNAGKKKRCEEISDEVSKKAKCSEGST
LSPEKELPKAKLRDKITTLQQIVSPFGKTDTASVLQEAITYINFYQEQVKLLSTPYMKNSSMKDP
WGGWDREDHNKRGPKHLDLRSRGLCLVPISYTPIAYRDNSATDYWNPTYRGSLYR
>AtbHLH112
MAEEFKATASICGGGGGAWWNSPRSVMSPSDHFLSPCFGAAITSNDFSSQENHLKSRMTCTDN
NNIVFGQREADSDSGGSTVTMDSTLQMMGLGFSSNCSSDWNQTILQEDLNSSFIRSSQDQDHG
QGFLSTTTSPYILNPACSSSPSTSSSSSLIRTFYDPEPSPYNFVSTTSGSINDPQLSWANKTNPHHQ
VAYGLINSFSNNANSRPFWNSSSTTNLNNTTPSNFVTTPQIISTRLEDKTKNLKTRAQSESLKRA
KDNESAAKKPRVTTPSPLPTFKVRKENLRDQITSLQQLVSPFGKTDTASVLQEAIEYIKFLHDQV
TVLSTPYMKQGASNQQQQQISGKSKSQDENENHELRGHGLCLVPISSTFPVANETTADFWTPTF
GGNNFR
>AtbHLH113
MGDTAEDQDDRAMMEAEGVTSFSELLMFSDGVLSSSSDHQPEGNVGDGGEDSLGFVFSGKTG
SRMLCFSGGYQNDDESLFLEPSVPTSGVSDLDPSCIKIDCRNSNDACTVDKSTKSSTKKRTGTG
NGQESDQNRKPGKKGKRNQEKSSVGIAKVRKERLGERIAALQQLVSPYGKTDAASVLHEAMG
YIKFLQDQIQVLCSPYLINHSLDGGVVTGDVMAAMKAKDLRSRGLCLVPVSSTVHVENSNGA
DFWSPATMGHTTSPSLPQGF
>AtbHLH114
MSHATSTLNSKVNYTIKNMTEEFEIAGISTGAWWSSPTNTAAVFSGYSLPCSTEISPDVTNFGW
QNFDNKINDHNDGCMNMHNSFFEGLLIDPNDQLLPDPWSKSTIPNAKSELLENFPFLDNMFLV
DSEAESLLDHEIRNHKSSKEQITQDYKNLTSKRSEELEENSDEYSPRLLKRPRLETLSPLPSFKVR
KEKLGDRITALQQLVSPFGKTDTASVLNEAVEYIKFLQEQVTVLSNPEQNTIGSVQQQQCSNKK
SINTQGEVEEDECSPRRYVDLSSRGLCLMPISASYPVAAAAASAAEMNVHLVSGIFHSL
>AtbHLH115
MVSPENTNWLSDYPLIEGAFSDQNPTFPWQIDGSATVRIGSYIWRSCYGCVITQLVFRCSCCDR
DIGLLHKSILCFFFCLRFAYVPLFPKEVDESRSLPIAKGSVEVDGFLCDADVIKEPSSRKRIKTESC
TGSNSKACREKQRRDRLNDKFTELSSVLEPGRTPKTDKVAIINDAIRMVNQARDEAQKLKDLN
SSLQEKIKELKDEKNELRDEKQKLKVEKERIDQQLKAIKTQPQPQPCFLPNPQTLSQAQAPGSK
LVPFTTYPGFAMWQFMPPAAVDTSQDHVLRPPVA
>AtbHLH116
MGLDGNNGGGVWLNGGGGEREENEEGSWGRNQEDGSSQFKPMLEGDWFSSNQPHPQDLQM
LQNQPDFRYFGGFPFNPNDNLLLQHSIDSSSSCSPSQAFSLDPSQQNQFLSTNNNKGCLLNVPSS
ANPFDNAFEFGSESGFLNQIHAPISMGFGSLTQLGNRDLSSVPDFLSARSLLAPESNNNNTMLC
GGFTAPLELEGFGSPANGGFVGNRAKVLKPLEVLASSGAQPTLFQKRAAMRQSSGSKMGNSES
SGMRRFSDDGDMDETGIEVSGLNYESDEINESGKAAESVQIGGGGKGKKKGMPAKNLMAERR
RRKKLNDRLYMLRSVVPKISKMDRASILGDAIDYLKELLQRINDLHNELESTPPGSLPPTSSSFH
PLTPTPQTLSCRVKEELCPSSLPSPKGQQARVEVRLREGRAVNIHMFCGRRPGLLLATMKALDN

LGLDVQQAVISCFNGFALDVFRAEQCQEGQEILPDQIKAVLFDTAGYAGMI
>AtbHLH117
METPAYDFDSLTDLPPLPPSDFTPSNAFTFPDHNLDFSFLDSTLSLLNRHHLSESTRLEQIFYDST
HTQLFHNDDTTTTTTPFLHLPDLKSIDAVEEPTTMKLFPSLSPPLPAAKRQKLNSTSSSTTSGSPT
ASNDGGIITKRRKISDKIRSLEKLMPWERKMNLAMTLEESHKYIKFLQSQIASLRWMPLESVYN
TAGEVGETDLLKSLTRQQILQVLANSPGSRNVLSSRGVCVFSYEQLLSLKTMSRNL
>AtbHLH118
MNNFQEKKRRRSKTPRVCNNEENMEKLVHKEIEKRRRQEMASLYASLRSLLPLEFIQGKRSTSD
QVKGAVNYIDYLQRNIKDINSKRDDLVLLSGRSFRSSNEQEWNEISNHVVIRPCLVGIEIVLSILQ
TPFSSVLQVLREHGLYVLGYICSSVNDRLIHTLQAEVQYLELTNFTAIEALLTLLRI
>AtbHLH119
MGEDDIVELLWNGQVVRTSQPQRPSSGKPSPTPPILRGSGSGSGEENAPLPLPLLQPPRPLHHQN
LFIREEEMSSWLHYSYTGVTSTPATHPQSSVSLPPPPPIAPSEDDVVELLWKSGQVVQSIQTQRPI
PPPIFRGSGSGGGEETVLPLPPLHPSHQNIFIQEDEMASWLYHPLRQDYFSSGVASTSATRPQSSA
SLAPTPPPPSVPYGQIPVERRTENFMNFLRLRGNIFSGGRVEAGPVVIESTQIGSSATPSSSAAESC
VIPATHGTESRAAAITGVSRTFAVPGLGRRGKEVATETAGTSYSGVNKAETERVQIQPERETKITE
DKKREETIAEIQGTEEAHGSTSRKRSRAADMHNLSERRRRERINERMKTLQELLPRCRKTDKV
SMLEDVIEYVKSLQLQIQMMSMGHGMMPPMMHEGNTQQFMPHMAMGMKGMNRPPPFVPFP
GKTFPRPGHMAGVGPSYPALRYPFPDTQASDLSRVHVPSLHSNPVPNQPRFPAYINPYSQFVGL
HQMQQPPLPLQGQPTSQPSFSHASTSK
>AtbHLH120
MKTTPLPRLHYLVSLLCFFLSSKIKEDRPNYVRAVSPINLTSSLEKTREKKKRLLLRSTISKPQPM
NPSNNPKKTRHQSHMPQERDETKKEKKLLHRNIERQRRQEMAILFASLRSQLPLKYIKGKRAM
SDHVNGAVSFIKDTQTRIKDLSARRDELKREIGDPTSLTGSGSGSGSSRSEPASVMVQPCVSGFE
VVVSSLASGLEAWPLSRVLEVLHGQGLEVISSLTARVNERLMYTIQVEVNSFDCFDLAWLQQK
LIEQLVLSTTRH
>AtbHLH121
MGIRENGIMLVSRERERARRLENRESIFAEPPCLLLAHRISPSPSILPAEEEVMDVSARKSQKAGR
EKLRREKLNEHFVELGNVLDPERPKNDKATILTDTVQLLKELTSEVNKLKSEYTALTDESRELT
QEKNDLREEKTSLKSDIENLNLQYQQRLRSMSPWGAAMDHTVMMAPPPSFPYPMPIAMPPGSI
PMHPSMPSYTYFGNQNPSMIPAPCPTYMPYMPPNTVVEQQSVHIPQNPGNRSREPRAKVSRES
RSEKAEDSNEVATQLELKTPGSTSDKDTLQRPEKTKRCKRNNNNNSIEESSHSSKCSSSPSVRD
HSSSSSVAGGQKPDDAK
>AtbHLH122
MESEFQQHHFLLHDHQHQRPRNSGLIRYQSAPSSYFSSFGESIEEFLDRPTSPETERILSGFLQTT
DTSDNVDSFLHHTFNSDGTEKKPPEVKTEDEDAEIPVTATATAMEVVVSGDGEISVNPEVSIGY
VASVSRNKRPREKDDRTPVNNLARHNSSPAGLFSSIDVETAYAAVMKSMGGFGGSNVMSTSNT
EASSLTPRSKLLPPTSRAMSPISEVDVKPGFSSRLPPRTLSGGFNRSFGNEGSASSKLTALARTQS
GGLDQYKTKDEDSASRRPPLAHHMSLPKSLSDIEQLLSDSIPCKIRAKRGCATHPRSIAERVRRT
KISERMRKLQDLVPNMDTQTNTADMLDLAVQYIKDLQEQVKALEESRARCRCSSA
>AtbHLH123
MGDHHDFINSGSWWKVSSSSSPSSSSSMRASSIESGGSAVFHDKLHHHSLATDHHLQMIGLGLS
SQSPVDQWNQSLLRGDSKAETSFGVMLQENLNLDATSNANANTTSSTSSYQLQESDSSHHHQ
ALWRDPQSDFKPQILTSGGNRGFFLDHQFSPHGSSSTDSSTVTCQGFAVDNSSNAMYAATTTTP

NSSSGMFHHQQAGGFGSSDQQPSRNHQQSSLGYSQFGSSTGNYDQMASALPSTWFLRSSPPPK
PHSPLRFSNNATFWNPAAAGNAGAPPPHDASSNFFPALQPPQIHPQSFDEQPKNISEIRDSSSNEV
KRGGNDHQPAAKRAKSEAASPSPAFKRKEKMGDRIAALQQLVSPFGKTDAASVLSEAIEYIKFL
HQQVSALSNPYMKSGASLQHQQSDHSTELEVSEEPDLRSRGLCLVPVSSTFPVTHDTTVDFWT
PTFGGTFR
>AtbHLH124
MEAKPLASSSSEPNMISPSSNIKPKLKDEDYMELVCENGQILAKIRRPKNNGSFQKQRRQSLLD
LYETEYSEGFKKNIKILGDTQVVPVSQSKPQQDKETNEQMNNNKKKLKSSKIEFERNVSKSNK
CVESSTLIDVSAKGPKNVEVTTAPPDEQSAAVGRSTELYFASSSKFSRGTSRDLSCCSLKRKYGD
IEEEESTYLSNNSDDESDDAKTQVHARTRKPVTKRKRSTEVHKLYERKRRDEFNKKMRALQD
LLPNCYKDDKASLLDEAIKYMRTLQLQVQMMSMGNGLIRPPTMLPMGHYSPMGLGMHMGA
AATPTSIPQFLPMNVQATGFPGMNNAPPQMLSFLNHPSGLIPNTPIFSPLENCSQPFVVPSCVSQT
QATSFTQFPKSASASNLEDAMQYRGSNGFSYYRSPN
>AtbHLH125
MDCVPSLFMPDSTYEDGLLFSDSFLLSPFISYQNNDVFHSITNKIGGSNKKRSLCDITYGANEAN
KNDDDRESKKMKHRDIERQRRQEVSSLFKRLRTLLPFQYIQGKRSTSDHIVQAVNYIKDLQIKI
KELNEKRNRVKKVISATTTTHSAIEECTSSLSSSAASTLSSSCSCVGDKHITVVVTPCLVGVEIIIS
CCLGRNKSCLSSVLQMLAQEQRFSVVSCLSARRQQRFMHTIVSQVEDGKQINILELKDKIMTM
>AtbHLH126
MDPYKNLNPKGYQRQRPFSSAGESGGSGGSGTAHETDDNKKKKKLLHRDIERQRRQEMATLF
ATLRTHLPLKYIKGKRAVSDHVNGAVNFIKDTEARIKELSARRDELSRETGQGYKSNPDPGKTG
SDVGKSEPATVMVQPHVSGLEVVVSSNSSGPEALPLSKVLETIQEKGLEVMSSFTTRVNDRLM
HTIQVEVNSFGCIDLLWLQQKLVEDLILSTGY
>AtbHLH127
MMIISSQILLLFGFKLFFETRGEDDIVELLCKIGQTQIPSSDPLPILRGSGSGGREENTPLPPPLPHQ
NLFIQEDEMSSWPHHPLRQDYLCSELYASTPAPHPQSSVSLAPPPPKPPSSAPYGQIIAPRSAPRIQ
GTEEARGSTSRKRSRAAEMHNLAERRRREKINERMKTLQQLIPRCNKSTKVSMLEDVIEYVKS
LEMQINQFMPHMAMGMNQPPAYIPFPSQAHMAGVGPSYPPPRYPFPNIQTFDPSRVWLQSPQP
NPVSNQPQMNPYGQFVGHHQMQQSLPPPLQVILSQYPLCLFLCSNK
>AtbHLH128
MYQSSSSTSSSSQRSSLPGGGGLIRYGSAPGSFLNSVVDEVIGGGSSNARDFTGYQPSSDNFIGN
FFTGAADSSSLRSDSTTCGVNNSSDGQKQLGNNNNNNSNKDIFLDRSYGGFNEISQQHKSNDI
GGGNSSGSYSLARQRSSPADFFTYLASDKNNFSLNQPTSDYSPQGGSNGGRGHSRLKSQLSFTN
HDSLARINEVNETPVHDGSGHSFSAASFGAATTDSWDDGSGSIGFTVTRPSKRSKDMDSGLFSQ
YSLPSDTSMNYMDNFMQLPEDSVPCKIRAKRGCATHPRSIAERERRTRISGKLKKLQDLVPNM
DKQTSYSDMLDLAVQHIKGLQHQLQNLKKDQENCTCGCSEKPS
>AtbHLH129
MYPPNSSKSTAHDGGGDADTNQYDSAAGATRDFSSLGPQTHHHPPPQRQQQHQQNPNLVGHY
LPGEPSSIGFDSNASSSSSLFRHRSSPAGFYDQHLPTDPNGFSLGRPNGGYGGGGEQGPSRLKSE
LRFSSGSSSHQEHNSLPRISEVEAAAAARNGVASSSMSFGNNRTNNWDNSSSHISFTIDQPGKRS
KNSDFFTLETQYSMPQTTLEMATMENLMNIPEDSVPCRARAKRGFATHPRSIAERERRTRISGK
LKKLQELVPNMDKQTSYADMLDLAVEHIKGLQHQVESLEKGMERCTCGACKKR
>AtbHLH130
MDSNNHLYDPNPTGSGLLRFRSAPSSVLAAFVDDDKIGFDSDRLLSRFVTSNGVNGDLGSPKF

EDKSPVSLTNTSVSYAATLPPPPQLEPSSFLGLPPHYPRQSKGIMNSVGLDQFLGINNHHTKPVE
SNLLRQSSSPAGMFTNLSDQNGYGSMRNLMNYEEDEESPSNSNGLRRHCSLSSRPPSSLGMLS
QIPEIAPETNFPYSHWNDPSSFIDNLSSLKREAEDDGKLFLGAQNGESGNRMQLLSHHLSLPKSS
STASDMVSVDKYLQLQDSVPCKIRAKRGCATHPRSIAERVRRTRISERMRKLQELVPNMDKQT
NTSDMLDLAVDYIKDLQRQYKVKFLIIVEKKQRGLFSLLLN
>AtbHLH131
MVLLHHVSLSHYQNSSSLFSSSSESILCLFLVLCVMQLEQGMRPISRCYNPTAYSTTMGRSFFAG
AATSSKLFSRGFSVTKPKSKTESKEVAAKKHSDAERRRRLRINSQFATLRTILPNLVKQDKASVL
GETVRYFNELKKMVQDIPTTPSLEDNLRLDHCNNNRDLARVVFSCSDREGLMSEVAESMKAV
KAKAVRAEIMTVGGRTKCALFVQGVNGNEGLVKLKKSLKLVVNGKSSSEAKNNNNGGSLLIQ
QQ
>AtbHLH132
MMFLPTDYCCRLSDQEYMELVFENGQILAKGQRSNVSLHNQRTKSIMDLYEAEYNEDFMKSII
HGGGGAITNLGDTQVVPQSHVAAAHETNMLESNKHVDDSETLKASSSKRMMVDYHNRKKIK
FIPPDEQSVVADRSFKLGFDTSSVGFTEDSEGSMYLSSSLDDESDDARPQVPARTRKALVKRKR
NAEAYNSPERNQRNDINKKMRTLQNLLPNSHKDDNESMLDEAINYMTNLQLQVQMMTMGN
RFVTPSMMMPLGPNYSQMGLAMGVGMQMGEQQFLPAHVLGAGLPGINDSADMLRFLNHPG
LMPMQNSAPFIPTENCSPQSVPPSCAAFPNQIPNPNSLSNLDGATLHKKSRKTNR
>AtbHLH133
MNRGVLESSPVQHLTAAGNPNWWNNVSRGLRPPTPLMSHEPPSTTAFIPSLLPNFFSSPTSSSSS
SPSFPPPNSNPNFSSWLEMSDLPLDQPWSLSQLLLGGLMMGEEEKMEMMNHHHHQNQHQSY
QAKRIQNWEEQVLRHQASMKQESSNNNSYGIMSSPNSPPNKSCATIINTNEDNNNNIHSGLNLS
ECNSSEMIGSSFANKKPKLQVPSSQSTLKVRKEKLGGRIASLHQLVSPFGKTDTASVLSEAIGYI
RFLHSQIEALSLPYFGTPSRNNMMHQHAQRNMNGIFPEDPGQADPPQKNLGCCEVGQPVLSPI
LPLDHTFNIILNIYAEEIFSDLVNQLVNEYCMKRGVSLSSTDNQKSNPNEEPMKDLRSRGLCLVP
ISCTLQVGSDNGADYWAPAFGTTLQ
>AtbHLH134
MSSSRRSRQASSSSRISDDQITDLISKLRQSIPEIRQNRRSNTVSASKVLQETCNYIRNLNKEADD
LSDRLTQLLESIDPNSPQAAVIRSLING
>AtbHLH135
MSGRRSRSRQSSGTSRISEDQINDLIIKLQQLLPELRDSRRSDKVSAARVLQDTCNYIRNLHREV
DDLSERLSELLANSDTAQAALIRSLLTQ
>AtbHLH136
MSNRRSRQSSSAPRISDNQMIDLVSKLRQILPEIGQRRRSDKASASKVLQETCNYIRNLNREVD
NLSERLSQLLESVDEDSPEAAVIRSLLM
>AtbHLH137
MATFSYFQNYPHSLLDPLLFPTPHSSINLTSFIDQNHLYPLPNISTVEDISFLEYNVDKTENSGSEK
LANTTKTATTGSSSCDQLSHGPSAITNTGKTRGRKARNSNNSKEGVEGRKSKKQKRGSKEEPP
TDYIHVRARRGQATDSHSLAERVRREKISERMRTLQNLVPGCDKVTGKALMLDEIINYVQTLQ
TQVEFLSMKLTSISPVVYDFGSDLDGLILQSEMGSPEVGTSFTNAMPTTTPIFPSLLDNSVVPTH
AQVQEEGEERENFVDRSGFNNNNFCSFP
>AtbHLH138
MERYTKKNERFKAEEGKGSKKSRTFLTERERRALFNDRFFDLKNLIPNPTKGGEASIVQDGIVY
INELQRLVSELKYLVEKKKCGARHNNIEVDNKNTIYGTSKIEHPFSKNKNTFNCLIRTLRFVHHF

>AtbHLH139
MENEAFVDGELESLLGMFNFDQCSSNESSFCNAPNETDVFSSDDFFPFGTILQSNYAAVLDGSN
HQTNRNVDSRQDLLKPRKKQKLSSESNLVTEPKTAWRDGQSLSSYNSSDDEKALGLVSNTSKS
LKRKAKANRGIASDPQSLYARKRRERINDRLKTLQSLVPNGTKVDISTMLEDAVHYVKFLQLQI
KLLSSEDLWMYAPLAHNGLNMGLHHNLLSRLI
>AtbHLH140
MDDFNLRSENPNSSSTTSSSSSSFHRHKSETGNTKRSRSTSTLSTDPQSVAARDRRHRISDRFKIL
QSMVPGGAKMDTVSMLDEAISYVKFLKAQIWYHQNMLLFINDHETTSSCTYSPGAGEFGPKL
FGYDDDYAPIMDTYSQGVPLTVADSKYTPWFGSVDDEQEHVTYFKYRRATRHALRGHCNCIIG
ETEEFADQREKMEVQIEESGKNQTSPESIEADKAKQIVVLLIGPPGSGKSTFCDTAMRSSHRPWS
RICQDIVNNGKAGTKAQCLKMATDSLREGKSVFIDRCNLDREQRSEFIKLGGPEFEVHAVVLEL
PAQVCISRSVKRTGHEGNLQGGRAAAVVNKMLQSKELPKVNEGFSRIMFCYSDADVDNAVNM
YNKLGPMDTLPSGCFGEKKLDTKSQPGIMKFFKKVSALPASSSNEATNTTRKADEMTANVRVS
PVKLGSADIVPTLAFPSISTADFQFDLEKASDIIVEKAEEFLSKLGTARLVLVDLSRGSKILSLVKA
KASQKNIDSAKFFTFVGDITKLRSEGGLHCNVIANATNWRLKPGGGGVNAAIFKAAGPDLETA
TRVRANTLLPGKAVVVPLPSTCPLHNAEGITHVIHVLGPNMNPNRPDNLNNDYTKGCKTLREA
YTSLFEGFLSVVQDQSKLPKRSSQTAVSDSGEDIKEDSERNKKYKGSQDKAVTNNLESESLEDT
RGSGKKMSKGWNTWALALHSIAMHPERHENVVLEYLDNIVVINDQYPKARKHVLVLARQES
LDGLEDVRKENLQLLQEMHNVGLKWVDRFQNEDASLIFRLGYHSVPSMRQLHLHVISQDFNS
DSLKNKKHWNSFTTSFFRDSVDVLEEVNSQGKANVASEDLLKGELRCNRCRSAHPNIPKLKSH
VRSCHSQFPDHLLQNNRLVARAET
>AtbHLH141
MNSHDIDDQLEADVYSNLPSRNDSSTGRRNRNSCRSKHSETEQRRRSKINERFQSLMDIIPQNQ
NDQKRDKASFLLEVIEYIHFLQEKVHMYEDSHQMWYQSPTKLIPWRNSHGSVAEENDHPQIV
KSFSSNDKVAASSGFLLDTYNSVNPDIDSAVSTKIPEHSPVSAVSSYLRTEPSLQFVQHDFWQPK
TSCGTINCFTNELLTSDEKTSASLSTVCSQRVLNTLTEALKSSGVNMSETMISVQLSLRKREDRE
YSVAAFASEDNGNSIADEEGDSPTETRSFCNDIDHSQKRIRR
>AtbHLH142
MPLDKRQRDLPLGLSPQACFKDIVGRSVLPRIPLPELGKLYAAKLQARCLQPPPFQSLLCSHDK
ESYGKRFSRSDMRSWCAAATTTTPLGALESSQKRLLIFDQSGDQTRLLQCPFPLRFPSHAAAE
PVKLSELQGIEKAFKEDGEEFHKSDGTESEMHEDTEEINALLYSDDDYDDDCESDDEVMSTGH
SPYPNEGVCNKRELEEIDGPCKRQKLLDKVNNISDLSSLVGTESSTQLNGSSFLKDKKLPESKTI
STKEDTGSGLSNEQSKKDKIRTALKILESVVPGAKGNEALLLLDEAIDYLKLLKRDLISTEVKN
QSSTTHKSPILLLKETTWGTRNLQTDKA
>AtbHLH143
MPLDTKQQKWLPLGLNPQACVQDKATEYFRPGIPFPELGKVYAAEHQFRYLQPPFQALLSRYD
QQSCGKQVSCLNGRSSNGAAPEGALKSSRKRFIVFDQSGEQTRLLQCGFPLRFPSSMDAERGNI
LGALHPEKGFSKDHAIQEKILQHEDHENGEEDSEMHEDTEEINALLYSDDDDNDDWESDDEV
MSTGHSPFTVEQQACNITTEELDETESTVDGPLLKRQKLLDHSYRDSSPSLVGTTKVKGLSDEN
LPESNISSKQETGSGLSDEQSRKDKIHTALRILESVVPGAKGKEALLLLDEAIDYLKLLKQSLNS
SKGLNNHW
>AtbHLH144
MQNNQFPHFSDEVGDRNMHNPYASGSSYDALFPPCAKLPYHGVELQPSAVCPKNFVIFDQTYD
RSQVMYHPELTHKLMNTPSLNNLASTFQNEYVGGSYGNYGNYEQEVSSSYQEDPNEIDALLS

ADEDYEENDDNEGEEDGGDSEEVSTARTSSRDYGNTTAESCCSSYGYNNNNNNNSRKQSLSG
SASSSNNDGKGRKKMKKMMGVLRRIVPGGEQMNTACVLDEAVQYLKSLKIEAQKLGVGHFS
NQS
>AtbHLH145
MGQDRGFGFPTQRLCSLSSLALSHLGKQDLNLVSKTCGDTTDMFSTRGSYQVSTQVSQSYFDG
YCGWVHGSSHLQQQFLPPQNQCMKQVPLQVDGVISKAEEQCSQKRFLVFDQSGDQTTLLLAS
DIRKSFETLKQHACPDMKEELQRSNKDLFVCHGMQGNSEPDLKEDSEELNALLYSEDESGYCS
EEDEVTSADHSPSIVVSGREDQKTFLGSYGQPLNAKKRKILETSNESMRDAESSCGSCDNTRISF
LKRSKLSSNKIGEEKIFETVSLLRSVVPGEELVDPILVIDRAIDYLKSLKMEAKNREA
>AtbHLH146
MERQIINRKKRVFSLEPNKNPSAVFTRKYTSHLVPALKKLNMNKNSSKQTVKHEVDMALALSA
QEFAWSRFLLQKLSSSSNPTTTTSSSSDGIRILERPDKEGGNEEGGIEERLRELKKLLPGGEEMN
VEEMLSEIGNYIKCLELQTIALKSIVQDST
>AtbHLH147
MESISPVSNQLLQPTTTSSNSDRSRRKRKKKSSPSSVEKSPSPSISLEKWRSEKQQQIYSTKLVHA
LRELRISQQPSSSSSSSIPRGGRAVREVADRALAVAARGKTLWSRAILSKAVKLKFRKHKRQRIS
NPTTTTLTTGSIRSKKQRATVLRLKAKGLPAVQRKVKVLSRLVPGCRKQSLPVVLEETTDYIAA
MEMQIRTMTAILSAVSSSPPPPTPGHEGGQTHMLG
>AtbHLH148
MASLISDIEPPTSTTSDLVRRKKRSSASSAASSRSSASSVSGEIHARWRSEKQQRIYSAKLFQALQ
QVRLNSSASTSSSPTAQKRGKAVREAADRALAVSARGRTLWSRAILANRIKLKFRKQRRPRAT
MAIPAMTTVVSSSSNRSRKRRVSVLRLNKKSIPDVNRKVRVLGRLVPGCGKQSVPVILEEATDY
IQALEMQVRAMNSLVQLLSSYGSAPPPI
>AtbHLH149
MVESLFPSIENTGESSRRKKPRISETAEAEIEARRVNEESLKRWKTNRVQQIYACKLVEALRRVR
QRSSTTSNNETDKLVSGAARREIRDTADRVLAASARGTTRWSRAILASRVRAKLKKHRKAKKST
GNCKSRKGLTETNRIKLPAVERKLKILGRLVPGCRKVSVPNLLDEATDYIAALEMQVRAMEAL
AELLTAAAPRTTLTGT
>AtbHLH150
MSSEQGNGSNPSTSPEVEGTKTIPFRRRLQRGQRVFAPKLMEALRRSRVSSEEAPVRHLSRRWR
ATTAQKVYSLKLYDALQRSRRSATVRDTADKVLATTARGATRWSRAILVSRFGTSLRRRRNTKP
ASALAAAIRGSGGSGRRRKLSAVGNRVRVLGGLVPGCRRTALPELLDETADYIAALEMQVRAM
TALSKILSELQPSTNLGSAL
>AtbHLH151
MGVTLEGQRKESIWVLMRRQRARRALVKKIMIRPRKSVEASRRPCRAIHRRVKTLKELVPNTK
TSEGLDGLFRQTADYILALEMKVKVMQTMVQVLTETNCV
>AtbHLH153
MEFSRDAGMMMENKRNVCSLGESSIKRHKSDLSFSSKERKDKVGERISALQQIVSPYGKTDTA
SVLLDAMHYIEFLHEQVKVCSSIPSMIHSSLSEFPCSFVQVLSAPYLQTVPDATQEELEQYSLRN
RGLCLVPMENTVGVAQSNGADIWAPVKTPLSPAFSVTSQSPFR
>AtbHLH154
MEYSRDSAEMMMETKRNVYSLEDNKIKRHKSSDLSFSSKERKDKLAERISALQQLVSPYGKT
DTASVLLEGMQYIQFLQEQVKVLSAPYLQATPSTTEEEVEEYSLRSKGLCLVPLEYTSEVAQTN
GADIWAPVKTPTSSHAFNLSSSNSPFQ

>AtbHLH155
MGSTSQEILKSFCFNTDWDYAVFWQLNHRGSRMVLTLEDAYYDHHGTNMHGAHDPLGLAVA
KMSYHVYSLGEGIVGQVAVSGEHQWVFPENYNNCNSAFEFHNVWESQISAGIKTILVVAVGPC
GVVQLGSLCKVNEDVNFVNHIRHLFLALRDPLADHAANLRQCNMNNSLCLPKMPSEGLHAE
AFPDCSGEVDKAMDVEESNILTQYKTRRSDSMPYNTPSSCLVMEKAAQVVGGREVVQGSTCG
SYSGVTFGFPVDLVGAKHENQVGTNIIRDAPHVGMTSGCKDSRDLDPNLHLYMKNHVLNDTS
TSALAIEAERLITSQSYPRLDSTFQATSRTDKESSYHNEVFQLSENQGNKYIKETERMLGRNCES
SQFDALISSGYTFAGSELLEALGSAFKQTNTGQEELLKSEHGSTMRPTDDMSHSQLTFDPGPEN
LLDAVVANVCQRDGNARDDMMSSRSVQSLLTNMELAEPSGQKKHNIVNPINSAMNQPPMAE
VDTQQNSSDICGAFSSIGFSSTYPSSSSDQFQTSLDIPKKNKKRAKPGESSRPRPRDRQLIQDRIK
ELRELVPNGSKCSIDSLLERTIKHMLFLQNVTKHAEKLSKSANEKMQQKETGMQGSSCAVEVG
GHLQVSSIIVENLNKQGMVLIEMLCEECGHFLEIANVIRSLDLVILRGFTETQGEKTWICFVTEV
GSRITQFMKEIPKQIKSQNSKVMQRMDILWSLVQIFQPKANEKG
>AtbHLH156
MGVLLREALRSMCVNNQWSYAVFWKIGCQNSSLLIWEECYNETESSSNPRRLCGLGVDTQGN
EKVQLLTNRMMLNNRIILVGEGLVGRAAFTGHHQWILANSFNRDVHPPEVINEMLLQFSAGIQ
TVAVFPVVPHGVVQLGSSLPIMENLGFVNDVKGLILQLGCVPGALLSENYRTYEPAADFIGVPV
SRIIPSQGHKILQSSAFVAETSKQHFNSTGSSDHQMVEESPCNLVDEHEGGWQSTTGFLTAGEVA
VPSNPDAWLNQNFSCMSNVDAAEQQQIPCEDISSKRSLGSDDLFDMLGLDDKNKGCDNSWGV
SQMRTEVLTRELSDFRIIQEMDPEFGSSGYELSGTDHLLDAVVSGACSSTKQISDETSESCKTTLT
KVSNSSVTTPSHSSPQGSQLFEKKHGQPLGPSSVYGSQISSWVEQAHSLKREGSPRMVNKNET
AKPANNRKRLKPGENPRPRPKDRQMIQDRVKELREIIPNGAKCSIDALLERTIKHMLFLQNVSK
HSDKLKQTGESKIMKEDGGGATWAFEVGSKSMVCPIVVEDINPPRIFQVEMLCEQRGFFLEIAD
WIRSLGLTILKGVIETRVDKIWARFTVEASRDVTRMEIFMQLVNILEQTMKCGGNSKTILDGIKA
TMPLPVTGGCSM
>AtbHLH157
MGSEYKHILKSLCLSHGWSYAVFWRYDPINSMILRFEEAYNDEQSVALVDDMVLQAPILGQGIV
GEVASSGNHQWLFSDTLFQWEHEFQNQFLCGFKILIRQFTYTQTIAIIPLGSSGVVQLGSTQKIL
ESTEILEQTTRALQETCLKPHDSGDLDTLFESLGDCEIFPAESFQGFSFDDIFAEDNPPSLLSPEMI
SSEAASSNQDLTNGDDYGFDILQSYSLDDLYQLLADPPEQNCSSMVIQGVDKDLFDILGMNSQ
TPTMALPPKGLFSELISSSLSNNTCSSSLTNVQEYSGVNQSKRRKLDTSSAHSSSLFPQEETVTSR
SLWIDDDERSSIGGNWKKPHEEGVKKKRAKAGESRRPRPKDRQMIQDRIKELRGMIPNGAKCS
IDTLLDLTIKHMVFMQSLAKYAERLKQPYESKLVKEKERTWALEVGEEGVVCPIMVEELNREG
EMQIEMVCEEREEFLEIGQVVRGLGLKILKGVMETRKGQIWAHFIVQAKPQVTRIQVLYSLVQL
FQHHTKHDDLLS
>AtbHLH158
MASADKLINTDVPEKDVFAFHFLQSLSNLRKQNPFDTPDQKNYRVRKIKKAAYVSMARAAGG
SSRLWSRALLRRADKDDNKIVRFSRRKWKISSKRRRSNQRAPVVEEAAERLRNLVPGGGGME
TSKLMEETAHYIKCLSMQVKVMQCLVDGLSPK
>AtbHLH159
MQPTSSMNEEFLKKWQMGLQIFRPSIDNTSVHERKKAIKLSADVAMASLRKGTTCWSRALIEK
TATEDNFLVRQMLSGIKAETLINKKLPKKTVCHRKIVRRSKKILRRKSKSASEEAAAKAKRLVK
RRTQGLRNVVPGGELMSNDVLLLQETLDYIVSLQTQVNVMRSIVDAAEAEIER
>AtbHLH160

MSSQPNHQTSISSLLHDRLHIPPAETIVEKESAEKDTCQSQRKRKEPVLHEVDGSSSGAAKKQD
HNAKERLRRMRLHASYLTLGTLLPDHSSSSSKVLFSLLLLQVRYVLLVVELYITFLADWQKKW
SAPSIIDNVITYIPKLQNEVGELTLRKQKLVELERRGPSIRAISVLELGESGYEAVVQICLKKENE
DEFSNLLHVMEVQGLSVLSASTSQVCREQRVVCYNFHVKMDEKPCEGDDYITVLKNNIISSLR
DNTKCK

>AtbHLH161
MSSRKSRSRQTGASMITDEQINDLVLQLHRLLPELANNRRSGKVSASRVLQETCSYIRNLSKEV
DDLSERLSQLLESTDSAQAALIRSLLMQ

>AtbHLH162
MEPSHSNTGQSRSVDRKTVEKNRRMQMKSLYSELISLLPHHSSTEPLTLPDQLDEAANYIKKLQ
VNVEKKRERKRNLVATTTLEKLNSVGSSSVSSSVDVSVPRKLPKIEIQETGSIFHIFLVTSLEHKF
MFCEIIRVLTEELGAEITHAGYSIVDDAVFHTLHCKVEEHDYGARSQIPERLEKIVNSVH

>AtbHLH163
MSSRRSSRSRQSGSSRISDDQISDLVSKLQHLIPELRRRRSDKVSASKVLQETCNYIRNLHREVD
DLSDRLSELLASTDDNSAEAAIIRSLLNY

>AtbHLH164
MSNRRSRQTSNASRISDDQMIDLVSKLRQFLPEIHERRRSDKVSASKVLQETCNYIRKLHREVD
NLSDRLSQLLDSVDEDSPEAAVIRSLLM

>AtbHLH165
MEETLATPDATRRSLSPSCSATVKSRAAGFERRTKRRLSETNASVREDREEAEEEEDEVKEKIEA
LQRIIPGGAALGVDALFEETAGYILSLQCQIKTIKVLTSFLQRIDQEDMKFGG

>AtbHLH166
MEKTLATSHTKRSSPPSPSSAVNTSSTGFNRRTRQRLSDATASVSETDVEDEDEDEEGVEEKIEA
LQTIVPGGTELGVDALFEETASYILALQCQINAIKVLTTFLERCEKKDMKFGG

>AtbHLH167
MMNTYNMVKQEFIKKWINTLHMLDSSIEHPLNVTERKNAIRLSSDLAMAAARNGSTVWSRAL
ISRSGNKTANKPMARRILKKARNRMKNRCNILRRNGNFTAKTWVRKRTDLLKSLVPGGELIDD
KDYLIRETLDYIVYLRAQVDVMRTVAAVDLFTRNLTNDRRNK

>AtbHLH168
MRTLKTQTTRGRRRANVSSRTRVLHTCCGNGSSDGGKTVMEKLLALKSLLPPPVNVGGGETE
ELFQETAEYIVKLRTQVVVLKKLIEIYDNSSDQKKDVVL

>AtbHLH169
MFLFFTMRILKTQRSRGGRRTSKKFGNRRTSGGEKFSEKLQALKSLLPPPSKMTEQSRQDAYVE
EDSSVGETEQLFQETADYIVRLRGQVVVLQKLIEIYGSSDQKEDNFVS

>AtbHLH170
MERQIINKRKRVFSLQPNKNPKAVFARRYVSHLVPALKKINMNKSSSKTNKQSLEQTVKHEVD
MAFALSAQEFAWSRFLQQKLLSSPYDDPISTSSSPSEILERSSKRQGGEKHQDSDEEEEGGEIKK
RLKELQKLLPGGEEMNMEEILSEIGSYIVCLELQMIVLKSIVQDNTS
